# Integrated redox-active reagents for photoinduced regio- and stereoselective fluorocarboborylation

**DOI:** 10.1038/s41467-020-16477-1

**Published:** 2020-05-22

**Authors:** Weigang Zhang, Zhenlei Zou, Wenxuan Zhao, Shuo Lu, Zhengguang Wu, Mengjun Huang, Xiaochen Wang, Yi Wang, Yong Liang, Yi Zhu, Youxuan Zheng, Yi Pan

**Affiliations:** 1 State Key Laboratory of Coordination Chemistry, Jiangsu Key Laboratory of Advanced Organic Materials, School of Chemistry and Chemical Engineering, Nanjing University, 210023 Nanjing, China; 2 Key Laboratory of Synthetic and Biological Colloids, Ministry of Education, School of Chemical and Material Engineering, Jiangnan University, 214122 Wuxi, Jiangsu China

**Keywords:** Reaction mechanisms, Synthetic chemistry methodology, Photochemistry

## Abstract

Vinylboronates and alkylboronates are key components in variegated transformations in all facets of chemical science. The synthesis of vinylboronates and alkylboronates suffers from step-tedious and poor stereoselective procedures. We have developed a regulated radical difunctionalization strategy for the construction of fluorine-containing vinylboronates and alkylboronates with an integrated redox-active reagent IMDN-SO_2_R_F_. This bench-stable imidazolium sulfonate cationic salt offers a scalable and operational protocol for the fluoroalkylation-borylation of unsaturated hydrocarbons in a high regio- and stereoselective manner. The products can be further transformed into valuable fluorinated building blocks.

## Abstract (web summary)

The synthesis of vinylboronates and alkylboronates often suffers from step-tedious and poorly stereoselective procedures. Here, the authors report a bench-stable redox-active reagent for the radical difunctionalization of alkenes and alkynes affording fluorine-containing vinylboronates and alkylboronates.

## Introduction

**Fig. 1:**
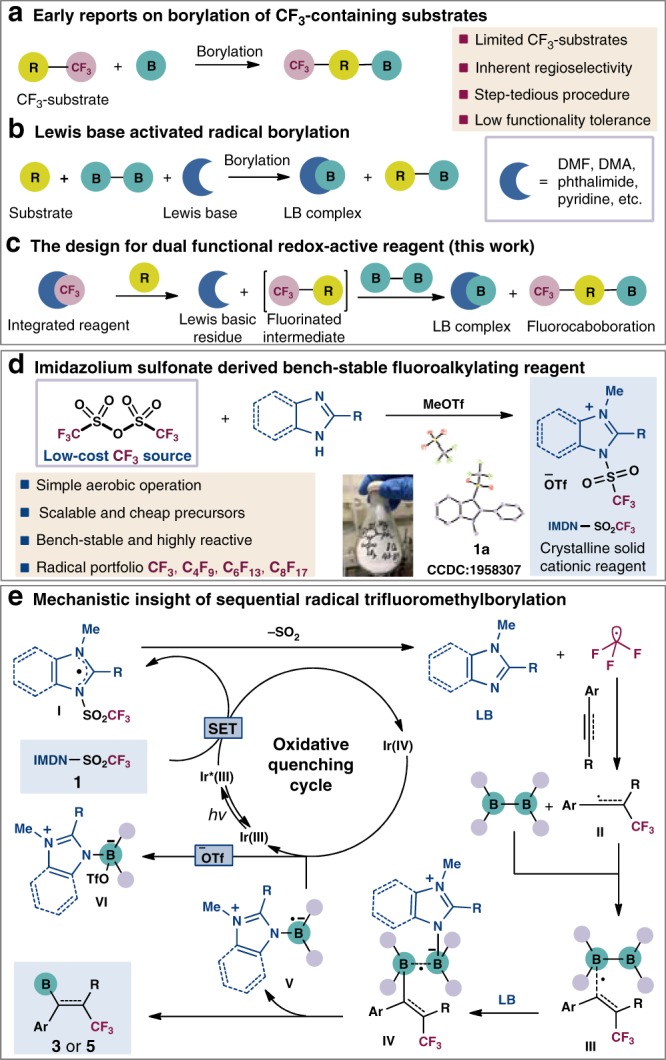
Origin of the reaction design. **a** Early reports on borylation of CF_3_-containing substrates. **b** Lewis base-activated radical borylation. **c** The design for dual functional redox-active reagent. **d** Imidazolium sulfonate-derived bench-stable fluoroalkylating reagent. **e** Mechanistic insight of sequential radical trifluoromethylborylation.

Difunctionalization of alkenes and alkynes has been widely explored for rapid diversification of double/triple bonds^1–6^. Traditional transition metal-catalyzed difunctionalization methods have been well-developed to control the regioselectivity and stereoselectivity^7,8^. Along these lines, cascade radical addition of unsaturated hydrocarbons in the absence of metallo-intermediate has been realized through careful manipulation of the radical reactivities^9–11^. A single process to achieve radical difunctionalization with extensive functionality tolerance, especially fluorine-containing moieties, is of great value in altering the physical and biological properties of the unsaturated hydrocarbons^4,12–18^. Studer and co-workers^19^ have reported a radical 1,2-trifluoromethylboration of unactivated alkenes using gaseous CF_3_I. Meanwhile, the direct 1,2-fluoroalkylboration of alkynes has also been explored^20–22^. The consequential vicinial vinylboronates, which can be readily transformed to a myriad of fluorine-containing building blocks, have been seldom realized. The only two existing approaches of trifluoromethylated vinylboronates were derived from fluorinated alkynes^23^ and oxiranes^24^. The inaccessibility of those pre-functionalized precursors and operationally tedious procedures prohibited the stepwise fluorination-borylation strategies from practical use (Fig. 1a). Thus, the development of regioselective installation of fluoroalkyl and boronated functionalities to unactivated hydrocarbons in the absence of transition-metal catalysts represents great challenge.

Due to the complexity of radical process incorporating C–C and C–B bonds formation, several issues need to be addressed, including the reactivities of carbon and boron-centered radicals, the regioselectivity of the radical additions to unsymmetrical alkynes, and stereoselectivity towards *E*/*Z* vinylboronates. Typically, a radical borylation process^25–32^ often employs Lewis basic solvents/mediators such as DMAc^33,34^, DMF^19,35^, phthalimide^36^, and pyridine^37,38^ for the activation of diboron reagents via homolytic cleavage of B–B bonds (Fig. 1b). However, the exogenous Lewis base-activated diboron species inevitably deplete CF_3_ radicals that generated promptly from the trifluromethylating reagents, unable to engage in the desired trifluoromethylborylation sequence (for DFT calculation details, see Supplementary Fig. 18). Inspired by recent radical-induced difunctionalization strategies^39,40^, we intend to design an integrated reagent that progressively releases CF_3_ radical for alkene/alkyne addition, and the endogenous Lewis basic residue subsequently activates the B–B bond for further borylation (Fig. 1c).

Trifluoromethanesulfinate-derived fluorinating reagents have been devised and adopted for direct functionalization of alkenes, alkynes, and arenes^41^. In contrast, the highly hydroscopic and corrosive trifluoromethanesulfonic anhydride (Tf_2_O) as a trifluoromethyl source is rarely explored. For its strong electrophilic nature, triflic anhydride is commonly used as an alcohol and amine protecting agent^42–44^. Qing and co-workers^45^ have described a triflated pyridine intermediate (Tf^−^Py^+^·OTf^−^) that generated in situ for trifluoromethylated arenes and alkynes. However, the strong electron-withdrawing triflate-derived pyridinium complex is preferably dissociated and unattainable in solid or liquid phase. We speculated that a more basic *N-*heterocycle such as imidazole could harness the highly reactive Tf_2_O to assemble a bench-stable redox-active reagent. The positive charge of the resulting imidazolium trifluoromethanesulfonate can be delocalized on both nitrogen. Through the cleavage of the stabilized N–S bond (BDE ≈ 70 kcal mol^−1^)^46^, this cationic complex undergoes SET process to generate CF_3_SO_2_ radical. Meanwhile, as a Lewis base, the imidazole residue can further activate the diboron reagents towards homolytic cleavage of the diboron reagent^20,34–39^. Herein, we have synthesized a dual functional reagent IMDN-SO_2_CF_3_ **1a–1g**, a scalable and air-stable crystalline salt for a sequential radical fluoroalkylation-borylation of unsaturated hydrocarbons (Fig. 1d). First, under the irradiation, Ir(III)* can reduce the cationic reagent **1** to a neutral radical **I** and releases CF_3_ radical, SO_2_, and imidazole. Then the addition of ·CF_3_ to the alkyne regioselectively furnishes vinylic radical **II**. Subsequent addition of vinyl radical **II** to B_2_cat_2_ affords a *Z*-vinyl diboron radical **III**. The control of stereoselectivity is governed by steric repulsion between the trifluoromethyl group and the boronates. The following activation of diboron by the Lewis basic imidazole forms a highly reactive B–N heteroleptic intermediate **IV**, which leads to the carboborylation product **3** and imidazole-stabilized boryl radical **V**. Finally, photo-oxidation of **V** followed by coupling with ^−^OTf affords boryl imidazolium salt **VI** and regenerates Ir(III) (Fig. 1e). These proposed intermediates and selectivities are supported by DFT calculations (see Supplementary Figs. 16–19) This photoinduced cascade radical difunctionalization offers a concise and applicable protocol for constructing highly regio- and stereoselective fluorine-substituted vinylboronates and vicinal fluoroalkyl boronates.

## Results

### Reaction optimization

To validate the above hypothesis, we selected phenylacetylene (**2a**) as pilot substrate to test the trifluoromethylborylation reaction (Table 1). After extensive screening of conditions (see Supplementary Tables 1 and 2), we found that when using 2.5 equivalents of IMDN-SO_2_CF_3_ (**1a**) (*E*_1/2_^red^ = −1.385 V vs SCE), 2 mol% of *fac*-Ir(ppy)_3_ (*E*_1/2_^IV/III*^ = −1.73 V vs SCE)^47^, 2.5 equivalents of B_2_cat_2_ in a mixed solvent of MeCN and EtOAc (1:3 v/v) at room temperature under the irradiation of 30 W blue LEDs, the vinylboronate product **3a** could be obtained in 82% yield (determined by ^19^F NMR) with over 20:1 *Z*/*E* ratio. Different imidazolium sulfonate reagents **1b**–**1h** were then examined (Table 1). The yield of **3a** descended when the benzoimidazolium reagents **1b** and **1c** were used (entries 2 and 3). The counterion was found to be important for this transformation, as evidenced by the low yield (53%) obtained when using BF_4_^−^ salt (**1d**, entry 4). The reaction proceeded with 2-phenylimidazole reagents **1e** and **1f** in 76% and 60% yield, respectively (entries 5–6). The dimethylated reagent **1g** resulted in a lower conversion (entry 7). The electroneutral reagent **1h** failed to produce the desired product under irradiation, which may due to the low reduction potential (*E*_1/2_^red^ = −1.808 V vs SCE) (entry 8). This result validated the precedential presumption that the cationic reagent can serve as a better electron acceptor to furnish *N*-centered neutral radicals^48–51^. Other diboron reagents, such as bis(pinacolato)diboron (B_2_pin_2_) and bis(neopentylglycolato)-diboron (B_2_neop_2_), did not provide the corresponding borylated products (entries 9–10). Addition of excess bases such as imidazole and pyridine resulted in much lower yields (entries 11–12).

**Table 1:** Optimization of the reaction conditions.


Entry	Variation from the conditions	Yield of 3a^a^ (%)	*Z*:*E* of 3a^b^
1	None	82 (65)^c^	>20:1
2	**1b** instead of **1a**	67	>20:1
3	**1c** instead of **1a**	69	>20:1
4	**1d** instead of **1a**	53	>20:1
5	**1e** instead of **1a**	76	>20:1
6	**1f** instead of **1a**	60	>20:1
7	**1g** instead of **1a**	32	>20:1
8	**1h** instead of **1a**	0	—
9	B_2_pin_2_ instead of B_2_cat_2_	0	—
10	B_2_neop_2_ instead of B_2_cat_2_	0	—
11	2.0 equiv of pyridine	58	>20:1
12	2.0 equiv of 1-methylimidazole	47	>20:1

^a^Yield determined by ^19^F NMR spectroscopy using trifluoromethoxybenzene as an internal standard. ^b^ The *Z*/*E* ratio was determined by ^19^F NMR. ^c^ Isolated yield.

### Substrate scope with respect to the alkynes

**Fig. 2:**
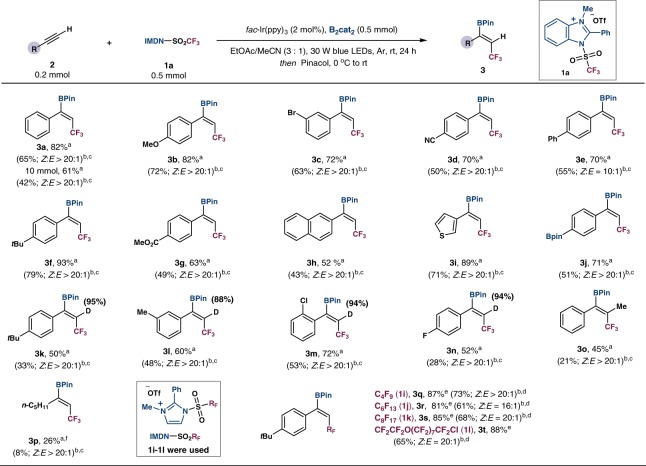
Substrate scope of the alkynes. ^a^Crude yields determined by ^19^F NMR spectroscopy using benzotrifluoride or trifluoromethoxybenzene as an internal standard. ^b^Values in parentheses are of isolated yields. ^c^The *E*/*Z* ratio was determined by ^19^F NMR. ^d^The *E*/*Z* ratio was determined by ^1^H NMR. ^e^Crude yields determined by ^1^H NMR spectroscopy using dibromomethane as an internal standard. ^f^2.0 mmol of **2p** was used.

Using 2 mol% of *fac*-Ir(ppy)_3_, IMDN-SO_2_CF_3_ (**1a**) (2.5 equiv), and B_2_cat_2_ (2.5 equiv) at ambient temperature, a range of alkyens underwent fluoroalkylation-borylation with good efficiency. As shown in Fig. 2, the reaction can be performed at a gram scale to give **3a** in 61% yield and high stereoselectivity. Aromatic alkynes with electro-donating or electro-withdrawing substituents afford the desired products **3b**–**3j** in good to excellent yields (60–93%) with high regio- and stereoselectivity (*Z*:*E* > 20:1).

Functionalities including halides (**3b**, **3m**, **3n**), nitrile (**3d**), ester (**3g**), and boronate (**3j**) are tolerated. Naphthyl- and thienyl-substituted alkynes also readily transformed into the *Z*-products **3h** and **3i** in good yields. The reaction could also be applied to alkynyl deuterium to produce the (*Z*)-selective deuterated vinylboronates **3k**–**3n** in 52–72% yields. An attempt of more challenging internal alkyne substrate resulted in the tetrasubstituted olefin in high regio- and stereoselectivity (**3o**, 45%). For further investigation of the reaction scope, different fluoroalkylating reagents **1i**–**1l** have been synthesized and applied to the standard cabonborylation conditions. Perfluoro-butyl (**1i**), hexyl (**1j**), and octanyl (**1k**) reagents could furnish the corresponding products **3q**–**3s** in good yields (81–87%). Using a perhalogenated ether-derived sulfonate (**1l**), the vinylboronate **3t** was formed in high yield. To demonstrate the scalability of such radical carboborylation protocol, the reaction was carried out on 10 mmol scale to afford **3a** in 61% yield with equally high *Z*/*E* ratio. Under the standard reaction conditions, alkyl-substituted alkynes could not transform to the desired products. DFT calculations illustrate that the energy barrier of CF_3_ radical addition to aliphatic alkynes is higher than that to aromatic alkynes. Furthermore, a competing pathway of CF_3_ radical addition to B_2_cat_2_ leads to other trifluoromethylated products. Therefore, an excess amount of alkyl alkyne substrate is needed to facilitate the main reaction pathway. By using four equivalents of the alkyne, the borylated product **3p** can be obtained in 26% yield. For internal aliphatic alkynes, the computed barrier with the CF_3_ radical is much higher than that for the reaction of B_2_Cat_2_ with the CF_3_ radical. Therefore, no desired product is obtained using internal aliphatic alkynes as substrate.

### Substrate scope with respect to the olefins

**Fig. 3:**
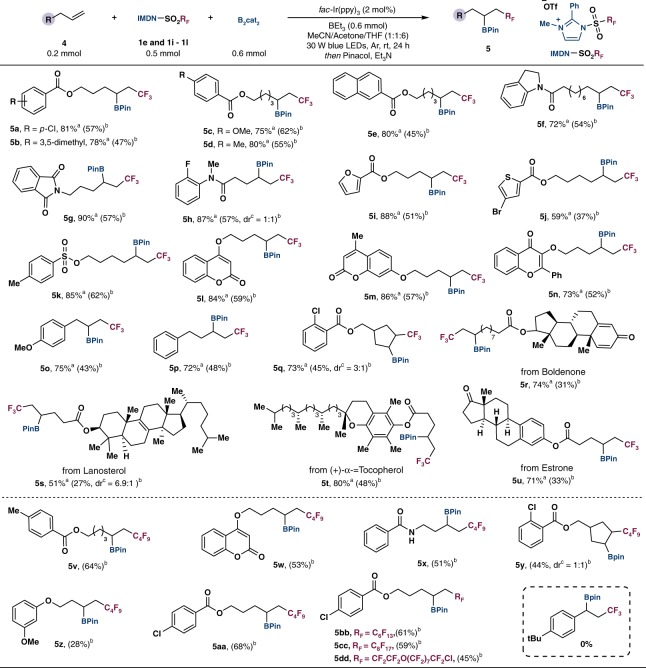
Substrate scope of the olefins. ^a^Crude yields determined by ^19^F NMR. ^b^Values in parentheses are isolated yields. ^c^The diastereomeric ratio determined by ^1^H NMR.

The *α*-fluoroalkylated boronates are also useful fluorine-containing synthons for further elaboration. By slight variation of the standard reaction conditions (see Supplementary Tables 3–10), we have extended this carbonborylation protocol to a range of unactivated alkenes (Fig. 3). Using IMDN-SO_2_CF_3_ (**1e**), alkenes bearing ester and amide functionalities underwent radical 1,2-carbonboration to afford trifluoromethylated boronates (**5a**–**5h**) in good yields. Heteroaryl (**5i**–**5j**), sulfonyl (**5k**), and oxygenated alkyl groups (**5l**–**5n**) at various positions of the alkenes were also found effective. Cyclic alkenes could also transform into the desired products **5q** and **5y**. Noteworthy, biorelevant molecules, such as boldenone, lanosterol, (+)-*α*-tocopherol, and estrone-derived terminal alkenes afforded *β*-trifluoromethylboronates (**5r**–**5u**) in good yields. Additionally, fluoroalkyl radicals including ·C_4_F_9_ (**1i**), ·C_6_F_13_ (**1j**), ·C_8_F_17_ (**1k**), and ·CF_2_CF_2_O(CF_2_)_7_CF_2_Cl (**1l**) were successfully stitched to unactivated olefins to afford fluoroalkylborylated products in moderate yields (**5v**–**5dd**). The reaction with styrene failed to afford the desired product due to inert reactivity of benzylic radical.

### Synthetic applications

**Fig. 4:**
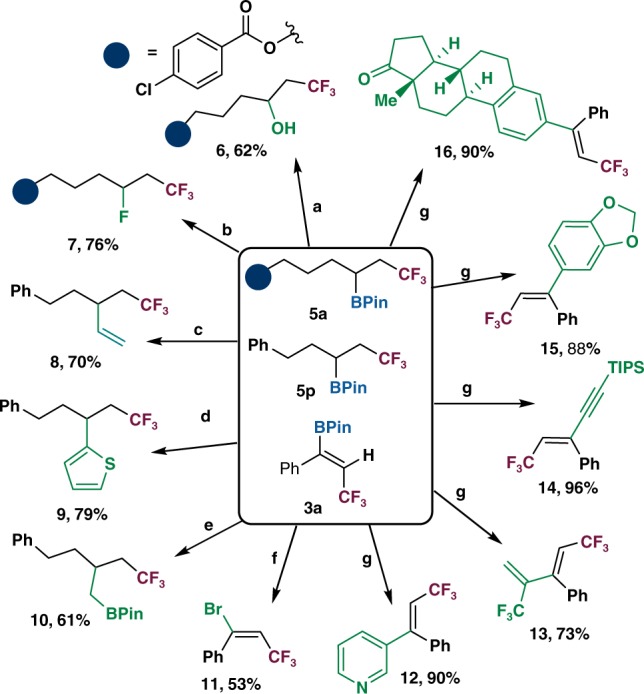
Further transformations. ^a^H_2_O_2_ (30%), NaOH (3 M), THF, 0 °C to rt. ^b^Selectfluor, AgNO_3_, TFA, H_3_PO_4_, DCM/H_2_O, 50 °C. ^c^Vinylmagnesium bromide, I_2_, THF, −78 °C to 0 °C. ^d^*n*-BuLi, NBS, thiophene, THF, −78 °C. ^e^*n*-BuLi, dibromomethane, THF, −78 °C to rt. ^f^CuBr_2_, MeOH, 80 °C. ^g^Pd(PPh_3_)_4_ (5 mol%), Cs_2_CO_3_, toluene, H_2_O, 80 °C.

The synthetic utility of the method was demonstrated in a number of transformations of the highly functionalized alkylboronates and alkenylboronates^20,52,53^ (Fig. 4). Oxidation of *β*-CF_3_ boronate **5a** afforded hydroxylated product **6** in 62% yield. Silver-catalyzed radical deboronofluorination of **5a** in aqueous solution provided the alkyl fluoride **7** in 76% yield. Vinylation, oxidative coupling, and homologation of **5p** afforded functionalized products **8**–**10** in good yields. Halogenation of vinylboronic ester **3a** resulted in the formation of *β*-CF_3_-vinyl bromide **11**(53%). Palladium-catalyzed Suzuki–Miyaura cross-coupling of **3a** with (hetero)aryl iodides afforded the corresponding trisubstituted alkenes **12** (90%) and **15** (88%). Olefination and alkynylation using vinyl bromide or alkynyl bromide also proceeded smoothly to generate **13** and **14** in 73% and 96% yields, respectively. The coupling of **3a** with bioactive estrone-derived triflate produced the corresponding product **16** with high stereoselectivity.

## Discussion

In summary, we have described an air-stable redox-active reagent IMDN-SO_2_R_F_ **1** with high reactivity and scalability. A key design feature of this dual functional imidazolium sulfonate reagent is the cationic nature that favors the progressive formation of fluoroalkyl radicals by SET reduction under photocatalytic conditions. Meanwhile, the in situ-generated Lewis basic imidazole residue promotes the B–B bond cleavage. The integrated reagent is applicable to regulate the reaction sequence of carbon and boron-centered radicals to access various fluorine-bearing vinylboronates and alkylboronates with high stereo- and regioselectivities. Further study of this reagent is underway in our laboratory.

## Methods

### General procedure for the synthesis of imidazolium salts 1

To a one-necked 1000 mL flask equipped with a magnetic stirrer, the corresponding imidazole (100 mmol), Et_3_N (150 mmol), and 600 mL DCM were added. The flask was then cooled in an ice bath, and 130 mmol (36.8 g) (CF_3_SO_2_)_2_O was bubbled into the flask slowly. The mixture was stirred at room temperature for 2 h and evaporated in vacuo, quenched with water, and extracted with ethyl acetate (300 mL × 3). The combined organic layers were dried over Na_2_SO_4_, filtered, and concentrated. The product was purified by flash column chromatography on silica gel with *n*-pentane/ethyl acetate as eluent to give the imidazolyl sulfonamide. Under argon, to a solution of the imidazolyl sulfonamide in dried DCM (400 mL) was added dropwise MeOTf (or Me_3_OBF_4_) (130 mmol) at 0 °C. Then, the mixture was stirred at room temperature for 12 h. (If EtOTf is used, the reaction is refluxed for 24 h.) After that, the mixture was concentrated under rotary evaporation to give a white solid (or a viscous liquid) crude product, to which Et_2_O (300 mL) was added. With vigorous stirring, a solid precipitate was formed and washed with Et_2_O (200 mL × 3) and dried in vacuo to yield the imidazolium salt **1** as a white solid.

### General procedure for the synthesis of vinylboronates 3

Under argon, to a solution of **1** (0.50 mmol, 2.5 equiv), B_2_Cat_2_ (0.5 mmol, 2.5 equiv) and *fac*-Ir(ppy)_3_ (2 mol%) in MeCN:EtOAc (1:3) (3 mL) was added corresponding alkynes **2** (0.2 mmol) at room temperature. After that, the tube was exposed to 30 W blue LEDs at room temperature about 30 h until the reaction was completed as monitored by TLC or GC-MS analysis. A solution of pinacol (236 mg, 2 mmol) in MeCN (1.0 mL) was added dropwise to the mixture at 0 °C. After 1 h, saturated ammonium chloride solution (15 mL) was added and the aqueous layer was extracted with hexane (3 × 15 mL). The combined organic layers were dried over Na_2_SO_4_, filtered, and concentrated. The product was purified by flash column chromatography on silica gel with *n*-pentane/ethyl acetate as eluent to give the vinylboronates **3**.

### General procedure for the synthesis of alkylboronates 5

Under argon, to a solution of **1** (0.50 mmol, 2.5 equiv), B_2_Cat_2_ (0.6 mmol, 3.0 equiv) and *fac*-Ir(ppy)_3_ (2 mol%) in 1:1 MeCN/acetone (0.2 mL) was added Et_3_B (0.6 mmol, 3.0 equiv, 1 mol/L in THF) and corresponding alkenes **4** (0.2 mmol) at room temperature. After that, the tube was exposed to 30 W blue LEDs at room temperature for 30 h until the reaction was completed as monitored by TLC or GC-MS analysis. A solution of pinacol (142 mg, 1.2 mmol) in Et_3_N (1.1 mL) was added to the mixture. After 1 h, the reaction mixture was evaporated in vacuo. The product was purified by flash column chromatography on silica gel with *n*-pentane/ethyl acetate as eluent to give the alkylboronates **5**.

## Supplementary information

Supplementary Information

Peer Review File

Description of Additional Supplementary Files

Supplementary Data 1

## Data Availability

The authors declare that the main data supporting the findings of this study, including experimental procedures and compound characterization, are available within the article and its Supplementary Information files. X-ray structural data of compound **1a** are available free of charge from the Cambridge Crystallographic Data Center under the deposition number CCDC 1958307. These data can be obtained free of charge from The Cambridge Crystallographic Data Center via www.ccdc.cam.ac.uk/data_request/cif.
