# Supplementary material for: Integrated redox-active reagents for photoinduced regio- and stereoselective fluorocarboborylation
Source: Nat Commun. 2020 May 22;11:2572. doi: 10.1038/s41467-020-16477-1 (PMC7244735; doi:10.1038/s41467-020-16477-1)
Supplement: Supplementary file 1 — Supplementary Information [file 41467_2020_16477_MOESM1_ESM.pdf]

Supplementary Information

Integrated redox-active reagents for photoinduced regio- and stereoselective fluorocarboborylation

Zhang et al.

Supplementary Methods

General Methods. All reactions were performed in flame-dried glassware with magnetic stirring bar and sealed with a rubber septum. The solvents were distilled by standard methods. Reagents were obtained from commercial suppliers and used without further purification unless otherwise noted. Silica gel column chromatography was carried out using silica Gel 60 (230–400 mesh). Analytical thin layer chromatography (TLC) was done using silica Gel (silica gel 60 F254). TLC plates were analyzed by an exposure to ultraviolet (UV) light and/or submersion in phosphomolybdic acid solution or submersion in $KMnO_4$ solution or in I_2. NMR experiments were measured on a Bruker AVANCE III-400 or 500 spectrometer and carried out in chloroform-*d* ($CDCl_3$) or acetonitrile-d_3 (CD_3CN). ^{1}H NMR and ^{13}C NMR spectra were recorded at 400 MHz or 500 MHz and 100 MHz or 125 MHz spectrometers, respectively. ^{19}F NMR spectra were recorded at 376 MHz or 470 MHz spectrometers with 5mm BBFO SMART probe. Chemical shifts are reported as δ values relative to internal TMS (δ 0.00 for ^{1}H NMR), chloroform (δ 7.26 for ^{1}H NMR), acetonitrile (δ 1.94 for ^{1}H NMR), chloroform (δ 77.00 for ^{13}C NMR), and acetonitrile (δ 1.32 or 118.26 for ^{13}C NMR) in parts per million (ppm). ^{19}F NMR is auto-calibrated by TopSpin 4.0.6 with deuterated solvents. The following abbreviations are used for the multiplicities: s: singlet, d: doublet, dd: doublet of doublet, t: triplet, q: quadruplet, m: multiplet, br: broad signal for proton spectra; Coupling constants (*J*) are reported in Hertz (Hz). Melting points were uncorrected. Infrared spectra were obtained on agilent Cary630. HRMS were recorded on a Bruker miccOTOF-Q111. GC-MS spectra were performed on Agilent 5977B.

Medium-sized screw-cap test tubes (8 mL) were used for all 0.20 mmol scale reactions: Fisher 13 x 100 mm tubes (Cat. No.1495935C)

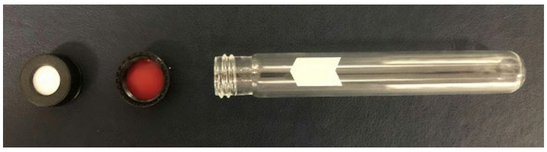

Cap with Septa: Thermo Scientific ASM PHN CAP w/PTFE/SIL (Cat. No.03378316)

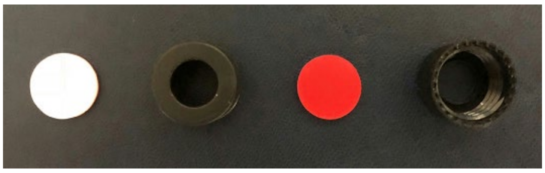

Synthesis of Starting Materials. Substituted alkynes **2a-2j**, **2o**, **2p** were purchased from commercial source and used without further purification. Substituted alkynes

2k-2n were prepared according to the literature[1]. Substituted alkenes **4a-4n, 4q, 4s-4u**, **4z**, **17** were prepared according to the literature [2-8]. Substituted alkenes **4o**, **4p**, **4r** were purchased from commercial source and used without further purification.

Synthesis of Imidazolium Salts (1a-1g and 1i-1l)

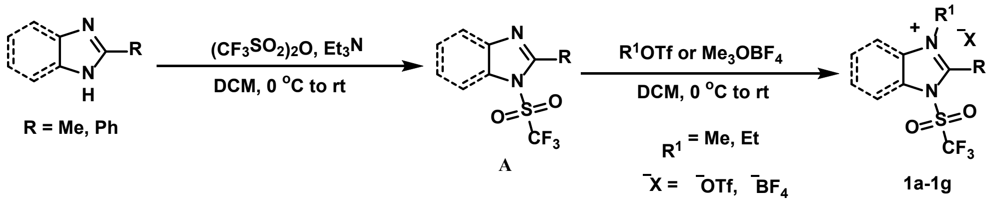

1) To a one-necked 1000 mL flask equipped with a magnetic stirrer, the corresponding imidazole (100 mmol), NEt_3 (150 mmol) and 600 mL DCM were added. The flask was then cooled in a ice bath, and about 130 mmol (36.8 g) $(CF_3SO_2)_2O$ was bubbled into it slowly. Then, The mixture was stirred at room temperature for 2 hours. After the reaction was completed by TLC monitoring, the reaction mixture was evaporated in vacuo. Then, the reaction mixture was quenched with water and extracted with ethyl acetate (300 mL x 3). The combined organic layers were dried over Na_2SO_4, filtered and concentrated. The product was purified by flash column chromatography on silica gel with *n*-pentane/ethyl acetate as eluent to give the corresponding intermediate **A**.

2) Under argon, to a solution of the corresponding intermediate **A** in dried DCM (400 mL) was added dropwise MeOTf (or Me_3OBF_4) (130 mmol) at 0 °C. Then, the mixture was stirred at room temperature for 12 hours (If EtOTf is used, the reaction is refluxed for 24 h), while monitoring by TLC. After that time, the mixture was concentrated under rotary evaporation to give a white solid (or a viscous liquid) crude product, to which Et_2O (300 mL) was added. With vigorous stirring, a solid precipitate was formed. The precipitate was washed with Et_2O (200 mL x 3) and dried in vacuo to yield the title compound (**1a-1g**) as a white solid.

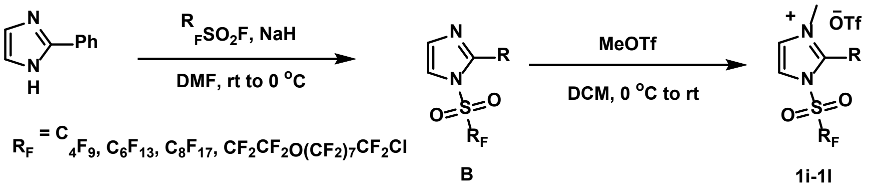

1) To a mixture consisting of 2-phenylimidazole (1.44 g, 10 mmol) and N,N-dimethylformamide (30 mL) was added 60 percent sodium hydride in mineral oil (0.48 g, 12 mmol). After stirring for one hour at room temperature, the corresponding perfluoroalkylsulfonyl fluoride was added dropwise at 0 °C while stirring. After stirring at room temperatures for 3 hours. After the reaction was completed by TLC

monitoring, the reaction mixture was quenched with water and extracted with ethyl acetate (100 mL x 3). The combined organic layers were dried over Na_2SO_4, filtered and concentrated. The product was purified by flash column chromatography on silica gel with *n*-pentane/ethyl acetate as eluent to give the corresponding intermediate **B**.

2） Under argon, to a solution of the corresponding intermediate **B** in dried DCM (60 mL) was added dropwise MeOTf (13 mmol) at 0 ^{o}C. Then, the mixture was stirred at room temperature for 12 hours, while monitoring by TLC. After that time, the mixture was concentrated under rotary evaporation to give a white solid crude product, to which Et_2O (60 mL) was added. With vigorous stirring, a solid precipitate was formed. The precipitate was washed with Et_2O (50 mL x 3) and dried in vacuo to yield the title compound (**1i-1k**) as a white solid.

3-methyl-2-phenyl-1-((trifluoromethyl)sulfonyl)-1H-benzo[d]imidazol-3-ium trifluoromethanesulfonate (1a)

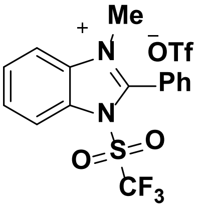

1a

91%; white solid; ^{1}H NMR (400 MHz, CD_3CN) δ 8.21 – 8.18 (m, 1H), 8.13 – 8.09 (m, 1H), 7.99 – 7.93 (m, 2H), 7.90 – 7.74 (m, 5H), 3.90 (s, 3H); ^{13}C NMR (101 MHz, CD_3CN) δ 155.3, 135.1, 133.2, 131.7, 131.4, 131.0, 130.6, 130.3, 122.0 (q, J = 319.0 Hz), 120.8, 119.8 (q, J = 321.0 Hz), 116.2, 116.1, 35.5; ^{19}F NMR (377 MHz, CD_3CN) δ -73.91, -79.32; IR (neat) ν (cm^{-1}): 3069.5, 3045.2, 1608.3, 1569.2, 1448.1, 1263.6, 1218.8, 1131.2, 1028.7, 965.4, 799.5, 762.2, 697.0; HRMS (ESI): calcd for $C_{15}H_{12}F_3N_2O_2S^+$ $[M]^+$ 341.0566; found 341.0571.

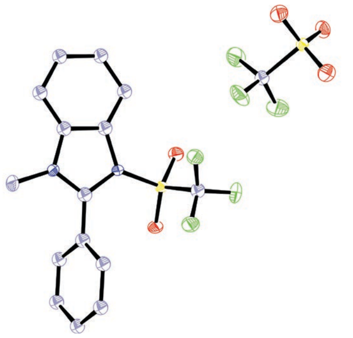

Supplementary Figure 1. X-ray crystallography for **1a** (CCDC number: 1958307)

3-methyl-1-((trifluoromethyl)sulfonyl)-1H-benzo[d]imidazol-3-ium trifluoromethanesulfonate (1b)

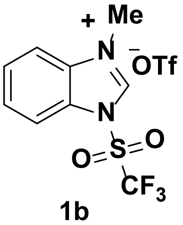

1b

82%; white solid; ^{1}H NMR (400 MHz, CD_3CN) δ 9.90 (s, 1H), 8.10-8.05 (m, 2H), 7.97 – 7.89 (m, 2H), 4.26 (d, J = 0.8 Hz, 3H); ^{13}C

NMR (101 MHz, CD_3CN) δ 146.1, 133.9, 131.8, 130.6, 130.3, 122.2 (q, J = 319.0 Hz), 119.8 (q, J = 321.0 Hz), 116.3, 115.3, 36.4; ^{19}F NMR (377 MHz, $CDCl_3$) δ -68.85 (d, J = 1.3 Hz), -74.08. IR (neat) ν (cm^{-1}): 3076.9, 3011.7, 2987.5, 2950.2, 1619.5, 1578.5, 1449.9, 1285.9, 1218.8, 1129.4, 1025.0, 971.0, 771.6, 751.1; HRMS (ESI): calcd for $C_9H_8F_3N_2O_2S^+$ $[M]^+$ 265.0253; found 265.0261.

2,3-dimethyl-1-((trifluoromethyl)sulfonyl)-1H-benzo[d]imidazol-3-ium trifluoromethanesulfonate (1c)

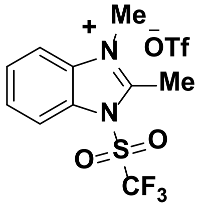

1c

89%; white solid; ^{1}H NMR (400 MHz, CD_3CN) δ 8.11 – 8.08 (m, 1H), 8.01 – 7.99 (m, 1H), 7.87 – 7.82 (m, 2H), 4.11 (s, 3H), 3.12 (s, 3H); ^{13}C NMR (101 MHz, CD_3CN) δ 157.6, 132.4, 130.4, 130.1, 129.5, 121.9 (q, J = 319.0 Hz), 120.0 (q, J = 321.0 Hz),115.3, 115.0, 34.2, 13.9; ^{19}F NMR (377 MHz, CD_3CN) δ -69.29, -74.04. IR (neat) ν (cm^{-1}): 3136.6, 3093.7, 1627.0, 1448.1, 1399.6, 1217.0, 1127.5, 1030.6, 971.0, 896.4, 760.4, 687.7; HRMS (ESI): calcd for $C_{10}H_{10}F_3N_2O_2S^+$ $[M]^+$ 279.0410; found 279.0417.

3-methyl-2-phenyl-1-((trifluoromethyl)sulfonyl)-1H-benzo[d]imidazol-3-ium tetrafluoroborate (1d)

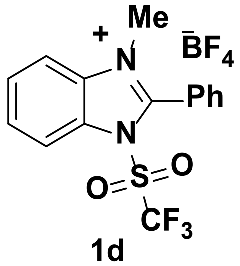

1d

76%; white solid; ^{1}H NMR (400 MHz, CD_3CN) δ 8.21 – 8.19 (m, 1H), 8.12 – 8.10 (m, 1H), 7.99 – 7.94 (m, 2H), 7.90 – 7.86 (m, 1H), 7.82 – 7.74 (m, 4H), 3.89 (s, 3H); ^{13}C NMR (101 MHz, CD_3CN) δ 155.3, 135.1, 133.2, 131.8, 131.4, 131.0, 130.7, 130.3, 121.4, 119.4 (q, J = 321.0 Hz), 116.3, 116.1, 35.5; ^{19}F NMR (377 MHz, CD_3CN) δ -73.87, -152.03; IR (neat) ν (cm^{-1}): 3069.5, 3045.2, 1606.5, 1561.8, 1444.3, 1384.7, 1231.9, 1110.7, 1030.6, 967.2, 836.8, 799.5, 698.9; HRMS (ESI): calcd for $C_{15}H_{12}F_3N_2O_2S^+$ $[M]^+$ 341.0566; found 341.0563.

3-methyl-2-phenyl-1-((trifluoromethyl)sulfonyl)-1H-imidazol-3-ium trifluoromethanesulfonate (1e)

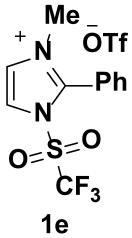

1e

92%; white solid; ^{1}H NMR (400 MHz, CD_3CN) δ 8.10 (s, 1H), 7.91 – 7.90 (m, 1H), 7.83 – 7.79 (m, 1H), 7.75 – 7.67 (m, 4H), 3.70 (s, 3H); ^{13}C NMR (101 MHz, $CDCl_3$) δ 150.7, 134.8, 131.9, 130.2, 127.2, 123.7, 122.1 (q, J =

319.0 Hz), 120.0, 119.5 (q, J = 321.0 Hz), 38.3; ^{19}F NMR (376 MHz, CD_3CN) δ -73.72, -79.23; IR (neat) ν (cm^{-1}): 3160.8, 3125.4, 1610.2, 1487.2, 1451.8, 1263.6, 1220.7, 1120.1, 1151.7, 1075.3, 1030.6, 937.4, 769.7, 685.8; HRMS (ESI): calcd for $C_{11}H_{10}F_3N_2O_2S^+$ $[M]^+$ 291.0410; found 291.0407.

3-ethyl-2-phenyl-1-((trifluoromethyl)sulfonyl)-1H-imidazol-3-ium trifluoromethanesulfonate (1f)

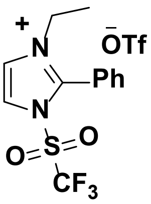

1f

46%; white solid; ^{1}H NMR (400 MHz, CD_3CN) δ 8.09 (d, J = 2.5 Hz, 1H), 7.93 – 7.91 (m, 1H), 7.85 – 7.80 (m, 1H), 7.72 – 7.67 (m, 4H), 4.00 (q, J = 7.3 Hz, 2H), 1.37 (t, J = 7.3 Hz, 3H); ^{13}C NMR (101 MHz, CD_3CN) δ 150.2, 134.8, 131.7, 130.2, 125.2, 124.3, 122.1 (q, J = 319.0 Hz), 120.0, 119.4 (q, J = 321.0 Hz), 47.3, 14.6; ^{19}F NMR (377 MHz, CD_3CN) δ -73.67, -79.33; IR (neat) ν (cm^{-1}): 3103.0, 3129.1, 1591.6, 1442.6, 1217.0, 1153.6, 1120.1, 1088.4, 1028.7, 989.6, 769.7, 721.2; HRMS (ESI): calcd for $C_{12}H_{12}F_3N_2O_2S^+$ $[M]^+$ 305.0572; found 305.0571.

2,3,5-trimethyl-1-((trifluoromethyl)sulfonyl)-1H-imidazol-3-ium trifluoromethanesulfonate (1g)

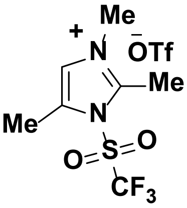

1g

43%; white solid; ^{1}H NMR (400 MHz, CD_3CN) δ 7.65 (s, 1H), 3.73 (s, 3H), 2.85 (s, 3H), 2.33 (s, 3H); ^{13}C NMR (101 MHz, CD_3CN) δ 151.9, 135.1, 122.0 (q, J = 319.0 Hz), 119.6 (q, J = 321.0 Hz), 118.6, 34.5, 13.1, 9.7; ^{19}F NMR (377 MHz, CD_3CN) δ -74.49, -79.38. IR (neat) ν (cm^{-1}): 3134.7, 1647.5, 1552.4, 1440.6, 1394.0, 1220.7, 1153.6, 1121.9, 1028.7, 792.1, 721.2; HRMS (ESI): calcd for $C_7H_{10}F_3N_2O_2S^+$ $[M]^+$ 243.0410; found 243.0408.

3-methyl-1-((perfluorobutyl)sulfonyl)-2-phenyl-1H-imidazol-3-ium trifluoromethanesulfonate (1i)

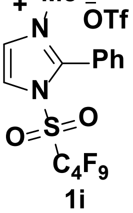

1i

82%; white solid; ^{1}H NMR (400 MHz, CD_3CN) δ 8.11 (d, J = 2.5 Hz, 1H), 7.91 (d, J = 2.6 Hz, 1H), 7.83 – 7.79 (m, 1H), 7.74 – 7.67 (m, 4H), 3.70 (s, 3H); ^{13}C NMR (101 MHz, CD_3CN) δ 150.9, 134.4, 132.0, 130.2, 127.1, 124.0, 122.1 (q, J = 319.0 Hz), 119.9, 38.4; ^{19}F NMR (377 MHz, CD_3CN)

δ -79.33 (s), -81.32 – -81.38 (m), -106.78 (t, *J* = 13.6 Hz), -120.64 – -120.74 (m), -126.14 – -126.25 (m); IR (neat) ν (cm^{-1}): 3123.5, 1608.3, 1449.9, 1351.2, 1261.7, 1200.2, 1142.4, 1026.9, 1006.4, 877.8, 766.0; HRMS (ESI): calcd for $C_{14}H_{10}F_9N_2O_2S^+$ $[M]^+$ 441.0314; found 441.0310.

3-methyl-1-((perfluorohexyl)sulfonyl)-2-phenyl-1H-imidazol-3-ium trifluoromethanesulfonate (1j)

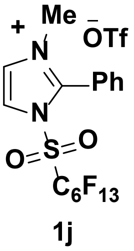

1j

71%; white solid; ^{1}H NMR (400 MHz, CD_3CN) δ 8.10 (d, *J* = 2.2 Hz, 1H), 7.87 (d, *J* = 2.5 Hz, 1H), 7.84 – 7.80 (m, 1H), 7.71 – 7.67 (m, 4H), 3.69 (s, 3H); ^{13}C NMR (101 MHz, CD_3CN) δ 150.9, 134.9, 131.9, 130.2, 127.0, 124.0, 122.1 (d, *J* = 319.0 Hz), 119.9, 38.4; ^{19}F NMR (377 MHz, CD_3CN) δ -79.36 (s), -81.47 – -81.52 (m), -106.46 (t, *J* = 14.2 Hz), -119.59 – -119.64 (m), -121.85 (s), -123.01 (s), -126.50 – -126.59 (m). IR (neat) ν (cm^{-1}): 3140.3, 1608.3, 1451.8, 1209.5, 1146.2, 1079.1, 1030.6, 887.1, 769.7, 698.9; HRMS (ESI): calcd for $C_{16}H_{10}F_{13}N_2O_2S^+$ $[M]^+$ 541.0255; found 541.0256.

3-methyl-1-((perfluorooctyl)sulfonyl)-2-phenyl-1H-imidazol-3-ium trifluoromethanesulfonate (1k)

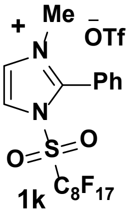

1k

67%; white solid; ^{1}H NMR (400 MHz, CD_3CN) δ 8.10 (d, *J* = 2.4 Hz, 1H), 7.87 (d, *J* = 2.5 Hz, 1H), 7.84 – 7.80 (m, 1H), 7.72 – 7.67 (m, 4H), 3.69 (s, 3H). ^{13}C NMR (101 MHz, CD_3CN) δ 150.9, 134.9, 131.9, 130.2, 127.0, 124.0, 119.8, 38.4. The CF_3- carbon of the $CF_3SO_3^-$ was not observed. ^{19}F NMR (377 MHz, CD_3CN) δ -79.35 (s), -81.54 (tt, *J* = 10.1, 2.2 Hz), -106.44 (t, *J* = 14.1 Hz), -119.48 – -119.58 (m), -121.62 (s), -121.98 (s), -122.24 (s), -123.12 (s), -126.54 – -126.64 (m). IR (neat) ν (cm^{-1}): 3132.8, 1608.3, 1449.9, 1401.5, 1369.8, 1205.8, 1148.0, 1079.1, 1030.6, 937.4, 769.7, 698.9; HRMS (ESI): calcd for $C_{18}H_{10}F_{17}N_2O_2S^+$ $[M]^+$ 641.0192; found 641.0187.

1-((2-((8-chloro-1,1,2,2,3,3,4,4,5,5,6,6,7,7,8,8-hexadecafluorooctyl)oxy)-1,1,2,2-tetrafluoroethyl)sulfonyl)-3-methyl-2-phenyl-1H-imidazol-3-ium trifluoromethanesulfonate (1l)

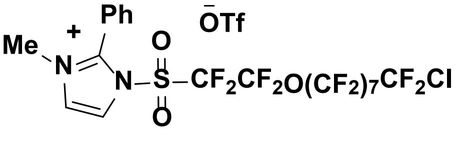

1l

61%; white solid; ^{1}H NMR (400 MHz, CD_3CN) δ 8.08 (d, *J* = 2.4 Hz, 1H), 7.87 (d, *J* = 2.5 Hz,

1H), 7.82 – 7.78 (m, 1H), 7.71 – 7.66 (m, 4H), 3.69 (s, 3H). ^{13}C NMR (126 MHz, CD_3CN) δ 150.8, 134.8, 131.9, 130.1, 127.0, 123.9, 119.8, 38.4. The CF_3- carbon of the $CF_3SO_3^-$ was not observed. ^{19}F NMR (377 MHz, CD_3CN) δ -69.28 (t, *J* = 13.8 Hz), -79.35 (s), -80.41 – -80.48 (m), -83.06 – -83.18 (m), -110.87 (s), -120.67 (s), -121.61 (s), -122.21 (s), -122.41 (s), -125.66 (s). IR (neat) ν (cm^{-1}): 3099.3, 1604.6, 1449.9, 1203.9, 1142.4, 1026.9, 969.1, 771.6, 684.0; HRMS (ESI): calcd for $C_{20}H_{10}ClF_{20}N_2O_3S^+$ $[M]^+$ 772.9781; found 772.9783.

Cyclic Voltammetry Studies for 1a-1l

Unless otherwise noted, the cyclic voltammetry measurements were conducted on a MPI-A multi-functional electrochemical and chemiluminescent system (Xi'an Remex Analytical Instrument Ltd. Co., China) at room temperature, with a polished Pt plate as the working electrode, platinum thread as the counter electrode and Ag-$AgNO_3$ (0.1 M) in CH_3CN as the reference electrode, tetra-n-butylammonium perchlorate (0.1 M) was used as the supporting electrolyte, using Fc+/Fc as the internal standard, the scan rate was 0.1 V/s.

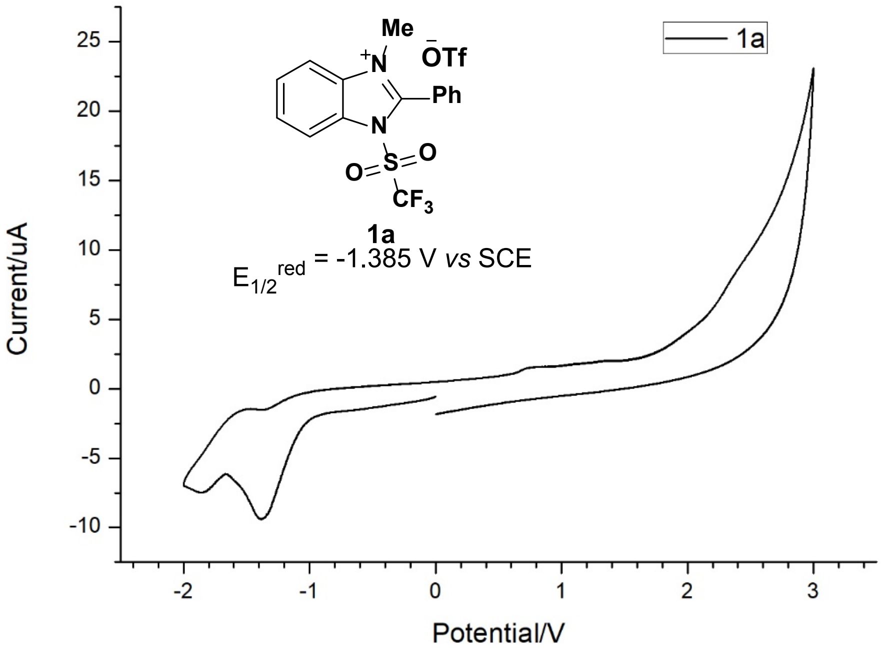

Supplementary Figure 2. Cyclic voltammograms of **1a**

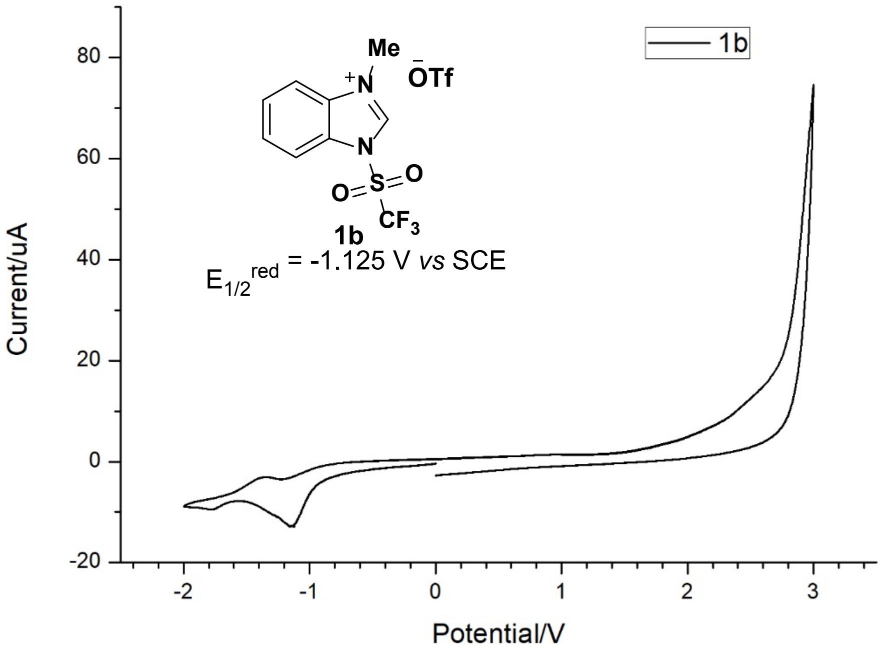

Supplementary Figure 3. Cyclic voltammograms of **1b**

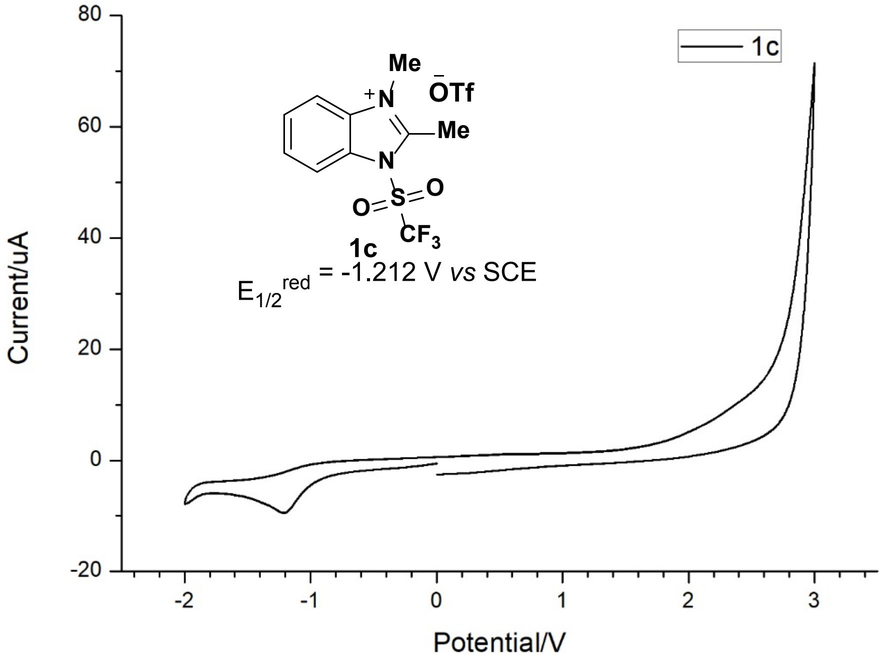

Supplementary Figure 4. Cyclic voltammograms of **1c**

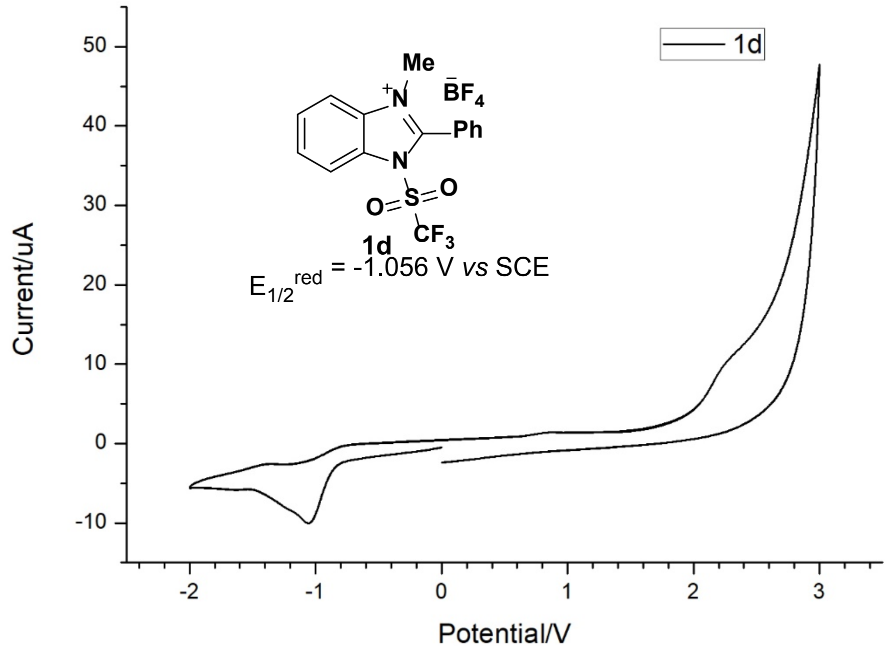

Supplementary Figure 5. Cyclic voltammograms of **1d**

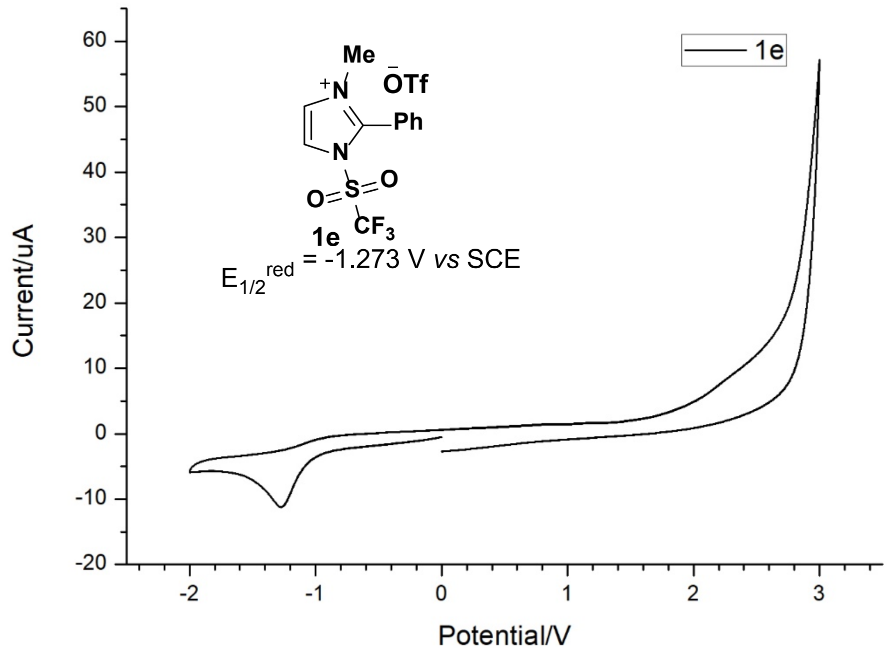

Supplementary Figure 6. Cyclic voltammograms of **1e**

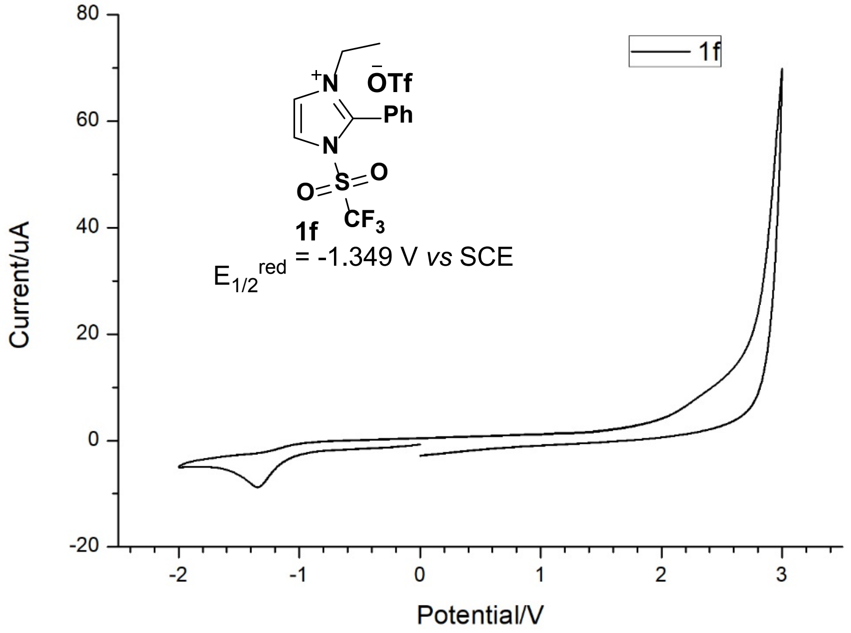

Supplementary Figure 7. Cyclic voltammograms of **1f**

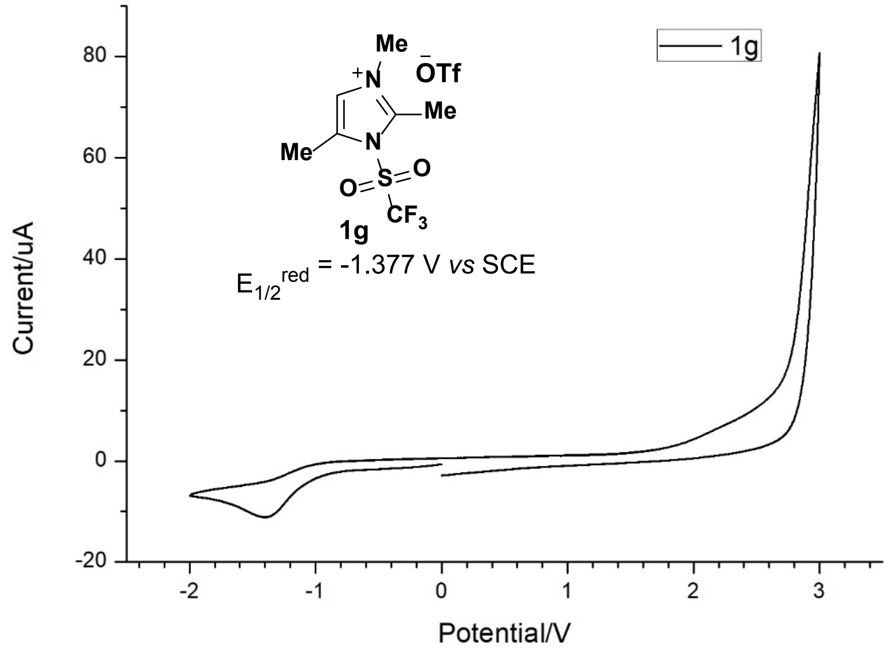

Supplementary Figure 8. Cyclic voltammograms of **1g**

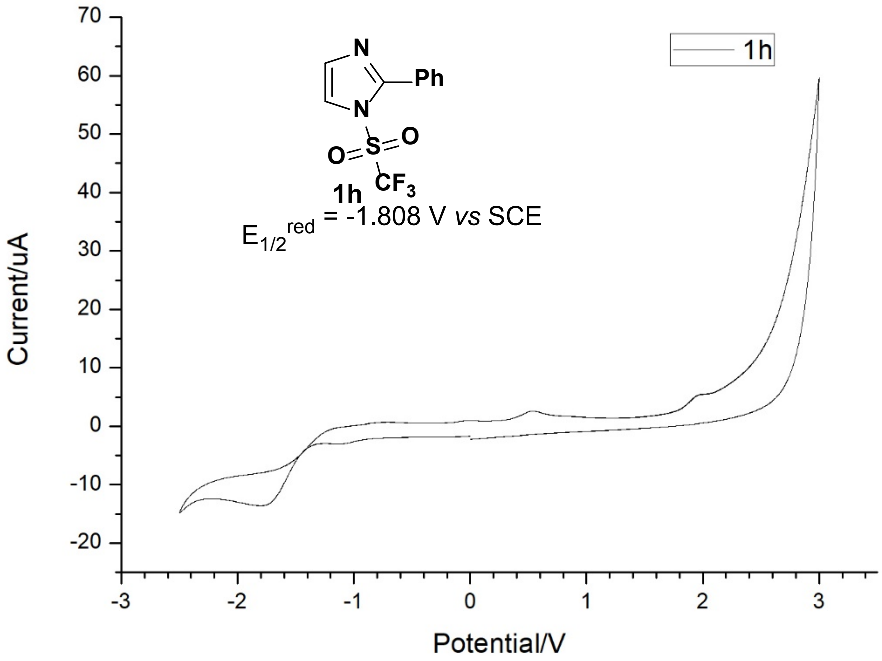

Supplementary Figure 9. Cyclic voltammograms of **1h**

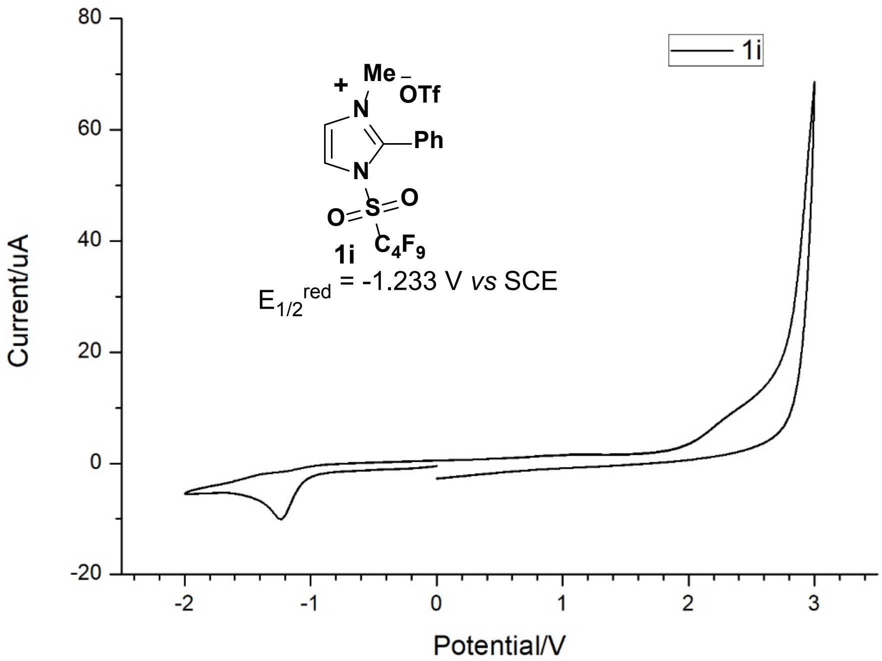

Supplementary Figure 10. Cyclic voltammograms of **1i**

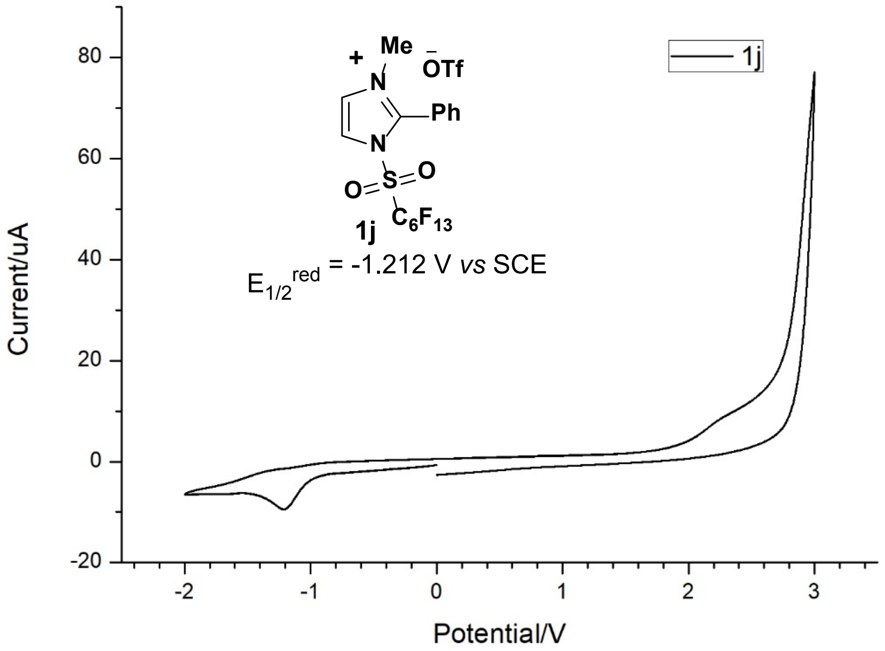

Supplementary Figure 11. Cyclic voltammograms of **1j**

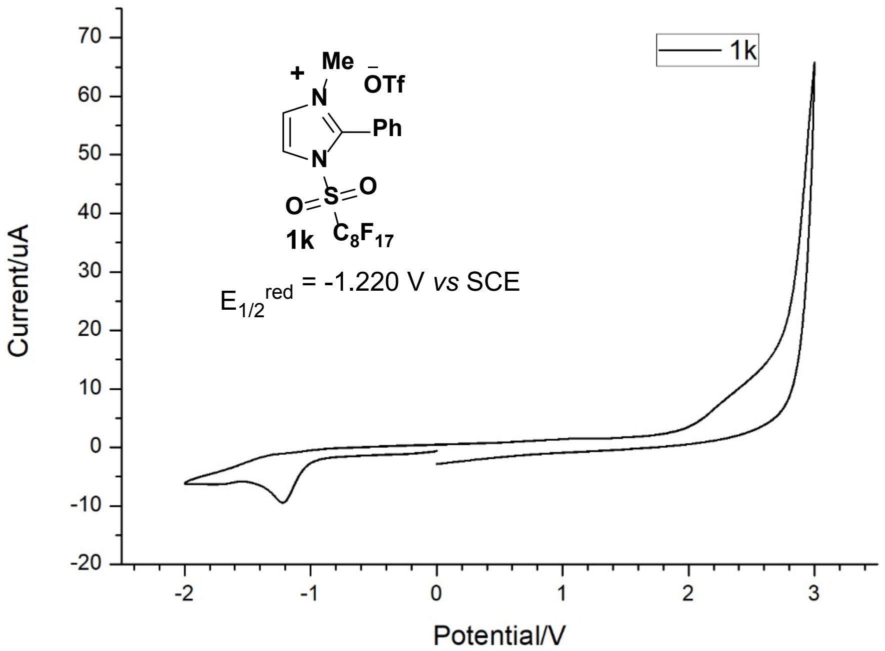

Supplementary Figure 12. Cyclic voltammograms of **1k**

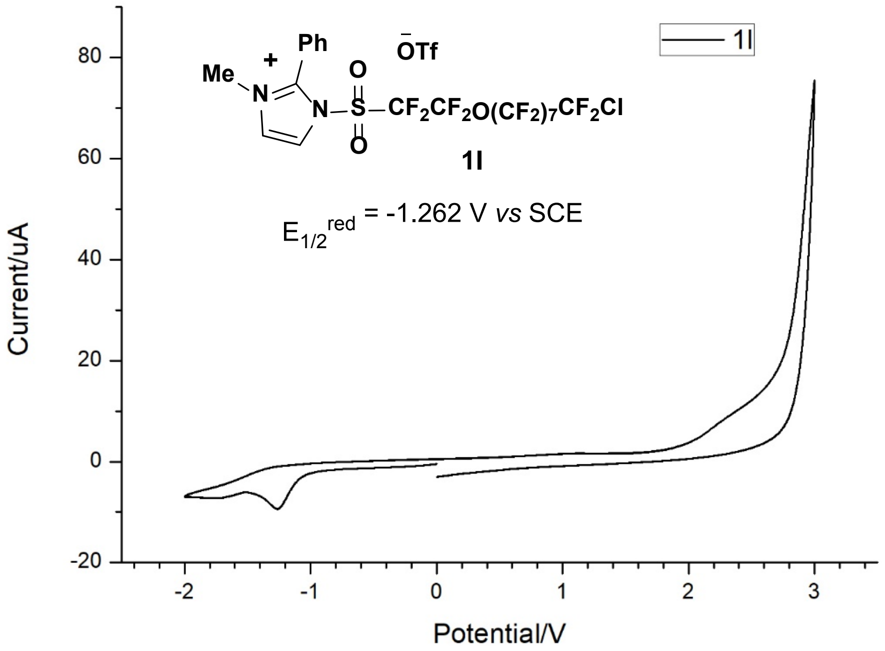

Supplementary Figure 13. Cyclic voltammograms of **1l**

Supplementary Table 1. Optimization of Solvent [a]

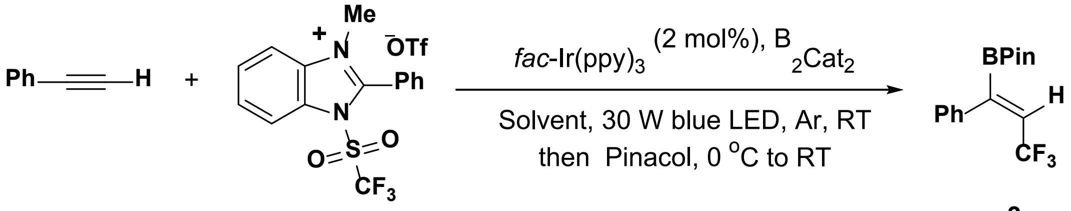

Entry	Solvent	Yield of **3a**[b] / %	Z:E of **3a**[c]
1	CH_3CN	33	>20:1
2	CH_3COCH_3	34	>20:1
3	THF	nd	>20:1
4	EA	53	>20:1

5	CH_3CN:EA = 1:3	71	>20:1
6	CH_3CN: CH_3COCH_3 = 3:1	61	>20:1
7	CH_3COCH_3:EA = 1:3	64	>20:1
8	CH_3CN:EA = 3:1	62	>20:1
9	CH_3CN:EA = 1:5	72	>20:1
10	CH_3CN:EA = 1:9	72	>20:1

[a] All reactions were carried out with **2a** (20.4 mg, 0.20 mmol), **1a** (0.40 mmol, 2.0 equiv), *fac*-Ir(ppy)$_3$ (2 mol%) and B_2Cat_2 (0.4 mmol, 2.0 equiv) in solvent at rt under Ar and 30 W blue LEDs; [b] Yields determined by ^{19}F NMR spectroscopy using Trifluoromethoxybenzene as an internal standard; [c] Determinded by ^{19}F NMR.

Supplementary Table 2. Optimization of Material ratio [a]

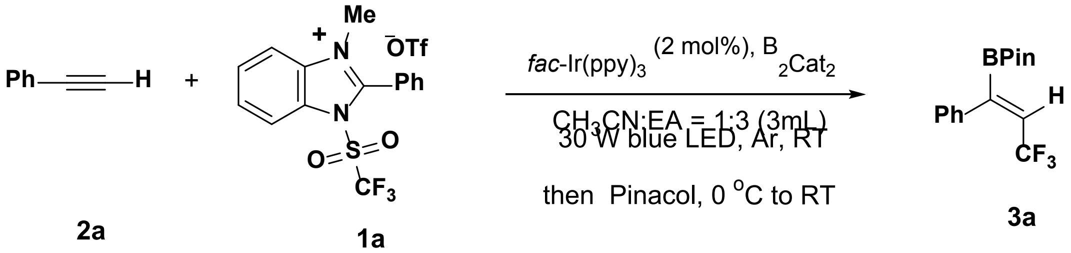

2a 1a 3a

Entry	Mole ratio of **2a**/**1a**/B_2Cat_2	Yield of **3a**[b] / %	Z:E of **3a**[c]
1	1:2:2	71	>20:1
2	1:2:2.5	75	>20:1
3	1:2:3	76	>20:1
4	1:2.5:2.5	82	>20:1
5	1:3:3	81	>20:1
6[d]	1:2.5:2.5	0	-

7[e]	1:2.5:2.5	0	-

[a] All reactions were carried out with **2a** (20.4 mg, 0.20 mmol), **1a**, *fac*-Ir(ppy)$_3$ (2 mol%) and B_2Cat_2 in CH_3CN:EA (1:3) at rt under Ar and 30 W blue LEDs; [b] Yields determined by ^{19}F NMR spectroscopy using Trifluoromethoxybenzene as an internal standard; [c] Determinded by ^{19}F NMR; [d] no *fac*-Ir(ppy)$_3$; [e] no light.

Supplementary Table 3. Optimization of Solvent [a]

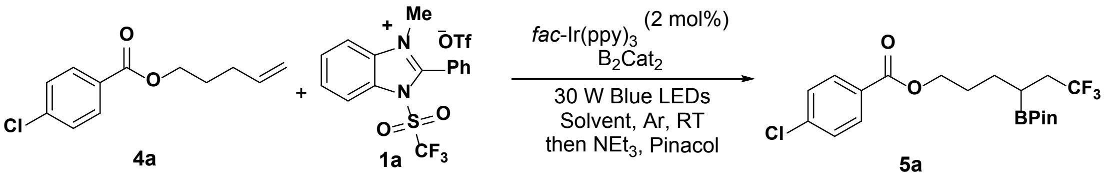

Entry	Solvent	Yield of **5a**[b] / %
1	CH_3CN	26
2	DMF	nd
3	DMAc	nd
4	CH_3COCH_3	39
5	DCM	nd
6	1,4-Dioxane	nd
7	CH_3OH	nd
8	THF	36
9	EtOAc	24
10	EtOAc:CH_3CN = 3:1	31
11	THF:CH_3CN = 3:1	44
12	CH_3COCH_3:CH_3CN = 3:1	37

13	Chlorobenzene: CH_3CN = 3:1	9
14	$PhCF_3$:CH_3CN = 3:1	4
15	$PhCH_3$:CH_3CN = 3:1	7
16	DCM:CH_3CN = 3:1	28
17	DCE:CH_3CN = 3:1	29
18	1,4-Dioxane:CH_3CN = 3:1	42
19	CH_3COCH_3:THF = 3:1	40
20	CH_3COCH_3:THF = 1:1	43
21	CH_3COCH_3:THF = 1:3	42
22	THF :CH_3COCH_3: CH_3CN = 3:1:1	45
23	THF :CH_3COCH_3: CH_3CN = 5:1:1	41
24	THF :CH_3COCH_3: CH_3CN = 8:1:1	42

[a] All reactions were carried out with **4a** (44.9 mg, 0.20 mmol), **1a** (0.40 mmol, 2 equiv), *fac*-Ir(ppy)$_3$ (2 mol%), and B_2Cat_2 (0.4 mmol, 2.0 equiv) in solvent (2.0 mL) at rt under Ar and 30 W blue LEDs; [b] Yields determined by ^{19}F NMR spectroscopy using trifluoromethoxybenzene as an internal standard.

Supplementary Table 4. Optimization of Photocatalysts [a]

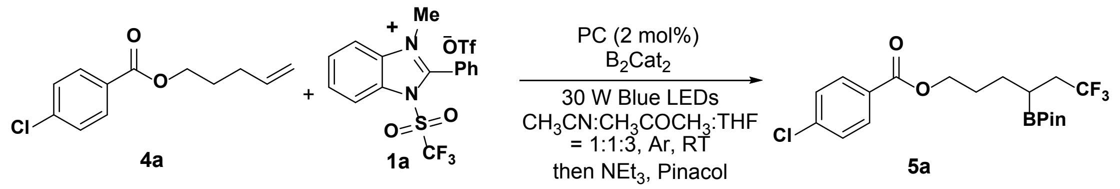

Entry	PC	Yield of **5a**[b] / %
1	Ir(ppy)$_3$	45

2	$Ir(mppy)_3$	44
3	Ir[(dF(p-*t*-Bu)ppy)]$_3$	43
4	$Ir[(dFppy)]_3$	43
5	Ir[(p-F(t-Bu)ppy)]$_3$	27
6	$Ir[(ppy)_2dtbbpy]PF_6$	26
7	$Ir[(dF(CF_3)ppy)_2bpy]PF_6$	21
8	$Ir[(dF(CF_3)ppy)_2dtbbpy]PF_6$	22
9	$Ru(phen)_3(PF_6)_2$	21
10	$Ru(bpy)_3(PF_6)_2$	11
11	4CzIPN	41

[a] All reactions were carried out with **4a** (44.9 mg, 0.20 mmol), **1a** (0.40 mmol, 2 equiv), PC (2 mol%), and B_2Cat_2 (0.4 mmol, 2.0 equiv) in CH_3CN:CH_3COCH_3:THF (1:1:3) 2.0 mL at rt under Ar and 30 W blue LEDs; [b] Yields determined by ^{19}F NMR spectroscopy using trifluoromethoxybenzene as an internal standard.

Supplementary Table 5. Optimization of Light Sources [a]

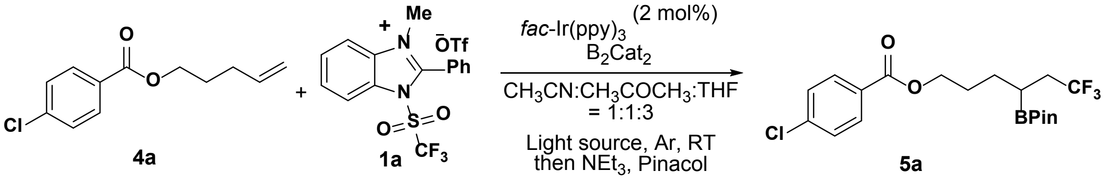

Entry	Light sources	Yield of **5a**[b] / %
1	10 W blue LEDs	46
2	30 W blue LEDs	45
3	60 W blue LEDs	46

4	26 W CFL	27
5	10 W White LEDs	44

[a] All reactions were carried out with **4a** (44.9 mg, 0.20 mmol), **1a** (0.40 mmol, 2 equiv), *fac*-Ir(ppy)$_3$ (2 mol%), and B_2Cat_2 (0.4 mmol, 2.0 equiv) in $CH_3CN:CH_3COCH_3$:THF (1:1:3) 2.0 mL at rt under Ar [b] Yields determined by ^{19}F NMR spectroscopy using trifluoromethoxybenzene as an internal standard.

Supplementary Table 6. Optimization of B Sources [a]

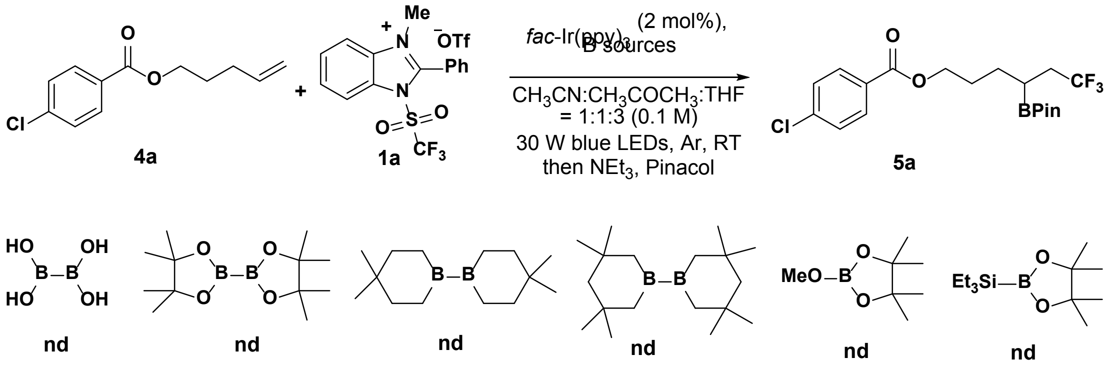

[a] Yield determined by ^{19}F NMR.

Supplementary Table 7. Optimization of Trifluoromethylating Reagent [a]

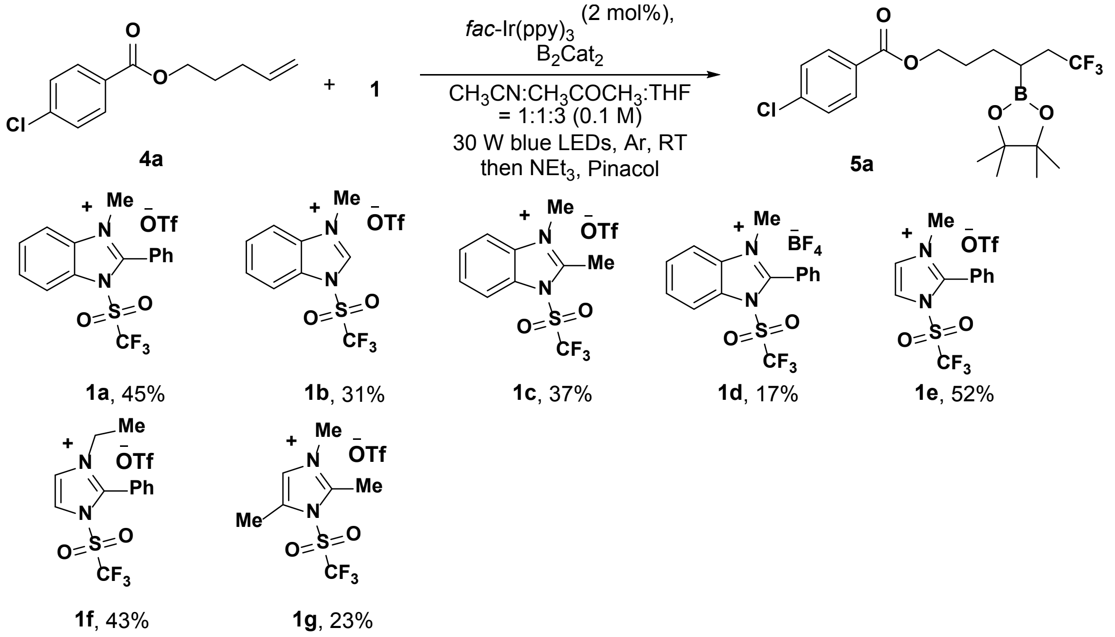

[a] Yield determined by ^{19}F NMR.

Supplementary Table 8. Optimization of Additives [a]

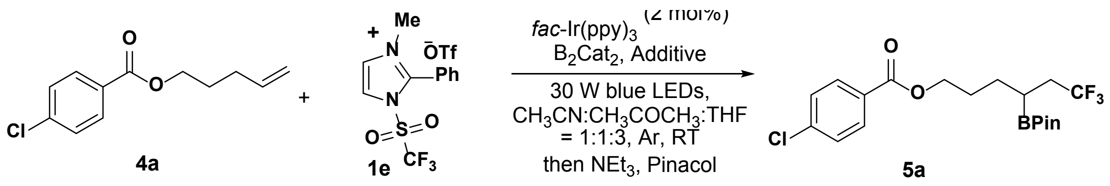

Entry	Additives (1.5 equiv)	Yield of **5a**[b] / %
1	CF_3SO_3H	20
2	CF_3CO_2H	48
3	$BF_3.Et_2O$	46
4	HOAc	47
5	HCOOH	45
6	Benzoic acid	48
7	BCl_3	27
8	BBr_3	nd
9	BEt_3	56
10	$B(OEt)_3$	42
11	bpy	39
12	dtbbpy	38
13	4,4'-diOMe-bpy	36
14	4,4'-diMe-bpy	36
15	6,6'-diMe-bpy	36
16	4,4',5,5'-tetrahydro-2,2'-bioxazole	30
17	DABCO	31

18	DBU	40
19	TBD	42
20	Pyridine	23
21	Isonicotinonitrile	27
22	4-(trifluoromethyl)pyridine	33
23	4-(tert-butyl)pyridine	20
24	N,N-dimethylpyridin-4-amine	13

[a] All reactions were carried out with **4a** (44.9 mg, 0.20 mmol), **1e** (0.40 mmol, 2 equiv), *fac*-Ir(ppy)$_3$ (2 mol%), B_2Cat_2 (0.4 mmol, 2.0 equiv) and Additive (0.3 mmol, 1.5 equiv) in CH_3CN:CH_3COCH_3:THF (1:1:3) 2.0 mL at rt under Ar and 30W blue LEDs. [b] Yields determined by ^{19}F NMR spectroscopy using trifluoromethoxybenzene as an internal standard.

Supplementary Table 9. Optimization of Material ratio [a]

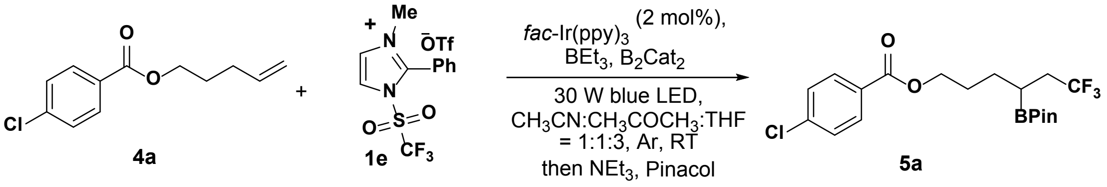

Entry	Mole ratio of **4a**/**1e**/B_2Cat_2/BEt_3	Yield of **5a**[b] / %
1	1:2:2:1.5	56
2	1:2:2:3	60
3	1:2:2:4	61
4	1:1.5:2:3	55
5	1:2.5:2:3	66
6	1:3:2:3	64

7	1:2.5:1.5:3	58
8	1:2.5:2.5:3	65
9	1:2.5:3:3	68
10	1:2.5:3.5:3	67

[a] All reactions were carried out with **4a**, **1e**, *fac*-Ir(ppy)$_3$ (2 mol%), B_2Cat_2 and BEt_3 in CH_3CN:CH_3COCH_3:THF (1:1:3) 2.0 mL at rt under Ar and 30W blue LEDs. [b] Yields determined by ^{19}F NMR spectroscopy using trifluoromethoxybenzene as an internal standard.

Supplementary Table 10. Optimization of Concentration [a]

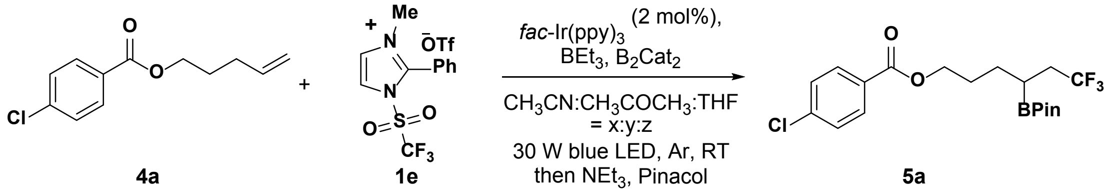

Entry	Solvent (mL)				Yield of **5a**[b] / %
	CH_3CN	CH_3COCH_3	THF	BEt_3 in THF	
1	0.4	0.4	0.6	0.6	68
2	0.2	0.2	0	0.6	73
3	0.1	0.1	0	0.6	81
4	0	0	0	0.6	66
5	0.2	0	0	0.6	59
6	0.4	0	0	0.6	69

[a] All reactions were carried out with **4a** (44.9 mg, 0.20 mmol), **1e** (0.50 mmol, 2.5 equiv), *fac*-Ir(ppy)$_3$ (2 mol%), B_2Cat_2 (0.6 mmol, 3.0 equiv) and BEt_3 (0.6 mmol, 3.0 equiv, 1.0 mmol/L in THF) in CH_3CN:CH_3COCH_3:THF at rt under Ar and 30W blue LEDs. [b] Yields determined by ^{19}F NMR spectroscopy using trifluoromethoxybenzene as an internal standard.

General Procedure

General Procedure A for the synthesis of the product 3

Under argon, to a solution of **1** (0.50 mmol, 2.5 equiv), B_2Cat_2 (0.5 mmol, 2.5 equiv) and *fac*-Ir(ppy)$_3$ (2 mol%) in CH_3CN:EA (1:3) (3 mL) was added corresponding Alkynes **2** (0.2 mmol) at room temperature. After that, the tube was exposed to a 30 W blue LEDs at room temperature about 30 h until the reaction was completed as monitored by TLC or GC-MS analysis. A solution of pinacol (236 mg, 2 mmol) in CH_3CN (1.0 mL) was added dropwise to the mixture at 0 ^oC. After 1 hour, saturated ammonium chloride solution (15 mL) was added and the aqueous layer was extracted with hexane (3x15mL). The combined organic layers were dried over Na_2SO_4, filtered and concentrated. The product was purified by flash column chromatography on silica gel with *n*-pentane/ethyl acetate as eluent to give the corresponding product **3**.

General Procedure B for the synthesis of the product 5

Under argon, to a solution of **1** (0.50 mmol, 2.5 equiv), B_2Cat_2 (0.6 mmol, 3.0 equiv) and *fac*-Ir(ppy)$_3$ (2 mol%) in CH_3CN:CH_3COCH_3 (1:1) (0.2 mL) was added BEt_3 (0.6 mmol, 3.0 equiv, 1 mol/L in THF) and corresponding alkenes **4** (0.2 mmol) at room temperature. After that, the tube was exposed to a 30 W blue LEDs at room temperature about 30 h until the reaction was completed as monitored by TLC or GC-MS analysis. A solution of pinacol (142 mg, 1.2 mmol) in triethylamine (1.1 mL) was added to the mixture. After 1 hour, the reaction mixture was evaporated in vacuo. The product was purified by flash column chromatography on silica gel with *n*-pentane/ethyl acetate as eluent to give the corresponding product **5**.

Procedure for the transformations of Vinylboronates 3a and alkylboronates 5a, 5p.

Procedure for the synthesis of the product 6

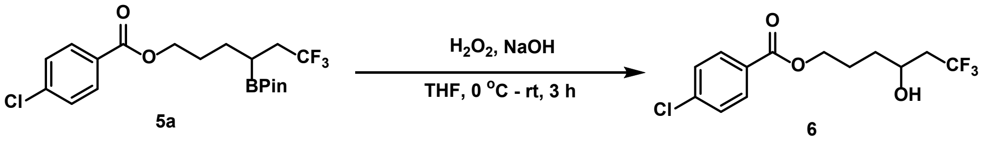

The title compound was prepared according to a literature procedure[9]

To a one-necked 25mL flask equipped with a magnetic stirrer was added **5a** (0.2 mmol, 0.2 equiv) and 2 mL THF. The solution was cooled to 0 °C, aqueous NaOH solution (3 M, 2.0 mL) was added, followed by dropwise addition of aqueous H_2O_2 (30%, 1.0 mL). The reaction mixture was allowed to warm up to room temperature and stirred for 4 h. The mixture was cooled to 0 °C and saturated aqueous $Na_2S_2O_3$ solution (3 mL) was added dropwise. After warming up to room temperature the aqueous layer was extracted with diethyl ether (3 x 15 mL). The combined organic

layers were dried over Na_2SO_4 and concentrated under reduced pressure. Flash column chromatography of the crude material afforded the desired alcohol **6**.

Procedure for the synthesis of the product 7

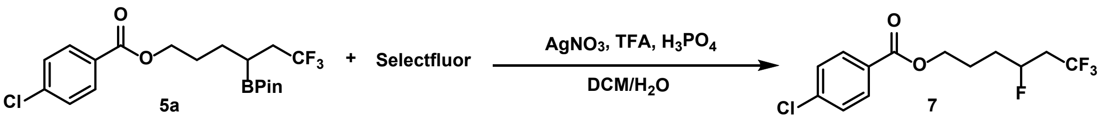

The title compound was prepared according to a literature procedure[10]

Compound **5a** (84.1 mg, 0.4 mmol), $AgNO_3$ (7.0 mg, 0.04 mmol), Selectfluor (425 mg, 1.2 mmol) were placed in a Schlenk tube. The reaction vessel was evacuated and filled with nitrogen for three times. Then dichloromethane (0.6 mL), TFA (0.4 mL), H_3PO_4 (0.1 mL) and water (0.9 mL) were added successively at rt. The reaction mixture was then stirred at 50 ºC for 8 h. The resulting mixture was extracted with CH_2Cl_2. The combined organic phases were dried over anhydrous Na_2SO_4. After the removal of solvent under reduced pressure. The resulting residue was purified by silica gel flash column chromatography to give **7**

Procedure for the synthesis of the product 8

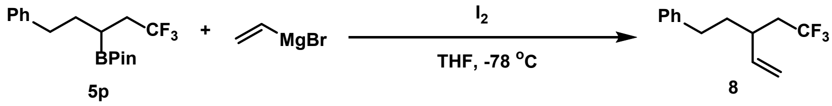

The title compound was prepared according to a literature procedure[10]

Vinylmagnesium bromide solution (0.8 mL, 1.0 M in THF, 0.8 mmol) was added to a solution of compound **5p** (65.6 mg, 0.2 mmol) in THF (3.0 mL) and the reaction mixture was stirred for 30 min at –78 °C. A solution of iodine (203 mg, 0.8 mmol) in MeOH (2.0 mL) was added. The mixture was stirred for another 30 min, and allowed to warm to 0 °C. Upon completion of the reaction, the reaction mixture was quenched with sat. $Na_2S_2O_3$ (aq. 4 mL), and extracted with ethyl acetate. The combined organic layers were dried over $MgSO_4$ and concentrated in *vacuo*. The resulting residue was purified by silica gel flash column chromatography to give **8**.

Procedure for the synthesis of the product 9

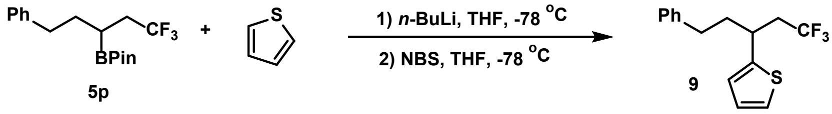

The title compound was prepared according to a literature procedure[11]

Thiophene (21 μL, 0.26mmol) in tetrahydrofuran (1 mL) was cooled to -78 ºC and *n*-butyllithium (187 μL, 0.3 mmol, 1.6 M in hexane) was added dropwise. Then the mixture was allowed to warm up to 0 °C and stirred for 30 minutes. After cooling to -78

°C again, a solutionof **5p** (65.6 mg, 0.2 mmol) in tetrahydrofuran (0.5 mL) was added dropwise. The reaction mixture was allowed to stir at -78 °C for 1 hour. *N*-bromosuccinimide (46.3 mg, 0.26 mmol) in tetrahydrofuran (1 mL) was added dropwise and the mixture was stirred at -78 °C for 1 hour. Saturated aqueous sodium thiosulfate solution (2mL) was added. The reaction mixture was allowed to warm to room temperature and diluted with water and ethyl acetate. The aqueous layer was extracted with ethyl acetate (3x15 mL).The combined organic layers were dried over magnesium sulfate, filtered and concentrated. The product was purified by flash column chromatography on silica gel with *n*-pentane as eluent to give the corresponding product **9.**

Procedure for the synthesis of the product 10

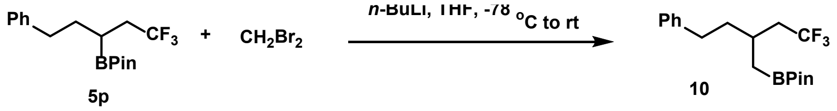

The title compound was prepared according to a literature procedure[11]
5p (65.6 mg, 0.2 mmol) was dissolved in tetrahydrofuran (2 mL), and dibromomethane (35 µL, 0.50 mmol)was added. The mixture was cooled to -78 °C, *n*-butyllithium (0.28 mL,0.44 mmol, 1.6 M in hexane) was added dropwise. The reaction mixture was stirred at -78 °C for 20 min, and then warmed to room temperature for another 2 hours. The mixture was quenched with saturated aqueous ammonium chloride solution, extracted with diethyl ether (3x15mL), dried over magnesium sulfate, filtered and concentrated. The product was purified by flash column chromatography on silica gel with *n*-pentane/diethyl ether (100:1) as eluent to give the corresponding product **10.**

Procedure for the synthesis of the product 11

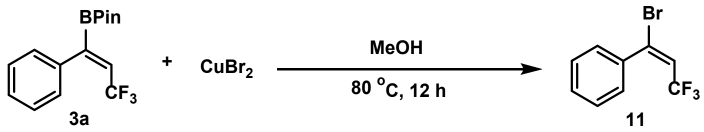

The title compound was prepared according to a literature procedure[12]
To a 10-mL Schlenk flask were added **3a** (0.2 mmol), $CuBr_2$ (0.2 mmol) and MeOH (1 mL). The resultant mixture was stirred for 12h at 80 °C, then cooled to room temperature. H_2O (5 mL) was added into the reaction mixture, and extracted with Et_2O (5 mL x 3). The combined organic layers were washed with brine (10 mL), dried over Na_2SO_4, and filtered. The filtrate was concentrated under reduced pressure. The residue was purified by flash column chromatography on silica gel to give the target product **11**.

Procedure for the synthesis of the product 12, 13, 14, 15, 16

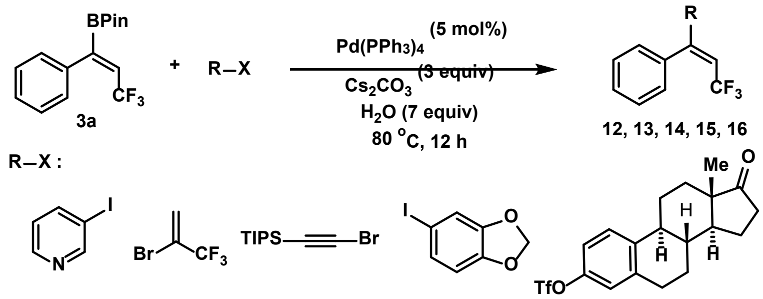

The title compound was prepared according to a literature procedure[13]

A 25 mL of Schlenk tube equipped with a magnetic stir bar was charged with $Pd(PPh_3)_4$ (5 mol%) and Cs_2CO_3 (3.0 equiv). Then toluene (2.0 mL), H_2O (7.0 equiv), **3a** (0.2mmol, 1.0 equiv) and **R-X** (0.4 mmol, 2.0 equiv) were added under Ar, respectively. The Schlenk tube was screw capped and heated to 80 °C (oil bath). After stirring for 24 h, the reaction mixture was cooled to room temperature and diluted with ethyl acetate (2 mL). Then the reaction mixture was diluted with EtOAc and filtered with a pad of cellite. The filtrate was concentrated, and the residue was purified with silica gel chromatography to give products **12**, **13**, **14, 15, 16**.

Mechanistic studies

Control Experiment (a):

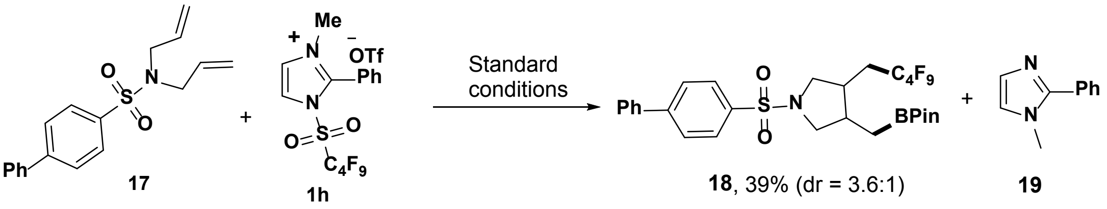

Under argon, to a solution of **1h** (0.50 mmol, 2.5 equiv), B_2Cat_2 (0.6 mmol, 3.0 equiv) and *fac*-Ir(ppy)$_3$ (2 mol%) in CH_3CN:CH_3COCH_3 (1:1) (0.2 mL) was added BEt_3 (0.6 mmol, 3.0 equiv, 1 mol/L in THF) and corresponding alkenes **2** (0.2 mmol) at room temperature. After that, the tube was exposed to a 30 W blue LEDs at room temperature about 30 h until the reaction was completed as monitored by TLC analysis. A solution of pinacol (142 mg, 1.2 mmol) in triethylamine (1.1 mL) was added to the mixture. After 1 hour, the reaction mixture was evaporated in vacuo. The product was purified by flash column chromatography on silica gel with *n*-pentane/ethyl acetate as eluent to give the corresponding product **18** and **19**.

1-([1,1'-biphenyl]-4-ylsulfonyl)-3-(2,2,3,3,4,4,5,5,5-nonafluoropentyl)-4-((4,4,5,5-tetramethyl-1,3,2-dioxaborolan-2-yl)methyl)pyrrolidine (18)

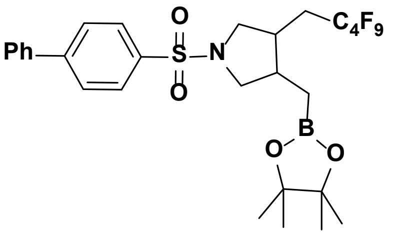

^{1}H NMR (400 MHz, $CDCl_3$, mixture of diastereomers, dr = 3.6:1) δ 7.91 – 7.89 (m, 2H), 7.76 – 7.72 (m, 2H), 7.63 – 7.59 (m, 2H), 7.50 – 7.40 (m, 3H), 3.71 – 3.47 (m, 2H), 3.23 – 2.82 (m, 2H), 2.50 – 2.34 (m, 2H), 2.17 – 1.66 (m, 2H), 1.23 (M, 12H), 0.97 – 0.53 (m, 2H). ^{13}C NMR (126 MHz, $CDCl_3$, asterisk denotes minor diastereomers peaks) δ 145.7, 145.6*, 139.3, 139.2*, 135.4, 135.2*, 129.0*, 129.0, 128.5*, 128.4, 128.0*, 127.8, 127.7, 127.7* (s), 127.3, 83.6, 83.6*, 53.8, 53.7*, 53.3* (d, *J* = 4.4 Hz), 51.5 (d, *J* = 3.9 Hz), 40.4, 38.8*, 37.5, 34.7*, 32.7* (t, *J* = 21.9 Hz), 28.7 (t, *J* = 21.5 Hz), 25.0*, 24.8, 24.7, 24.7*. ^{19}F NMR (377 MHz, $CDCl_3$, mixture of diastereomers) δ -81.00 – -81.07 (m), -111.71 – -114.90 (m), -124.36 – -124.38 (m), -125.86 – -126.00 (m). ^{11}B NMR (128 MHz, $CDCl_3$) δ 33.95.

methyl-2-phenyl-1H-imidazole (19)

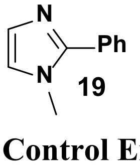

19

^{1}H NMR (400 MHz, $CDCl_3$) δ 7.63 – 7.60 (m, 2H), 7.47 – 7.37 (m, 3H), 7.11 (d, *J* = 1.2 Hz, 1H), 6.96 (d, *J* = 1.2 Hz, 1H), 3.73 (s, 3H). ^{13}C NMR (101 MHz, $CDCl_3$) δ 147.8, 130.6, 128.7, 128.6, 128.5, 128.4, 122.4, 34.5.

Control Experiment (b):

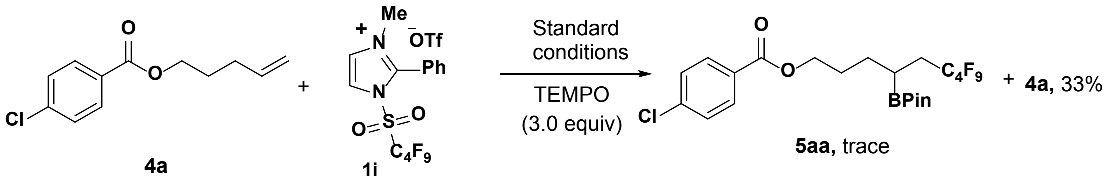

Under argon, to a solution of **1i** (0.50 mmol, 2.5 equiv), B_2Cat_2 (0.6 mmol, 3.0 equiv), TEMPO (3.0 equiv) and *fac*-Ir(ppy)$_3$ (2 mol%) in CH_3CN:CH_3COCH_3 (1:1) (0.2 mL) was added BEt_3 (0.6 mmol, 3.0 equiv, 1 mol/L in THF) and corresponding alkenes **4a** (0.2 mmol) at room temperature. After that, the tube was exposed to a 30 W blue LEDs at room temperature about 30 h. A solution of pinacol (142 mg, 1.2 mmol) in triethylamine (1.1 mL) was added to the mixture. After 1 hour, ^{19}F NMR analysis of this reaction mixture showed that the desired product **5aa** was not formed and substrate **4a** was recovered in 33% yield.

Luminescence Quenching Experiment

The luminescence quenching experiment was taken using a F-7000 FL Spectrophotometer (Hitachi, Japan). The experiments were carried out in 5 x 10^{-4} mol/L of Ir(ppy)$_3$ in CH_3CN:CH_3COCH_3:THF (1:1:6) at 25 ^{o}C. The excitation wavelength was 430 nm and the emission intensity was collected at 516 nm. The concentrations of quencher (**4a**, **1e**, B_2cat_2) in CH_3CN:CH_3COCH_3:THF were 0.01 mmol/mL (**Supplementary Figure 14**). The concentrations of quencher **1e** in CH_3CN:CH_3COCH_3:THF (1:1:6) was 4 mmol/L, 8 mmol/L, 12 mmol/L, 16 mmol/L (**Supplementary Figure 15**).

To determine whether a reductive or oxidative quenching cycle is operative in the reaction, fluorescence quenching studies were conducted. Based on the above data, photoexcited Ir(ppy)$_3$* can be quenched by **1e**, involved a reductive quenching cycle.

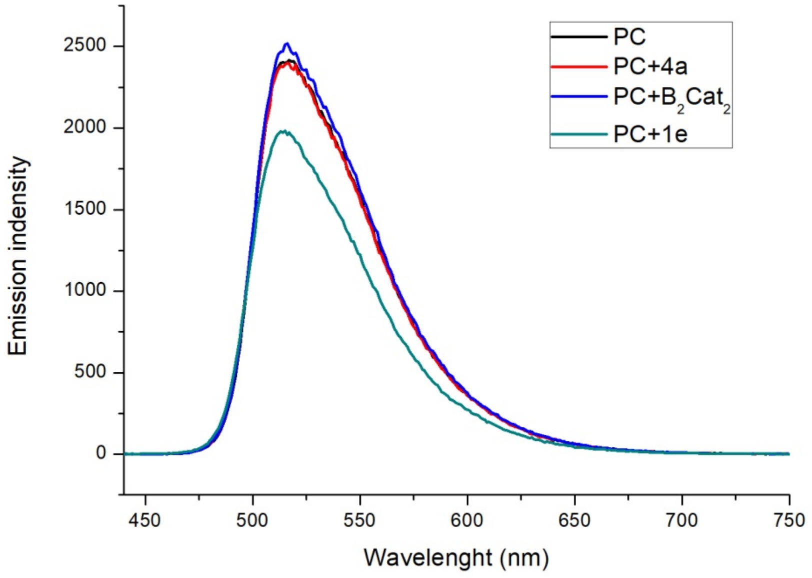

Supplementary Figure 14. The data of fluorescence quenching of Ir(ppy)$_3$ by **4a**, **1e**, B_2cat_2.

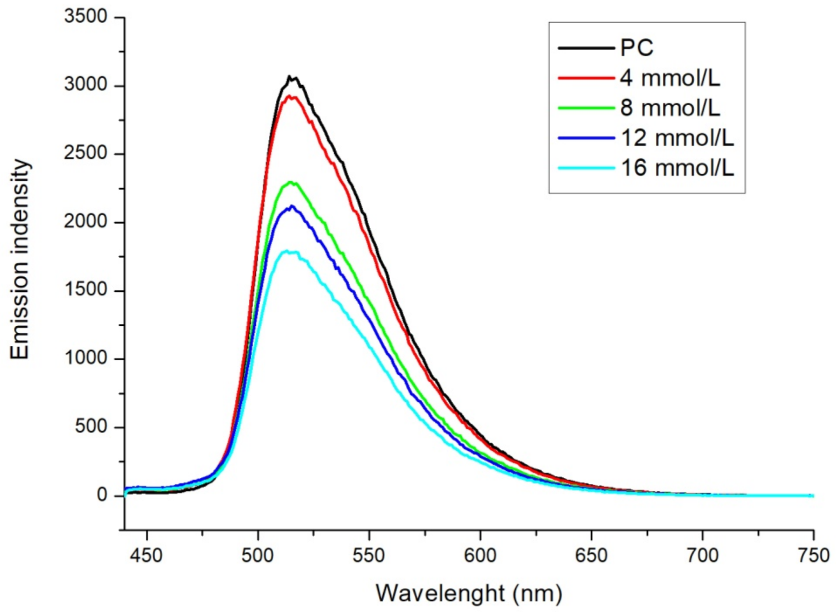

Supplementary Figure 15. The data of fluorescence quenching of Ir(ppy)$_3$ by different concentrations of **1e**.

Computational Studies

DFT studies on the mechanism and selectivities of this reaction have been conducted. The calculations were performed with Gaussian 09.[14] Geometry optimizations of all the ground state, excitation state and transition structures were carried out at the B3LYP-D3 level of theory[15-17] with the 6-31G(d,p) basis set (LANL2DZ for Ir) in ethyl acetate using the CPCM solvation model.[18-20] Vibrational frequencies were computed at the same level to verify that optimized structures are local minimums or transition states and to evaluate zero-point vibrational energies (ZPE) and thermal corrections at 298 K. Solvent effects in ethyl acetate were evaluated at the more accurate B3LYP-D3/6-311+G(2d,p) level (SDD for Ir) with the CPCM model using the above optimized structures.

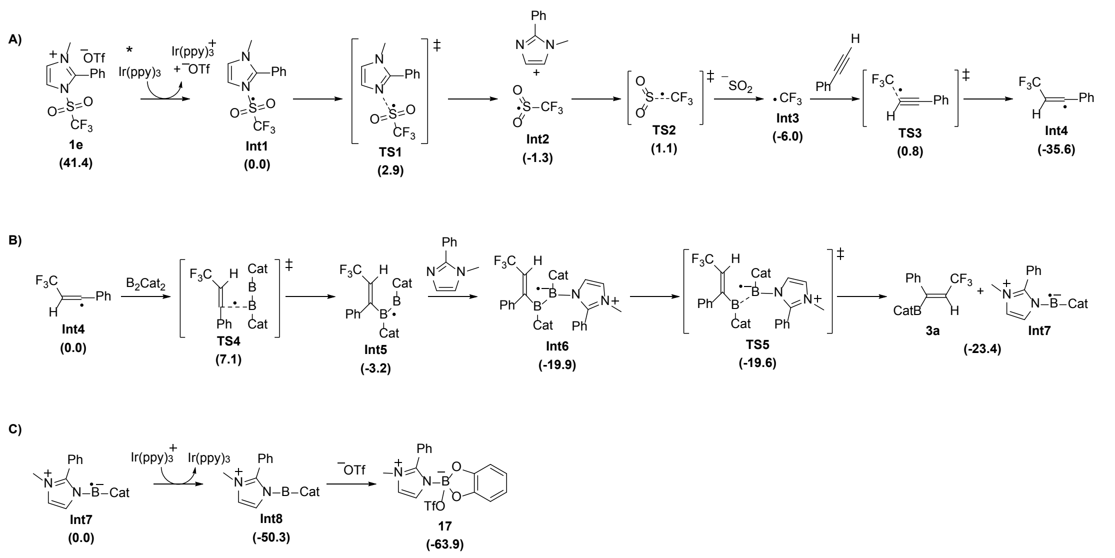

Supplementary Figure 16. DFT calculations on reaction pathways. Numbers associated with each molecule are relative Gibbs free energy (kcal·mol^{-1}) in ethyl acetate. Note that in each part the zero points of relative energies are different. (A) Reductive release of CF_3 radical from **1e** and its addition to aryl alkyne. (B) Addition of alkenyl radical to B_2Cat_2 and imidazole-facilitated B-B bond cleavage. (C) Oxidation of boryl radical and regeneration of photoredox catalyst.

The reaction of phenylacetylene (**2a**) and **1e** is chosen as the model system. The computed reaction pathway is summarized in Supplementary Figure 16. Excited state $Ir(ppy)_3$ reduces the N-trifluoromethylsulfonylimidazolium cation to its radical **Int1**. Subsequent fragmentation of **Int1** generates CF_3 radical, SO_2 and 1-methyl-2-phenyl imidazole (**IMD**). The CF_3 radical regiospecifically attacks to terminal site of alkyne (*vide infra*), affording alkenyl radical **Int4**. Then stereospecific addition of **Int4** to B_2Cat_2 (*vide infra*) leads to **Int5**. The coordination of **IMD** to **Int5** forms a more

stable intermediate **Int6**, in which the B-B bond is pre-activated. Facile cleavage of **Int6** affords product **3a** and **Int7**. The boryl radical **Int7** finally reduces $Ir(ppy)_3^+$ to $Ir(ppy)_3$, finishing the catalytic cycle.

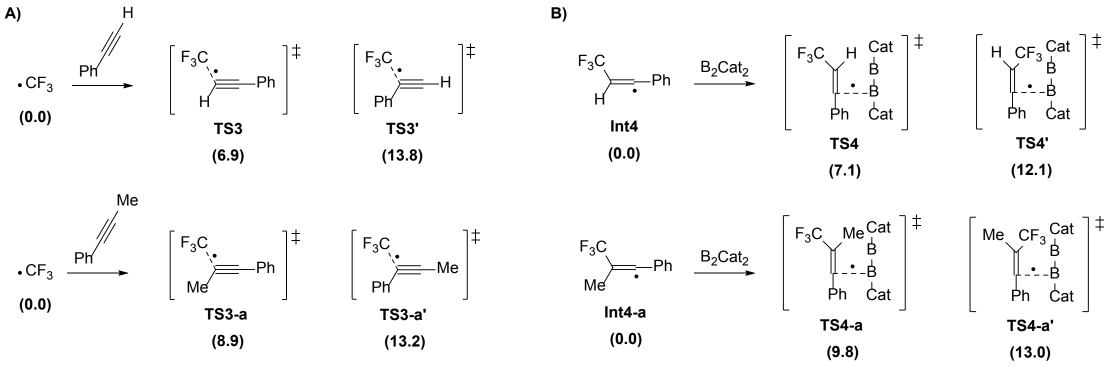

Supplementary Figure 17. DFT calculations on regioselectivity and stereoselectivity. Numbers associated with each molecule are relative Gibbs free energy (kcal·mol^{-1}) in ethyl acetate. Note that in each reaction the zero points of relative energies are different. (A) Regiochemical control on the CF_3 radical addition to phenylacetylene and 1-phenylpropyne. (B) Stereochemical control on additions of **Int4** and **Int4-a** to B_2Cat_2.

The regio- and stereoselectivity have been computationally explained based on the above reaction pathway. The results are summarized in Supplementary Figure 17. The CF_3 radical addition to alkyne controls the regioselectivity. In **TS3**, the radical character is being built at the alpha-carbon of phenyl group. Conjugation effect lowers the energy of **TS3**. However, in **TS3'**, the conjugative stabilization is not present. The alkenyl radical addition to B_2Cat_2 controls the stereoselectivity. As shown in Supplementary Figure 17B, the steric repulsion between trifluoromethyl and B_2Cat_2 caused **TS4'** to be higher by 5 kcal mol^{-1} than **TS4**. Replacing phenylacetylene with 1-phenylpropyne leads to the same regio- and stereoselectivity, although the energy differences of both transition states are slightly diminished.

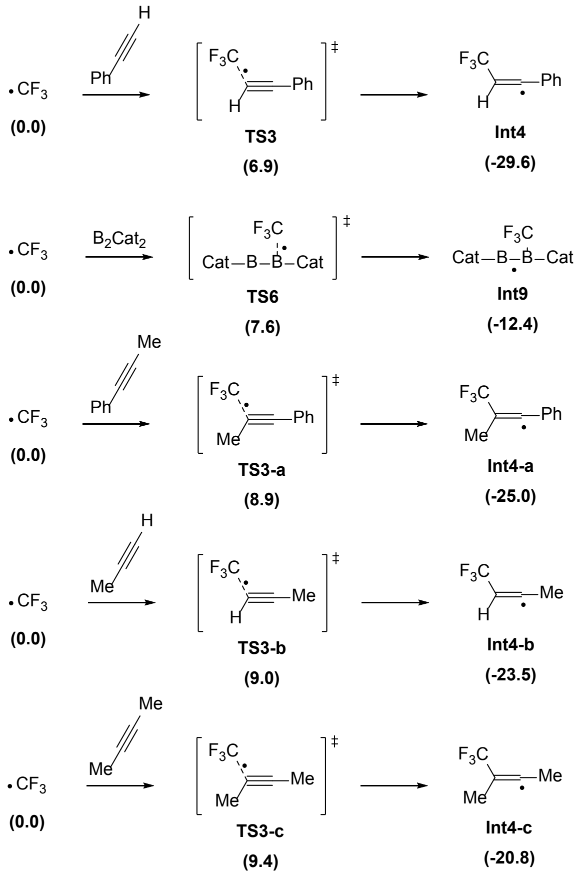

Supplementary Figure 18. DFT calculations on CF_3 radical addition to phenylacetylene, B_2Cat_2, 1-phenylpropyne, propyne and 2-butyne. Numbers associated with each molecule are relative Gibbs free energy (kcal·mol^{-1}) in ethyl acetate. Note that in each reaction the zero points of relative energies are different.

The experimentally observed substrate scope is attributed to the interference of a competing pathway in which CF_3 radical reacts with B_2Cat_2. The addition barriers of CF_3 radical to alkynes and B_2Cat_2 are shown in Supplementary Figure 18. It is found that the addition to phenylacetylene is more favorable than that to B_2Cat_2. However, for 1-phenylpropyne and propyne, the CF_3 radical addition to B_2Cat_2 is a bit lower in energy. This leads to the consumption of generated CF_3 radical and low conversion of alkyne (low yield of the desired product). Increasing the concentration of alkyne is in favor of its competition for CF_3 radical against B_2Cat_2.

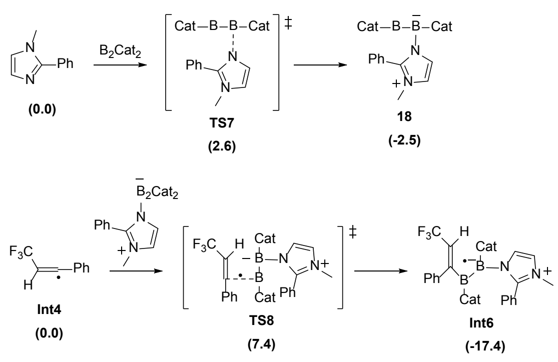

Supplementary Figure 19. DFT calculations on possible radical addition to complex between IMD and B_2Cat_2. Numbers associated with each molecule are relative Gibbs free energy (kcal·mol^{-1}) in ethyl acetate. Note that in each part the zero points of relative energies are different.

As shown in Supplementary Figure 19, the generated **IMD** can react with B_2Cat_2 to form a slightly more stable complex **18**. The addition of alkenyl radical to**18** has a similar barrier compared with its addition to B_2Cat_2. However, since **IMD** is consumed along with product formation, the concentration of **18** is very low compared with stoichiometric or even excess reagent B_2Cat_2. Therefore, alkenyl radical is expected to preferentially react with B_2Cat_2 instead of **18**. Nevertheless, the pathways shown in Supplementary Figure 19 and 16 will gave the same intermediate **Int6** for further transformation.

(*Z*)-4,4,5,5-tetramethyl-2-(3,3,3-trifluoro-1-phenylprop-1-en-1-yl)-1,3,2-dioxaborolane (3a)

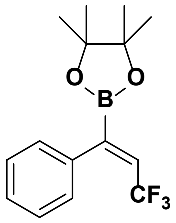

3a

Following the general procedure A, **3a** was synthesized from the corresponding alkyne (20.4 mg) and purified by silica gel chromatography (EA/PE = 1/30); 38.8 mg (65%); Gram-scale reaction: **3a** was synthesized from the corresponding alkyne (10.0 mmol, 1.02 g); 1.25 g (42%). colorless liquid. ^{1}H NMR (400 MHz, $CDCl_3$) δ 7.36 – 7.28 (m, 3H), 7.21 – 7.19 (m, 2H), 6.45 (q, *J* = 8.6 Hz, 1H), 1.28 (s, 12H). ^{13}C NMR (101 MHz, $CDCl_3$) δ 137.0, 129.4 (q, *J* = 33.0 Hz), 127.8, 127.7 (q, *J* = 2.1 Hz), 127.5, 122.3 (q, *J* = 272.9 Hz), 84.7, 24.7. The signal of the α-B-carbon was not observed. ^{19}F NMR (377 MHz, $CDCl_3$) δ -57.45 (d, *J* = 8.5 Hz). ^{11}B NMR (128 MHz, $CDCl_3$) δ 29.95. IR (neat) ν (cm^{-1}): 2981.9, 2931.6, 1634.4, 1496.5, 1382.8, 1340.0, 1265.4, 1118.2, 978.4, 862.9, 779.0, 697.0, 618.7, ; HRMS (ESI): calcd for $C_{15}H_{18}BF_3O_2Na^+$ $[M + Na]^+$ 321.1244; found 321.1247.

(*Z*)-4,4,5,5-tetramethyl-2-(3,3,3-trifluoro-1-(4-methoxyphenyl)prop-1-en-1-yl)-1,3,2-dioxaborolane (3b)

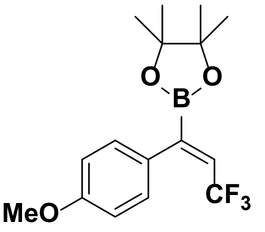

3b

Following the general procedure A, **3b** was synthesized from the corresponding alkyne (26.4 mg) and purified by silica gel chromatography (EA/PE = 1/30); 47.1mg (72%); colorless liquid. ^{1}H NMR (400 MHz, $CDCl_3$) δ 7.20 – 7.16 (m, 2H), 6.89 – 6.85 (m, 2H), 6.39 (q, *J* = 8.8 Hz, 1H), 3.81 (s, 3H), 1.29 (s, 12H). ^{13}C NMR (101 MHz, $CDCl_3$) δ 159.1, 129.4 (q, *J* = 2.0 Hz), 129.2, 128.5 (q, *J* = 32.9 Hz), 122.5 (q, *J* = 273.2 Hz), 113.3, 84.7, 55.1, 24.7. The signal of the α-B-carbon was not observed. ^{19}F NMR (377 MHz, $CDCl_3$) δ -57.33 (d, *J* = 8.8 Hz). ^{11}B NMR (128 MHz, $CDCl_3$) δ 30.29. IR (neat) ν (cm^{-1}): 3203.6, 2980.0, 2845.8, 1684.8, 1600.9, 1513.3, 1459.3, 1371.7, 1336.3, 1235.6, 1174.1, 1105.2, 1028.7, 978.4, 833.1, 603.8; HRMS (ESI): calcd for $C_{16}H_{21}BF_3O_3^+$ $[M + H]^+$ 329.1530; found 329.1527.

(*Z*)-2-(1-(4-bromophenyl)-3,3,3-trifluoroprop-1-en-1-yl)-4,4,5,5-tetramethyl-1,3,2-dioxaborolane (3c)

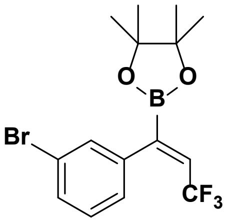

3c

Following the general procedure A, **3c** was synthesized from the corresponding alkyne (36.2 mg) and purified by silica gel chromatography (EA/PE = 1/30); 47.5 mg (63%); colorless liquid. ^{1}H NMR (400 MHz, $CDCl_3$) δ 7.36 – 7.33 (m, 1H), 7.26 (t, J = 1.7 Hz, 1H), 7.13 (t, J = 7.8 Hz, 1H), 7.05 – 7.03 (m, 1H), 6.41 (q, J = 8.5 Hz, 1H), 1.21 (s, 12H). ^{13}C NMR (101 MHz, $CDCl_3$) δ 139.0, 130.6 (q, J = 2.0 Hz), 130.5, 130.5 (q, J = 33.2 Hz), 129.4, 126.40 (q, J = 2.1 Hz), 122.1 (q, J = 273.5 Hz), 121.9, 85.0, 24.7. The signal of the α-B-carbon was not observed. ^{19}F NMR (377 MHz, $CDCl_3$) δ -57.61 (d, J = 8.4 Hz). ^{11}B NMR (128 MHz, $CDCl_3$) δ 29.71. IR (neat) ν (cm^{-1}): 2981.9, 2931.6, 1559.9, 1474.2, 1373.5, 1338.1, 1267.3, 1138.7, 952.3, 862.9, 788.3, 684.0, 629.9; HRMS (EI): calcd for $C_{15}H_{17}BBrF_3O_2$ [M] 375.0493; found 375.0500.

(*Z*)-4-(3,3,3-trifluoro-1-(4,4,5,5-tetramethyl-1,3,2-dioxaborolan-2-yl)prop-1-en-1-yl)benzonitrile (3d)

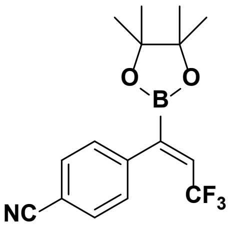

3d

Following the general procedure A, **3d** was synthesized from the corresponding alkyne (25.4 mg) and purified by silica gel chromatography (EA/PE = 1/5); 32.4 mg (50%); colorless liquid. ^{1}H NMR (400 MHz, $CDCl_3$) δ 7.65 – 7.61 (m, 2H), 7.30 – 7.27 (m, 2H), 6.54 (q, J = 8.4 Hz, 1H), 1.28 (s, 12H). ^{13}C NMR (101 MHz, $CDCl_3$) δ 142.0, 131.7, 131.2 (q, J = 33.4 Hz), 128.5 (q, J = 3.9, 1.9 Hz), 121.9 (q, J = 273.5 Hz), 118.8, 111.3, 85.2, 24.7. The signal of the α-B-carbon was not observed. ^{19}F NMR (377 MHz, $CDCl_3$) δ -57.76 (d, J = 8.3 Hz). ^{11}B NMR (128 MHz, $CDCl_3$) δ 29.73. IR (neat) ν (cm^{-1}): 2981.9, 2931.6, 2857.0, 2230.8, 1709.0, 1606.5, 1382.8, 1336.3, 1267.3, 1138.7, 980.3, 861.0, 624.3; HRMS (ESI): calcd for $C_{16}H_{17}BF_3NO_2Na^+$ [M + Na]$^+$ 346.1197; found 346.1195.

(*Z*)-2-(1-([1,1'-biphenyl]-4-yl)-3,3,3-trifluoroprop-1-en-1-yl)-4,4,5,5-tetramethyl-1,3,2-dioxaborolane (3e)

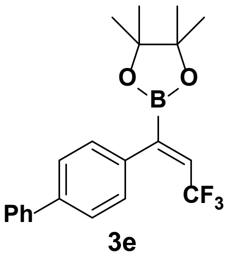

3e

Following the general procedure A, **3e** was synthesized from the corresponding alkyne (35.6 mg) and purified by silica gel

chromatography (EA/PE = 1/20); 41.1 mg (55%); white solid; mp 68-71 °C. ^{1}H NMR (400 MHz, $CDCl_3$) δ 7.64 – 7.55 (m, 5H), 7.45 – 7.41 (m, 2H), 7.36 – 7.28 (m, 3H), 6.48 (q, J = 8.7 Hz, 1H), 1.30 (s, 12H). ^{13}C NMR (101 MHz, $CDCl_3$) δ 140.7, 140.3, 135.9, 129.5 (q, J = 33.0 Hz), 128.7, 128.4 (q, J = 3.9, 1.8 Hz), 127.3, 127.0, 126.5, 122.4 (q, J = 273.5 Hz), 84.8, 24.7. The signal of the α-B-carbon was not observed. ^{19}F NMR (377 MHz, $CDCl_3$) δ -57.33 (d, J = 8.8 Hz). ^{11}B NMR (128 MHz, $CDCl_3$) δ 30.09. IR (neat) ν (cm^{-1}): 2980.0, 2929.7, 1686.6, 1604.6, 1487.2, 1373.5, 1326.9, 1265.4, 1116.3, 980.3, 848.0, 745.5, 693.3, 624.3; HRMS (ESI): calcd for $C_{21}H_{22}BF_3O_2Na^+$ [M + Na]$^+$ 397.1557; found 397.1544.

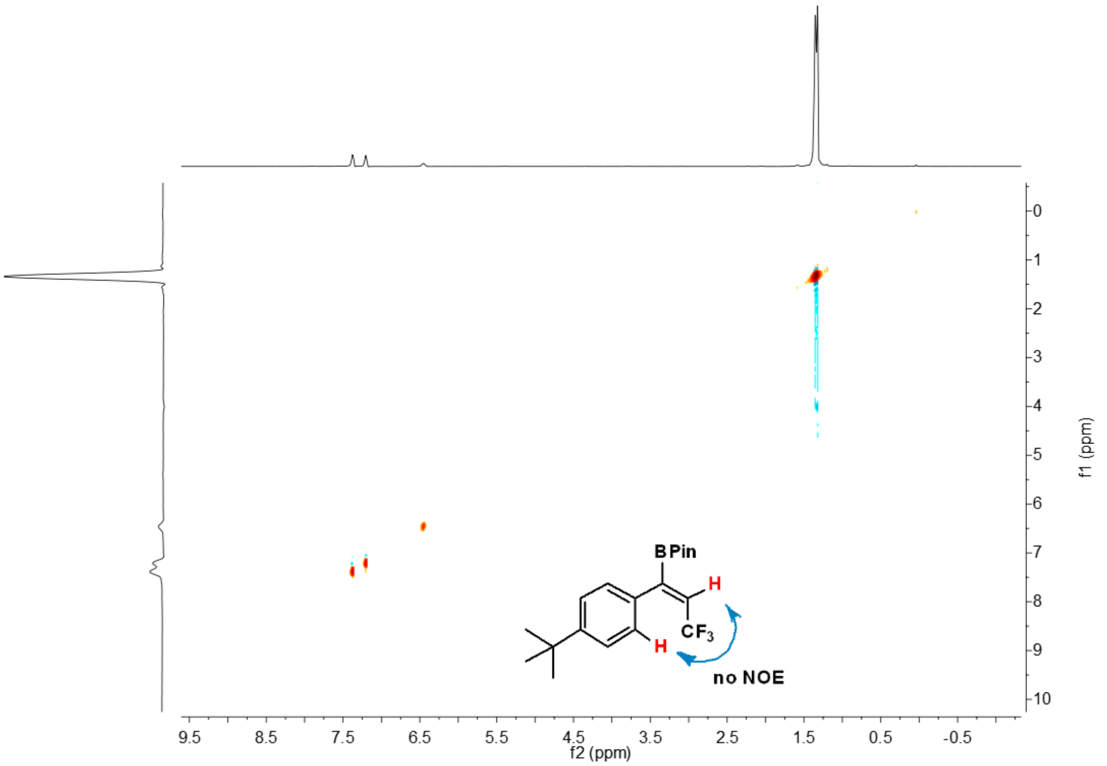

(*Z*)-2-(1-(4-(tert-butyl)phenyl)-3,3,3-trifluoroprop-1-en-1-yl)-4,4,5,5-tetramethyl-1,3,2-dioxaborolane (3f)

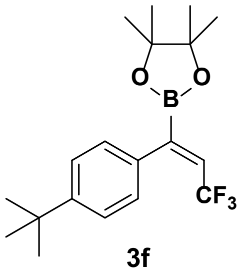

3f

Following the general procedure A, **3f** was synthesized from the corresponding alkyne (31.6 mg) and purified by silica gel chromatography (EA/PE = 1/30); 56.0 mg (79%); white solid; mp 62-65 °C. ^{1}H NMR (400 MHz, $CDCl_3$) δ 7.35 – 7.32 (m, 2H), 7.17 – 7.15 (m, 2H), 6.41 (q, J = 8.8 Hz, 1H), 1.32 (s, 59), 1.29 (s, 12H). ^{13}C NMR (101 MHz, $CDCl_3$) δ 150.4, 133.7, 128.9 (q, J = 33.0 Hz), 127.7 (q, J = 2.0

Hz), 124.7, 122.5 (q, J = 273.6 Hz), 84.7, 34.5, 31.3, 24.7. The signal of the α-B-carbon was not observed. ^{19}F NMR (377 MHz, $CDCl_3$) δ -57.27 (d, J = 8.7 Hz). ^{11}B NMR (128 MHz, $CDCl_3$) δ 29.97. IR (neat) ν (cm^{-1}): 2965.1, 2870.1, 1632.6, 1511.4, 1466.7, 1373.5, 1328.8, 1265.4, 1112.6, 1023.2, 982.2, 862.9, 626.2, 557.2; HRMS (ESI): calcd for $C_{19}H_{26}BF_3O_2Na^+$ $[M + Na]^+$ 377.1870; found 377.1871.

(*Z*)-methyl-4-(3,3,3-trifluoro-1-(4,4,5,5-tetramethyl-1,3,2-dioxaborolan-2-yl)prop-1-en-1-yl)benzoate (3g)

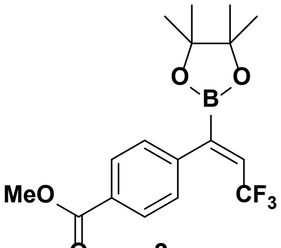

Following the general procedure A, **3g** was synthesized from the corresponding alkyne (32.1 mg) and purified by silica gel chromatography (EA/PE = 1/10); 34.9 mg (49%); white solid; mp 68-70 °C. ^{1}H NMR (400 MHz, $CDCl_3$) δ 8.02 – 7.99 (m, 2H), 7.27 – 7.24 (m, 2H), 6.51 (q, J = 8.4 Hz, 1H), 3.91 (s, 3H), 1.28 (s, 12H). ^{13}C NMR (101 MHz, $CDCl_3$) δ 166.9, 142.0, 130.5 (q, J = 33.2 Hz), 129.1, 129.1, 127.7 (q, J = 4.0, 2.0 Hz), 122.1 (q, J = 273.8 Hz), 85.0, 52.1, 24.7. The signal of the α-B-carbon was not observed. ^{19}F NMR (377 MHz, $CDCl_3$) δ -57.69 (d, J = 8.4 Hz). ^{11}B NMR (128 MHz, $CDCl_3$) δ 30.0. IR (neat) ν (cm^{-1}): 3393.7, 3183.1, 2920.4, 2849.5, 1716.4, 1645.6, 1436.9, 1382.8, 1332.5, 1269.2, 1190.9, 1112.6, 1023.2, 982.2, 861.0, 784.6, 732.4, 620.6; HRMS (ESI): calcd for $C_{17}H_{20}BF_3O_4Na^+$ $[M + Na]^+$ 379.1299; found 379.1294.

(*Z*)-4,4,5,5-tetramethyl-2-(3,3,3-trifluoro-1-(naphthalen-2-yl)prop-1-en-1-yl)-1,3,2-dioxaborolane (3h)

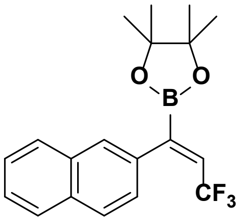

Following the general procedure A, **3h** was synthesized from the corresponding alkyne (30.4 mg) and purified by silica gel chromatography (EA/PE = 1/30); 30.1 mg (43%); light yellow oil. ^{1}H NMR (400 MHz, $CDCl_3$) δ 7.86 – 7.81 (m, 3H), 7.69 (s, 1H), 7.51 – 7.45 (m, 2H), 7.34 (dd, J = 8.5, 1.6 Hz, 1H), 6.56 (q, J = 8.6 Hz, 1H), 1.31 (s, 12H). ^{13}C NMR (101 MHz, $CDCl_3$) δ 134.6, 132.9, 132.6, 129.7 (q, J = 33.0 Hz), 128.2, 127.6, 127.3, 126.7 (q, J = 4.3, 2.1 Hz), 126.1, 126.0, 126.0, 122.4 (q, J = 273.7 Hz), 84.8, 24.7. The signal of the α-B-carbon was not observed. ^{19}F NMR (377 MHz, $CDCl_3$) δ -57.38 (d, J = 8.8 Hz). ^{11}B NMR (128 MHz, $CDCl_3$) δ 30.13. IR

(neat) ν (cm^{-1}): 3062.0, 2980.0, 2931.6, 1470.4, 1373.5, 1334.4, 1265.4, 1135.0, 959.8, 859.2, 816.3, 758.5, 650.4; HRMS (ESI): calcd for $C_{19}H_{20}BF_3O_2Na^+$ $[M + Na]^+$ 371.1401; found 371.1399.

(*Z*)-4,4,5,5-tetramethyl-2-(3,3,3-trifluoro-1-(thiophen-3-yl)prop-1-en-1-yl)-1,3,2-dioxaborolane (3i)

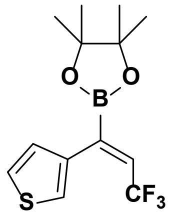

3i

Following the general procedure A, **3i** was synthesized from the corresponding alkyne (21.6 mg) and purified by silica gel chromatography (EA/PE = 1/20); 34.9 mg (49%); white solid; mp 53-55 °C. ^{1}H NMR (400 MHz, $CDCl_3$) δ 7.41 (dd, *J* = 3.0, 1.3 Hz, 1H), 7.28 – 7.26 (m, 1H), 7.16 – 7.14 (m, 1H), 1.31 (s, 12H). ^{13}C NMR (101 MHz, $CDCl_3$) δ 136.3, 128.5 (q, *J* = 2.5 Hz), 128.2 (q, *J* = 33.7 Hz), 125.2 (q, *J* = 2.7 Hz), 124.6, 122.5 (q, *J* = 273.1 Hz), 84.8, 24.7. The signal of the *α*-B-carbon was not observed. ^{19}F NMR (377 MHz, $CDCl_3$) δ -57.86 (d, *J* = 9.0 Hz). ^{11}B NMR (128 MHz, $CDCl_3$) δ 30.2. IR (neat) ν (cm^{-1}): 3360.2, 3185.0, 2980.0, 2920.4, 2849.5, 1627.0, 1470.4, 1336.3, 1258.0, 1116.3, 957.9, 859.2, 786.5, 739.9, 641.1; HRMS (ESI): calcd for $C_{13}H_{16}BF_3O_2SNa^+$ $[M + Na]^+$ 327.0808; found 327.0803.

(*Z*)-4,4,5,5-tetramethyl-2-(4-(3,3,3-trifluoro-1-(4,4,5,5-tetramethyl-1,3,2-dioxaborolan-2-yl)prop-1-en-1-yl)phenyl)-1,3,2-dioxaborolane (3j)

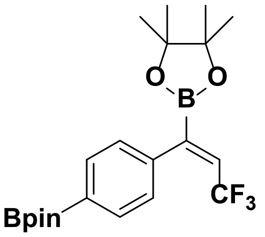

3j

Following the general procedure A, **3j** was synthesized from the corresponding alkyne (45.6 mg) and purified by silica gel chromatography (EA/PE = 1/20); 43.3 mg (51%); white solid; mp 127-129 °C. ^{1}H NMR (400 MHz, $CDCl_3$) δ 7.79 – 7.77 (m, 2H), 7.20 – 7.18 (m, 2H), 6.45 (q, *J* = 8.5 Hz, 1H), 1.34 (s, 12H), 1.27 (s, 12H). ^{13}C NMR (101 MHz, $CDCl_3$) δ 140.0, 134.2, 129.7 (q, *J* = 33.1 Hz), 127.0 (q, *J* = 3.8, 1.9 Hz), 122.3 (q, *J* = 273.7 Hz), 84.8, 83.7, 24.9, 24.7. The signal of the *α*-B-carbon was not observed. ^{19}F NMR (377 MHz, $CDCl_3$) δ -57.54 (d, *J* = 8.5 Hz). ^{11}B NMR (128 MHz, $CDCl_3$) δ 30.42. IR (neat) ν (cm^{-1}): 2983.7, 2929.7, 1610.2, 1470.4, 1360.5, 1330.7, 1263.6, 1189.0, 1120.1, 1021.3, 961.7, 857.3, 657.9; HRMS (ESI): calcd for $C_{21}H_{29}B_2F_3O_4Na^+$ $[M + Na]^+$ 447.2096; found 447.2093.

(*Z*)-2-(1-(4-(tert-butyl)phenyl)-3,3,3-trifluoroprop-1-en-1-yl-2-*d*)-4,4,5,5-tetramethyl-1,3,2-dioxaborolane (3k)

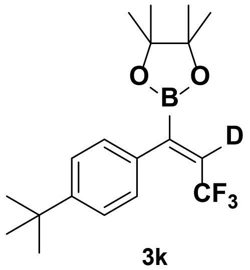

3k

Following the general procedure A, **3k** was synthesized from the corresponding alkyne (31.6 mg) and purified by silica gel chromatography (EA/PE = 1/30); 47.5 mg (63%); colorless liquid. ^{1}H NMR (400 MHz, $CDCl_3$) δ 7.35 – 7.32 (m, 2H), 7.17 – 7.15 (m, 2H), 1.32 (s, 9H), 1.29 (s, 12H); ^{13}C NMR (101 MHz, $CDCl_3$) δ 150.4, 133.7, 127.7 (q, *J* = 2.1 Hz), 124.7, 122.5 (q, *J* = 273.0 Hz), 84.7, 34.5, 31.3, 24.7 The signal of the α-B-carbon and the quadruplet of the α-CF_3-carbon were not observed. ^{19}F NMR (377 MHz, $CDCl_3$) δ -57.35; ^{11}B NMR (128 MHz, $CDCl_3$) δ 30.1; IR (neat) ν (cm^{-1}): 2965.1, 2870.1, 1507.7, 1463.0, 1366.1, 1334.4, 1265.4, 1166.7, 1108.9, 1008.2, 969.1, 922.5, 853.6. HRMS (ESI): calcd for $C_{19}H_{25}DBF_3O_2Na^+$ [M + Na]$^+$ 378.1933; found 378.1931.

(*Z*)-4,4,5,5-tetramethyl-2-(3,3,3-trifluoro-1-(m-tolyl)prop-1-en-1-yl-2-*d*)-1,3,2-dioxaborolane (3l)

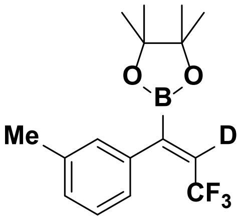

3l

Following the general procedure A, **3l** was synthesized from the corresponding alkyne (23.4 mg) and purified by silica gel chromatography (EA/PE = 1/30); 47.5 mg (63%); colorless liquid. ^{1}H NMR (400 MHz, $CDCl_3$) δ 7.24 – 7.20 (m, 1H), 7.11 – 7.09 (m, 1H), 7.00 – 6.99 (m, 2H), 2.35 (s, 3H), 1.28 (s, 12H); ^{13}C NMR (101 MHz, $CDCl_3$) δ 137.2, 136.9, 128.4 (q, *J* = 2.0 Hz), 128.3, 127.6, 124.8 (q, *J* = 2.1 Hz), 121.0 (q, *J* = 272.0 Hz), 84.7, 24.7, 21.5; ^{19}F NMR (377 MHz, $CDCl_3$) δ -57.50; ^{11}B NMR (128 MHz, $CDCl_3$) δ 29.86 The signal of the α-B-carbon and the quadruplet of the α-CF_3-carbon were not observed. IR (neat) ν (cm^{-1}): 2980.0, 2929.7, 1602.8, 1366.1, 1336.3, 1267.3, 1183.4, 1114.5, 1039.9, 967.2, 849.8, 672.8. HRMS (ESI): calcd for $C_{16}H_{19}DBF_3O_2Na^+$ [M + Na]$^+$ 336.1463; found 336.1464.

(*Z*)-2-(1-(2-chlorophenyl)-3,3,3-trifluoroprop-1-en-1-yl-2-*d*)-4,4,5,5-tetramethyl-1,3,2-dioxaborolane (3m)

Following the general procedure A, **3m** was synthesized from the corresponding alkyne (27.5 mg) and purified by silica gel chromatography (EA/PE = 1/30); 47.5 mg (63%);

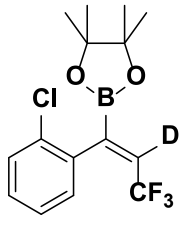

3m

colorless liquid. ^{1}H NMR (400 MHz, $CDCl_3$) δ 7.38 – 7.35 (m, 1H), 7.26 – 7.22 (m, 2H), 7.15 – 7.12 (m, 1H), 1.27 (s, 12H); ^{13}C NMR (101 MHz, $CDCl_3$) δ 136.3, 131.8, 129.1 (q, *J* = 2.3 Hz), 128.9, 128.8, 126.4, 122.1 (q, *J* = 274.3 Hz), 84.8, 24.6. The signal of the *α*-B-carbon and the quadruplet of the *α*-CF_3-carbon were not observed. ^{19}F NMR (377 MHz, $CDCl_3$) δ -58.93; ^{11}B NMR (128 MHz, $CDCl_3$) δ 29.70; IR (neat) ν (cm^{-1}): 2981.9, 2931.6, 1470.4, 1340.0, 1265.4, 1170.4, 1116.3, 1036.2, 967.2, 922.5, 849.8, 743.6. HRMS (EI): calcd for $C_{15}H_{16}DBClF_3O_2Na^+$ $[M + Na]^+$ 356.0917; found 356.0915.

(*Z*)-4,4,5,5-tetramethyl-2-(3,3,3-trifluoro-1-(4-fluorophenyl)prop-1-en-1-yl-2-*d*)-1,3,2-dioxaborolane (3n)

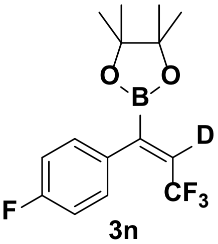

3n

Following the general procedure A, **3n** was synthesized from the corresponding alkyne (24.2 mg) and purified by silica gel chromatography (EA/PE = 1/30); 47.5 mg (63%); colorless liquid. ^{1}H NMR (400 MHz, $CDCl_3$) δ 7.20 – 7.17 (m, 2H), 7.05 – 6.99 (m, 2H), 1.29 (s, 12H); ^{13}C NMR (101 MHz, $CDCl_3$) δ 162.3 (d, *J* = 246.5 Hz), 129.6 (dq, *J* = 8.0, 4.2, 2.1 Hz), 122.3 (q, *J* = 273.3 Hz), 115.0, 114.8, 84.8, 24.7. The signal of the *α*-B-carbon and the quadruplet of the *α*-CF_3-carbon were not observed. ^{19}F NMR (377 MHz, $CDCl_3$) δ -57.58, -114.71 – -114.79 (m); ^{11}B NMR (128 MHz, $CDCl_3$) δ 30.06; IR (neat) ν (cm^{-1}): 2981.9, 2931.6, 1602.8, 1507.7, 1366.1, 1336.3, 1265.4, 1224.4, 1116.3, 1012.0, 969.1, 851.7, 758.5. HRMS (ESI): calcd for $C_{15}H_{16}DBF_4O_2Na^+$ $[M + Na]^+$ 340.1213; found 340.1204.

(*Z*)-4,4,5,5-tetramethyl-2-(3,3,3-trifluoro-2-methyl-1-phenylprop-1-en-1-yl)-1,3,2-dioxaborolane (3o)

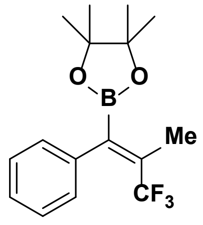

3o

Following the general procedure A, **3o** was synthesized from the corresponding alkyne (23.2 mg) and purified by silica gel chromatography (EA/PE = 1/30); 13.0 mg (43%); colorless liquid. ^{1}H NMR (400 MHz, $CDCl_3$) δ 7.31 – 7.20 (m, 3H), 7.13 – 7.10 (m, 2H), 2.11 (s, 3H), 1.25 (s, 12H). ^{13}C NMR (101 MHz, $CDCl_3$) δ 138.9, 133.5 (q, *J* = 27.8 Hz), 127.8, 127.2 (q, *J* = 1.9 Hz), 126.6, 123.5 (q, *J* = 277.7 Hz), 84.3,

24.6, 17.56 (q, J = 2.6 Hz). The signal of the α-B-carbon was not observed. ^{19}F NMR (377 MHz, $CDCl_3$) δ -60.49. ^{11}B NMR (128 MHz, $CDCl_3$) δ 30.30. IR (neat) ν (cm^{-1}): 2980.0, 2931.6, 1444.3, 1360.5, 1295.2, 1164.8, 1105.2, 1026.9, 965.4, 851.7, 749.2, 698.9, 672.8, 568.4; HRMS (ESI): calcd for $C_{16}H_{20}BF_3O_2Na^+$ $[M + Na]^+$ 335.1401; found 335.1402.

(*Z*)-4,4,5,5-tetramethyl-2-(1,1,1-trifluorooct-2-en-3-yl)-1,3,2-dioxaborolane (3p)

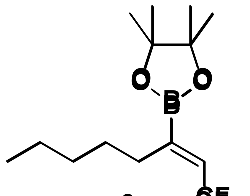

3p

Following the general procedure A, **3p** was synthesized from the corresponding alkyne (192.3 mg, 2 mmol) and purified by silica gel chromatography (EA/PE = 1/30); 12.1 mg (8%); light yellow oil. ^{1}H NMR (400 MHz, $CDCl_3$) δ 6.13 (q, J = 8.9 Hz, 1H), 2.27 (t, J = 7.2 Hz, 2H), 1.37 – 1.23 (m, 6H), 1.20 (s, 12H), 0.81 (t, J = 6.9 Hz, 3H); ^{13}C NMR (101 MHz, $CDCl_3$) δ 128.4 (q, J = 32.5 Hz), 123.0 (q, J = 273.8 Hz), 84.2, 31.7, 29.9, 29.1, 24.7, 22.4, 13.9. The signal of the α-B-carbon was not observed. ^{19}F NMR (377 MHz, $CDCl_3$) δ -58.31 (d, J = 8.9 Hz); ^{11}B NMR (128 MHz, $CDCl_3$) δ 30.24. IR (neat) ν (cm^{-1}): 2961.4, 29297, 2862.6, 1468.6, 1381.0, 1336.3, 1211.4, 1261.7, 1108.9, 961.7, 864.7, 691.4; HRMS (ESI): calcd for $C_{14}H_{24}BF_3O_2Na^+$ $[M + Na]^+$ 315.1714; found 315.1698.

(*Z*)-2-(1-(4-(tert-butyl)phenyl)-3,3,4,4,5,5,6,6,6-nonafluorohex-1-en-1-yl)-4,4,5,5-tetramethyl-1,3,2-dioxaborolane (3q)

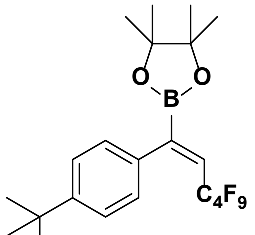

3q

Following the general procedure A, **3q** was synthesized from the corresponding alkyne (31.6 mg) and purified by silica gel chromatography (EA/PE = 1/30); 73.7 mg (73%); white solid; mp 59-61 °C. ^{1}H NMR (400 MHz, $CDCl_3$) δ 7.33 – 7.30 (m, 2H), 7.12 – 7.10 (m, 2H), 6.40 (t, J = 15.5 Hz, 1H), 1.32 (s, 9H), 1.28 (s, 12H). ^{13}C NMR (101 MHz, $CDCl_3$) δ 150.1, 134.0, 127.4 (t, J = 2.9 Hz), 126.0 (t, J = 20.3 Hz), 124.5, 84.7, 34.5, 31.3, 24.7. The signal of the α-B-carbon was not observed. ^{19}F NMR (377 MHz, $CDCl_3$) δ -81.04 (tt, J = 9.7, 2.7 Hz), -105.27 (q, J = 13.3 Hz), -123.75 – -123.82 (m), -125.72 – -125.83 (m). ^{11}B NMR (128 MHz, $CDCl_3$) δ 30.28. IR (neat) ν (cm^{-1}): 2968.8, 2931.6, 2871.9, 1686.6, 1604.6, 1459.3, 1334.4, 1230.0,

1131.2, 980.3, 881.5, 851.7, 702.6, 577.7; HRMS (ESI): calcd for $C_{22}H_{26}BF_9O_2Na^+$ [M + Na]$^+$ 527.1774; found 527.1764.

(*Z*)-2-(1-(4-(tert-butyl)phenyl)-3,3,4,4,5,5,6,6,7,7,8,8,8-tridecafluorooct-1-en-1-yl)-4,4,5,5-tetramethyl-1,3,2-dioxaborolane (3r)

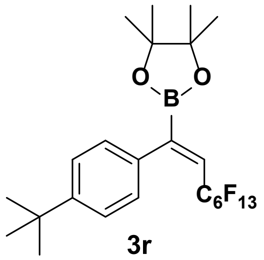
3r

Following the general procedure A, **3r** was synthesized from the corresponding alkyne (31.6 mg) and purified by silica gel chromatography (EA/PE = 1/30); 73.6 mg (61%); light yellow oil. ^{1}H NMR (500 MHz, $CDCl_3$) δ 7.33 – 7.30 (m, 2H), 7.12 – 7.10 (m, 2H), 6.40 (t, J = 15.5 Hz, 1H), 1.32 (s, 9H), 1.28 (s, 12H). ^{13}C NMR (126 MHz, $CDCl_3$) δ 150.0, 134.0, 127.3 (t, J = 2.7 Hz), 126.1 (t, J = 20.5 Hz), 124.5, 84.7, 34.5, 31.3, 24.7. The signal of the α-B-carbon was not observed. ^{19}F NMR (377 MHz, $CDCl_3$) δ -80.77 – -80.82 (m), -105.04 (q, J = 13.7 Hz), -121.62 – -121.70 (m), -122.80 (s), -126.07 – -126.18 (m). ^{11}B NMR (128 MHz, $CDCl_3$) δ 30.20. IR (neat) ν (cm^{-1}): 2965.1, 2931.6, 2858.9, 1686.6, 1604.6, 1459.3, 1340.0, 1235.6, 1142.4, 982.2, 559.1; HRMS (ESI): calcd for $C_{24}H_{26}BF_{13}O_2Na^+$ [M + Na]$^+$ 627.1710; found 627.1709.

(Z)-2-(1-(4-(tert-butyl)phenyl)-3,3,4,4,5,5,6,6,7,7,8,8,9,9,10,10,10-heptadecafluorodec-1-en-1-yl)-4,4,5,5-tetramethyl-1,3,2-dioxaborolane (3s)

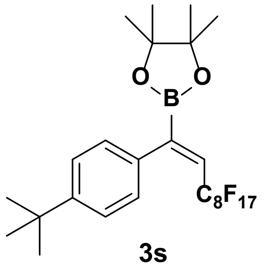
3s

Following the general procedure A, **3s** was synthesized from the corresponding alkyne (31.6 mg) and purified by silica gel chromatography (EA/PE = 1/30); 95.7 mg (68%); light yellow oil. ^{1}H NMR (400 MHz, $CDCl_3$) δ 7.33 – 7.30 (m, 2H), 7.12 - 7.10 (m, 2H), 6.40 (t, J = 15.5 Hz, 1H), 1.32 (s, 9H), 1.28 (s, 12H). ^{13}C NMR (101 MHz, $CDCl_3$) δ 150.1, 134.0, 127.4 (t, J = 2.7 Hz), 126.2 (t, J = 20.5 Hz), 124.5, 84.7, 34.5, 31.3, 24.7. The signal of the α-B-carbon was not observed. ^{19}F NMR (377 MHz, $CDCl_3$) δ -80.74 – -80.79 (m), -105.04 (q, J = 14.1 Hz), -121.44 (s), -121.89 (s), -122.74 (s), -126.06 – -126.17 (m). ^{11}B NMR (128 MHz, $CDCl_3$) δ 30.38. IR (neat) ν (cm^{-1}): 2967.0, 2929.7, 2860.7, 1686.6, 1604.6, 1459.3, 1336.3, 1202.1, 1144.3, 982.2, 851.7, 704.5, 657.9, 557.2; HRMS (ESI): calcd for $C_{26}H_{26}BF_{17}O_2Na^+$ [M + Na]$^+$ 727.1647; found 727.1648.

(*Z*)-2-(1-(4-(tert-butyl)phenyl)-4-((8-chloro-1,1,2,2,3,3,4,4,5,5,6,6,7,7,8,8-hexadeca fluorooctyl)oxy)-3,3,4,4-tetrafluorobut-1-en-1-yl)-4,4,5,5-tetramethyl-1,3,2-dioxa borolane (3t)

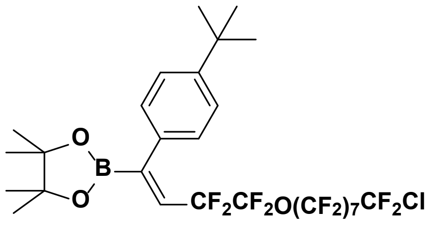

3t

Following the general procedure A, **3t** was synthesized from the corresponding alkyne (31.6 mg) and purified by silica gel chromatography (EA/PE = 1/30); 108.6 mg (65%); light yellow oil. ^{1}H NMR (400 MHz, $CDCl_3$) δ 7.26 – 7.23 (m, 2H), 7.05 – 7.03 (m, 2H), 6.27 (t, *J* = 15.2 Hz, 1H), 1.25 (s, 9H), 1.20 (s, 12H). ^{13}C NMR (101 MHz, $CDCl_3$) δ 150.2, 133.9, 127.4 (t, *J* = 2.9 Hz), 125.7 (t, *J* = 20.1 Hz), 124.5, 84.7, 34.5, 31.3, 24.6. The signal of the *α*-B-carbon was not observed. ^{19}F NMR (377 MHz, $CDCl_3$) δ -68.05 (q, *J* = 13.6 Hz), -83.10 – -83.22 (m), -87.32 – -87.39 (m), -108.86 (dt, *J* = 15.0, 2.7 Hz), -120.10 (s), -121.17 (s), -121.79 (s), -122.05 (s), -125.50 (s). ^{11}B NMR (128 MHz, $CDCl_3$) δ 30.28. IR (neat) ν (cm^{-1}): 2968.8, 2931.6, 2860.7, 1686.6, 1606.5, 1459.3, 1332.5, 1213.2, 1140.6, 1032.5, 976.6, 853.6, 654.1, 549.8; HRMS (ESI): calcd for $C_{28}H_{27}BClF_{20}O_3^+$ $[M + H]^+$ 837.1417; found 837.1408.

6,6,6-trifluoro-4-(4,4,5,5-tetramethyl-1,3,2-dioxaborolan-2-yl)hexyl 4-chlorobenzoate (5a)

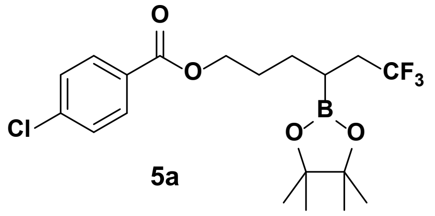

5a

Following the general procedure B, **5a** was synthesized from the corresponding alkene (44.9 mg) and purified by silica gel chromatography (EA/PE = 1/20); 47.6 mg (57%); colorless liquid. ^{1}H NMR (400 MHz, $CDCl_3$) δ 7.99 – 7.95 (m, 2H), 7.43 – 7.39 (m, 2H), 4.31 (t, *J* = 6.4 Hz, 2H), 2.38 – 2.25 (m, 1H), 2.19 – 2.04 (m, 1H), 1.84 – 1.73 (m, 2H), 1.70 – 1.55 (m, 2H), 1.40 – 1.31 (m, 1H), 1.24 (s, 12H). ^{13}C NMR (101 MHz, $CDCl_3$) δ 165.7, 139.3, 130.9, 128.7, 128.7, 127.3 (q, *J* = 277.0 Hz), 83.7, 65.1, 35.0 (q, *J* = 28.0 Hz), 27.7, 27.2, 24.7, 24.7. The signal of the *α*-B-carbon was not observed. ^{19}F NMR (377 MHz, $CDCl_3$) δ -64.90 (t, *J* = 11.2 Hz). ^{11}B NMR (128 MHz, $CDCl_3$) δ 33.92. IR (neat) ν (cm^{-1}): 2980.0, 2935.3, 1720.2, 1595.3, 1373.5, 1269.2, 1136.8, 1090.2, 1015.7, 851.7,

758.5, 685.8; HRMS (ESI): calcd for $C_{19}H_{25}BClF_3O_4Na^+$ $[M + Na]^+$ 443.1379; found 443.1378.

6,6,6-trifluoro-4-(4,4,5,5-tetramethyl-1,3,2-dioxaborolan-2-yl)hexyl 3,5-dimethylbenzoate (5b)

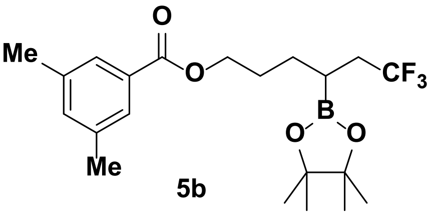

Following the general procedure B, **5b** was synthesized from the corresponding alkene (43.7 mg) and purified by silica gel chromatography (EA/PE = 1/20); 39.1 mg (47%); colorless liquid.^{1}H NMR (400 MHz, $CDCl_3$) δ 7.64 (s, 2H), 7.18 (s, 1H), 4.29 (t, J = 6.4 Hz, 2H), 2.42 – 2.24 (m, 7H), 2.22 – 2.04 (m, 1H), 1.86 – 1.73 (m, 2H), 1.72 – 1.54 (m, 3H), 1.41 – 1.31 (m, 1H), 1.24 (s, 12H); ^{13}C NMR (101 MHz, $CDCl_3$) δ 166.9, 138.0, 134.5, 130.2, 127.2, 127.4 (q, J = 277.1 Hz), 83.7, 64.7, 35.0 (q, J = 27.9 Hz), 27.8, 27.3, 24.7, 24.6, 21.1. The signal of the α-B-carbon was not observed. ^{19}F NMR (377 MHz, $CDCl_3$) δ -64.94 (t, J = 11.2 Hz); ^{11}B NMR (128 MHz, $CDCl_3$) δ 33.92 (s); IR (neat) ν (cm^{-1}): 2980.0, 2935.3, 2866.3, 1716.4, 1608.3, 1373.5, 1308.3, 1211.4, 1135.0, 1071.6, 864.7, 767.8; HRMS (ESI): calcd for $C_{21}H_{30}BF_3O_4Na^+$ $[M + Na]^+$ 437.2081; found 437.2079.

7,7,7-trifluoro-5-(4,4,5,5-tetramethyl-1,3,2-dioxaborolan-2-yl)heptyl 4-methoxybenzoate (5c)

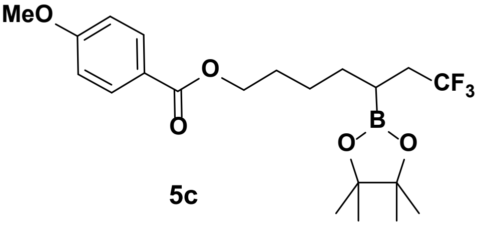

Following the general procedure B, **5c** was synthesized from the corresponding alkene (46.9 mg) and purified by silica gel chromatography (EA/PE = 1/20); 53.1 mg (62%); colorless liquid. ^{1}H NMR (400 MHz, $CDCl_3$) δ 8.00 – 7.97 (m, 2H), 6.93 – 6.89 (m, 2H), 4.28 (t, J = 6.5 Hz, 2H), 3.86 (s, 3H), 2.37 – 2.23 (m, 1H), 2.15 – 2.02 (m, 1H), 1.81 – 1.72 (m, 2H), 1.63 – 1.42 (m, 4H), 1.38 – 1.28 (m, 1H), 1.22 (s, 12H). ^{13}C NMR (101 MHz, $CDCl_3$) δ 166.4, 163.3, 131.5, 127.4 (q, J = 277.0 Hz), 122.8, 113.5, 83.5, 64.5, 55.4, 35.2 (q, J = 27.9 Hz), 30.6, 28.8, 25.2, 24.7, 24.6. The signal of the α-B-carbon was not observed. ^{19}F NMR (377 MHz, $CDCl_3$) δ -64.99 (t, J = 11.2 Hz).

^{11}B NMR (128 MHz, $CDCl_3$) δ 34.44. IR (neat) ν (cm^{-1}): 2978.1, 2937.1, 2860.7, 1710.8, 1606.5, 1511.4, 1373.5, 1326.9, 1252.4, 1135.0, 1030.6, 848.0, 769.7; HRMS (ESI): calcd for $C_{21}H_{30}BF_3O_5Na^+$ $[M + Na]^+$ 453.2031; found 453.2031.

7,7,7-trifluoro-5-(4,4,5,5-tetramethyl-1,3,2-dioxaborolan-2-yl)heptyl 4-methylbenzoate (5d)

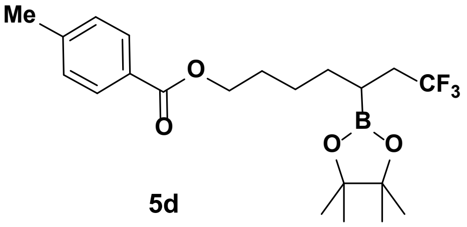

5d

Following the general procedure B, **5d** was synthesized from the corresponding alkene (43.7 mg) and purified by silica gel chromatography (EA/PE = 1/20); 45.8 mg (55%); colorless liquid. ^1H NMR (400 MHz, $CDCl_3$) δ 7.93 – 7.91 (m, 2H), 7.23 – 7.21 (m, 2H), 4.29 (t, J = 6.5 Hz, 2H), 2.41 (s, 3H), 2.37 – 2.22 (m, 1H), 2.09 (m, 1H), 1.81 – 1.73 (m, 2H), 1.59 – 1.44 (m, 4H), 1.36 – 1.27 (m, 1H), 1.22 (s, 12H). ^{13}C NMR (126 MHz, $CDCl_3$) δ 166.7, 143.4, 129.5, 129.0, 127.6, 127.4 (q, J = 277.0 Hz), 83.6, 64.6, 35.2 (q, J = 27.9 Hz), 30.6, 28.8, 25.2, 24.7, 24.6, 21.6. The signal of the α-B-carbon was not observed. ^{19}F NMR (377 MHz, $CDCl_3$) δ -64.99 (t, J = 11.2 Hz). ^{11}B NMR (128 MHz, $CDCl_3$) δ 33.63. IR (neat) ν (cm^{-1}): 2980.0, 2933.4, 2862.6, 1716.4, 1612.1, 1373.5, 1271.0, 1136.8, 1107.0, 1021.3, 963.5, 862.9, 754.8, 691.4; HRMS (ESI): calcd for $C_{21}H_{30}BF_3O_4Na^+$ $[M + Na]^+$ 437.2081; found 437.2082.

7,7,7-trifluoro-5-(4,4,5,5-tetramethyl-1,3,2-dioxaborolan-2-yl)heptyl 2-naphthoate (5e)

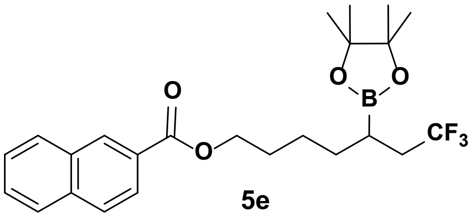

5e

Following the general procedure B, **5e** was synthesized from the corresponding alkene (50.9 mg) and purified by silica gel chromatography (EA/PE = 1/20); 40.8 mg (45%); white solid; mp 50-52 °C. ^1H NMR (400 MHz, $CDCl_3$) δ 8.59 (s, 1H), 8.05 (dd, J = 8.6, 1.7 Hz, 1H), 7.96 – 7.86 (m, 3H), 7.61 – 7.52 (m, 2H), 4.38 (t, J = 6.5 Hz, 2H), 2.39 – 2.23 (m, 1H), 2.11 (m, 1H), 1.88 – 1.79 (m, 2H), 1.57 – 1.48 (m, 4H), 1.37 – 1.30 (m, 1H), 1.21 (s, 12H). ^{13}C NMR (126 MHz, $CDCl_3$) δ 166.8, 135.5, 132.5, 130.9,

129.3, 128.2, 128.1, 127.7, 127.6, 127.4 (q, J = 277.1 Hz), 126.6, 125.2, 83.6, 65.0, 35.2 (q, J = 27.9 Hz), 30.6, 28.9, 25.2, 24.7, 24.6. The signal of the α-B-carbon was not observed. ^{19}F NMR (376 MHz, $CDCl_3$) δ -64.96 (t, J = 11.2 Hz). ^{11}B NMR (160 MHz, $CDCl_3$) δ 34.27. IR (neat) ν (cm^{-1}): 2980.0, 2935.3, 2860.7, 1718.3, 1630.7, 1459.3, 1373.5, 1323.2, 1259.8, 1220.7, 1129.4, 1075.3, 967.2, 866.6, 779.0, 695.1, 587.1; HRMS (ESI): calcd for $C_{24}H_{30}BF_3O_4Na^+$ $[M + Na]^+$ 473.2081; found 473.2083.

12,12,12-trifluoro-1-(indolin-1-yl)-10-(4,4,5,5-tetramethyl-1,3,2-dioxaborolan-2-yl)dodecan-1-one (5f)

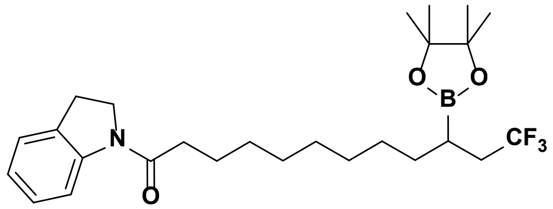

5f

Following the general procedure B, **5f** was synthesized from the corresponding alkene (57.1 mg) and purified by silica gel chromatography (EA/PE = 1/10); 51.7 mg (54%); colorless liquid. ^{1}H NMR (400 MHz, $CDCl_3$) δ 8.24 (d, J = 8.0 Hz, 1H), 7.20 (m, 2H), 7.02 – 6.98 (m, 1H), 4.04 (t, J = 8.5 Hz, 2H), 3.19 (t, J = 8.5 Hz, 2H), 2.40 (t, J = 7.5 Hz, 2H), 2.35 – 2.00 (m, 2H), 1.77 – 1.68 (m, 2H), 1.50 – 1.30 (m, 13H), 1.24 (s, 12H). ^{13}C NMR (126 MHz, $CDCl_3$) δ 171.4, 143.1, 131.0, 127.5, 127.5 (q, J = 277.0 Hz), 124.4, 123.4, 117.0, 83.4, 47.9, 36.0, 35.2 (q, J = 27.8 Hz), 30.9, 29.6, 29.4, 29.4, 29.3, 28.5, 28.0, 24.7, 24.6, 24.5. The signal of the α-B-carbon was not observed. ^{19}F NMR (376 MHz, $CDCl_3$) δ -65.05 (t, J = 11.2 Hz). ^{11}B NMR (128 MHz, $CDCl_3$) δ 34.66. IR (neat) ν (cm^{-1}): 2980.0, 2929.7, 2857.0, 1664.3, 1483.5, 1403.3, 1328.8, 1254.2, 1213.2, 1135.0, 1053.0, 862.9, 764.1, 695.1; HRMS (ESI): calcd for $C_{26}H_{39}BF_3NO_3Na^+$ $[M + Na]^+$ 504.2867; found 504.2865.

2-(6,6,6-trifluoro-4-(4,4,5,5-tetramethyl-1,3,2-dioxaborolan-2-yl)hexyl)isoindoline-1,3-dione (5g)

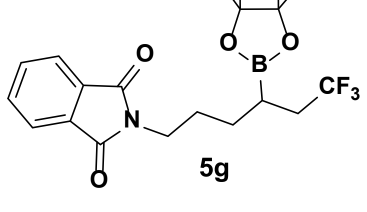

5g

Following the general procedure B, **5g** was synthesized from the corresponding alkene (43.1 mg) and purified by silica gel chromatography (EA/PE = 1/10); 40.8 mg

(57%); white solid; mp 65-67 °C. ^{1}H NMR (400 MHz, $CDCl_3$) δ 7.86 – 7.82 (m, 2H), 7.73 – 7.69 (m, 2H), 3.68 (t, J = 7.1 Hz, 2H), 2.35 – 2.20 (m, 1H), 2.15 – 2.00 (m, 1H), 1.76 – 1.66 (m, 2H), 1.59 – 1.43 (m, 2H), 1.37 – 1.28 (m, 1H), 1.22 (s, 12H). ^{13}C NMR (126 MHz, $CDCl_3$) δ 168.4, 133.9, 132.1, 127.3 (q, J = 277.0 Hz), 123.2, 83.7, 37.9, 35.2 (q, J = 27.9 Hz), 28.1, 27.6, 24.7, 24.6. The signal of the α-B-carbon was not observed. ^{19}F NMR (376 MHz, $CDCl_3$) δ -65.01 (t, J = 11.2 Hz). ^{11}B NMR (128 MHz, $CDCl_3$) δ 34.09. IR (neat) ν (cm^{-1}): 2980.0, 2935.3, 2860.7, 1764.9, 1705.3, 1392.2, 1330.7, 1252.4, 1131.2, 1060.4, 862.9, 717.5; HRMS (ESI): calcd for $C_{20}H_{25}BF_3NO_4Na^+$ $[M + Na]^+$ 434.1721; found 434.1719.

6,6,6-trifluoro-N-(2-fluorophenyl)-N-methyl-4-(4,4,5,5-tetramethyl-1,3,2-dioxaborolan-2-yl)hexanamide (5h)

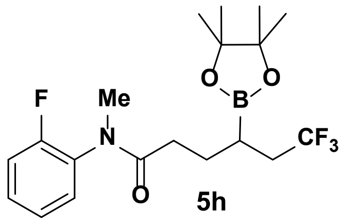

5h

Following the general procedure B, **5h** was synthesized from the corresponding alkene (41.1 mg) and purified by silica gel chromatography (EA/PE = 1/5); 45.6 mg (57%); colorless liquid. dr = 1:1; ^{1}H NMR (400 MHz, $CDCl_3$, mixture of diastereomers) δ 7.37 – 7.15 (m, 4H), 3.22 (s, 3H), 2.34 – 1.59 (m, 7H), 1.14 – 1.08 (m, 12H). ^{13}C NMR (126 MHz, $CDCl_3$, asterisk denotes minor diastereomers peaks) δ 172.6, 172.6*, 158.0 (d, J = 250.0 Hz), 131.4 (d, J = 14.9 Hz), 129.8 (d, J = 7.8 Hz), 129.6* (d, J = 15.1 Hz), 127.20 (q, J = 277.0 Hz), 125.1, 120.6*, 117.0 (d, J = 8.3 Hz), 116.9* (d, J = 8.0 Hz), 115.26, 83.5, 36.5, 35.3* (q, J = 28.2 Hz), 35.2 (q, J = 28.1 Hz), 32.6, 32.5*, 26.4, 26.3*, 24.6*, 24.6, 24.5. ^{19}F NMR (377 MHz, $CDCl_3$, mixture of diastereomers) δ -64.95 – -65.07 (m), -121.30 – -121.51 (m). ^{11}B NMR (128 MHz, $CDCl_3$) δ 34.07. IR (neat) ν (cm^{-1}): 2980.0, 2935.3, 1735.1, 1664.3, 1608.3, 1504.0, 1457.4, 1373.5, 1328.8, 1258.0, 1131.2, 862.9, 762.2, 672.8, 575.9; HRMS (ESI): calcd for $C_{19}H_{27}BF_4NO_3^+$ $[M + H]^+$ 404.2015; found 404.2014.

7,7,7-trifluoro-5-(4,4,5,5-tetramethyl-1,3,2-dioxaborolan-2-yl)heptyl furan-2-carboxylate (5i)

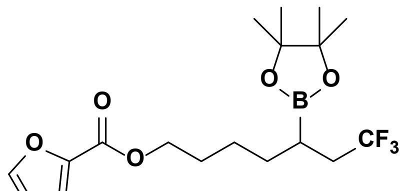

5i

Following the general procedure B, **5i** was synthesized from the corresponding alkene (38.8 mg) and purified by silica gel chromatography (EA/PE = 1/20); 39.7 mg (51%); colorless liquid. ^{1}H NMR (400 MHz, $CDCl_3$) δ 7.57 – 7.56 (m, 1H), 7.16 (dd, J = 3.5, 0.6 Hz, 1H), 6.50 (dd, J = 3.5, 1.7 Hz, 1H), 4.30 (t, J = 6.6 Hz, 2H), 2.37 – 2.22 (m, 1H), 2.15 – 2.02 (m, 1H), 1.80 – 1.71 (m, 2H), 1.58 – 1.41 (m, 4H), 1.35 – 1.27 (m, 1H), 1.23 (s, 12H). ^{13}C NMR (126 MHz, $CDCl_3$) δ 158.8, 146.2, 144.8, 127.4 (q, J = 276.9 Hz), 117.7, 111.8, 83.6, 64.7, 35.1 (q, J = 27.9 Hz), 30.5, 28.7, 25.0, 24.7, 24.6. The signal of the α-B-carbon was not observed. ^{19}F NMR (376 MHz, $CDCl_3$) δ -65.00 (t, J = 11.2 Hz). ^{11}B NMR (128 MHz, $CDCl_3$) δ 33.67. IR (neat) ν (cm^{-1}): 2980.0, 2935.3, 2864.5, 1720.2, 1582.3, 1474.2, 1373.5, 1293.4, 1256.1, 1116.3, 1075.3, 1012.0, 963.5, 862.9, 762.2; HRMS (ESI): calcd for $C_{18}H_{26}BF_3O_5Na^+$ $[M + Na]^+$ 413.1718; found 413.1716.

7,7,7-trifluoro-5-(4,4,5,5-tetramethyl-1,3,2-dioxaborolan-2-yl)heptyl 4-bromothiophene-2-carboxylate (5j)

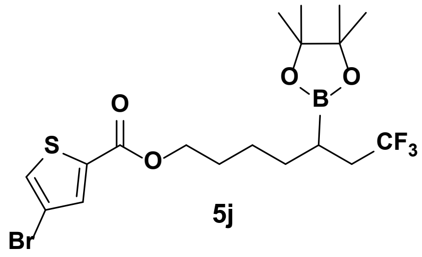

5j

Following the general procedure B, **5j** was synthesized from the corresponding alkene (57.8 mg) and purified by silica gel chromatography (EA/PE = 1/20); 36.2 mg (37%); colorless liquid. ^{1}H NMR (400 MHz, $CDCl_3$) δ 7.67 (d, J = 1.5 Hz, 1H), 7.44 (d, J = 1.5 Hz, 1H), 4.29 (t, J = 6.5 Hz, 2H), 2.38 – 2.23 (m, 1H), 2.16 – 2.01 (m, 1H), 1.81 – 1.69 (m, 2H), 1.56 – 1.40 (m, 4H), 1.34 – 1.27 (m, 1H), 1.23 (s, 12H). ^{13}C NMR (126 MHz, $CDCl_3$) δ 161.0, 135.2, 134.9, 129.4, 127.4 (q, J = 276.9 Hz), 110.5, 83.6, 65.4, 35.1 (q, J = 27.9 Hz), 30.5, 28.7, 25.0, 24.7, 24.6. The signal of the α-B-carbon was not observed. ^{19}F NMR (376 MHz, $CDCl_3$) δ -64.96 (t, J = 11.2 Hz). ^{11}B NMR (128 MHz, $CDCl_3$) δ 34. 42. IR (neat) ν (cm^{-1}): 3103.0, 2980.0, 2935.3, 2860.7, 1712.7, 1517.0, 1407.1, 1328.8, 1246.8, 1135.0, 1097.7, 1064.2, 963.5, 862.9, 769.7, 579.6; HRMS (ESI): calcd for $C_{18}H_{25}BBrF_3O_4SNa^+$ $[M + Na]^+$ 507.0594; found 507.0592.

7,7,7-trifluoro-5-(4,4,5,5-tetramethyl-1,3,2-dioxaborolan-2-yl)heptyl 4-methylbenzenesulfonate (5k)

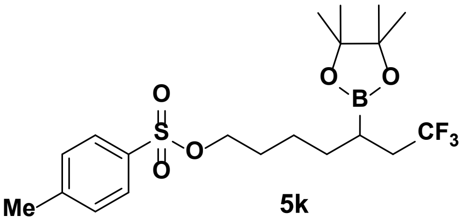

5k

Following the general procedure B, **5k** was synthesized from the corresponding alkene (50.9 mg) and purified by silica gel chromatography (EA/PE = 1/10); 55.5 mg (62%); colorless liquid. ^{1}H NMR (400 MHz, $CDCl_3$) δ 7.80 – 7.77 (m, 2H), 7.35 – 7.33 (m, 2H), 4.02 (t, J = 6.4 Hz, 2H), 2.45 (s, 3H), 2.32 – 2.16 (m, 1H), 2.08 – 1.94 (m, 1H), 1.70 – 1.58 (m, 2H), 1.48 – 1.29 (m, 4H), 1.27 – 1.18 (m, 13H). ^{13}C NMR (126 MHz, $CDCl_3$) δ 144.7, 133.1, 129.8, 127.9, 127.3 (q, J = 277.0 Hz), 83.6, 70.2, 35.1 (q, J = 27.9 Hz), 30.0, 28.8, 24.7, 24.6, 24.4, 21.6. The signal of the α-B-carbon was not observed. ^{19}F NMR (376 MHz, $CDCl_3$) δ -64.99 (t, J = 11.2 Hz). ^{11}B NMR (128 MHz, $CDCl_3$) δ 33.56. IR (neat) ν (cm^{-1}): 2980.0, 2933.4, 2864.5, 1599.0, 1459.3, 1358.6, 1328.8, 1256.1, 1176.0, 1135.0, 1073.5, 937.4, 814.4, 661.6, 575.9, 553.5; HRMS (ESI): calcd for $C_{20}H_{30}BF_3O_5SNa^+$ $[M + Na]^+$ 473.1751; found 473.1750.

4-((6,6,6-trifluoro-4-(4,4,5,5-tetramethyl-1,3,2-dioxaborolan-2-yl)hexyl)oxy)-2*H*-chromen-2-one (5l)

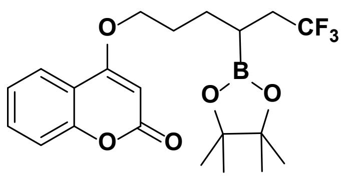

5l

Following the general procedure B, **5l** was synthesized from the corresponding alkene (46.1 mg) and purified by silica gel chromatography (EA/PE = 1/5); 50.6 mg (59%); white solid; mp 61-63 °C. ^{1}H NMR (400 MHz, $CDCl_3$) δ 7.84 – 7.80 (m, 1H), 7.58 – 7.53 (m, 1H), 7.34 – 7.25 (m, 2H), 5.67 (s, 1H), 4.14 (t, J = 6.2 Hz, 2H), 2.45 – 2.29 (m, 1H), 2.24 – 2.08 (m, 1H), 2.02 – 1.87 (m, 2H), 1.81 – 1.67 (m, 2H), 1.43 – 1.32 (m, 1H), 1.26 (s, 12H). ^{13}C NMR (126 MHz, $CDCl_3$) δ 165.6, 162.9, 153.3, 132.4, 127.3 (q, J = 277.2 Hz), 123.8, 123.0, 116.8, 115.7, 90.4, 83.8, 69.1, 35.1 (q, J = 28.1 Hz), 27.6, 27.1, 24.8, 24.7. The signal of the α-B-carbon was not observed . ^{19}F NMR (376 MHz, $CDCl_3$) δ -64.80 (t, J = 11.2 Hz). ^{11}B NMR (128 MHz, $CDCl_3$) δ 34. 51. IR (neat) ν (cm^{-1}): 3091.8, 2978.1, 2935.3, 2858.9, 1716.4, 1623.3,

1567.3, 1456.6, 1373.5, 1328.8, 1222.6, 1135.0, 1073.5, 933.7, 859.2, 747.3, 693.3, 575.8; HRMS (ESI): calcd for $C_{21}H_{26}BF_3O_5Na^+$ $[M + Na]^+$ 449.1718; found 449.1716.

4-methyl-7-((6,6,6-trifluoro-4-(4,4,5,5-tetramethyl-1,3,2-dioxaborolan-2-yl)hexyl) oxy)-2H-chromen-2-one (5m)

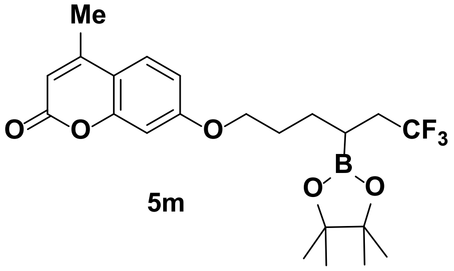

5m

Following the general procedure B, **5m** was synthesized from the corresponding alkene (48.9 mg) and purified by silica gel chromatography (EA/PE = 1/5); 49.9 mg (57%); white solid; mp 44-46 °C. ^{1}H NMR (400 MHz, $CDCl_3$) δ 7.51 – 7.47 (m, 1H), 6.86 – 6.79 (m, 2H), 6.13 (d, J = 1.1 Hz, 1H), 4.02 (t, J = 6.3 Hz, 2H), 2.40 – 2.27 (m, 4H), 2.20 – 2.08 (m, 1H), 1.89 – 1.81 (m, 2H), 1.70 – 1.61 (m, 2H), 1.42 – 1.30 (m, 1H), 1.25 (s, 12H). ^{13}C NMR (126 MHz, $CDCl_3$) δ 162.1, 161.4, 155.2, 152.6, 127.4 (q, J = 277.0 Hz), 125.5, 113.5, 112.6, 111.9, 101.3, 83.7, 68.2, 35.1 (q, J = 28.0 Hz), 28.0, 27.1, 24.7, 24.7, 18.7. The signal of the α-B-carbon was not observed . ^{19}F NMR (376 MHz, $CDCl_3$) δ -64.89 (t, J = 11.0 Hz). ^{11}B NMR (128 MHz, $CDCl_3$) δ 34.28. IR (neat) ν (cm^{-1}): 2980.0, 2937.1, 2873.8, 1718.3, 1610.2, 1509.6, 1388.4, 1328.8, 1258.0, 1135.0, 1069.7, 834.9, 706.3; HRMS (ESI): calcd for $C_{22}H_{28}BF_3O_5Na^+$ $[M + Na]^+$ 463.1874; found 463.1876.

2-phenyl-3-((6,6,6-trifluoro-4-(4,4,5,5-tetramethyl-1,3,2-dioxaborolan-2-yl)hexyl) oxy)-4H-chromen-4-one (5n)

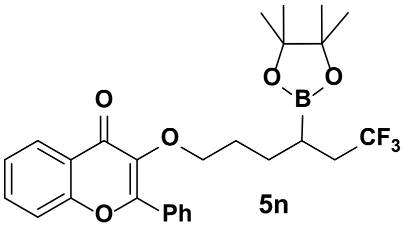

5n

Following the general procedure B, **5n** was synthesized from the corresponding alkene (61.3 mg) and purified by silica gel chromatography (EA/PE = 1/5); 52.1 mg (52%); colorless liquid. ^{1}H NMR (400 MHz, $CDCl_3$) δ 8.26 (dd, J = 8.0, 1.5 Hz, 1H), 8.11 – 8.06 (m, 2H), 7.70 – 7.65 (m, 1H), 7.54 – 7.49 (m, 4H), 7.42 – 7.38 (m, 1H), 4.09 – 4.04 (m, 2H), 2.31 – 2.16 (m, 1H), 2.05 – 1.92 (m, 1H), 1.78 – 1.64 (m, 3H), 1.57 – 1.42 (m, 2H), 1.21 (s, 12H). ^{13}C NMR (126 MHz, $CDCl_3$) δ 175.2, 155.9, 155.3, 140.5, 133.4, 131.0, 130.6, 128.7, 128.3, 127.3 (q,

J = 276.8 Hz), 125.8, 124.6, 124.2, 83.5, 72.4, 35.2 (q, J = 27.9 Hz), 29.1, 27.3, 24.7, 24.6. The signal of the α-B-carbon was not observed. ^{19}F NMR (376 MHz, $CDCl_3$) δ -64.97 (t, J = 11.2 Hz). ^{11}B NMR (128 MHz, $CDCl_3$) δ 34.68. IR (neat) ν (cm^{-1}): 3062.0, 2978.1, 2935.3, 2868.2, 1638.2, 1559.9, 1488.7, 1373.5, 1328.8, 1256.1, 1198.3, 1135.0, 1071.6, 963.5, 900.2, 862.9, 758.5, 691.4, 650.4, 577.7; HRMS (ESI): calcd for $C_{27}H_{31}BF_3O_5^+$ $[M + H]^+$ 503.2211; found 503.2210.

4,4,5,5-tetramethyl-2-(4,4,4-trifluoro-1-(4-methoxyphenyl)butan-2-yl)-1,3,2-dioxaborolane (5o)

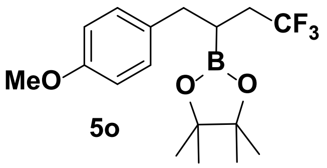
5o

Following the general procedure B, **5o** was synthesized from the corresponding alkene (29.6 mg) and purified by silica gel chromatography (EA/PE = 1/100); 29.3 mg (43%); colorless liquid. ^{1}H NMR (400 MHz, $CDCl_3$) δ 7.13 – 7.09 (m, 2H), 6.83 – 6.80 (m, 2H), 3.78 (s, 3H), 2.78 – 2.72 (m, 1H), 2.65 – 2.59 (m, 1H), 2.30 – 2.02 (m, 2H), 1.67 – 1.59 (m, 1H), 1.16 (d, J = 15.9 Hz, 12H). ^{13}C NMR (101 MHz, $CDCl_3$) δ 158.1, 132.2, 129.8, 127.4 (q, J = 277.0 Hz), 113.7, 83.6, 55.3, 35.4, 34.4 (q, J = 28.0 Hz), 24.7, 24.6. The signal of the α-B-carbon was not observed. ^{19}F NMR (376 MHz, $CDCl_3$) δ -64.75 (t, J = 11.2 Hz). ^{11}B NMR (128 MHz, $CDCl_3$) δ 34.05. IR (neat) ν (cm^{-1}): 2980.0, 2933.4, 2857.0, 1612.1, 1511.4, 1371.7, 1328.8, 1246.8, 1129.4, 1079.1, 1038.1, 965.4, 862.9, 834.9, 669.1, 520.0; HRMS (ESI): calcd for $C_{17}H_{25}BF_3O_3^+$ $[M + H]^+$ 345.1843; found 345.1841.

4,4,5,5-tetramethyl-2-(1,1,1-trifluoro-5-phenylpentan-3-yl)-1,3,2-dioxaborolane (5p)

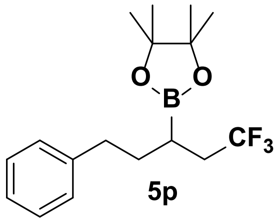
5p

Following the general procedure B, **5p** was synthesized from the corresponding alkene (26.4 mg) and purified by silica gel chromatography (EA/PE = 1/50); 31.2 mg (48%); colorless liquid. ^{1}H NMR (400 MHz, $CDCl_3$) δ 7.29 – 7.25 (m, 2H), 7.20 – 7.16 (m, 3H), 2.70 – 2.57 (m, 2H), 2.40 – 2.26 (m, 1H), 2.20 – 2.08 (m, 1H), 1.87 – 1.68 (m, 2H), 1.41 – 1.33 (m, 1H), 1.27 (s, 12H). ^{13}C NMR (101 MHz, $CDCl_3$) δ 142.0,

128.4, 128.4, 127.4 (q, *J* = 277.0 Hz), 125.9, 83.6, 35.1 (q, *J* = 28.0 Hz), 35.0, 32.9, 24.8, 24.7. The signal of the α-B-carbon was not observed. ^{19}F NMR (377 MHz, $CDCl_3$) δ -64.88 (t, *J* = 11.2 Hz). ^{11}B NMR (128 MHz, $CDCl_3$) δ 33.85. IR (neat) ν (cm^{-1}): 3065.7, 3028.5, 2983.7, 2931.6, 2864.5, 1604.6, 1496.5, 1459.3, 1371.7, 1325.1, 1252.4, 1129.4, 1069.7, 965.4, 862.9, 738.0, 698.9, 590.8; HRMS (ESI): calcd for $C_{17}H_{24}BF_3O_2Na^+$ $[M + Na]^+$ 351.1714; found 351.1717.

(3-(4,4,5,5-tetramethyl-1,3,2-dioxaborolan-2-yl)-4-(trifluoromethyl)cyclopentyl) methyl 2-chlorobenzoate (5q)

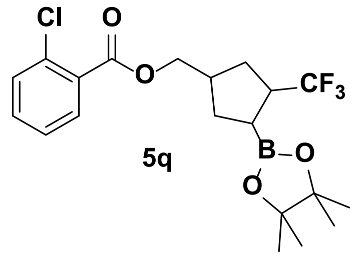

5q

Following the general procedure B, **5q** was synthesized from the corresponding alkene (47.3 mg) and purified by silica gel chromatography (EA/PE = 1/10); 39.1 mg (45%); colorless liquid. dr = 3:1; ^{1}H NMR (400 MHz, $CDCl_3$) δ 7.83 – 7.80 (m, 1H), 7.47 – 7.40 (m, 2H), 7.34 – 7.30 (m, 1H), 4.29 – 4.23 (m, 2H), 2.91 – 2.78 (m, 1H), 2.56 – 2.46 (m, 1H), 2.21 – 1.99 (m, 2H), 1.90 – 1.68 (m, 1H), 1.61 – 1.35 (m, 2H), 1.24 (s, 12H). ^{13}C NMR (126 MHz, $CDCl_3$) δ 165.7, 133.7, 133.6*, 132.5, 132.5*, 131.5, 131.4, 131.1, 131.1, 130.2*, 130.1, 128.4 (q, *J* = 277.5 Hz), 128.1* (q, *J* = 277.5 Hz), 126.6, 83.7*, 83.6, 68.2, 68.1*, 45.6* (q, *J* = 27.2 Hz), 44.8 (q, *J* = 27.0 Hz), 39.9, 38.8*, 32.9, 31.3*, 30.7* (q, *J* = 2.2 Hz), 30.2 (q, *J* = 2.1 Hz), 24.7, 24.5. The signal of the α-B-carbon was not observed. ^{19}F NMR (377 MHz, $CDCl_3$) δ -70.84 (d, *J* = 9.1 Hz), -71.18 (d, *J* = 9.6 Hz). ^{11}B NMR (128 MHz, $CDCl_3$) δ 34.29. IR (neat) ν (cm^{-1}): 2980.0, 1727.6, 1595.3, 1369.8, 1250.5, 1127.5, 1047.4, 972.8, 889.0, 849.8, 752.9, 674.6, 579.6; HRMS (ESI): calcd for $C_{20}H_{26}BClF_3O_4^+$ $[M + H]^+$ 433.1559; found 433.1556.

(8R,9S,10R,13S,14S)-10,13-dimethyl-3-oxo-6,7,8,9,10,11,12,13,14,15,16,17-dodeca hydro-3H-cyclopenta[*a*]phenanthren-17-yl-12,12,12-trifluoro-10-(4,4,5,5-tetrame thyl-1,3,2-dioxaborolan-2-yl)dodecanoate (5r)

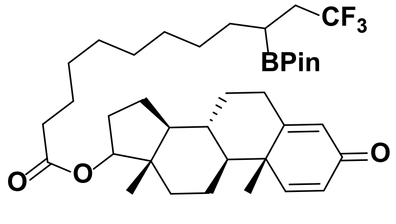

5r

Following the general procedure B, **5r** was synthesized from the corresponding alkene (90.5 mg)

and purified by silica gel chromatography (EA/PE = 1/3); 43.2 mg (33%); colorless liquid. ^{1}H NMR (400 MHz, $CDCl_3$) δ 7.05 (d, *J* = 10.2 Hz, 1H), 6.24 (dd, *J* = 10.1, 1.9 Hz, 1H), 6.08 (t, *J* = 1.4 Hz, 1H), 4.59 (t, *J* = 8.0 Hz, 1H), 2.52 – 1.93 (m, 9H), 1.82 – 1.38 (m, 13H), 1.29 (t, *J* = 12.4 Hz, 10H), 1.24 (s, 12H), 1.20 – 1.01 (m, 5H), 0.87 (s, 3H). ^{13}C NMR (126 MHz, $CDCl_3$) δ 186.4, 173.8, 169.0, 155.8, 127.5, 127.5 (q, *J* = 277.0 Hz), 123.9, 83.5, 82.0, 52.2, 49.9, 43.5, 42.8, 36.5, 35.3, 35.2 (q, *J* = 27.8 Hz), 34.5, 33.1, 32.7, 30.8, 29.5, 29.3, 29.2, 29.1, 28.5, 27.5, 25.0, 24.7, 24.6, 23.7, 22.3, 18.7, 12.1. ^{19}F NMR (377 MHz, $CDCl_3$) δ -65.04 (t, *J* = 11.2 Hz). The signal of the *α*-B-carbon was not observed. ^{19}F NMR (377 MHz, $CDCl_3$) δ -65.04 (t, *J* = 11.2 Hz). ^{11}B NMR (128 MHz, $CDCl_3$) δ 34.42. IR (neat) ν (cm^{-1}): 2974.4, 2927.8, 2855.1, 1731.3, 1662.4, 1623.3, 1448.1, 1373.5, 1326.9, 1258.0, 1136.8, 1075.3, 1017.6, 887.1, 862.9; HRMS (ESI): calcd for $C_{37}H_{57}BF_3O_5^+$ $[M + H]^+$ 649.4246; found 649.4247.

(3S,5R,10S,13R,14R,17R)-4,4,10,13,14-pentamethyl-17-((R)-6-methylheptan-2-yl)-2,3,4,5,6,7,10,11,12,13,14,15,16,17-tetradecahydro-1*H*-cyclopenta[*a*]phenanthren-3-yl-6,6,6-trifluoro-4-(4,4,5,5-tetramethyl-1,3,2-dioxaborolan-2-yl)hexanoate (5s)

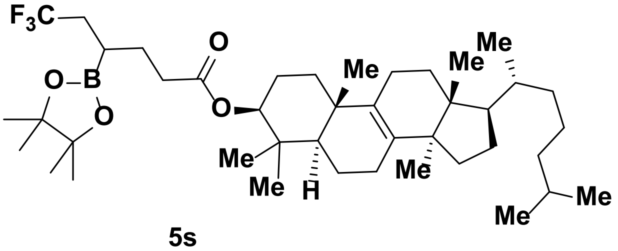

5s

Following the general procedure B, **5s** was synthesized from the corresponding alkene (101.8 mg) and purified by silica gel chromatography (EA/PE = 1/30); 38.1 mg (27%); colorless liquid. dr = 6.9:1; ^{1}H NMR (400 MHz, $CDCl_3$,) δ 4.54 – 4.48 (m, 1H), 2.40 – 2.26 (m, 3H), 2.17 – 1.86 (m, 7H), 1.83 – 1.65 (m, 7H), 1.60 – 1.43 (m, 5H), 1.41 – 1.29 (m, 7H), 1.25 (s, 12H), 1.20 – 1.08 (m, 5H), 1.00 (s, 3H), 1.00 – 0.83 (m, 17H), 0.69 – 0.56 (m, 3H). ^{13}C NMR (101 MHz, $CDCl_3$) δ 172.9, 134.5, 134.2, 127.3 (q, *J* = 275.8 Hz), 83.7, 80.9, 50.5, 49.8, 44.4, 39.5, 37.8, 36.9, 36.5, 35.2, 35.0 (q, *J* = 28.1 Hz), 33.7, 31.0, 30.8, 29.7, 28.2, 28.0, 27.9, 26.4, 26.1, 24.7, 24.7, 24.3, 24.1, 24.1, 22.8, 22.5, 21.0, 19.2, 18.7, 18.1, 16.6, 16.6, 15.7. The signal of the *α*-B-carbon was not observed. ^{19}F NMR (377 MHz, $CDCl_3$, asterisk denotes minor diastereomers

peaks) δ -64.84* (t, J = 11.2 Hz), -64.87 (t, J = 11.2 Hz). ^{11}B NMR (128 MHz, $CDCl_3$) δ 34.25. IR (neat) ν (cm^{-1}): 2929.7, 2871.9, 1731.3, 1459.3, 1371.7, 1328.8, 1258.0, 1135.0, 1067.9, 967.2, 862.9, 734.3, 669.1; HRMS (ESI): calcd for $C_{42}H_{71}BF_3O_4^+$ [M + H]$^+$ 707.5392; found 707.5388.

(R)-2,5,7,8-tetramethyl-2-((4R,8R)-4,8,12-trimethyltridecyl)chroman-6-yl 6,6,6-trifluoro-4-(4,4,5,5-tetramethyl-1,3,2-dioxaborolan-2-yl)hexanoate (5t)

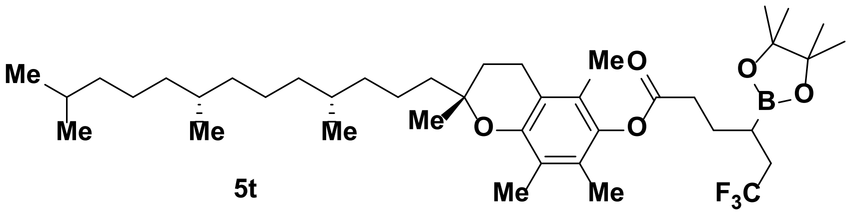

5t

Following the general procedure B, **5t** was synthesized from the corresponding alkene (120.6 mg) and purified by silica gel chromatography (EA/PE = 1/30); 137.1 mg (48%); colorless liquid. ^{1}H NMR (400 MHz, $CDCl_3$) δ 2.68 – 2.56 (m, 4H), 2.41 – 2.30 (m, 1H), 2.21 – 2.12 (m, 1H), 2.08 (s, 3H), 2.00 (s, 3H), 2.00 – 1.93 (m, 5H), 1.83 – 1.72 (m, 2H), 1.56 – 1.49 (m, 3H), 1.46 – 1.33 (m, 5H), 1.28 – 1.20 (m, 22H), 1.16 – 1.03 (m, 7H), 0.87 – 0.84 (m, 12H). ^{13}C NMR (101 MHz, $CDCl_3$) δ 171.6, 149.4, 140.4, 127.2 (q, J = 277.1 Hz), 126.6, 124.8, 123.0, 117.3, 83.8, 75.0, 39.4, 37.4, 37.4, 37.4, 37.3, 35.0 (q, J = 28.1 Hz), 32.9, 32.8, 32.9, 27.9, 25.9, 24.8, 24.7, 24.7, 24.4, 22.7, 22.6, 21.0, 20.6, 19.7, 19.6, 12.9, 12.1, 11.8. The signal of the α-B-carbon was not observed. ^{19}F NMR (377 MHz, $CDCl_3$) δ -64.80 (t, J = 11.1 Hz). ^{11}B NMR (128 MHz, $CDCl_3$) δ 34.92. IR (neat) ν (cm^{-1}): 2927.8, 2868.2, 1753.7, 1459.3, 1373.5, 1330.7, 1258.0, 1135.0, 1066.0, 965.4, 862.9, 669.1; HRMS (ESI): calcd for $C_{41}H_{68}BF_3O_5Na^+$ [M + Na]$^+$ 731.5004; found 731.5002.

13-methyl-17-oxo-7,8,9,11,12,13,14,15,16,17-decahydro-6*H*-cyclopenta[*a*]phenanthren-3-yl 6,6,6-trifluoro-4-(4,4,5,5-tetramethyl-1,3,2-dioxaborolan-2-yl) hexanoate (5u)

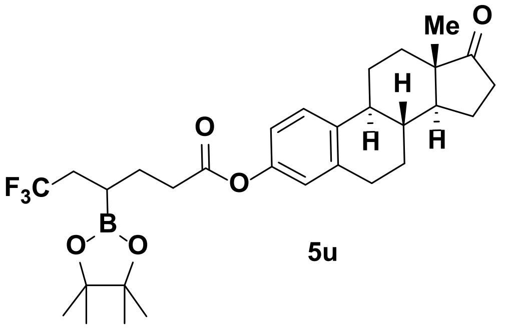

5u

Following the general procedure B, **5u** was synthesized from the corresponding alkene (70.4 mg) and purified by silica gel

chromatography (EA/PE = 1/5); 36.5 mg (33%); colorless liquid. ^{1}H NMR (400 MHz, $CDCl_3$) δ 7.29 – 7.26 (m, 1H), 6.86 – 6.80 (m, 2H), 2.91 – 2.89 (m, 2H), 2.63 – 1.89 (m, 12H), 1.67 – 1.36 (m, 8H), 1.26 (s, 12H), 0.91 (s, 3H). ^{13}C NMR (101 MHz, $CDCl_3$) δ 220.8, 171.9, 148.5, 138.0, 137.3, 127.3 (q, J = 277.1 Hz), 126.3, 121.5, 118.7, 83.8, 50.4, 47.9, 44.1, 38.0, 35.8, 35.1 (q, J = 28.1 Hz), 33.3, 31.5, 29.4, 26.3, 25.7, 24.8, 24.7, 21.6, 13.8. The signal of the α-B-carbon was not observed. ^{19}F NMR (377 MHz, $CDCl_3$) δ -64.76 (t, J = 11.1 Hz). ^{11}B NMR (128 MHz, $CDCl_3$) δ 34.60. IR (neat) ν (cm^{-1}): 2978.1, 2927.8, 2860.7, 1736.9, 1492.8, 1457.4, 1373.5, 1330.7, 1258.0, 1220.7, 1129.4, 1082.8, 862.9, 670.9, 579.6; HRMS (ESI): calcd for $C_{30}H_{41}BF_3O_5^+$ [M + H]$^+$ 549.2994; found 549.2990.

7,7,8,8,9,9,10,10,10-nonafluoro-5-(4,4,5,5-tetramethyl-1,3,2-dioxaborolan-2-yl)decyl 4-methylbenzoate (5v)

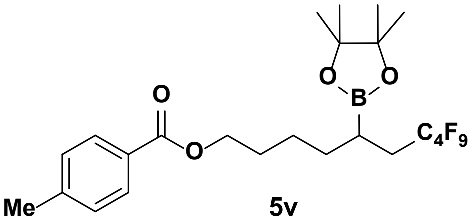

5v

Following the general procedure B, **5v** was synthesized from the corresponding alkene (43.7 mg) and purified by silica gel chromatography (EA/PE = 1/20); 71.8 mg (64%); colorless liquid. ^{1}H NMR (400 MHz, $CDCl_3$) δ 7.86 – 7.83 (m, 2H), 7.16 – 7.13 (m, 2H), 4.22 (t, J = 6.5 Hz, 2H), 2.33 (s, 3H), 2.31 – 2.19 (m, 1H), 2.05 – 1.90 (m, 1H), 1.74 – 1.67 (m, 2H), 1.57 – 1.30 (m, 5H), 1.14 (d, J = 2.4 Hz, 12H). ^{13}C NMR (126 MHz, $CDCl_3$) δ 166.7, 143.5, 129.5, 129.0, 127.6, 83.6, 64.6, 32.1 (t, J = 21.9 Hz), 31.1, 28.8, 25.2, 24.7, 24.5, 21.6. ^{19}F NMR (377 MHz, $CDCl_3$) δ -81.06 – -81.12 (m), -112.01 – -113.98 (m), -124.66 (q, J = 9.1 Hz), -125.94 – -126.04 (m). ^{11}B NMR (128 MHz, $CDCl_3$) δ 34.76 (s), 34.76. The signal of the α-B-carbon was not observed. ^{19}F NMR (376 MHz, $CDCl_3$) δ -79.13 – -84.56 (m), -109.81 – -117.27 (m), -124.33 – -125.09 (m), -125.40 – -126.39 (m).^{11}B NMR (128 MHz, $CDCl_3$) δ 34.84. IR (neat) ν (cm^{-1}): 2980.0, 2933.4, 2864.5, 1718.3, 1612.1, 1459.3, 1390.3, 1328.8, 1272.9, 1217.0, 1177.8, 1131.2, 1107.0, 1021.3, 877.8, 846.1, 754.8, 725.0, 691.4, 577.7, 527.4; HRMS (ESI): calcd for $C_{24}H_{31}BF_9O_4^+$ [M + H]$^+$ 565.2166; found 565.2168.

4-((6,6,7,7,8,8,9,9,9-nonafluoro-4-(4,4,5,5-tetramethyl-1,3,2-dioxaborolan-2-yl)nonyl)oxy)-2H-chromen-2-one (5w)

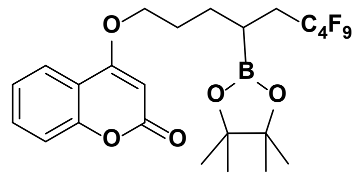
5w

Following the general procedure B, **5w** was synthesized from the corresponding alkene (46.1 mg) and purified by silica gel chromatography (EA/PE = 1/5); 61.2 mg (53%); colorless liquid. ^{1}H NMR (400 MHz, $CDCl_3$) δ 7.83 (dd, *J* = 7.9, 1.5 Hz, 1H), 7.57 – 7.53 (m, 1H), 7.33 – 7.25 (m, 2H), 5.67 (s, 1H), 4.14 (t, *J* = 6.2 Hz, 2H), 2.48 – 2.31 (m, 1H), 2.18 – 1.92 (m, 3H), 1.81 – 1.64 (m, 3H), 1.54 – 1.45 (m, 1H), 1.26 (d, *J* = 1.2 Hz, 12H). ^{13}C NMR (101 MHz, $CDCl_3$) δ 165.6, 162.9, 153.3, 132.4, 123.8, 123.0, 116.8, 115.7, 90.5, 83.8, 69.1, 31.9 (t, *J* = 21.8 Hz), 27.6, 24.7, 24.6. The signal of the α-B-carbon was not observed. ^{19}F NMR (377 MHz, $CDCl_3$) δ -81.01 – -81.07 (m), -112.00 – -113.84 (m), -124.59 (q, *J* = 9.0 Hz), -125.89 – -126.00 (m). ^{11}B NMR (128 MHz, $CDCl_3$) δ 34.87. IR (neat) ν (cm^{-1}): 3076.9, 2980.0, 2935.3, 2864.5, 1723.9, 1627.0, 1571.1, 1459.3, 1373.5, 1326.9, 1217.0, 1133.1, 1075.3, 1015.7, 930.0, 846.1, 725.0, 687.7; HRMS (ESI): calcd for $C_{24}H_{27}BF_9O_5^+$ $[M + H]^+$ 577.1802; found 577.1804.

N-(5,5,6,6,7,7,8,8,8-nonafluoro-3-(4,4,5,5-tetramethyl-1,3,2-dioxaborolan-2-yl)octyl)benzamide (5x)

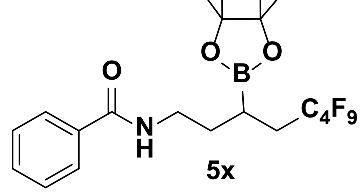
5x

Following the general procedure B, **5x** was synthesized from the corresponding alkene (35.0 mg) and purified by silica gel chromatography (EA/PE = 1/3); 53.1 mg (51%); colorless liquid. ^{1}H NMR (400 MHz, $CDCl_3$) δ 7.76 (d, *J* = 7.6 Hz, 2H), 7.50 – 7.41 (m, 3H), 6.46 (s, 1H), 3.62 – 3.41 (m, 2H), 2.45 – 2.07 (m, 2H), 1.85 – 1.72 (m, 2H), 1.52 – 1.47 (m, 1H), 1.24 (s, 12H). ^{13}C NMR (126 MHz, $CDCl_3$) δ 167.6, 134.6, 131.4, 128.5, 126.9, 84.0, 39.1, 31.9 (t, *J* = 21.8 Hz), 30.7, 24.7, 24.6. The signal of the α-B-carbon was not observed. ^{19}F NMR (377 MHz, $CDCl_3$) δ -81.01 – -81.06 (m), -111.92 – -113.83 (m), -124.60 (q, *J* = 8.9 Hz), -125.91 – -126.07 (m). ^{11}B NMR (128 MHz, $CDCl_3$) δ 34.68. IR (neat) ν (cm^{-1}): 3274.5, 3062.0,

2980.0, 2935.3, 2871.9, 1535.7, 1448.1, 1332.5, 1215.1, 1131.2, 1075.3, 848.0, 695.1, 531.1; HRMS (ESI): calcd for $C_{21}H_{26}BF_9NO_3^+$ $[M + H]^+$ 522.1857; found 522.1859.

(3-(perfluorobutyl)-4-(4,4,5,5-tetramethyl-1,3,2-dioxaborolan-2-yl)cyclopentyl)methyl 2-chlorobenzoate (5y)

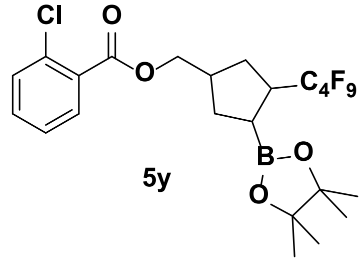

5y

Following the general procedure B, **5y** was synthesized from the corresponding alkene (47.3 mg) and purified by silica gel chromatography (EA/PE = 1/20); 51.4 mg (44%); colorless liquid. dr = 1:1; ^{1}H NMR (400 MHz, $CDCl_3$, mixture of diastereomers) δ 7.83 – 7.80 (m, 1H), 7.47 – 7.27 (m, 3H), 4.30 – 4.26 (m, 2H), 3.04 – 2.91 (m, 1H), 2.56 – 2.46 (m, 1H), 2.17 – 2.05 (m, 2H), 1.85 – 1.56 (m, 2H), 1.43 – 1.35 (m, 1H), 1.23 (d, J = 3.1 Hz, 12H). ^{13}C NMR (126 MHz, $CDCl_3$, asterisk denotes minor diastereomers peaks) δ 165.8, 133.6, 133.6*, 132.5, 131.4, 131.4*, 131.1, 130.2*, 130.1, 126.6, 83.6, 68.2, 68.0*, 43.7* (t, J = 21.5 Hz), 42.9 (t, J = 21.5 Hz), 40.0, 39.0*, 32.5, 30.9*, 30.8*, 29.7, 24.6, 24.4. The signal of the α-B-carbon was not observed. ^{19}F NMR (377 MHz, $CDCl_3$) δ -80.96 – -81.02 (m), -112.47 – -113.30 (m), -119.84 – -121.12 (m), -121.87 – -122.29 (m), -125.17 – -127.09 (m). ^{11}B NMR (128 MHz, $CDCl_3$) δ 34.45. IR (neat) ν (cm^{-1}): 2980.0, 1707.1, 1593.4, 1436.9, 1373.5, 1325.1, 1282.2, 1230.0, 1129.4, 1051.1, 967.2, 885.2, 851.7, 747.3, 685.8, 581.5, 534.9; HRMS (ESI): calcd for $C_{23}H_{26}BClF_9O_4^+$ $[M + H]^+$ 583.1463; found 583.1462.

4,4,5,5-tetramethyl-2-(5,5,6,6,7,7,8,8,8-nonafluoro-1-(3-methoxyphenoxy)octan-3-yl)-1,3,2-dioxaborolane (5z)

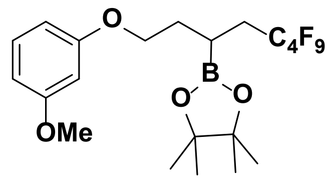

5z

Following the general procedure B, **5z** was synthesized from the corresponding alkene (35.6 mg) and purified by silica gel chromatography (EA/PE = 1/50); 29.6 mg (28%); colorless liquid. ^{1}H NMR (400 MHz, $CDCl_3$) δ 7.17 (t, J = 8.2 Hz, 1H), 6.52 – 6.46 (m, 3H), 4.04 – 3.94 (m, 2H), 3.78 (s, 3H), 2.45 – 2.11 (m, 2H), 2.06 – 1.89 (m, 3H), 1.66 – 1.59 (m, 1H), 1.24 (d, J = 3.7 Hz, 12H). ^{13}C NMR (126

MHz, $CDCl_3$) δ 160.8, 160.1, 129.8, 106.8, 106.4, 101.1, 83.7, 66.8, 55.2, 31.8 (t, J = 21.8 Hz), 30.4, 24.8, 24.6. The signal of the α-B-carbon was not observed. ^{19}F NMR (377 MHz, $CDCl_3$) δ -80.99 – -81.06 (m), -111.89 – -113.94 (m), -124.59 – -124.66 (m), -125.88 – -125.99 (m). ^{11}B NMR (128 MHz, $CDCl_3$) δ 33.53. IR (neat) ν (cm^{-1}): 2981.9, 2935.3, 1593.4, 1492.8, 1382.8, 1332.5, 1217.0, 1131.2, 1045.5, 848.0, 734.3, 687.7, 529.3; HRMS (ESI): calcd for $C_{21}H_{27}BF_9O_4^+$ [M + H]$^+$ 525.1853; found 525.1850.

6,6,7,7,8,8,9,9,9-nonafluoro-4-(4,4,5,5-tetramethyl-1,3,2-dioxaborolan-2-yl)nonyl 4-chlorobenzoate (5aa)

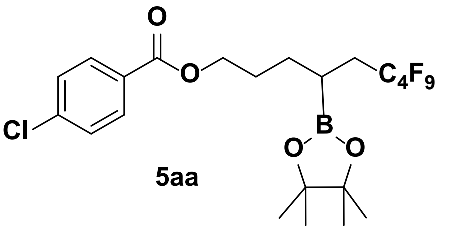

5aa

Following the general procedure B, **5aa** was synthesized from the corresponding alkene (44.9 mg) and purified by silica gel chromatography (EA/PE = 1/20); 77.4 mg (68%); colorless liquid. ^{1}H NMR (400 MHz, $CDCl_3$) δ 7.99 – 7.96 (m, 2H), 7.42 – 7.40 (m, 2H), 4.31 (t, J = 6.3 Hz, 2H), 2.43 – 2.27 (m, 1H), 2.15 – 2.01 (m, 1H), 1.85 – 1.78 (m, 2H), 1.72 – 1.57 (m, 2H), 1.49 – 1.40 (m, 1H), 1.24 (s, 12H). ^{13}C NMR (101 MHz, $CDCl_3$) δ 165.7, 139.3, 130.9, 128.8, 128.7, 83.7, 32.0 (t, J = 21.9 Hz), 27.8, 27.8, 24.7, 24.6. The signal of the α-B-carbon was not observed. ^{19}F NMR (377 MHz, $CDCl_3$) δ -81.03 – -81.08 (m), -112.03 – -113.94 (m), -124.59 – -124.63 (m), -125.94 – -126.02 (m). ^{11}B NMR (128 MHz, $CDCl_3$) δ 34.44. IR (neat) ν (cm^{-1}): 2981.9, 2937.1, 1720.2, 1597.2, 1392.2, 1330.7, 1269.2, 1217.0, 1131.2, 1092.1, 1015.7, 848.0, 760.4, 725.0, 525.8; HRMS (ESI): calcd for $C_{22}H_{26}BClF_9O_4^+$ [M + H]$^+$ 571.1463; found 571.1464.

6,6,7,7,8,8,9,9,10,10,11,11,11-tridecafluoro-4-(4,4,5,5-tetramethyl-1,3,2-dioxaborolan-2-yl)undecyl 4-chlorobenzoate (5bb)

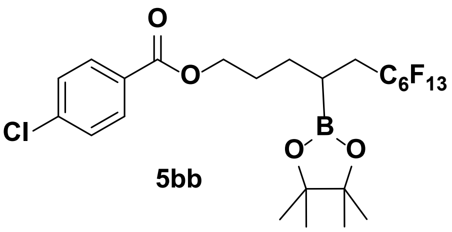

5bb

Following the general procedure B, **5bb** was synthesized from the corresponding alkene (44.9 mg) and purified by silica gel chromatography

(EA/PE = 1/20); 82.1 mg (61%); white solid; mp 37-39 °C. ^{1}H NMR (400 MHz, $CDCl_3$) δ 7.99 – 7.96 (m, 2H), 7.43 – 7.39 (m, 2H), 4.31 (t, *J* = 6.3 Hz, 2H), 2.44 – 2.28 (m, 1H), 2.14 – 2.01 (m, 1H), 1.85 – 1.78 (m, 2H), 1.72 – 1.55 (m, 2H), 1.49 – 1.40 (m, 1H), 1.24 (s, 12H). ^{13}C NMR (126 MHz, $CDCl_3$) δ 165.7, 139.3, 130.9, 128.8, 128.7, 83.7, 65.0, 32.1 (t, *J* = 21.9 Hz), 27.8 (d, *J* = 3.4 Hz), 24.7, 24.6. The signal of the *α*-B-carbon was not observed. ^{19}F NMR (377 MHz, $CDCl_3$) δ -80.78 – -80.84 (m), -111.77 – -113.68 (m), -121.84 (s), -122.87 (s), -123.68 (s), -126.10 – -126.20 (m). ^{11}B NMR (128 MHz, $CDCl_3$) δ 34.52. IR (neat) ν (cm^{-1}): 2980.0, 2937.1, 1722.0, 1597.2, 1489.1, 1392.2, 1330.7, 1269.2, 1235.6, 1142.4, 1015.7, 965.4, 849.8, 760.4, 706.3, 527.4; HRMS (ESI): calcd for $C_{24}H_{26}BClF_{13}O_4^+$ [M + H]$^+$ 671.1400; found 671.1395.

6,6,7,7,8,8,9,9,10,10,11,11,12,12,13,13,13-heptadecafluoro-4-(4,4,5,5-tetramethyl-1,3,2-dioxaborolan-2-yl)tridecyl 4-chlorobenzoate (5cc)

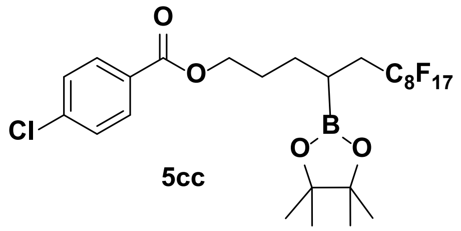

5cc

Following the general procedure B, **5cc** was synthesized from the corresponding alkene (44.9 mg) and purified by silica gel chromatography (EA/PE = 1/20); 91.6 mg (59%); white solid; mp 53-55 °C. ^{1}H NMR (400 MHz, $CDCl_3$) δ 7.99 – 7.96 (m, 2H), 7.43 – 7.39 (m, 2H), 4.31 (t, *J* = 6.3 Hz, 2H), 2.44 – 2.28 (m, 1H), 2.14 – 2.01 (m, 1H), 1.85 – 1.78 (m, 2H), 1.72 – 1.55 (m, 2H), 1.49 – 1.40 (m, 1H), 1.24 (s, 12H). ^{13}C NMR (126 MHz, $CDCl_3$) δ 165.7, 139.3, 130.9, 128.8, 128.7, 83.7, 65.0, 32.1 (t, *J* = 21.9 Hz), 27.8, 27.8, 24.7, 24.6. The signal of the *α*-B-carbon was not observed. ^{19}F NMR (377 MHz, $CDCl_3$) δ -80.78 (t, *J* = 9.9 Hz), -111.72 – -113.66 (m), -121.62 (s), -121.92 (s), -122.72 (s), -123.65 (s), -126.08 – -126.18 (m). ^{11}B NMR (128 MHz, $CDCl_3$) δ 34.61. IR (neat) ν (cm^{-1}): 2976.3, 2952.1, 2858.9, 1710.8, 1593.4, 1371.7, 1328.8, 1278.5, 1196.5, 1142.4, 1090.2, 1013.8, 954.2, 760.4, 654.1, 529.3; HRMS (ESI): calcd for $C_{26}H_{26}BClF_{17}O_4^+$ [M + H]$^+$ 771.1336; found 771.1335.

7-((8-chloro-1,1,2,2,3,3,4,4,5,5,6,6,7,7,8,8-hexadecafluorooctyl)oxy)-6,6,7,7-tetrafluoro-4-(4,4,5,5-tetramethyl-1,3,2-dioxaborolan-2-yl)heptyl 4-chlorobenzoate (5dd)

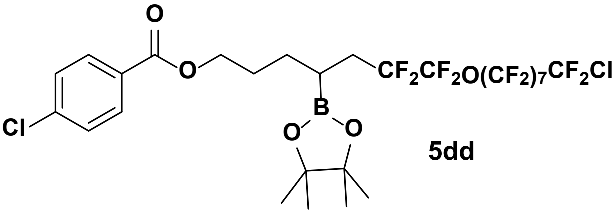

5dd

Following the general procedure B, **5dd** was synthesized from the corresponding alkene (44.9 mg) and purified by silica gel chromatography (EA/PE = 1/20); 81.3 mg (45%); white solid; mp 37-39 °C. ^{1}H NMR (400 MHz, $CDCl_3$) δ 7.99 – 7.95 (m, 2H), 7.42 – 7.39 (m, 2H), 4.31 (t, J = 6.4 Hz, 2H), 2.36 – 2.21 (m, 1H), 2.08 – 1.93 (m, 1H), 1.84 – 1.75 (m, 2H), 1.71 – 1.53 (m, 2H), 1.46 – 1.39 (m, 1H), 1.23 (s, 12H). ^{13}C NMR (126 MHz, $CDCl_3$) δ 165.7, 139.3, 130.9, 128.8, 128.7, 83.7, 65.0, 31.5 (t, J = 21.6 Hz), 27.8, 27.7, 24.7, 24.6. The signal of the α-B-carbon was not observed. ^{19}F NMR (377 MHz, $CDCl_3$) δ -68.07 (t, J = 13.5 Hz), -83.19 – -83.31 (m), -87.47 – -88.51 (m), -116.00 – -117.89 (m), -120.11 (s), -121.18 (s), -121.78 (s), -122.09 (s), -125.51 (s). ^{11}B NMR (128 MHz, $CDCl_3$) δ 34.98. The signal of the α-B-carbon was not observed. IR (neat) ν (cm^{-1}): 2981.9, 2935.3, 1723.9, 1597.2, 1373.5, 1271.0, 1209.5, 1142.4, 1015.7, 760.4; HRMS (ESI): calcd for $C_{28}H_{26}BCl_2F_{20}O_5^+$ [M + H]$^+$ 903.0926; found 903.0928.

6,6,6-trifluoro-4-hydroxyhexyl 4-chlorobenzoate (6)

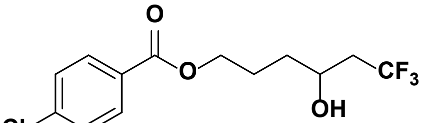

6

Following the procedure VI-3, **6** was synthesized from **5a** (84.1 mg) and purified by silica gel chromatography (EA/PE = 1/10); 38.0 mg (62%); colorless liquid. ^{1}H NMR (400 MHz, $CDCl_3$) δ 7.98 – 7.95 (m, 1H), 7.43 – 7.40 (m, 1H), 4.37 (t, J = 6.5 Hz, 1H), 4.13 – 4.07 (m, 1H), 2.38 – 2.23 (m, 1H), 2.04 – 1.81 (m, 2H), 1.71 – 1.65 (m, 1H). ^{13}C NMR (101 MHz, $CDCl_3$) δ 165.8, 139.5, 130.9, 128.7, 128.6, 126.3 (q, J = 277.1 Hz), 65.7 (q, J = 2.9 Hz), 64.8, 41.3 (q, J = 26.5 Hz), 33.5, 24.7. ^{19}F NMR (377 MHz, $CDCl_3$) δ -63.50 (t, J = 10.9 Hz). IR (neat) ν (cm^{-1}): 3449.7, 2957.6, 1718.3, 1595.3, 1429.4, 1246.8, 1144.3, 1058.6, 1015.7, 930.0, 851.7, 766.0,

698.9, 622.5, 542.3; HRMS (ESI): calcd for $C_{13}H_{14}ClF_3O_3Na^+$ $[M + Na]^+$ 333.0476; found 333.0475.

4,6,6,6-tetrafluorohexyl 4-chlorobenzoate (7)

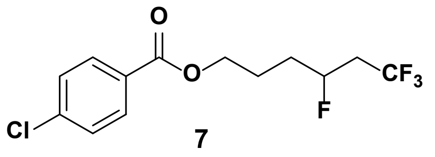

Following the general procedure VII-4, **7** was synthesized from **5a** (84.1 mg) and purified by silica gel chromatography (EA/PE = 1/20); 47.6 mg (76%); colorless liquid. ^{1}H NMR (400 MHz, $CDCl_3$) δ 7.98 – 7.95 (m, 2H), 7.44 – 7.40 (m, 2H), 4.98 – 4.79 (m, 1H), 4.41 – 4.32 (m, 2H), 2.64 – 2.26 (m, 2H), 2.05 – 1.73 (m, 4H). ^{13}C NMR (101 MHz, $CDCl_3$) δ 165.6, 139.5, 130.9, 128.7, 128.5, 125.3 (qd, J = 276.8, 3.9 Hz), 87.2 (dq, J = 172.9, 3.2 Hz), 64.3, 39.3 (qd, J = 28.6, 23.0 Hz), 31.6 (d, J = 21.1 Hz), 24.1 (d, J = 3.9 Hz). ^{19}F NMR (377 MHz, $CDCl_3$) δ -64.06– -64.14 (m), -182.59 – -182.97 (m). IR (neat) ν (cm^{-1}): 2957.6, 1718.3, 1595.3, 1489.1, 1403.3, 1269.2, 1090.2, 1015.7, 849.8, 758.5, 685.8, 592.6, 523.7; HRMS (ESI): calcd for $C_{13}H_{13}ClF_4O_2Na^+$ $[M + Na]^+$ 335.0432; found 335.0429.

(3-(2,2,2-trifluoroethyl)pent-4-en-1-yl)benzene (8)

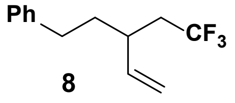

Following the procedure VII-5, **8** was synthesized from **5p** (65.6 mg) and purified by silica gel chromatography (PE); 32.1 mg (70%); colorless liquid. ^{1}H NMR (400 MHz, $CDCl_3$) δ 7.30 – 7.25 (m, 2H), 7.20 – 7.15 (m, 3H), 5.69 – 5.60 (m, 1H), 5.14 – 5.08 (m, 2H), 2.71 – 2.64 (m, 1H), 2.57 – 2.39 (m, 2H), 2.23 – 2.10 (m, 2H), 1.84 – 1.75 (m, 1H), 1.68 – 1.59 (m, 1H). ^{13}C NMR (101 MHz, $CDCl_3$) δ 141.7, 139.9, 128.4, 128.3, 126.6 (q, J = 277.5 Hz), 125.9, 116.3, 39.0 (q, J = 27.0 Hz), 37.8 (q, J = 2.4 Hz), 36.2, 33.0. ^{19}F NMR (377 MHz, $CDCl_3$) δ -63.04 (t, J = 11.0 Hz). IR (neat) ν (cm^{-1}): 3360.2, 3084.4, 3028.5, 2920.4, 2851.4, 1632.6, 1496.5, 1455.5, 1377.3, 1252.4, 1123.8, 1051.1, 918.8, 836.8, 747.3, 698.9, 654.1; HRMS (EI): calcd for $C_{13}H_{15}F_3$ [M] 228.1126; found 228.1130.

2-(1,1,1-trifluoro-5-phenylpentan-3-yl)thiophene (9)

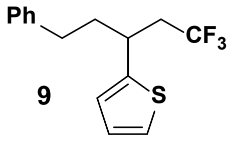

Following the procedure VII-6, **9** was synthesized from **5p** (65.6 mg) and purified by silica gel chromatography (EA/PE = 1/30); 45.2 mg (79%); colorless liquid. ^{1}H NMR (400 MHz, $CDCl_3$) δ 7.29 – 7.11 (m, 5H), 6.97 – 6.95 (m, 1H), 6.87 – 6.86 (m, 1H), 3.34 – 3.27 (m, 1H), 2.60

– 2.41 (m, 4H), 2.17 – 2.08 (m, 1H), 2.00 – 1.90 (m, 1H). ^{13}C NMR (101 MHz, $CDCl_3$) δ 146.6, 141.2, 128.4, 128.4, 126.7, 126.3 (q, J = 277.6 Hz), 126.0, 124.8, 123.7, 41.7 (q, J = 27.3 Hz), 39.0, 34.9 (q, J = 2.6 Hz), 33.1. ^{19}F NMR (377 MHz, $CDCl_3$) δ -63.77 (t, J = 10.7 Hz). IR (neat) ν (cm^{-1}): 3065.7, 3028.5, 2927.8, 2857.0, 1602.8, 1496.5, 1453.7, 1377.3, 1254.2, 1099.6, 1038.1, 849.8, 749.2, 695.1; HRMS (ESI): calcd for $C_{15}H_{16}F_3S^+$ $[M + H]^+$ 285.0919; found 285.0914.

4,4,5,5-tetramethyl-2-(4,4,4-trifluoro-2-phenethylbutyl)-1,3,2-dioxaborolane (10)

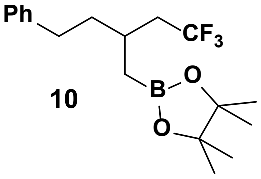

10

Following the procedure VII-7, **10** was synthesized from **5p** (65.6 mg) and purified by silica gel chromatography (EA/PE = 1/50); 41.7 mg (61%); colorless liquid. ^{1}H NMR (400 MHz, $CDCl_3$) δ 7.29 – 7.26 (m, 2H), 7.20 – 7.16 (m, 3H), 2.65 – 2.58 (m, 2H), 2.23 – 2.06 (m, 3H), 1.78 – 1.62 (m, 2H), 1.24 (s, 12H), 1.00 (d, J = 6.3 Hz, 2H). ^{13}C NMR (101 MHz, $CDCl_3$) δ 142.2, 128.4, 128.3, 127.3 (q, J = 285.3 Hz), 125.8, 83.2, 39.3 (q, J = 26.7 Hz), 38.1, 32.9, 28.8 (q, J = 2.2 Hz), 24.8. The signal of the α-B-carbon was not observed. ^{19}F NMR (377 MHz, $CDCl_3$) δ -63.00 (t, J = 10.7 Hz). ^{11}B NMR (128 MHz, $CDCl_3$) δ 33.96. IR (neat) ν (cm^{-1}): 3025.8, 2980.0, 2931.6, 2864.5, 1604.6, 1373.5, 1321.3, 1254.2, 1131.2, 967.2, 846.1, 747.3, 698.9, 665.3; HRMS (ESI): calcd for $C_{18}H_{26}BF_3O_2Na^+$ $[M + Na]^+$ 365.1870; found 365.1872.

(*E*)-(1-bromo-3,3,3-trifluoroprop-1-en-1-yl)benzene (11)

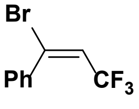

11

Following the procedure VII-8, **11** was synthesized from **3a** (59.6 mg) and purified by silica gel chromatography (PE); 26.5 mg (53%); colorless liquid. ^{1}H NMR (400 MHz, $CDCl_3$) δ 7.43 – 7.36 (m, 5H, mixture of isomer), 6.56 – 6.38 (m, 1H, mixture of isomer). ^{13}C NMR (101 MHz, $CDCl_3$, asterisk denotes minor isomer peaks) δ 137.1, 135.8 (q, J = 6.5 Hz), 130.7*, 130.0, 128.6*, 128.2, 128.0 (q, J = 1.7 Hz), 127.8*, 121.8 (q, J = 35.3 Hz), 121.5 (q, J = 272.1 Hz). ^{19}F NMR (377 MHz, $CDCl_3$, 10:1 isomer ratio, asterisk denotes minor isomer peaks) δ -57.24 (d, J = 7.4 Hz), -59.42* (d, J = 7.3 Hz). IR (neat) ν (cm^{-1}): 3073.2, 2927.8, 1653.1, 1347.4, 1263.6, 1177.8, 1120.1, 818.2, 767.8, 725.0, 691.4, 643.0, 590.8. HRMS (EI): calcd for $C_9H_6BrF_3$ [M] 249.9605; found 249.9603.

(*E*)-3-(3,3,3-trifluoro-1-phenylprop-1-en-1-yl)pyridine (12)

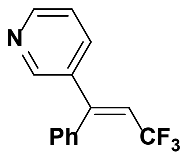
12

Following the procedure VII-9, **12** was synthesized from **3a** (59.6 mg) and purified by silica gel chromatography (EA/PE = 1/20); 45.1 mg (90%); colorless liquid. ^{1}H NMR (400 MHz, $CDCl_3$) δ 8.60 – 8.56 (m, 2H), 7.52 – 7.49 (m, 1H), 7.43 – 7.40 (m, 3H), 7.28 – 7.22 (m, 3H), 6.17 (q, *J* = 8.0 Hz, 1H). ^{13}C NMR (101 MHz, $CDCl_3$) δ 150.3, 149.6 (q, *J* = 5.6 Hz), 148.6, 136.1, 135.9, 135.3, 128.9, 128.9 (q, *J* = 1.8 Hz), 128.3, 123.2, 122.6 (q, *J* = 270.9 Hz), 116.8 (q, *J* = 34.2 Hz). ^{19}F NMR (377 MHz, $CDCl_3$) δ -55.96 (d, *J* = 8.1 Hz). IR (neat) ν (cm^{-1}): 3056.4, 3034.1, 1640.0, 1414.5, 1362.3, 1269.2, 1110.7, 1025.0, 849.8, 777.1, 741.7, 697.0, 633.6, 583.3; HRMS (ESI): calcd for $C_{14}H_{11}F_3N^+$ [M + H]$^+$ 250.0838; found 250.0840.

(*E*)-(5,5,5-trifluoro-2-(trifluoromethyl)penta-1,3-dien-3-yl)benzene (13)

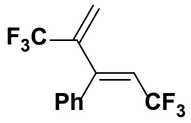
13

Following the procedure VII-9, **13** was synthesized from **3a** (59.6 mg) and purified by silica gel chromatography (PE); 38.6mg (73%); colorless liquid. ^{1}H NMR (400 MHz, $CDCl_3$) δ 7.41 – 7.38 (m, 3H), 7.21 – 7.18 (m, 2H), 6.12 (q, *J* = 7.9 Hz, 1H), 6.08 (s, 1H), 5.39 (q, *J* = 1.8 Hz, 1H). ^{13}C NMR (101 MHz, $CDCl_3$) δ 144.6 (q, *J* = 5.5 Hz), 138.6 (q, *J* = 29.8 Hz), 134.5, 128.8, 128.7 (q, *J* = 1.5 Hz), 128.2, 127.2 (q, *J* = 5.4 Hz), 122.5 (q, *J* = 274.8 Hz), 122.4 (q, *J* = 271.6 Hz), 119.3 (qq, *J* = 34, 2.1 Hz). ^{19}F NMR (377 MHz, $CDCl_3$) δ -56.29 (d, *J* = 7.8 Hz), -63.8. IR (neat) ν (cm^{-1}): 2922.2, 2851.4, 1723.9, 1645.6, 1410.8, 1341.8, 1282.2, 1213.2, 1172.2, 1123.8, 959.8, 881.5, 697.0, 657.9, 598.2. HRMS (EI): calcd for $C_{12}H_8F_6$ [M] 266.0530; found 266.0538.

(*E*)-triisopropyl(5,5,5-trifluoro-3-phenylpent-3-en-1-yn-1-yl)silane (14)

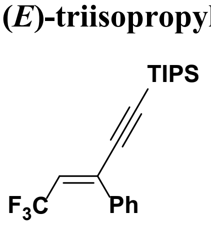
14

Following the procedure VII-9, **14** was synthesized from **3a** (59.6 mg) and purified by silica gel chromatography (PE); 68.2 mg (96%); colorless liquid. ^{1}H NMR (400 MHz, $CDCl_3$) δ 7.46 – 7.45 (m, 2H), 7.37 – 7.35 (m, 3H), 6.14 (q, *J* = 8.7 Hz, 1H), 1.08 (s, 21H). ^{13}C NMR (101 MHz, $CDCl_3$) δ 135.2, 135.0 (q, *J* = 6.2 Hz), 129.1, 128.3 (q, *J* = 2.0 Hz), 128.1, 122.9 (q, *J* = 34.8 Hz), 122.4 (q, *J* = 270.6 Hz), 105.8, 97.4, 18.6, 11.2. ^{19}F NMR (377 MHz, $CDCl_3$) δ -56.21 (d, *J* = 8.7 Hz). IR (neat) ν (cm^{-1}): 2944.6, 2866.3, 2119.0, 1619.5, 1463.0, 1358.6, 1269.2, 1224.4, 1120.1, 1062.3, 997.1, 877.8, 773.4, 752.9,

695.1, 607.6; HRMS (ESI): calcd for $C_{20}H_{27}F_3SiNa^+$ $[M + Na]^+$ 375.1726; found 375.1724.

(*E*)-5-(3,3,3-trifluoro-1-phenylprop-1-en-1-yl)benzo[*d*][1,3]dioxole (15)

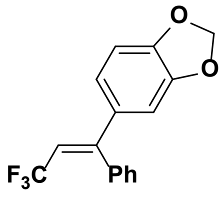

15

Following the procedure VII-9, **15** was synthesized from **3a** (59.6 mg) and purified by silica gel chromatography (EA/PE = 1/20); 51.3 mg (88%); colorless liquid. ^{1}H NMR (400 MHz, $CDCl_3$) δ 7.38 – 7.37 (m, 3H), 7.24-7.21 (m, 2H), 6.75 – 6.71 (m, 3H), 6.03 (q, *J* = 8.3 Hz, 1H), 5.96 (s, 2H). ^{13}C NMR (101 MHz, $CDCl_3$) δ 151.9 (q, *J* = 5.7 Hz), 148.7, 147.9, 137.3, 134.3, 129.1 (q, *J* = 1.7 Hz), 128.5, 128.0, 123.2 (q, *J* = 270.5 Hz), 122.6, 114.1 (q, *J* = 33.8 Hz), 108.1, 108.0, 101.5. ^{19}F NMR (377 MHz, $CDCl_3$) δ -55.26 (d, *J* = 8.3 Hz). IR (neat) ν (cm^{-1}): 3060.1, 2980.0, 2780.6, 1638.2, 1485.3, 1444.3, 1373.5, 1224.4, 1092.1, 1038.1, 805.1, 777.1, 700.7, 643.0, 551.6; HRMS (ESI): calcd for $C_{16}H_{12}F_3O_2^+$ $[M + H]^+$ 293.0784; found 293.0782.

(8R,9S,13S,14S)-8,13-dimethyl-3-((*E*)-3,3,3-trifluoro-1-phenylprop-1-en-1-yl)-7,8, 9,11,12,13,15,16-octahydro-6*H*-cyclopenta[*a*]phenanthren-17(14*H*)-one (16)

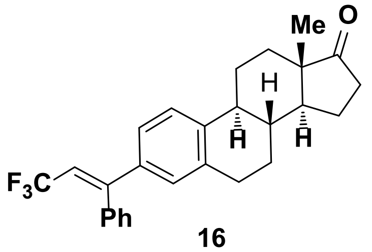

16

Following the procedure VII-9, **16** was synthesized from **3a** (59.6 mg) and purified by silica gel chromatography (EA/PE = 1/20); 76.4 mg (90%); colorless liquid. ^{1}H NMR (400 MHz, $CDCl_3$) δ 7.40 – 7.36 (m, 3H), 7.25 – 7.22 (m, 3H), 7.03 – 6.99 (m, 2H), 6.10 (q, *J* = 8.3 Hz, 1H), 2.88 – 2.85 (m, 3H), 2.54 – 2.27 (m, 4H), 2.19 – 1.94 (m, 6H), 1.68 – 1.43 (m, 3H). ^{13}C NMR (101 MHz, $CDCl_3$) δ 220.6, 152.2 (q, *J* = 5.5 Hz), 141.4, 137.5, 137.3, 136.7, 129.0 (q, *J* = 1.7 Hz), 128.4, 128.3, 127.9, 125.5, 125.4, 123.1 (q, *J* = 270.6 Hz), 114.7 (q, *J* = 33.7 Hz), 50.4, 47.9, 44.4, 37.9, 35.8, 31.5, 29.3, 26.3, 25.6, 21.5, 13.8. ^{19}F NMR (377 MHz, $CDCl_3$) δ -55.35 (d, *J* = 8.3 Hz). IR (neat) ν (cm^{-1}): 3058.3, 2931.6, 2860.7, 1735.1, 1638.2, 1492.8, 1358.6, 1269.2, 1107.0, 1008.2, 907.6, 821.9, 779.0, 728.7, 700.7, 644.8. HRMS (EI): calcd for $C_{28}H_{29}F_3O$ [M] 424.2014; found 424.2017.

X-ray crystallography data for 1a

Supplementary Table 11 Crystal data and structure refinement

Identification code	**1a**
Empirical formula	$C_{16}H_{12}F_6N_2O_5S_2$
Formula weight	490.40
Temperature/K	196
Crystal system	monoclinic
Space group	$P2_1/c$
a/Å	14.4215(14)
b/Å	9.3331(8)
c/Å	13.9228(14)
α/	90
β/	90.476(4)
γ/	90
Volume/Å^3	1873.9(3)
Z	4
ρ_{calc}g/cm^3	1.738
μ/mm^{-1}	0.377
F(000)	992.0
Crystal size/mm^3	0.21 × 0.17 × 0.13
Radiation	MoKα (λ = 0.71073)
2Θ range for data collection/	5.978 to 55.046
Index ranges	-18≤h≤18, -12≤k≤12, -18≤l≤13
Reflections collected	15370
Independent reflections	4237 [R_{int}= 0.0587, R_{sigma}= 0.0535]
Data/restraints/parameters	4237/0/281
Goodness-of-fit on F^2	1.069
Final R indexes [I>=2σ (I)]	R_1 = 0.0426, wR_2 = 0.1196
Final R indexes [all data]	R_1 = 0.0454, wR_2 = 0.1226
Largest diff. peak/hole / e Å^{-3}	0.53/-0.41

Crystal structure determination of 1a

Crystal Data for $C_{16}H_{12}F_6N_2O_5S_2$ (M =490.40 g/mol): monoclinic, space group $P2_1/c$ (no. 14), a = 14.4215(14) Å, b = 9.3331(8) Å, c = 13.9228(14) Å, β = 90.476(4), V = 1873.9(3) Å^3, Z = 4, T = 196 K, μ(MoKα) = 0.377 mm^{-1}, $Dcalc$ = 1.738 g/cm^3, 15370 reflections measured (5.978 ≤ 2Θ ≤ 55.046), 4237 unique (R_{int} = 0.0587, R_{sigma} = 0.0535) which were used in all calculations. The final R_1 was 0.0426 (I > 2σ(I)) and wR_2 was 0.1226 (all data).

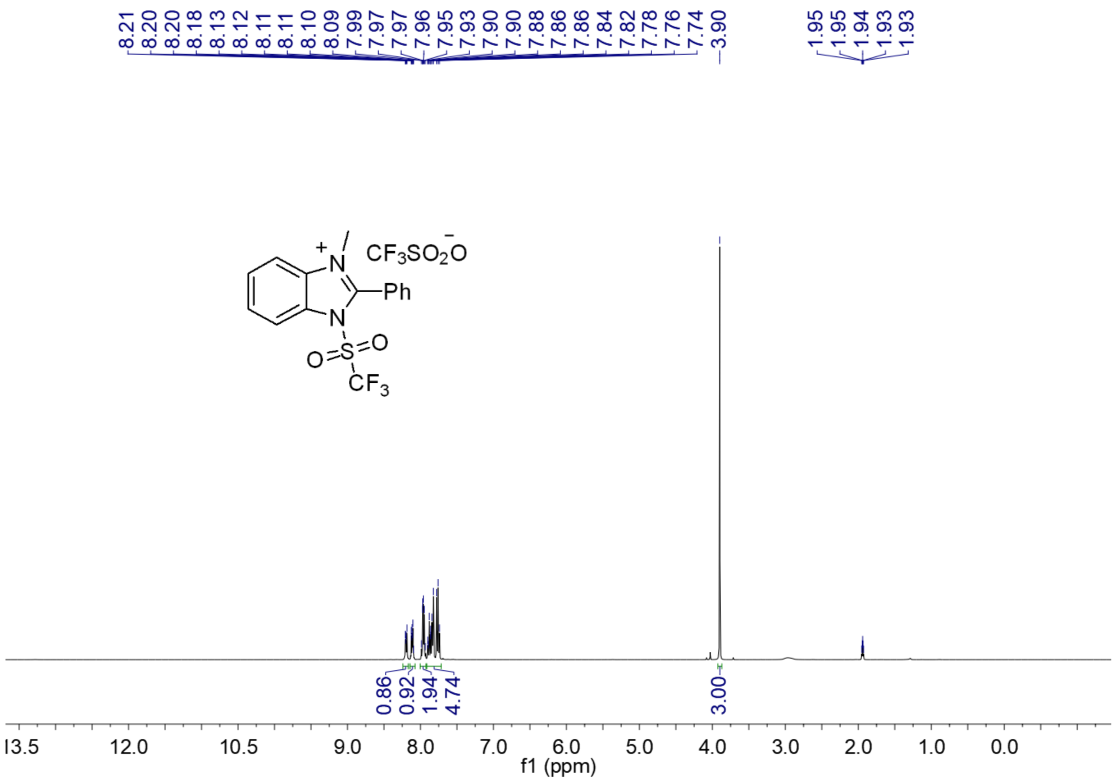

Supplementary Figure 20. ^{1}H NMR Spectra of product **1a**

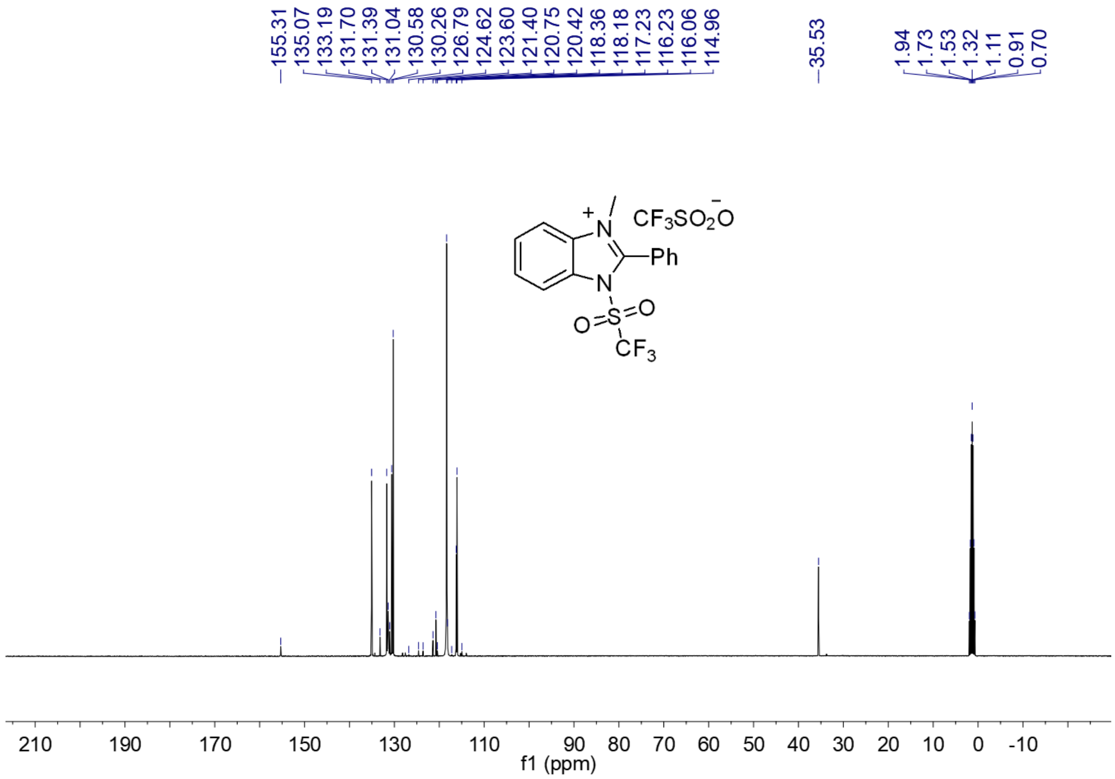

Supplementary Figure 21. ^{13}C NMR Spectra of product **1a**

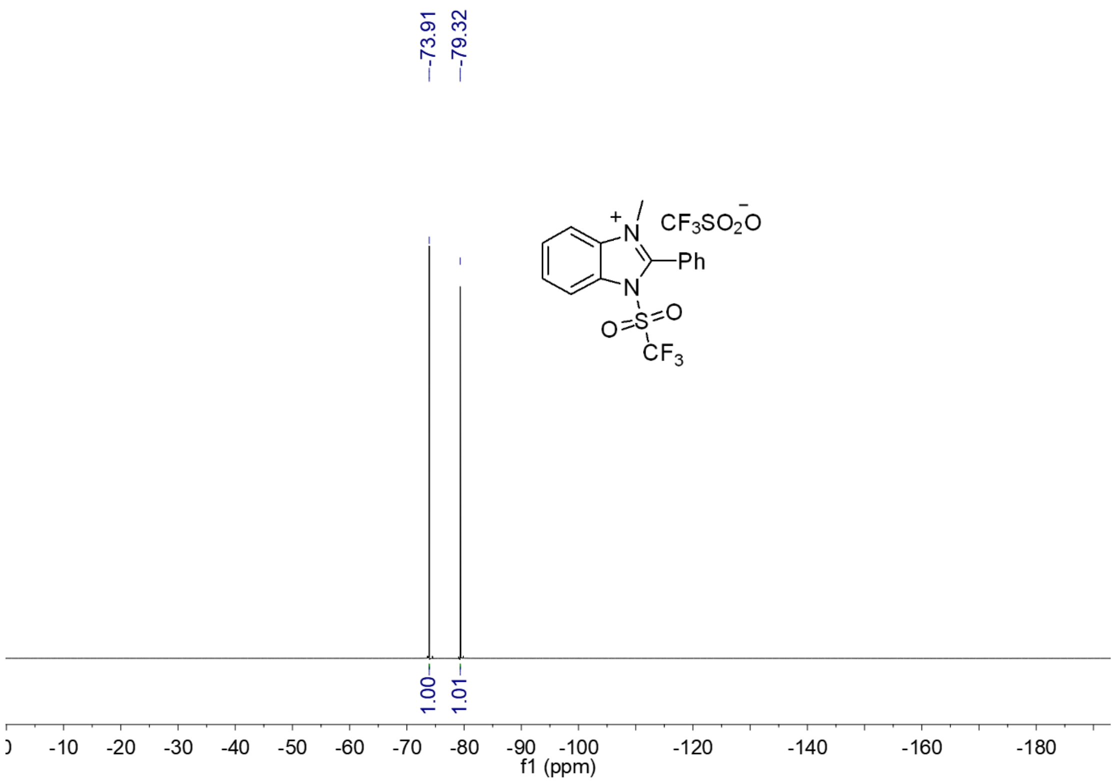

Supplementary Figure 22. ^{19}F NMR Spectra of product **1a**

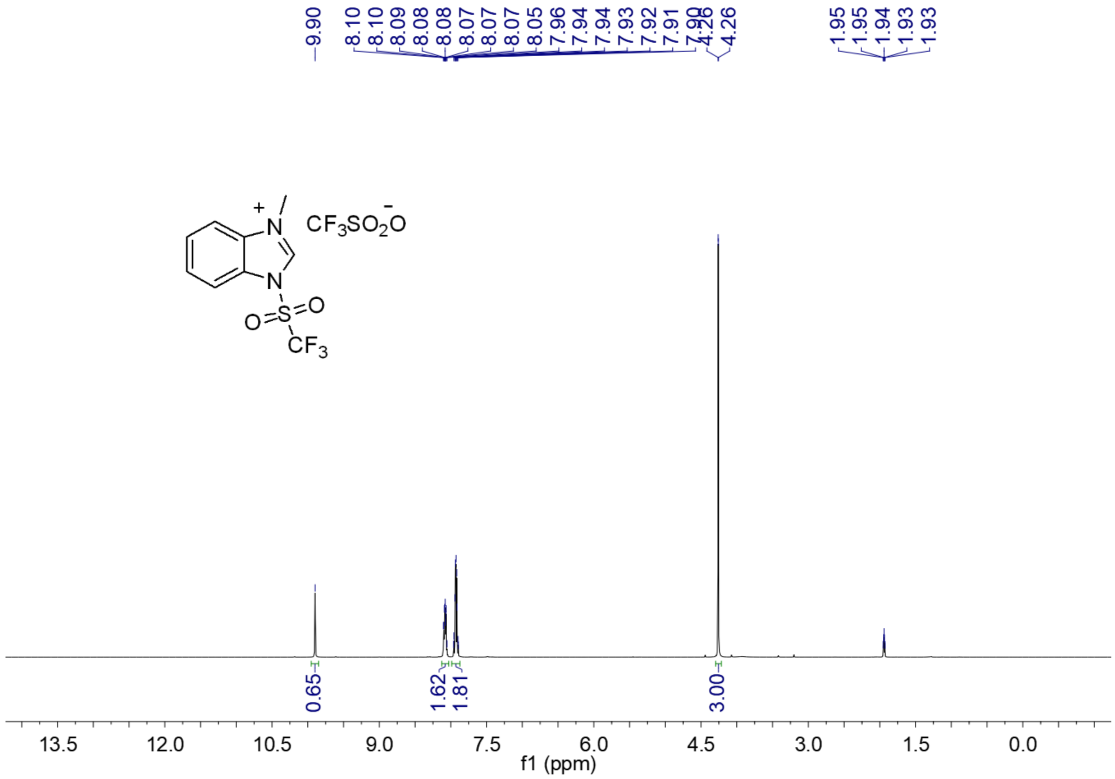

Supplementary Figure 23. ^{1}H NMR Spectra of product **1b**

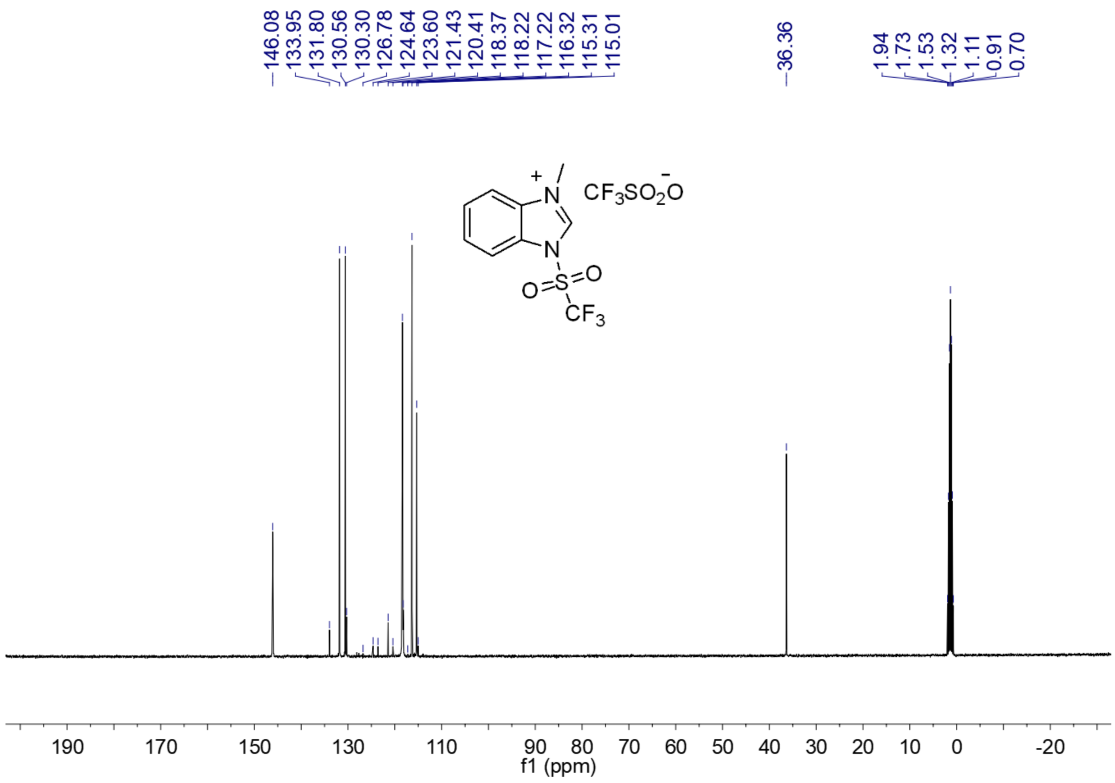

Supplementary Figure 24. ^{13}C NMR Spectra of product **1b**

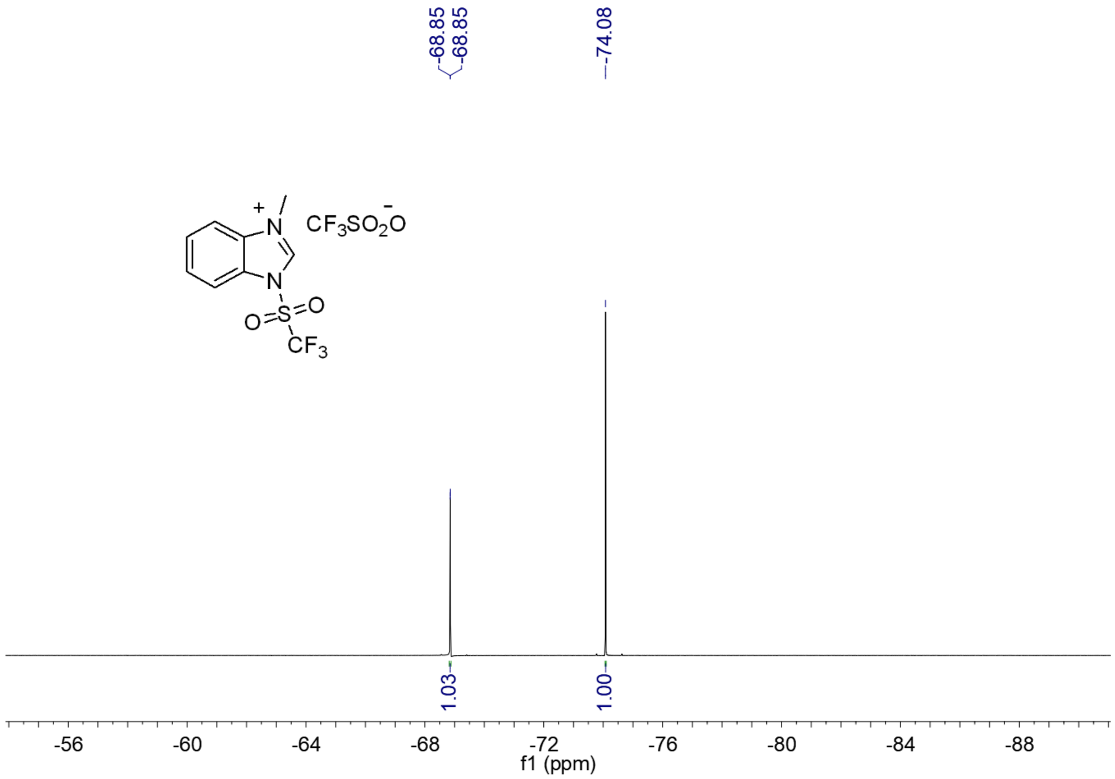

Supplementary Figure 25. ^{19}F NMR Spectra of product **1b**

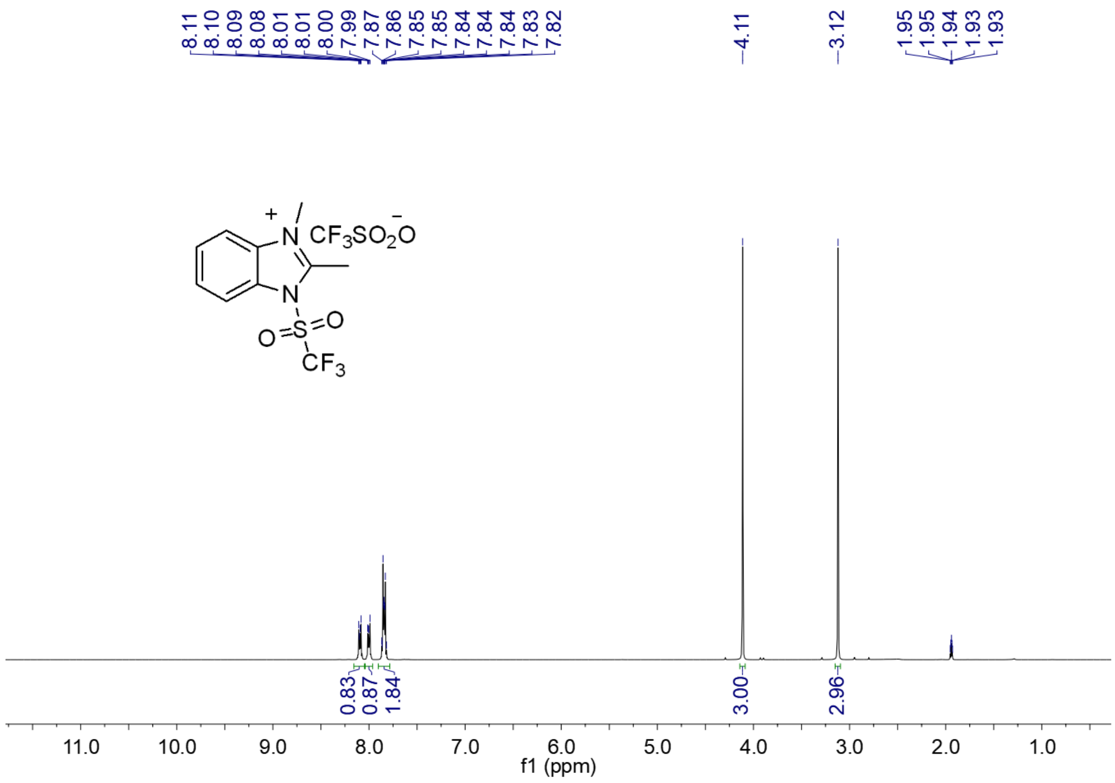

Supplementary Figure 26. ^{1}H NMR Spectra of product **1c**

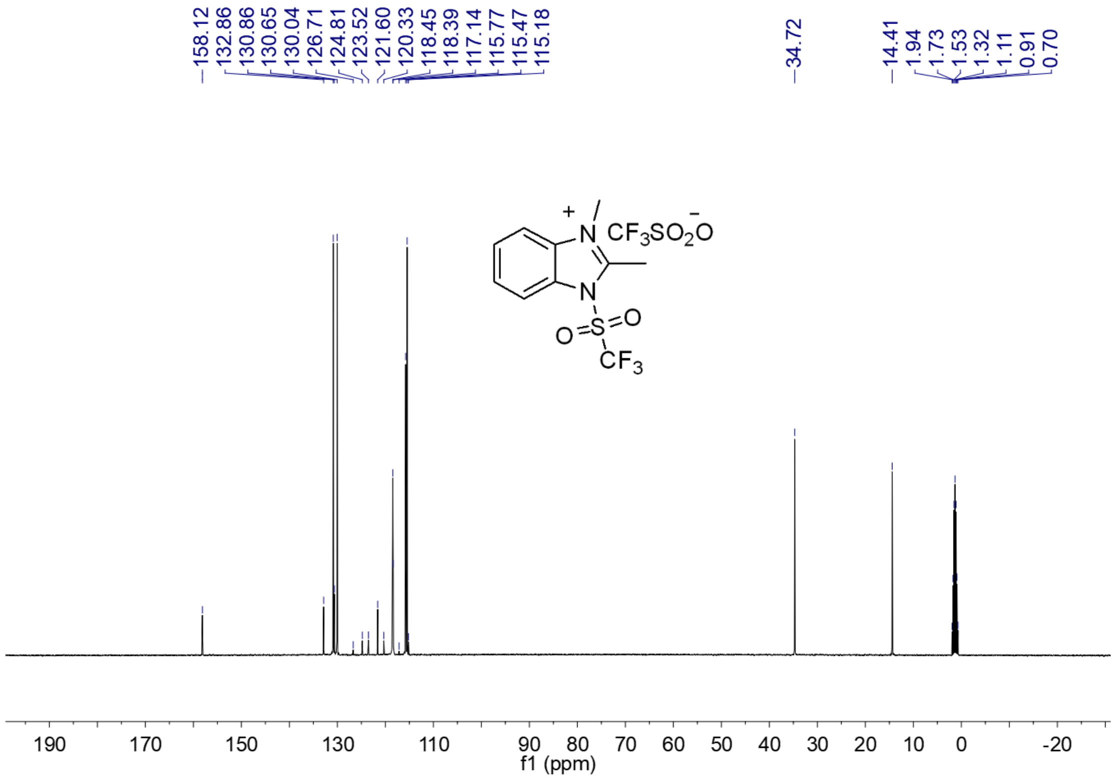

Supplementary Figure 27. ^{13}C NMR Spectra of product **1c**

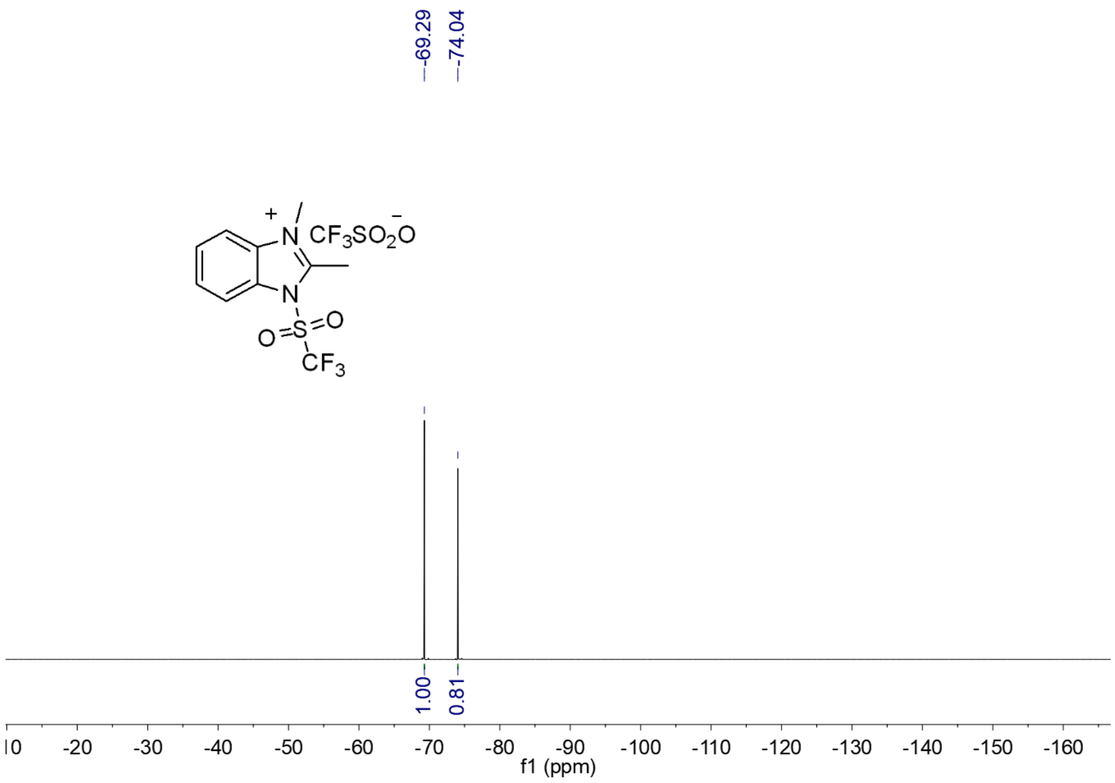

Supplementary Figure 28. ^{19}F NMR Spectra of product **1c**

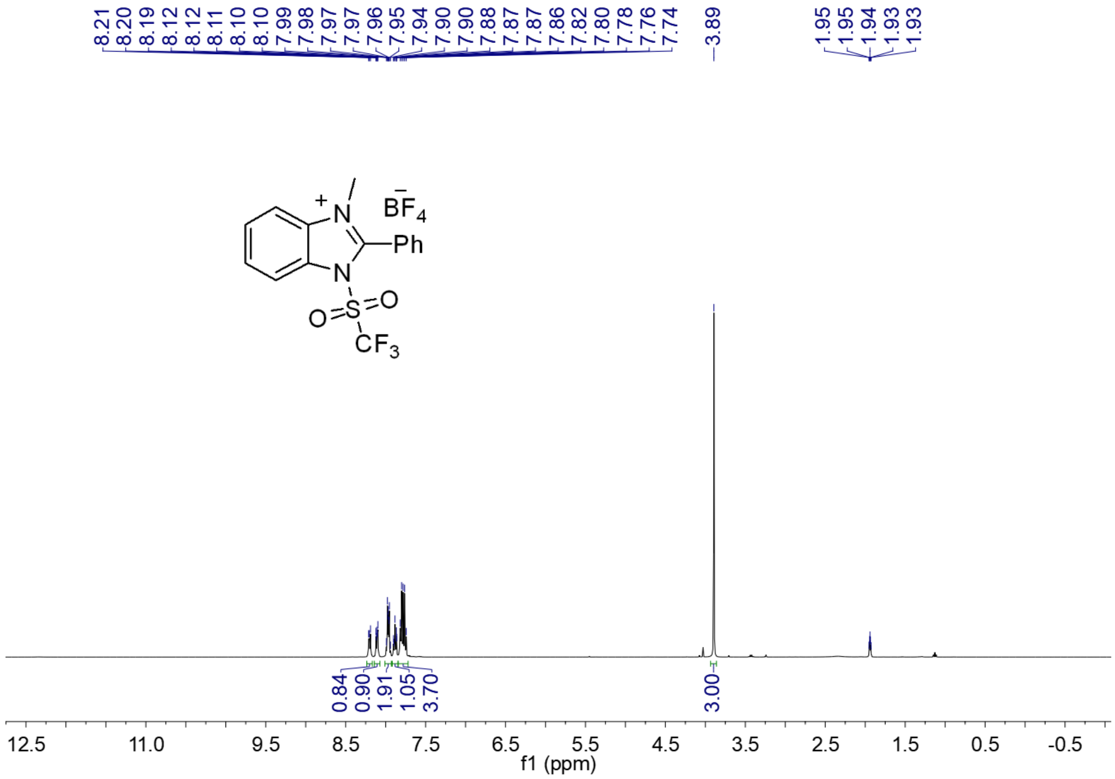

Supplementary Figure 29. ^{1}H NMR Spectra of product **1d**

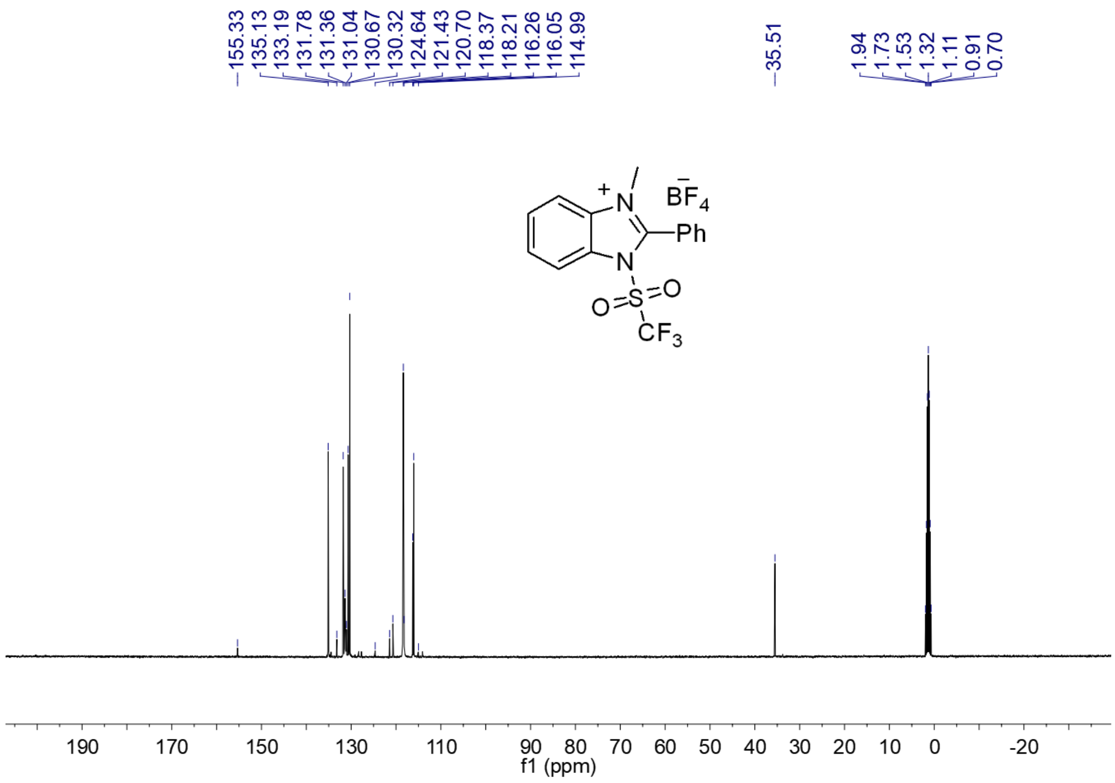

Supplementary Figure 30. ^{13}C NMR Spectra of product **1d**

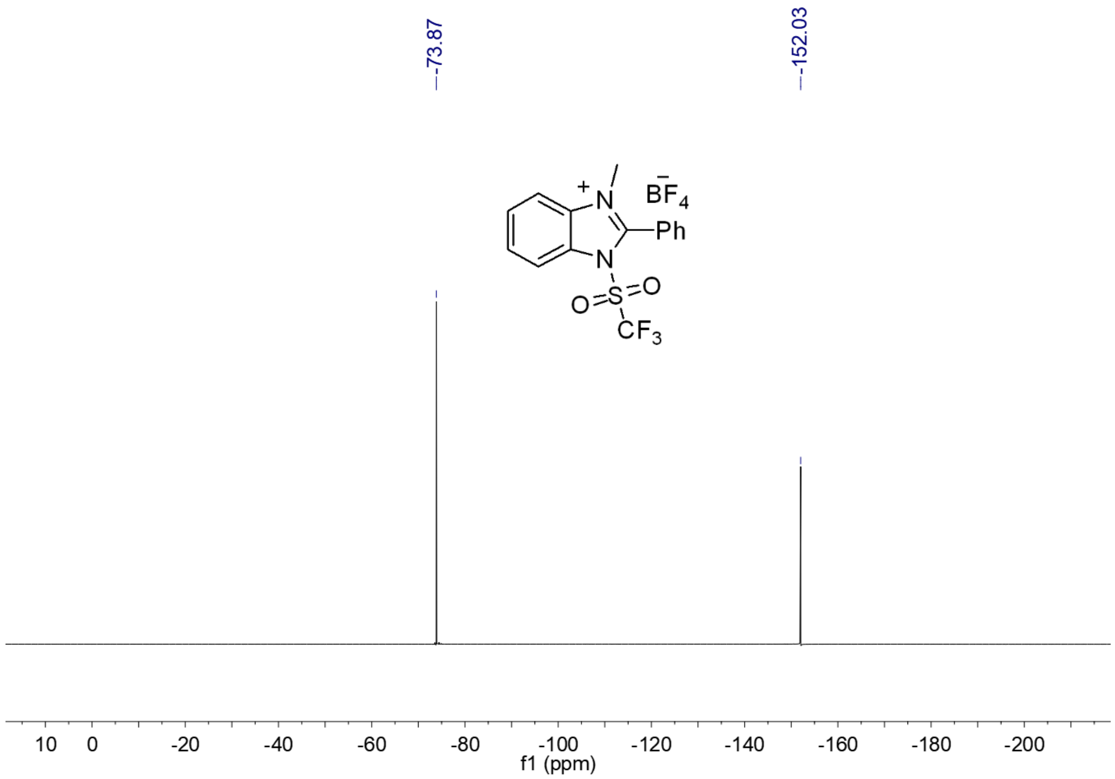

Supplementary Figure 31. ^{19}F NMR Spectra of product **1d**

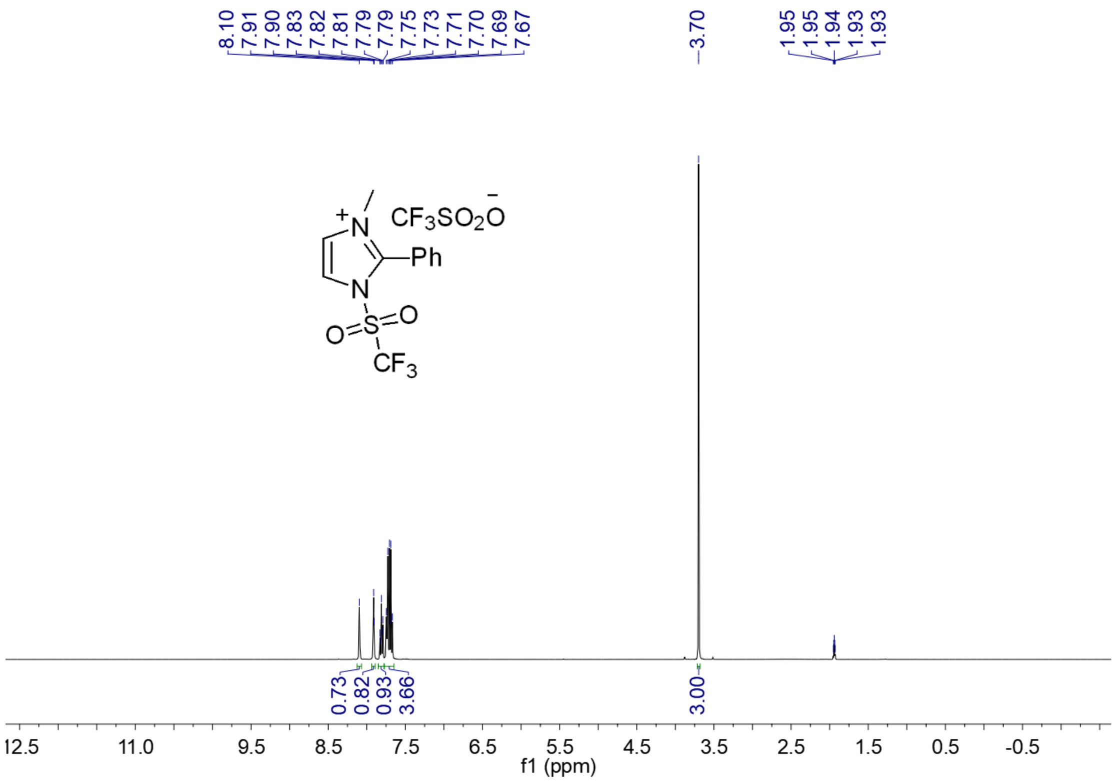

Supplementary Figure 32. ^{1}H NMR Spectra of product **1e**

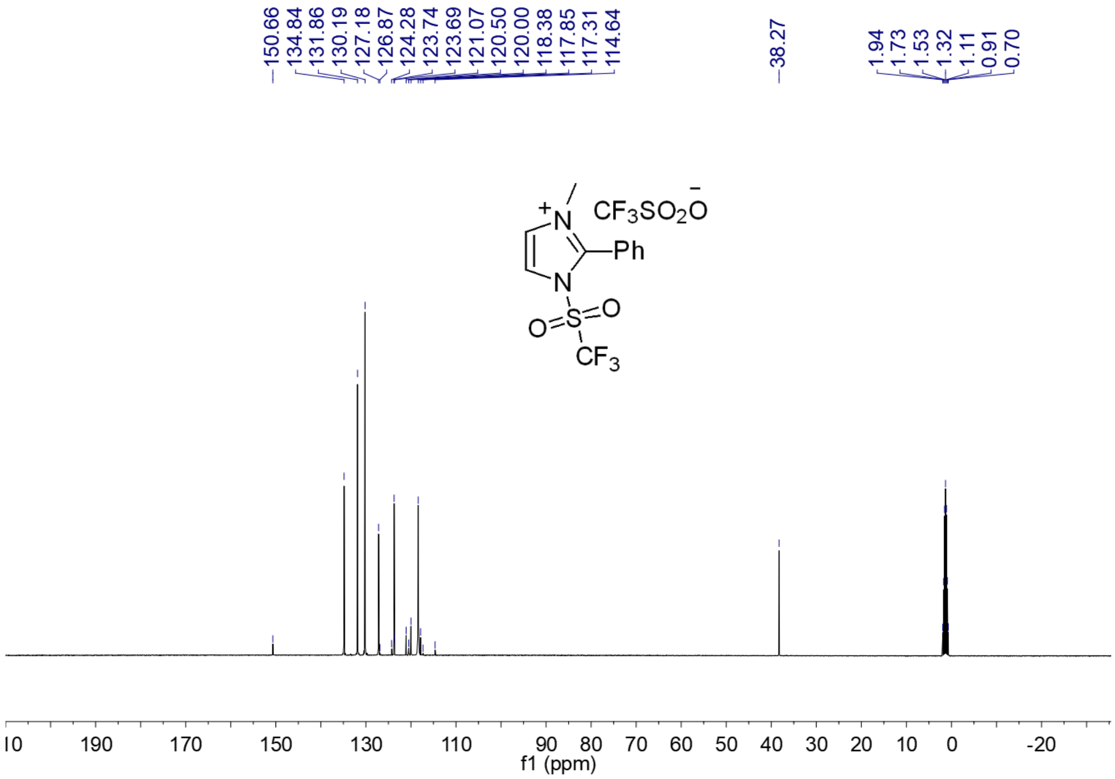

Supplementary Figure 33. ^{13}C NMR Spectra of product **1e**

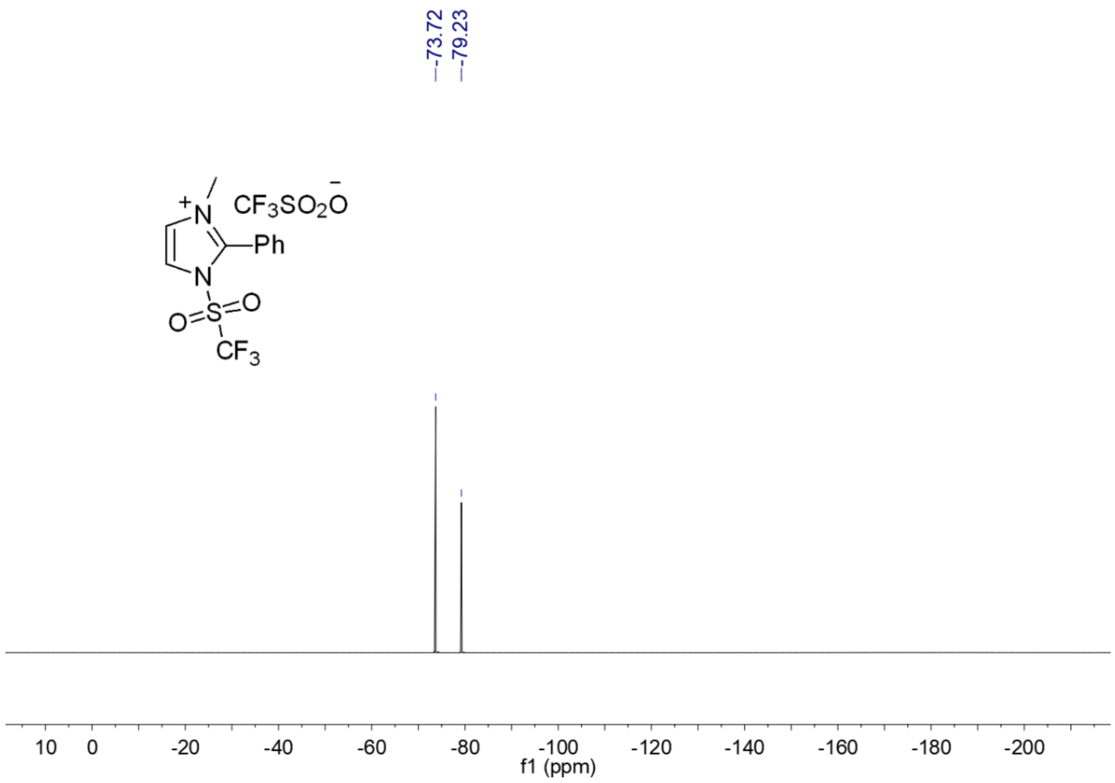

Supplementary Figure 34. ^{19}F NMR Spectra of product **1e**

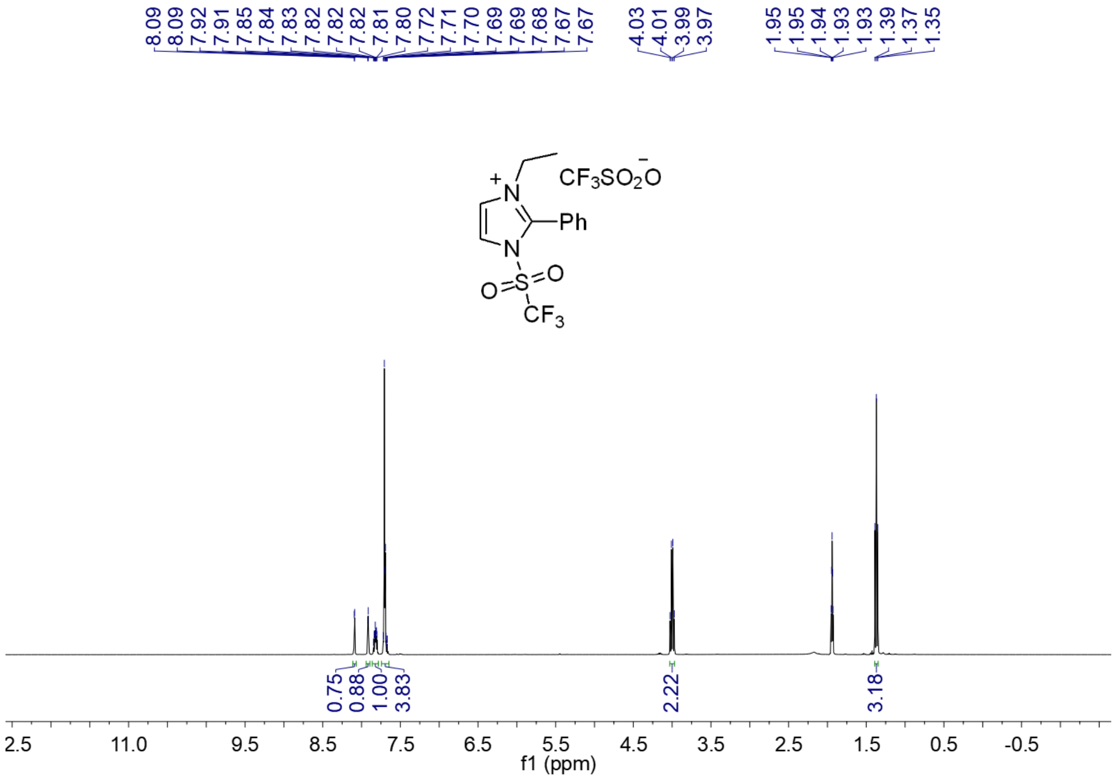

Supplementary Figure 35. ^{1}H NMR Spectra of product **1f**

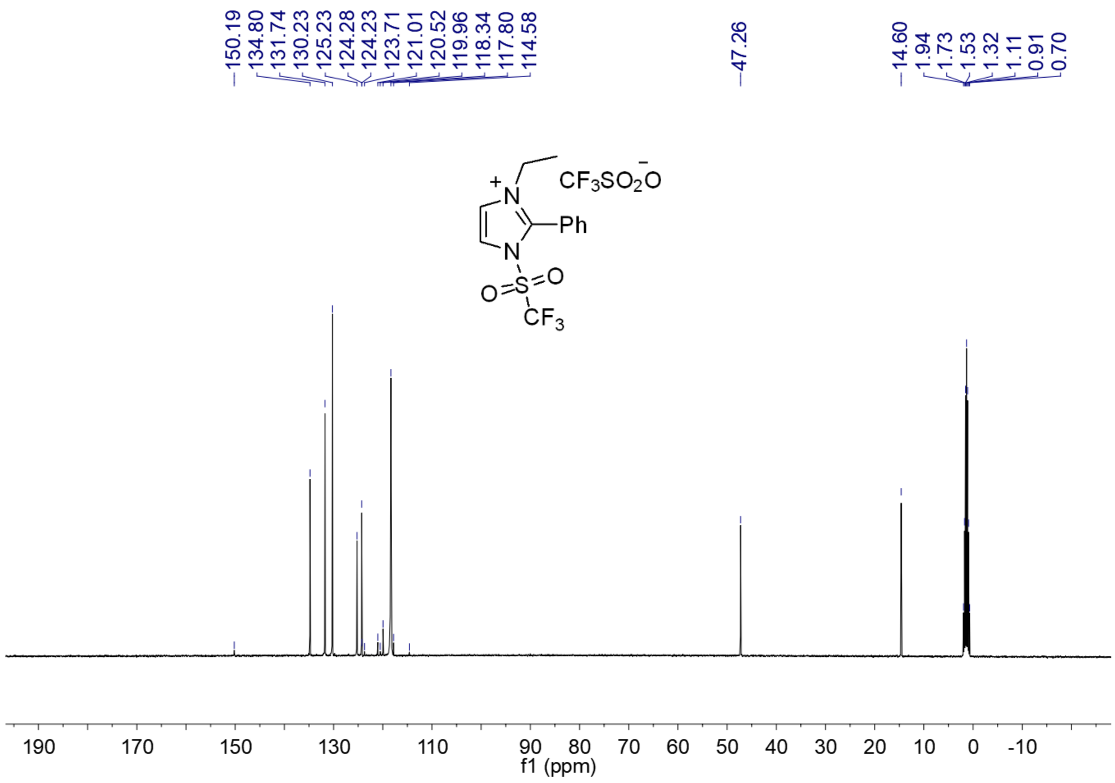

Supplementary Figure 36. ^{13}C NMR Spectra of product **1f**

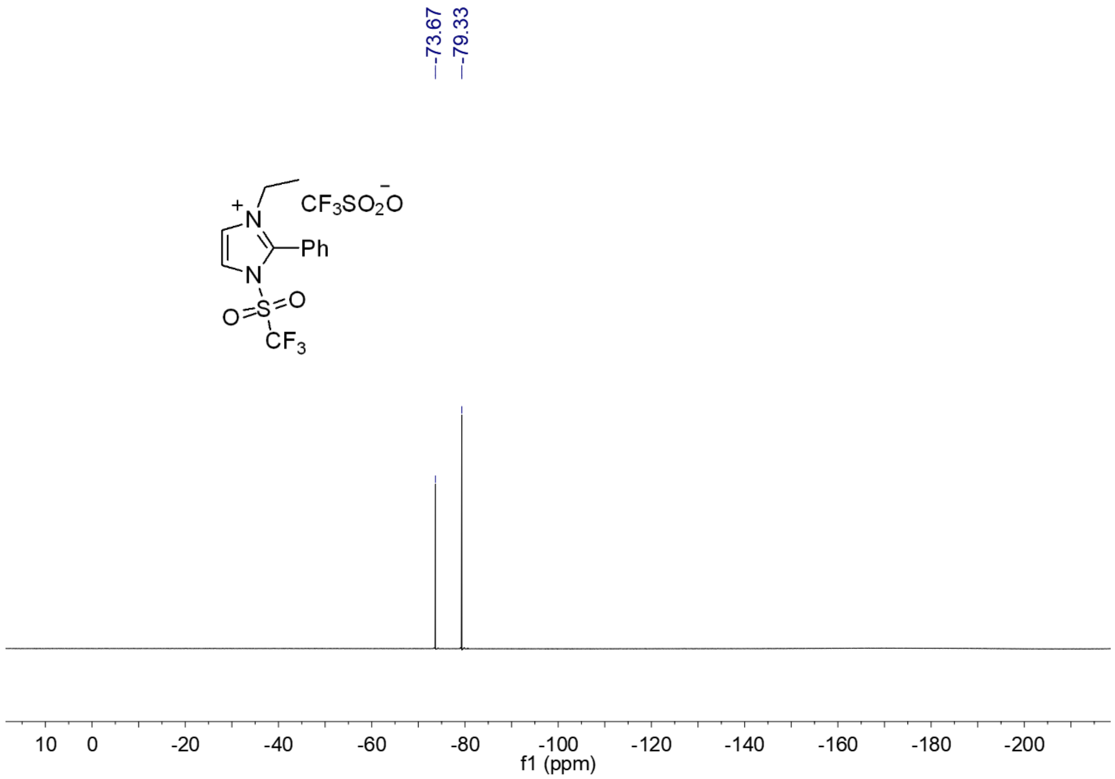

Supplementary Figure 37. ^{19}F NMR Spectra of product **1f**

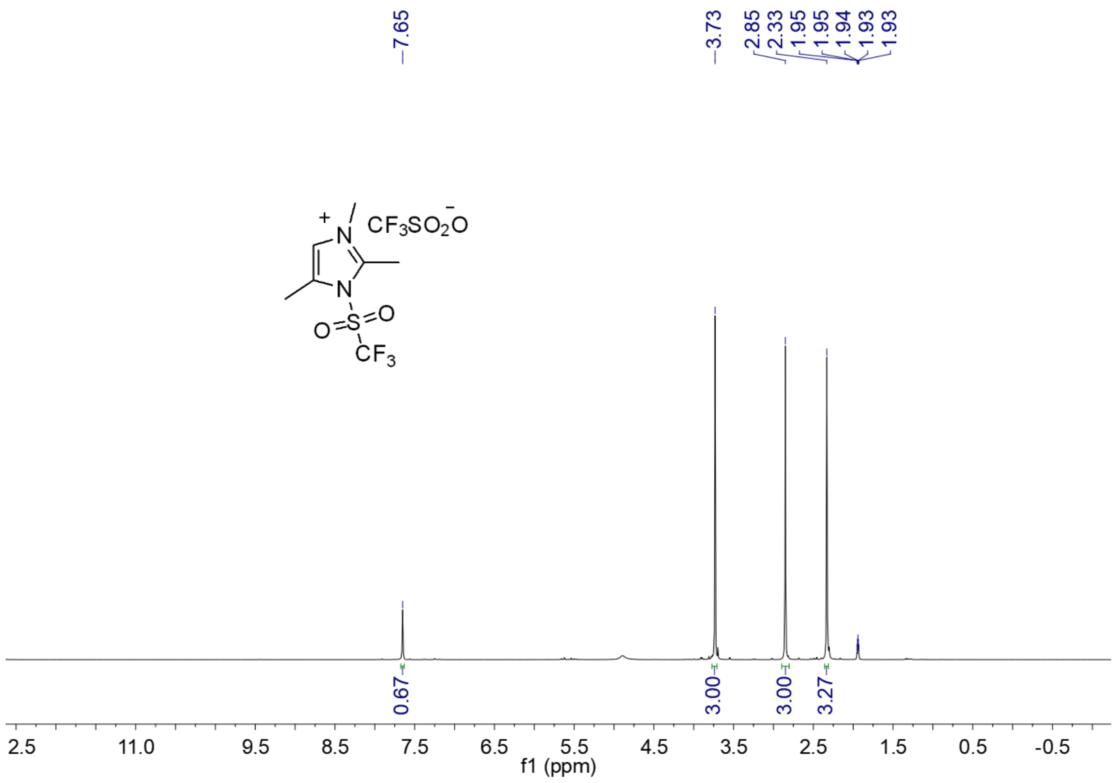

Supplementary Figure 38. ^{1}H NMR Spectra of product **1g**

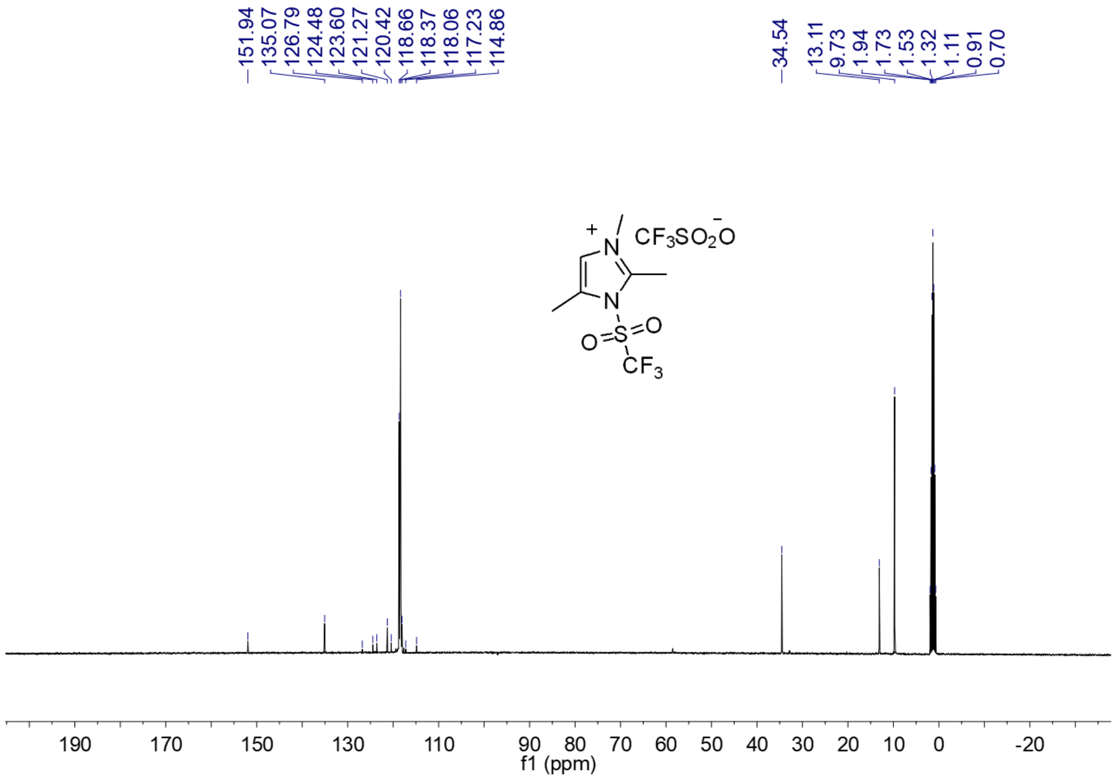

Supplementary Figure 39. ^{13}C NMR Spectra of product **1g**

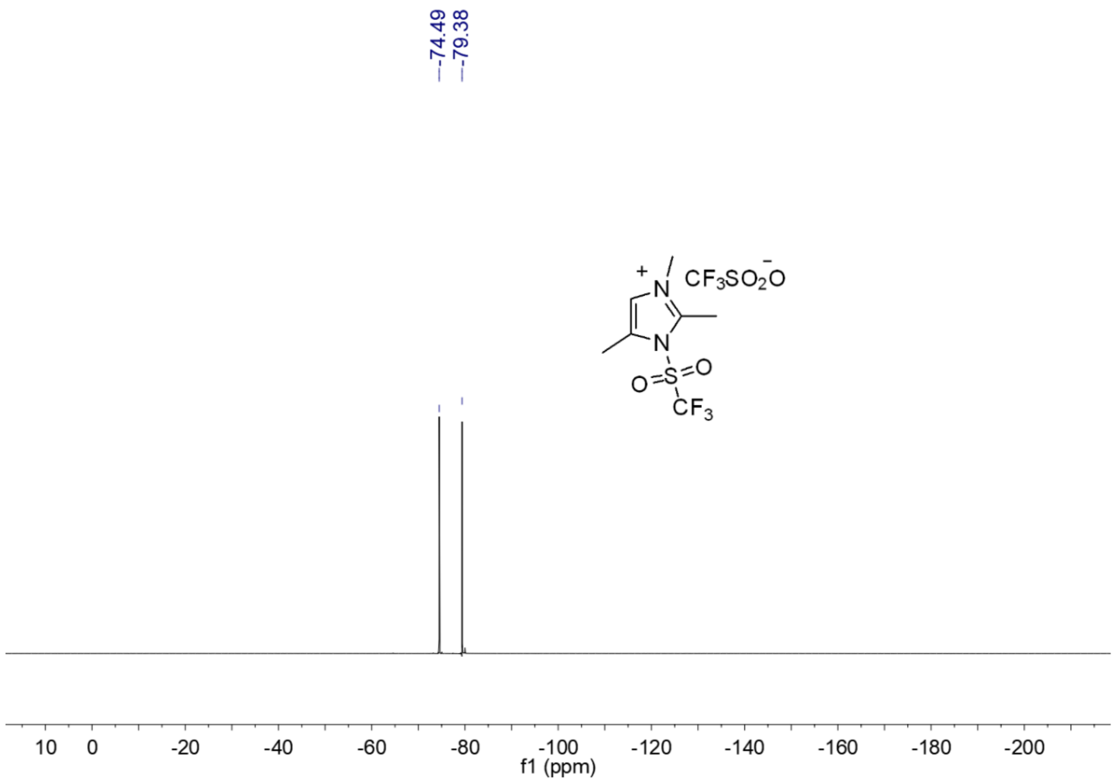

Supplementary Figure 40. ^{19}F NMR Spectra of product **1g**

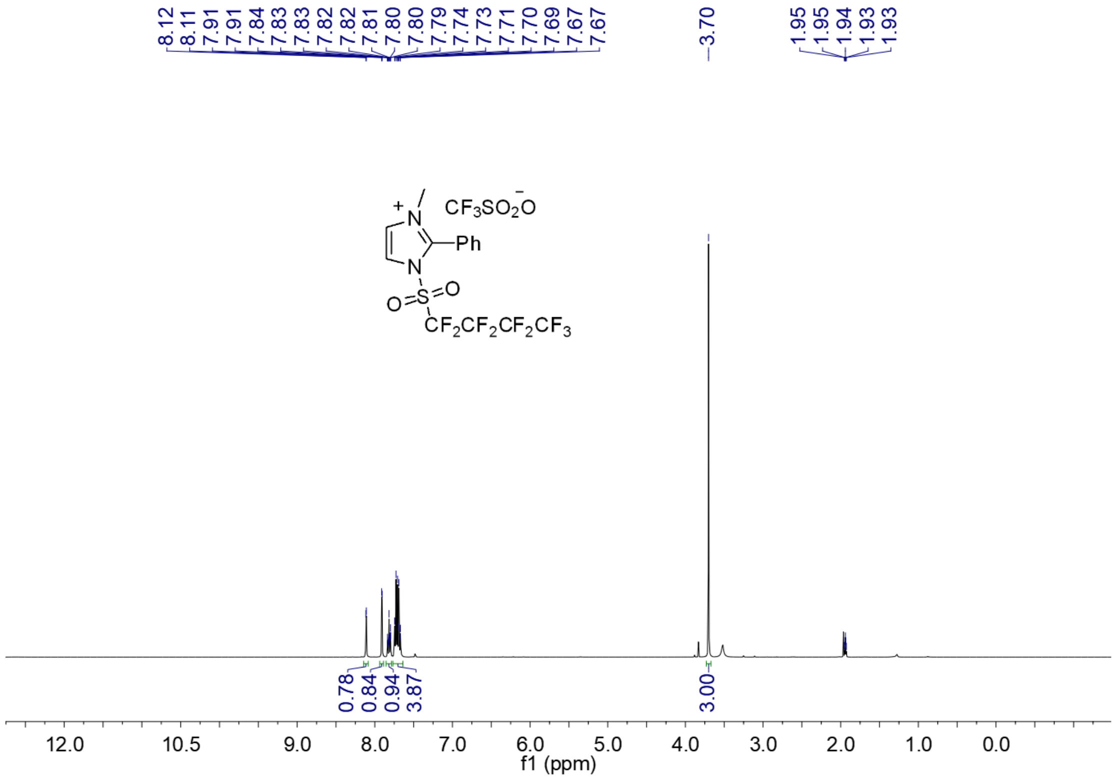

Supplementary Figure 41. ^{1}H NMR Spectra of product **1i**

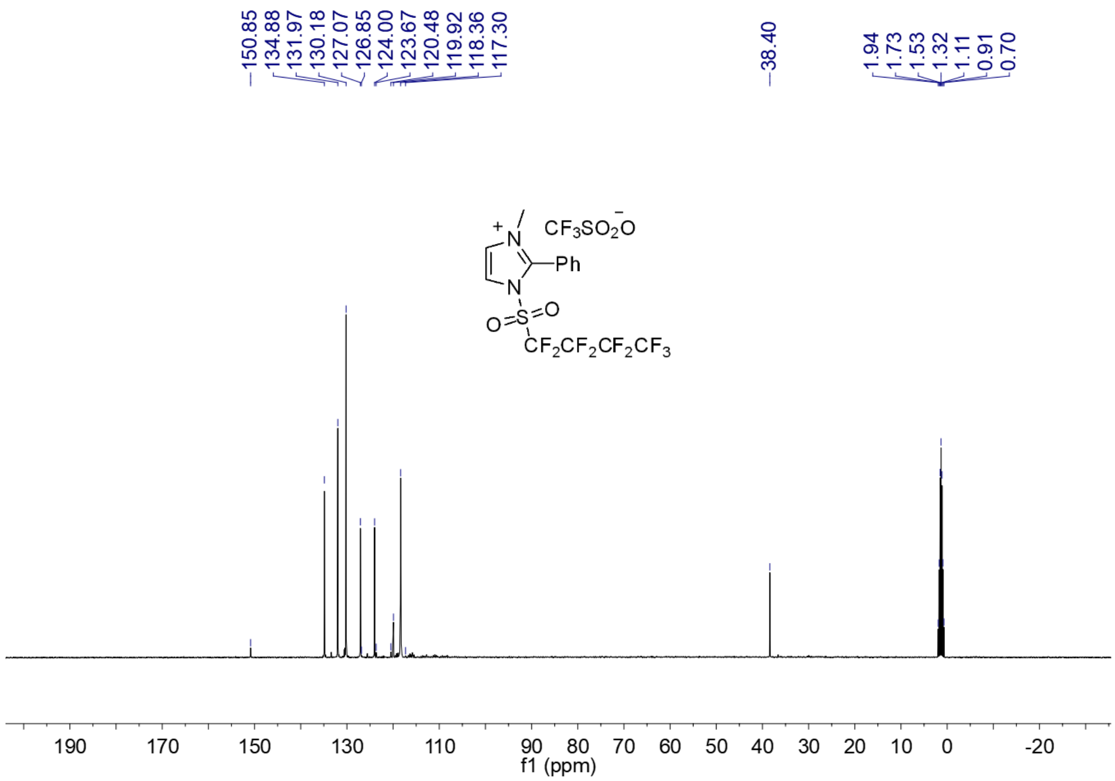

Supplementary Figure 42. ^{13}C NMR Spectra of product **1i**

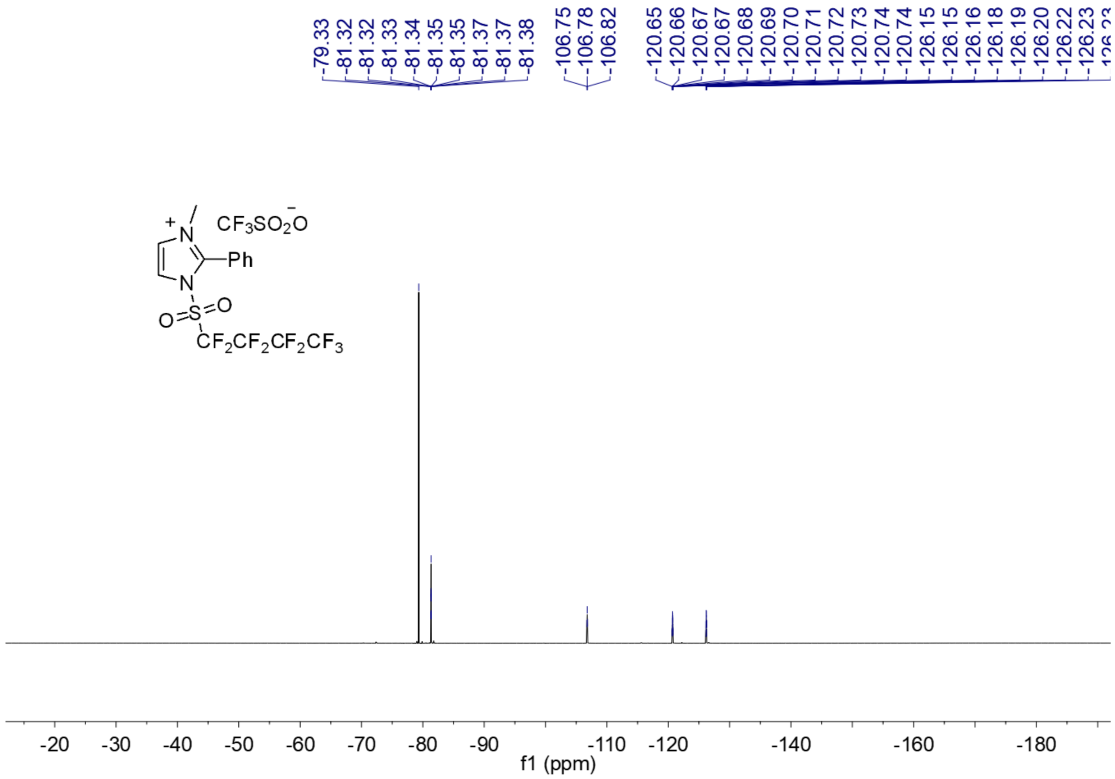

Supplementary Figure 43. ^{19}F NMR Spectra of product **1i**

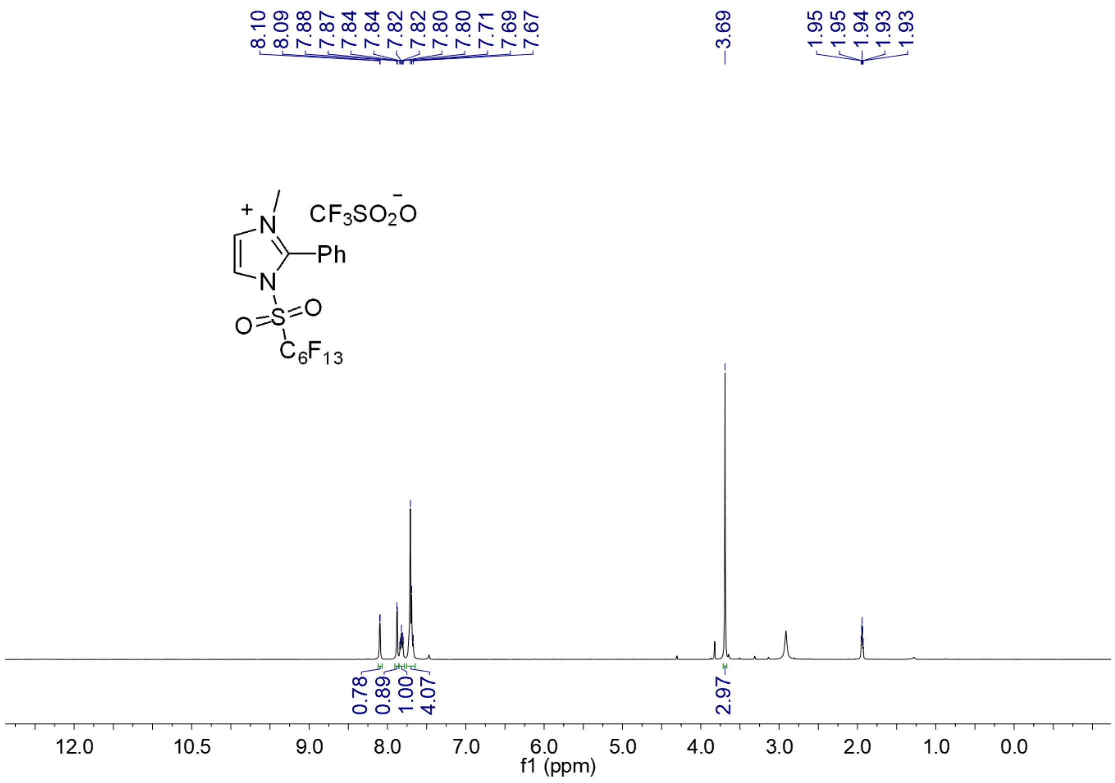

Supplementary Figure 44. ^{1}H NMR Spectra of product **1j**

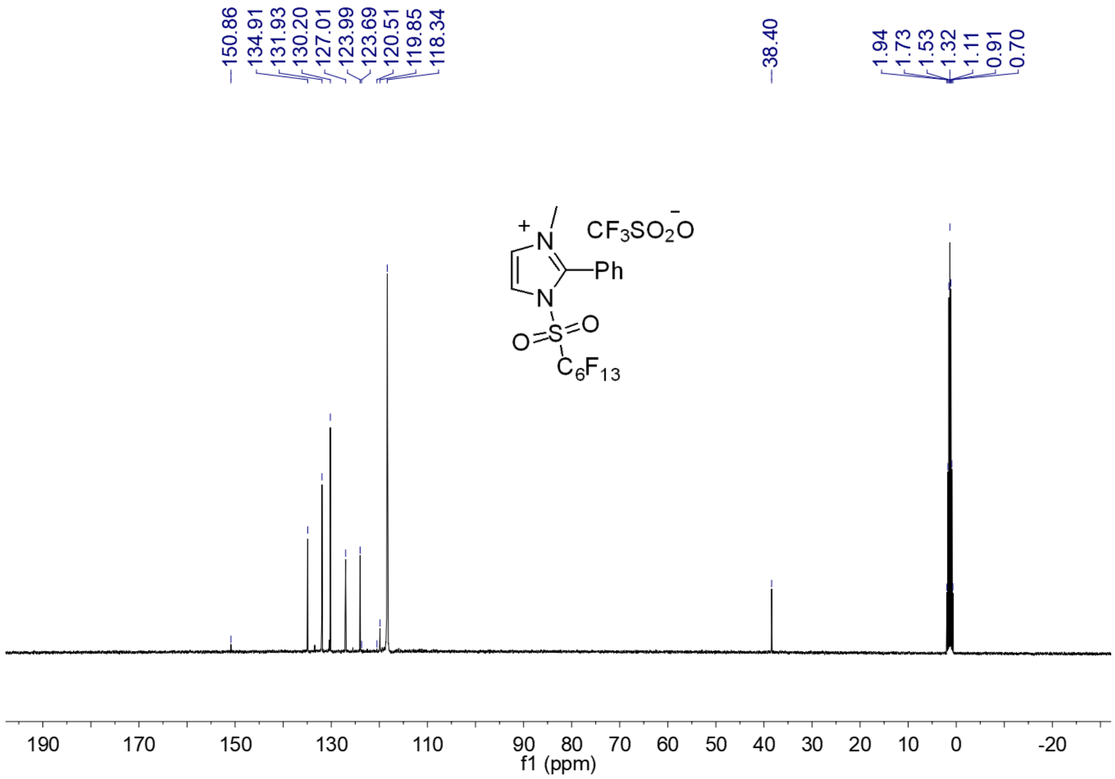

Supplementary Figure 45. ^{13}C NMR Spectra of product **1j**

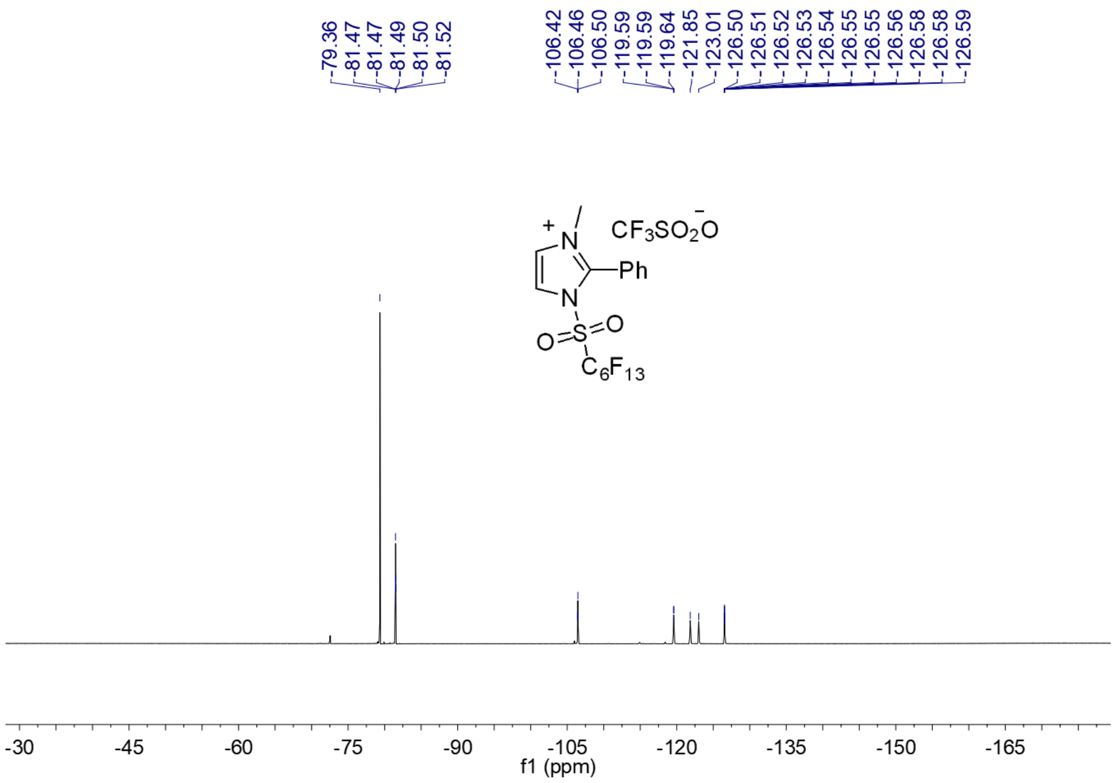

Supplementary Figure 46. ^{19}F NMR Spectra of product **1j**

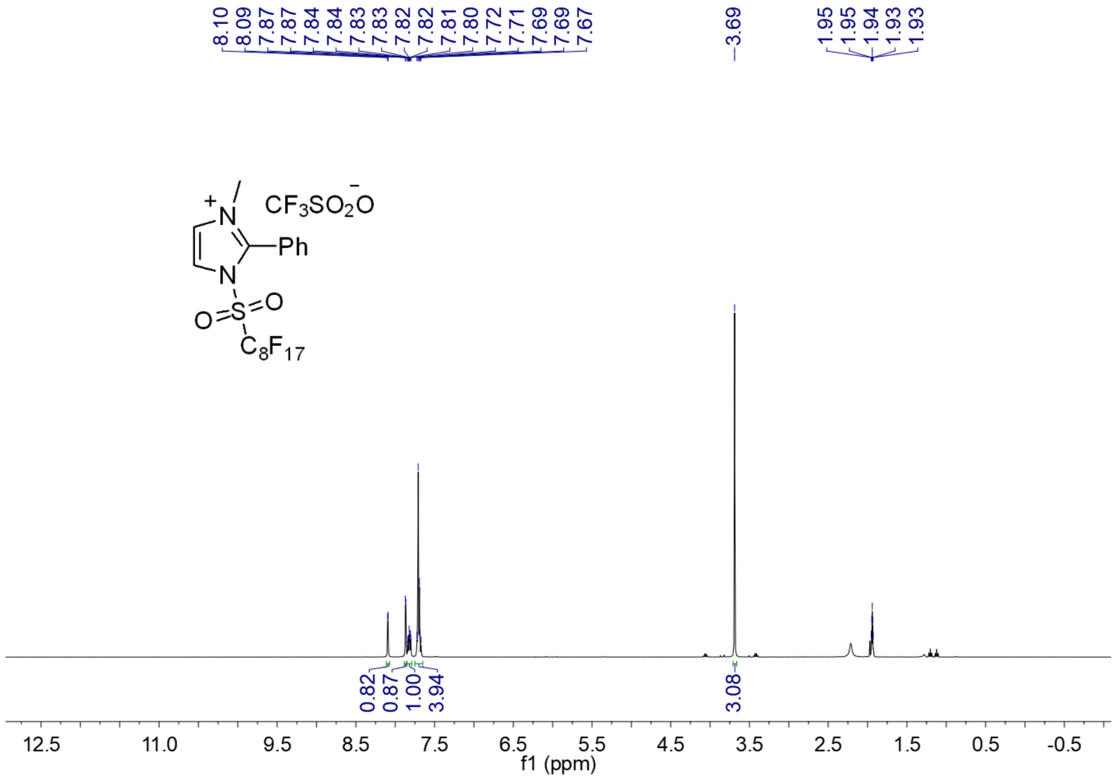

Supplementary Figure 47. ^{1}H NMR Spectra of product **1k**

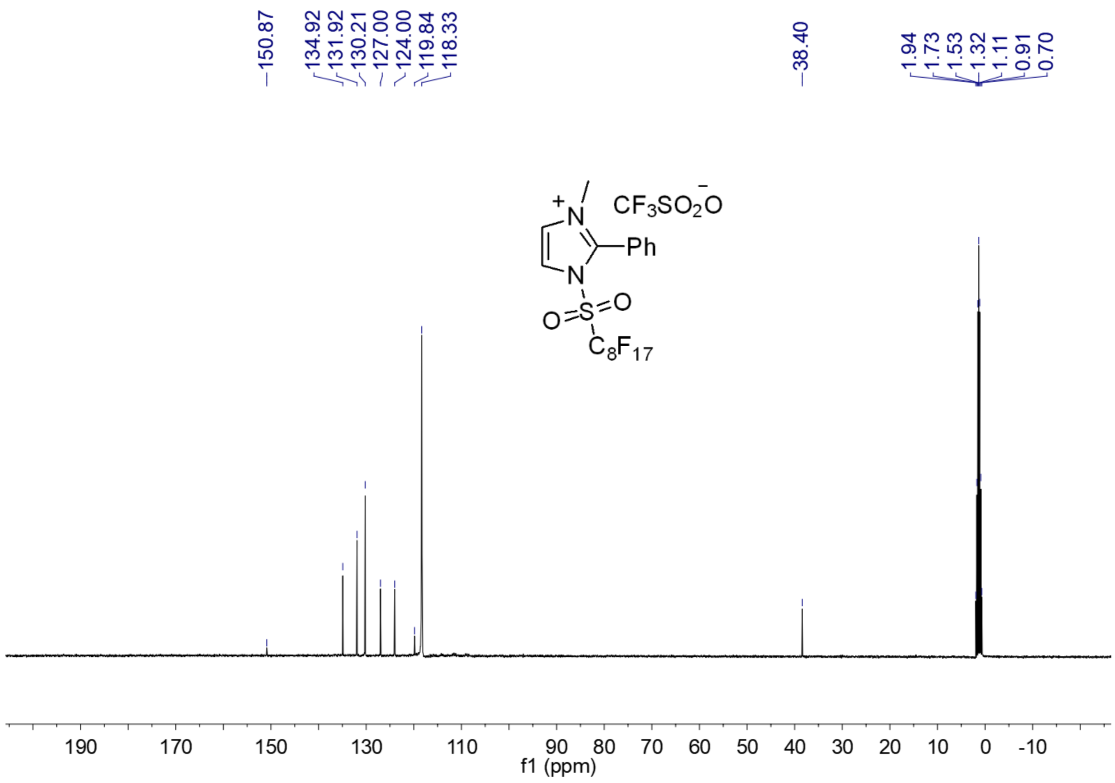

Supplementary Figure 48. ^{13}C NMR Spectra of product **1k**

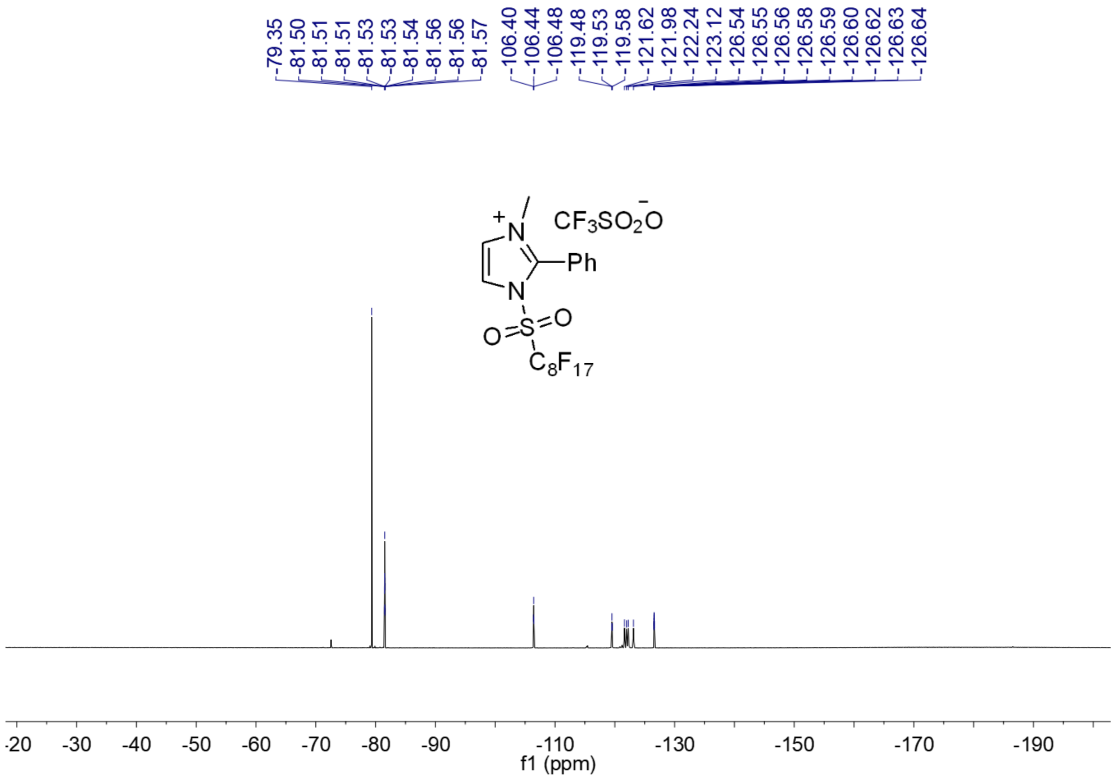

Supplementary Figure 49. ^{19}F NMR Spectra of product **1k**

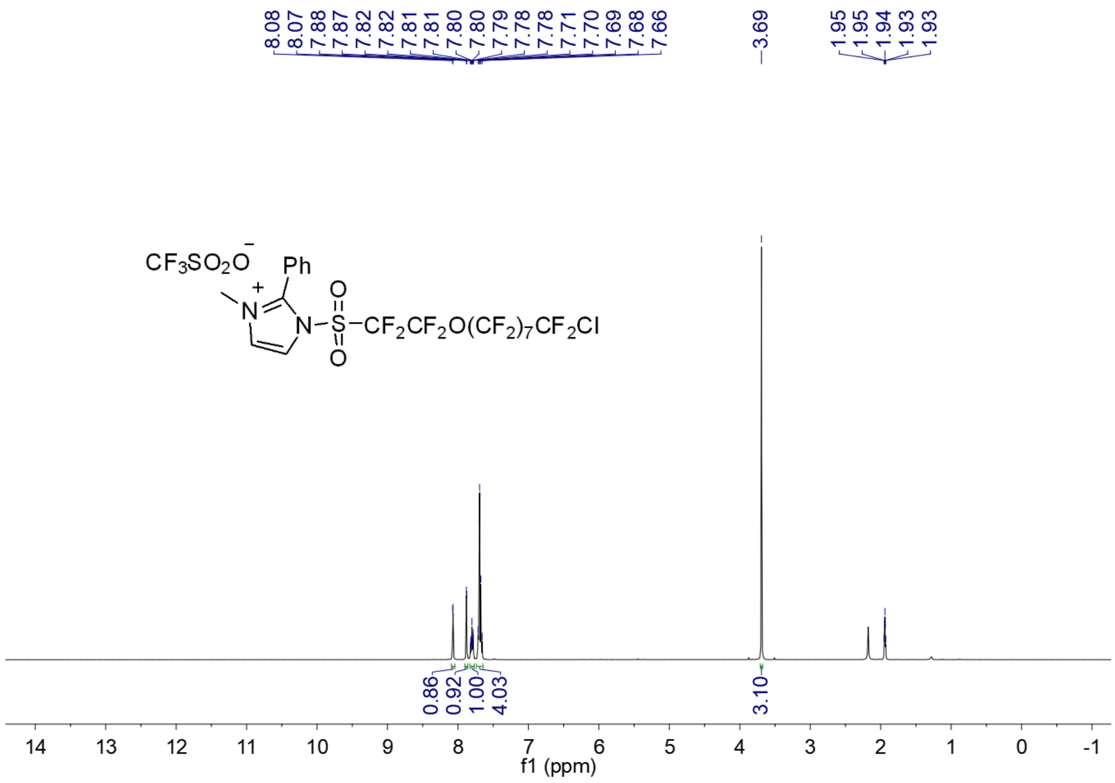

Supplementary Figure 50. ^{1}H NMR Spectra of product **1l**

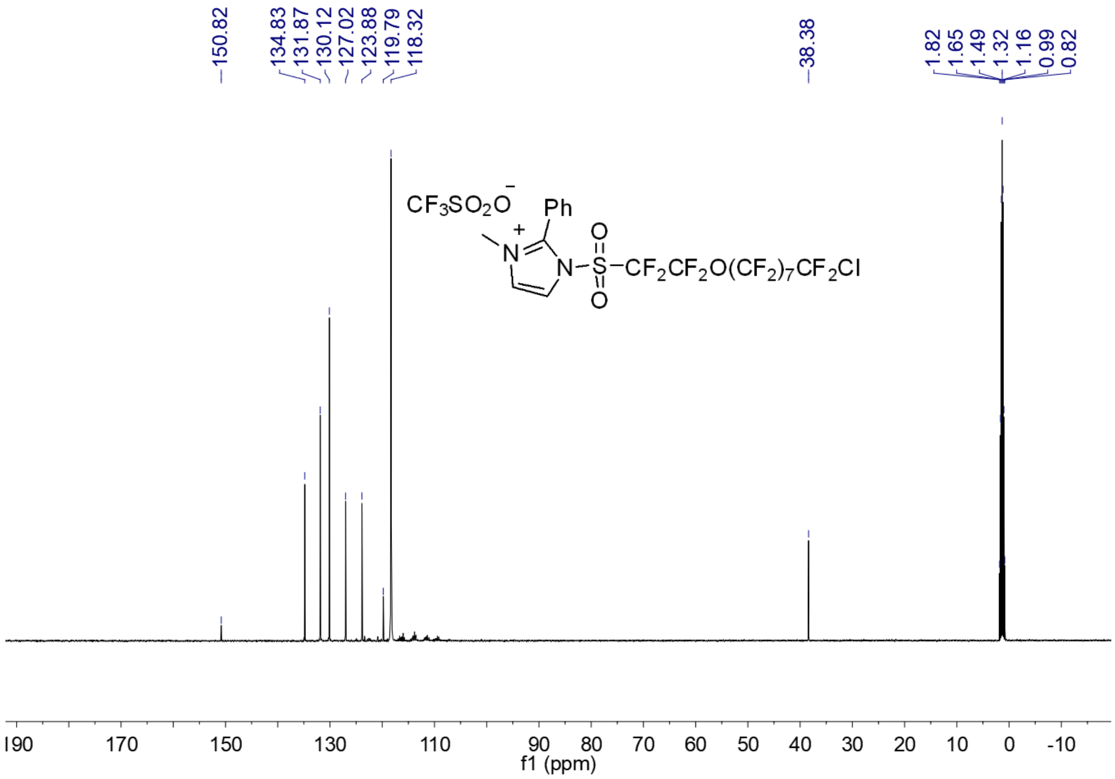

Supplementary Figure 51. ^{13}C NMR Spectra of product **1l**

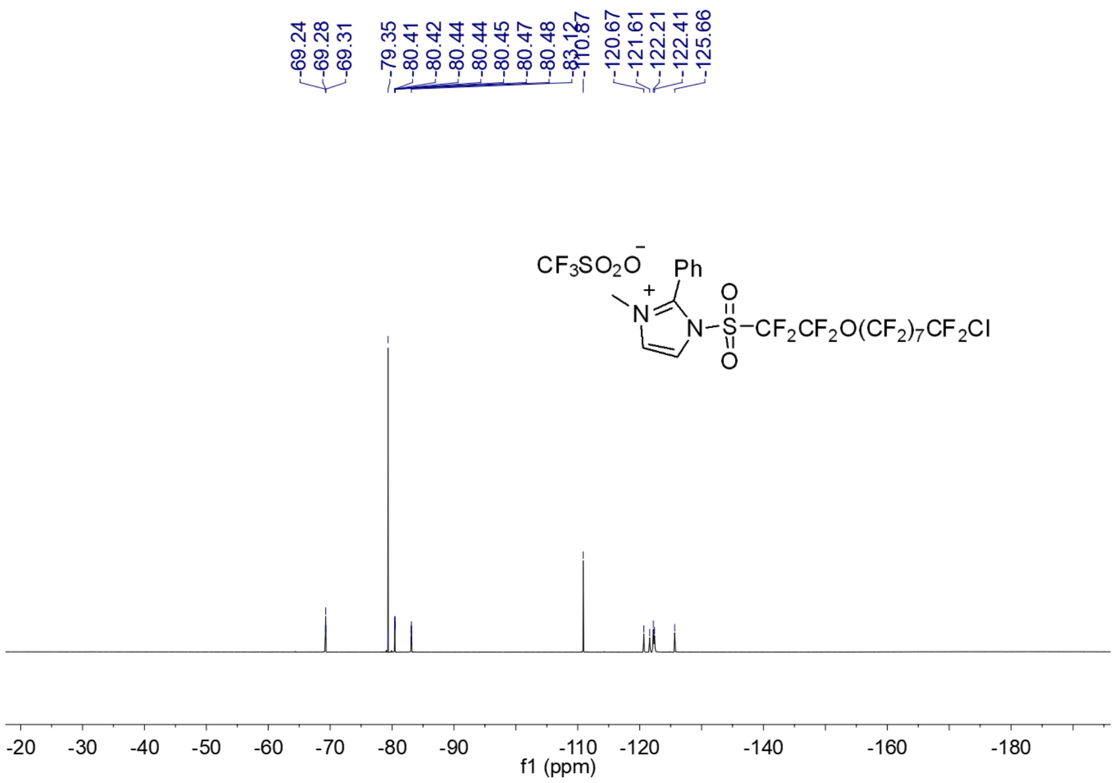

Supplementary Figure 52. ^{19}F NMR Spectra of product **1l**

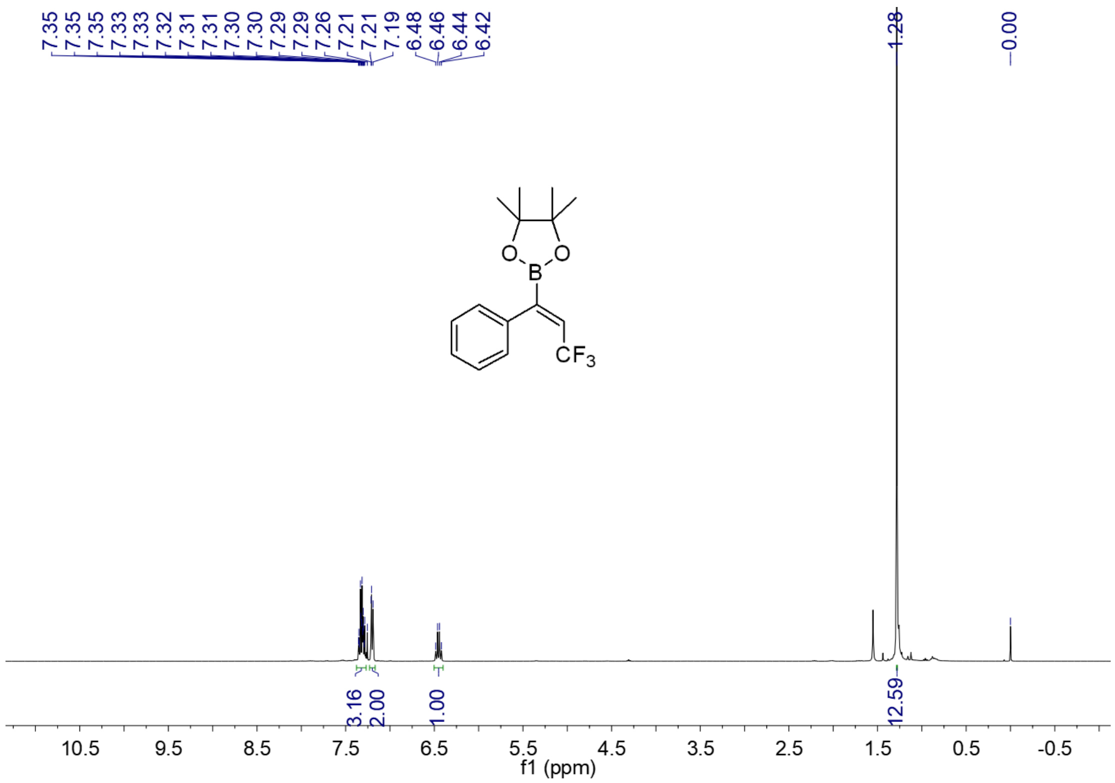

Supplementary Figure 53. ^{1}H NMR Spectra of product **3a**

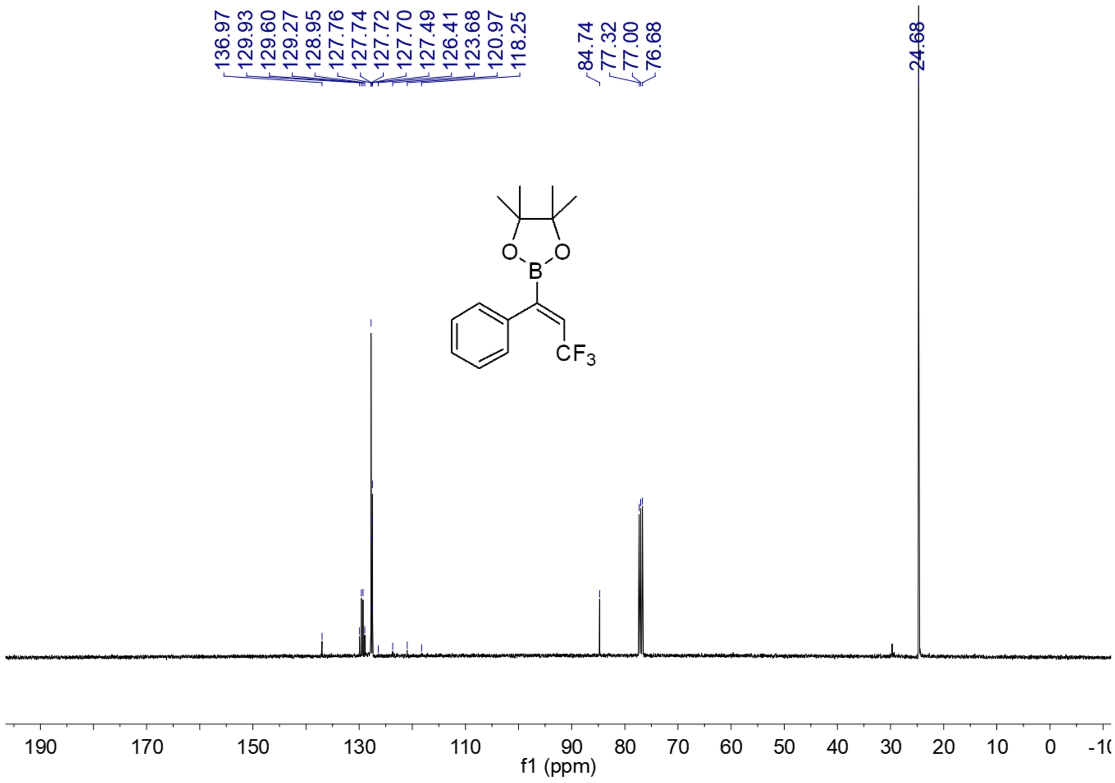

Supplementary Figure 54. ^{13}C NMR Spectra of product **3a**

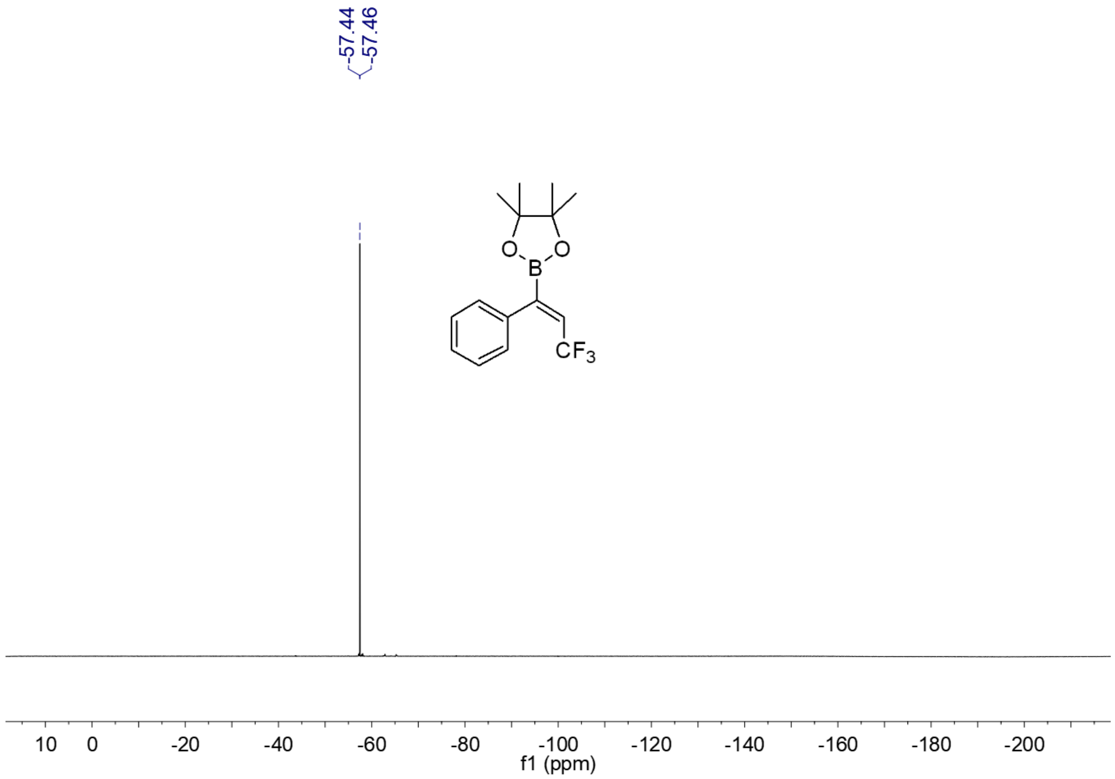

Supplementary Figure 55. ^{19}F NMR Spectra of product **3a**

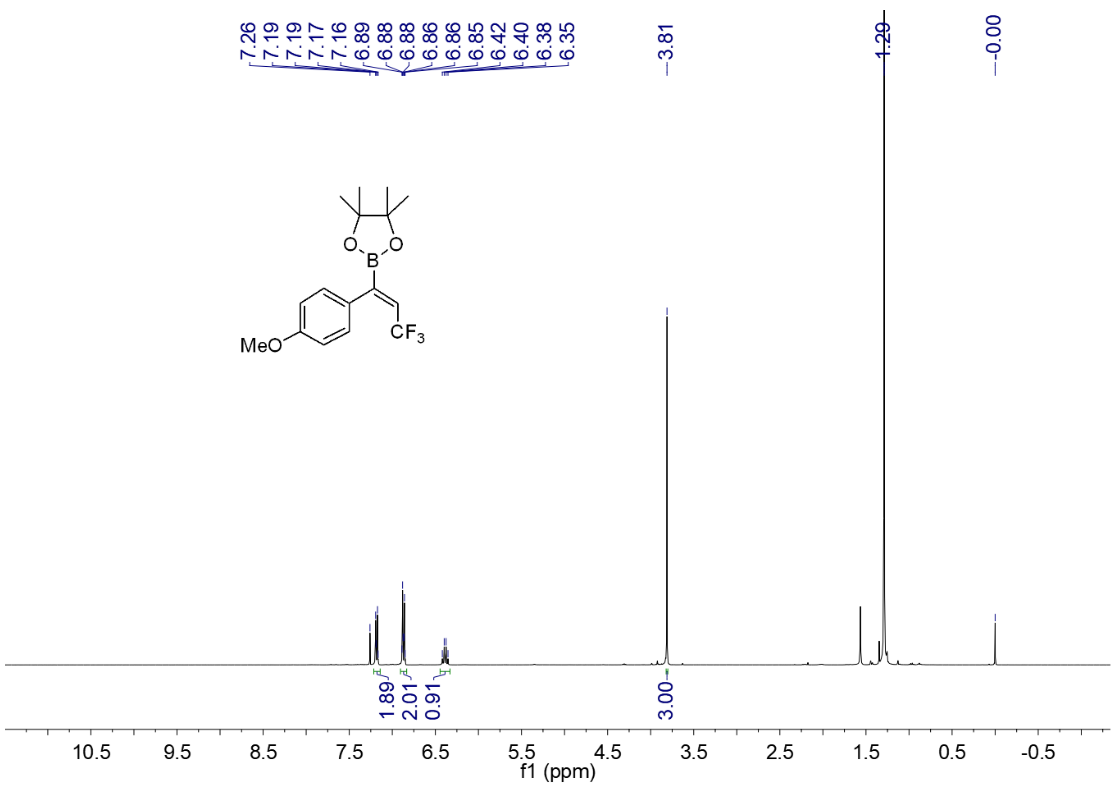

Supplementary Figure 56. ^{1}H NMR Spectra of product **3b**

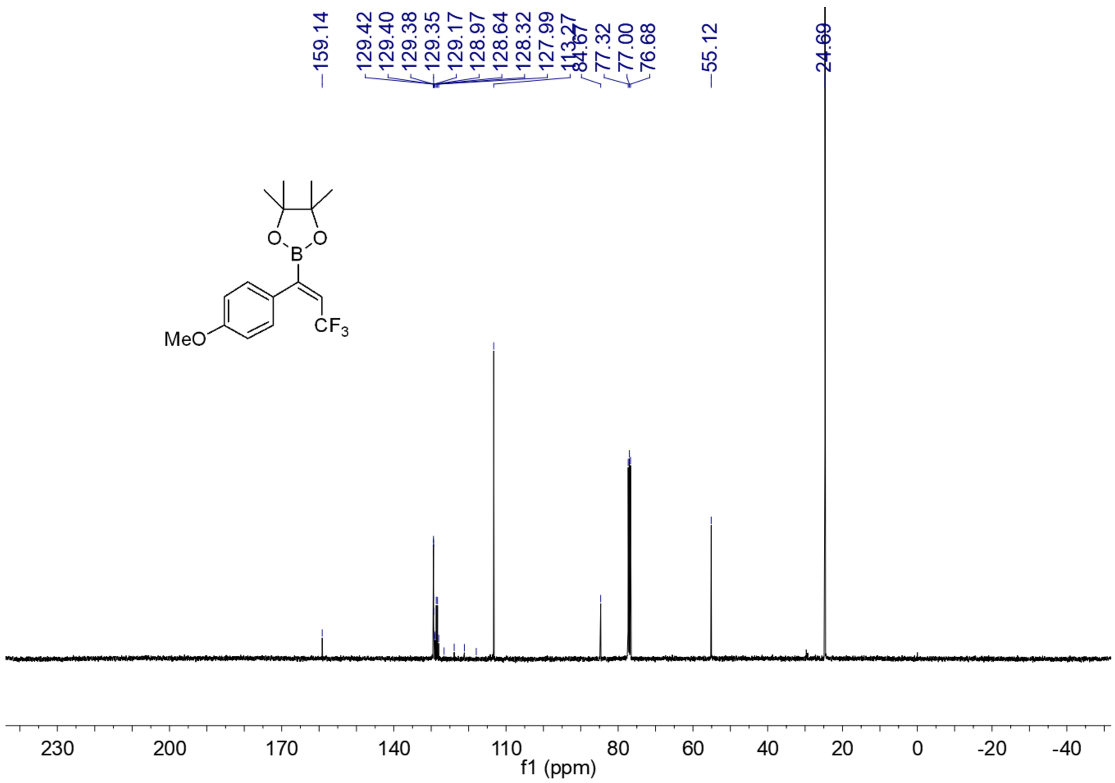

Supplementary Figure 57. ^{13}C NMR Spectra of product **3b**

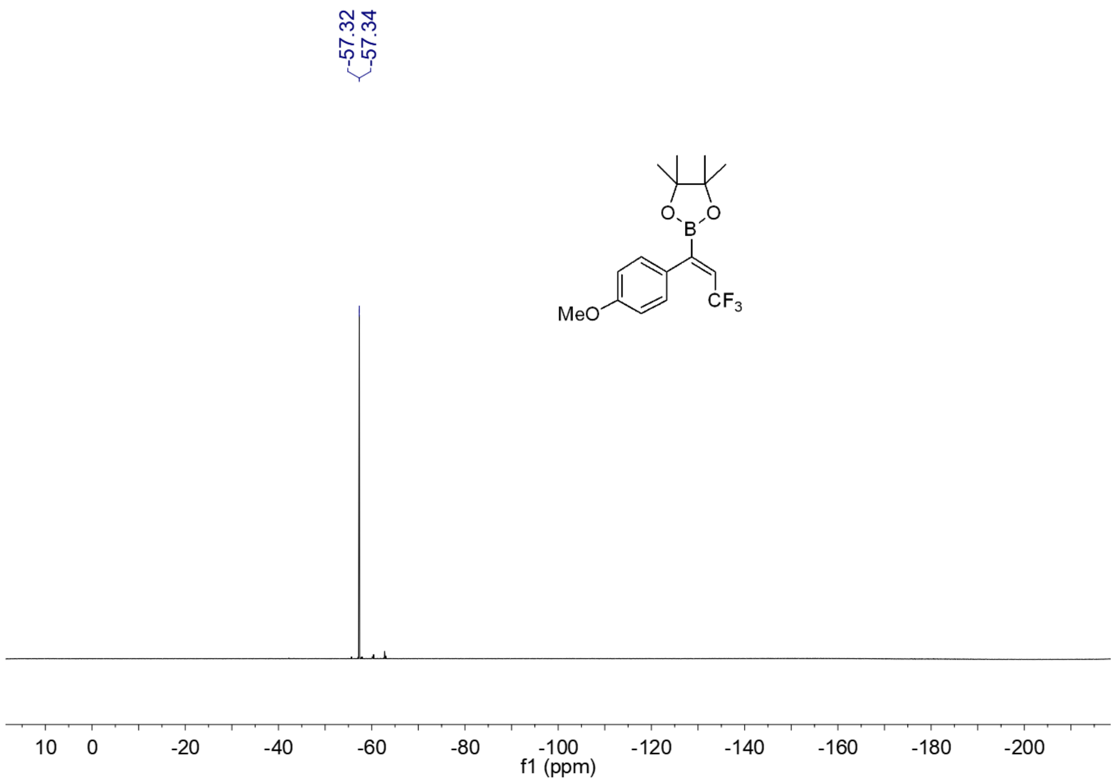

Supplementary Figure 58. ^{19}F NMR Spectra of product **3b**

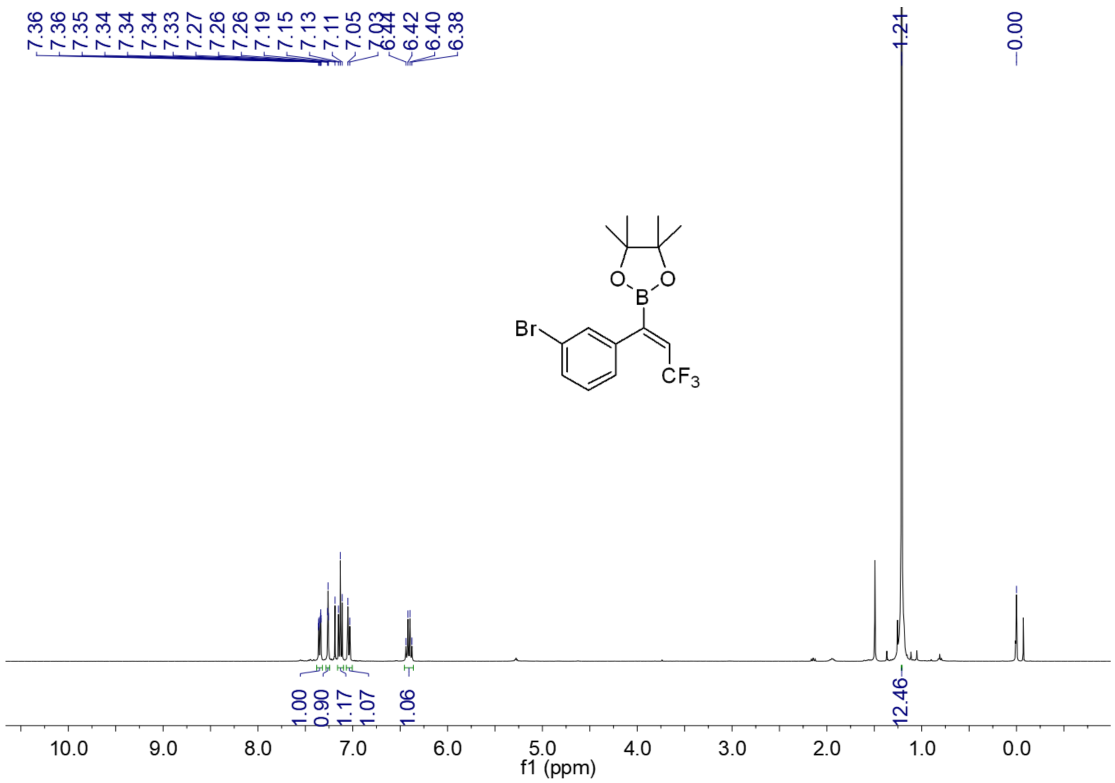

Supplementary Figure 59. ^{1}H NMR Spectra of product **3c**

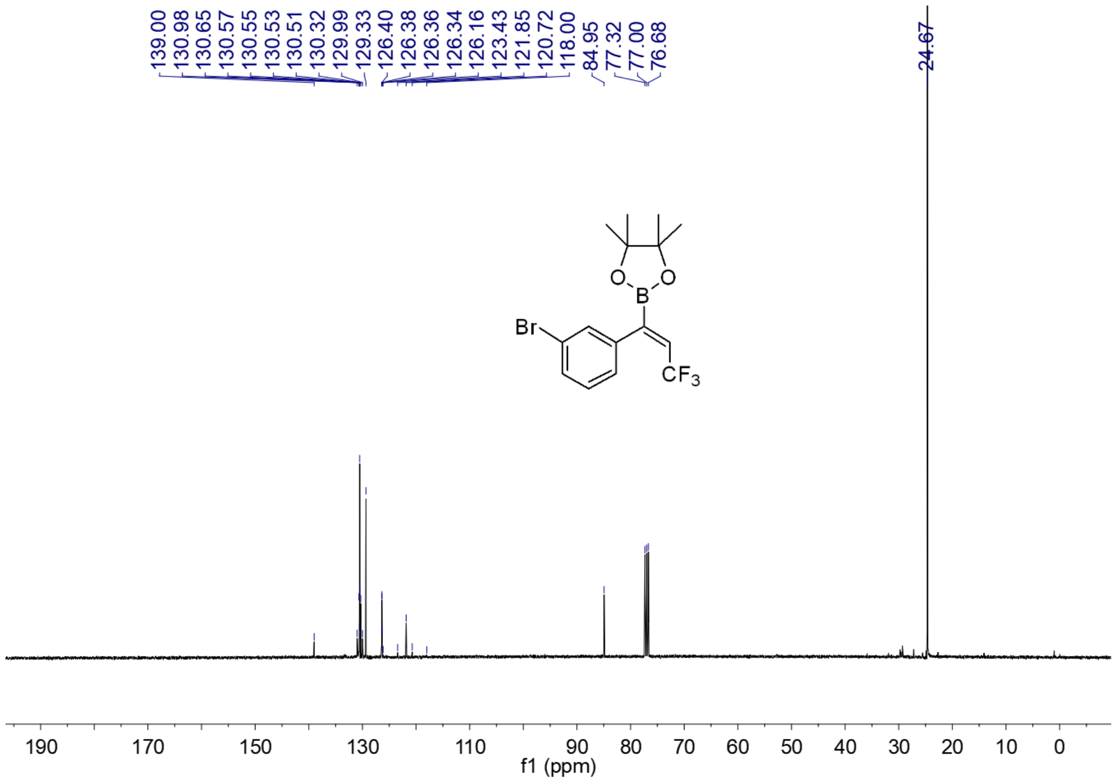

Supplementary Figure 60. ^{13}C NMR Spectra of product **3c**

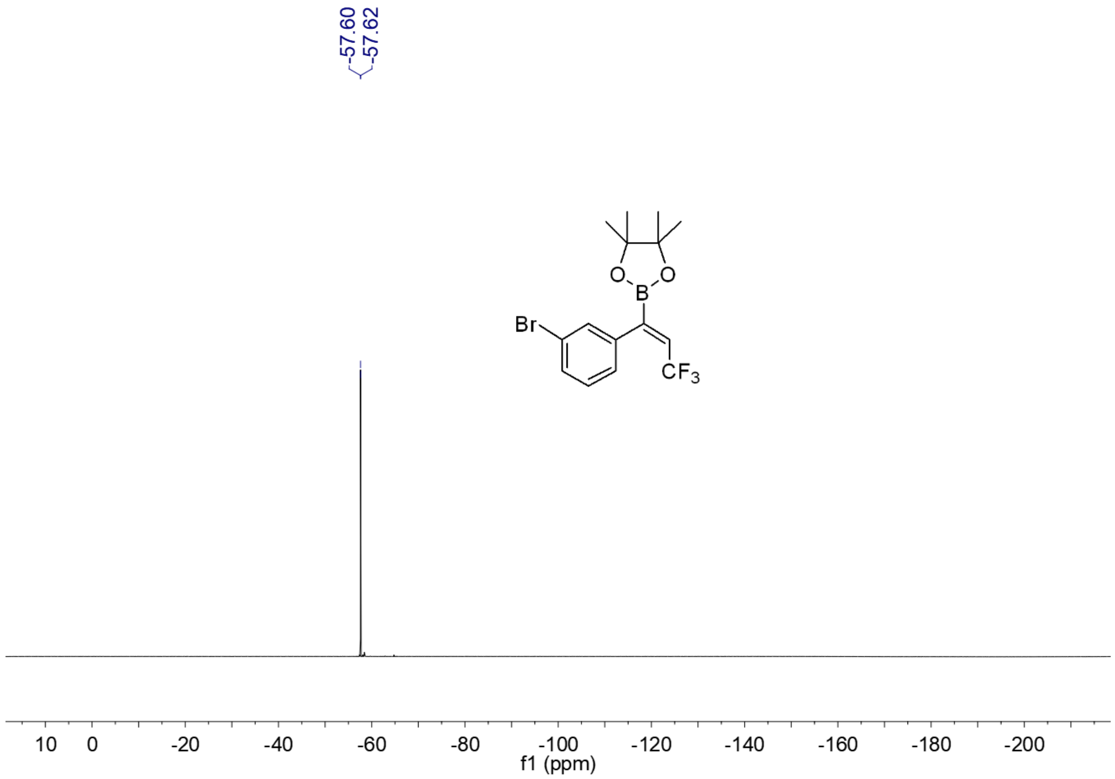

Supplementary Figure 61. ^{19}F NMR Spectra of product **3c**

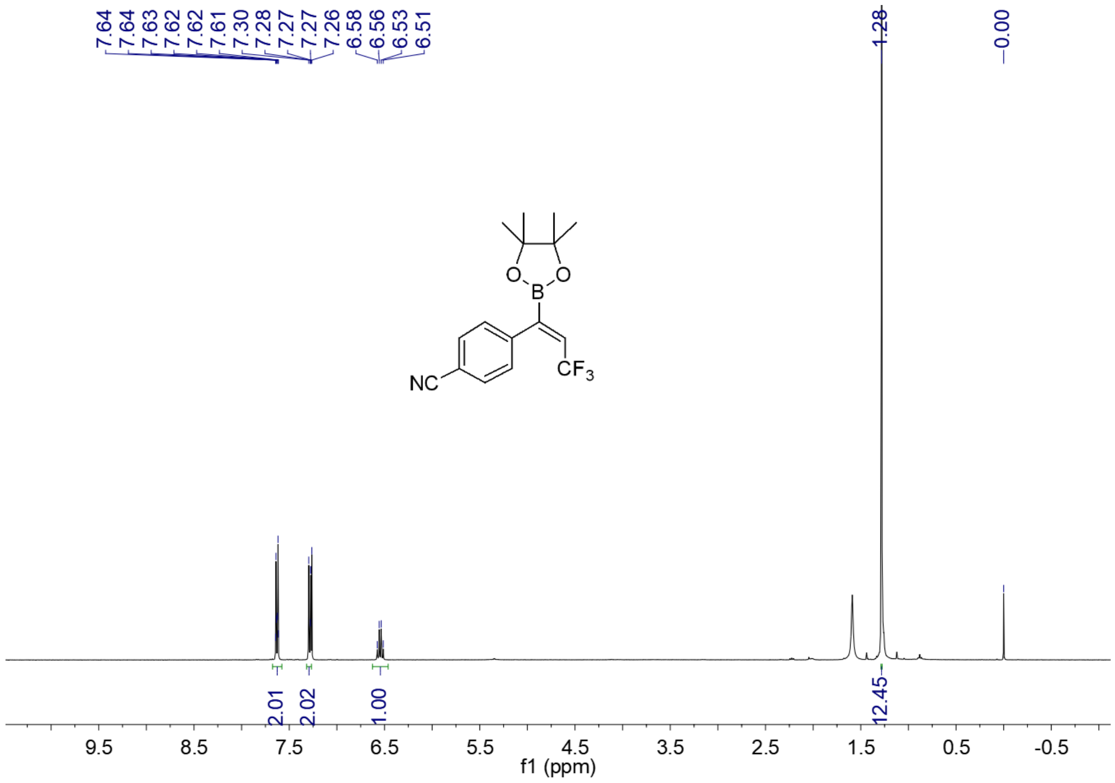

Supplementary Figure 62. ^{1}H NMR Spectra of product **3d**

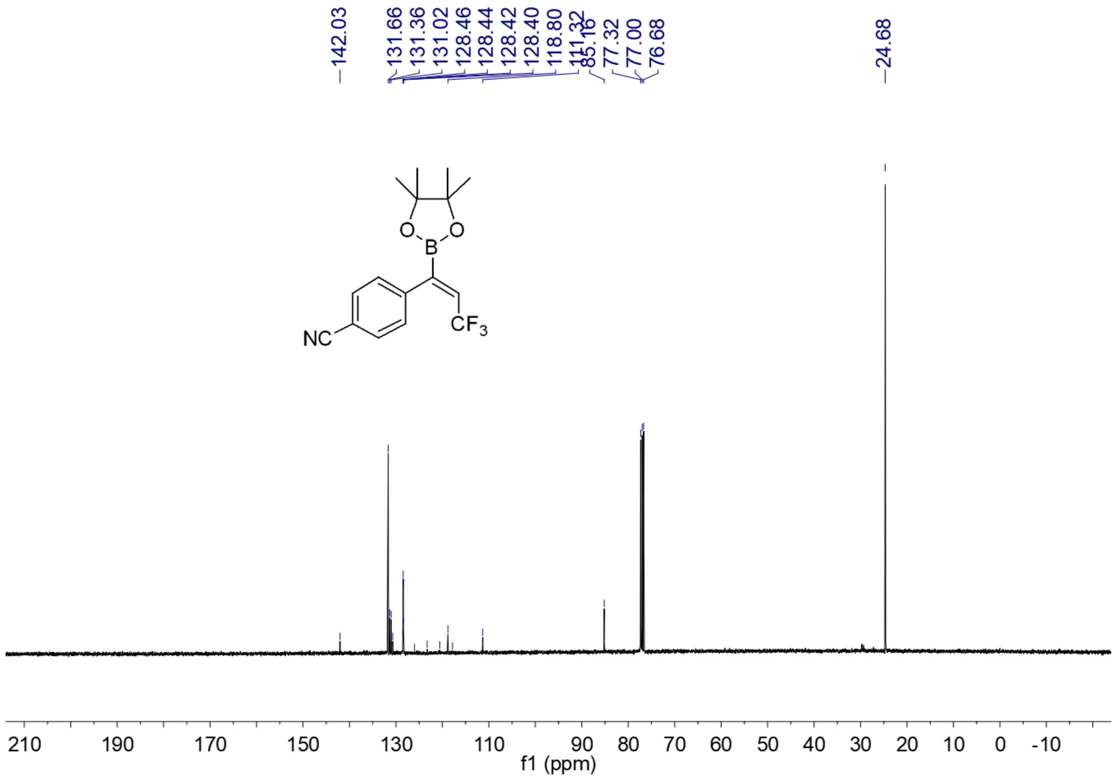

Supplementary Figure 63. ^{13}C NMR Spectra of product **3d**

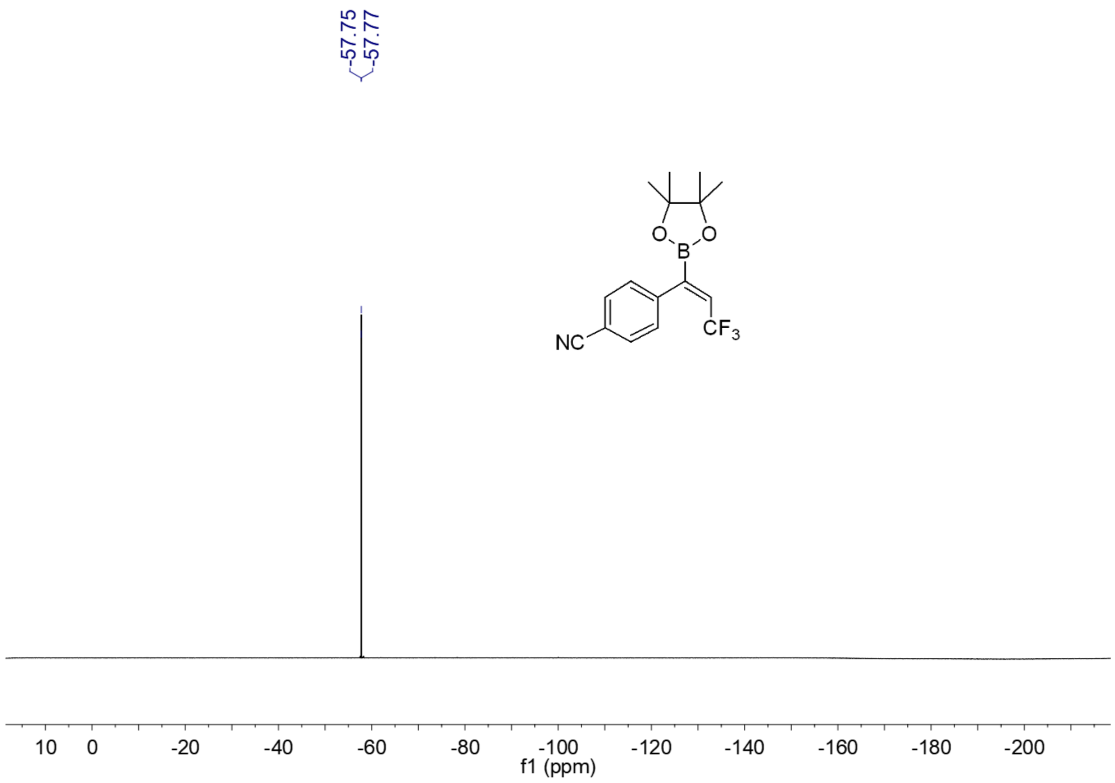

Supplementary Figure 64. ^{19}F NMR Spectra of product **3d**

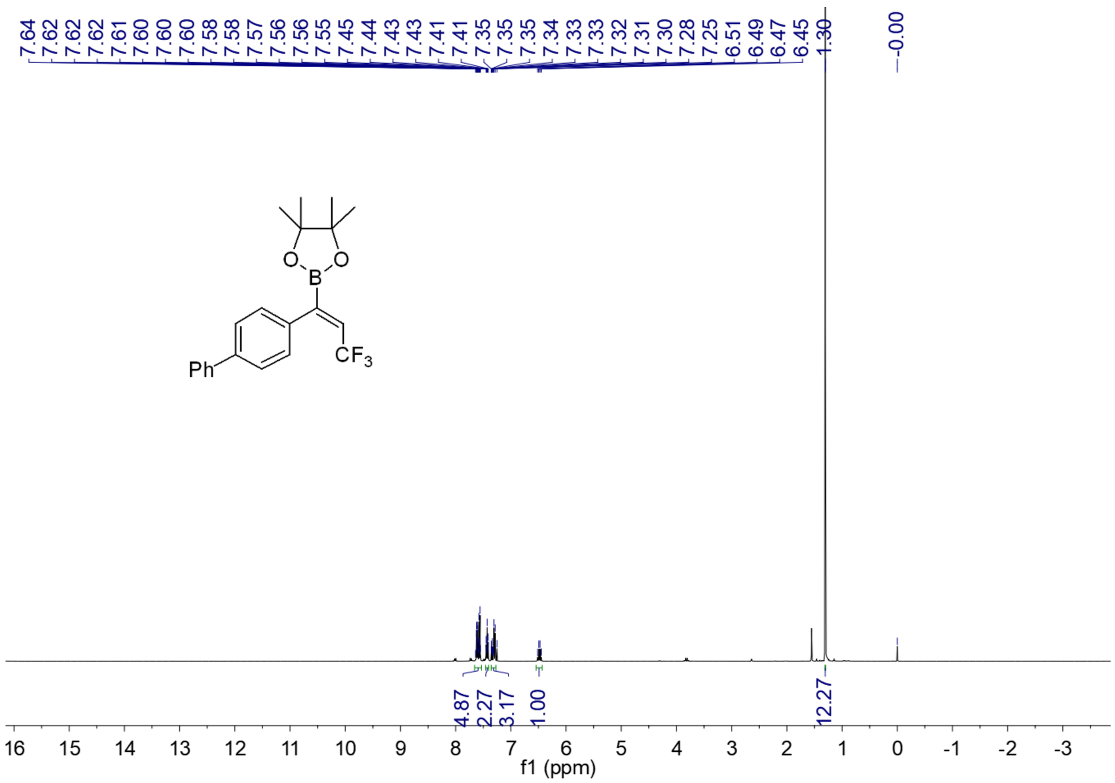

Supplementary Figure 65. ^{1}H NMR Spectra of product **3e**

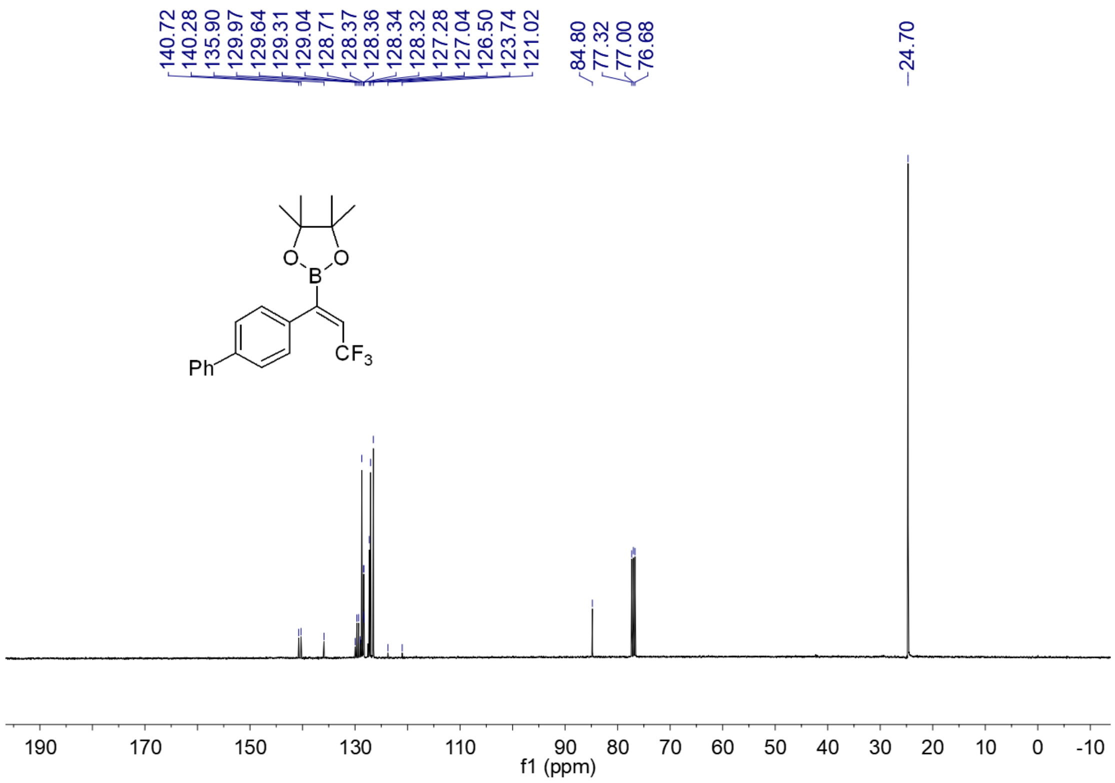

Supplementary Figure 66. ^{13}C NMR Spectra of product **3e**

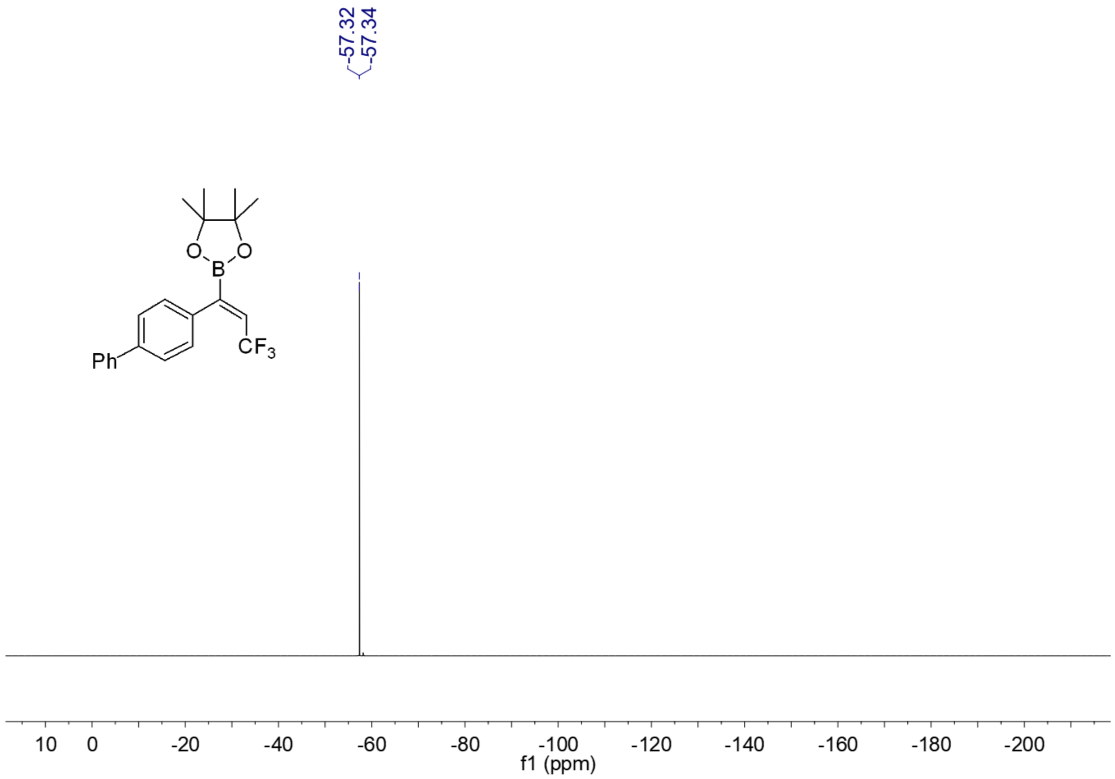

Supplementary Figure 67. ^{19}F NMR Spectra of product **3e**

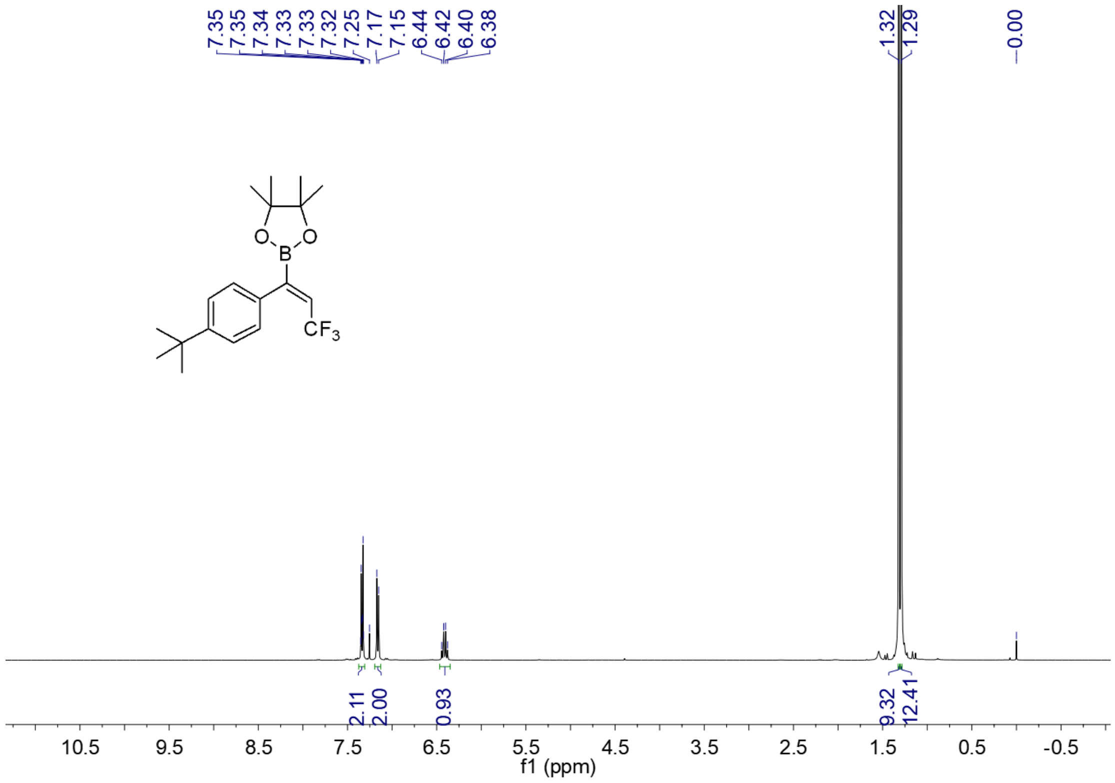

Supplementary Figure 68. ^{1}H NMR Spectra of product **3f**

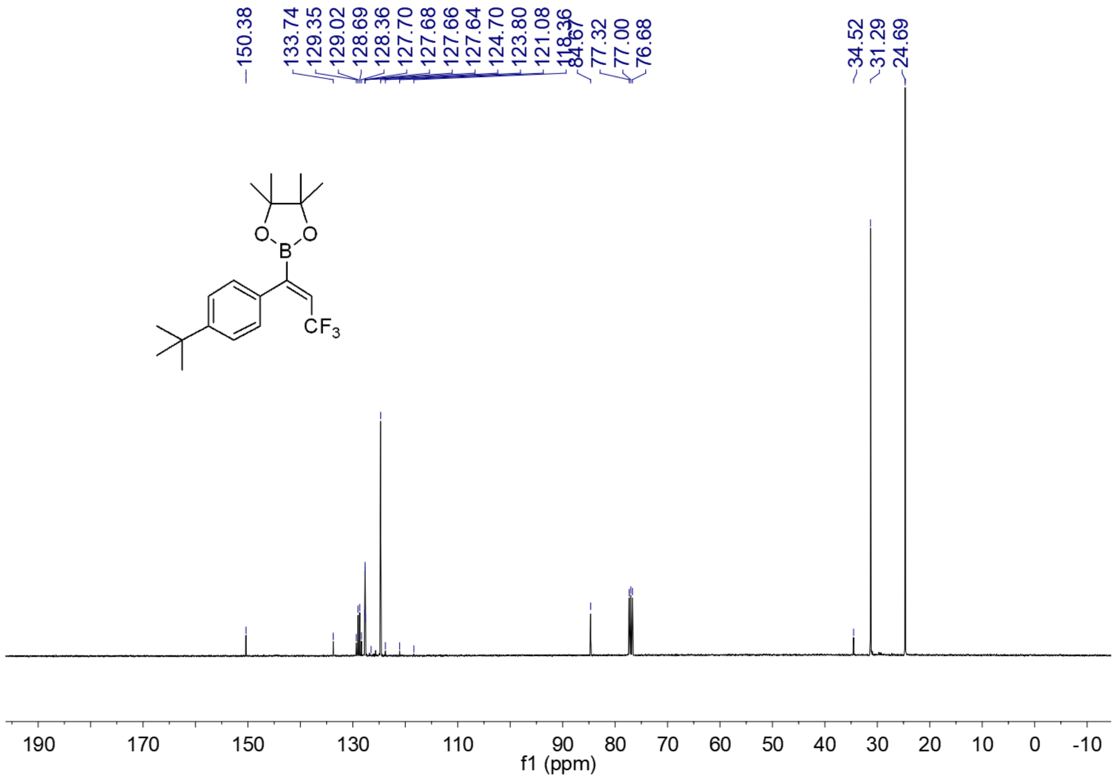

Supplementary Figure 69. ^{13}C NMR Spectra of product **3f**

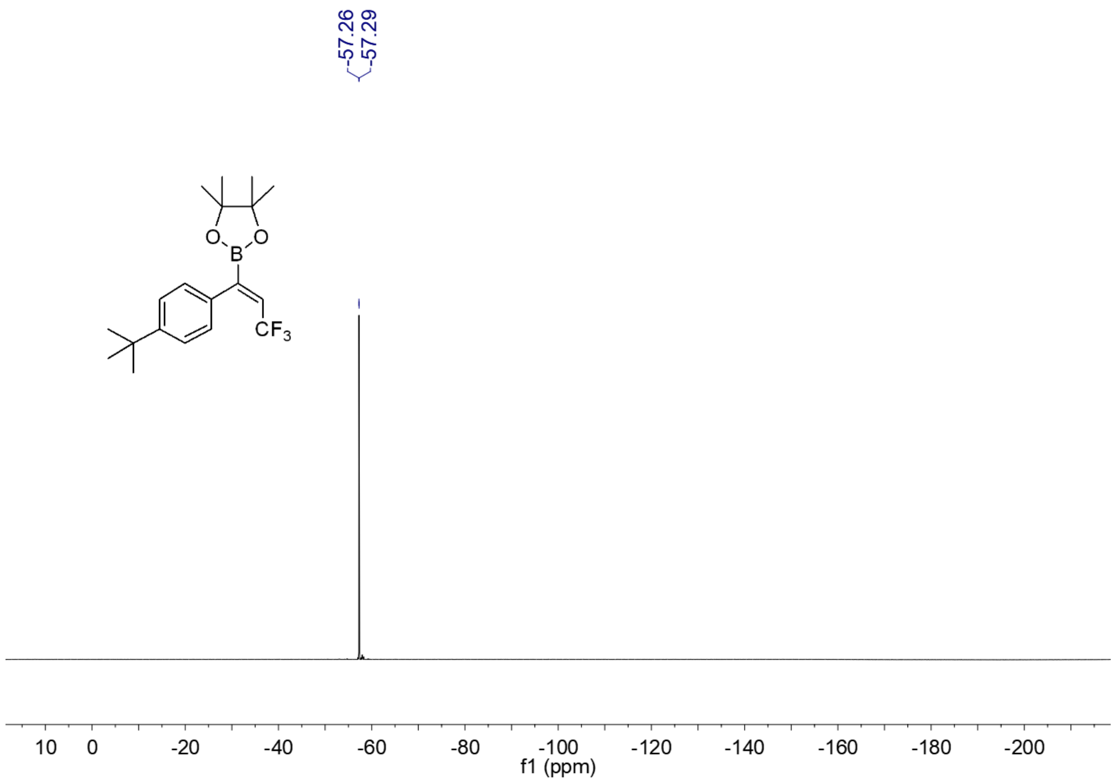

Supplementary Figure 70. ^{19}F NMR Spectra of product **3f**

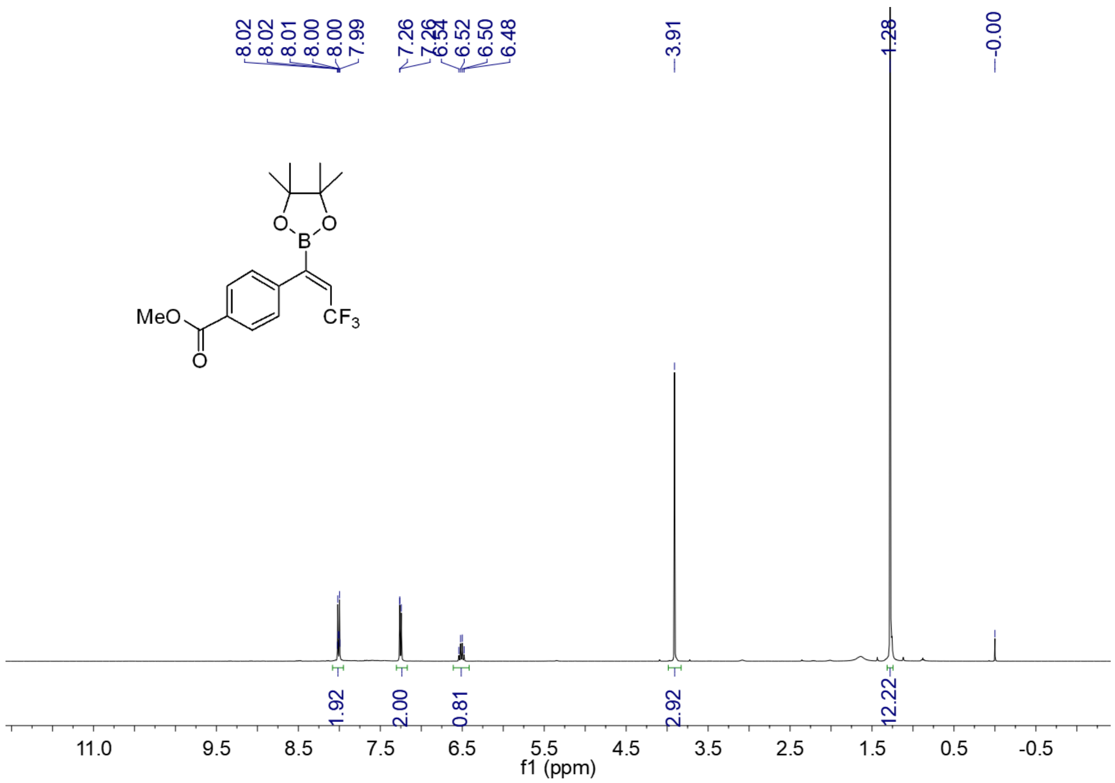

Supplementary Figure 71. ^{1}H NMR Spectra of product **3g**

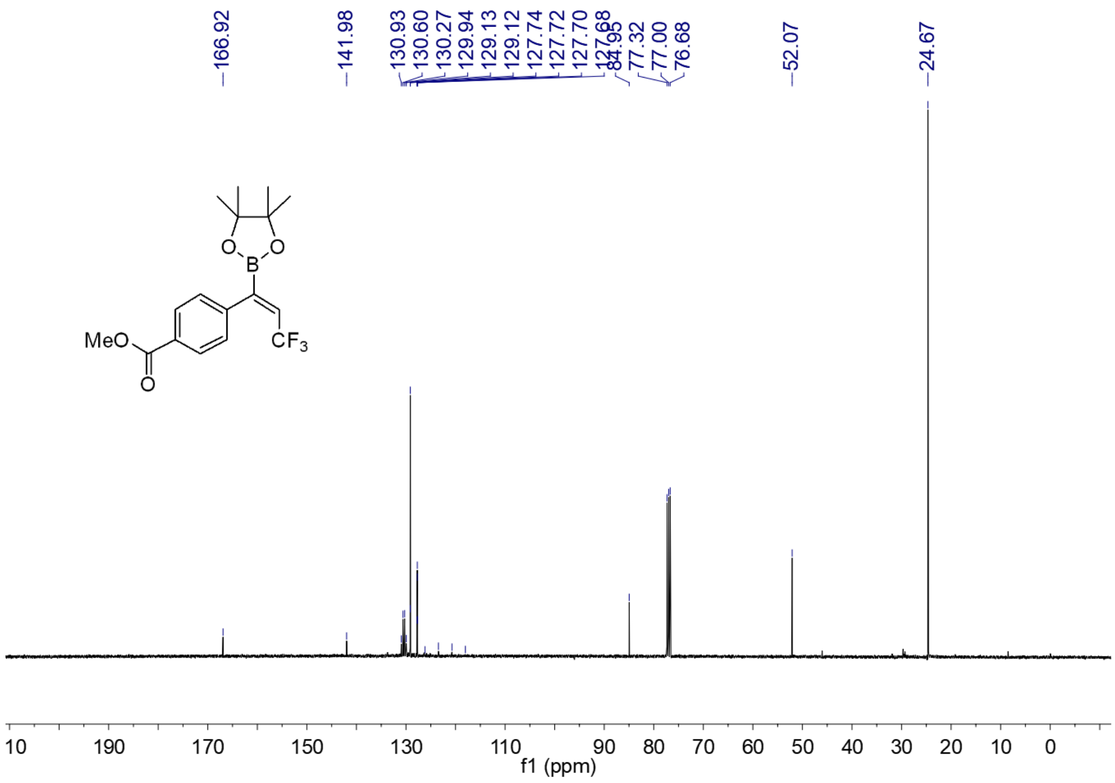

Supplementary Figure 72. ^{13}C NMR Spectra of product **3g**

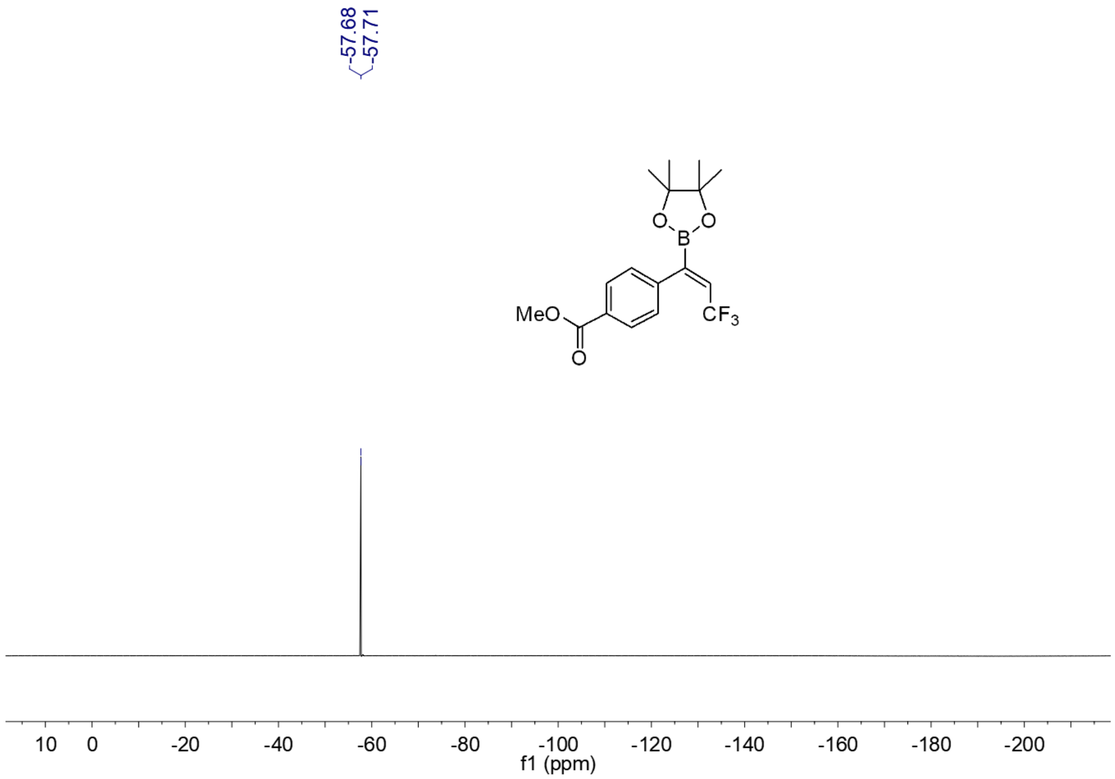

Supplementary Figure 73. ^{19}F NMR Spectra of product **3g**

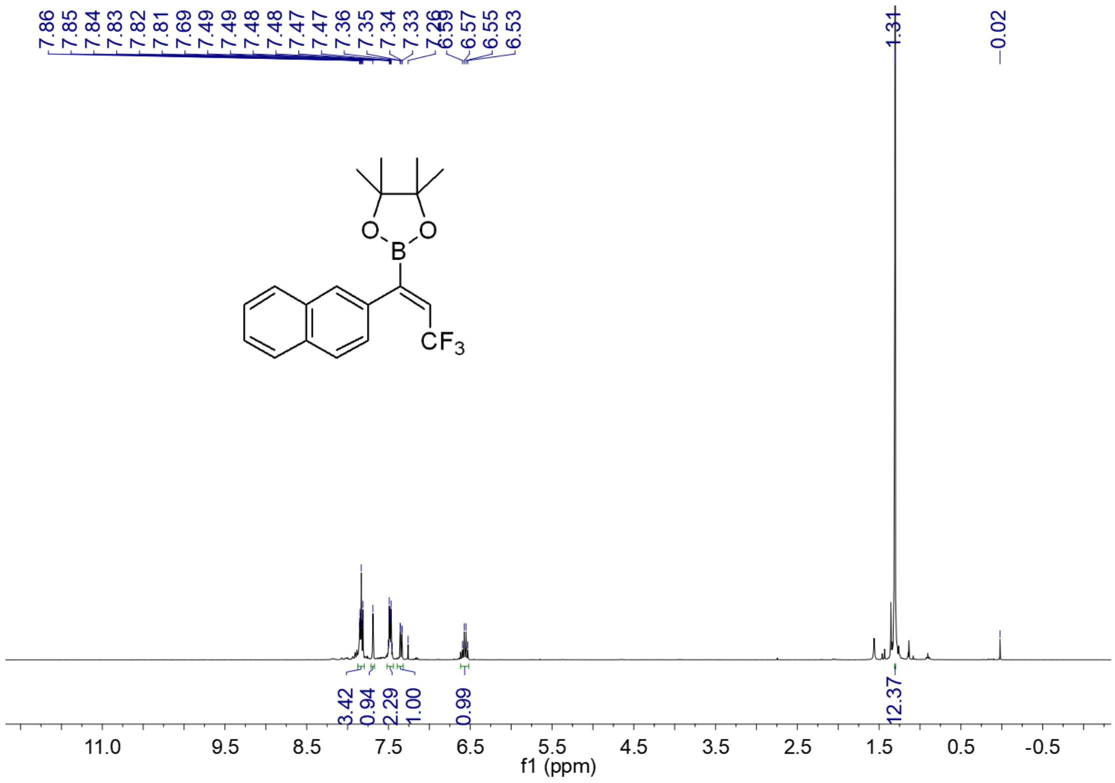

Supplementary Figure 74. ^{1}H NMR Spectra of product **3h**

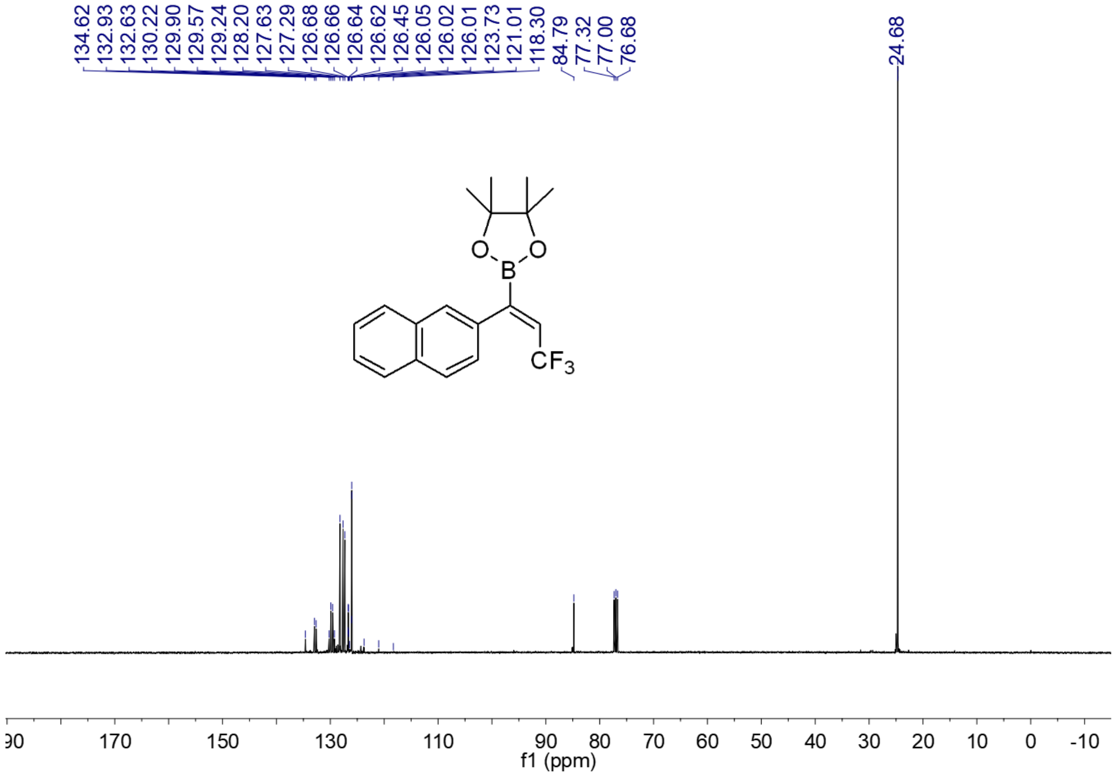

Supplementary Figure 75. ^{13}C NMR Spectra of product **3h**

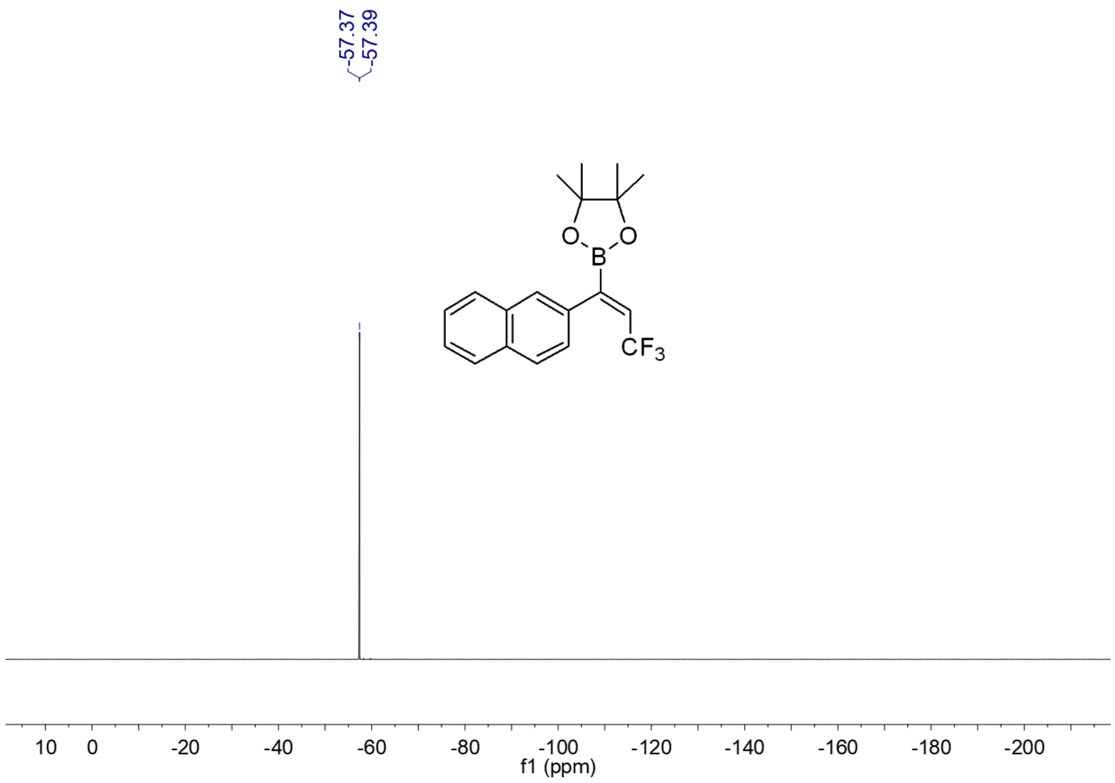

Supplementary Figure 76. ^{19}F NMR Spectra of product **3h**

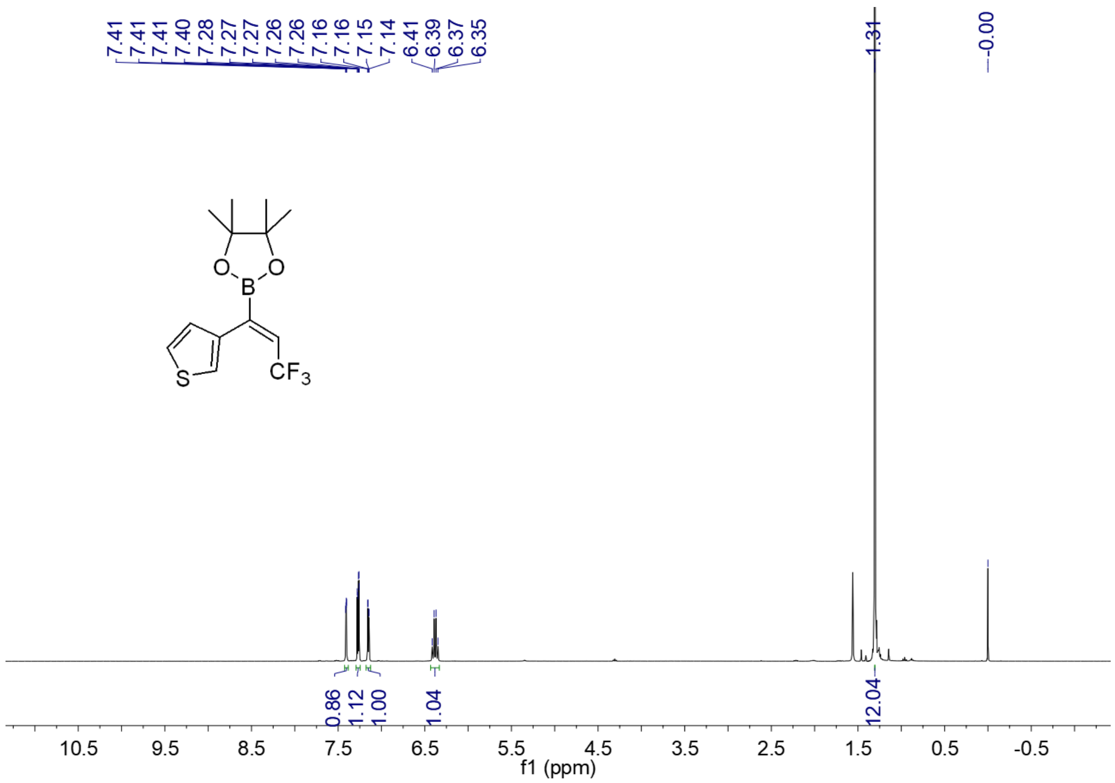

Supplementary Figure 77. ^{1}H NMR Spectra of product **3i**

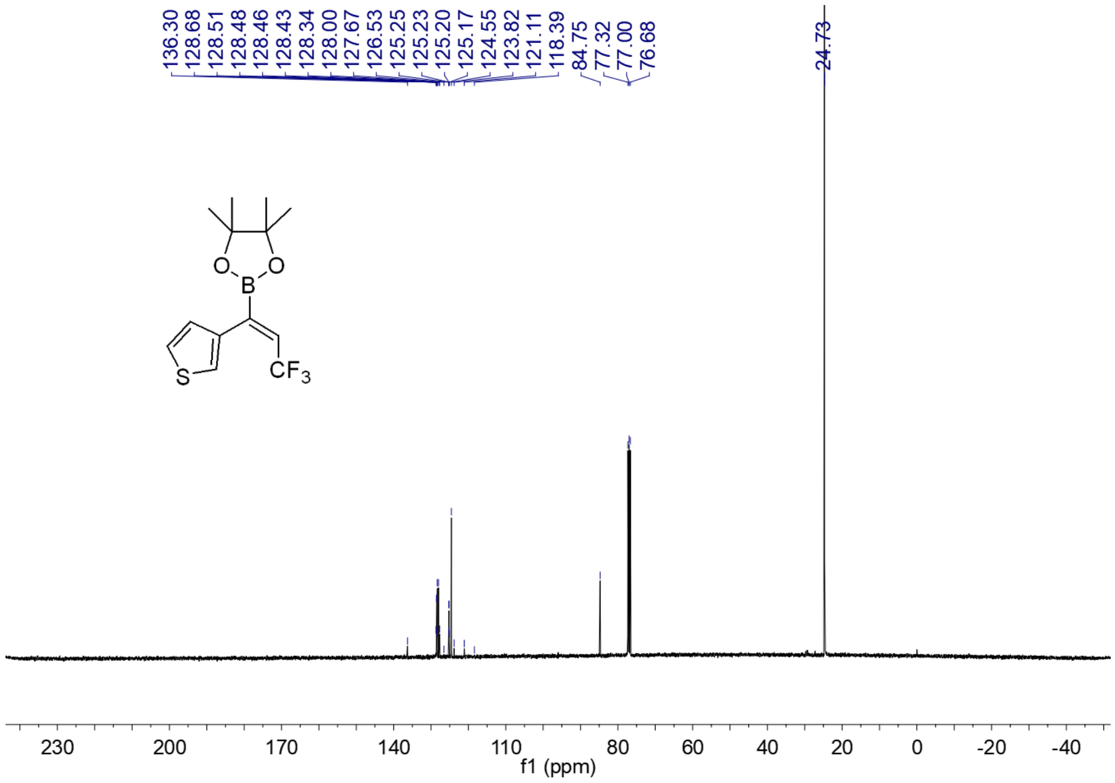

Supplementary Figure 78. ^{13}C NMR Spectra of product **3i**

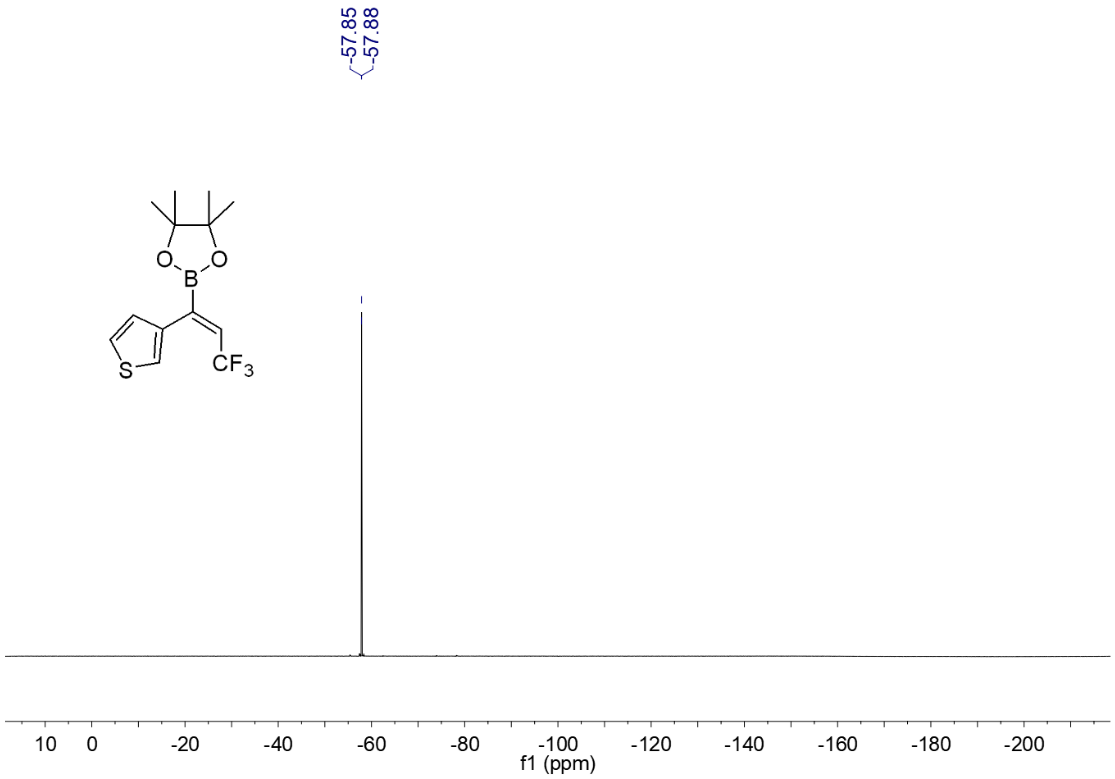

Supplementary Figure 79. ^{19}F NMR Spectra of product **3i**

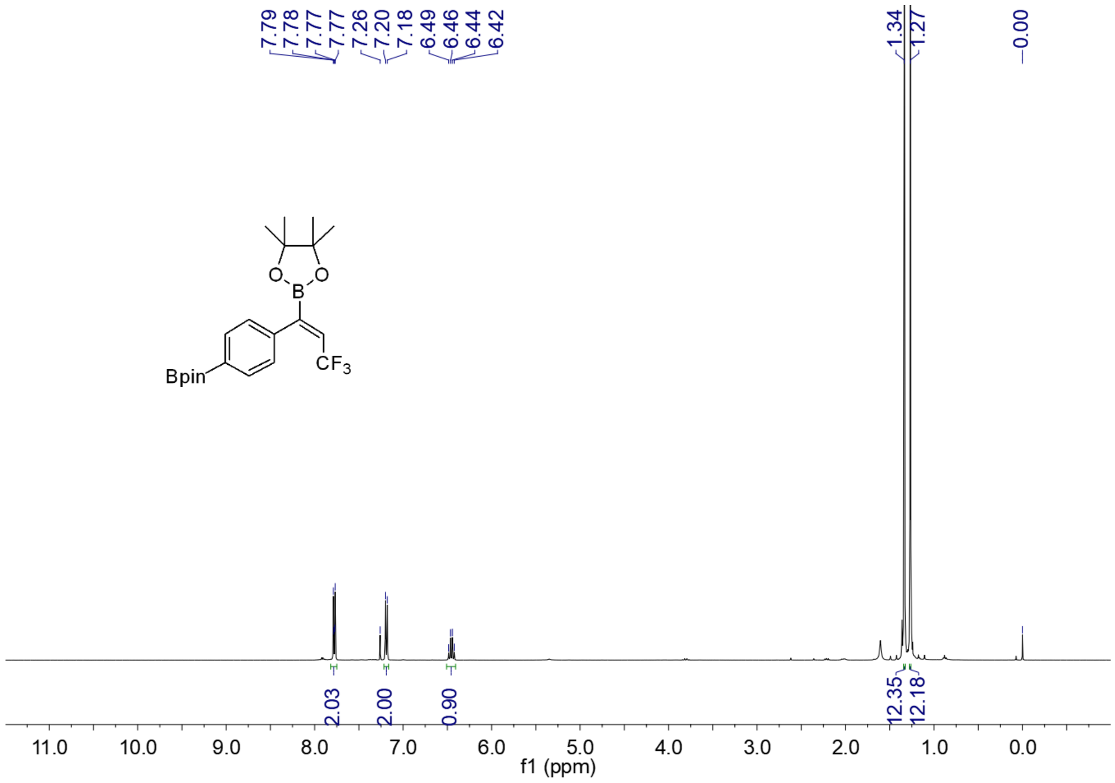

Supplementary Figure 80. ^{1}H NMR Spectra of product **3j**

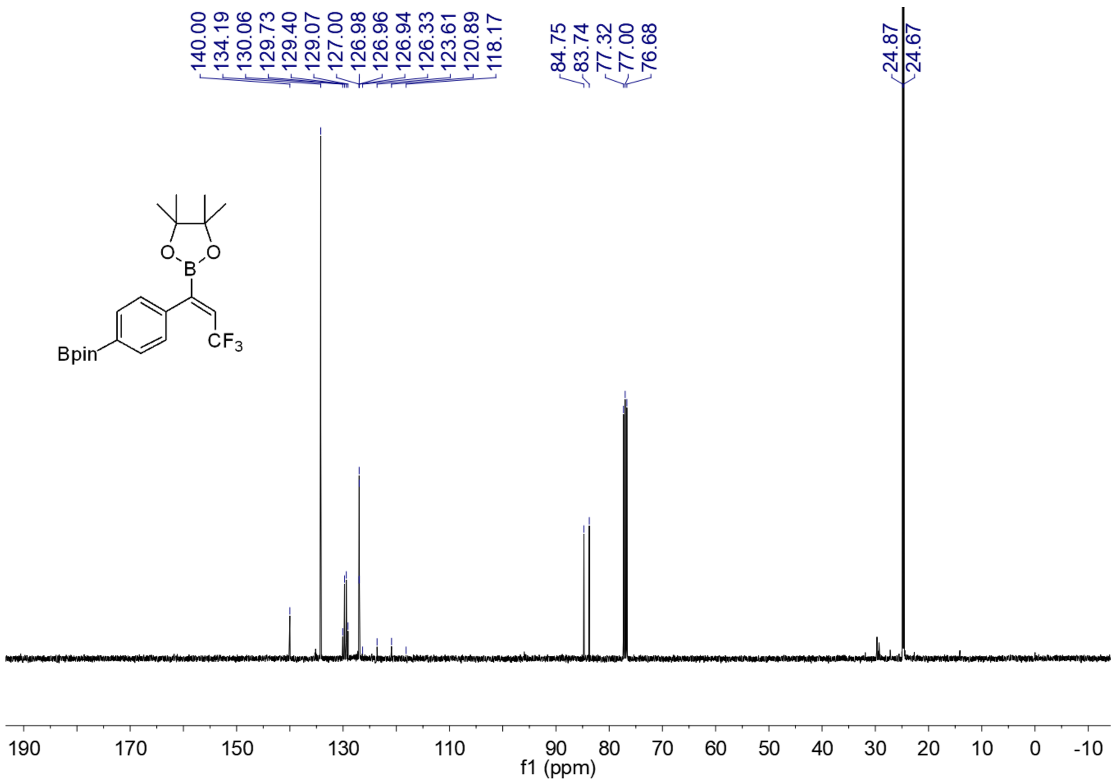

Supplementary Figure 81. ^{13}C NMR Spectra of product **3j**

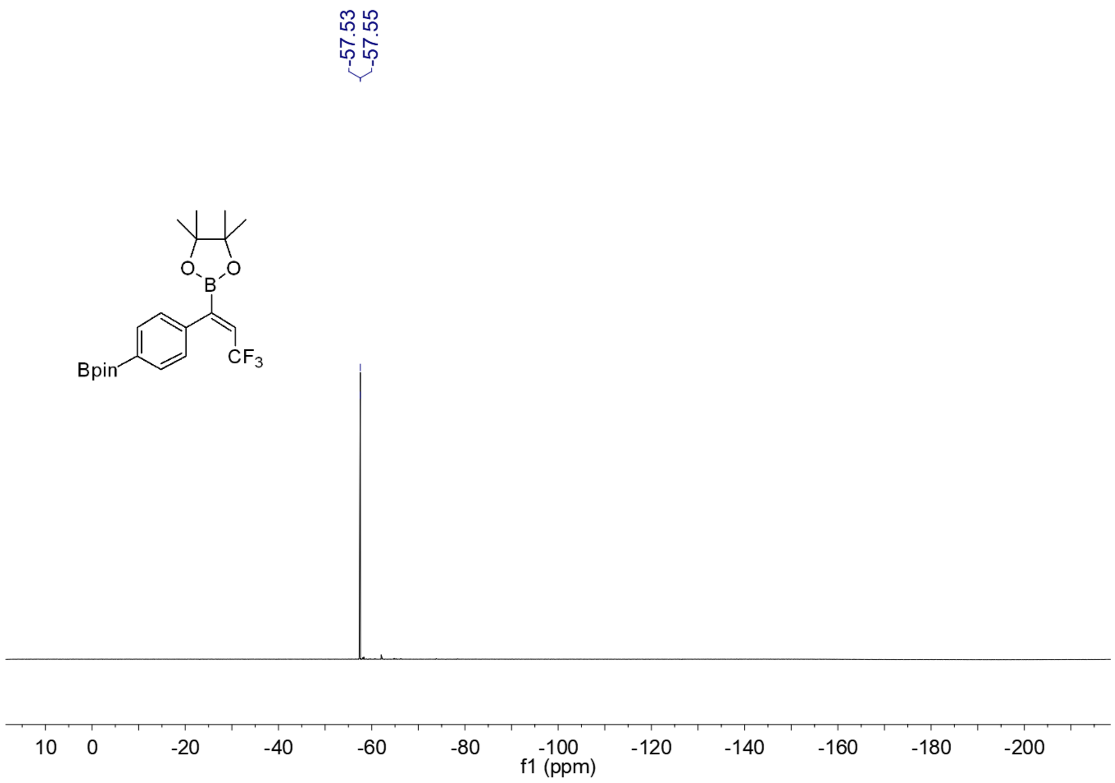

Supplementary Figure 82. ^{19}F NMR Spectra of product **3j**

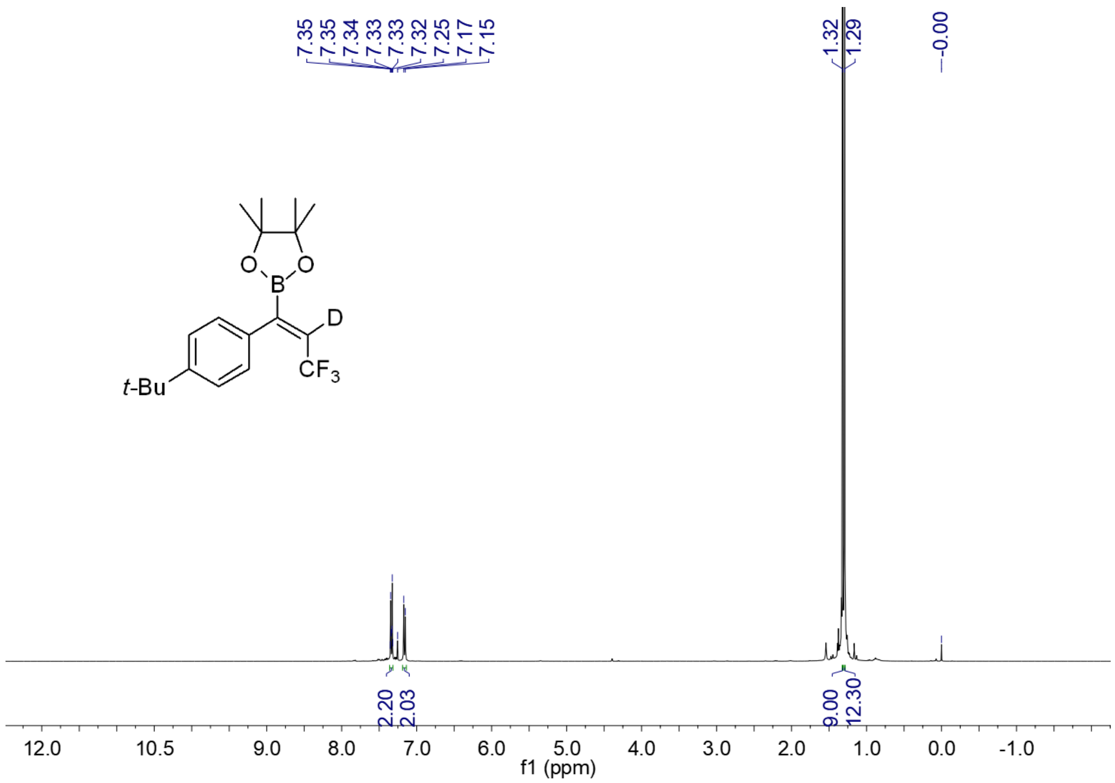

Supplementary Figure 83. ^{1}H NMR Spectra of product **3k**

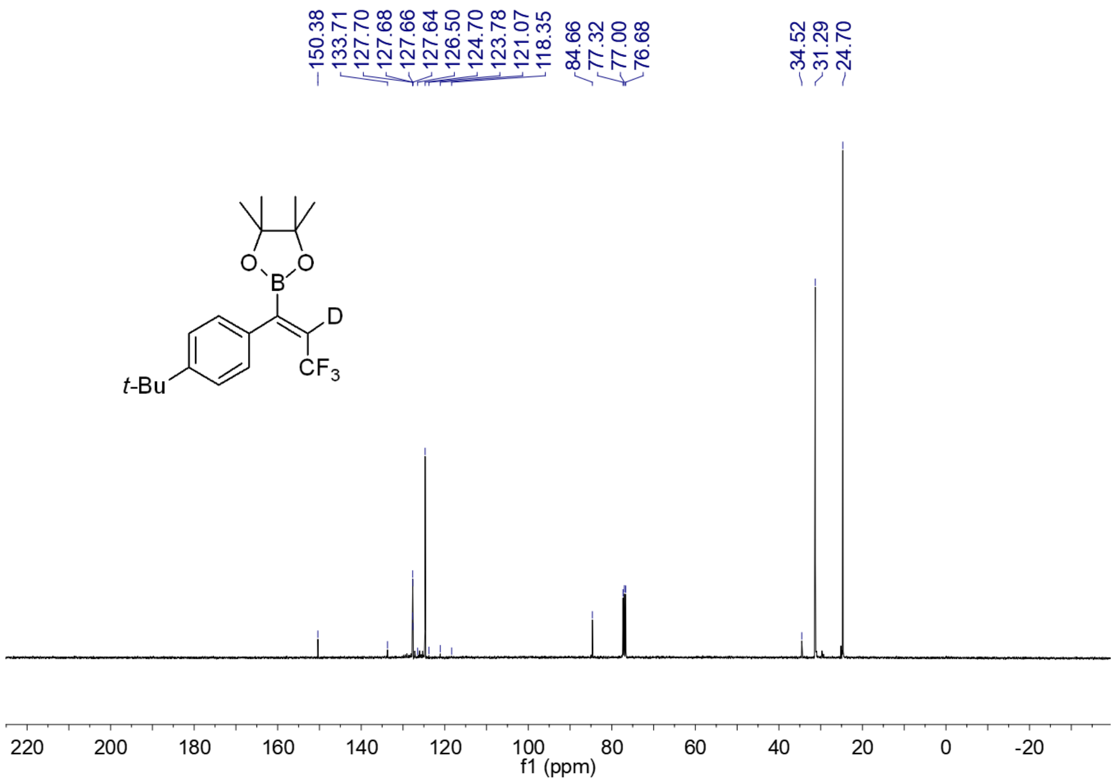

Supplementary Figure 84. ^{13}C NMR Spectra of product **3k**

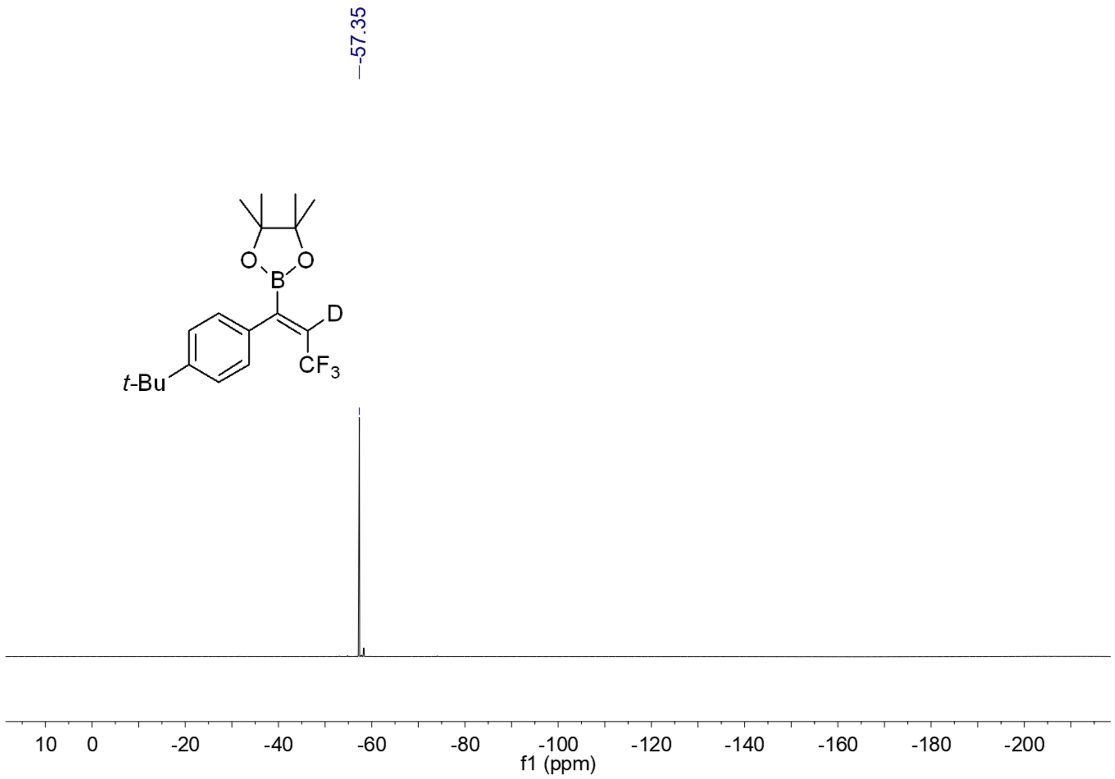

Supplementary Figure 85. ^{19}F NMR Spectra of product **3k**

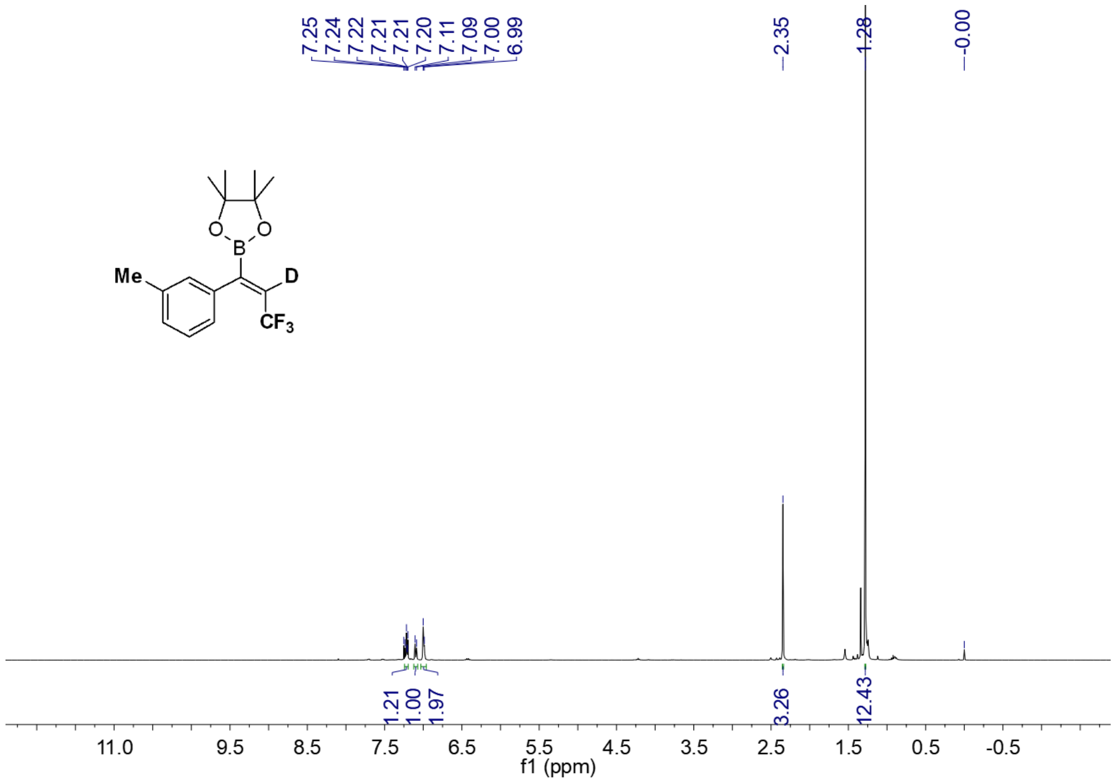

Supplementary Figure 86. ^{1}H NMR Spectra of product **3l**

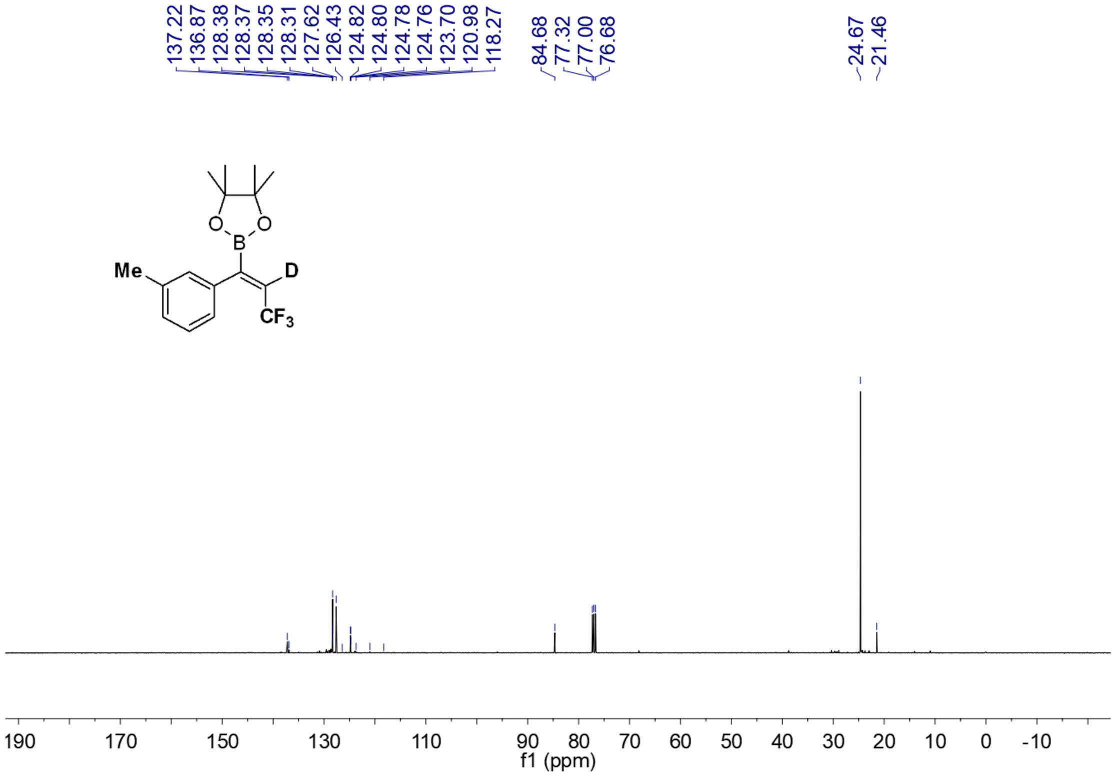

Supplementary Figure 87. ^{13}C NMR Spectra of product **3l**

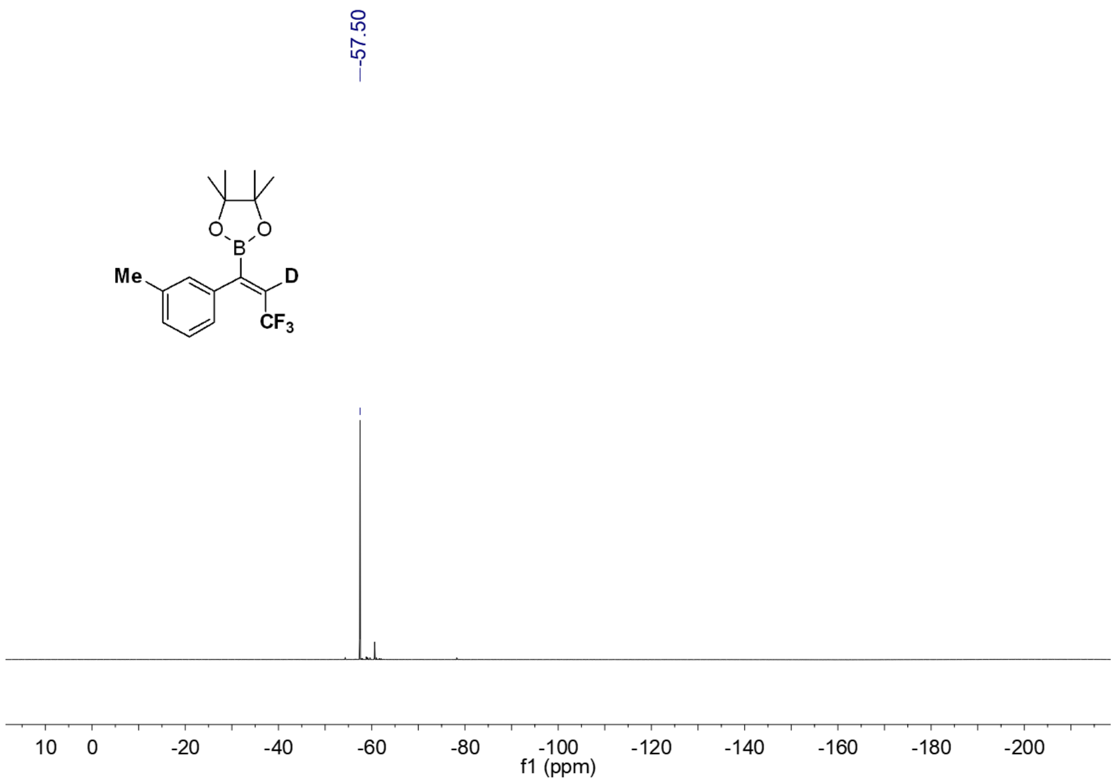

Supplementary Figure 88. ^{19}F NMR Spectra of product **3l**

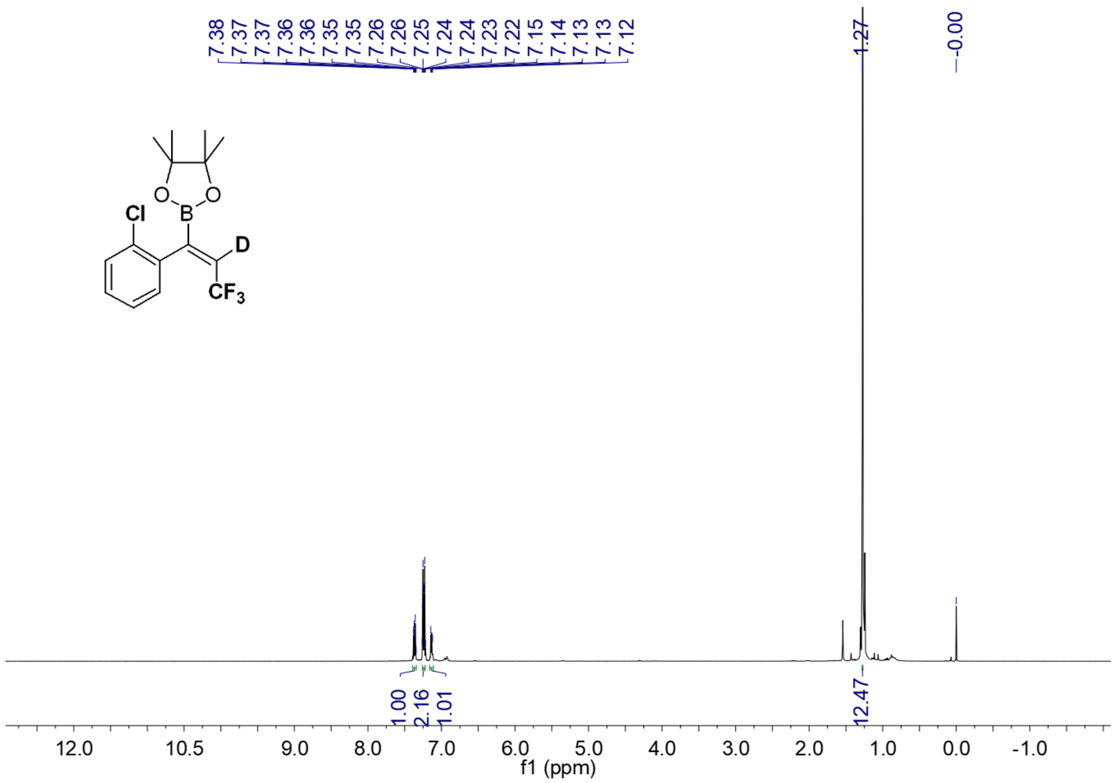

Supplementary Figure 89. ^{1}H NMR Spectra of product **3m**

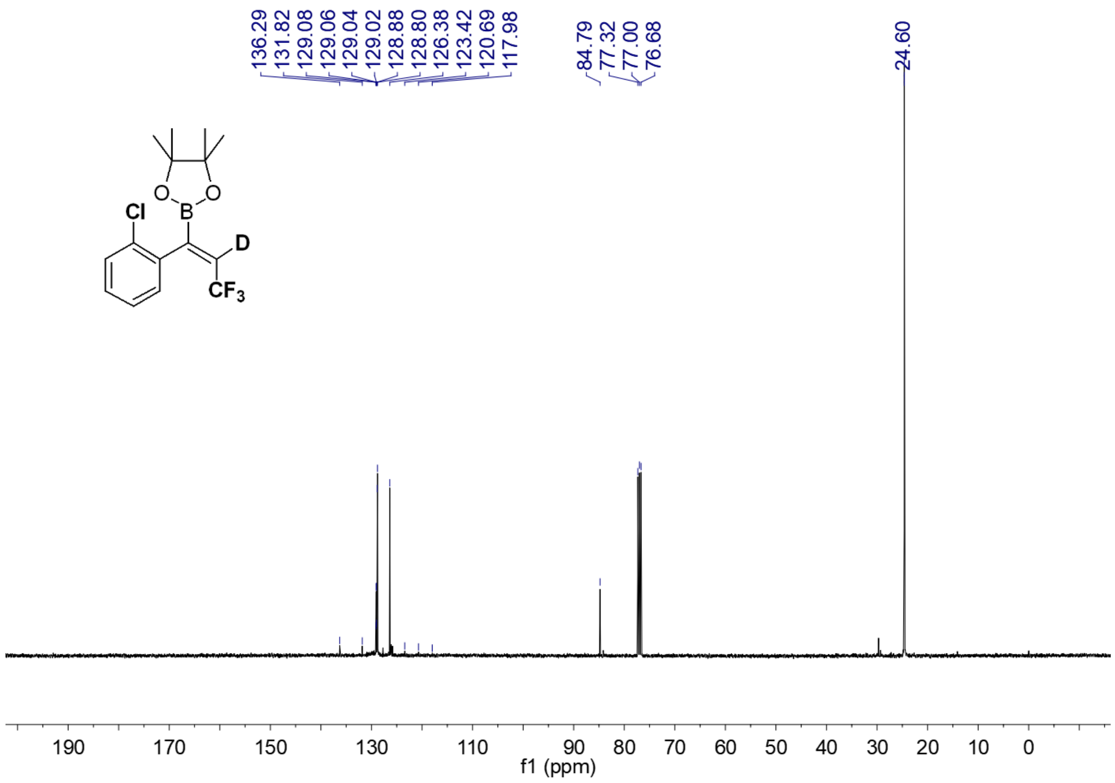

Supplementary Figure 90. ^{13}C NMR Spectra of product **3m**

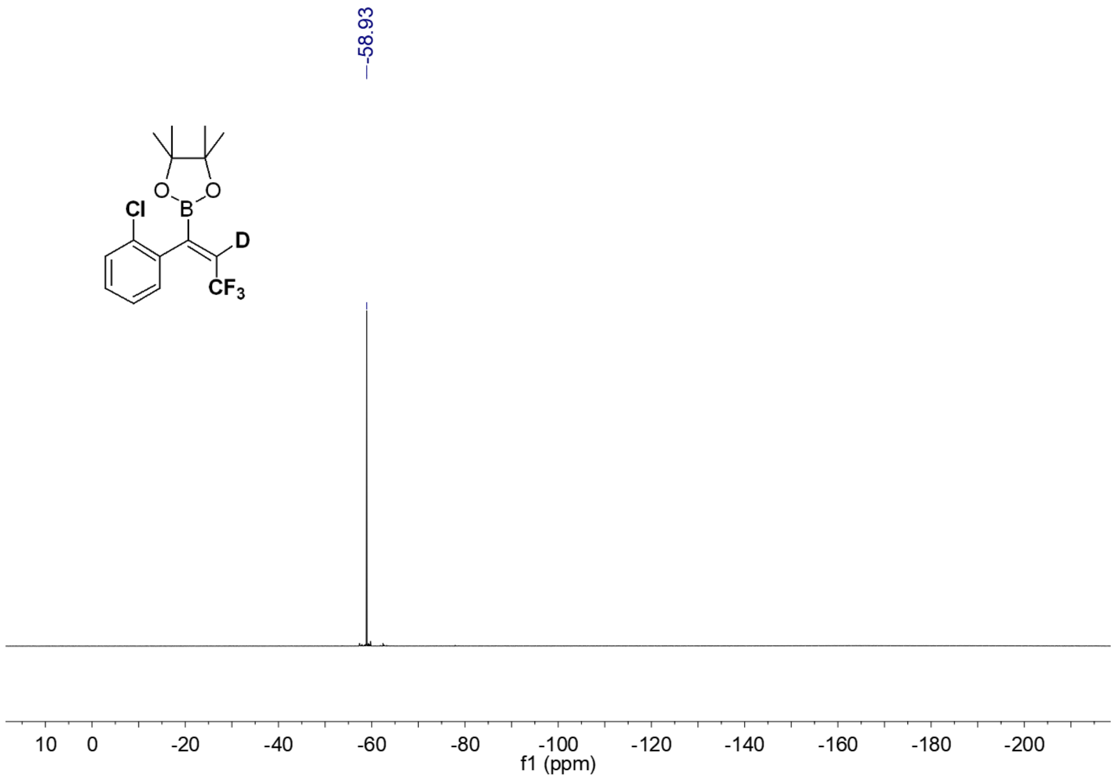

Supplementary Figure 91. ^{19}F NMR Spectra of product **3m**

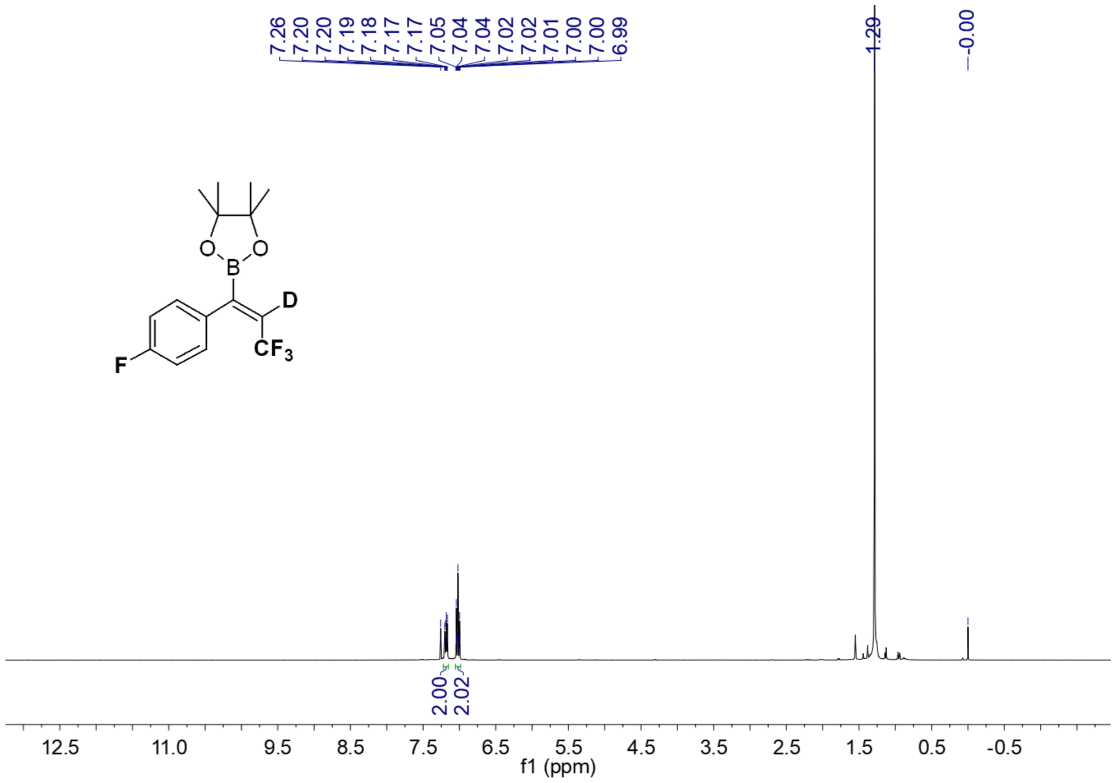

Supplementary Figure 92. ^{1}H NMR Spectra of product **3n**

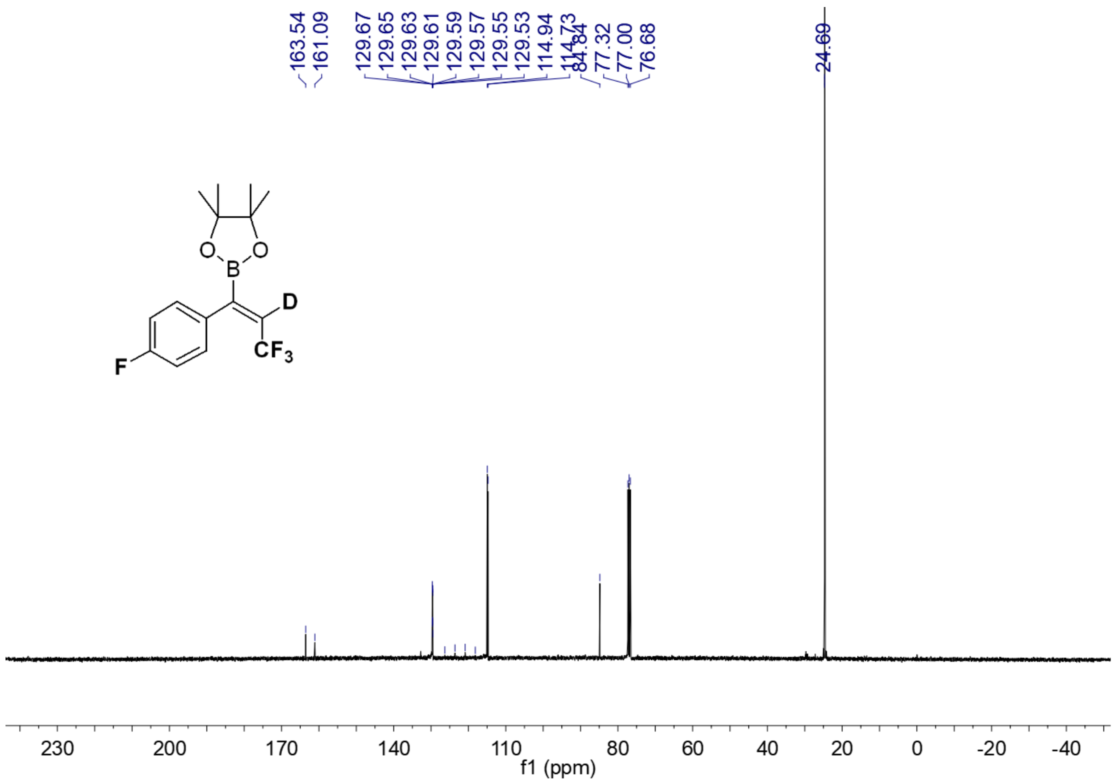

Supplementary Figure 93. ^{13}C NMR Spectra of product **3n**

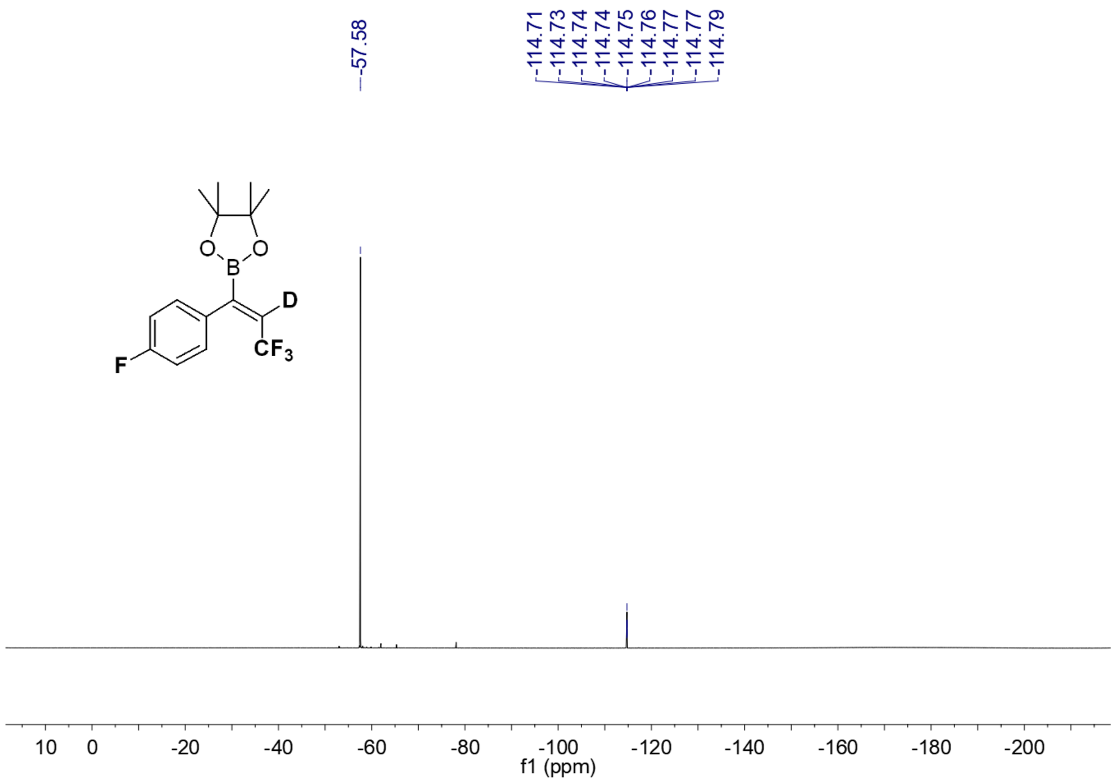

Supplementary Figure 94. ^{19}F NMR Spectra of product **3n**

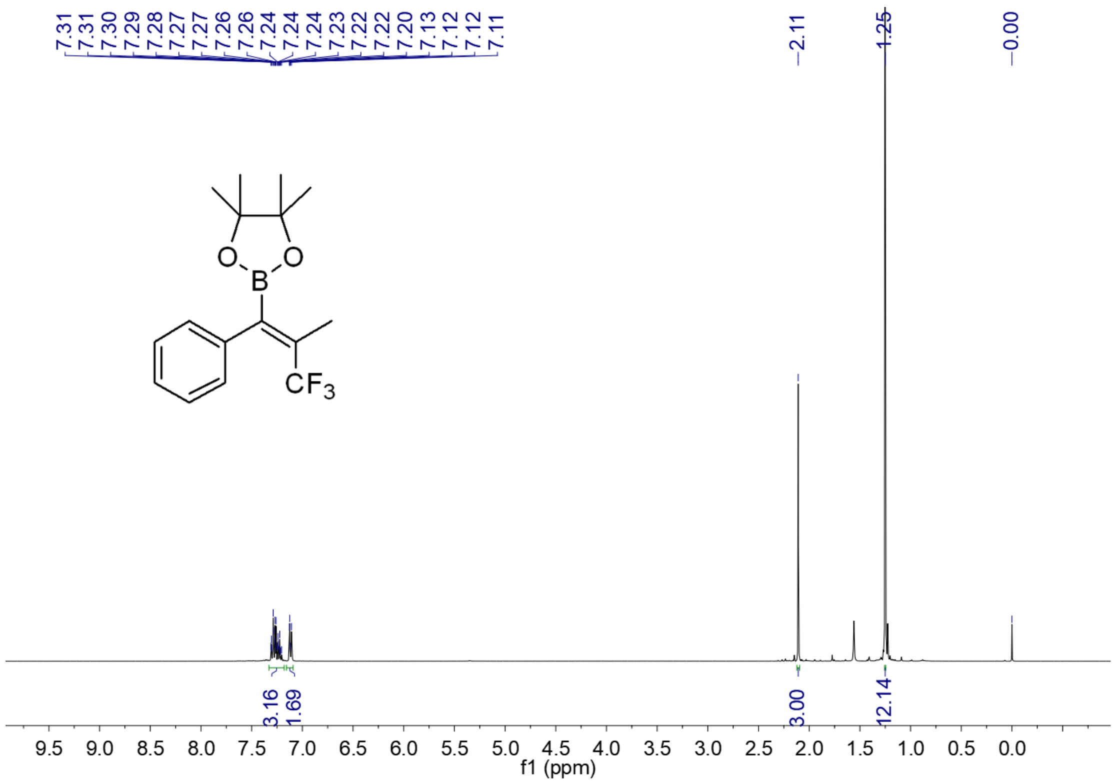

Supplementary Figure 95. ^{1}H NMR Spectra of product **3o**

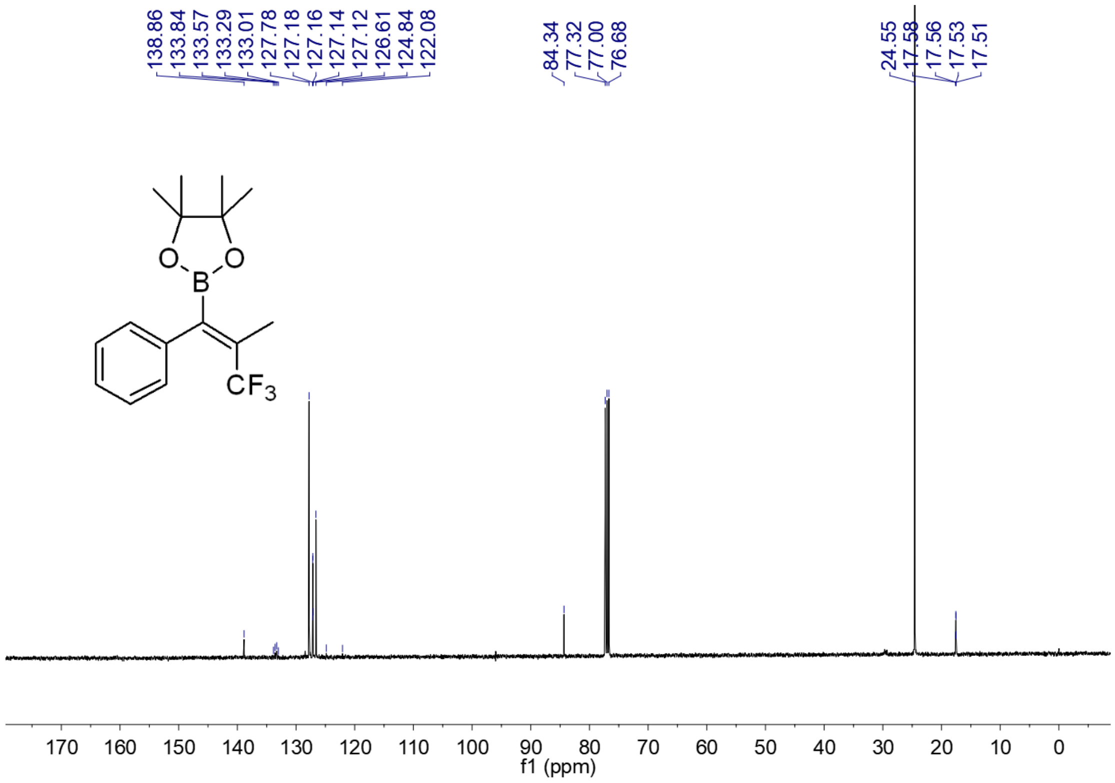

Supplementary Figure 96. ^{13}C NMR Spectra of product **3o**

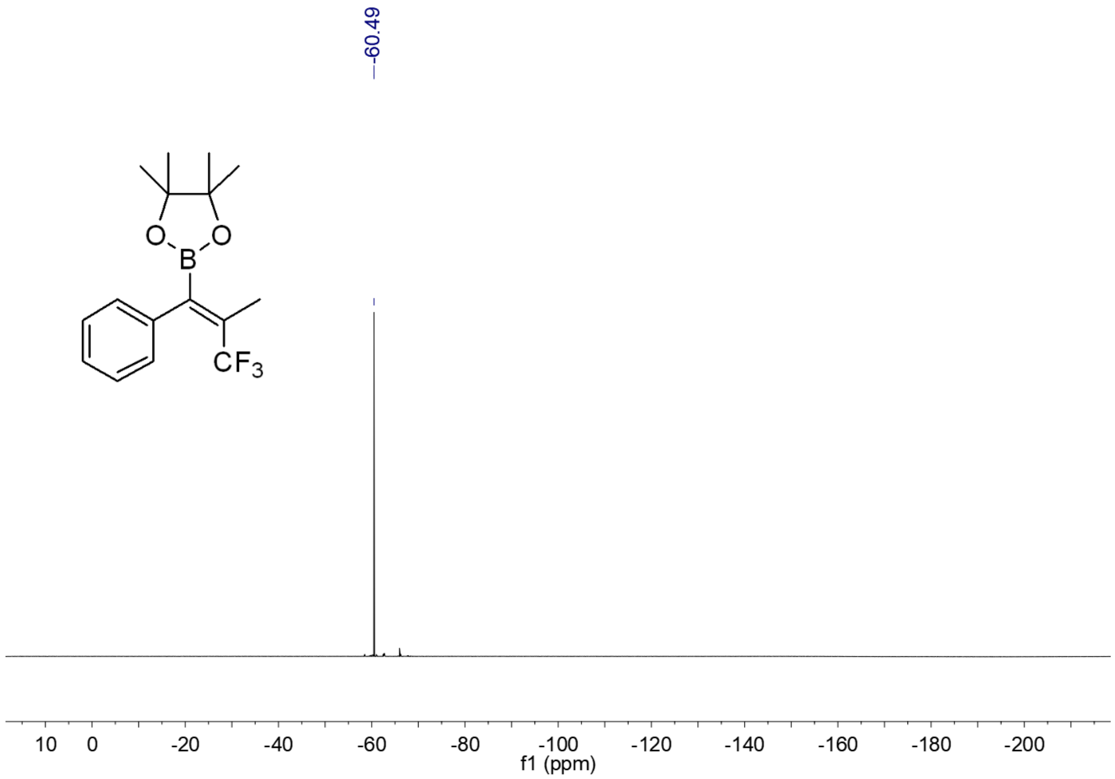

Supplementary Figure 97. ^{19}F NMR Spectra of product **3o**

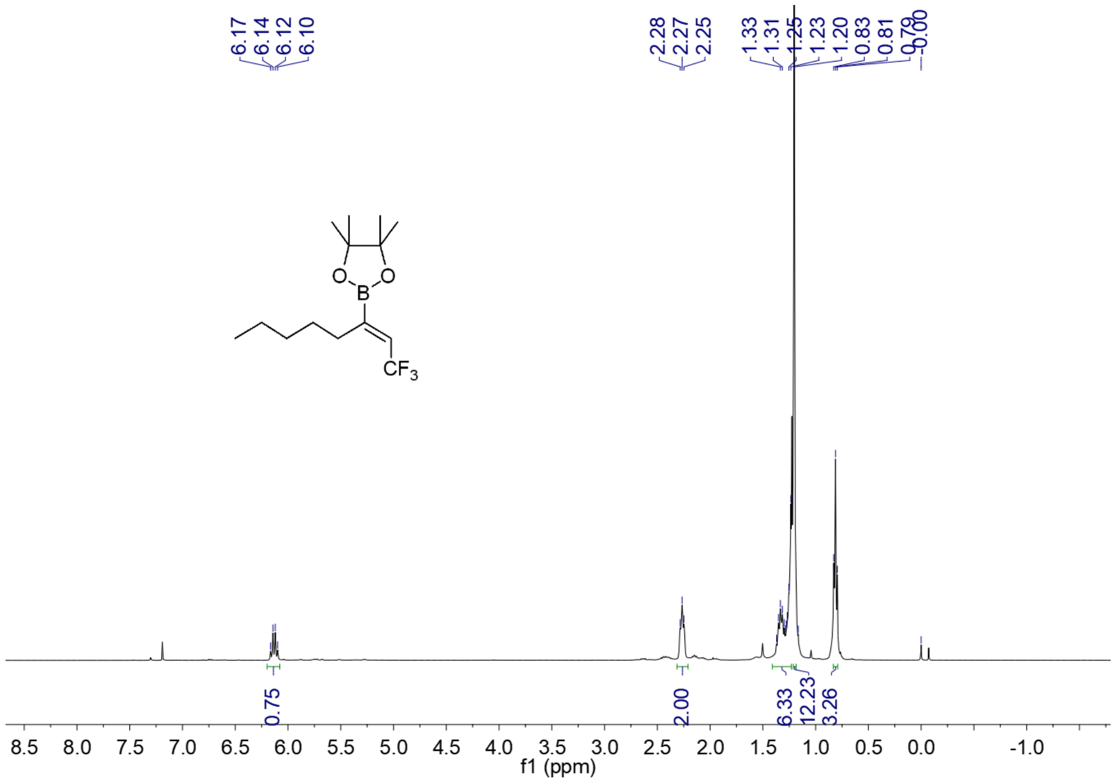

Supplementary Figure 98. ^{1}H NMR Spectra of product **3p**

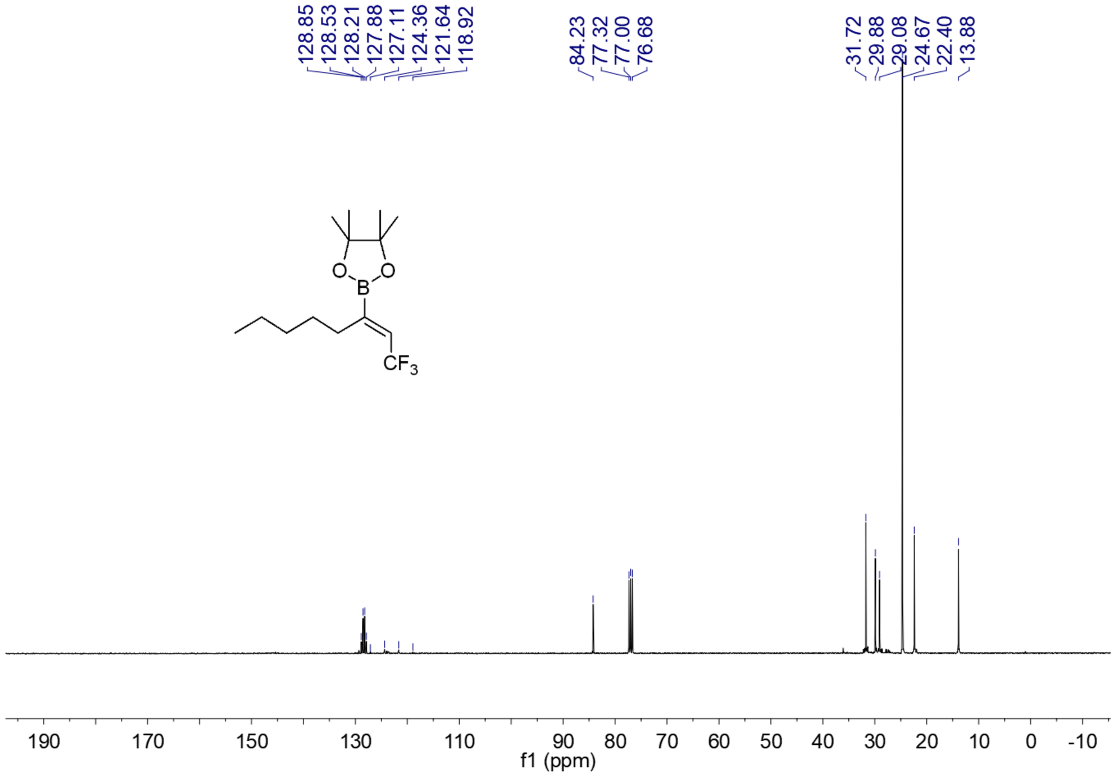

Supplementary Figure 99. ^{13}C NMR Spectra of product **3p**

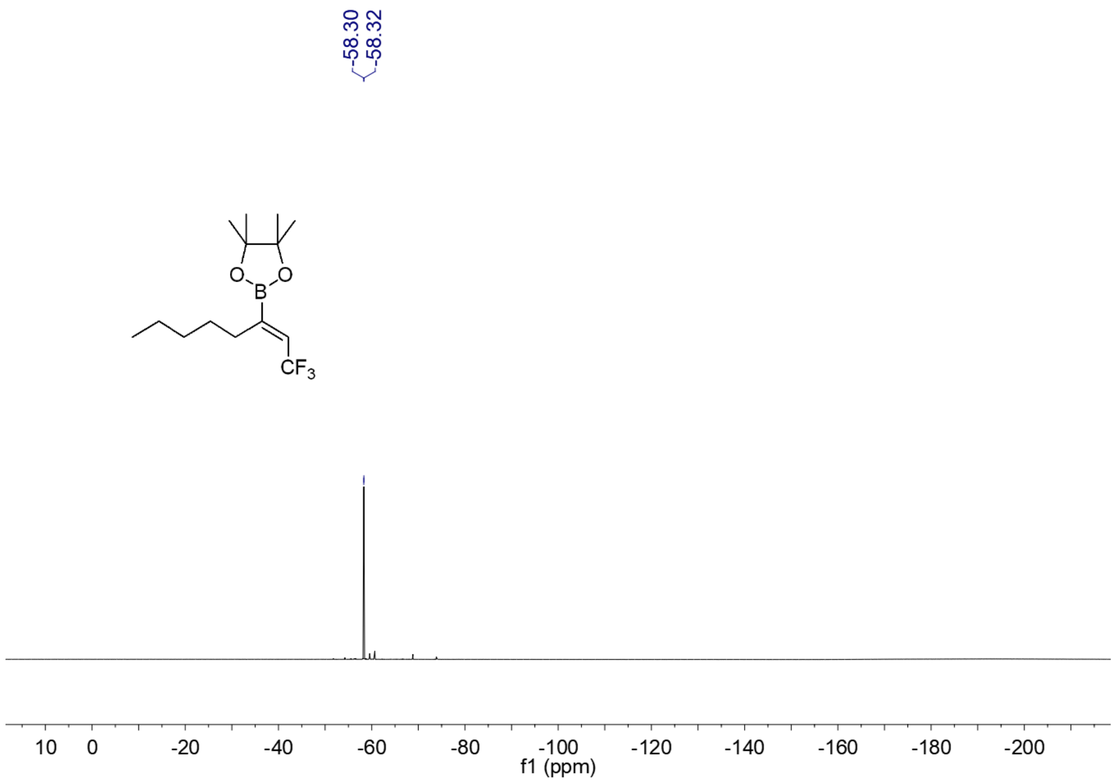

Supplementary Figure 100. ^{19}F NMR Spectra of product **3p**

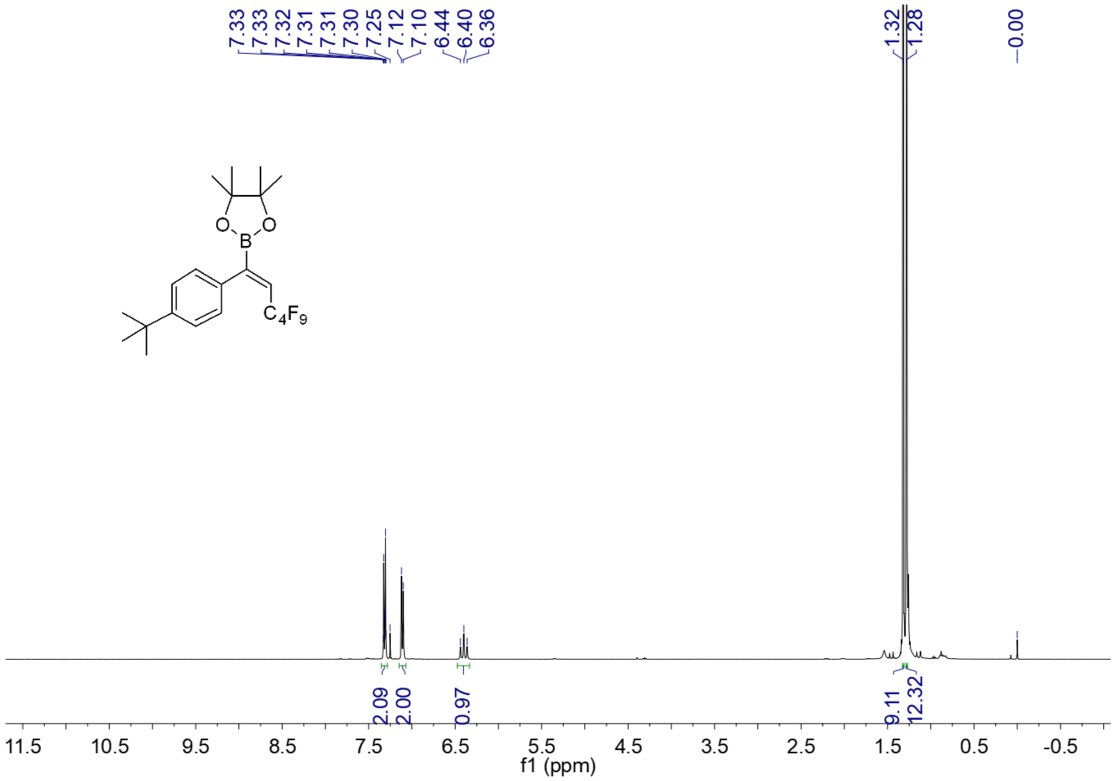

Supplementary Figure 101. ^{1}H NMR Spectra of product **3q**

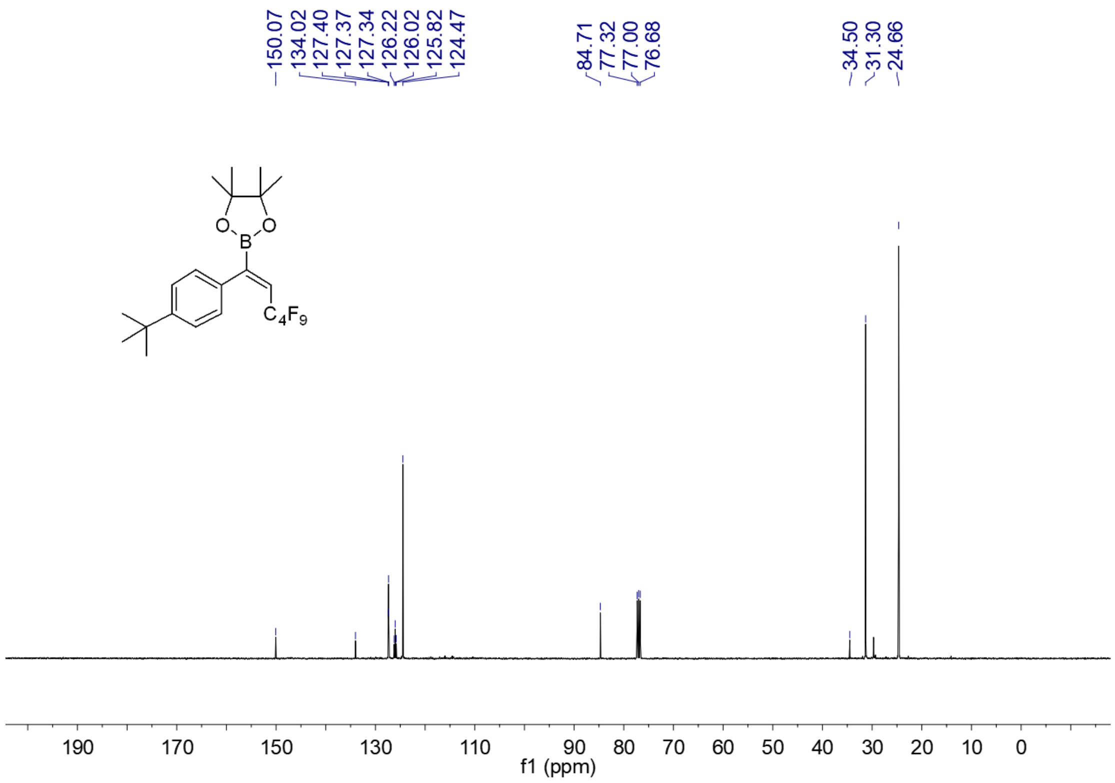

Supplementary Figure 102. ^{13}C NMR Spectra of product **3q**

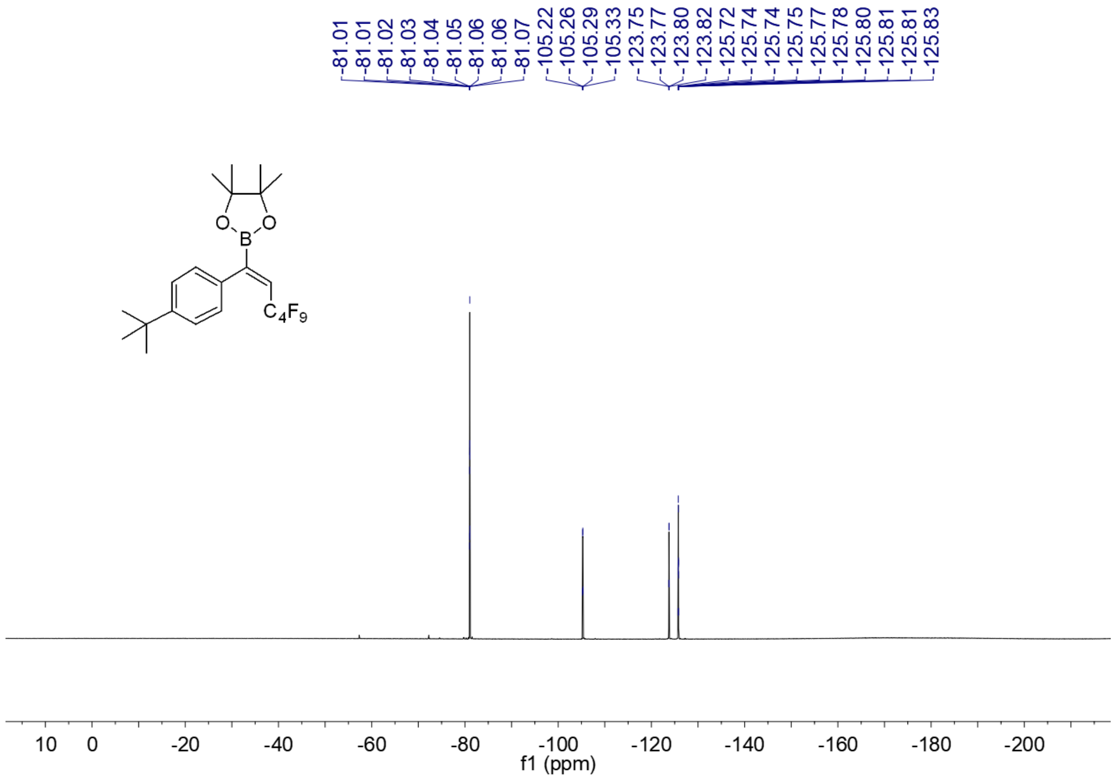

Supplementary Figure 103. ^{19}F NMR Spectra of product **3q**

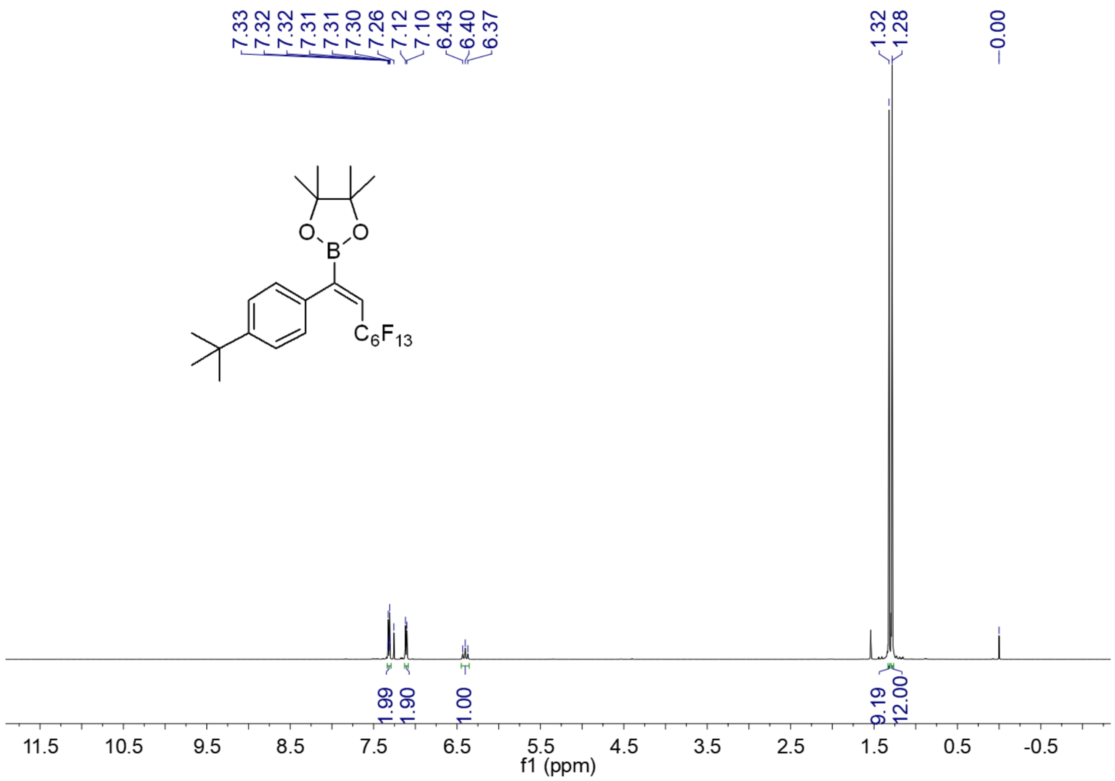

Supplementary Figure 104. ^{1}H NMR Spectra of product **3r**

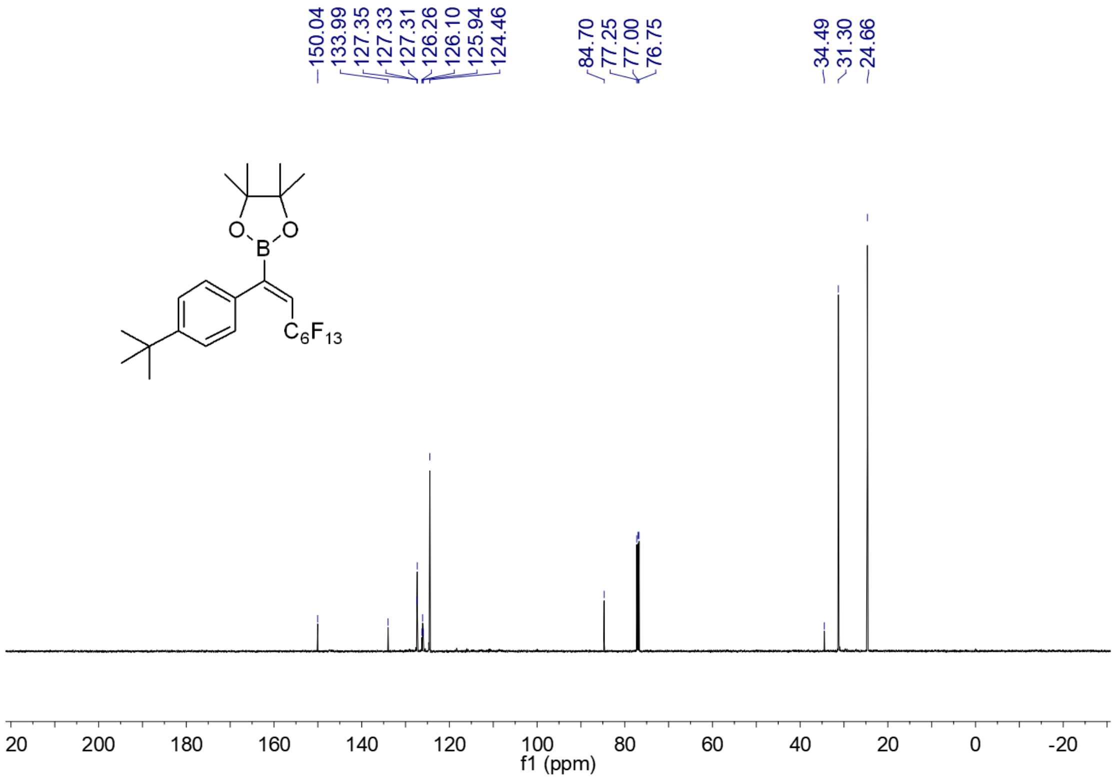

Supplementary Figure 105. ^{13}C NMR Spectra of product **3r**

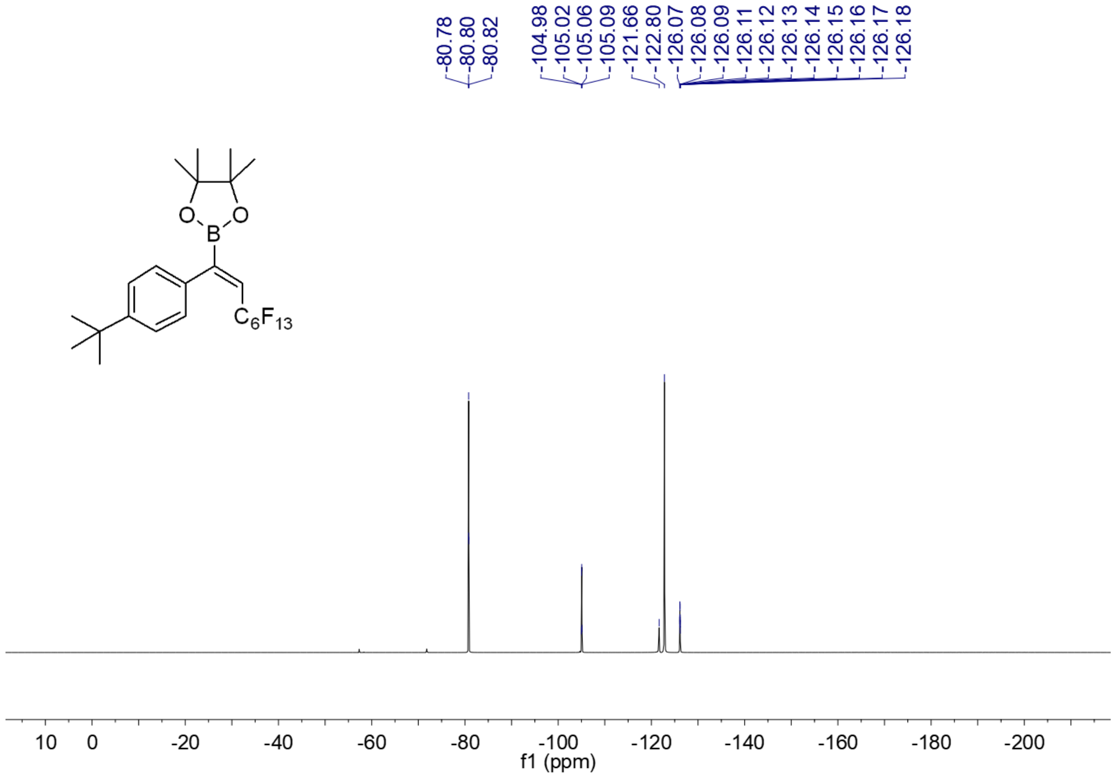

Supplementary Figure 106. ^{19}F NMR Spectra of product **3r**

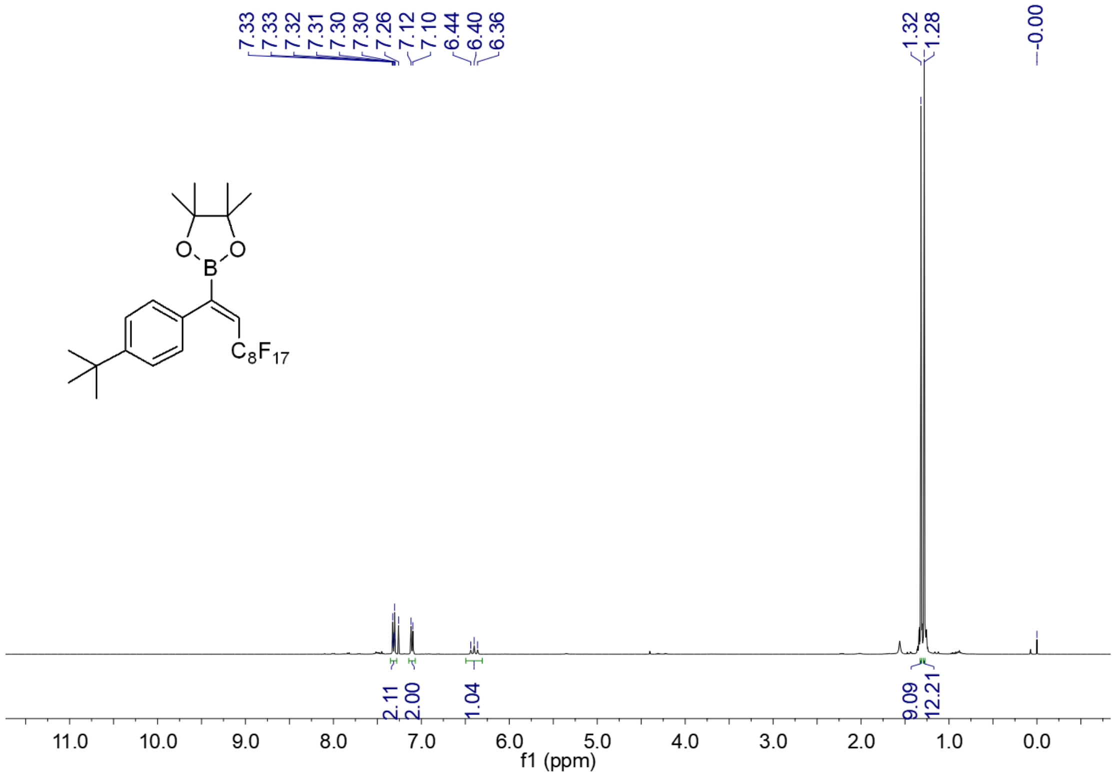

Supplementary Figure 107. ^{1}H NMR Spectra of product **3s**

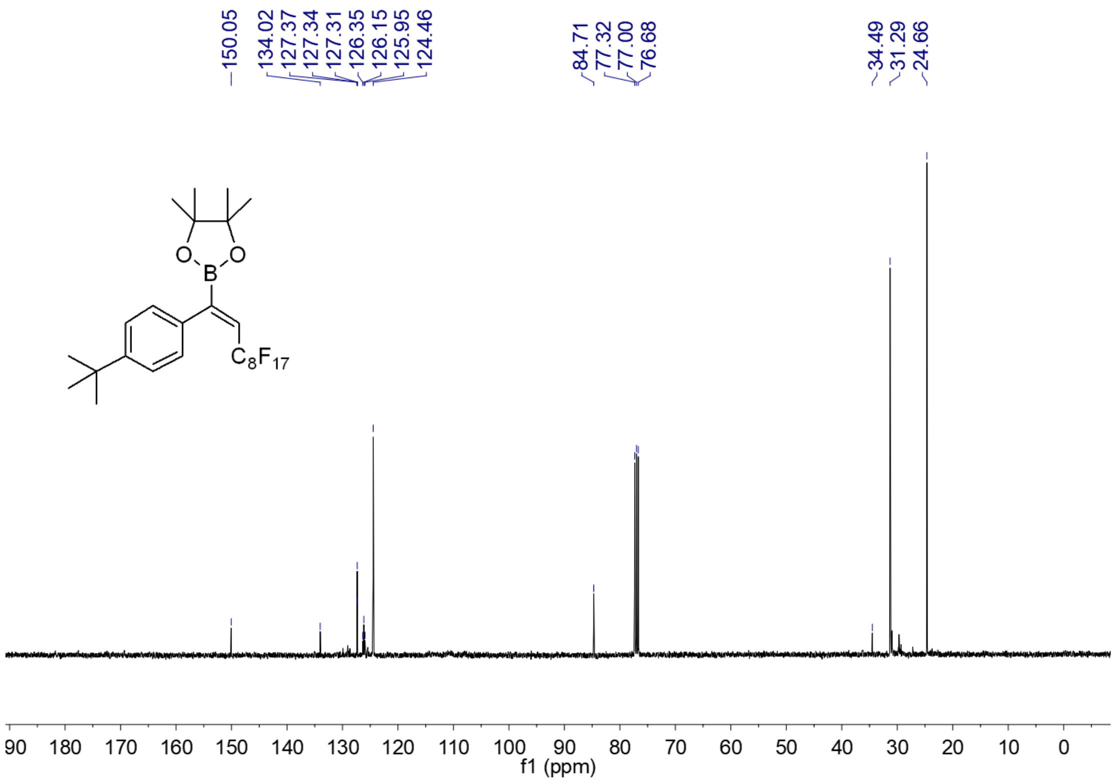

Supplementary Figure 108. ^{13}C NMR Spectra of product **3s**

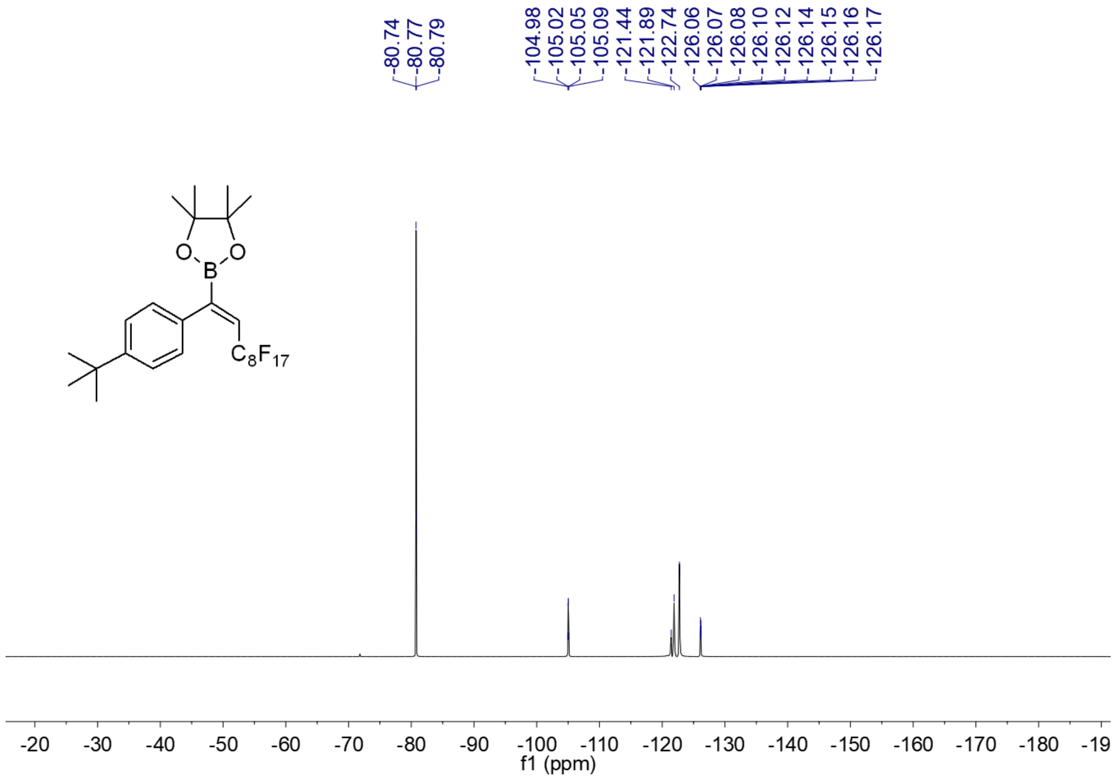

Supplementary Figure 109. ^{19}F NMR Spectra of product **3s**

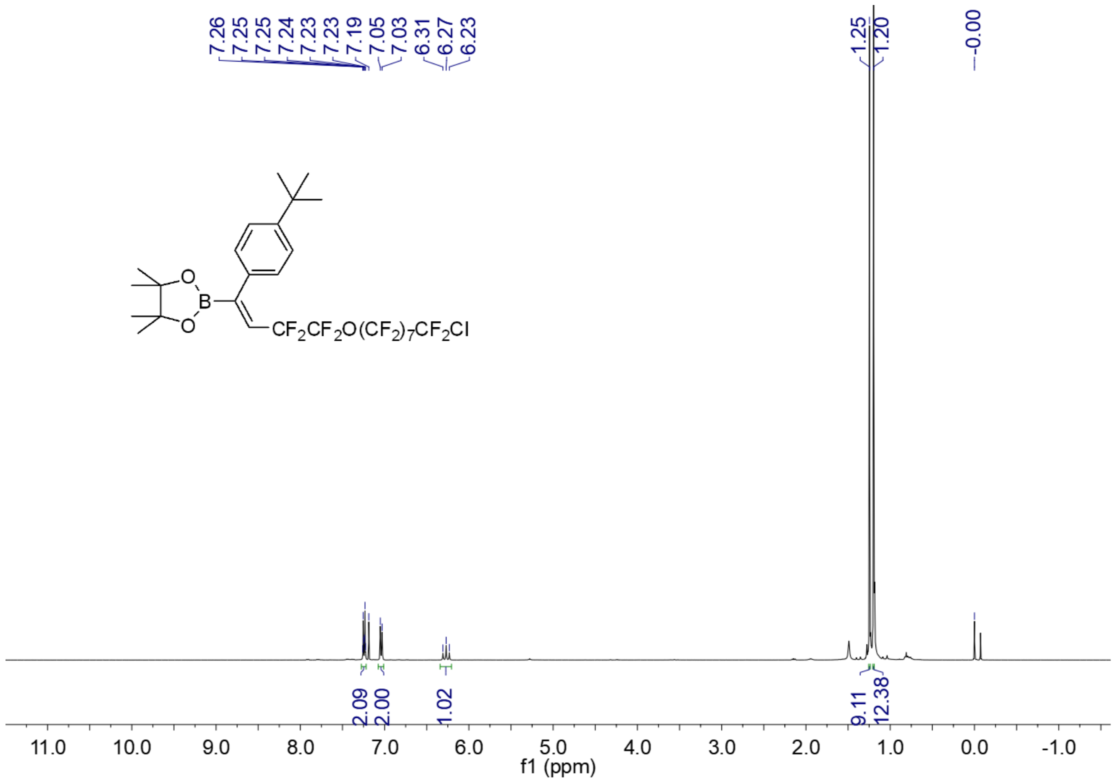

Supplementary Figure 110. ^{1}H NMR Spectra of product **3t**

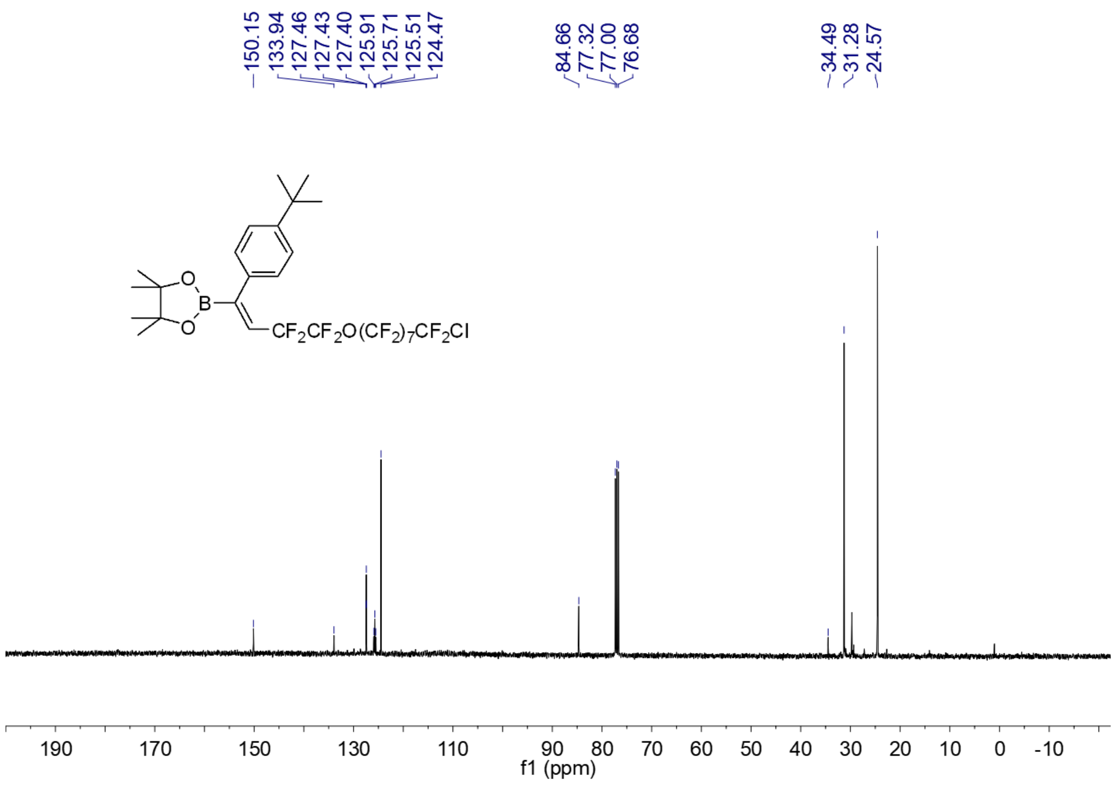

Supplementary Figure 111. ^{13}C NMR Spectra of product **3t**

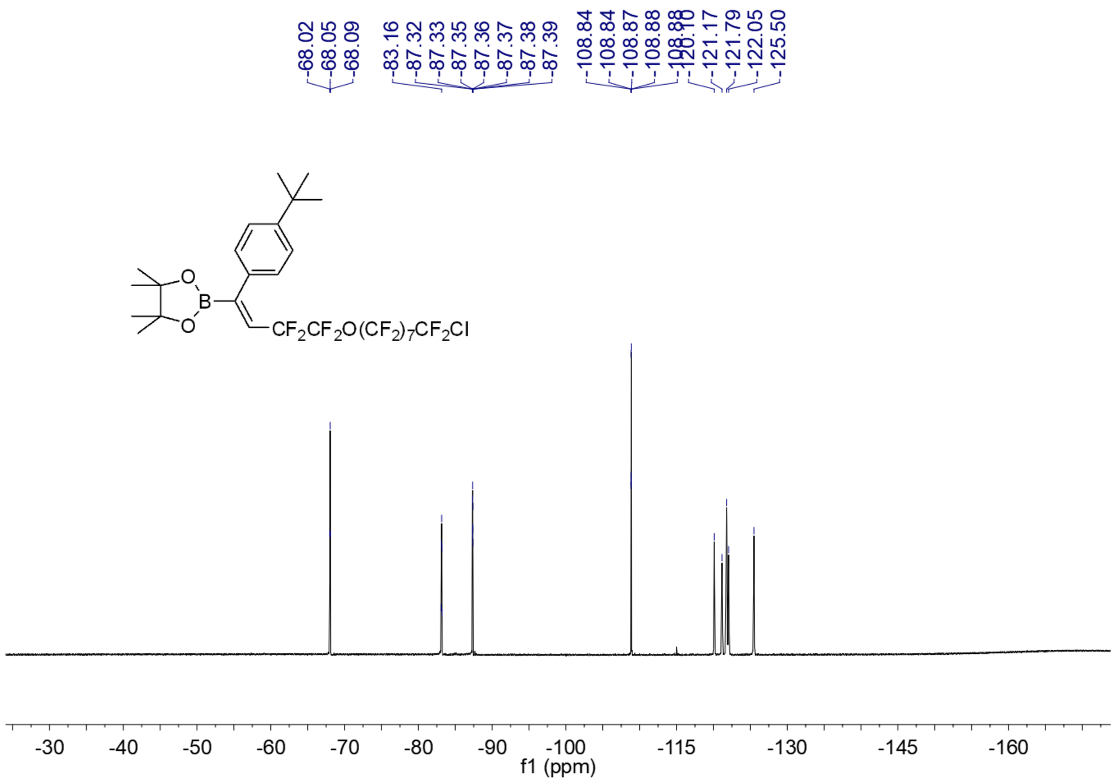

Supplementary Figure 112. ^{19}F NMR Spectra of product **3t**

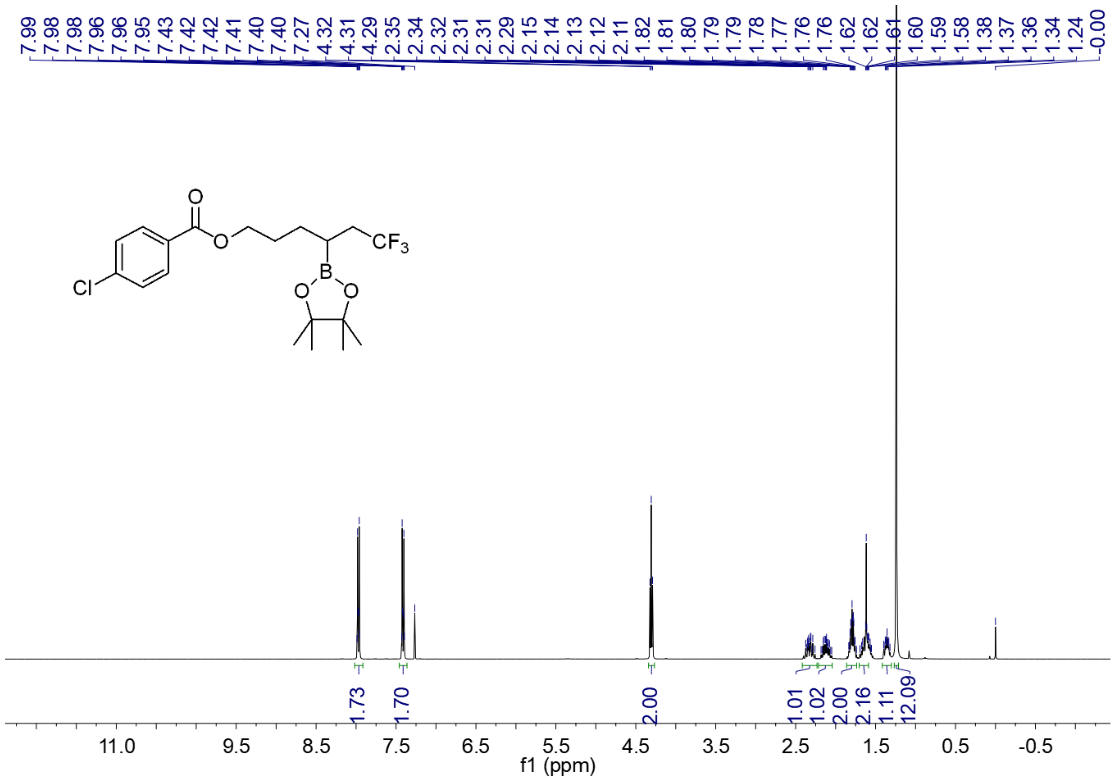

Supplementary Figure 113. ^{1}H NMR Spectra of product **5a**

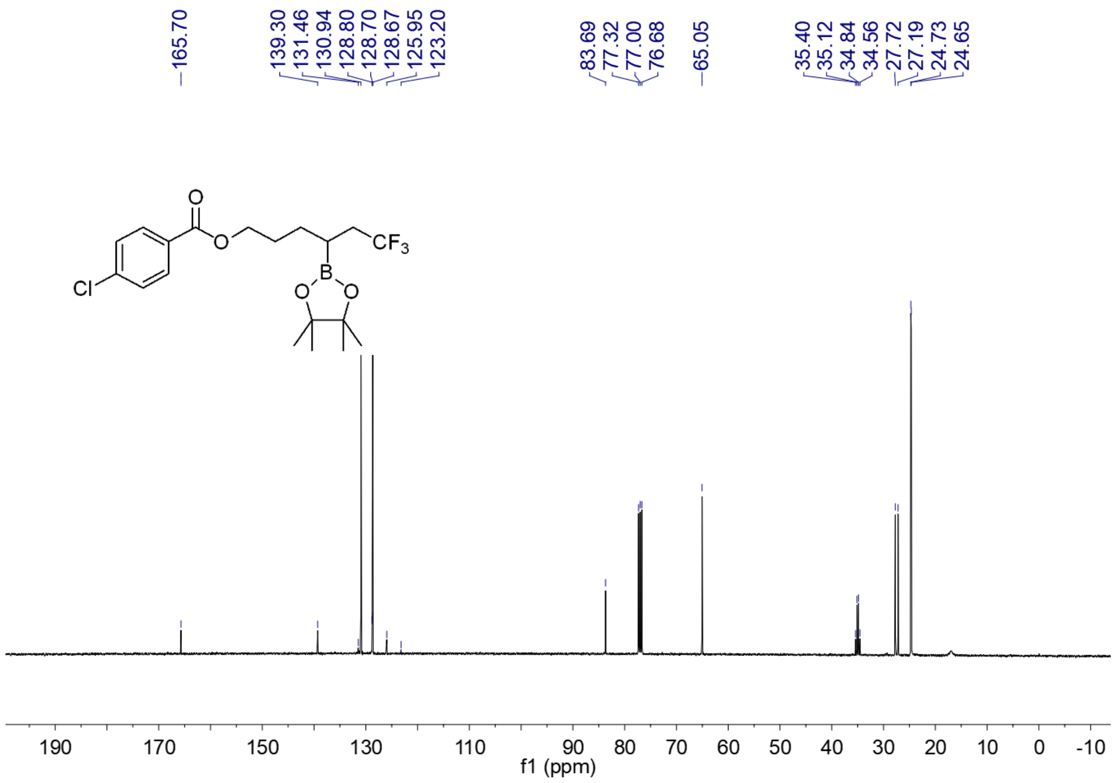

Supplementary Figure 114. ^{13}C NMR Spectra of product **5a**

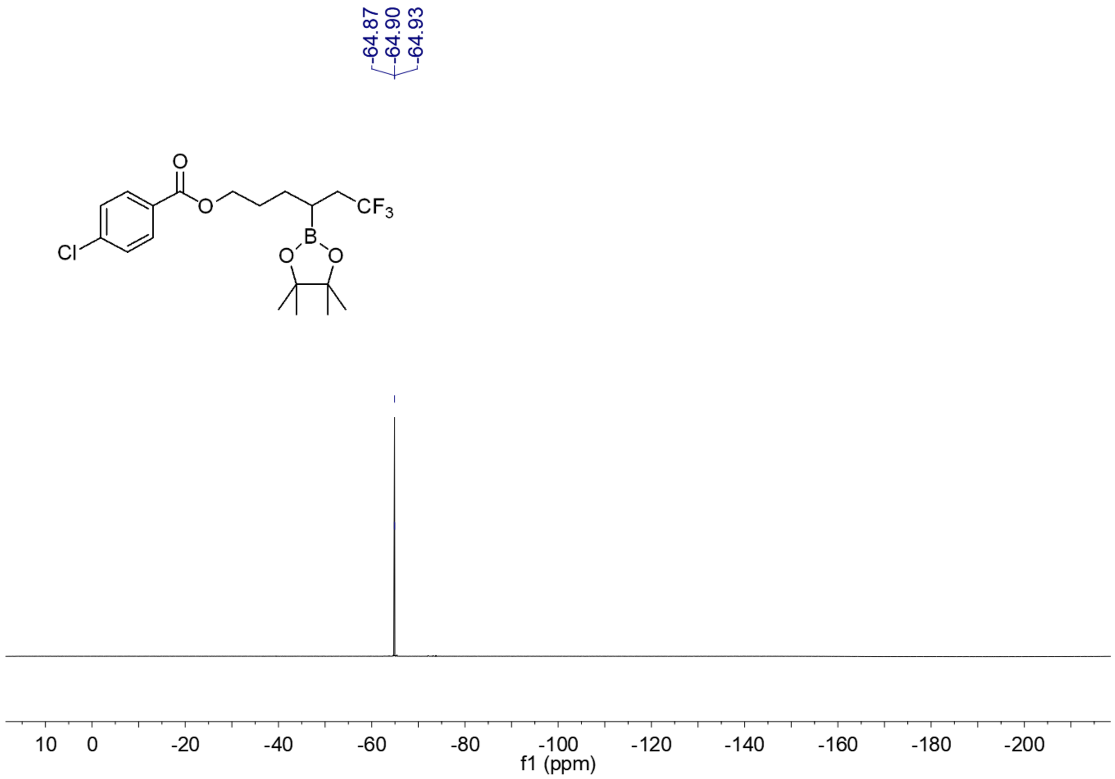

Supplementary Figure 115. ^{19}F NMR Spectra of product **5a**

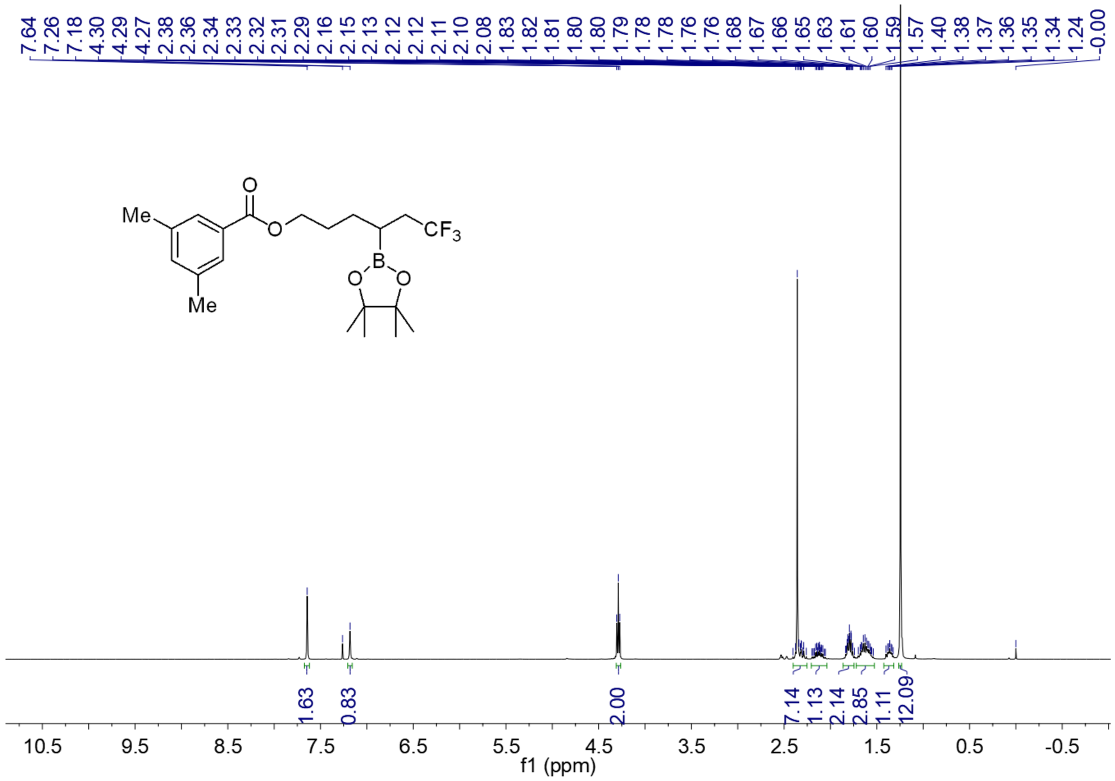

Supplementary Figure 116. ^{1}H NMR Spectra of product **5b**

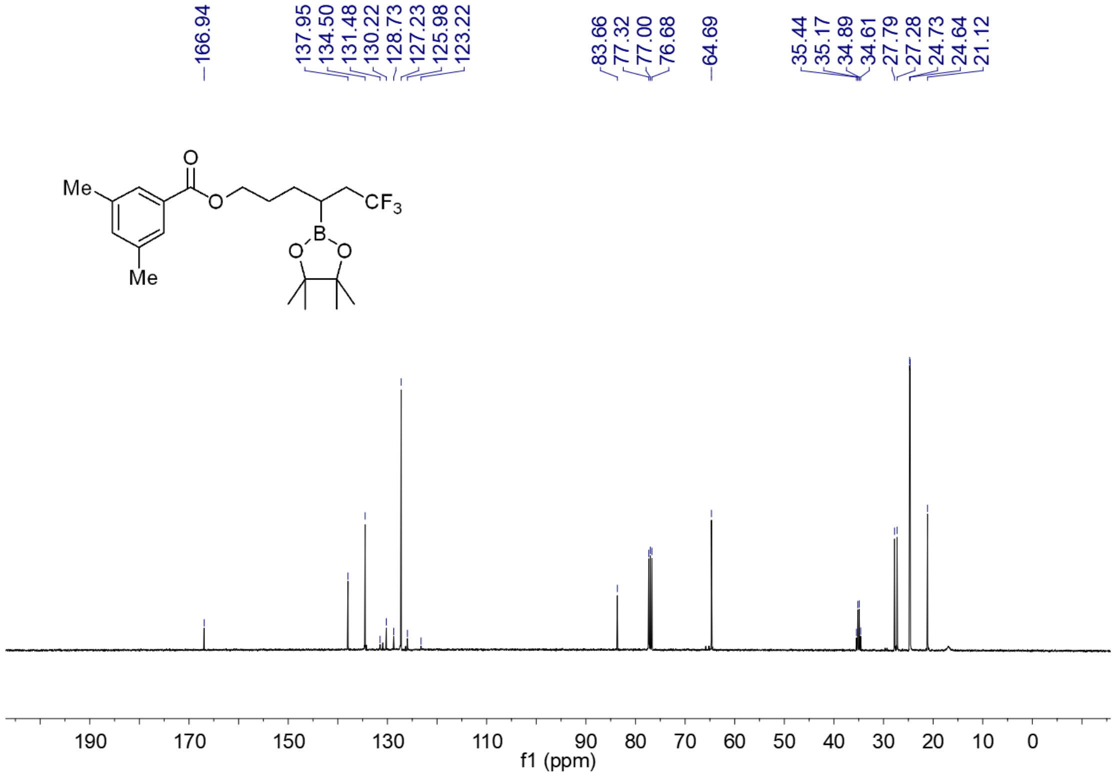

Supplementary Figure 117. ^{13}C NMR Spectra of product **5b**

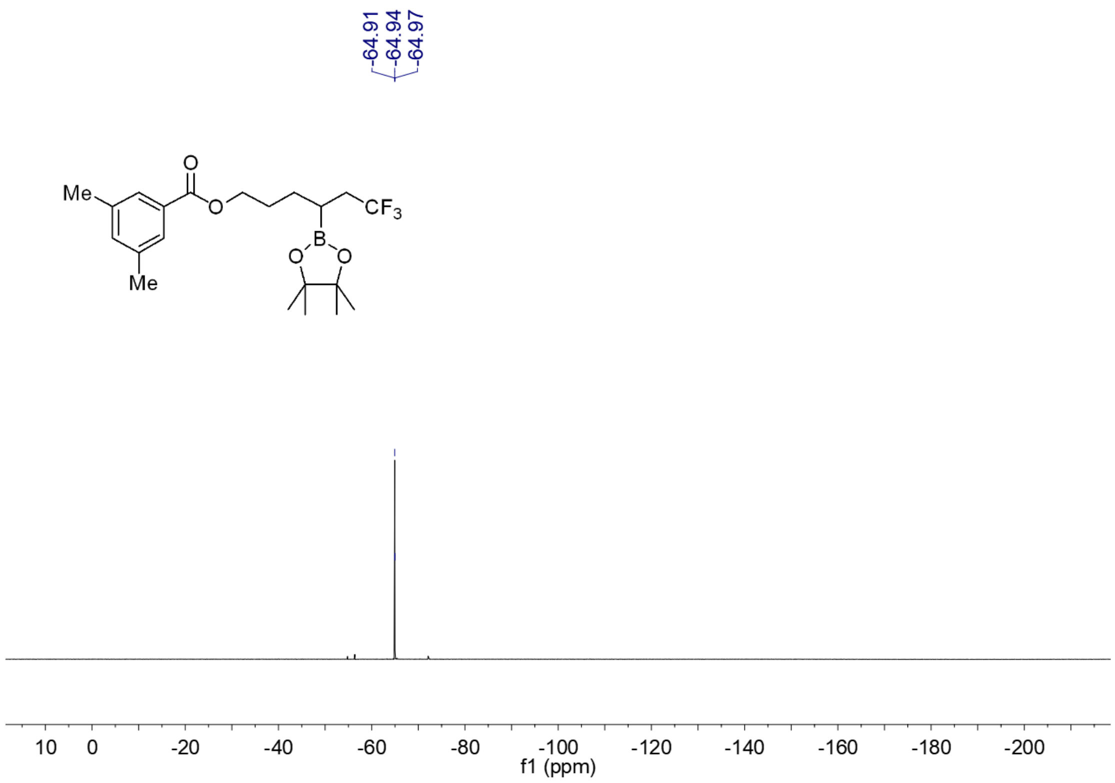

Supplementary Figure 118. ^{19}F NMR Spectra of product **5b**

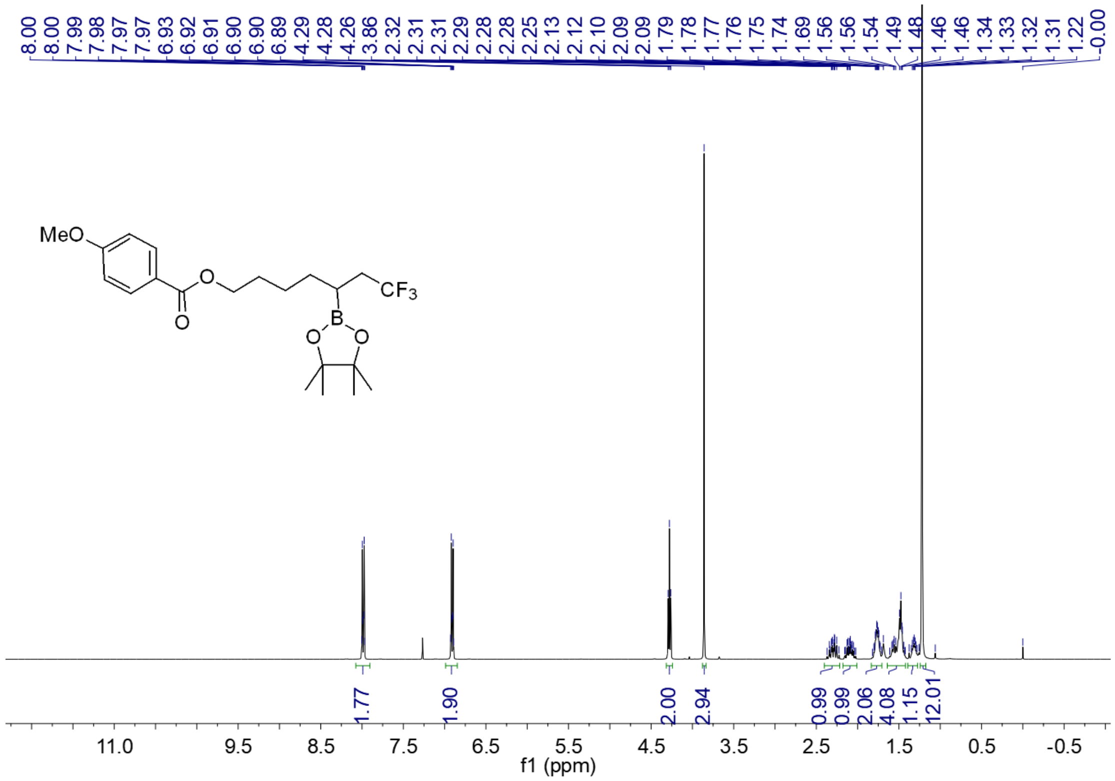

Supplementary Figure 119. ^{1}H NMR Spectra of product **5c**

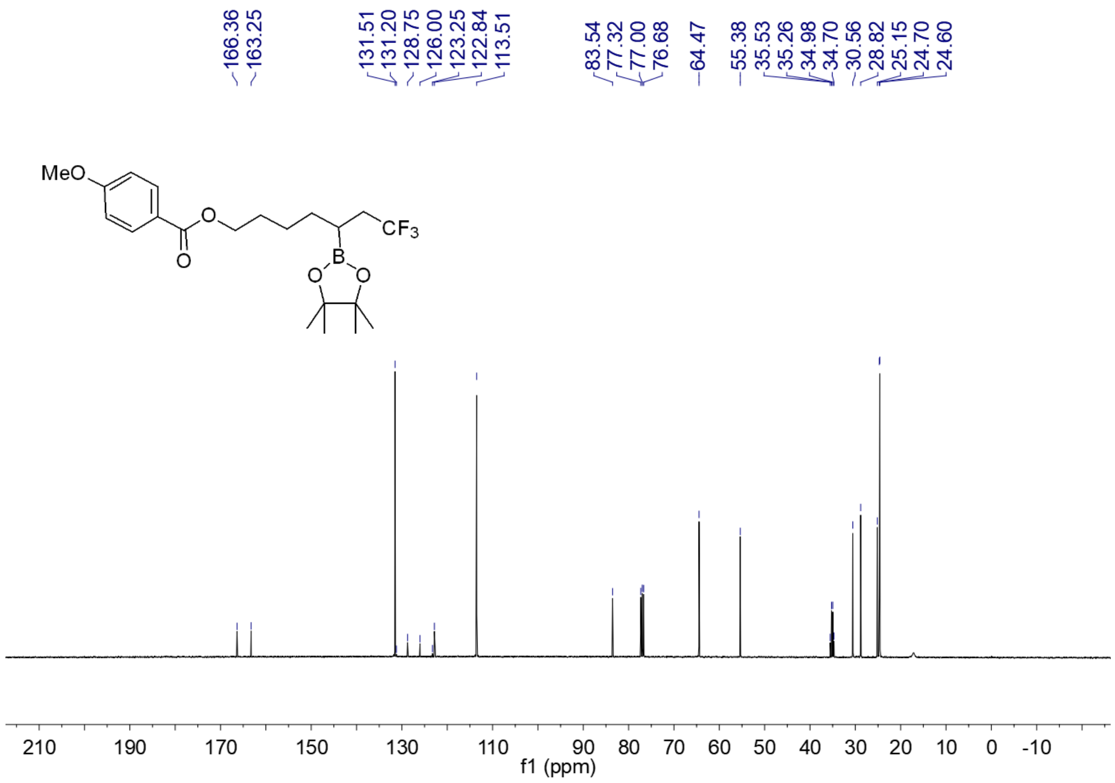

Supplementary Figure 120. ^{13}C NMR Spectra of product **5c**

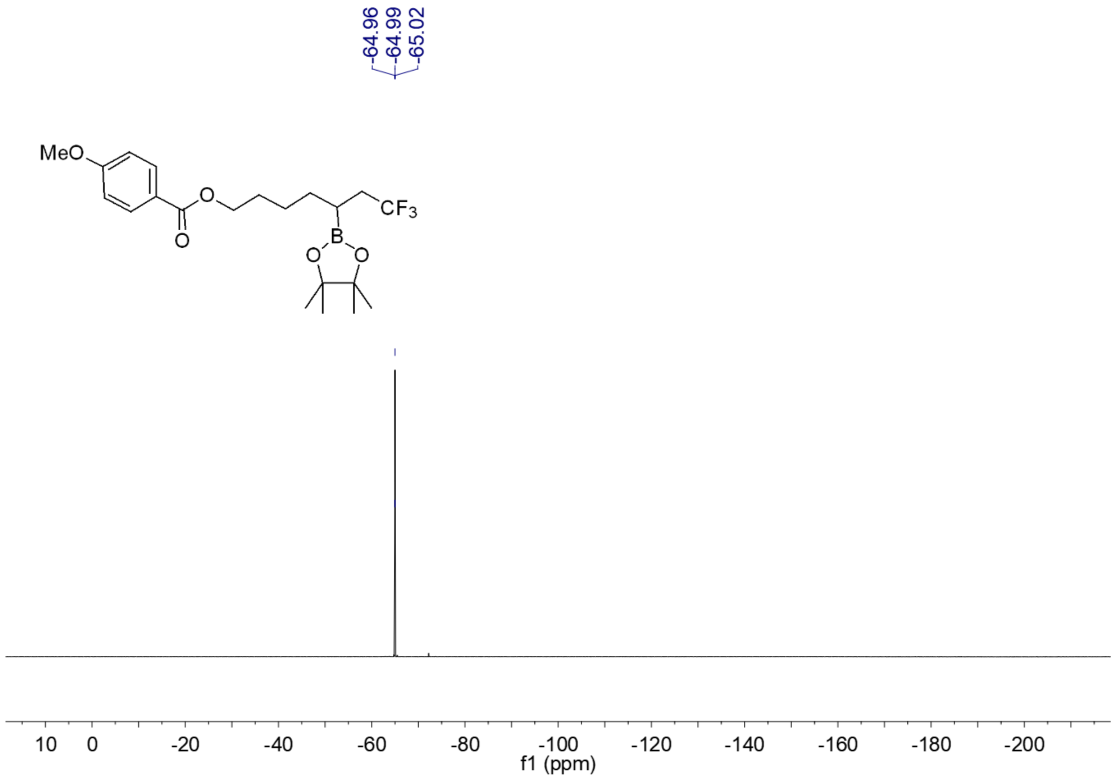

Supplementary Figure 121. ^{19}F NMR Spectra of product **5c**

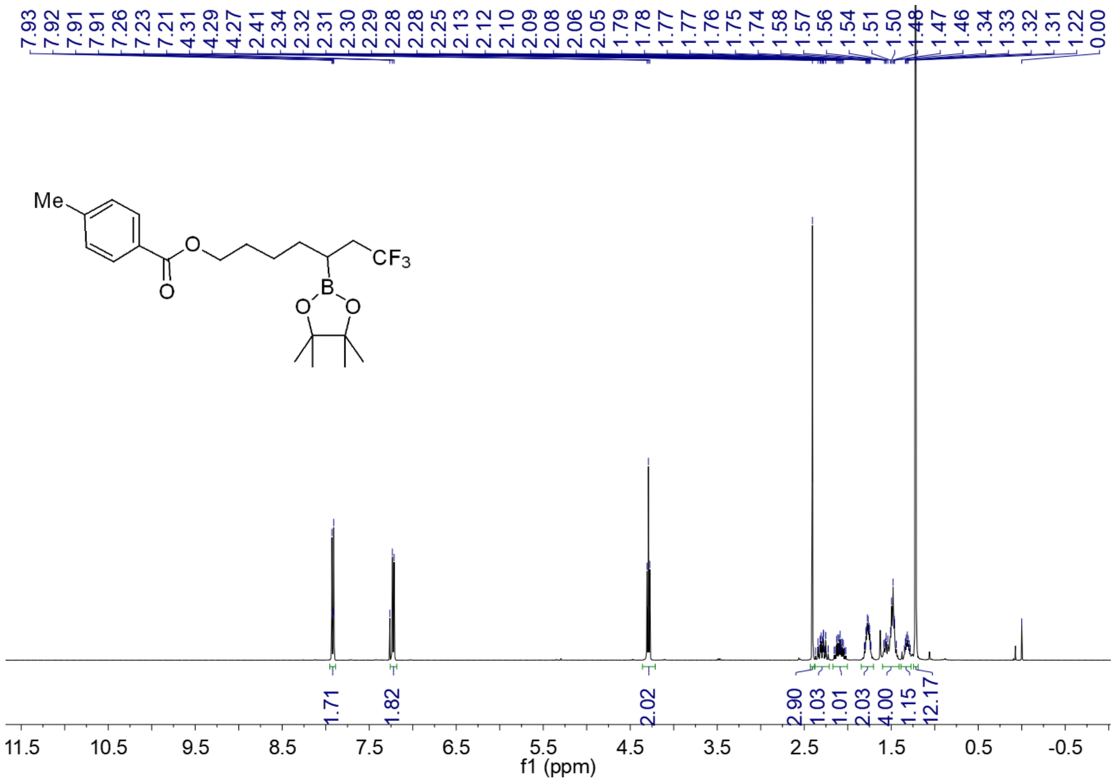

Supplementary Figure 122. ^{1}H NMR Spectra of product **5d**

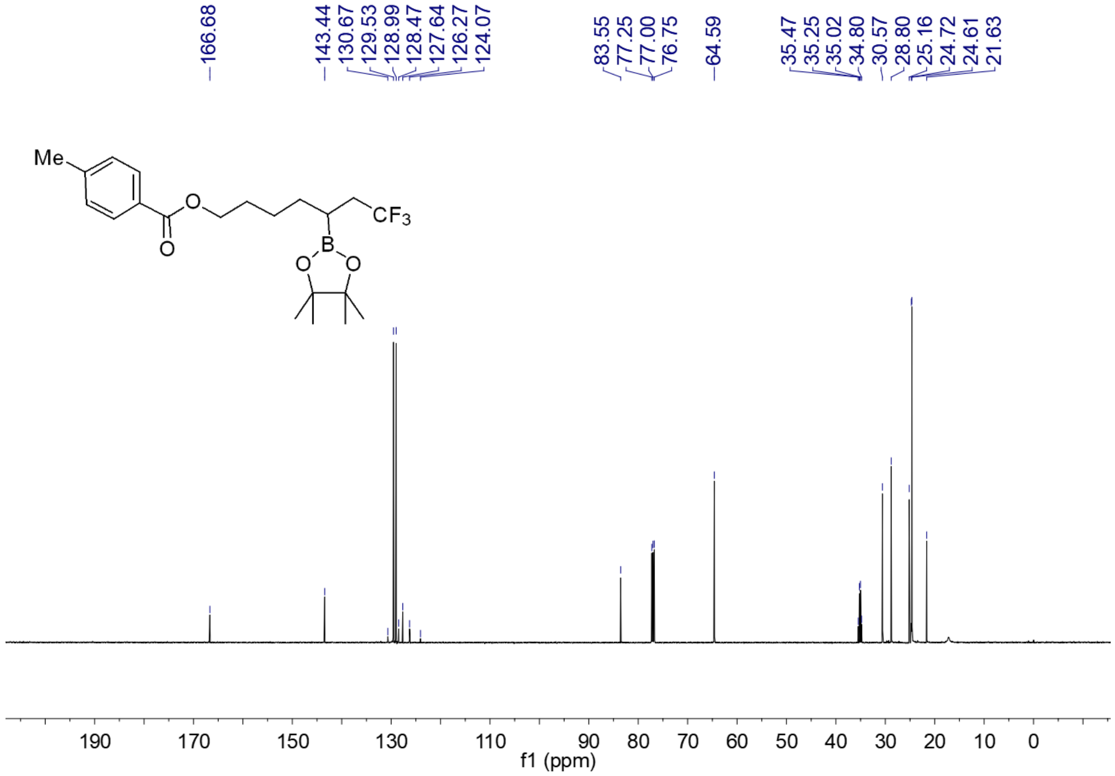

Supplementary Figure 123. ^{13}C NMR Spectra of product **5d**

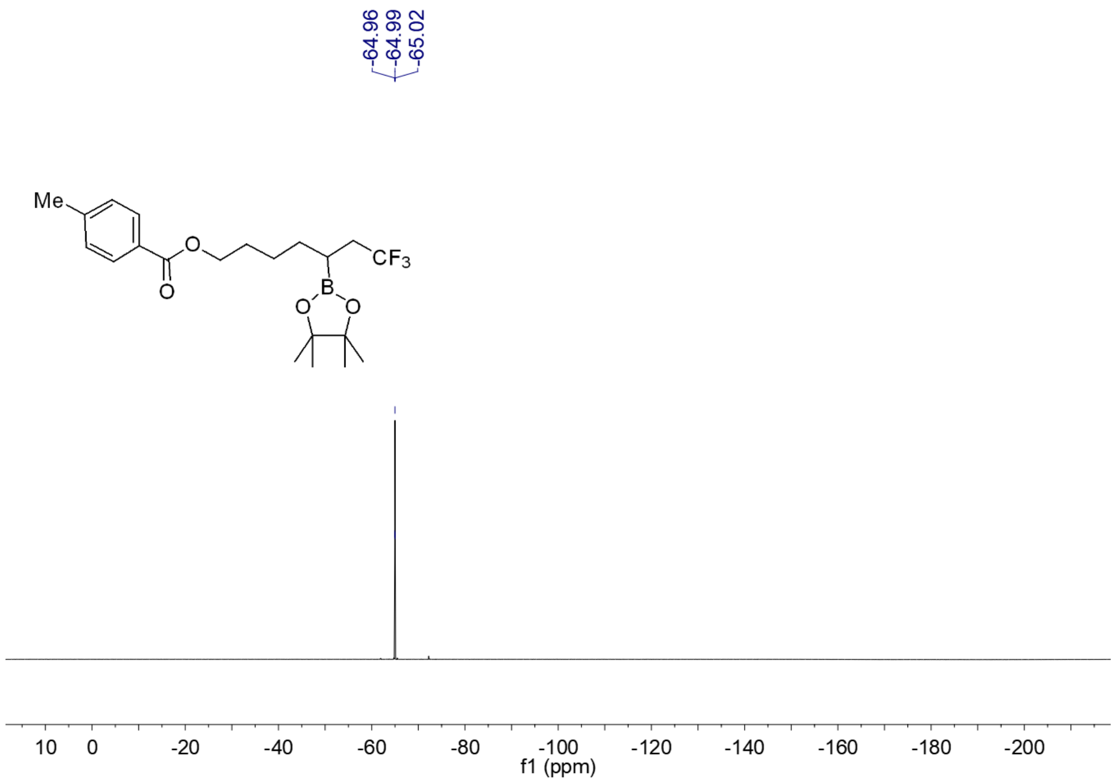

Supplementary Figure 124. ^{19}F NMR Spectra of product **5d**

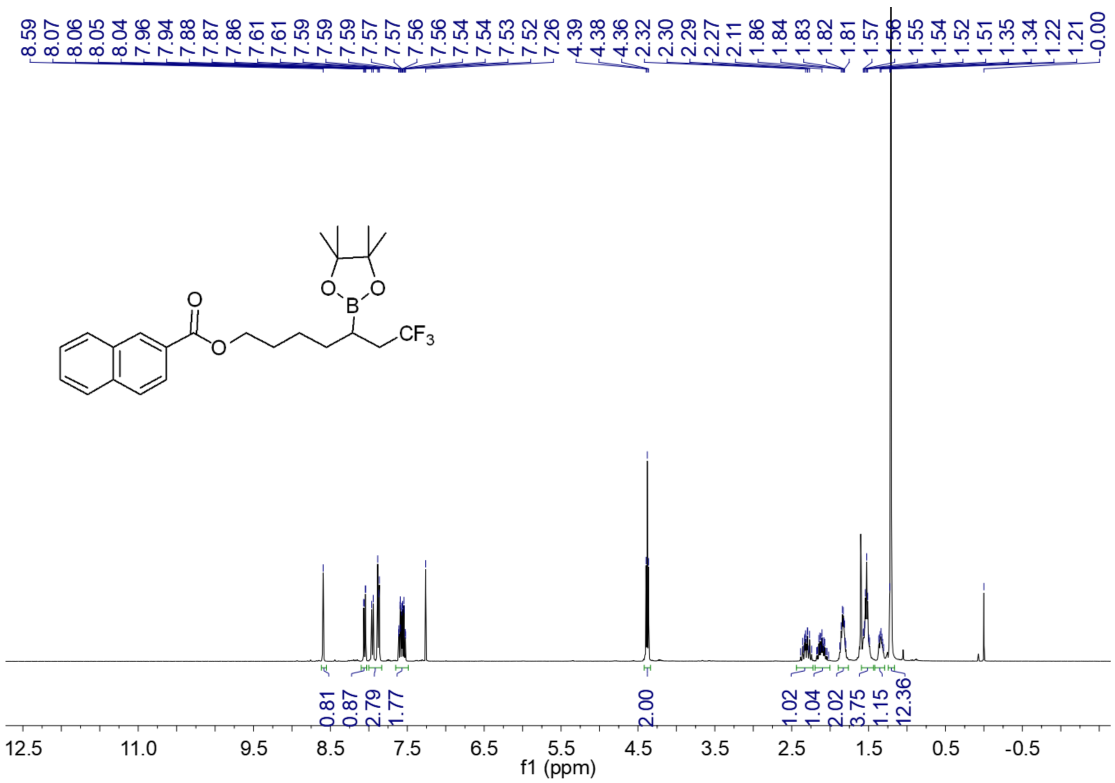

Supplementary Figure 125. ^{1}H NMR Spectra of product **5e**

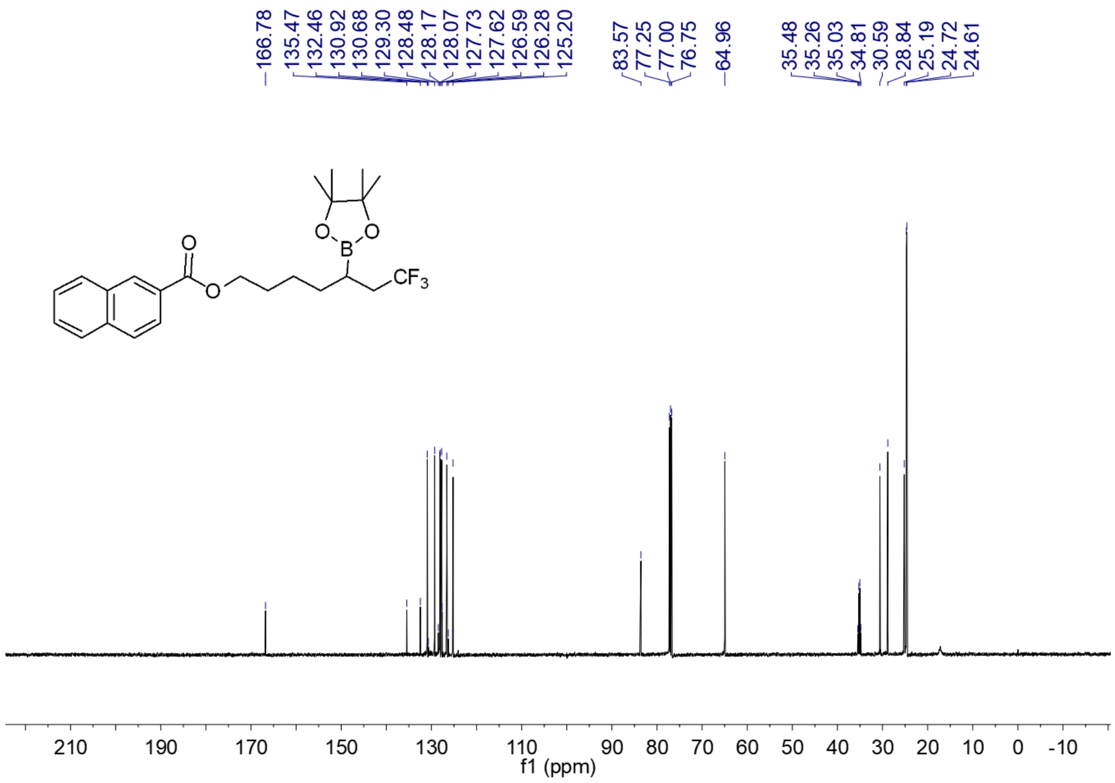

Supplementary Figure 126. ^{13}C NMR Spectra of product **5e**

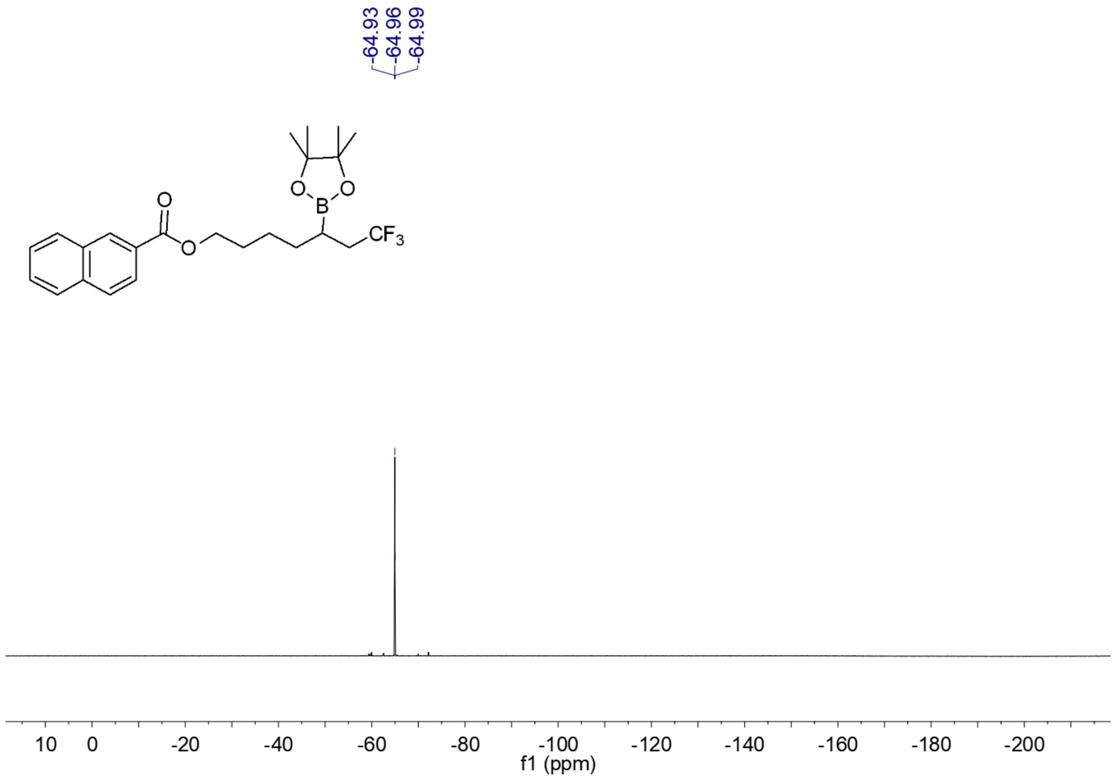

Supplementary Figure 127. ^{19}F NMR Spectra of product **5e**

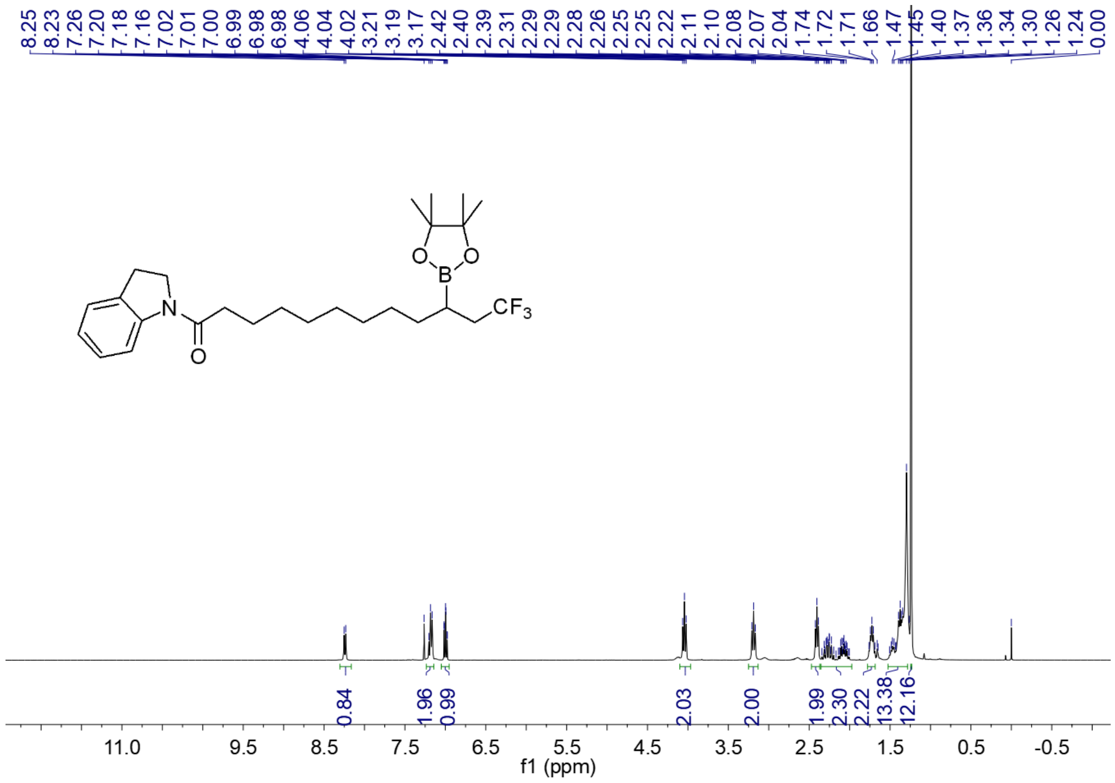

Supplementary Figure 128. ^{1}H NMR Spectra of product **5f**

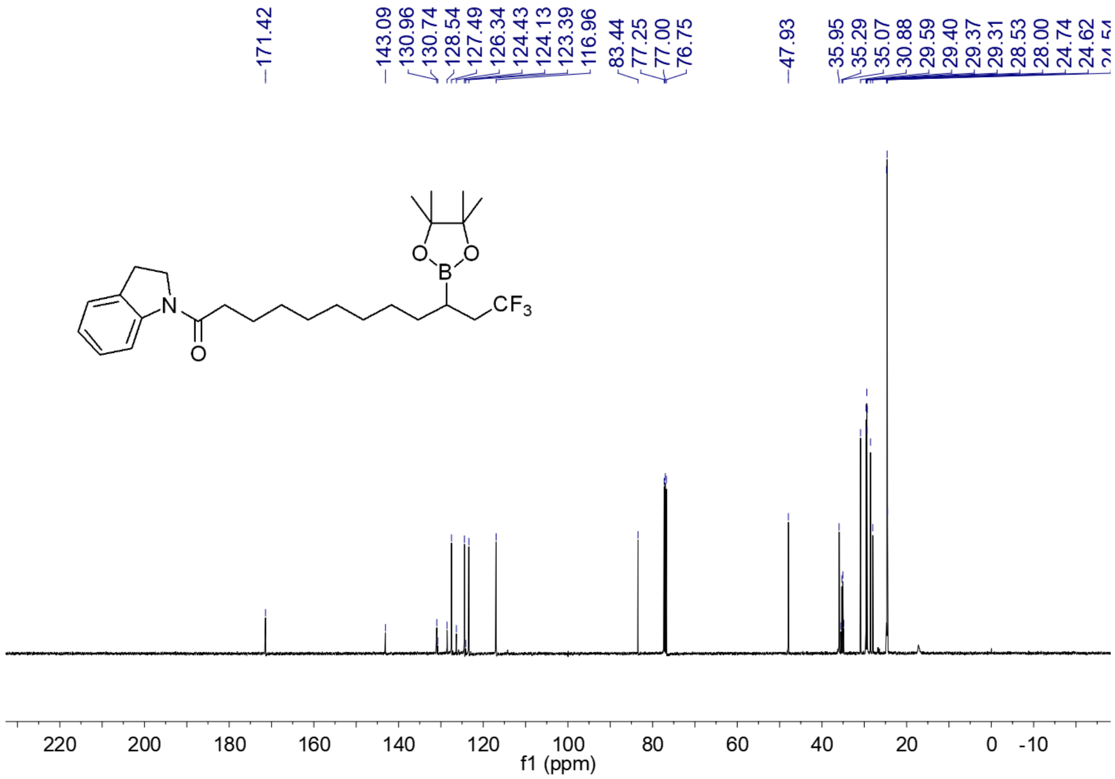

Supplementary Figure 129. ^{13}C NMR Spectra of product **5f**

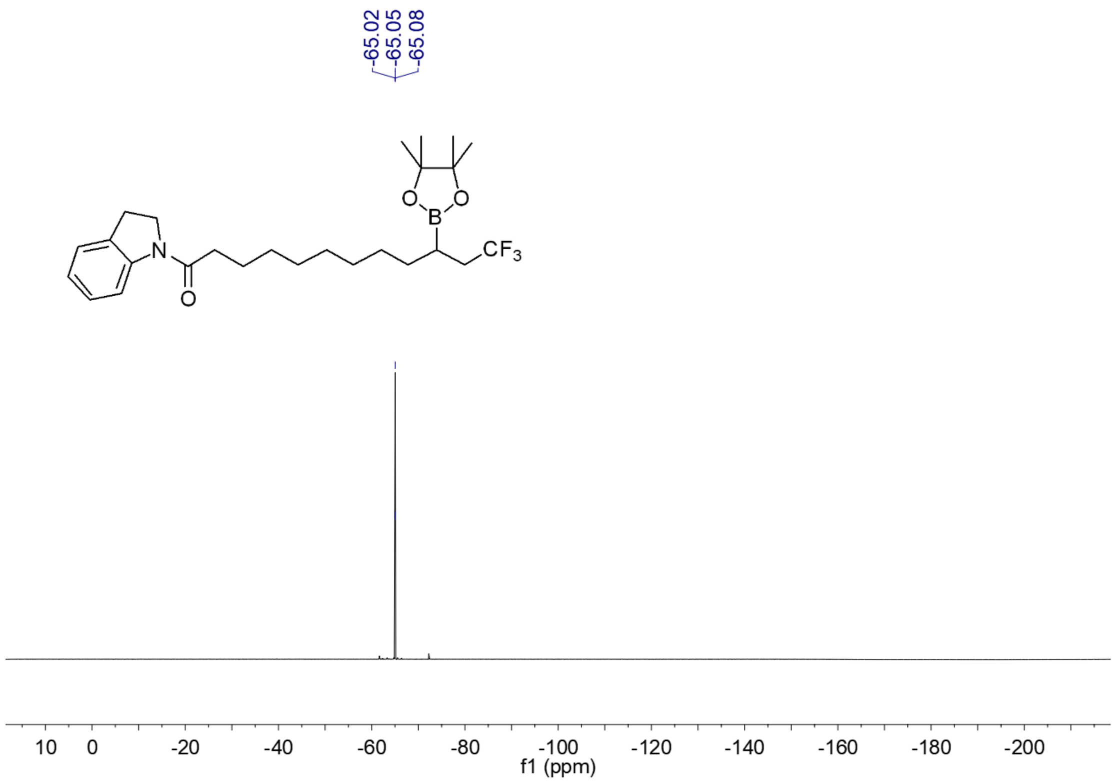

Supplementary Figure 130. ^{19}F NMR Spectra of product **5f**

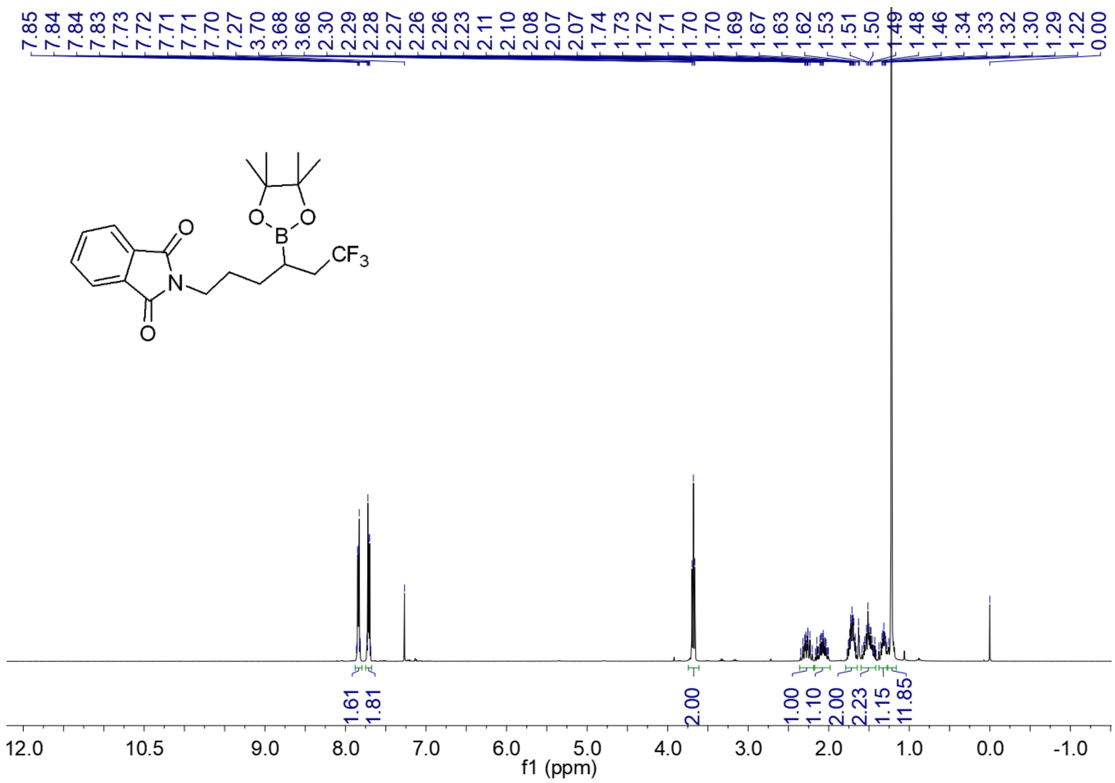

Supplementary Figure 131. ^{1}H NMR Spectra of product **5g**

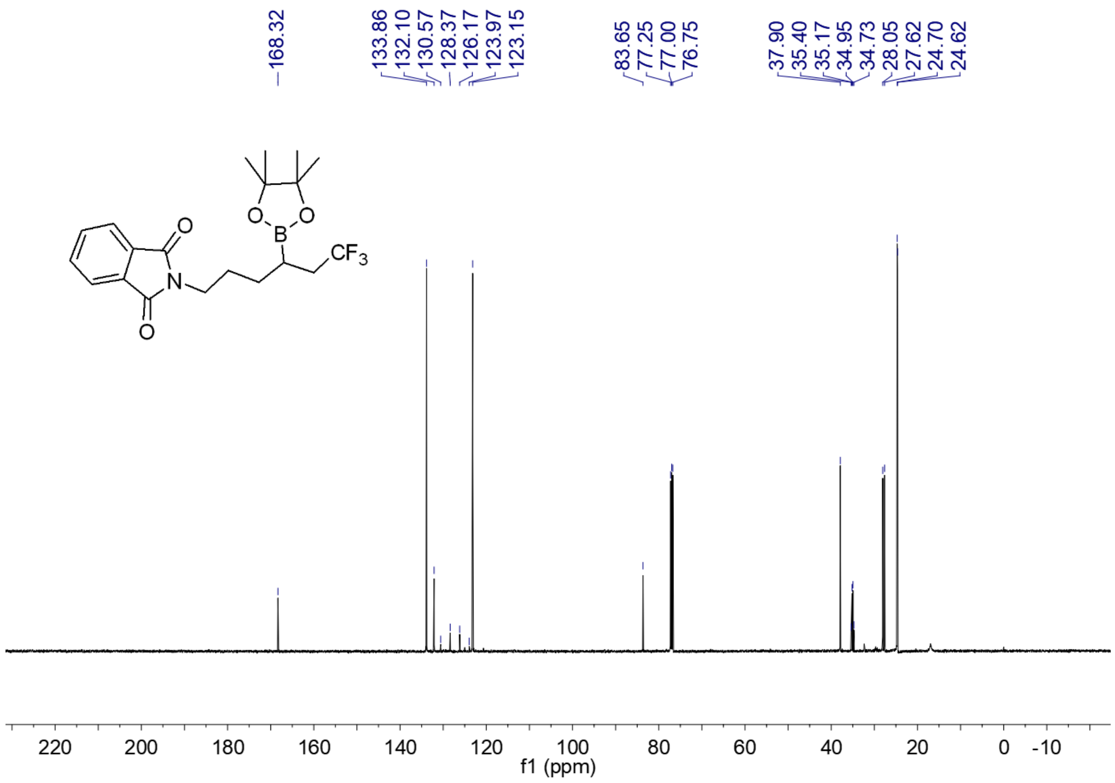

Supplementary Figure 132. ^{13}C NMR Spectra of product **5g**

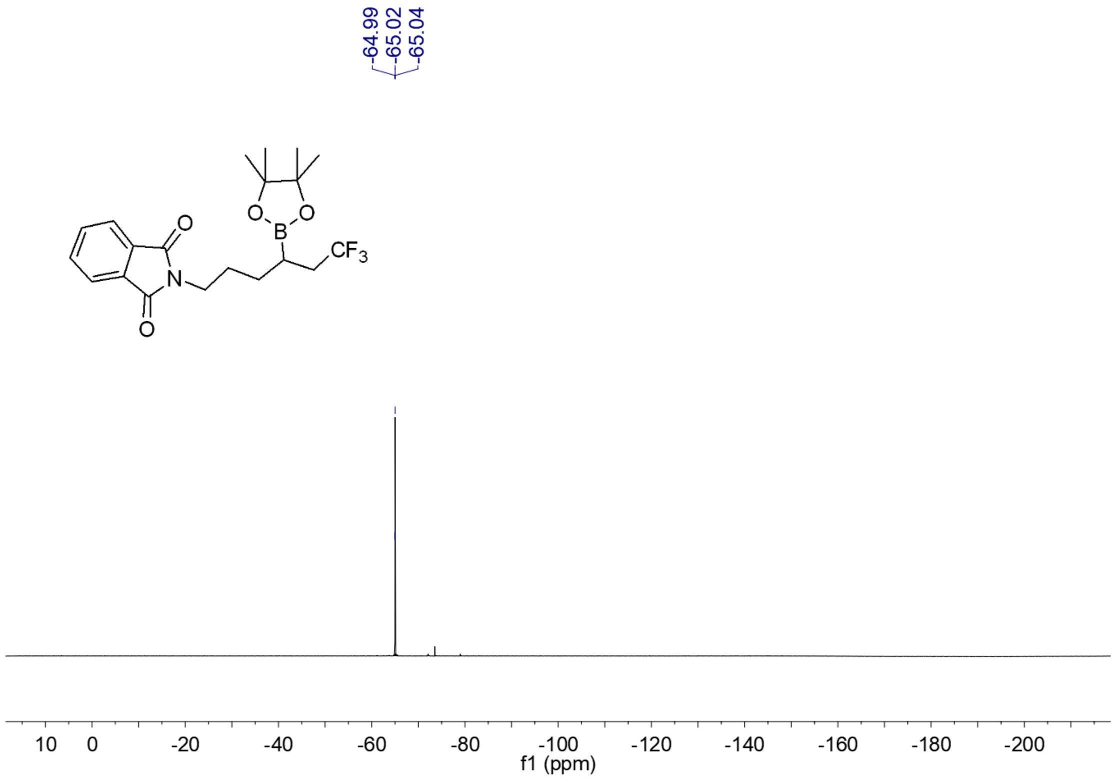

Supplementary Figure 133. ^{19}F NMR Spectra of product **5g**

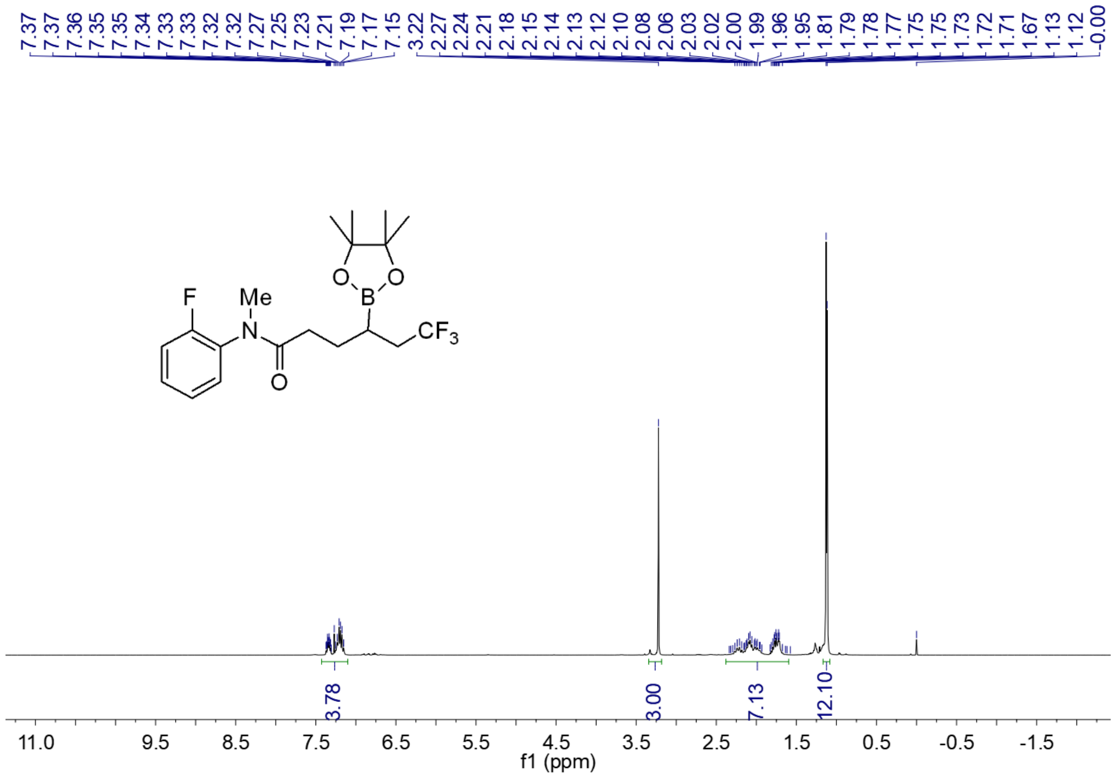

Supplementary Figure 134. ^{1}H NMR Spectra of product **5h**

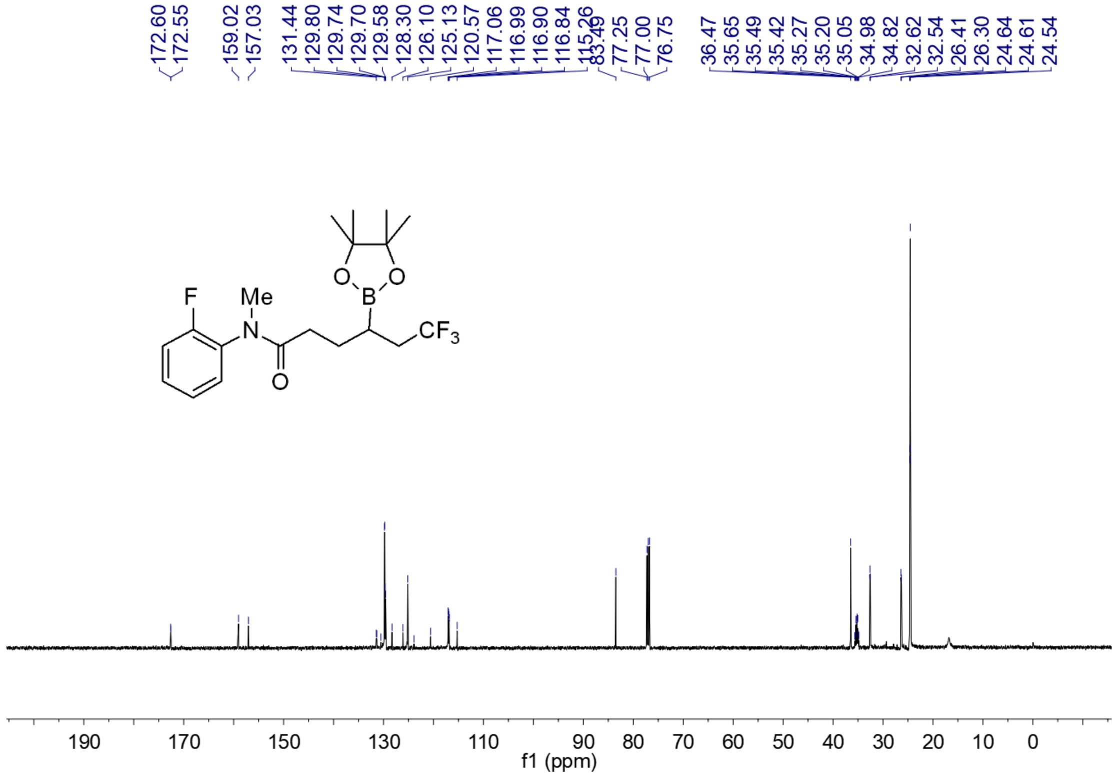

Supplementary Figure 135. ^{13}C NMR Spectra of product **5h**

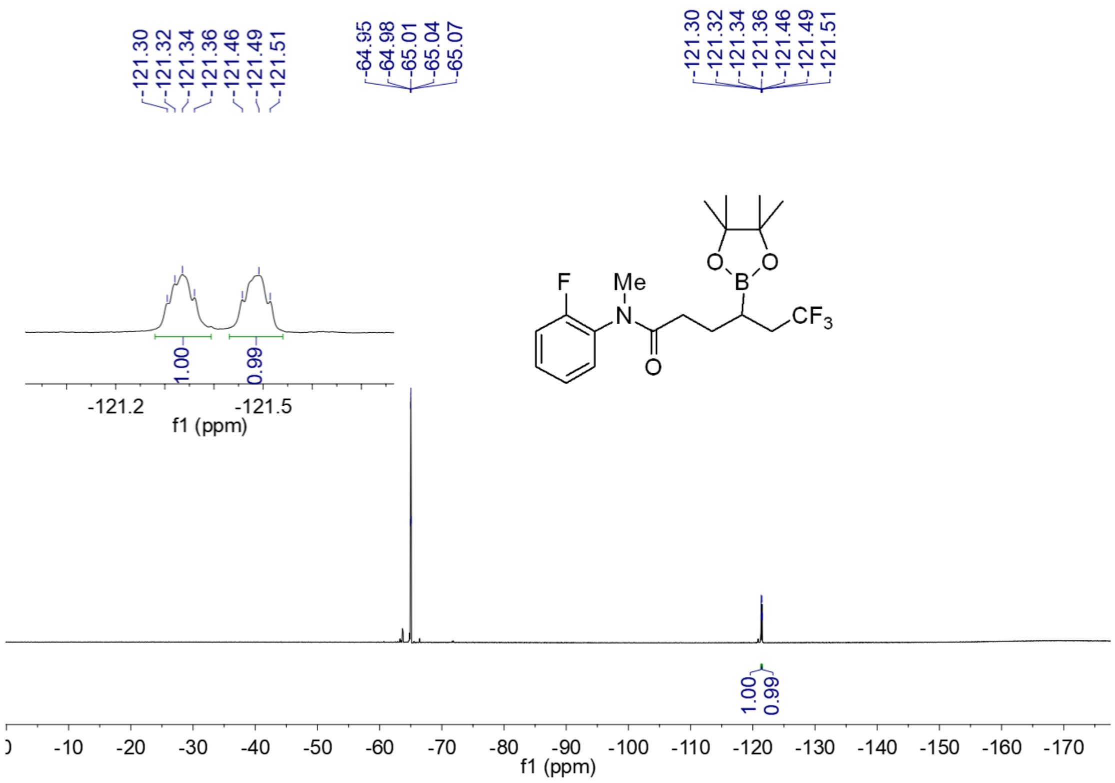

Supplementary Figure 136. ^{19}F NMR Spectra of product **5h**

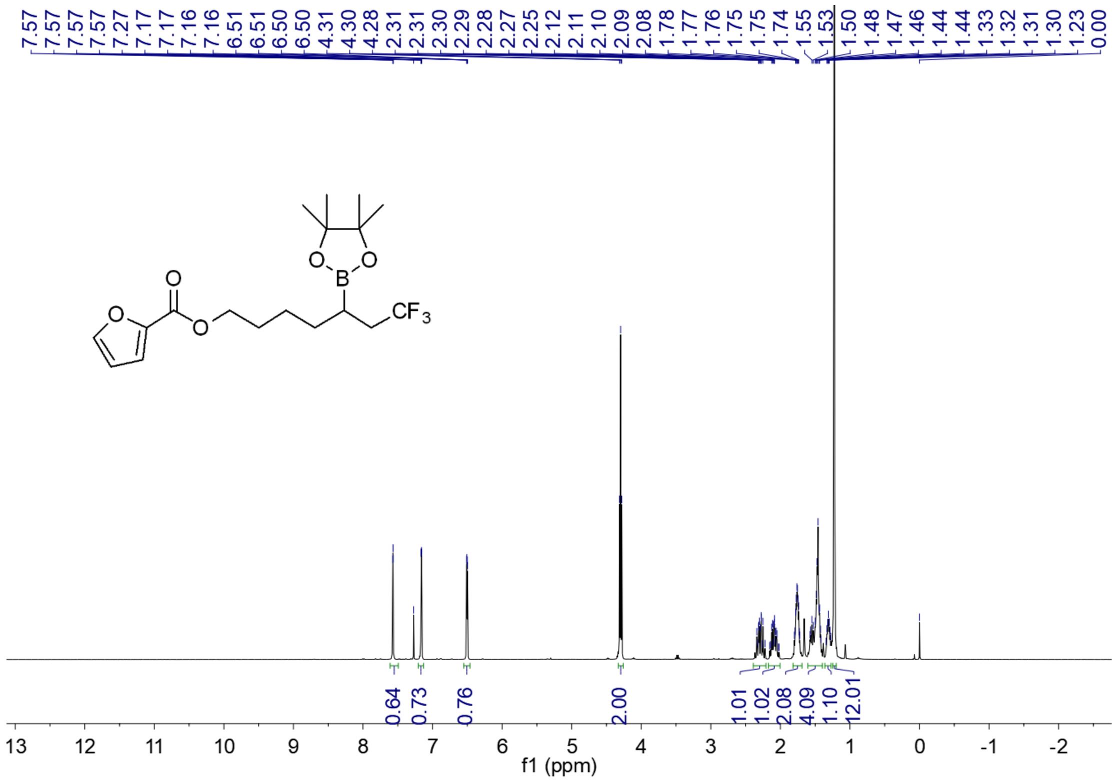

Supplementary Figure 137. ^{1}H NMR Spectra of product **5i**

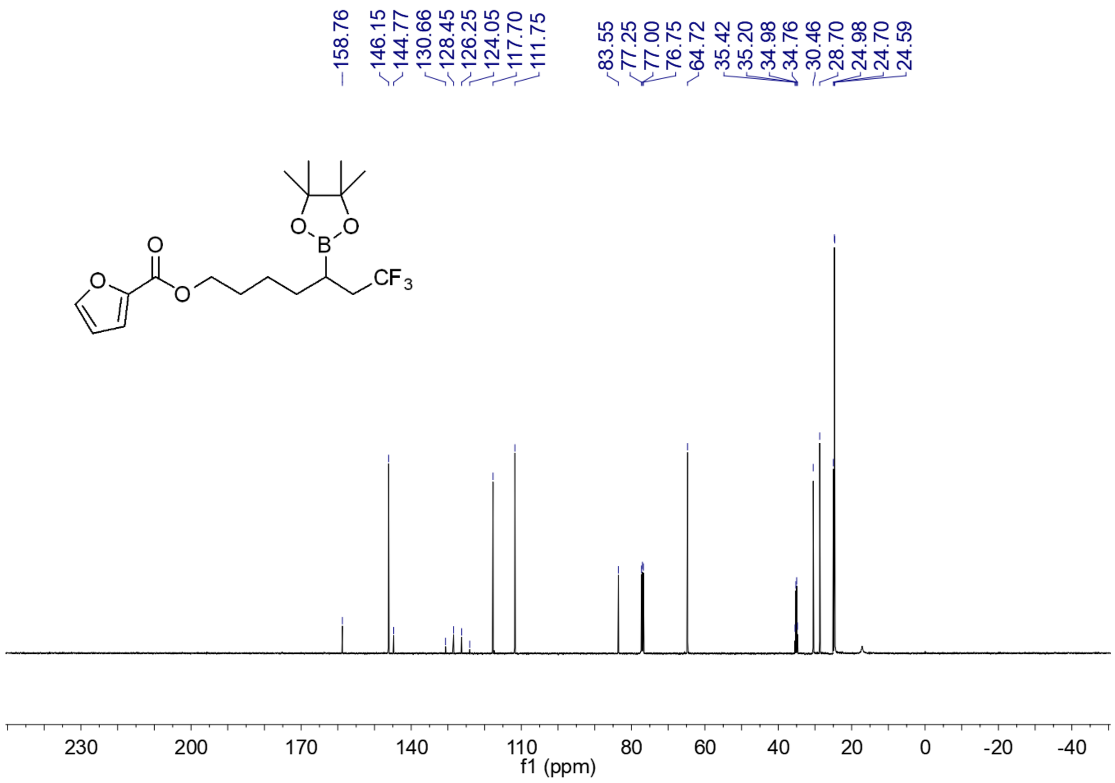

Supplementary Figure 138. ^{13}C NMR Spectra of product **5i**

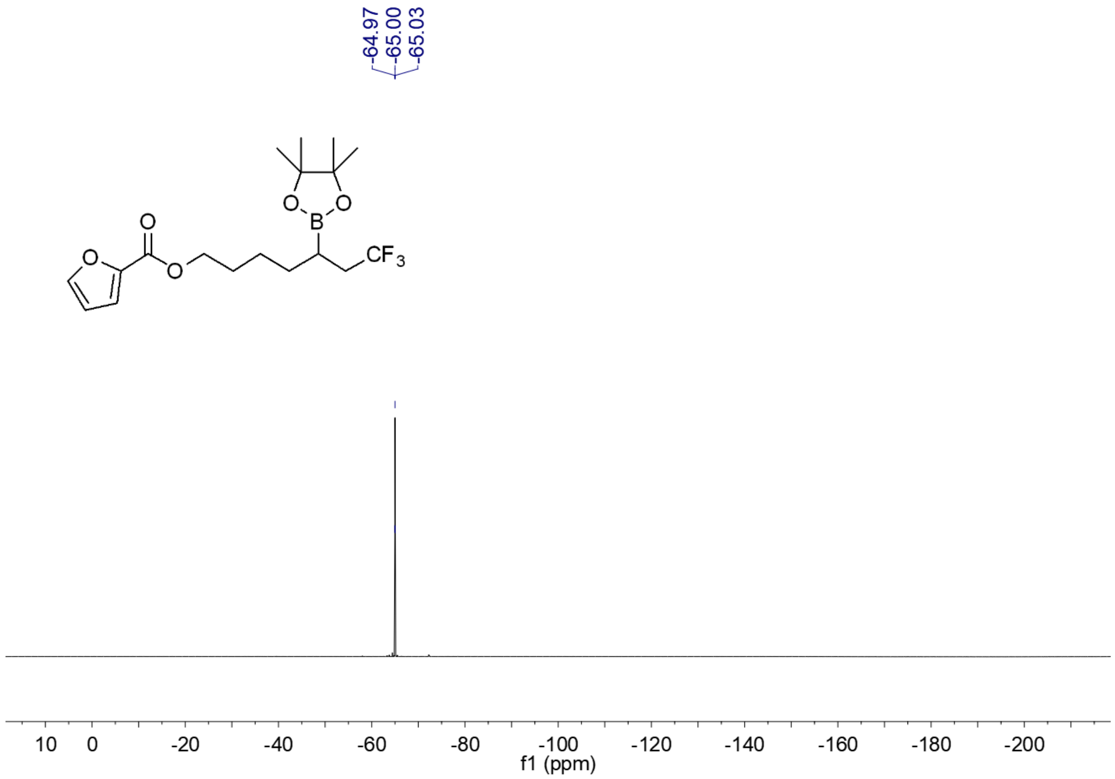

Supplementary Figure 139. ^{19}F NMR Spectra of product **5i**

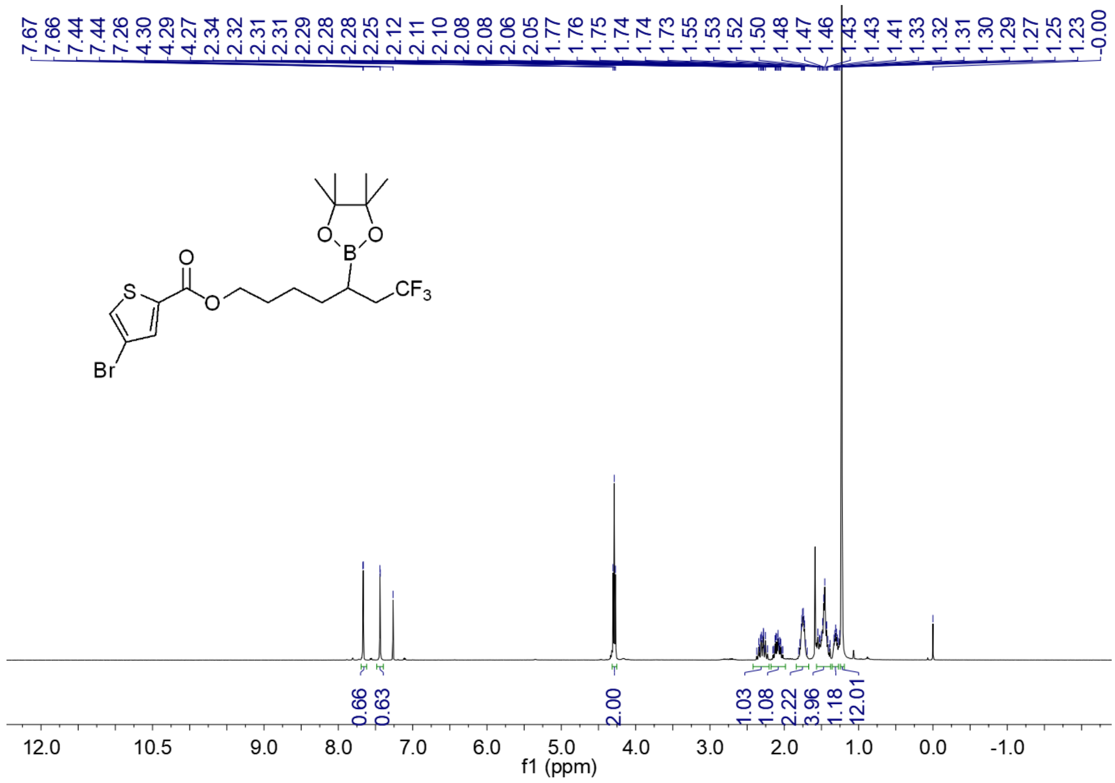

Supplementary Figure 140. ^{1}H NMR Spectra of product **5j**

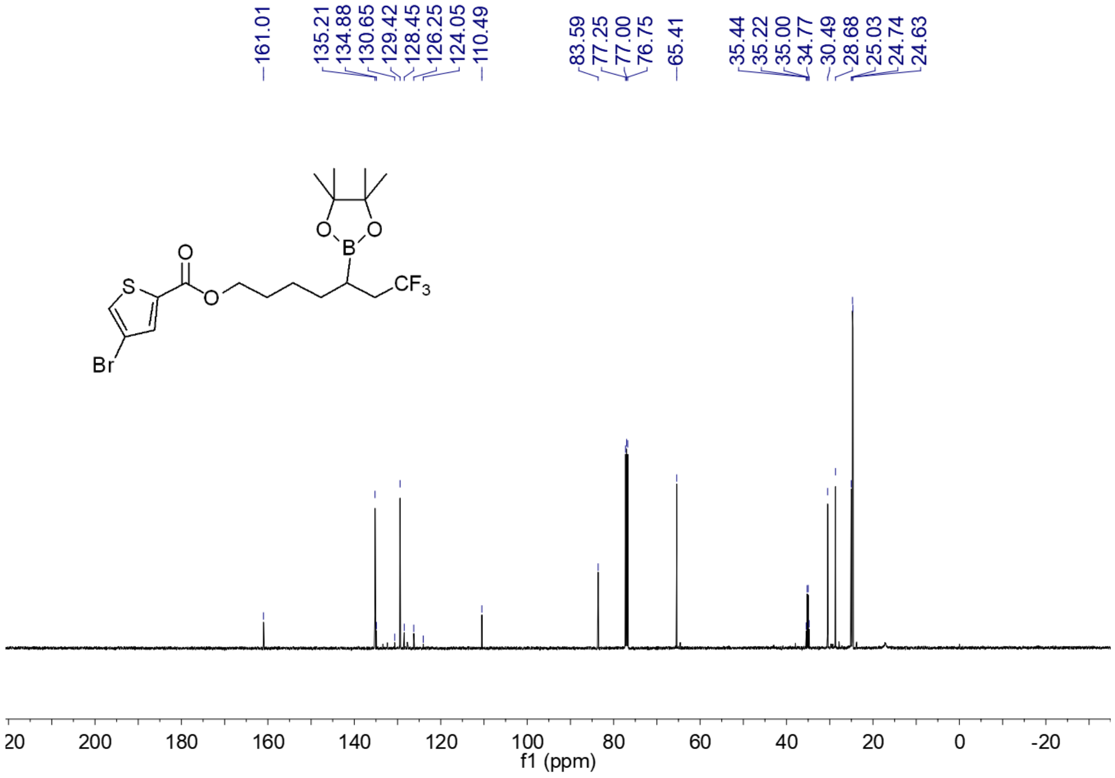

Supplementary Figure 141. ^{13}C NMR Spectra of product **5j**

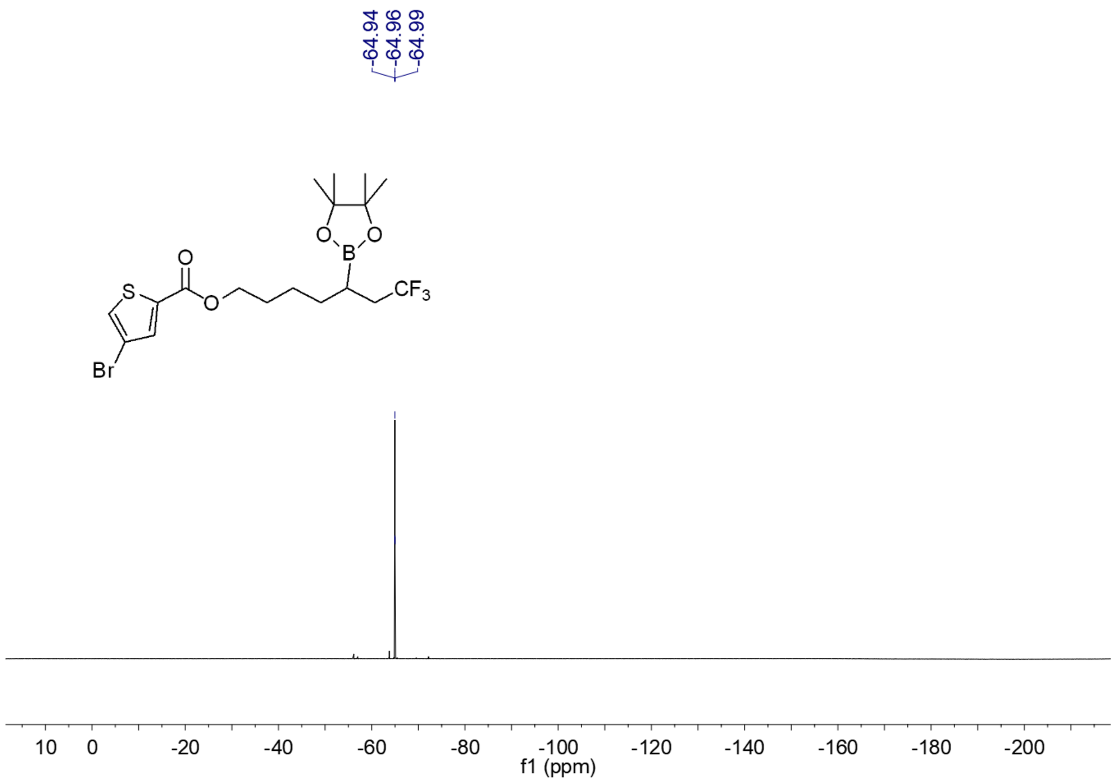

Supplementary Figure 142. ^{19}F NMR Spectra of product **5j**

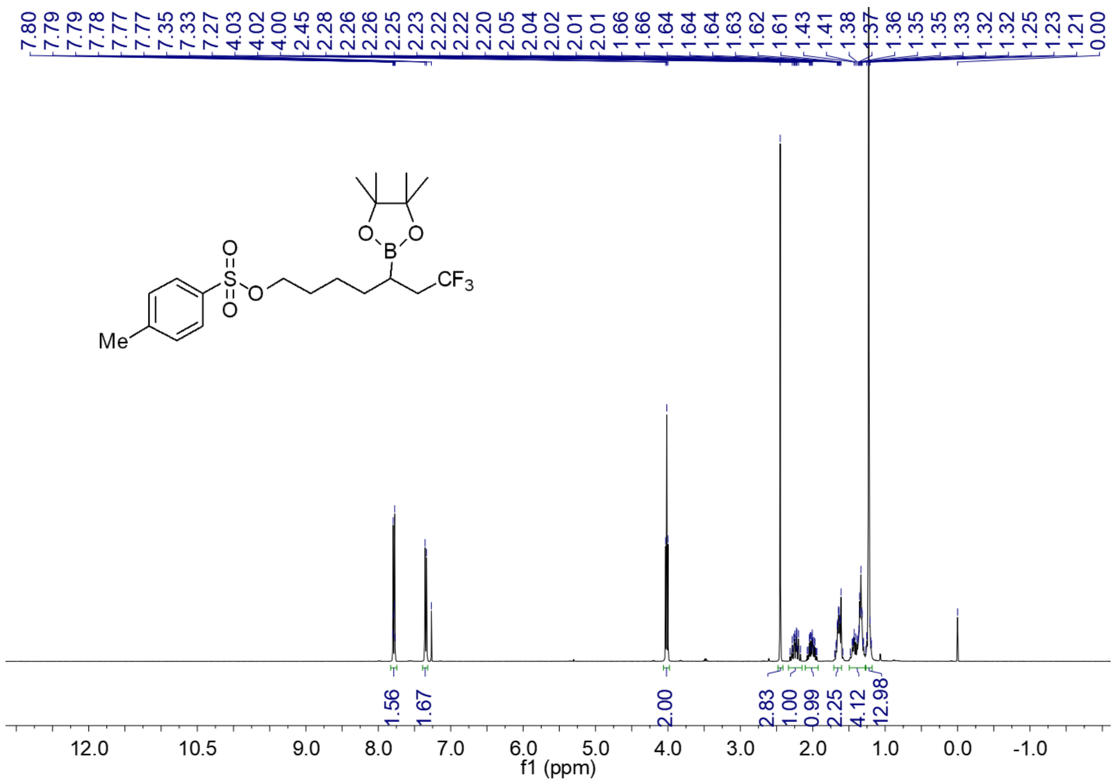

Supplementary Figure 143. ^{1}H NMR Spectra of product **5k**

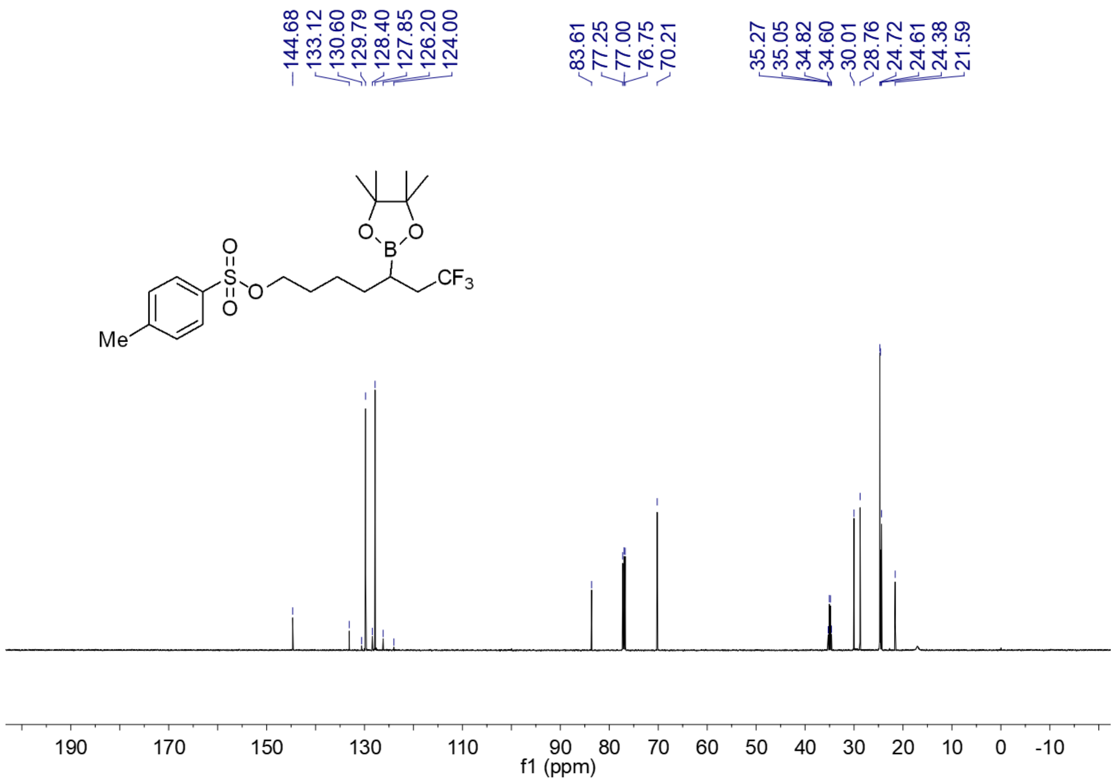

Supplementary Figure 144. ^{13}C NMR Spectra of product **5k**

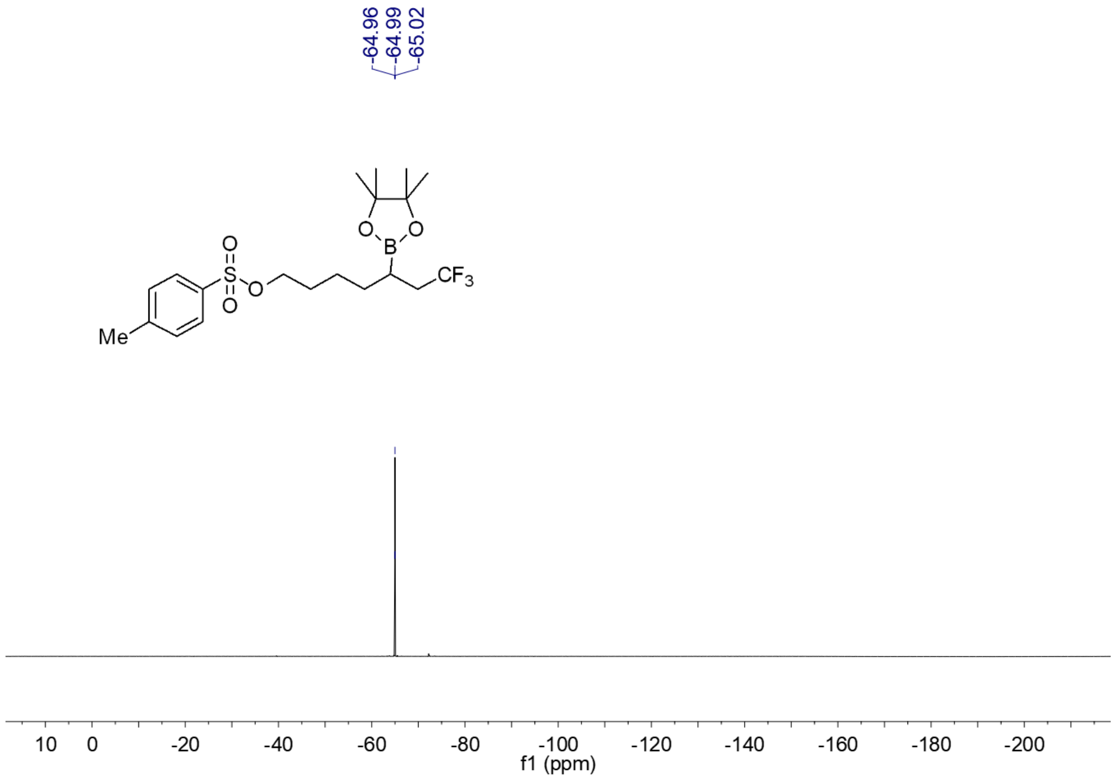

Supplementary Figure 145. ^{19}F NMR Spectra of product **5k**

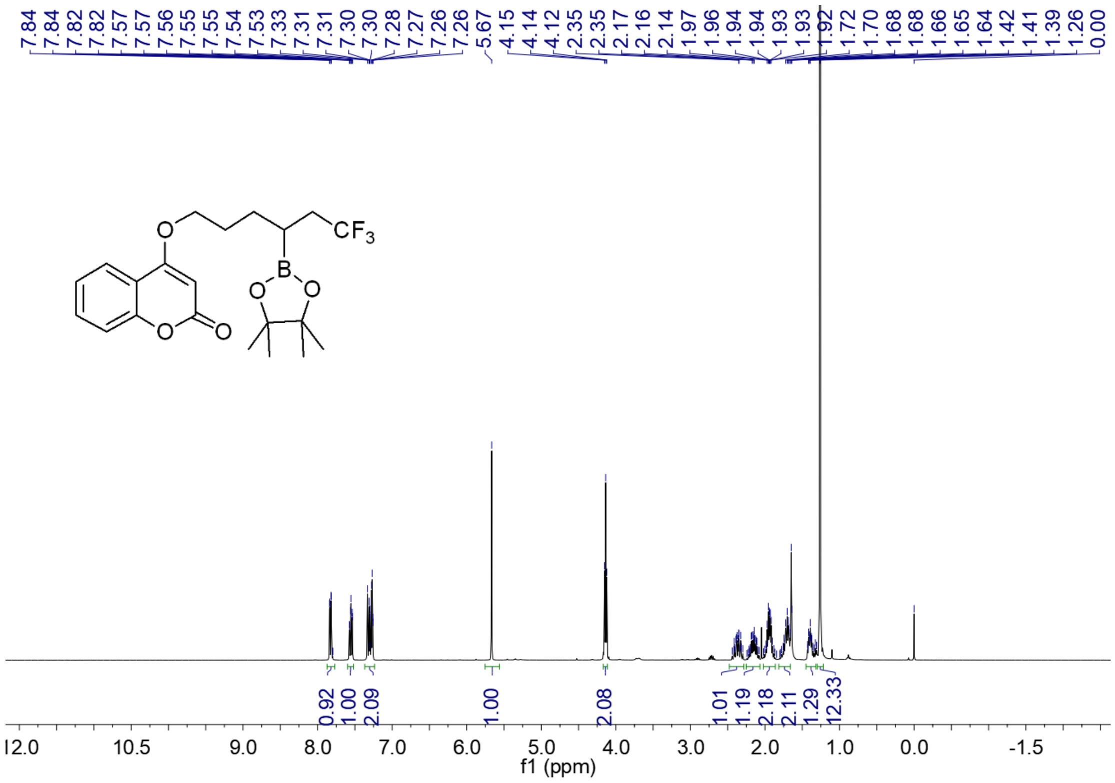

Supplementary Figure 146. ^{1}H NMR Spectra of product **5l**

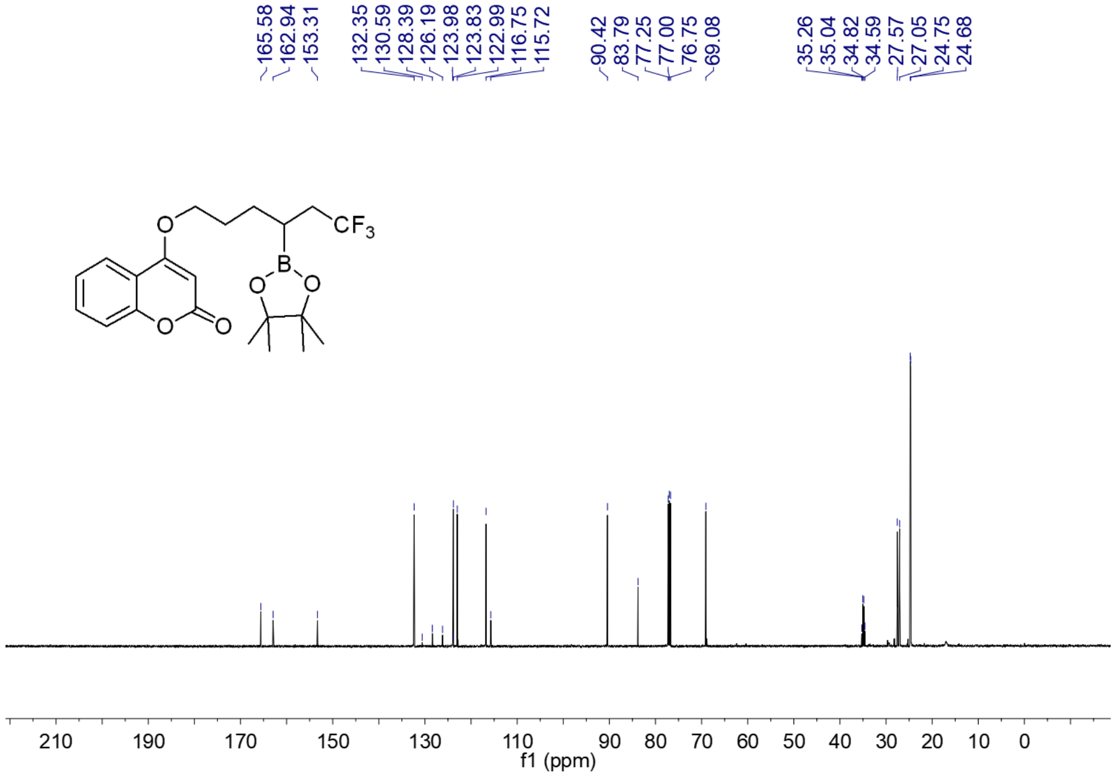

Supplementary Figure 147. ^{13}C NMR Spectra of product **5l**

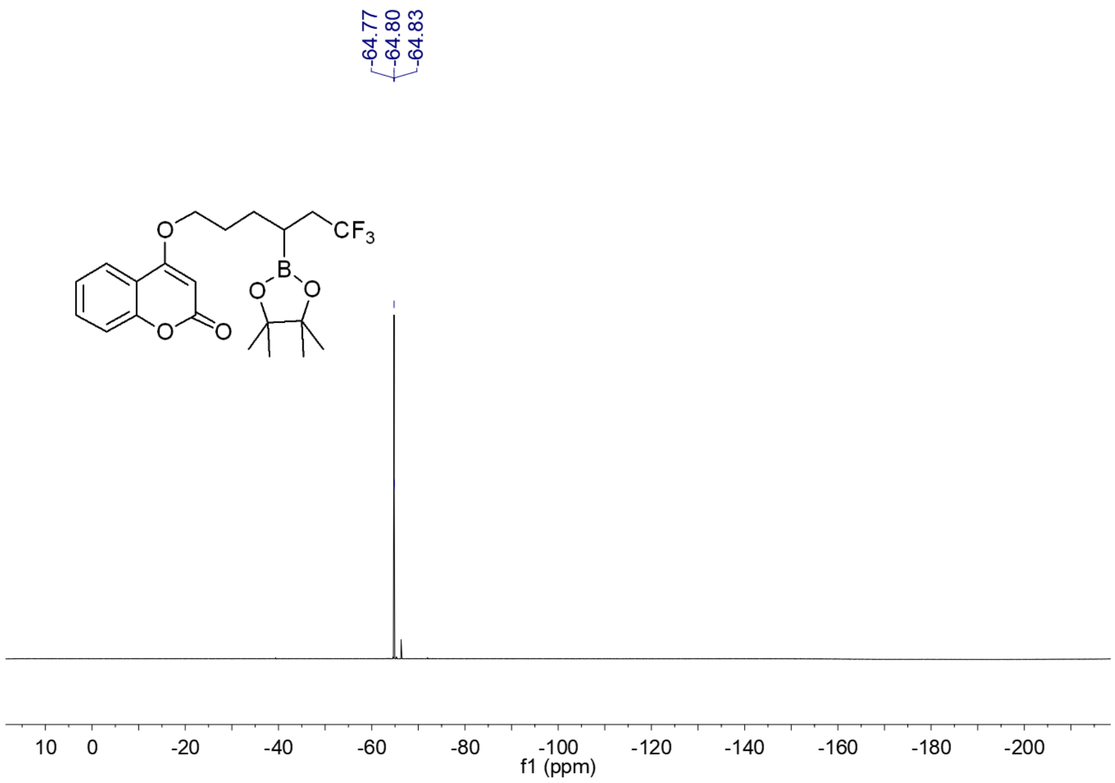

Supplementary Figure 148. ^{19}F NMR Spectra of product **5l**

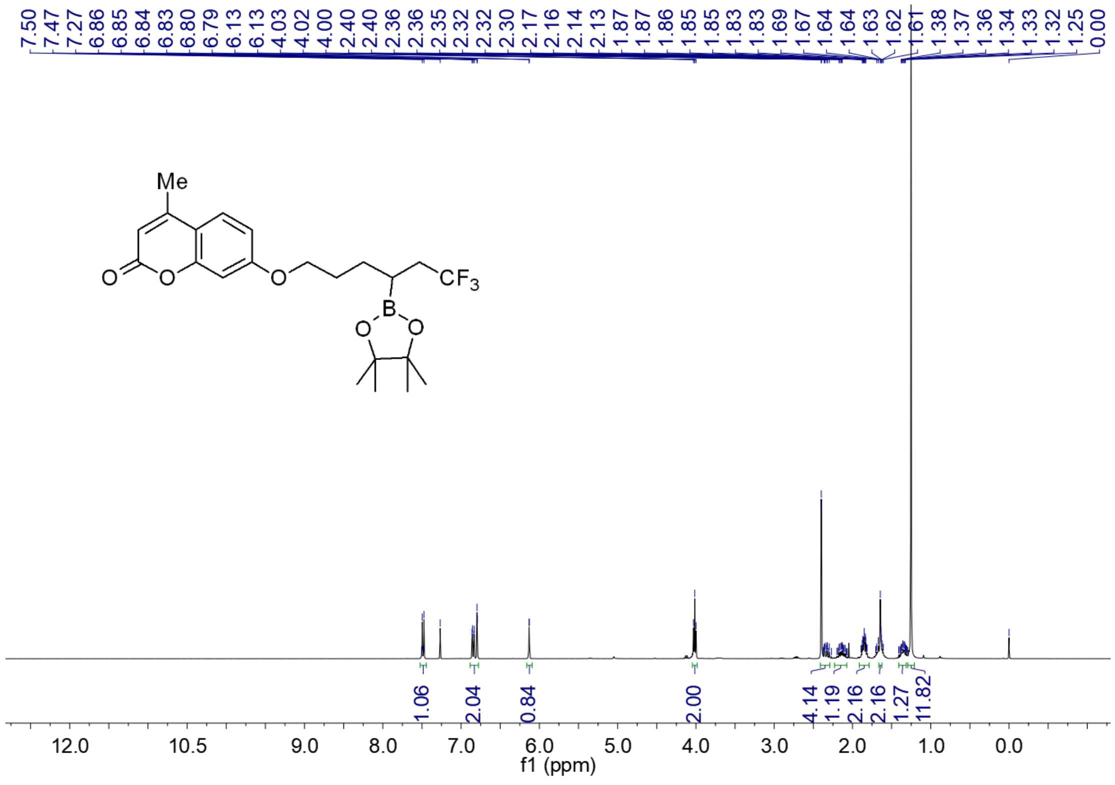

Supplementary Figure 149. ^{1}H NMR Spectra of product **5m**

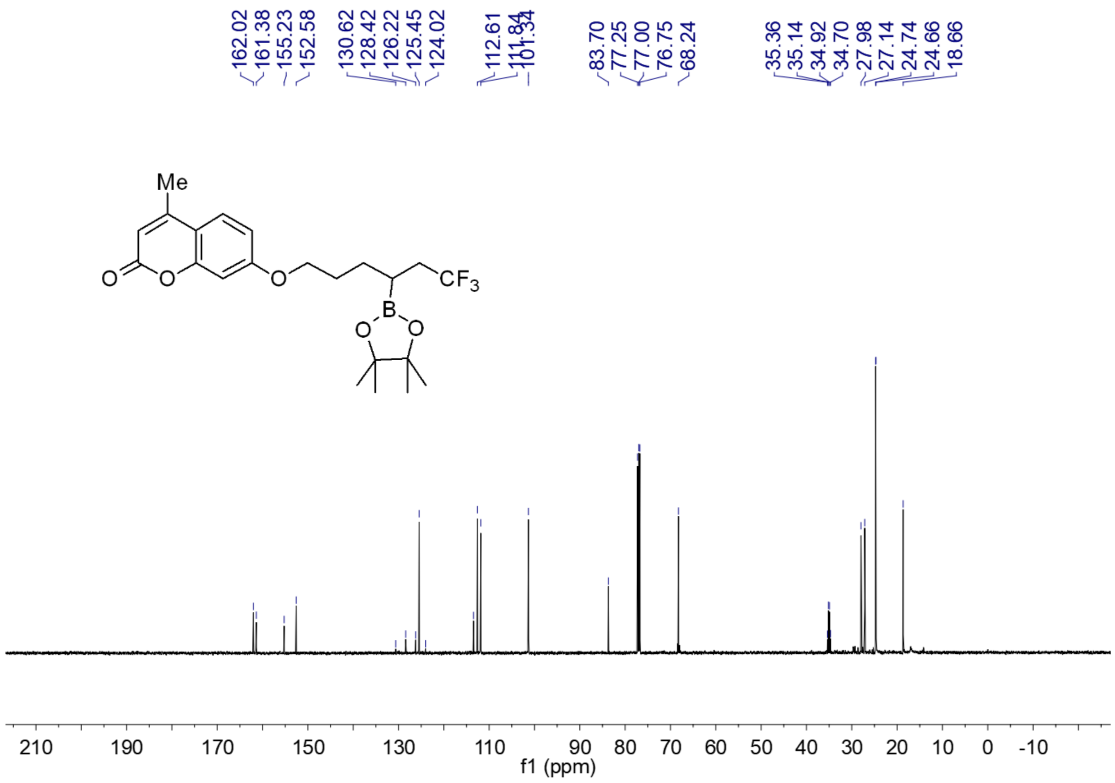

Supplementary Figure 150. ^{13}C NMR Spectra of product **5m**

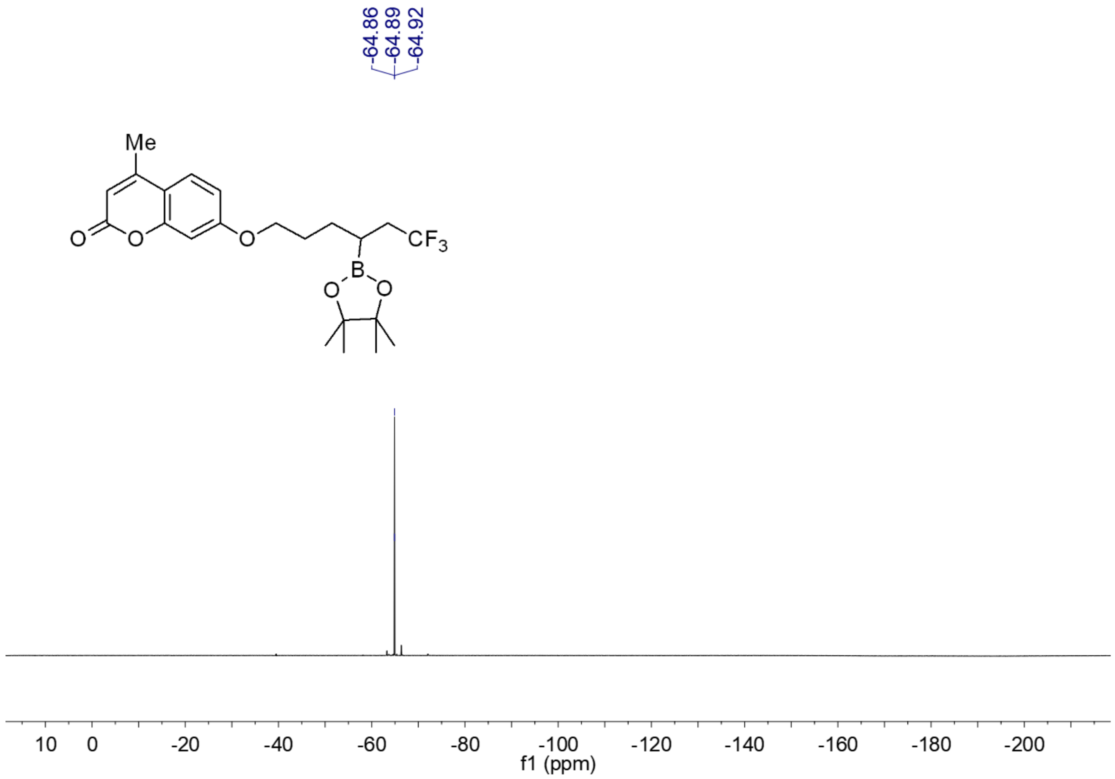

Supplementary Figure 151. ^{1}F NMR Spectra of product **5m**

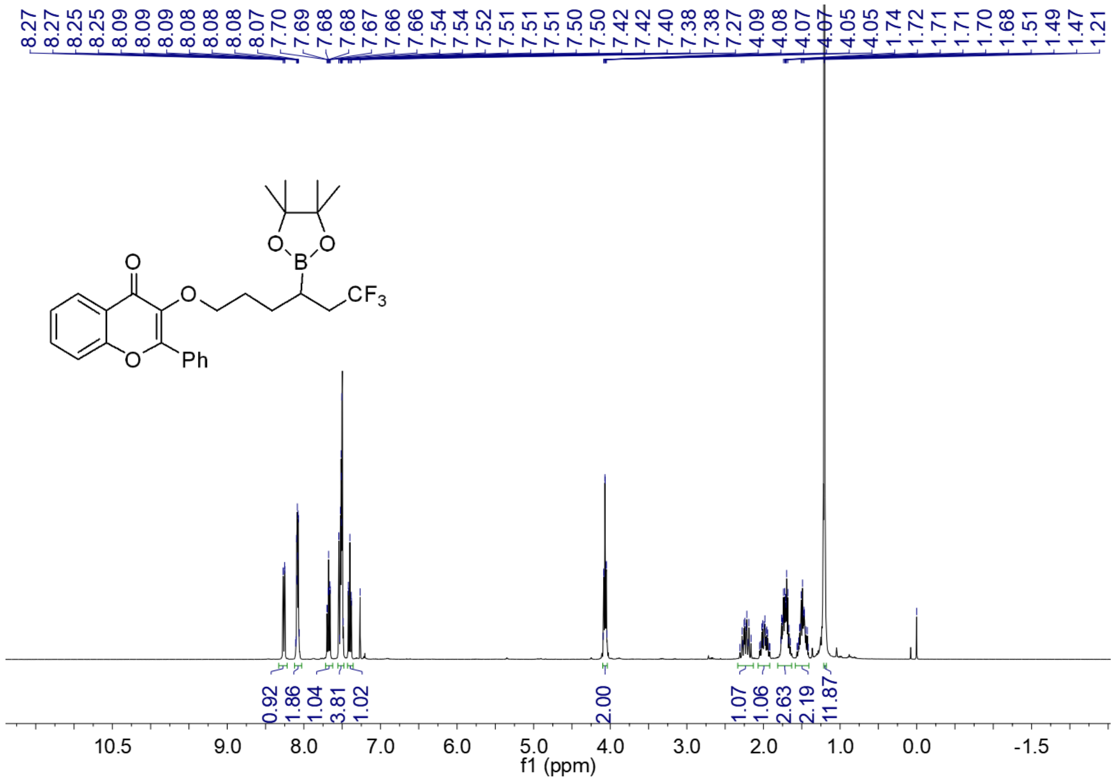

Supplementary Figure 152. ^{1}H NMR Spectra of product **5n**

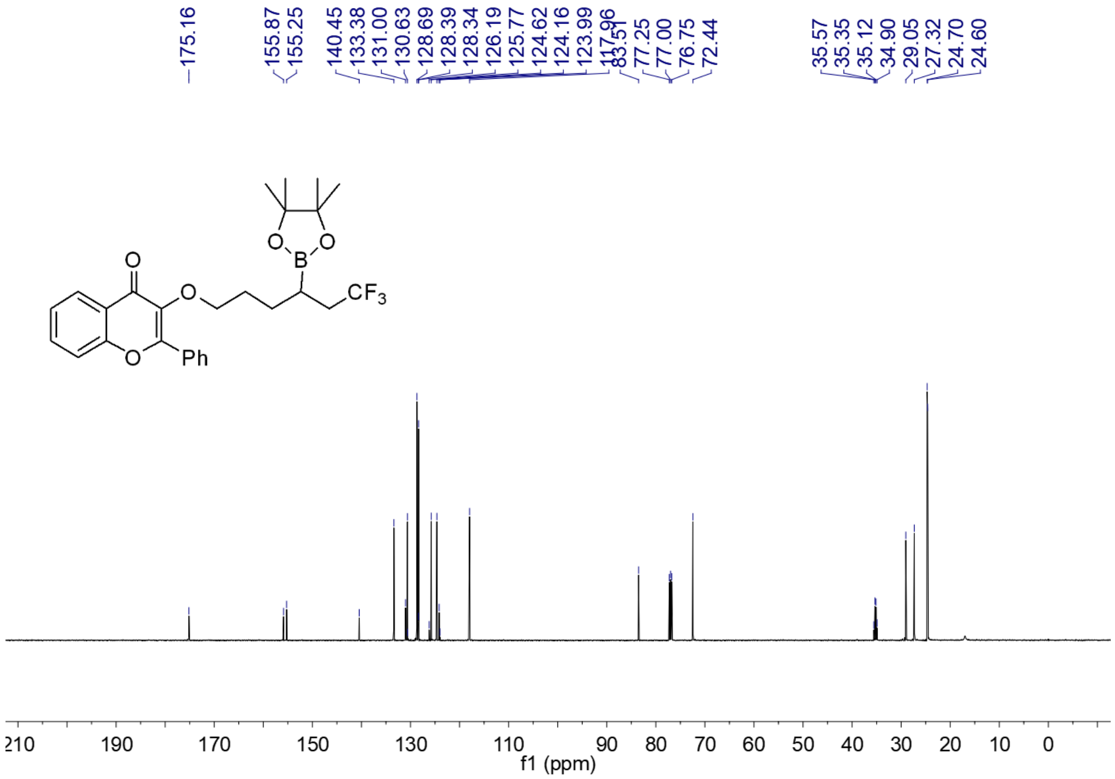

Supplementary Figure 153. ^{13}C NMR Spectra of product **5n**

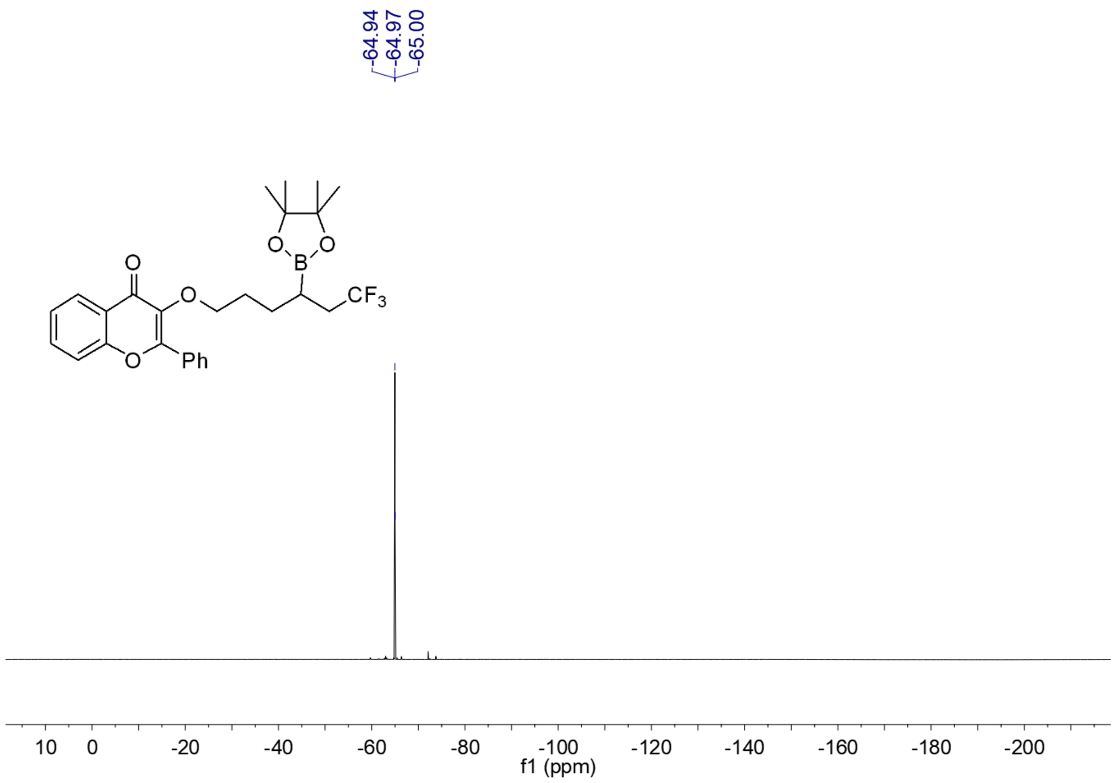

Supplementary Figure 154. ^{19}F NMR Spectra of product **5n**

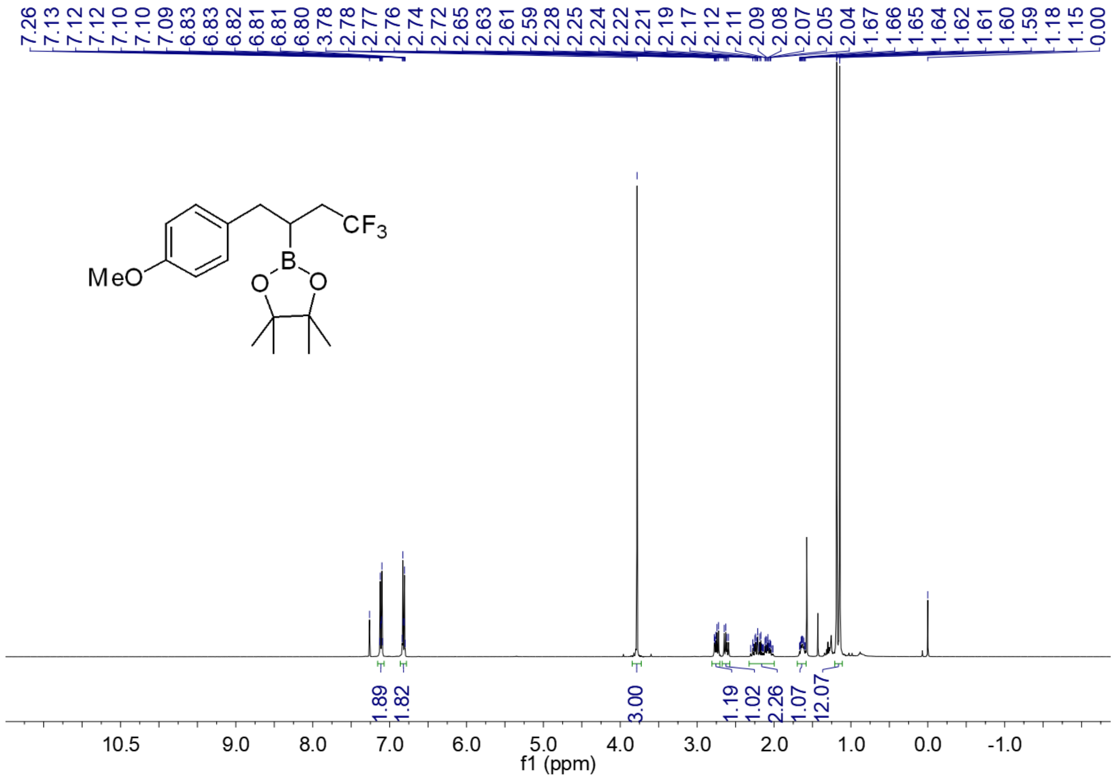

Supplementary Figure 155. ^{1}H NMR Spectra of product **5o**

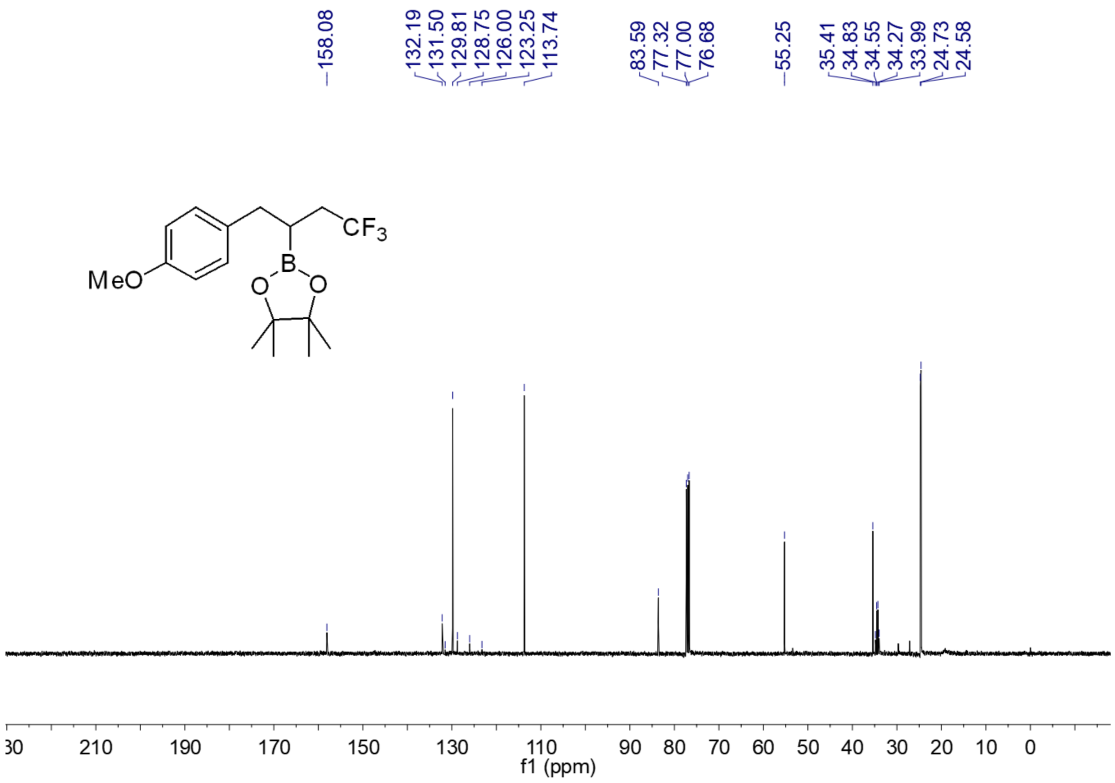

Supplementary Figure 156. ^{13}C NMR Spectra of product **5o**

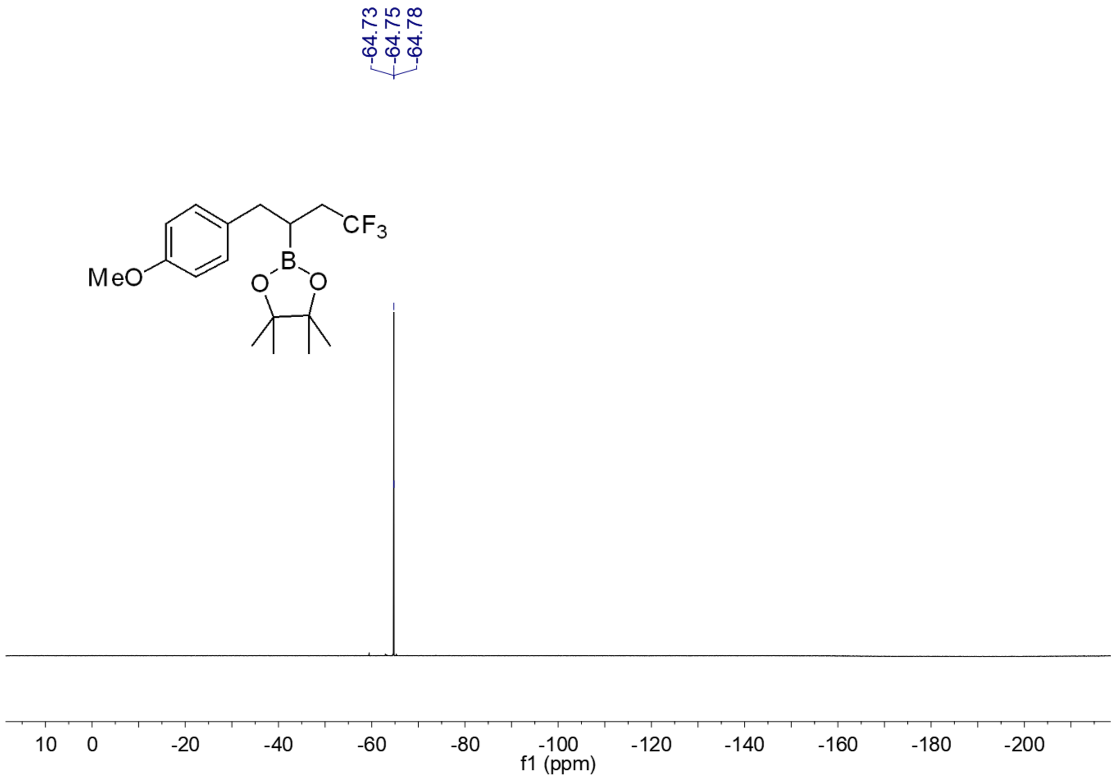

Supplementary Figure 157. ^{19}F NMR Spectra of product **5o**

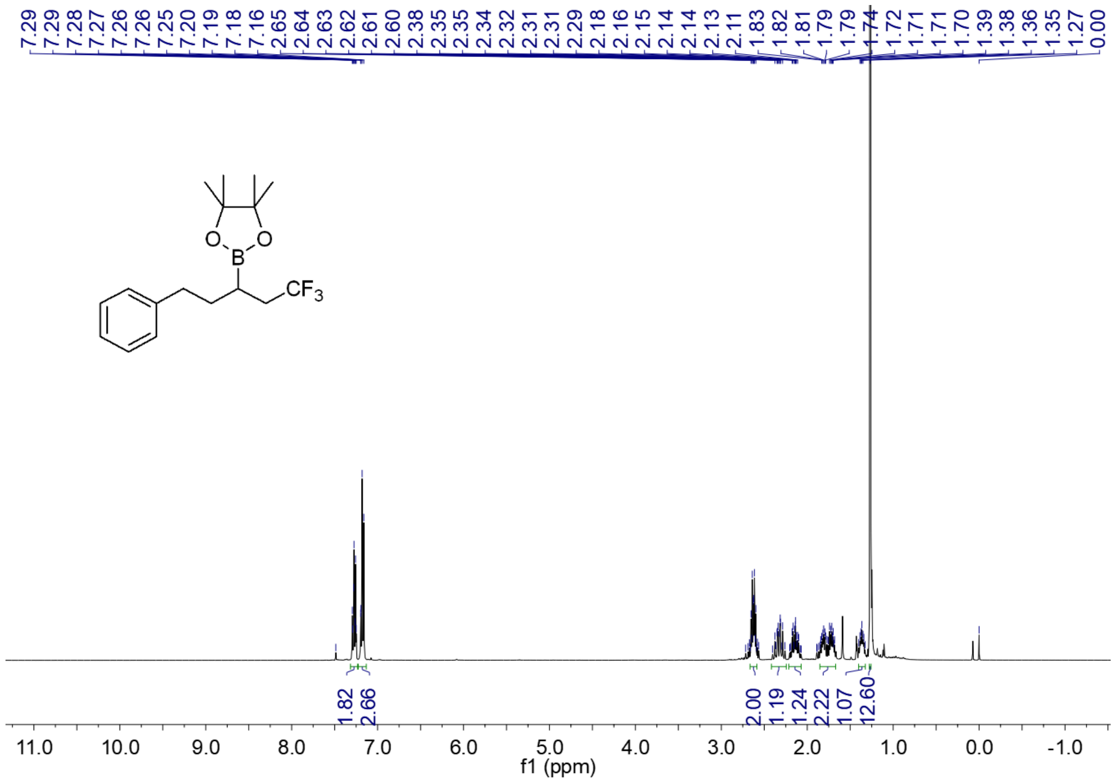

Supplementary Figure 158. ^{1}H NMR Spectra of product **5p**

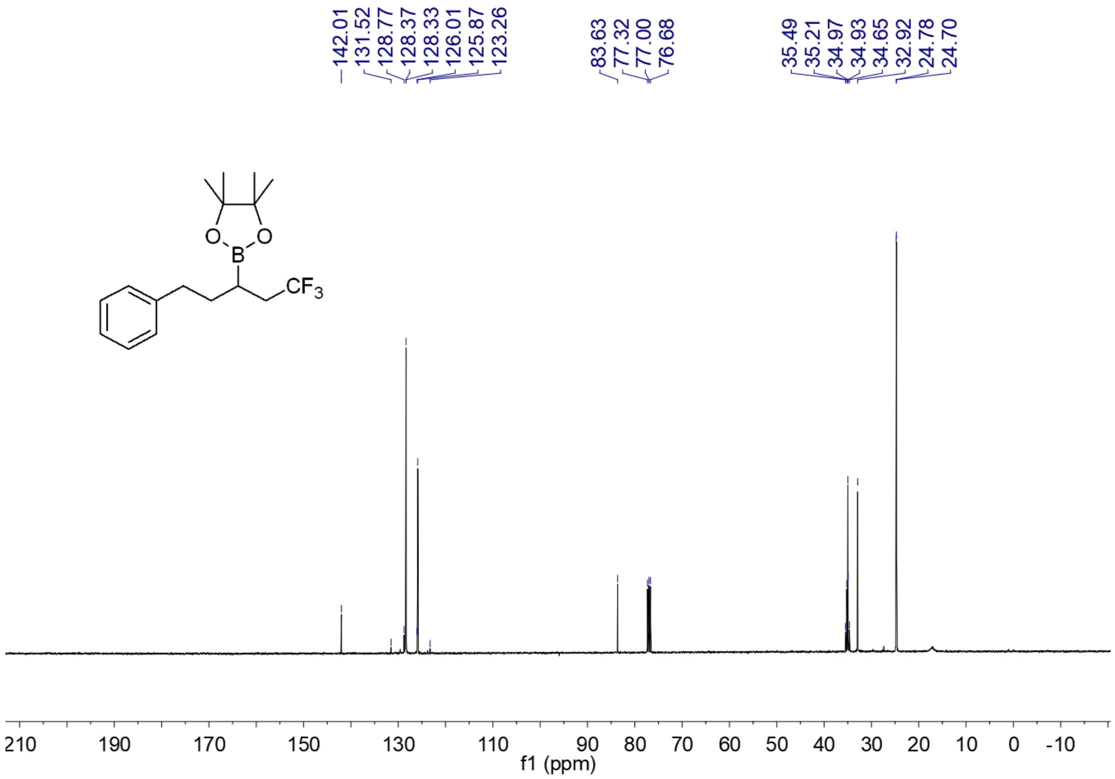

Supplementary Figure 159. ^{13}C NMR Spectra of product **5p**

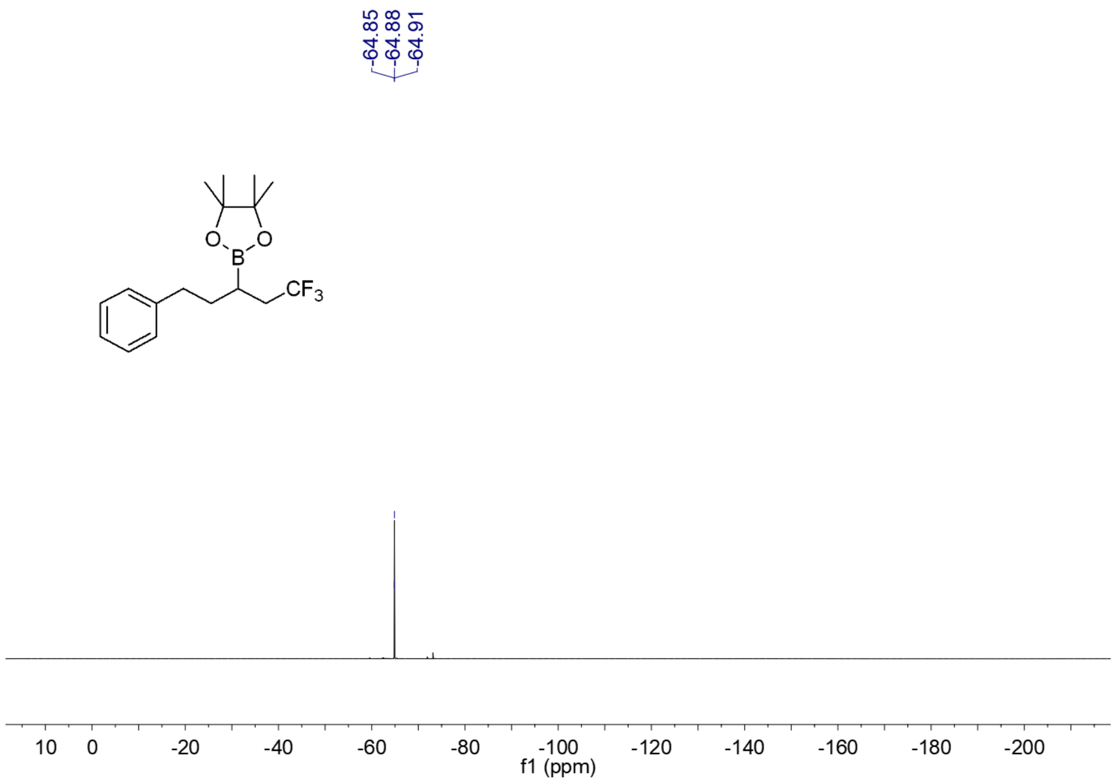

Supplementary Figure 160. ^{19}F NMR Spectra of product **5p**

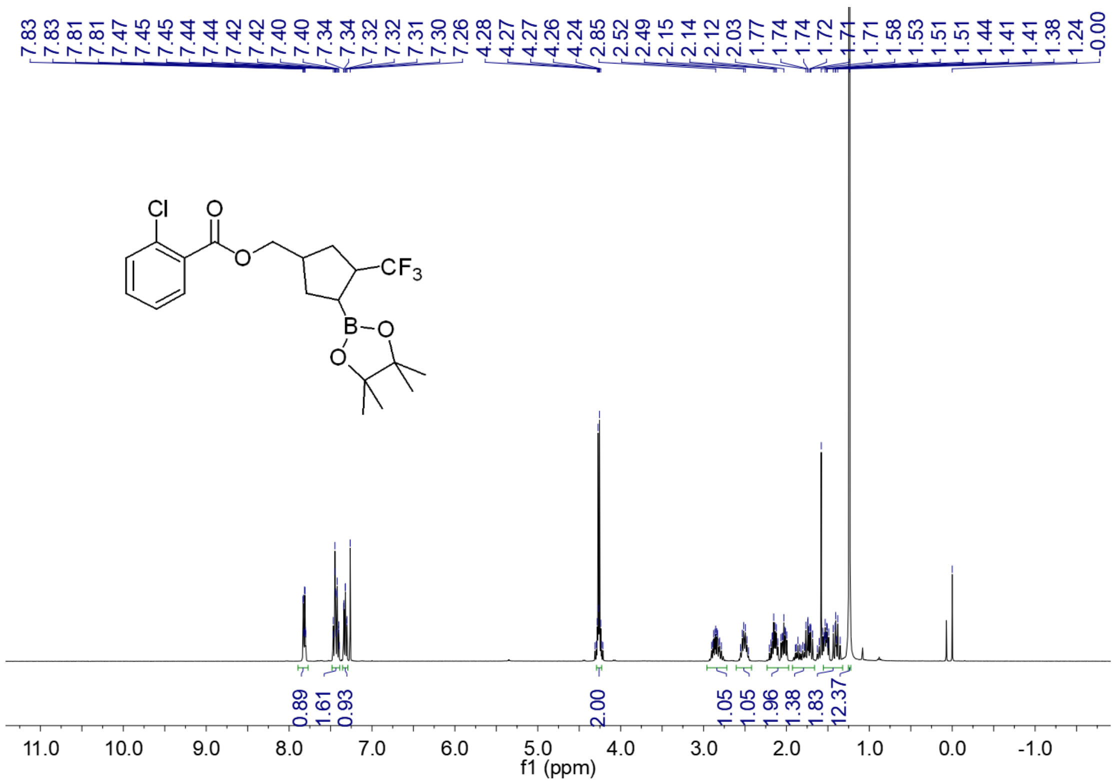

Supplementary Figure 161. ^{1}H NMR Spectra of product **5q**

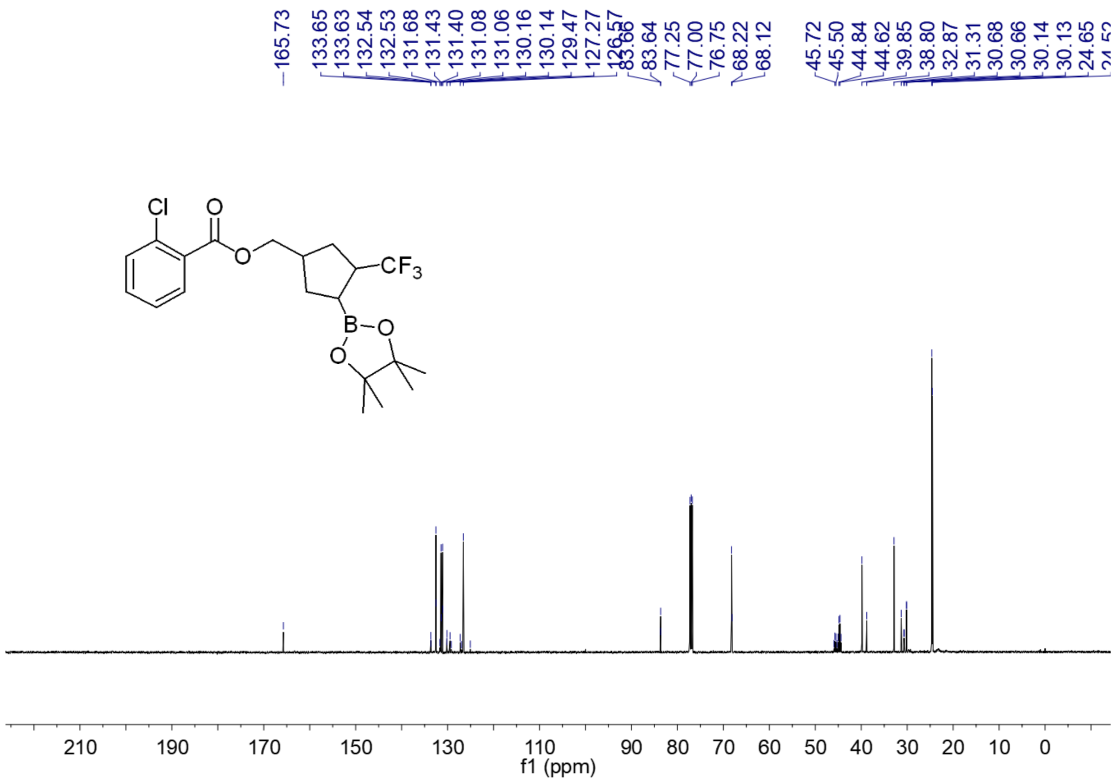

Supplementary Figure 162. ^{13}C NMR Spectra of product **5q**

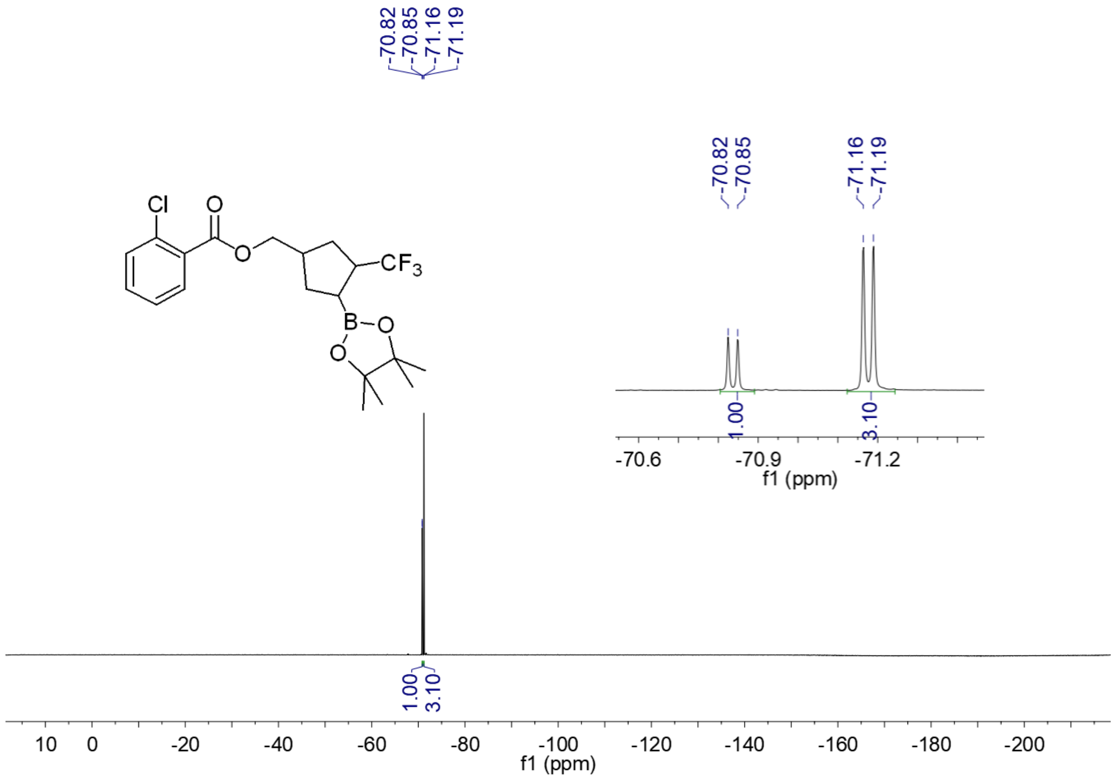

Supplementary Figure 163. ^{19}F NMR Spectra of product **5q**

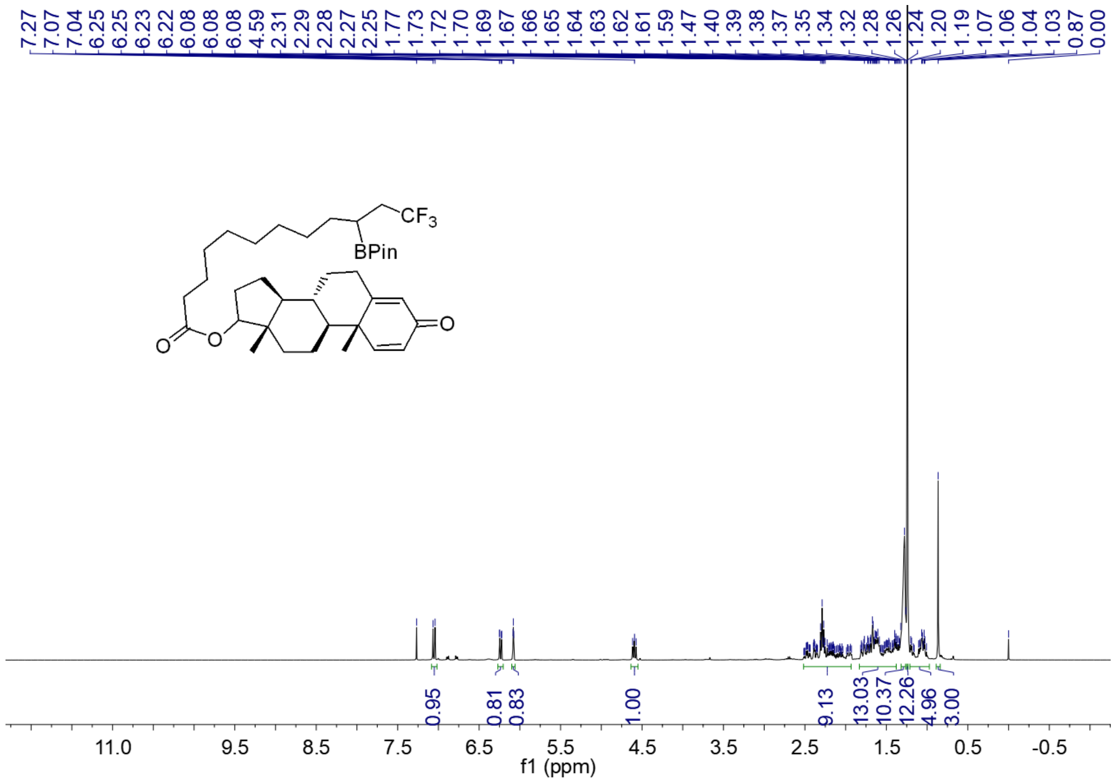

Supplementary Figure 164. ^{1}H NMR Spectra of product **5r**

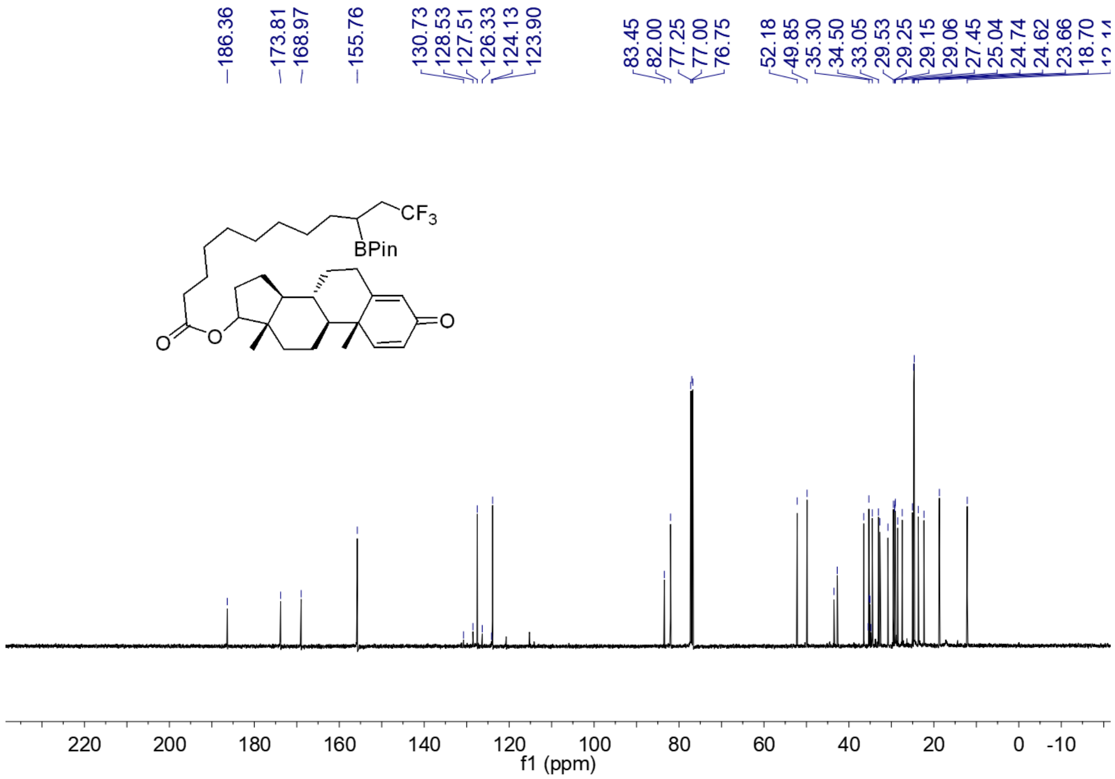

Supplementary Figure 165. ^{13}C NMR Spectra of product **5r**

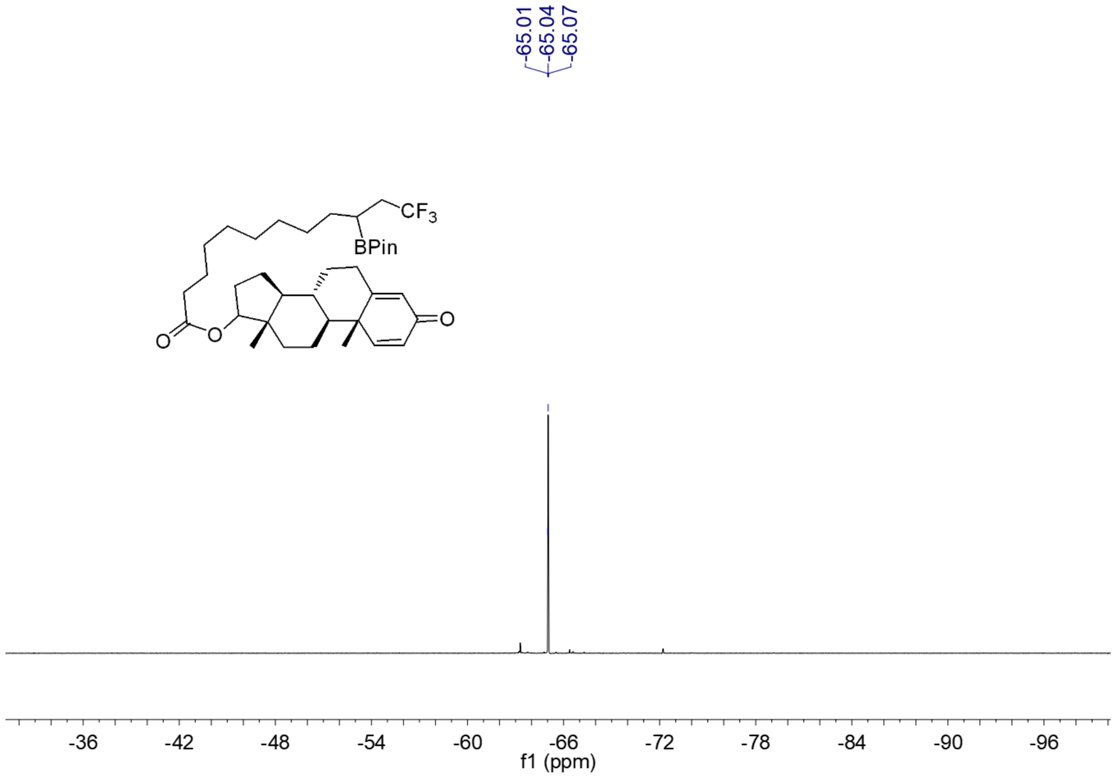

Supplementary Figure 166. ^{19}F NMR Spectra of product **5r**

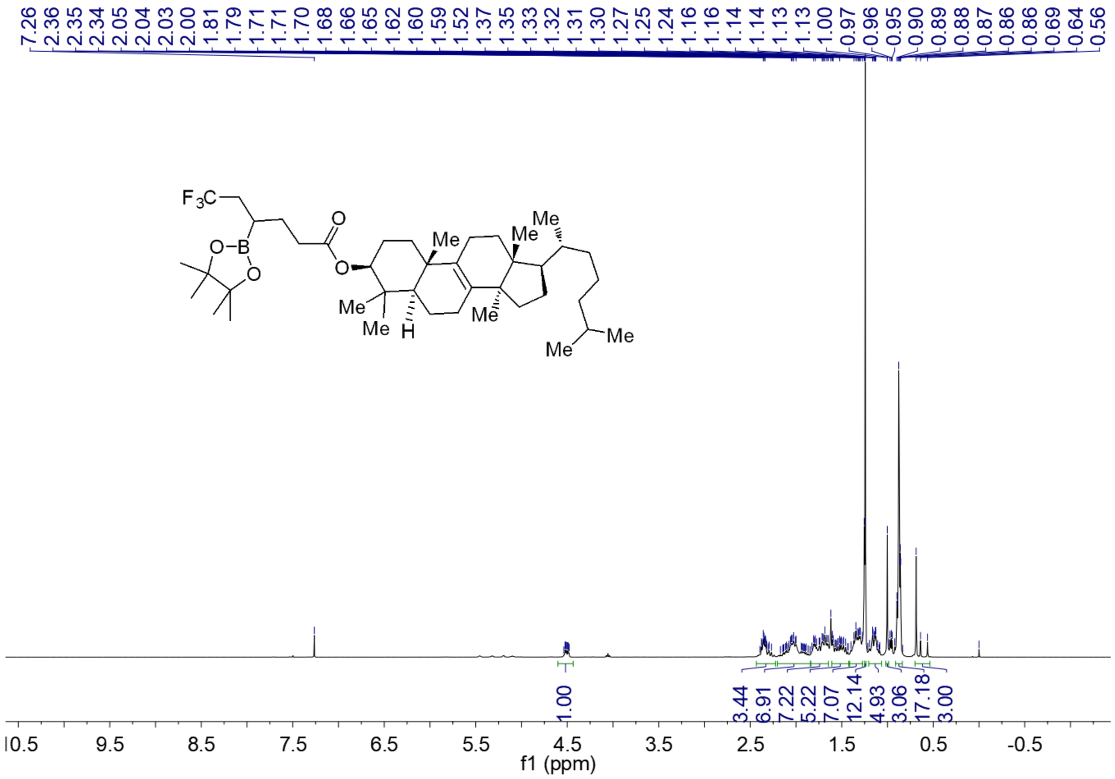

Supplementary Figure 167. ^{1}H NMR Spectra of product **5s**

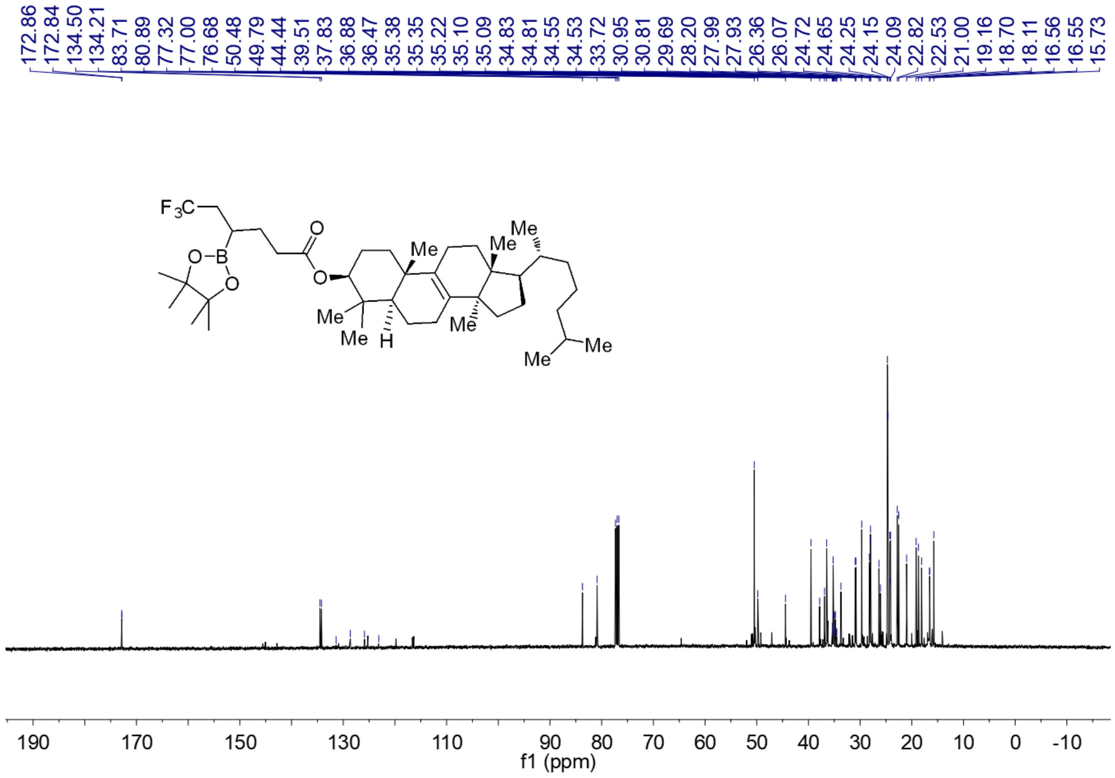

Supplementary Figure 168. ^{13}C NMR Spectra of product **5s**

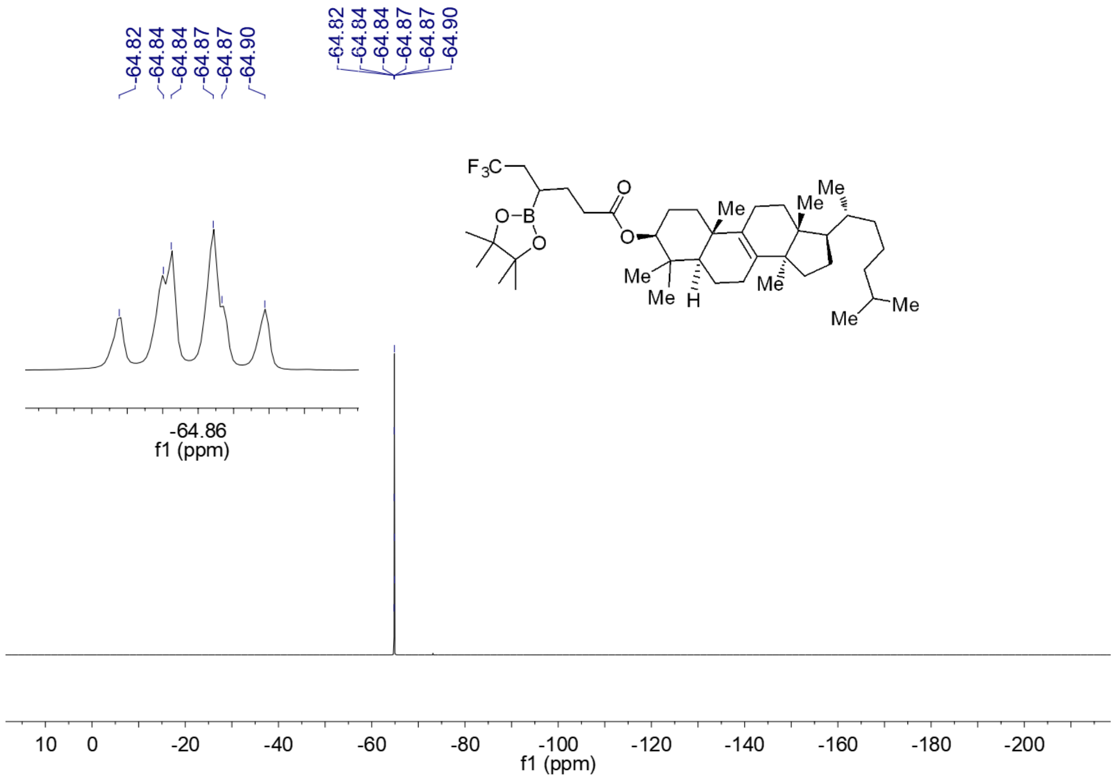

Supplementary Figure 169. ^{19}F NMR Spectra of product **5s**

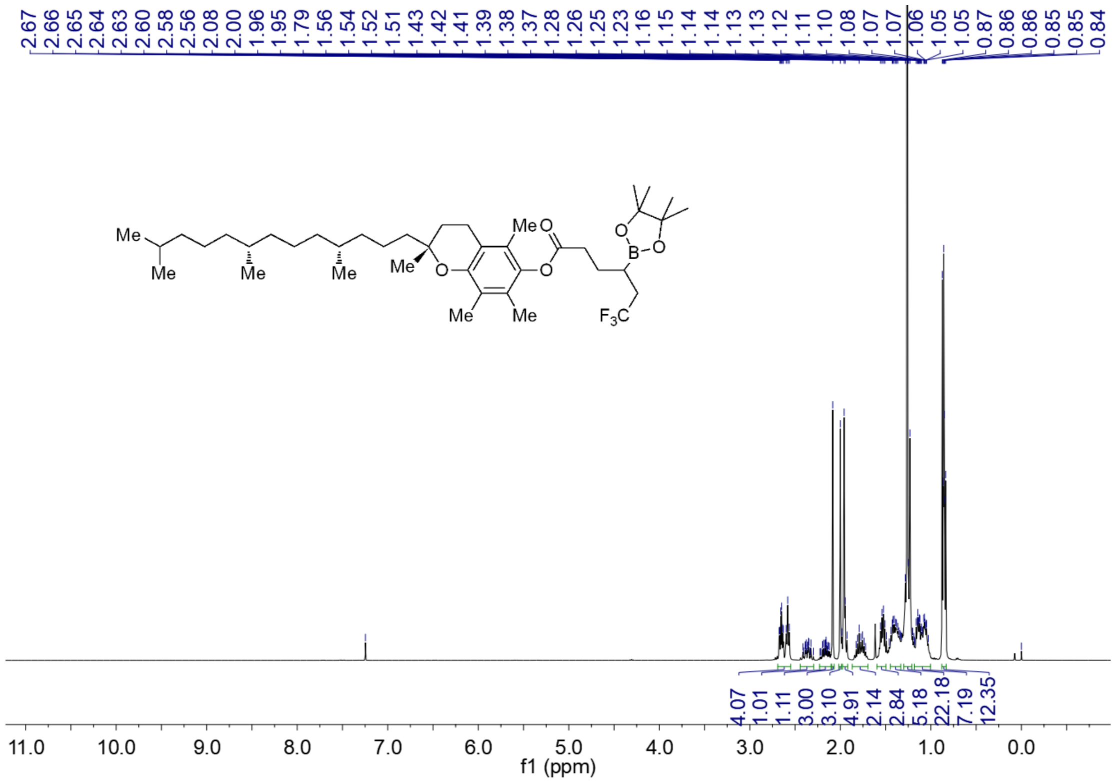

Supplementary Figure 170. ^{1}H NMR Spectra of product **5t**

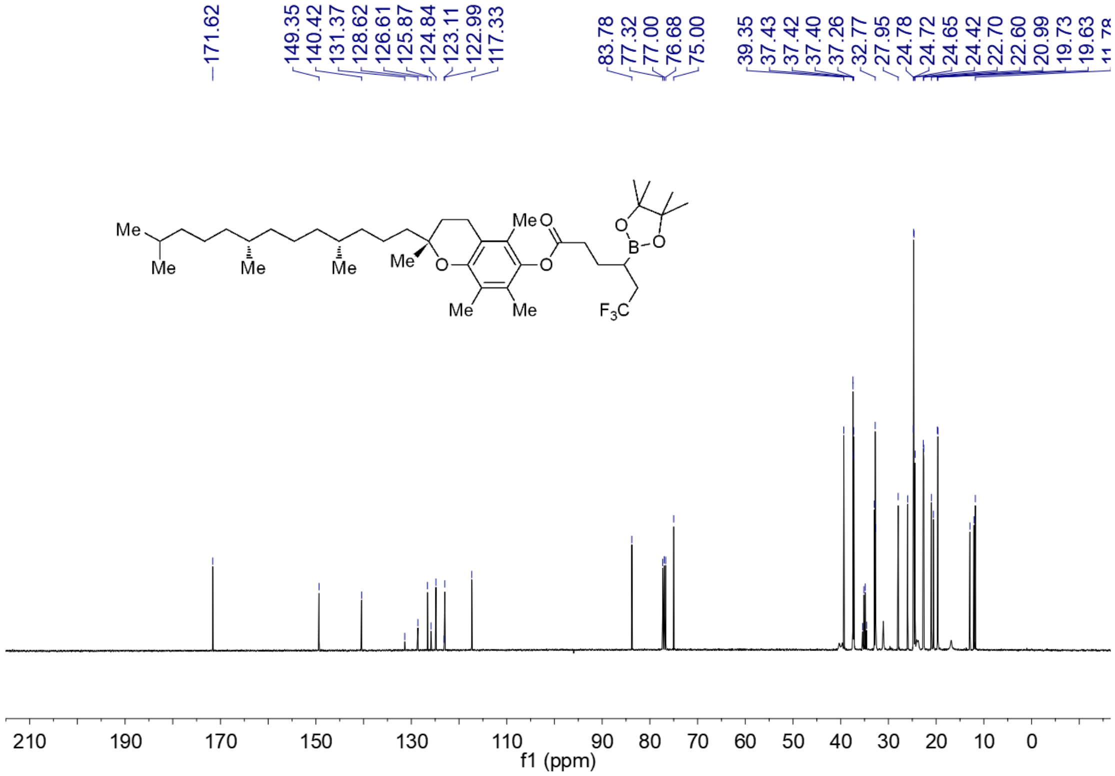

Supplementary Figure 171. ^{13}C NMR Spectra of product **5t**

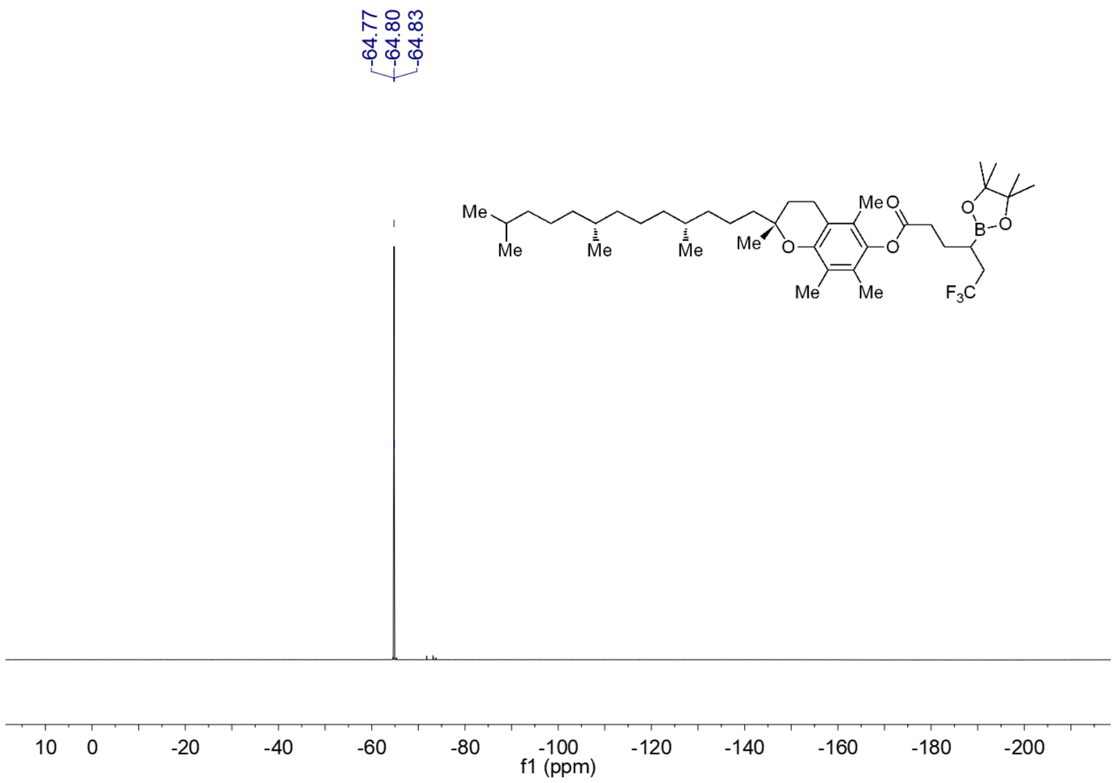

Supplementary Figure 172. ^{19}F NMR Spectra of product **5t**

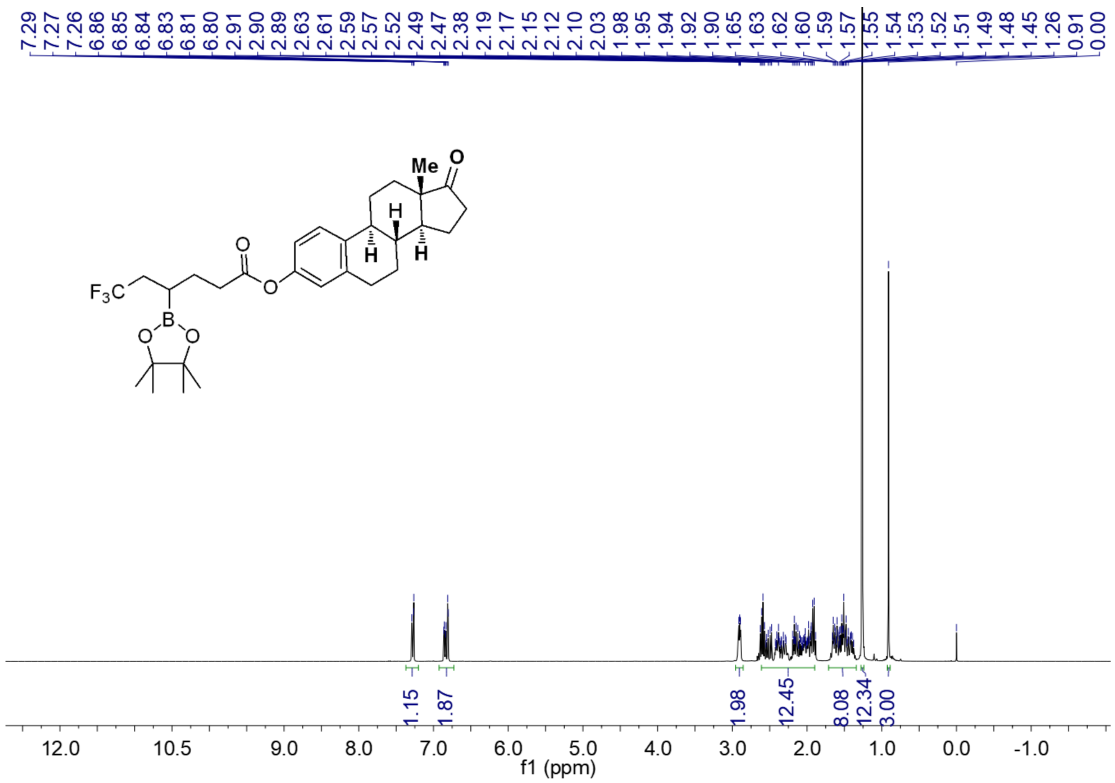

Supplementary Figure 173. ^{1}H NMR Spectra of product **5u**

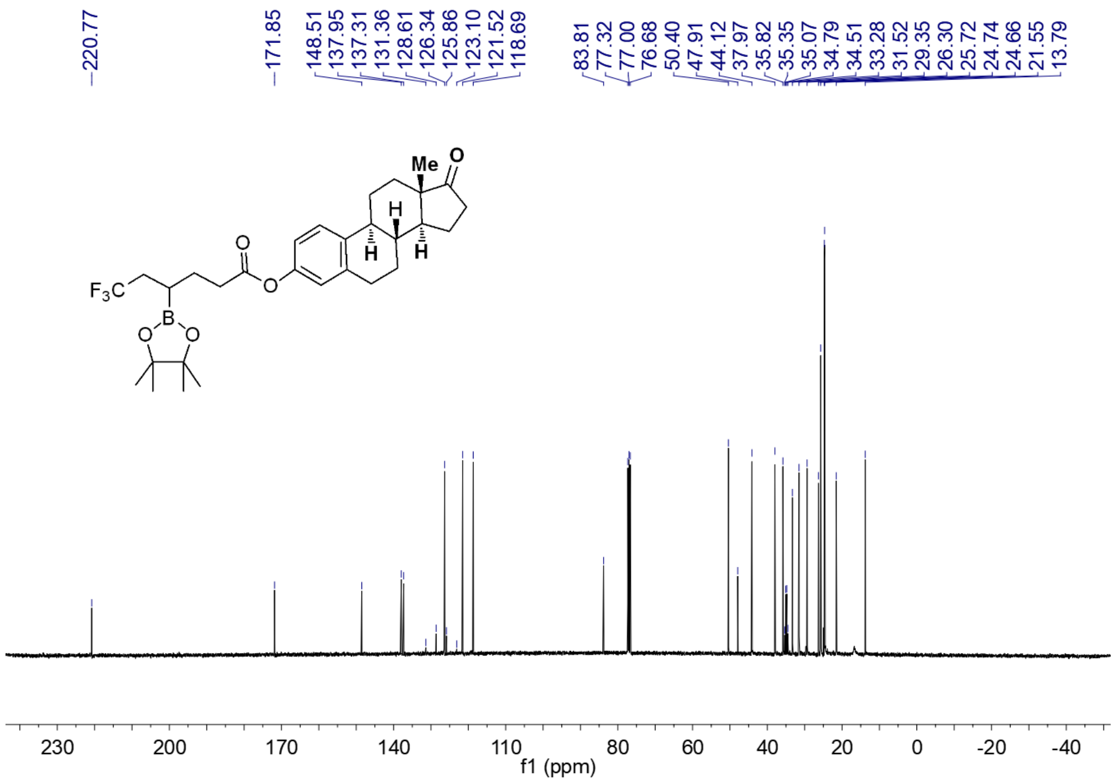

Supplementary Figure 174. ^{13}C NMR Spectra of product **5u**

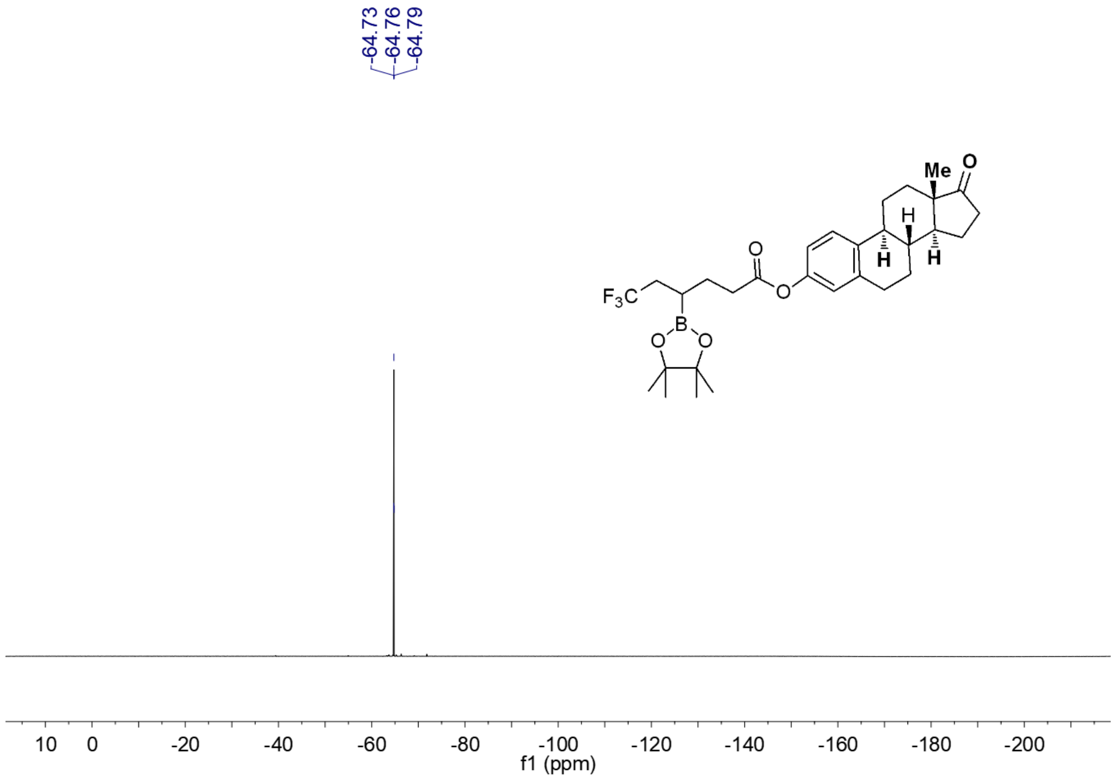

Supplementary Figure 175. ^{19}F NMR Spectra of product **5u**

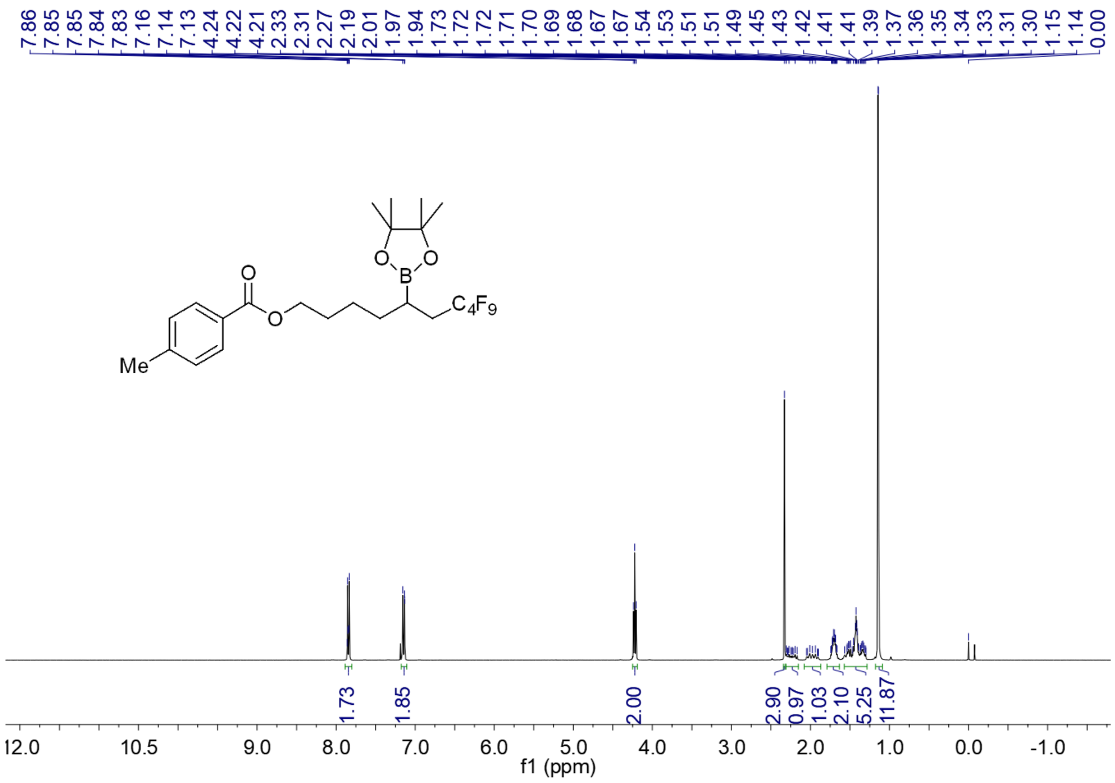

Supplementary Figure 176. ^{1}H NMR Spectra of product **5v**

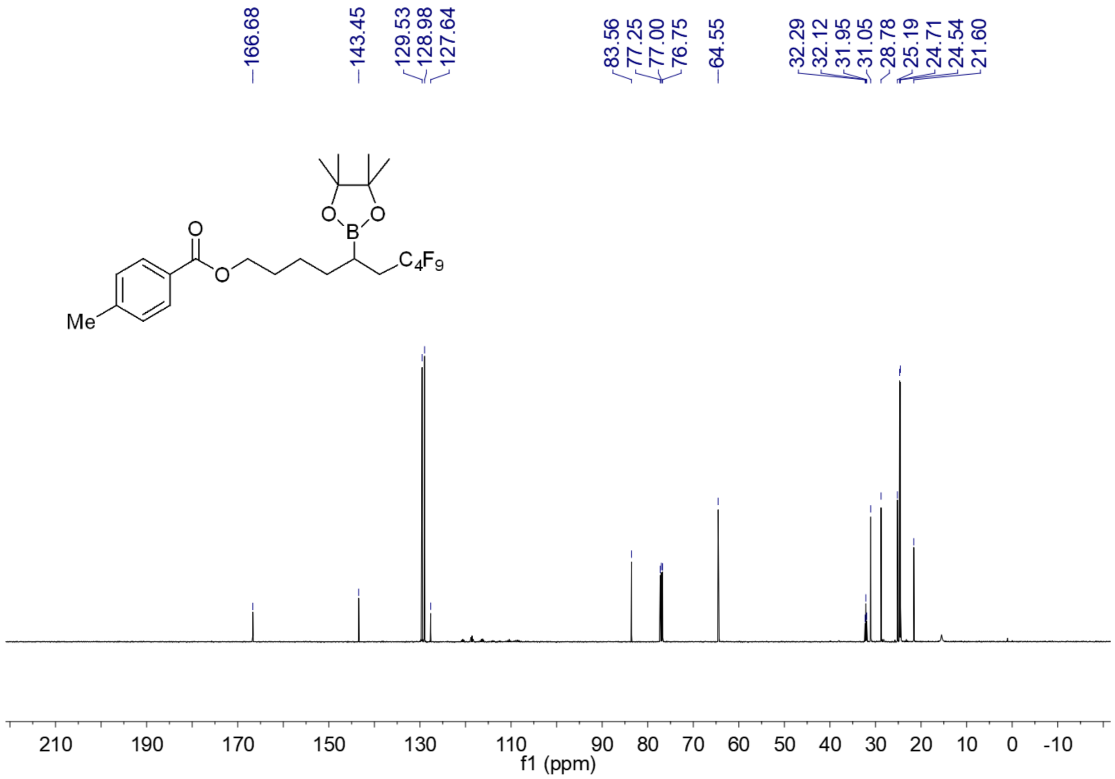

Supplementary Figure 177. ^{13}C NMR Spectra of product **5v**

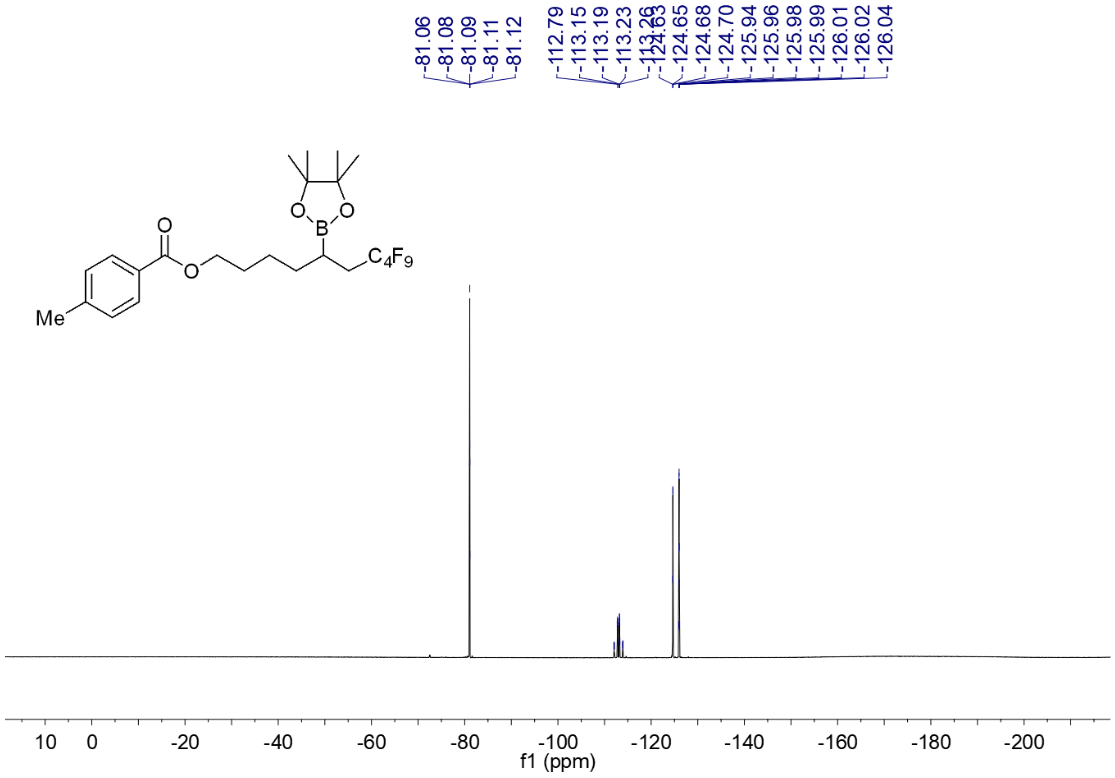

Supplementary Figure 178. ^{19}F NMR Spectra of product **5v**

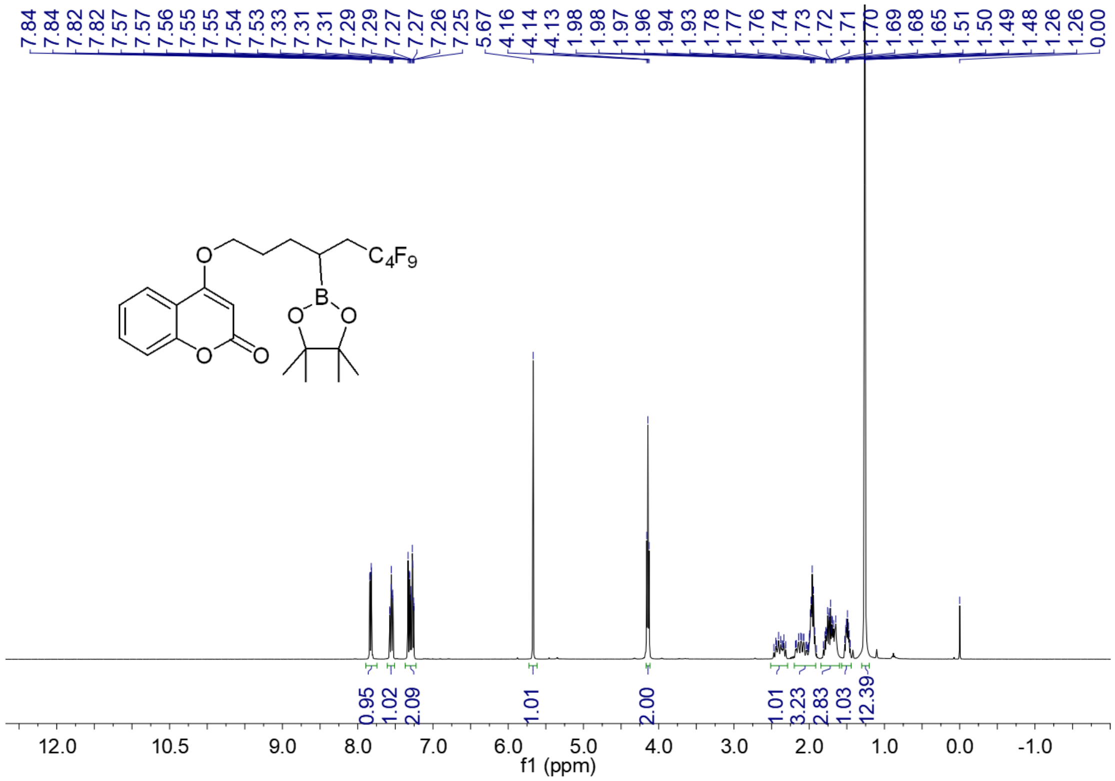

Supplementary Figure 179. ^{1}H NMR Spectra of product **5w**

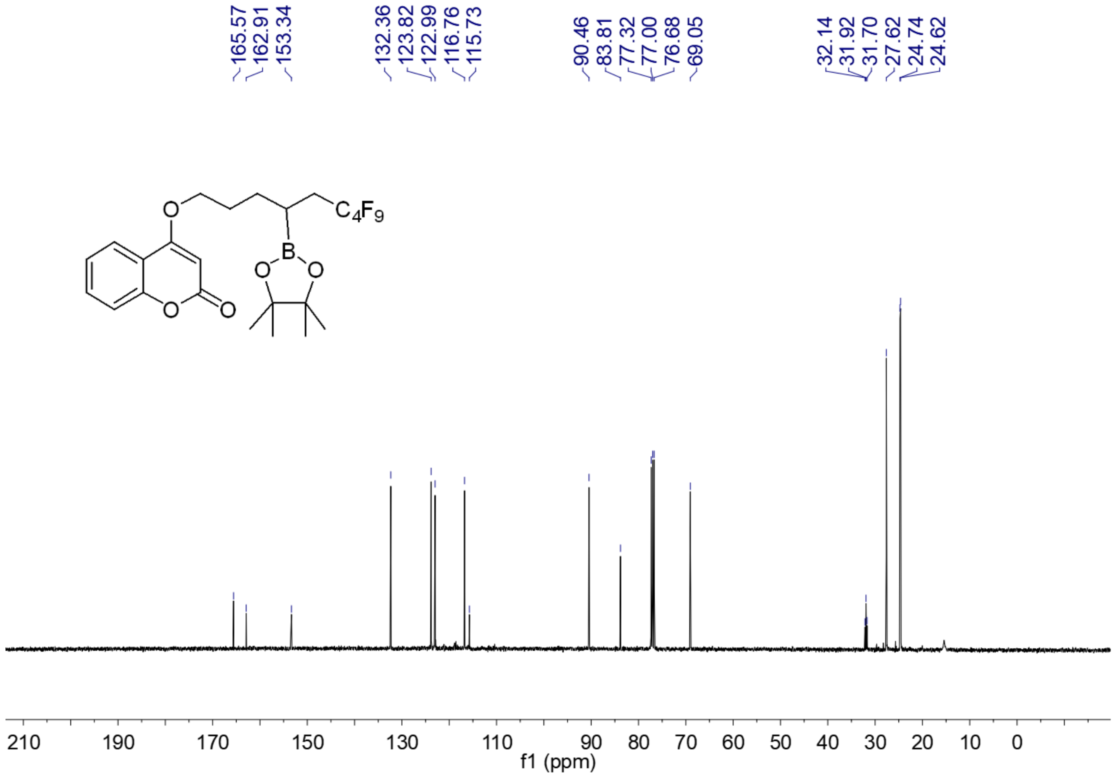

Supplementary Figure 180. ^{13}C NMR Spectra of product **5w**

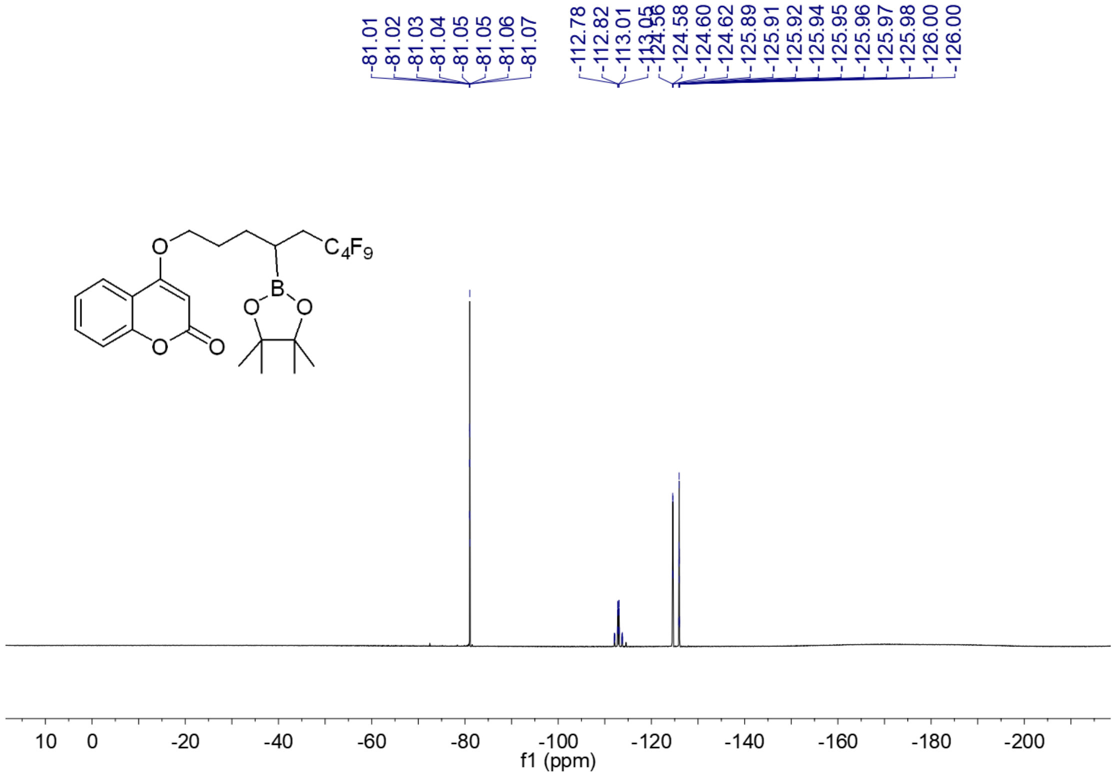

Supplementary Figure 181. ^{19}F NMR Spectra of product **5w**

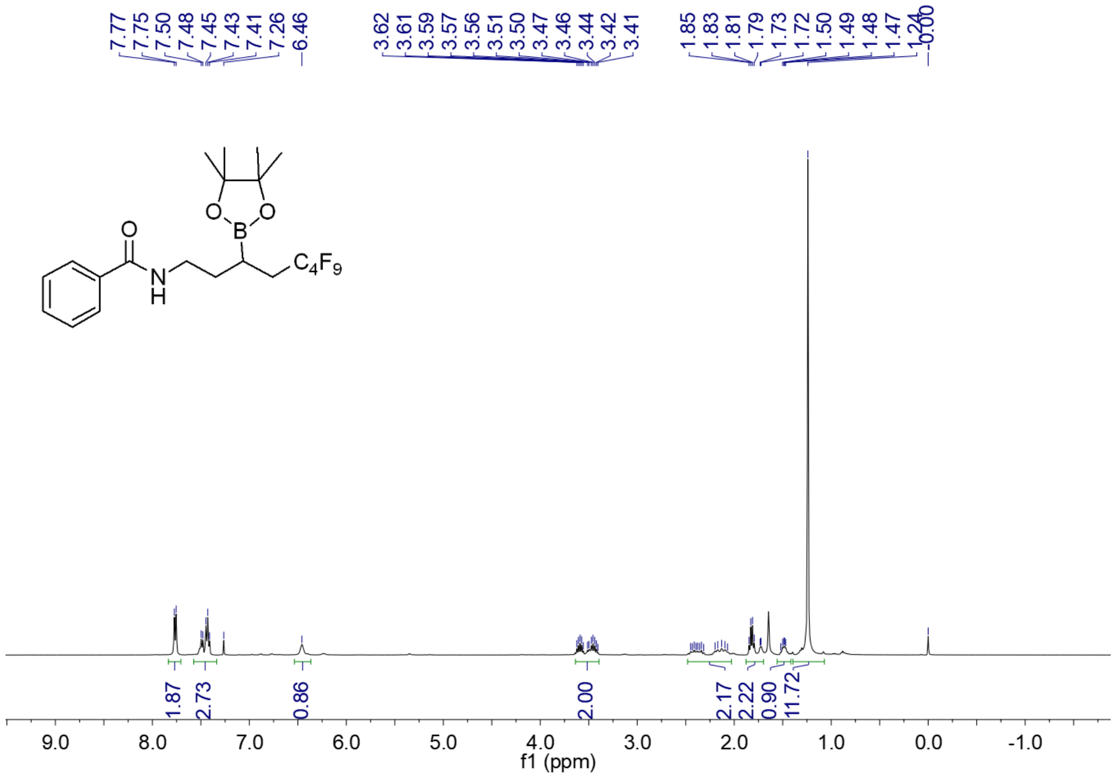

Supplementary Figure 182. ^{1}H NMR Spectra of product **5x**

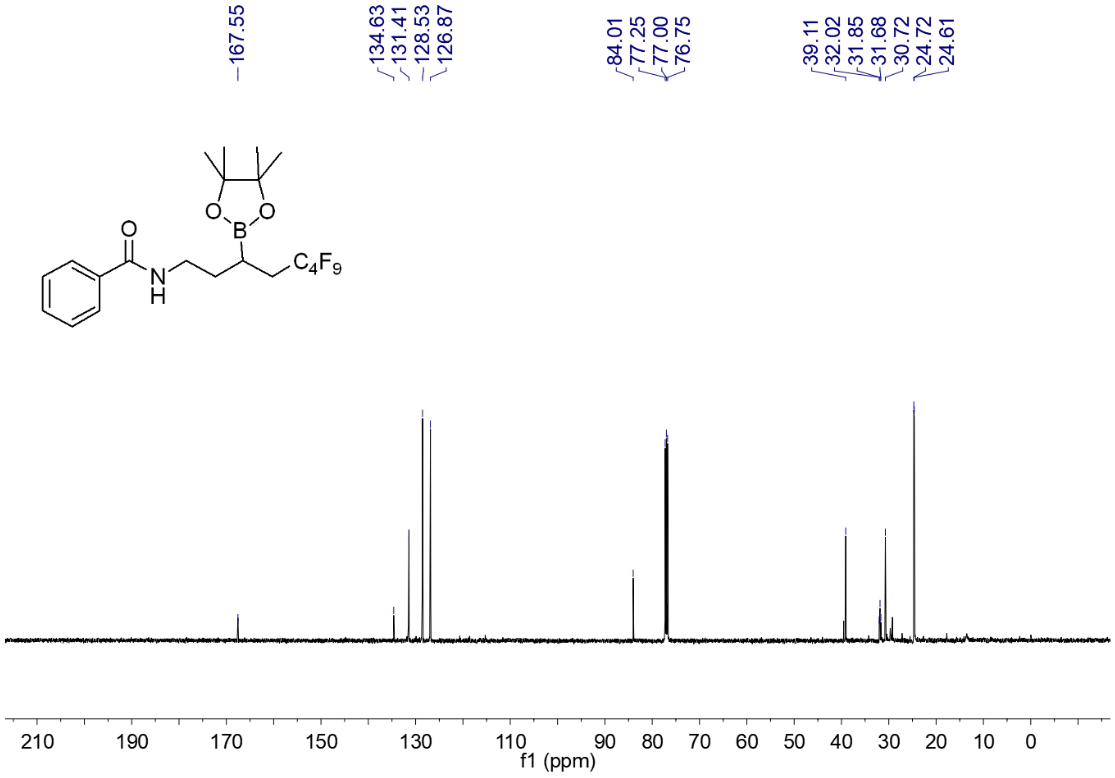

Supplementary Figure 183. ^{13}C NMR Spectra of product **5x**

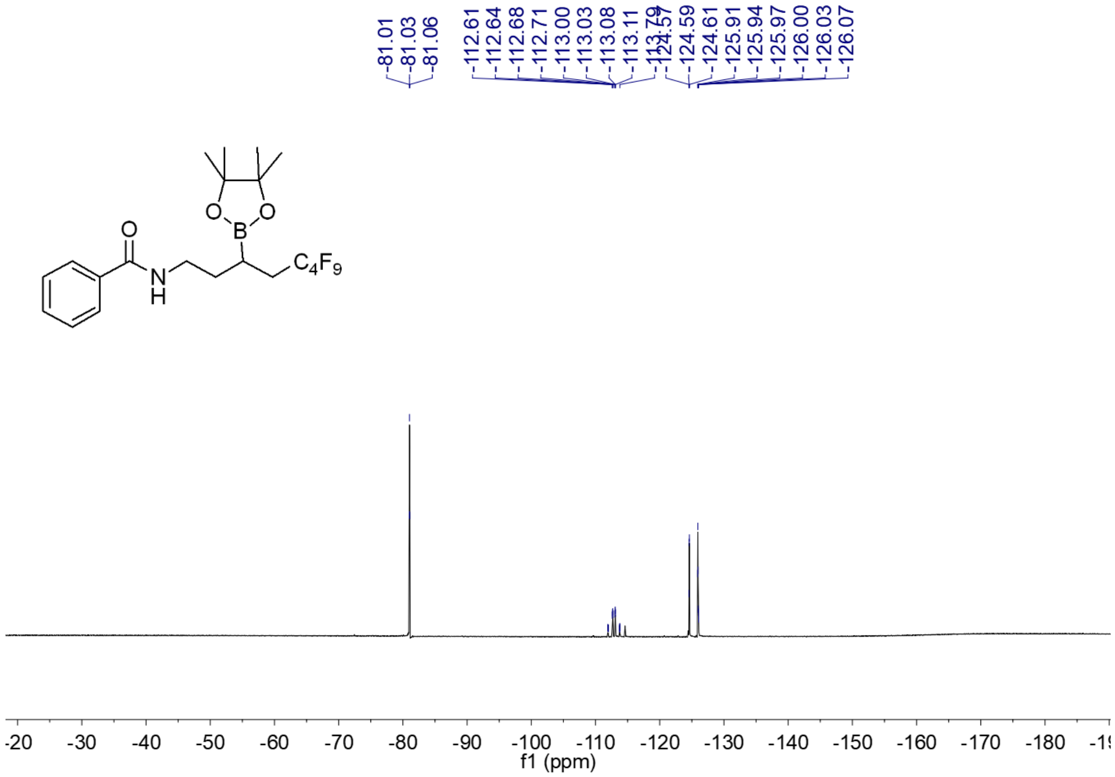

Supplementary Figure 184. ^{19}F NMR Spectra of product **5x**

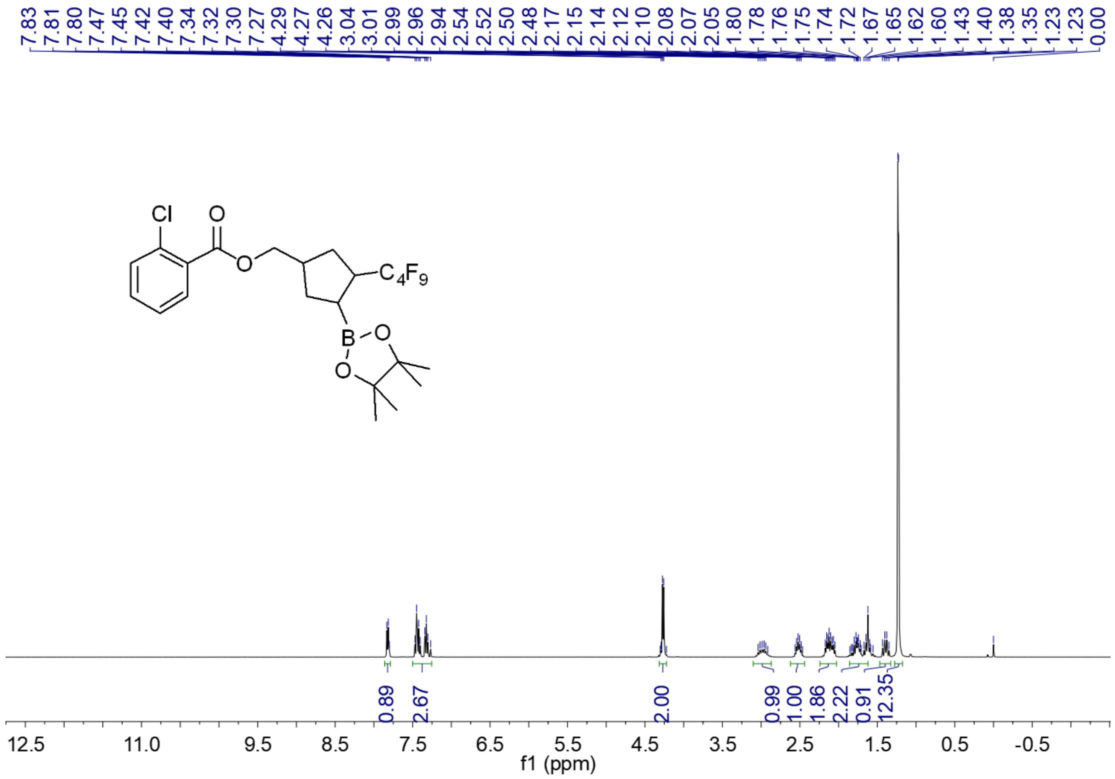

Supplementary Figure 185. ^{1}H NMR Spectra of product **5y**

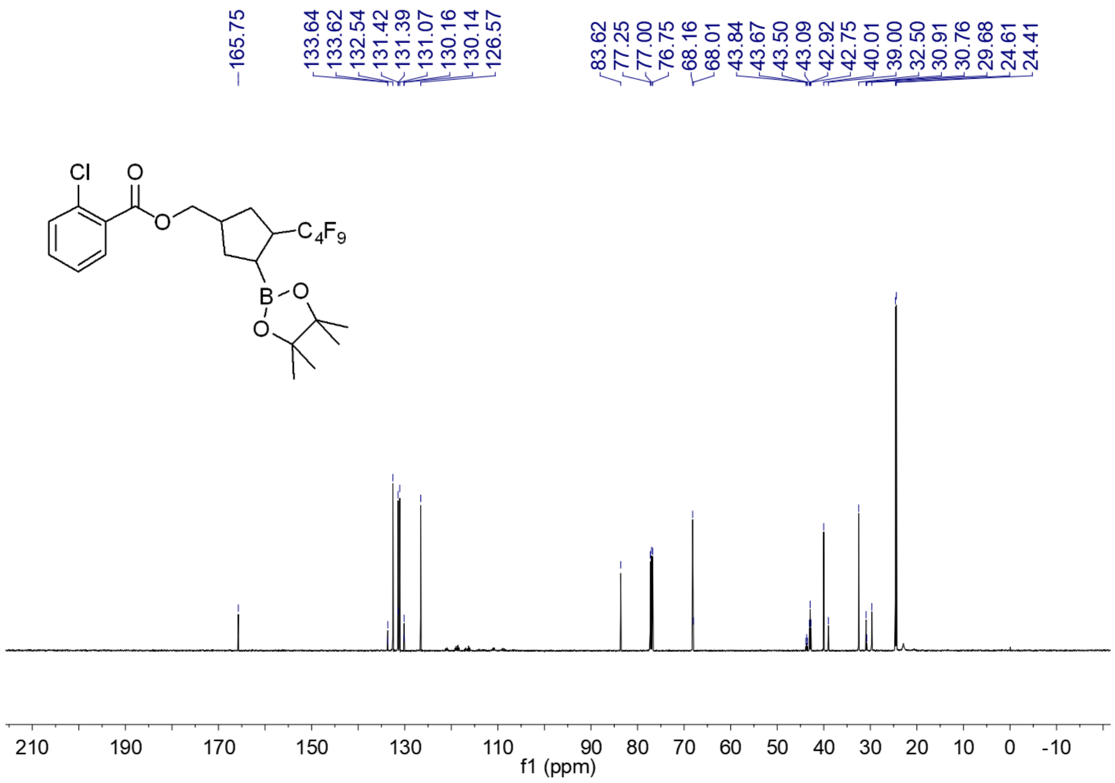

Supplementary Figure 186. ^{13}C NMR Spectra of product **5y**

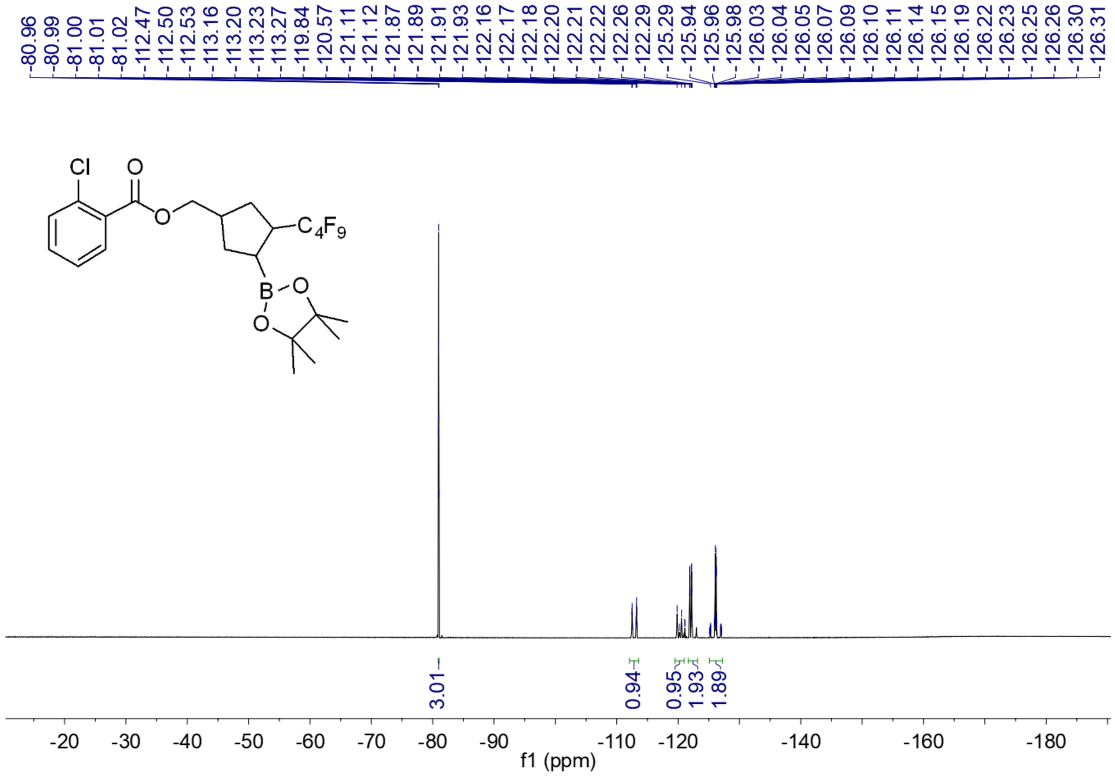

Supplementary Figure 187. ^{19}F NMR Spectra of product **5y**

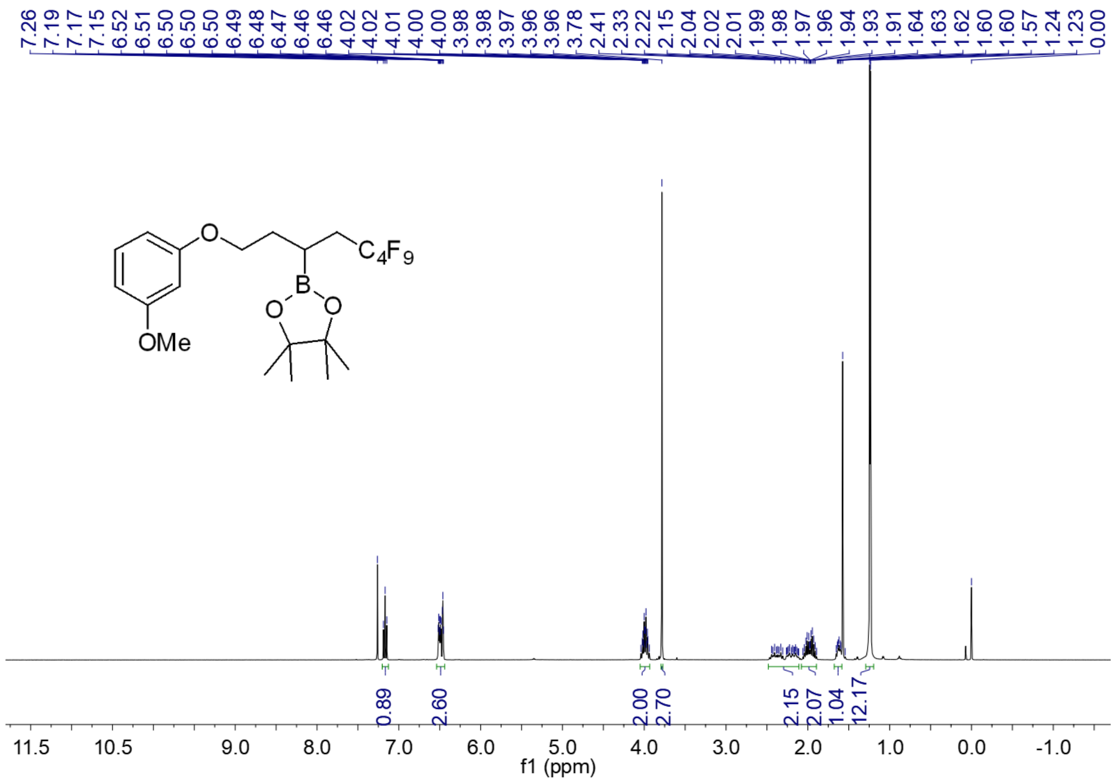

Supplementary Figure 188. ^{1}H NMR Spectra of product **5z**

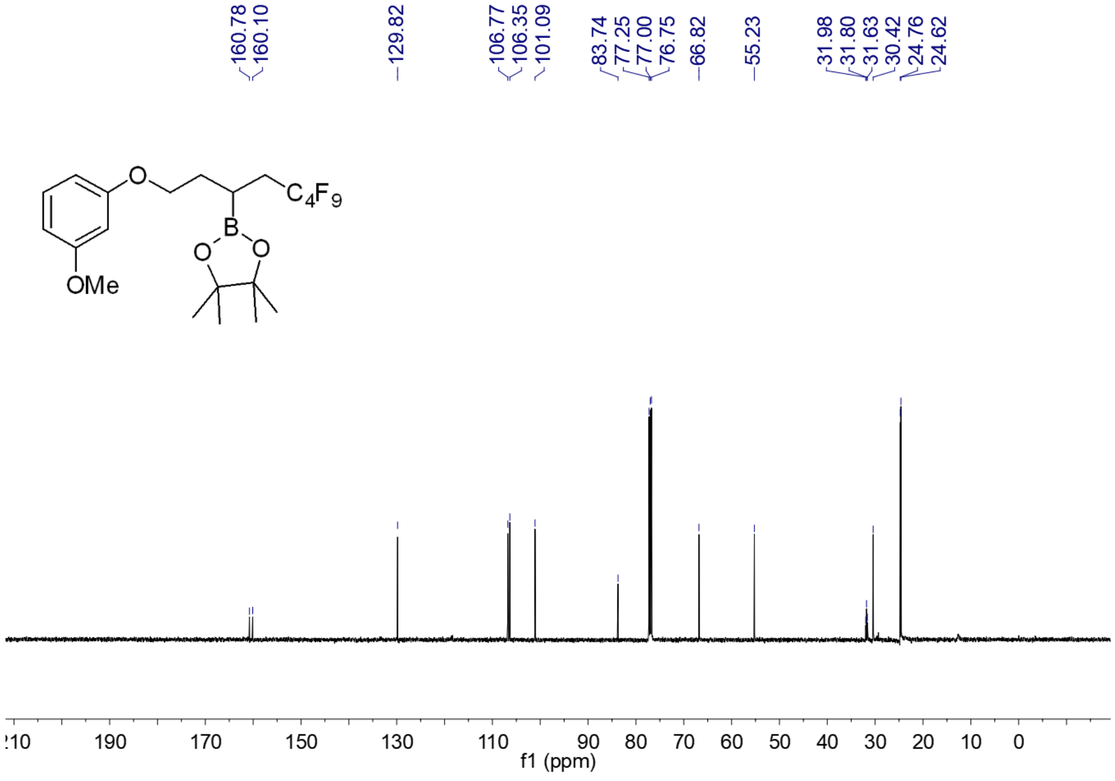

Supplementary Figure 189. ^{13}C NMR Spectra of product **5z**

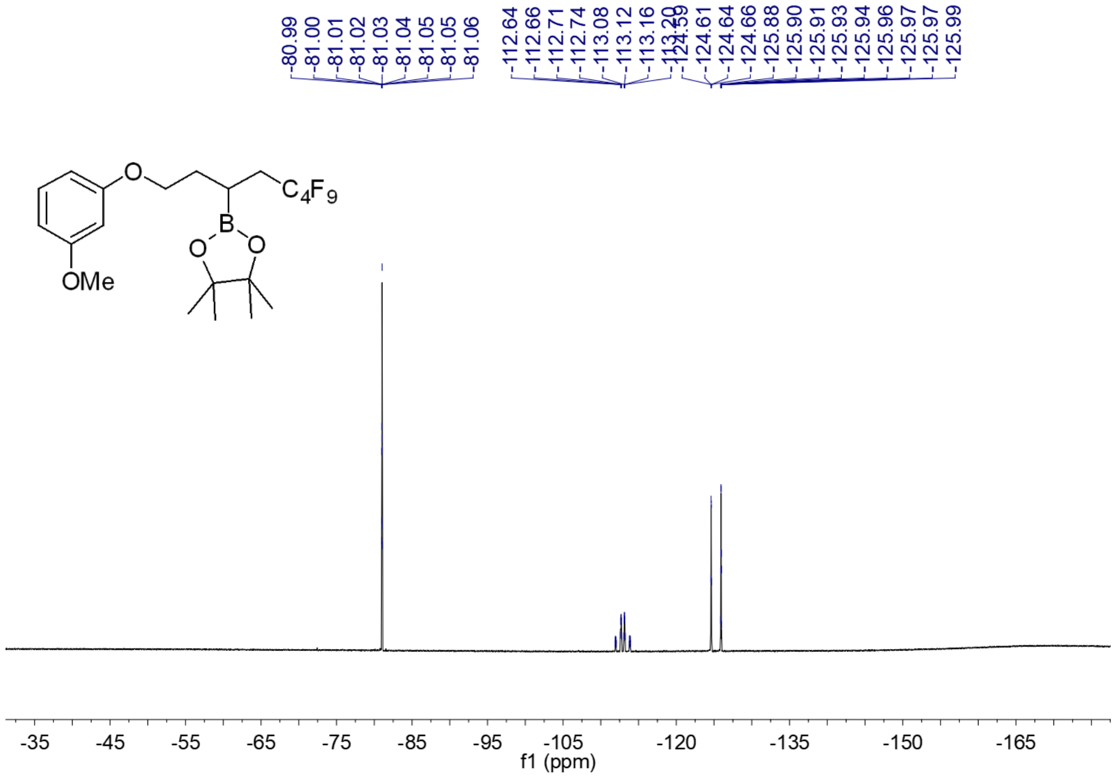

Supplementary Figure 190. ^{19}F NMR Spectra of product **5z**

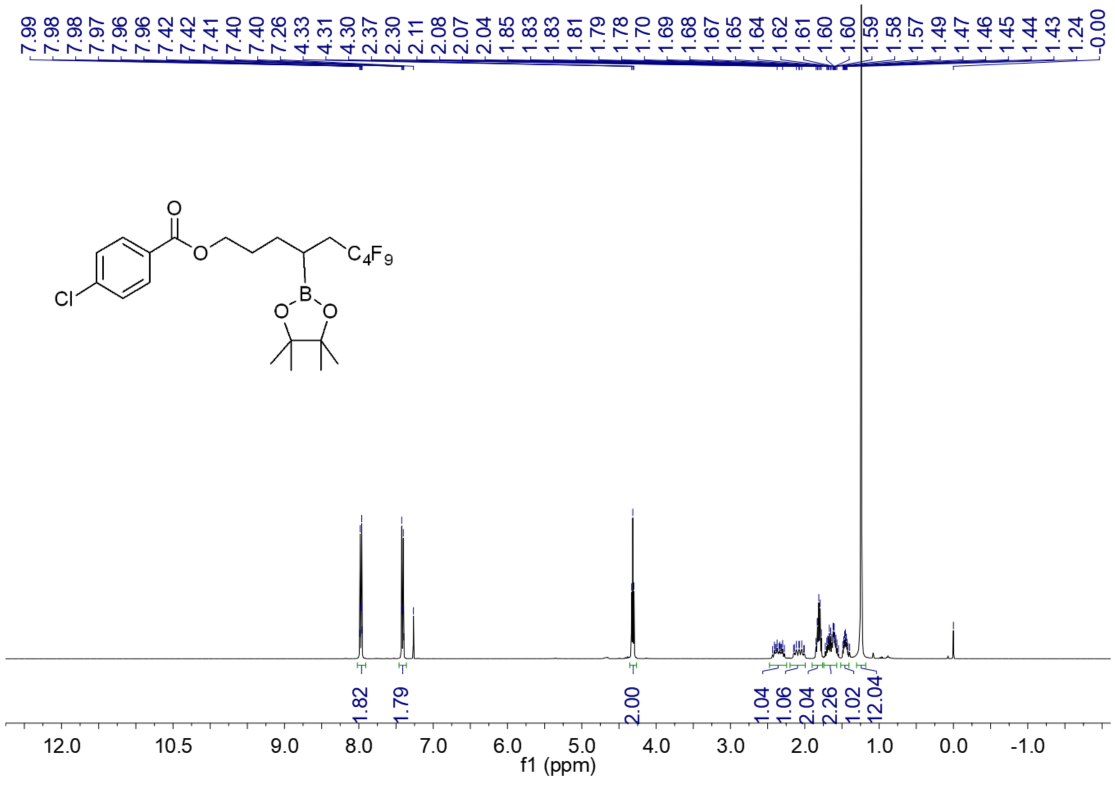

Supplementary Figure 191. ^{1}H NMR Spectra of product **5aa**

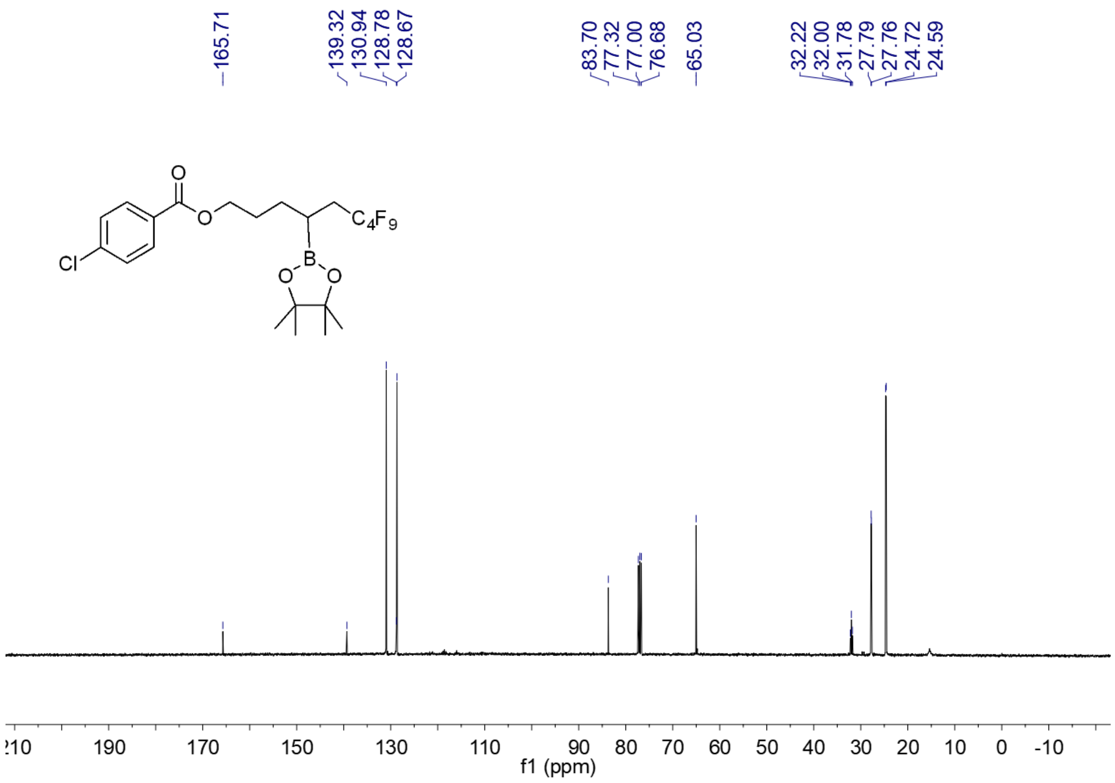

Supplementary Figure 192. ^{13}C NMR Spectra of product **5aa**

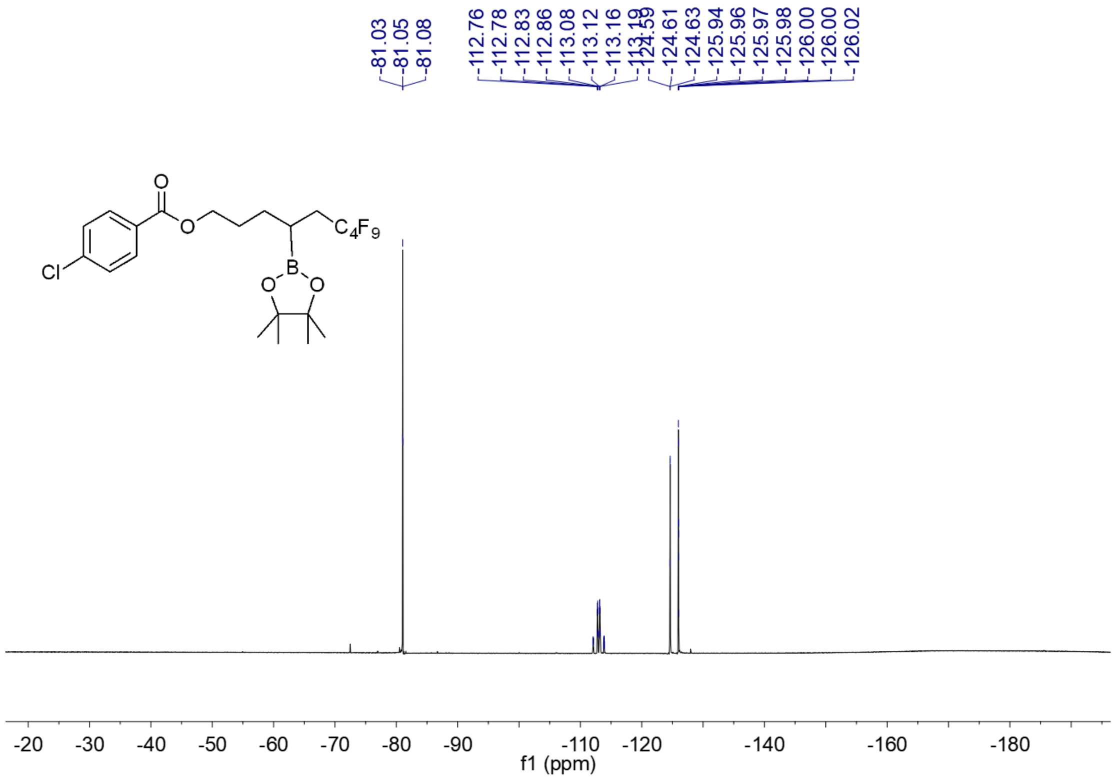

Supplementary Figure 193. ^{19}F NMR Spectra of product **5aa**

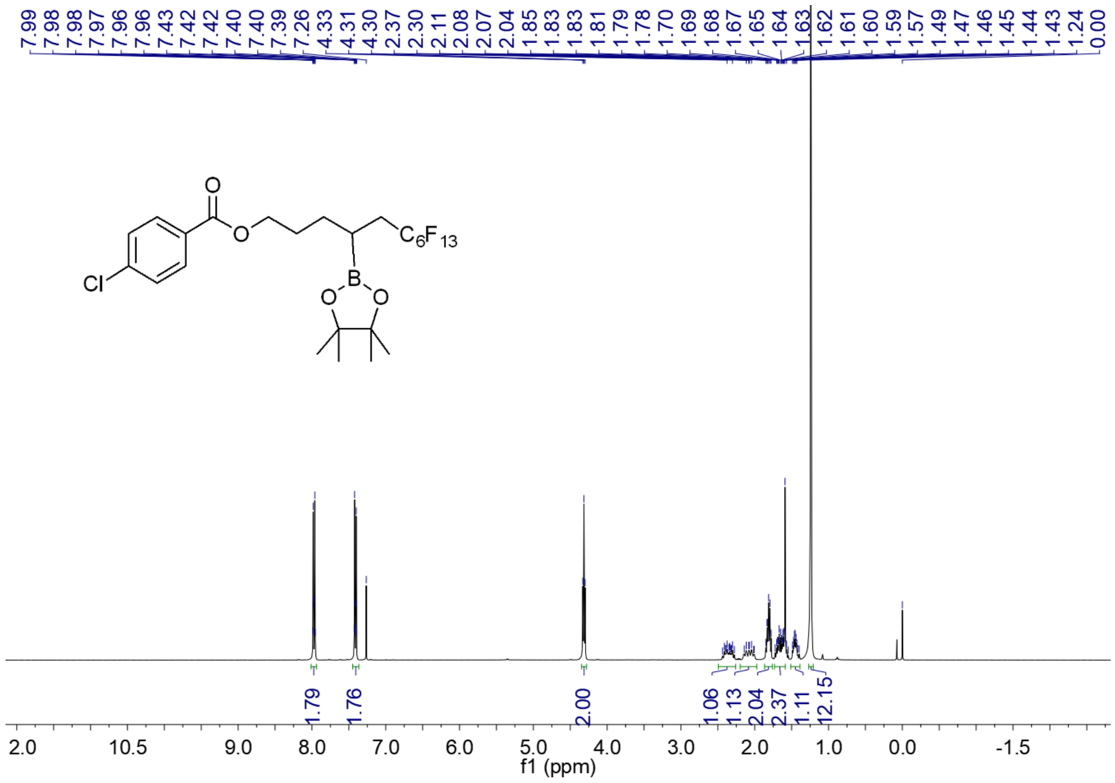

Supplementary Figure 194. ^{1}H NMR Spectra of product **5bb**

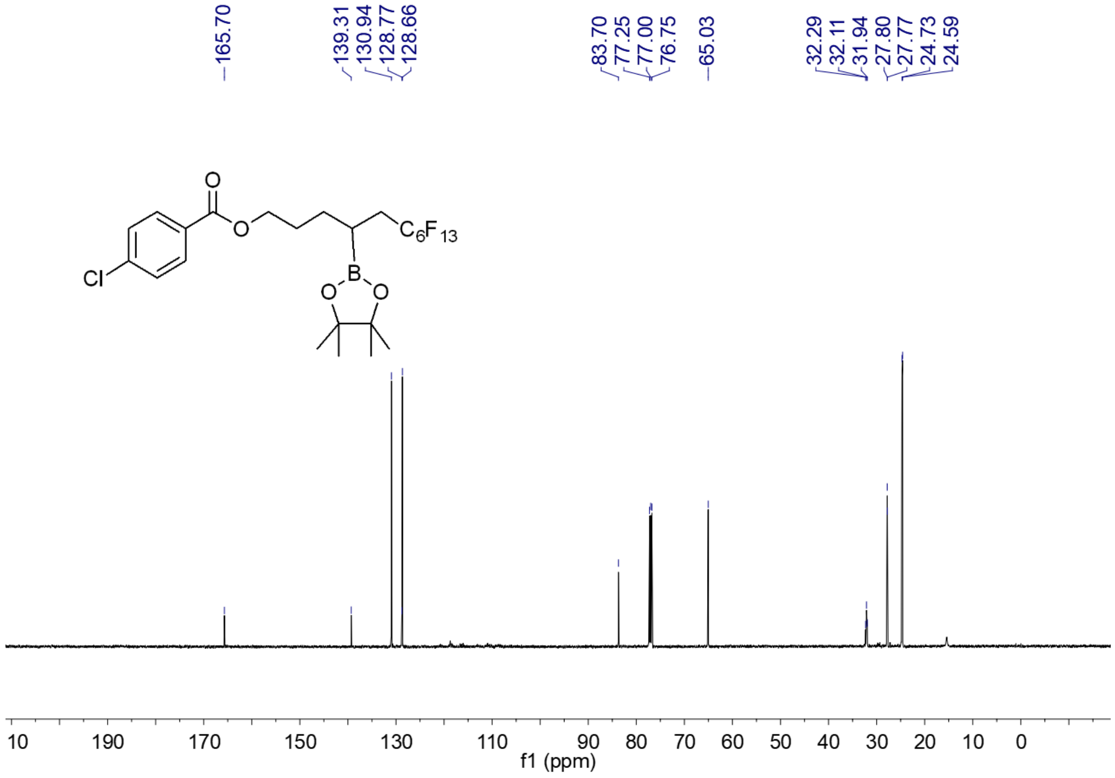

Supplementary Figure 195. ^{13}C NMR Spectra of product **5bb**

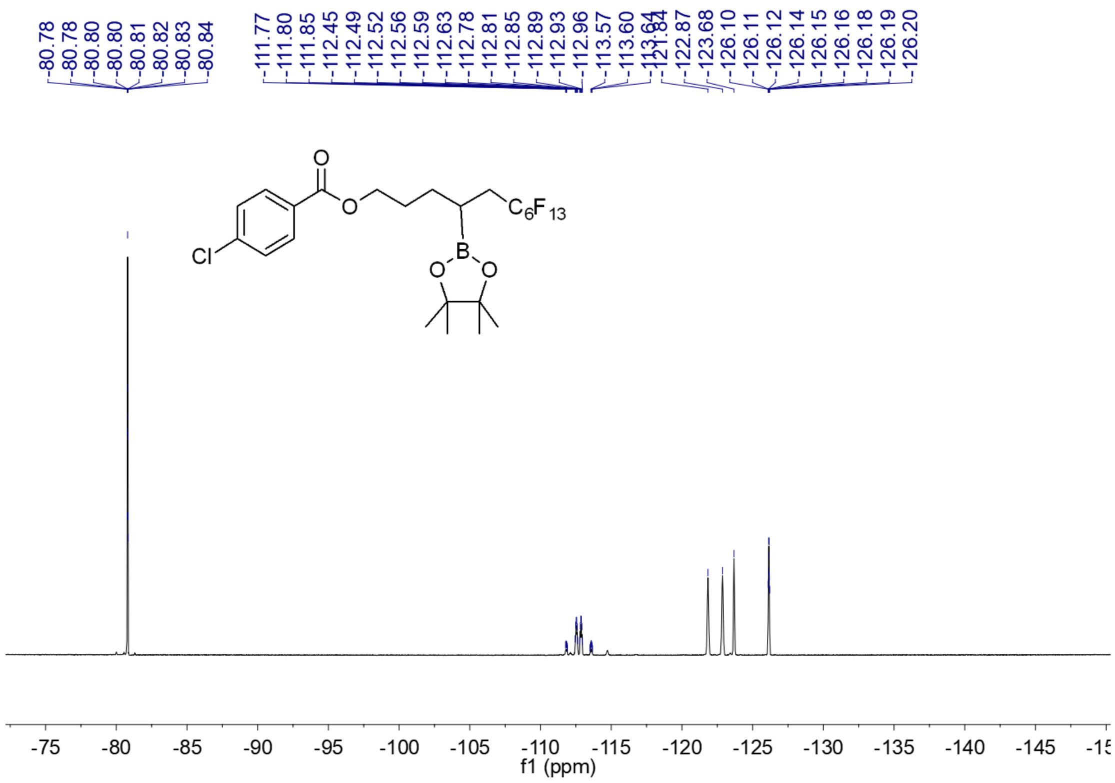

Supplementary Figure 196. ^{19}F NMR Spectra of product **5bb**

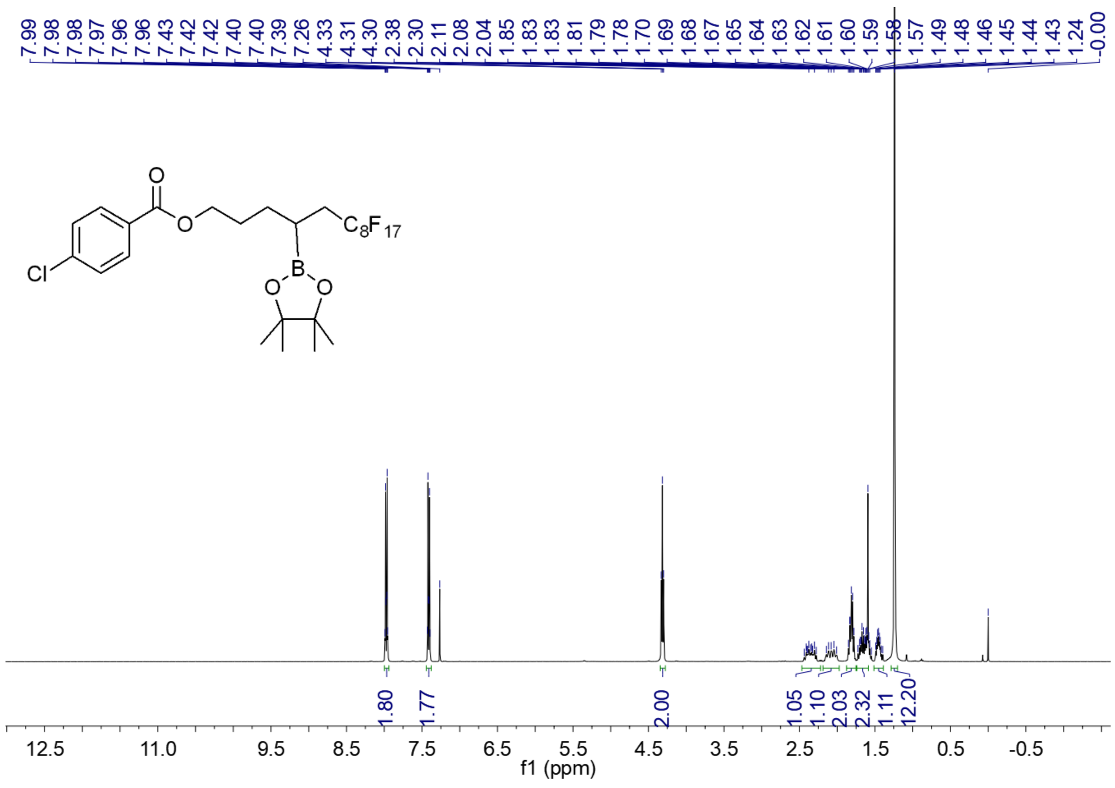

Supplementary Figure 197. ^{1}H NMR Spectra of product **5cc**

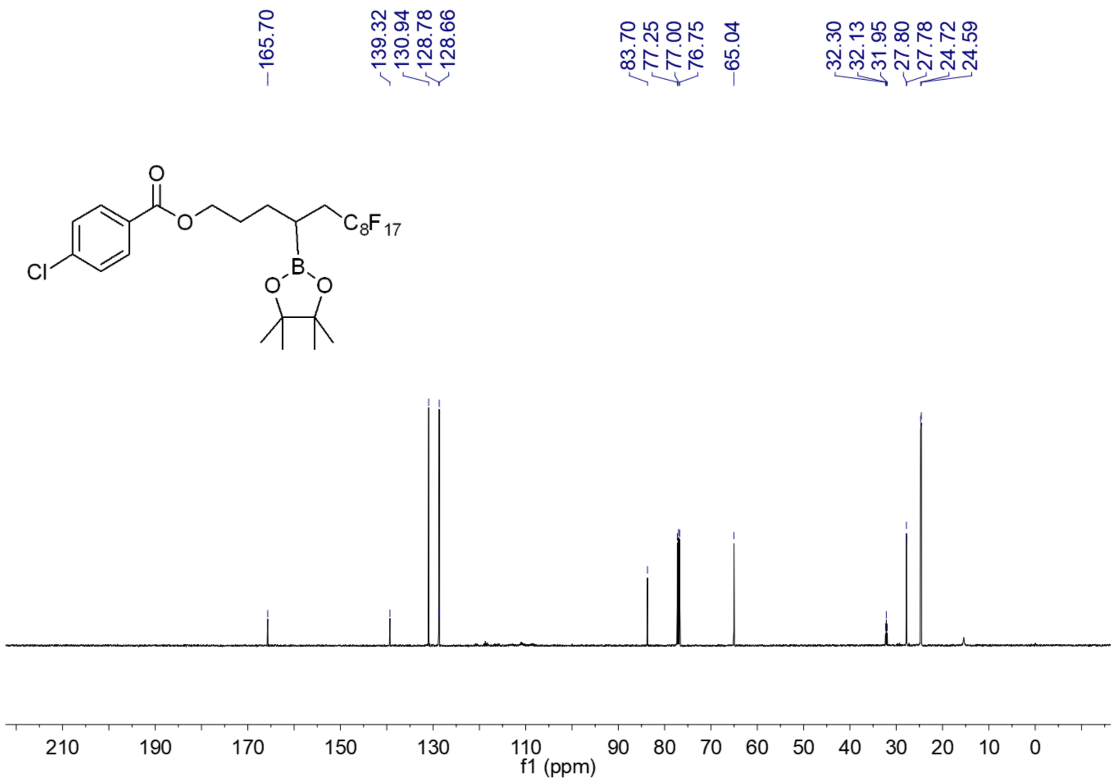

Supplementary Figure 198. ^{13}C NMR Spectra of product **5cc**

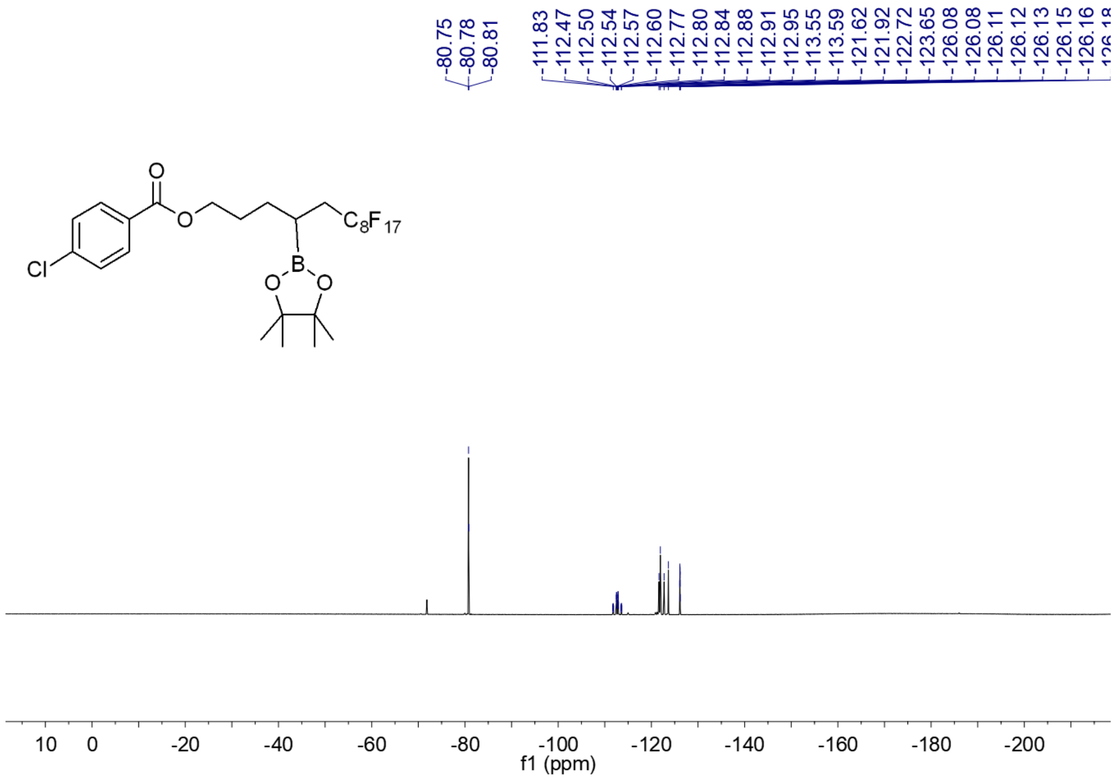

Supplementary Figure 199. ^{19}F NMR Spectra of product **5cc**

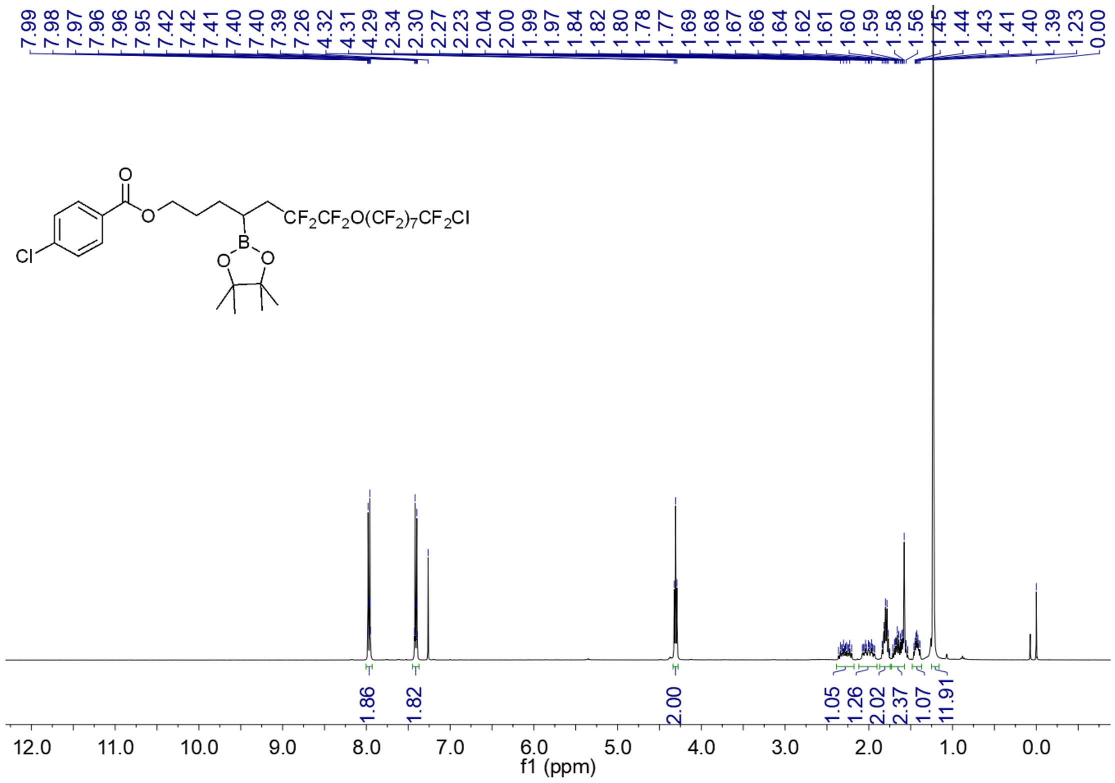

Supplementary Figure 200. ^{1}H NMR Spectra of product **5dd**

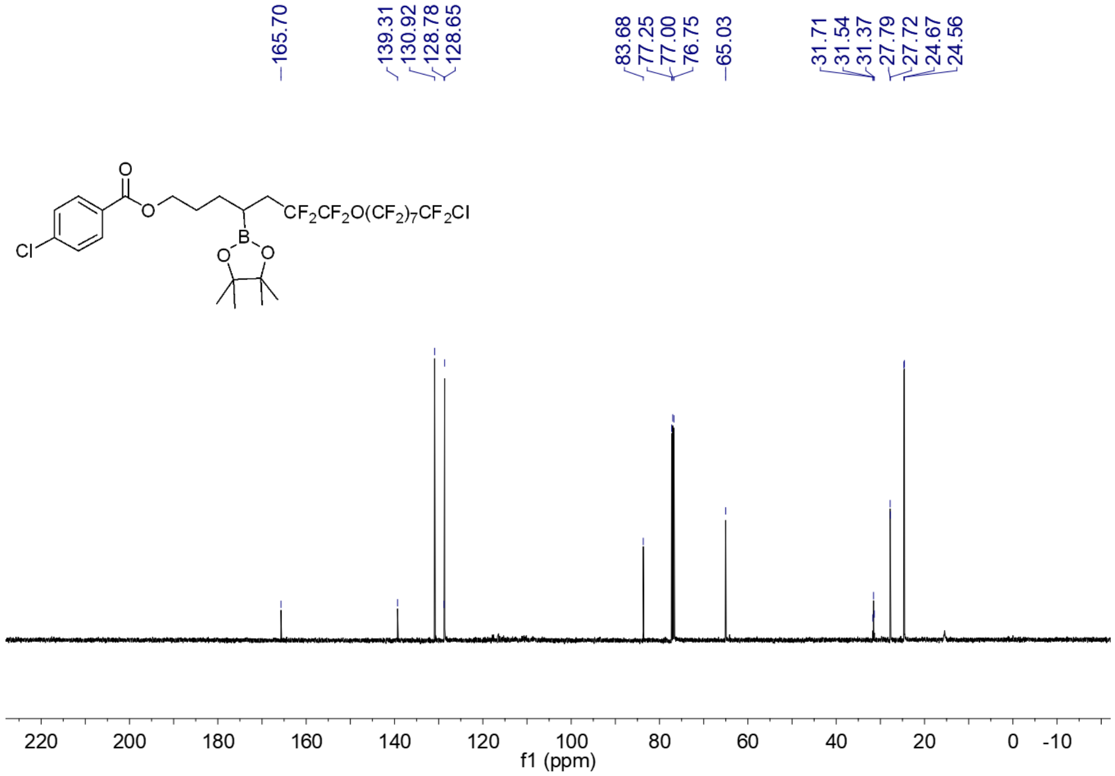

Supplementary Figure 201. ^{13}C NMR Spectra of product **5dd**

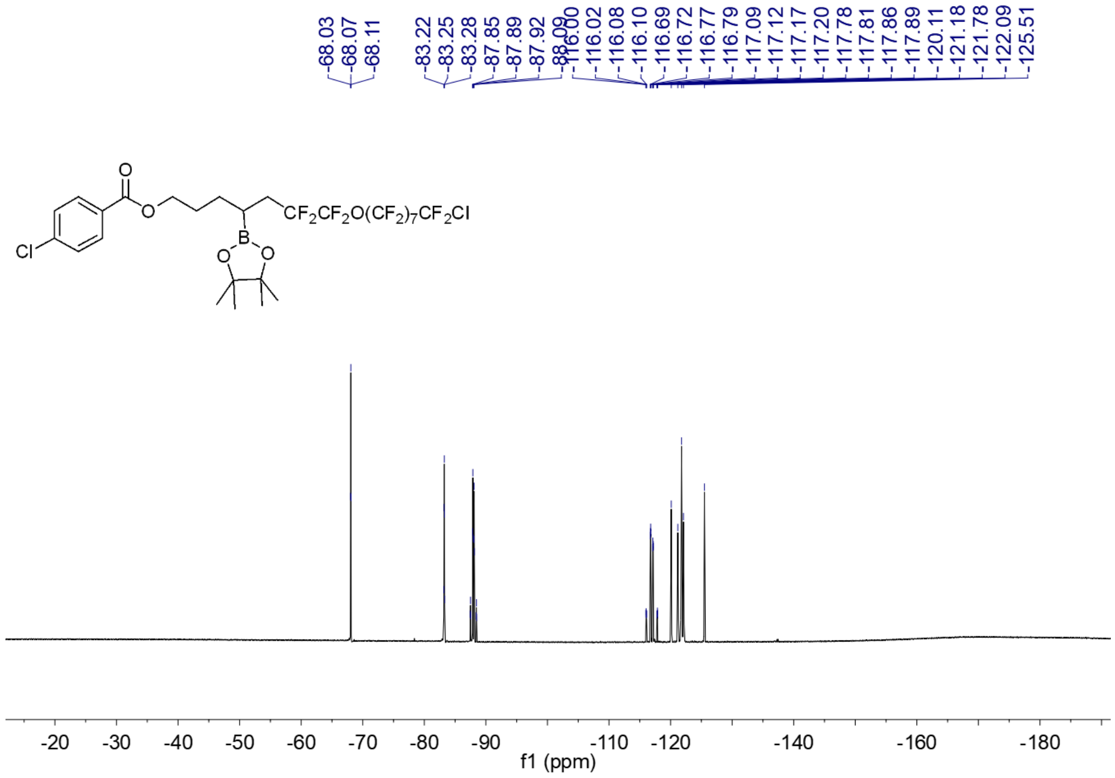

Supplementary Figure 202. ^{19}F NMR Spectra of product **5dd**

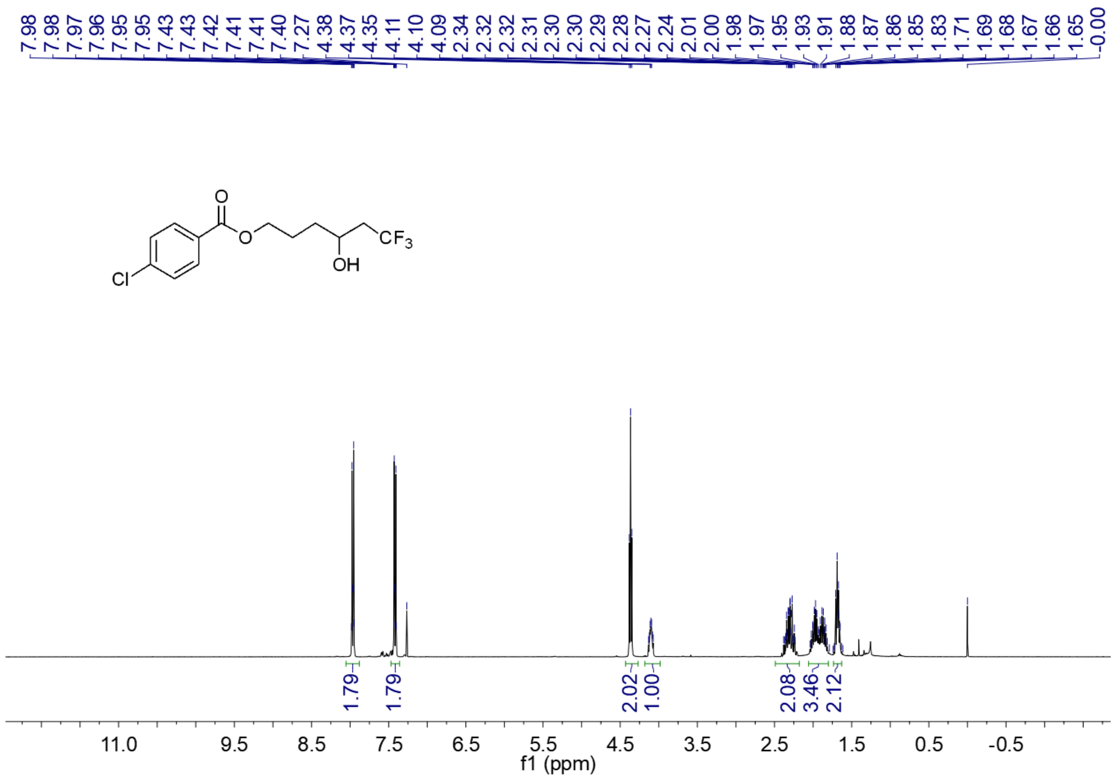

Supplementary Figure 203. ^{1}H NMR Spectra of product **6**

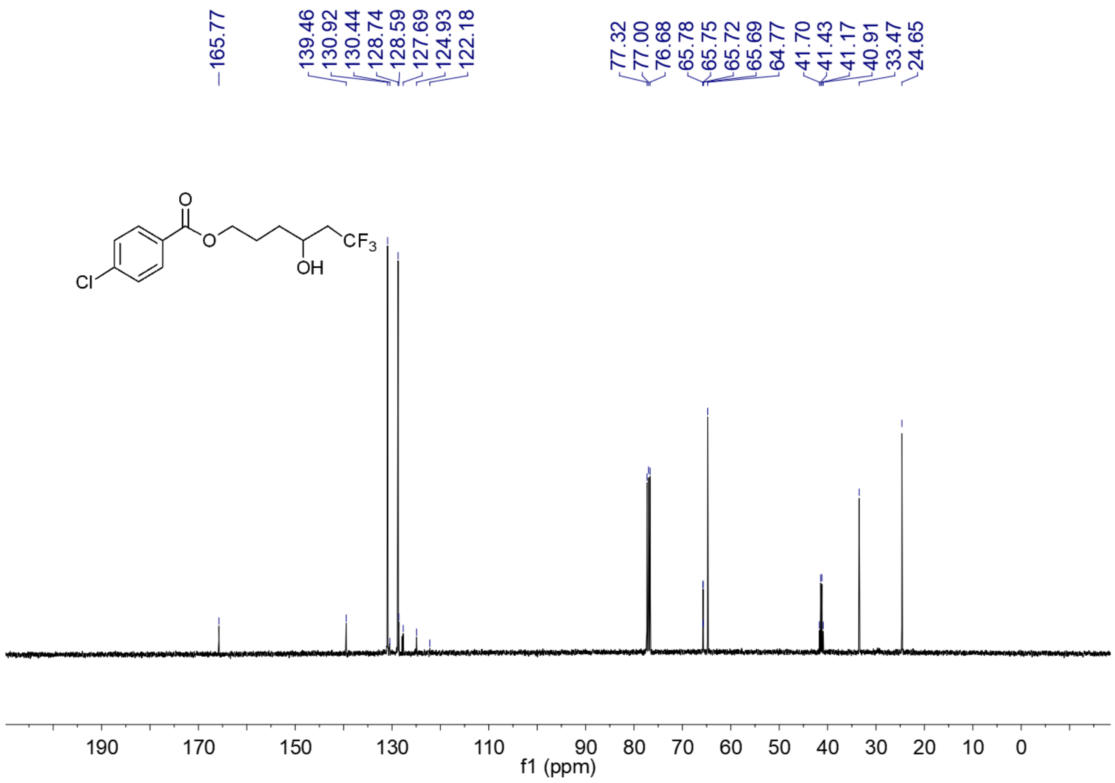

Supplementary Figure 204. ^{13}C NMR Spectra of product **6**

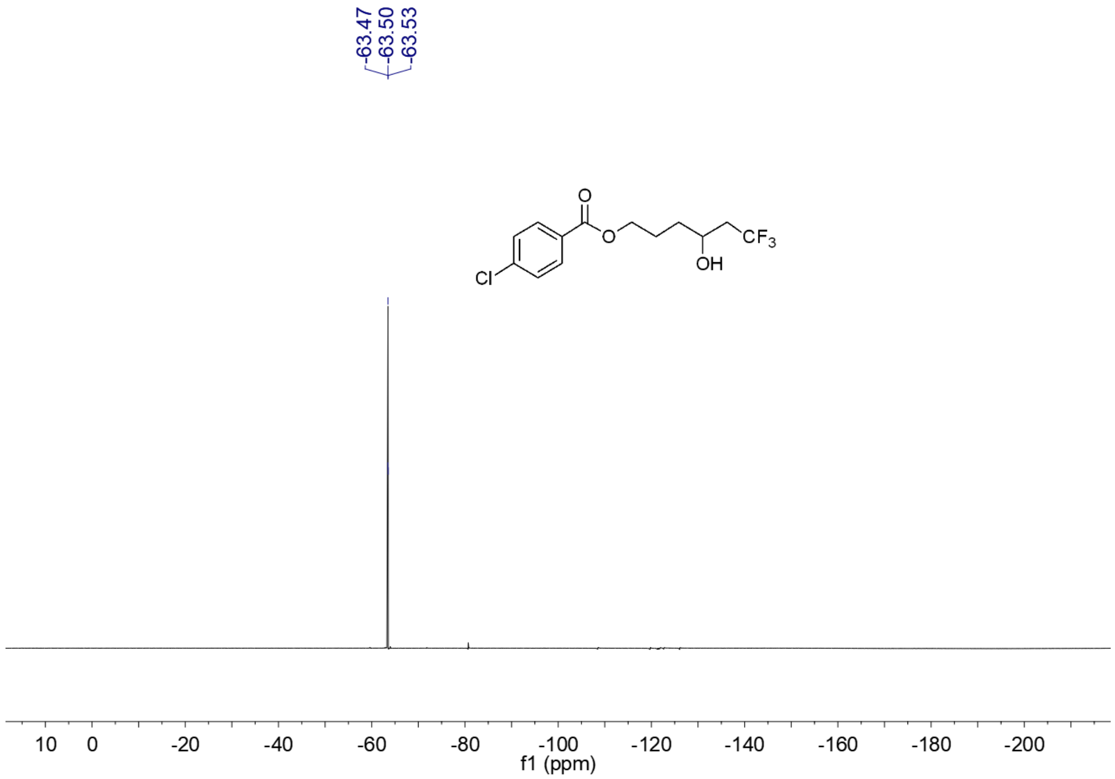

Supplementary Figure 205. ^{19}F NMR Spectra of product **6**

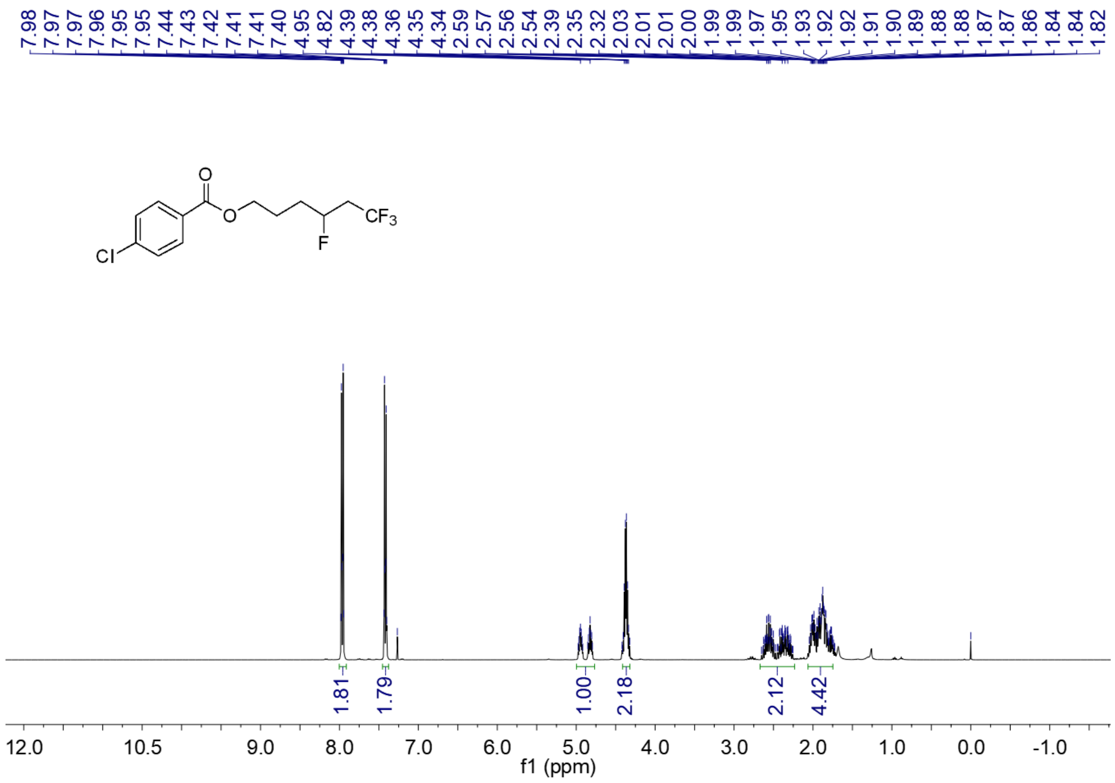

Supplementary Figure 206. ^{1}H NMR Spectra of product **7**

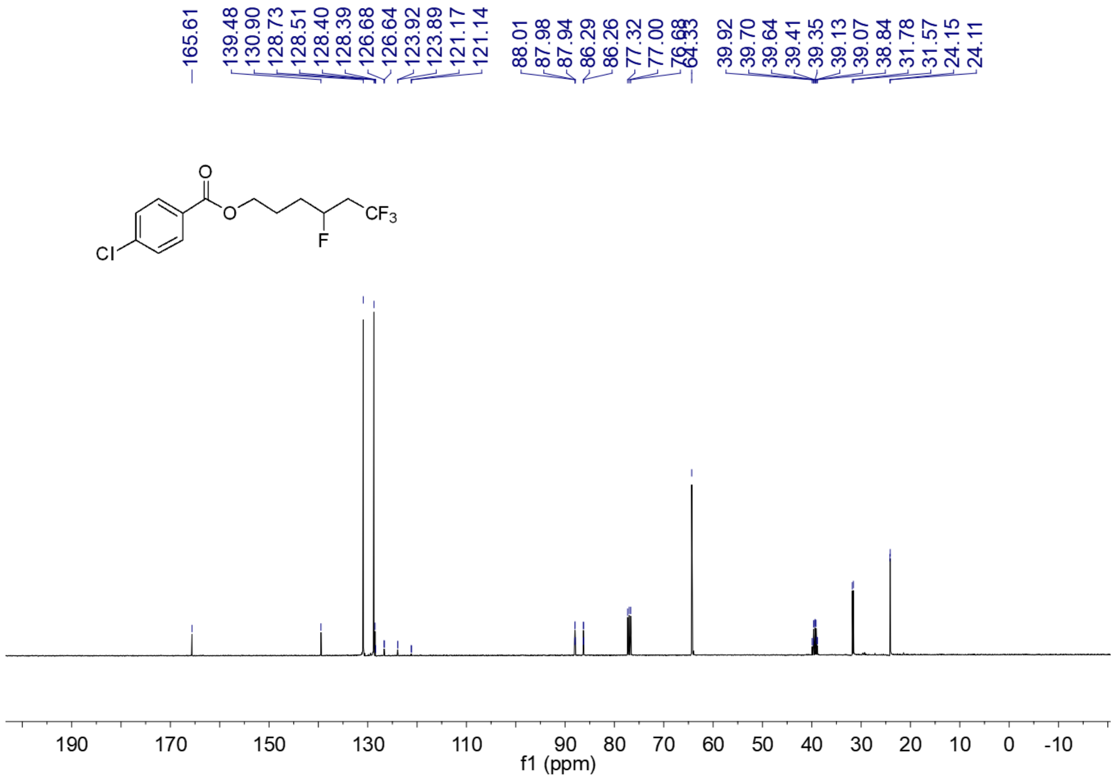

Supplementary Figure 207. ^{13}C NMR Spectra of product **7**

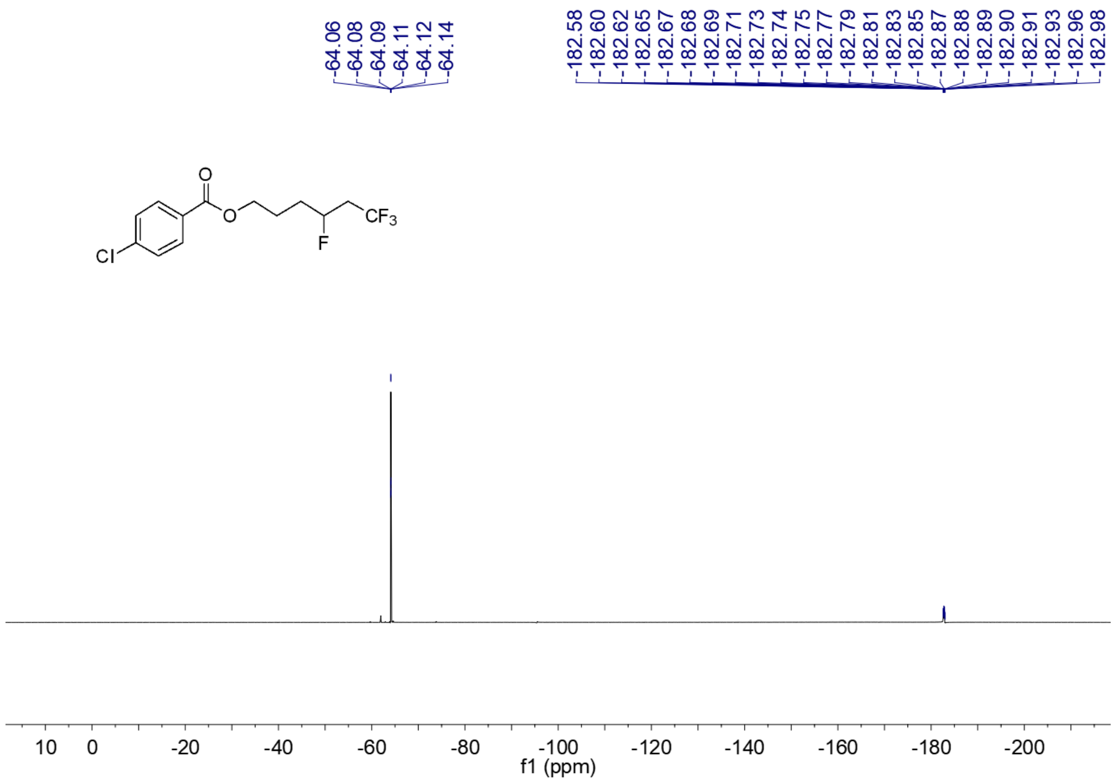

Supplementary Figure 208. ^{19}F NMR Spectra of product **7**

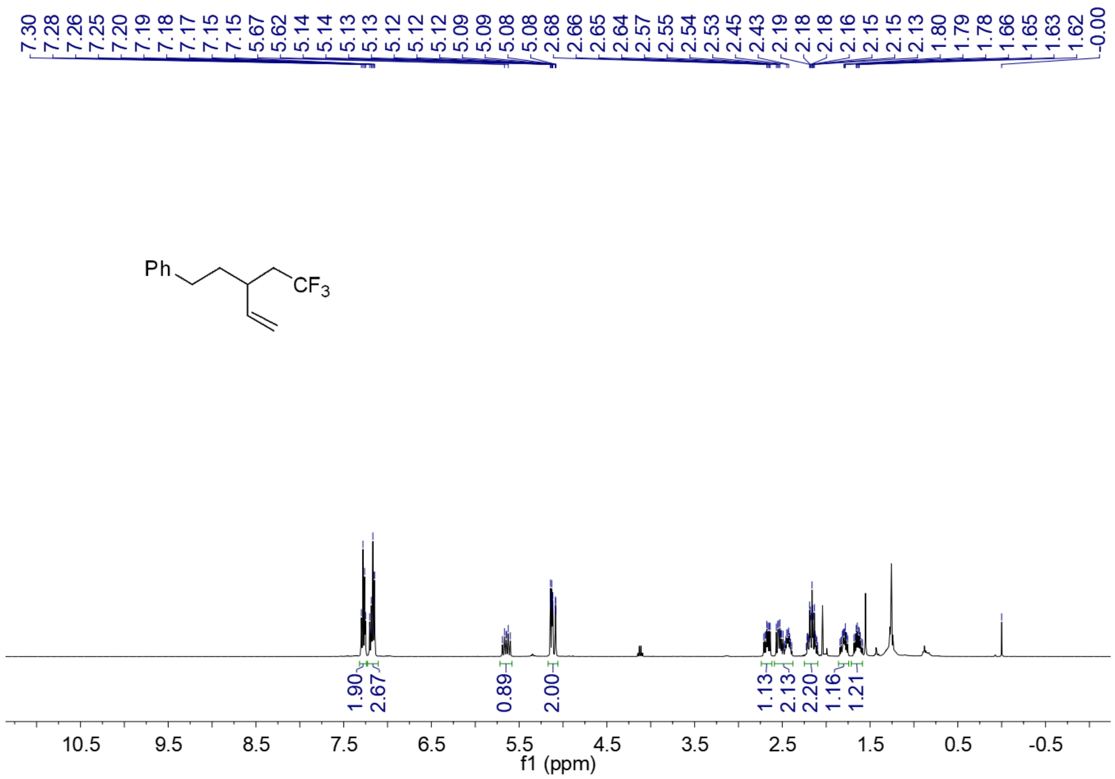

Supplementary Figure 209. ^{1}H NMR Spectra of product **8**

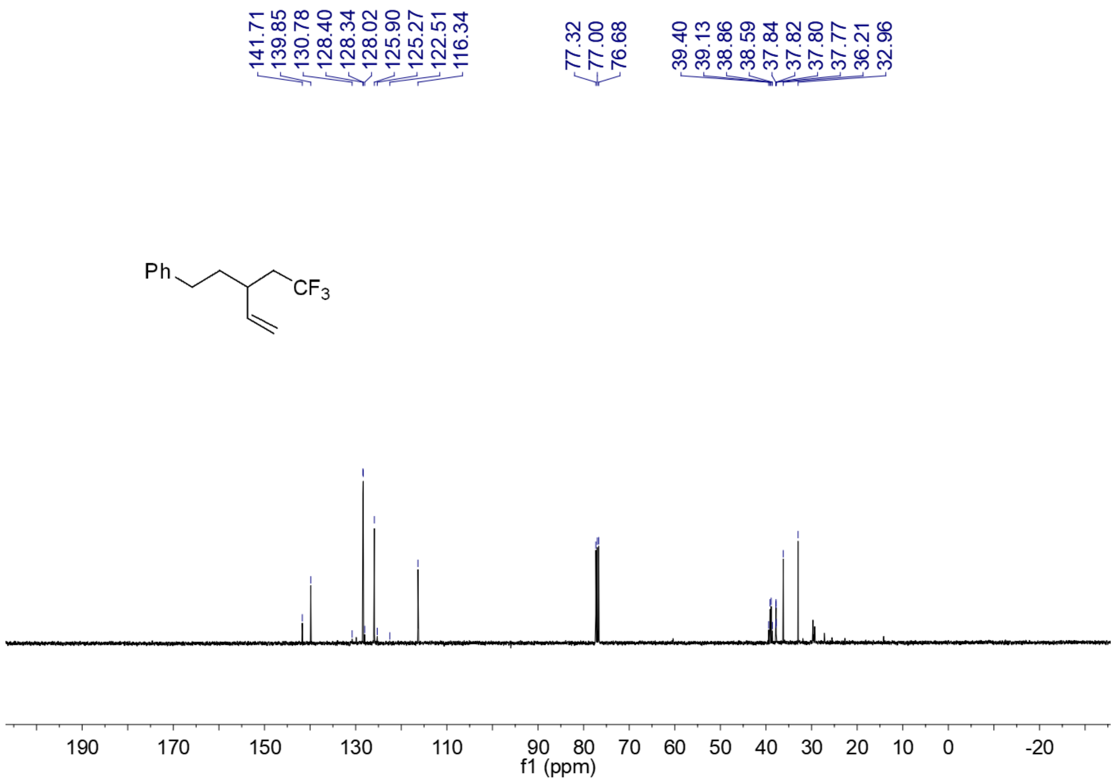

Supplementary Figure 210. ^{13}C NMR Spectra of product **8**

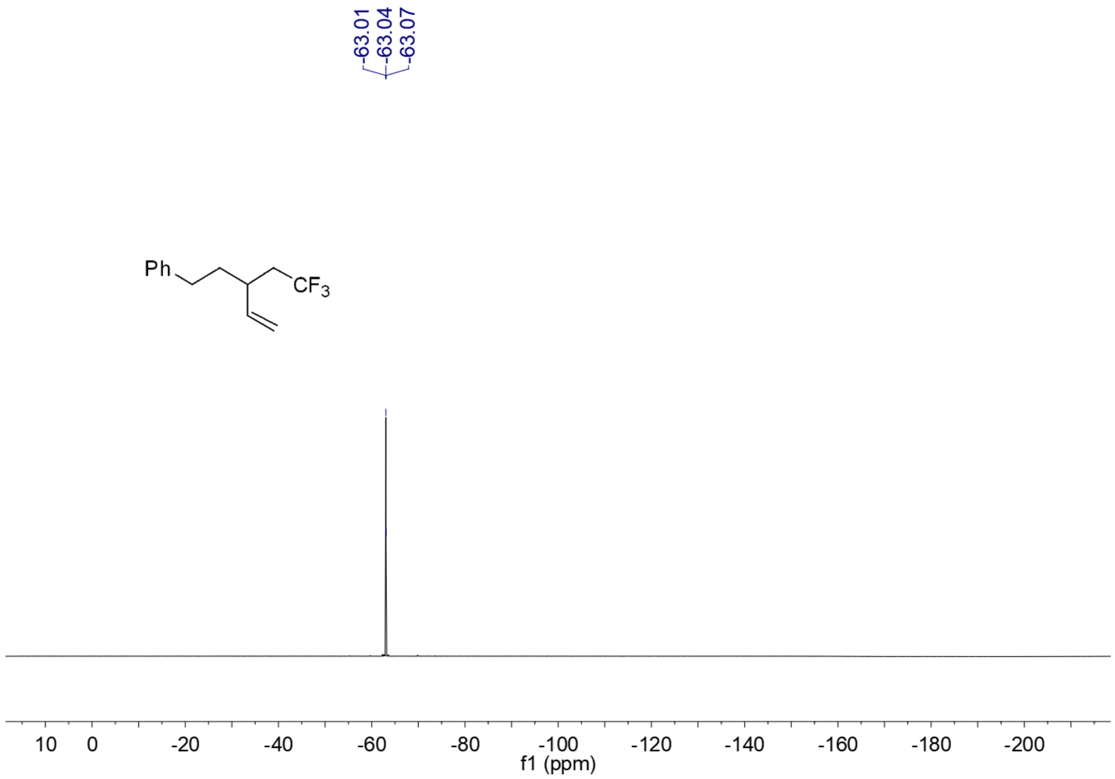

Supplementary Figure 211. ^{19}F NMR Spectra of product **8**

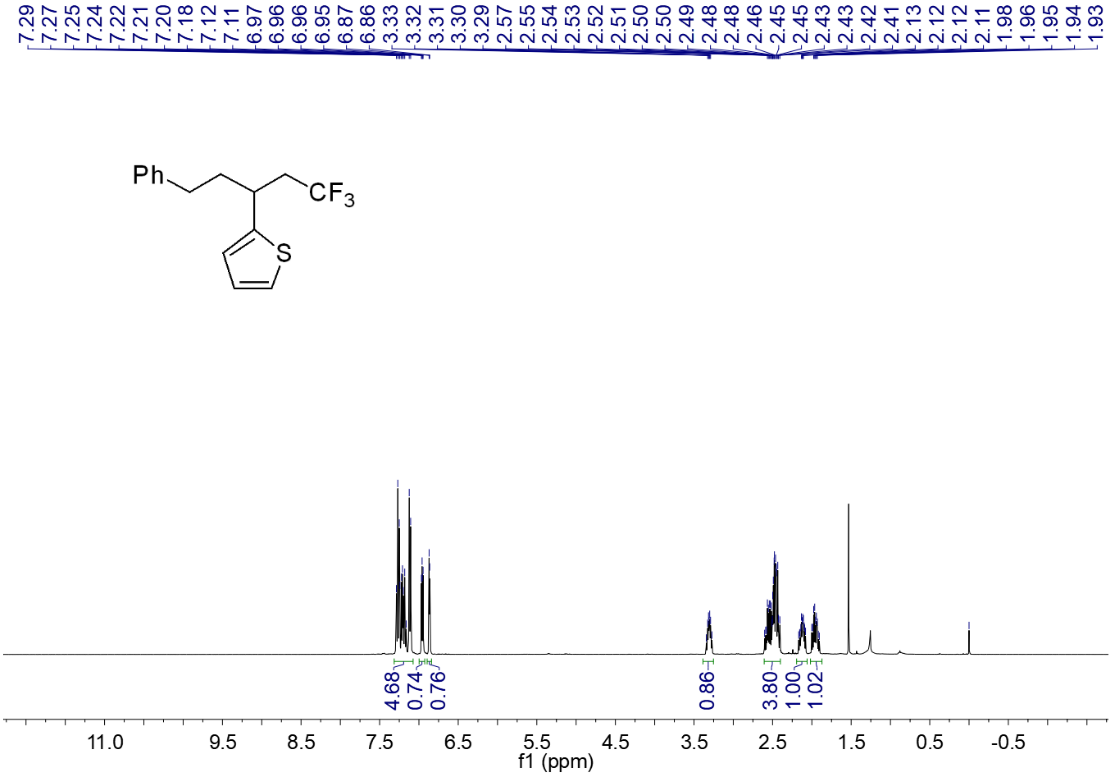

Supplementary Figure 212. ^{1}H NMR Spectra of product **9**

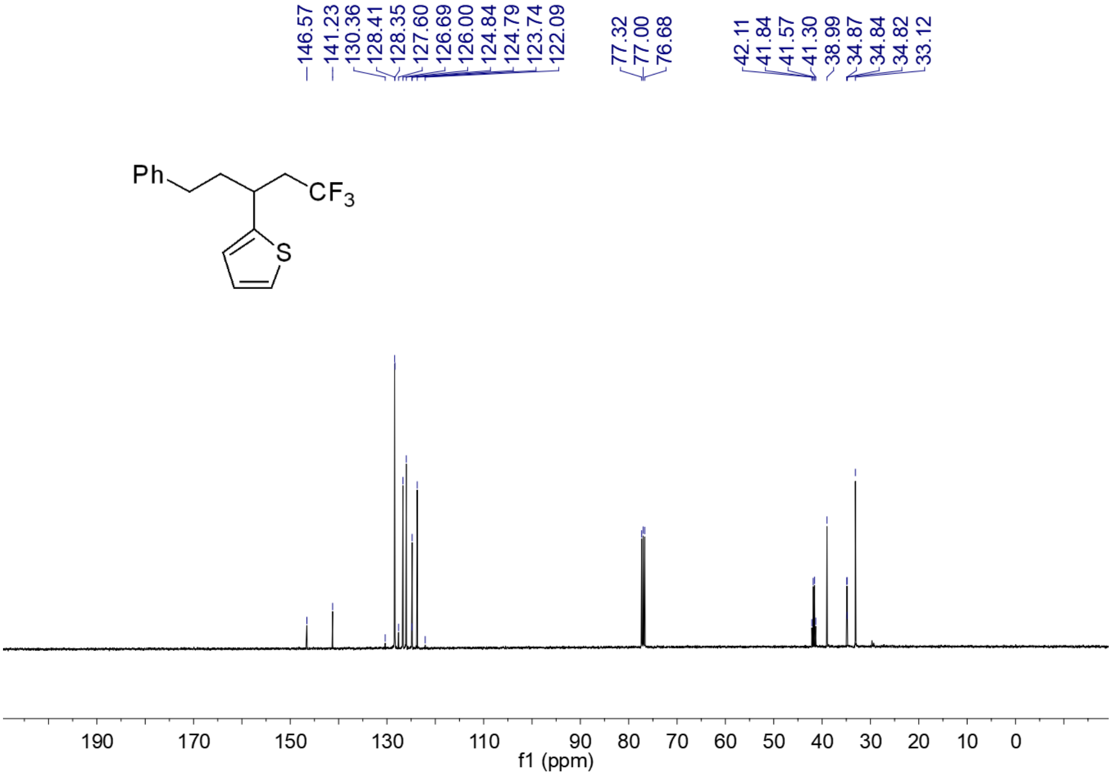

Supplementary Figure 213. ^{13}C NMR Spectra of product **9**

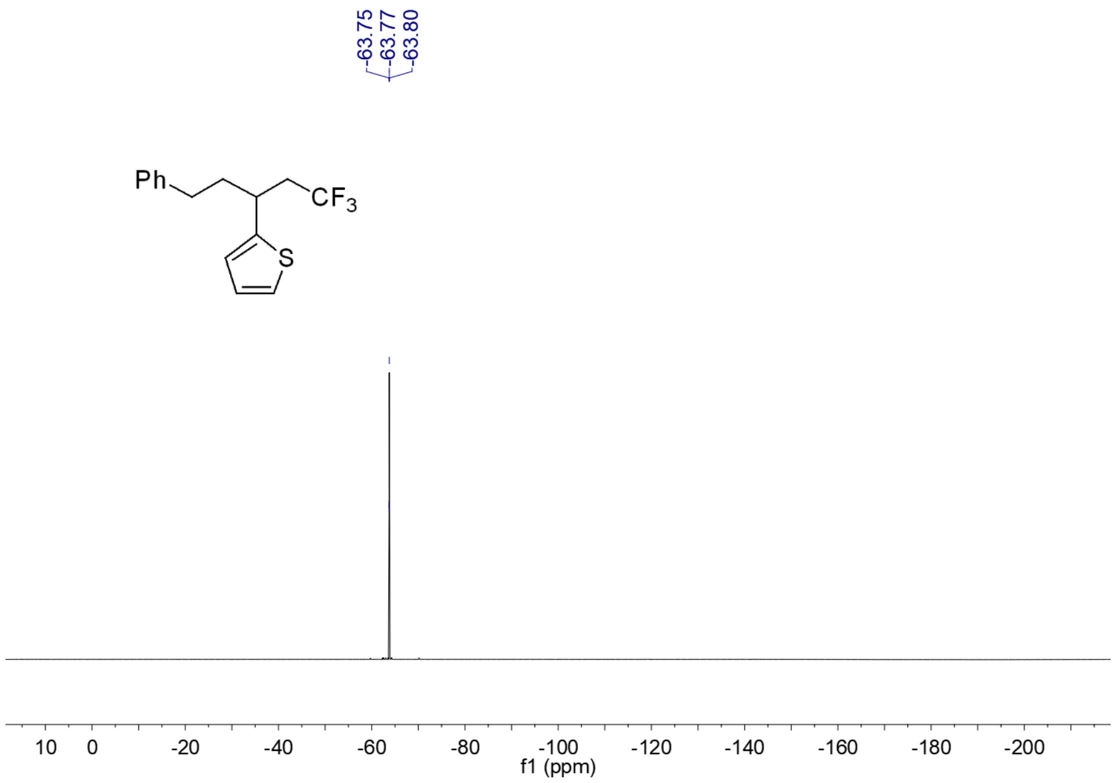

Supplementary Figure 214. ^{19}F NMR Spectra of product **9**

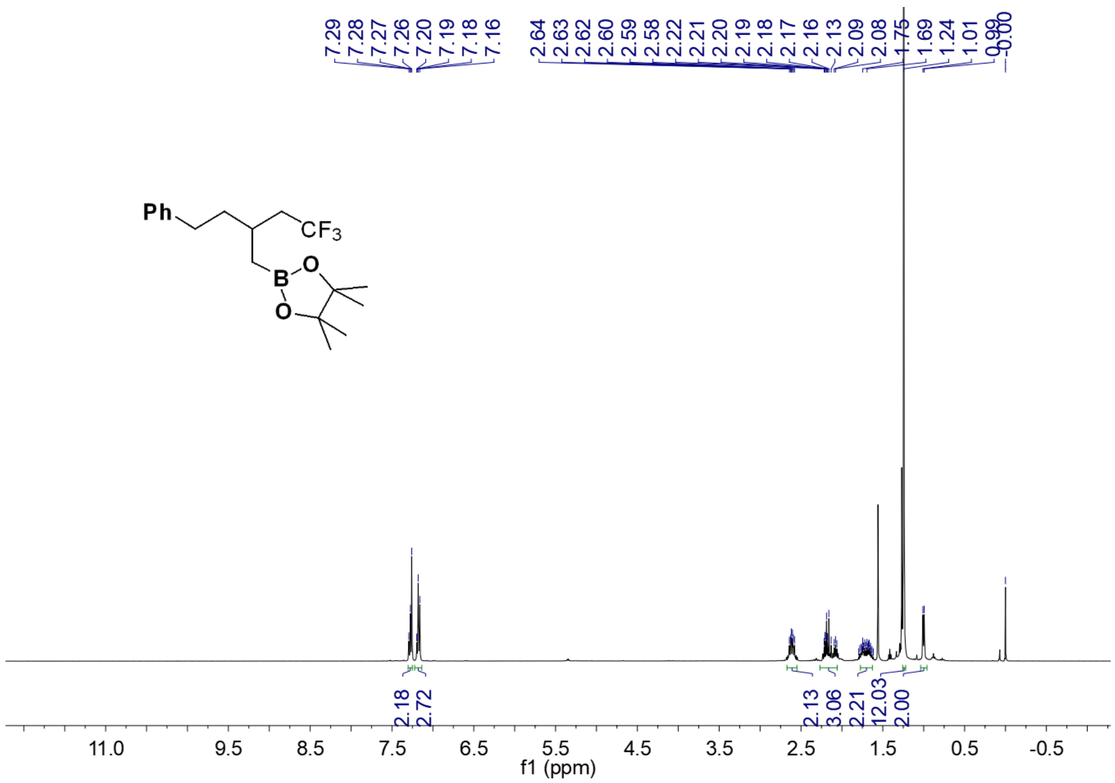

Supplementary Figure 215. ^{1}H NMR Spectra of product **10**

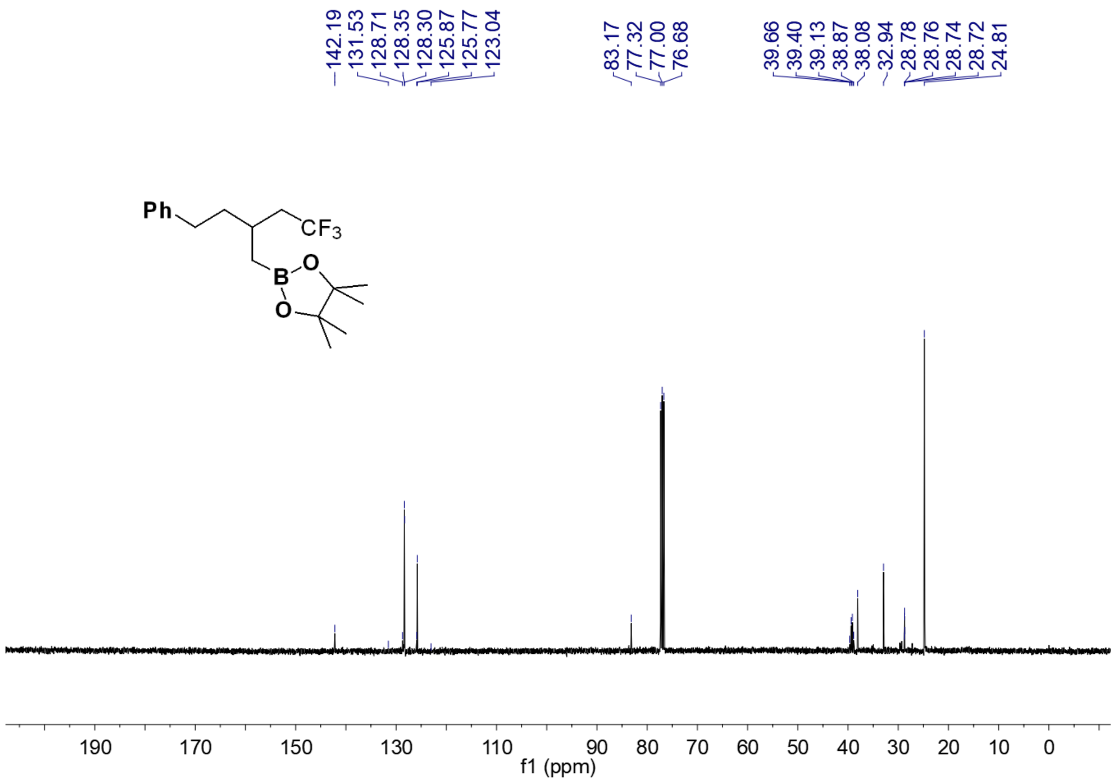

Supplementary Figure 216. ^{13}C NMR Spectra of product **10**

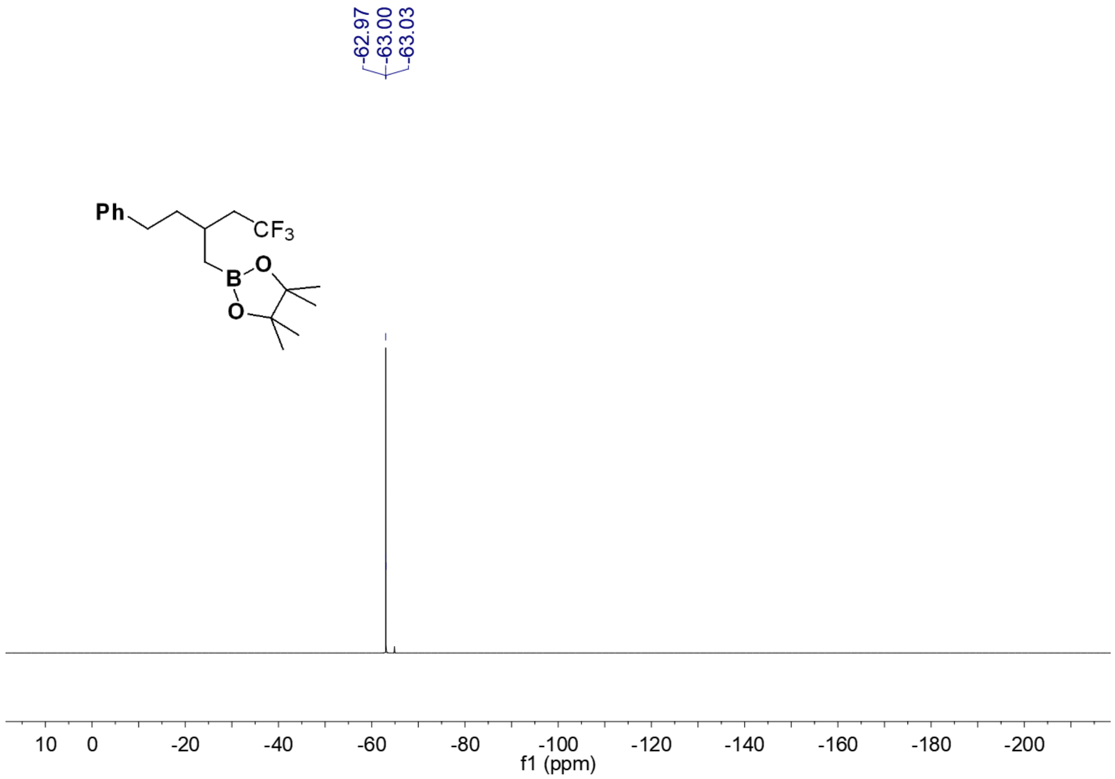

Supplementary Figure 217. ^{19}F NMR Spectra of product **10**

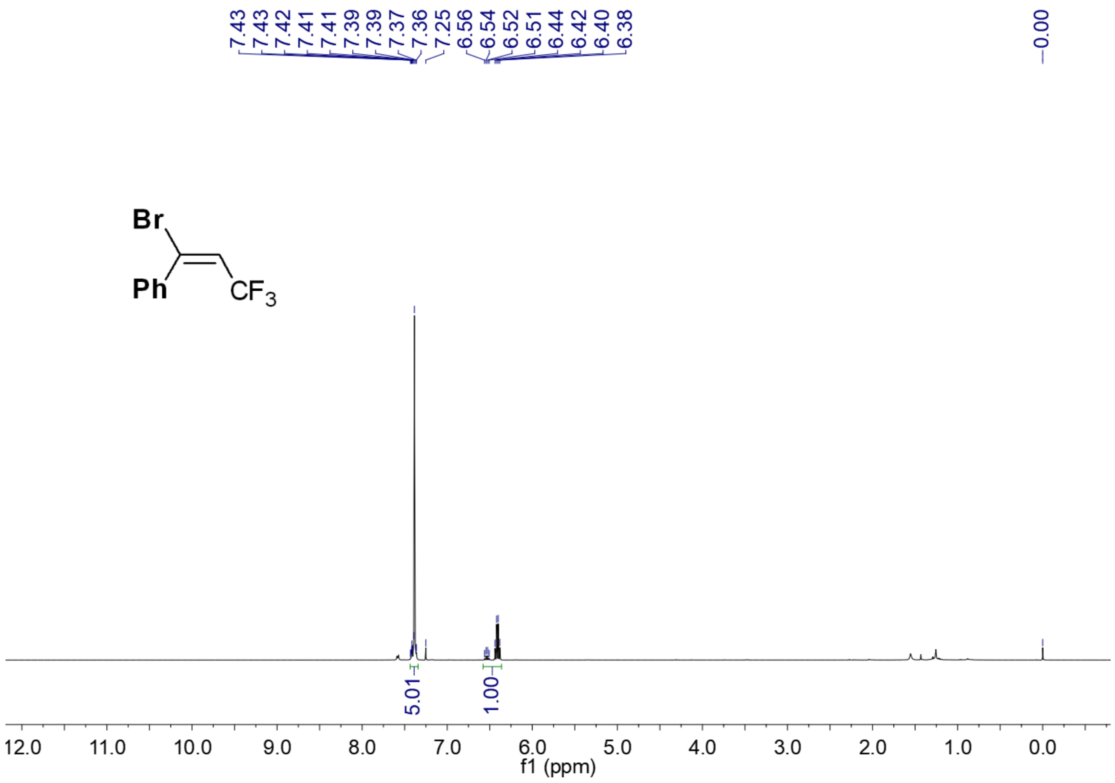

Supplementary Figure 218. ^{1}H NMR Spectra of product **11**

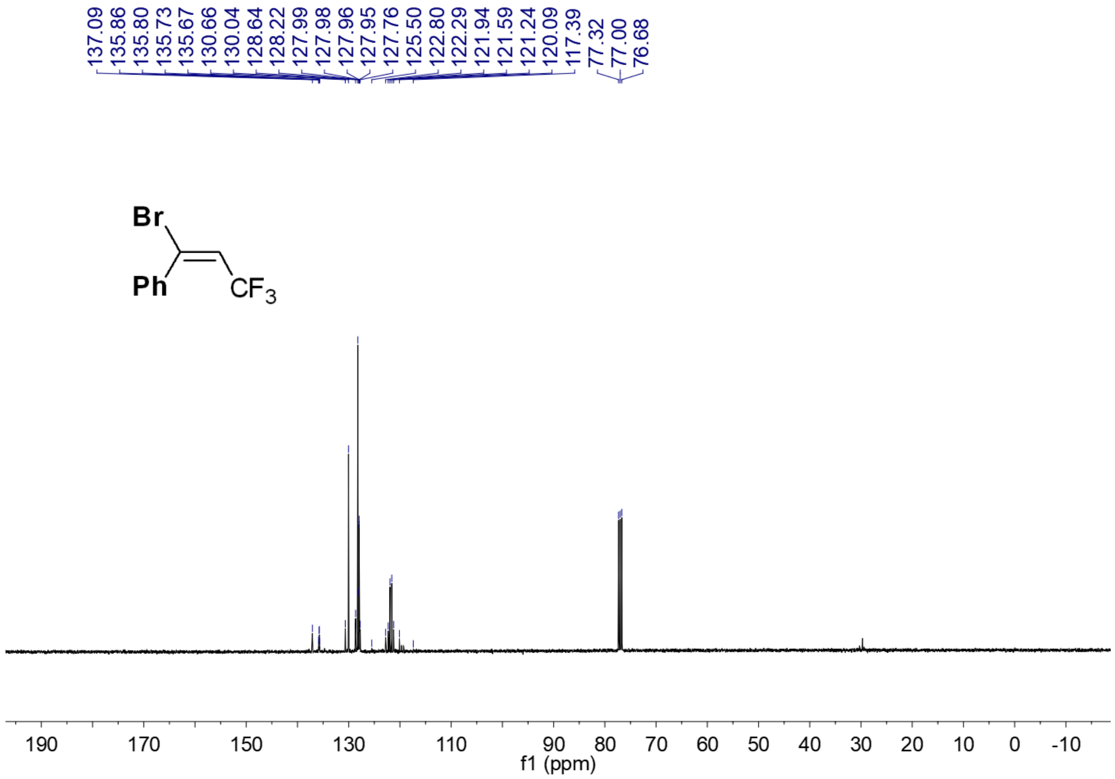

Supplementary Figure 219. ^{13}C NMR Spectra of product **11**

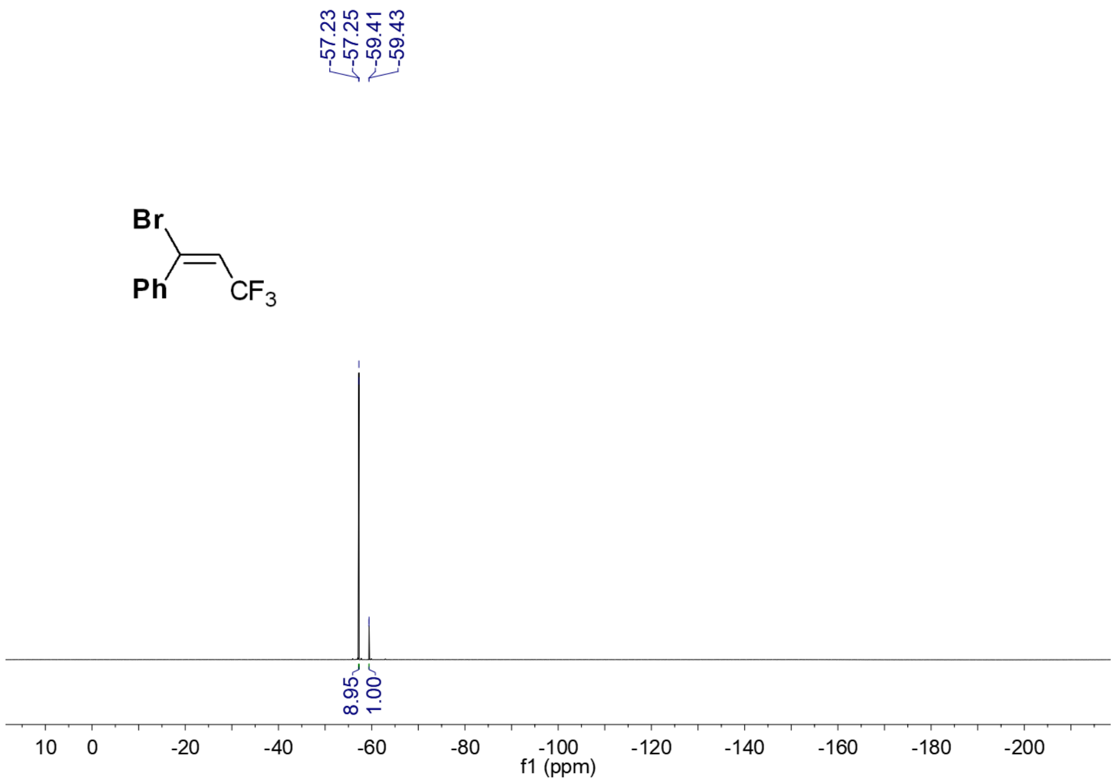

Supplementary Figure 220. ^{19}F NMR Spectra of product **11**

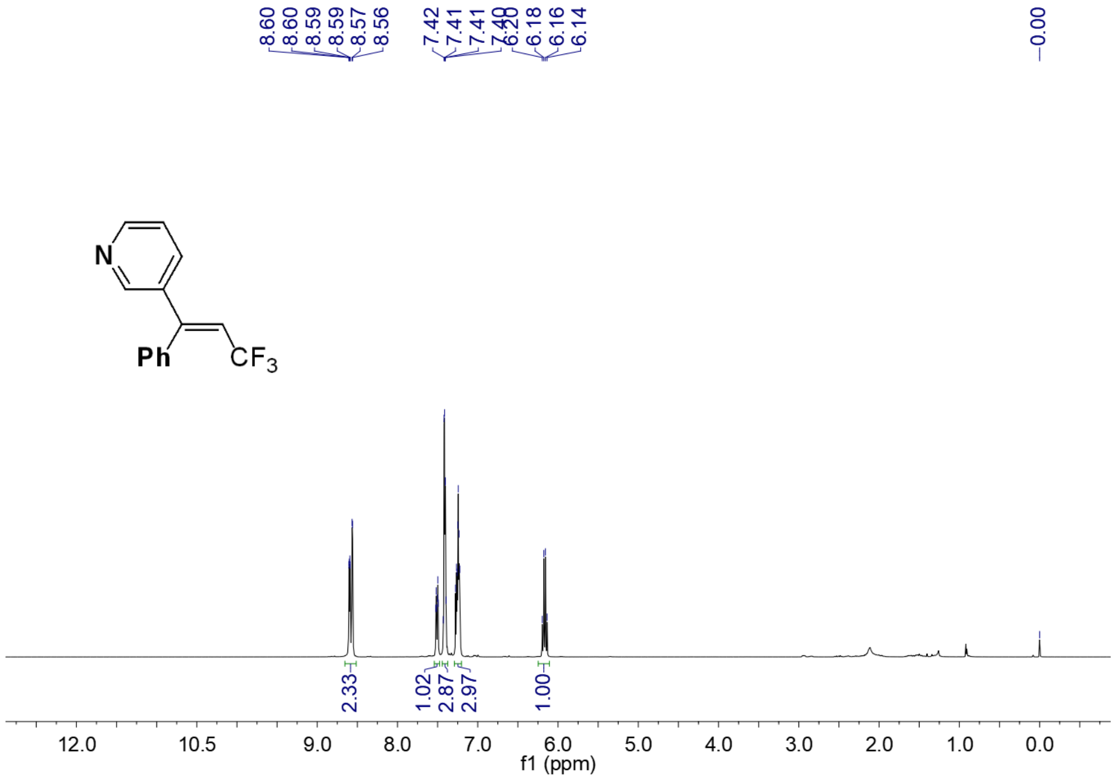

Supplementary Figure 221. ^{1}H NMR Spectra of product **12**

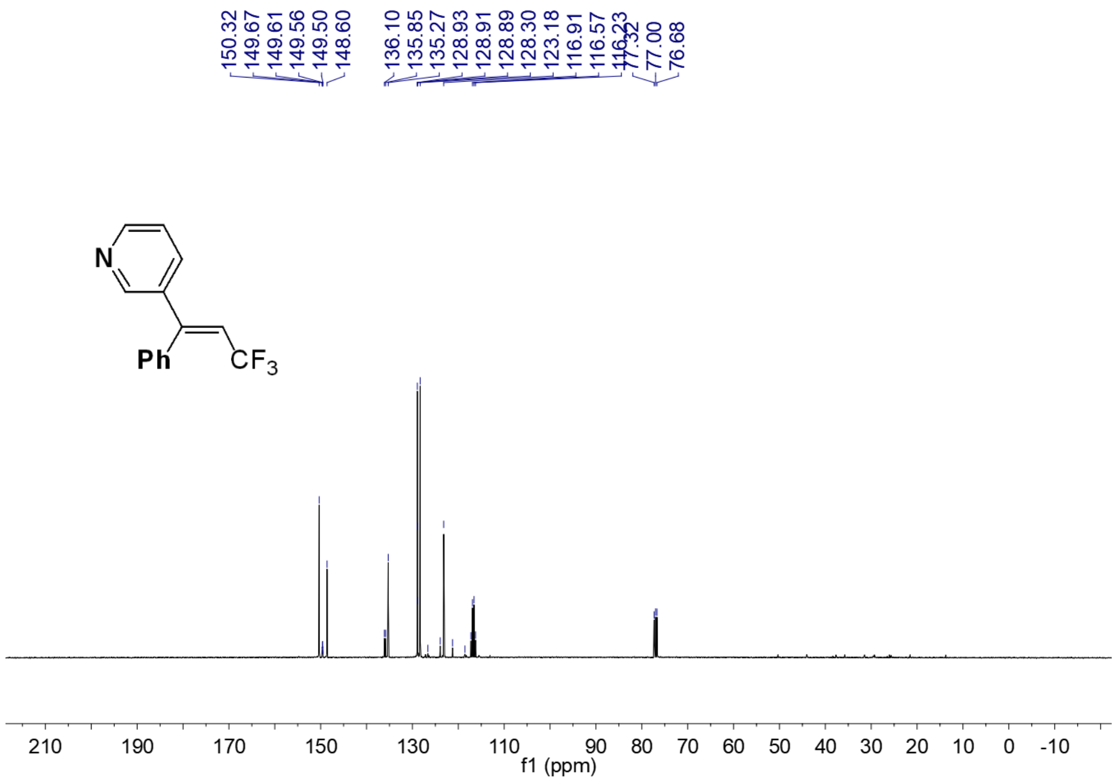

Supplementary Figure 222. ^{13}C NMR Spectra of product **12**

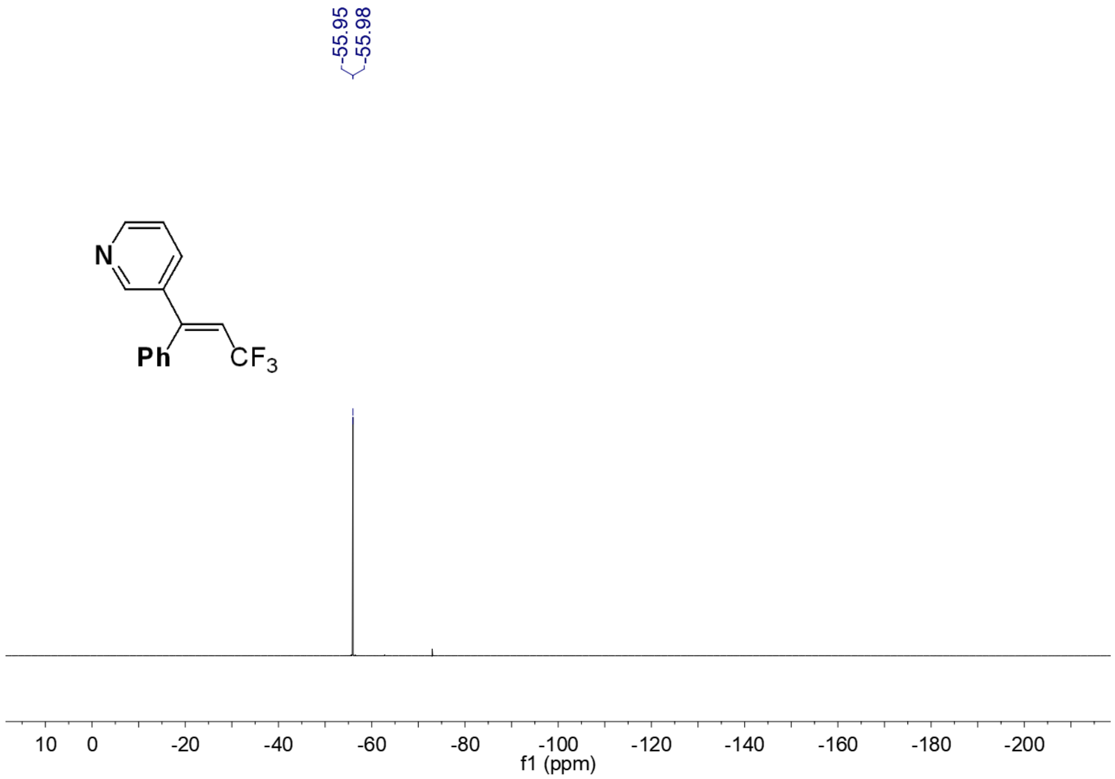

Supplementary Figure 223. ^{19}F NMR Spectra of product **12**

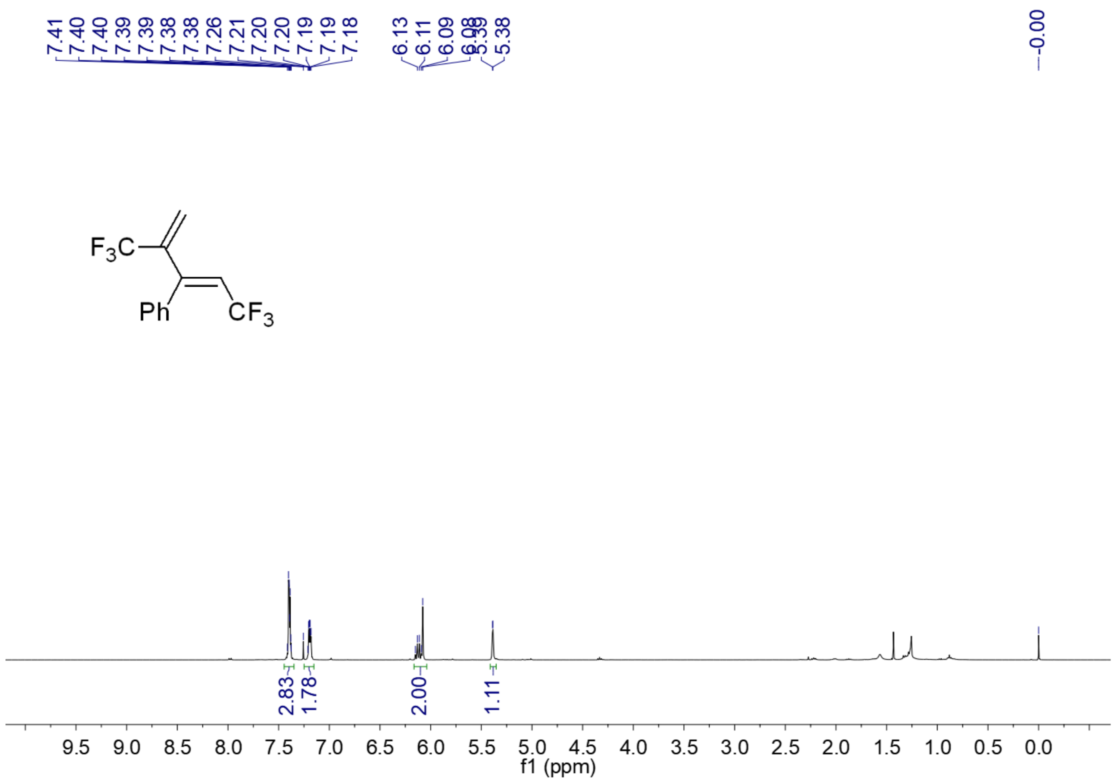

Supplementary Figure 224. ^{1}H NMR Spectra of product **13**

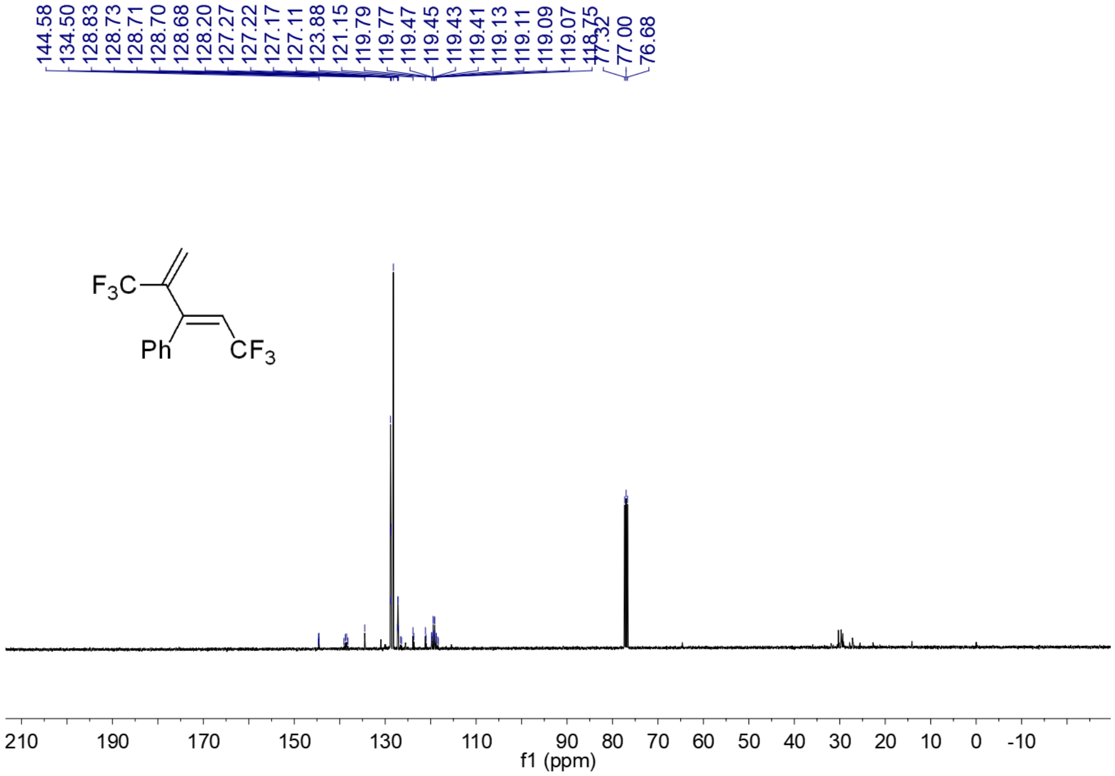

Supplementary Figure 225. ^{13}C NMR Spectra of product **13**

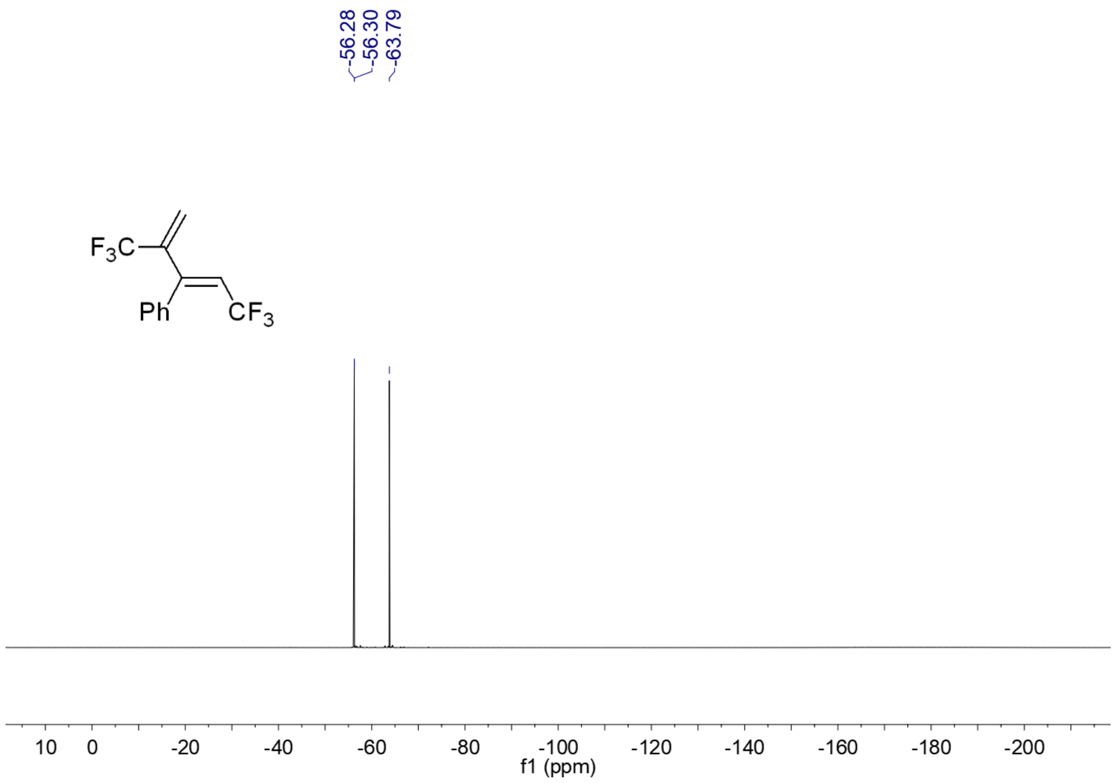

Supplementary Figure 226. ^{19}F NMR Spectra of product **13**

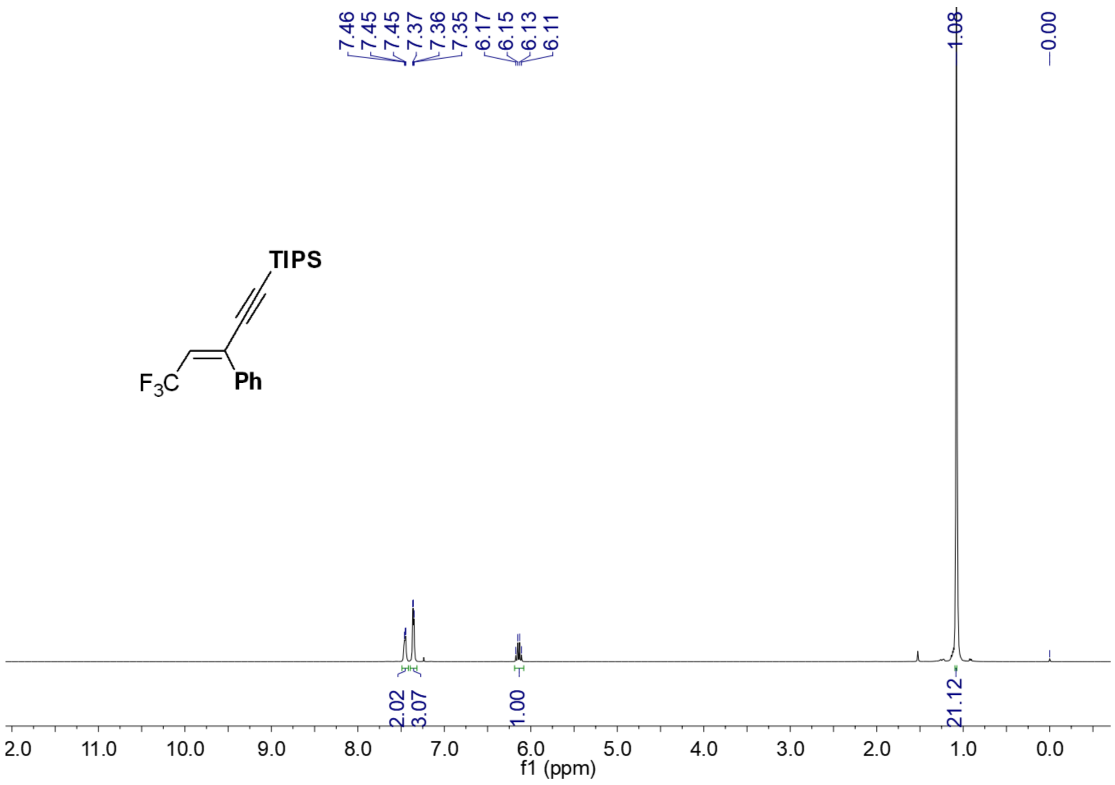

Supplementary Figure 227. ^{1}H NMR Spectra of product **14**

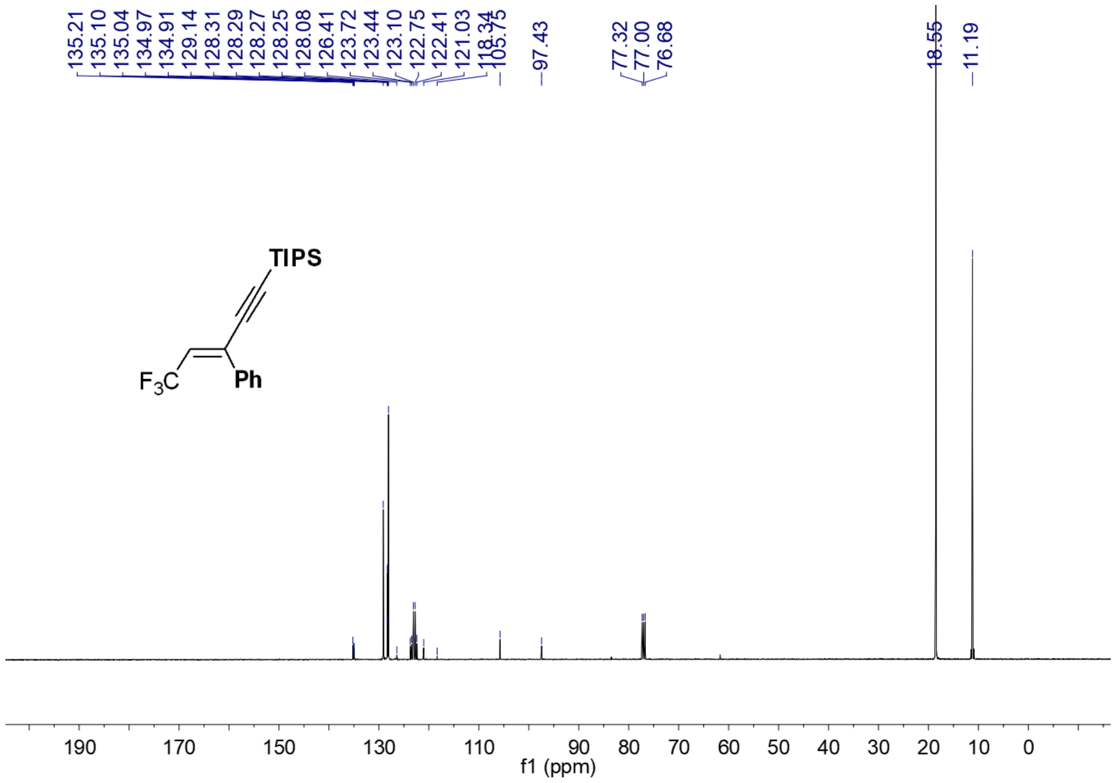

Supplementary Figure 228. ^{13}C NMR Spectra of product **14**

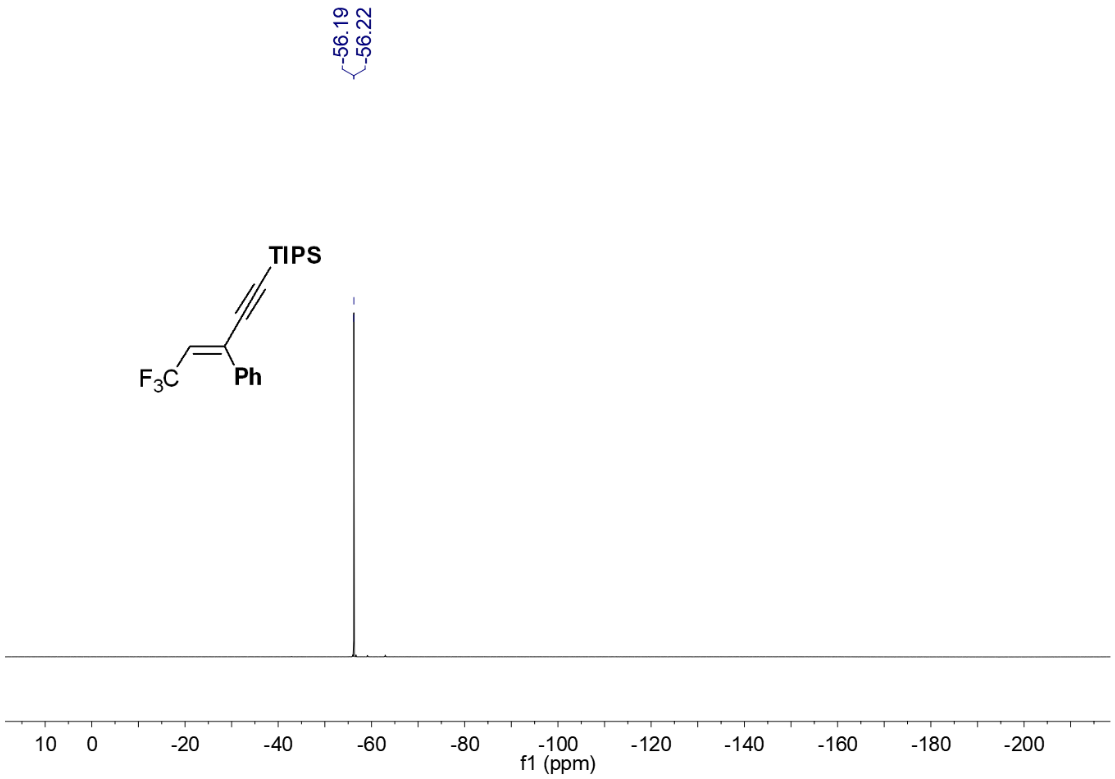

Supplementary Figure 229. ^{19}F NMR Spectra of product **14**

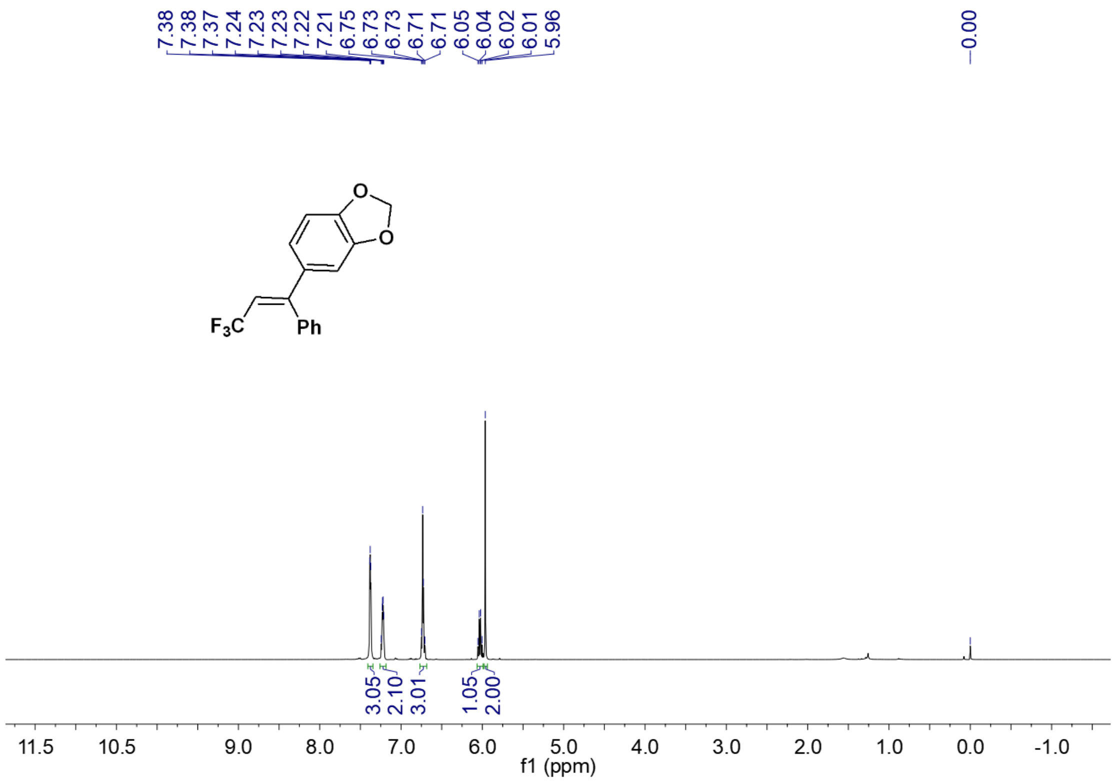

Supplementary Figure 230. ^{1}H NMR Spectra of product **15**

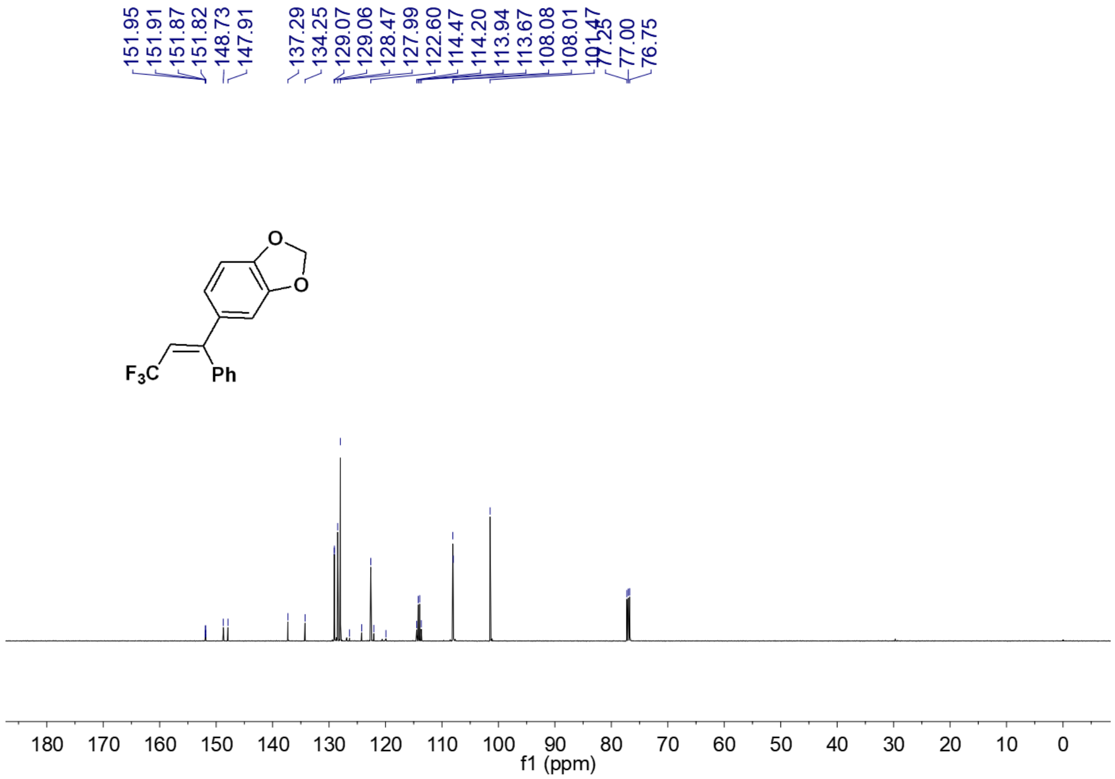

Supplementary Figure 231. ^{13}C NMR Spectra of product **15**

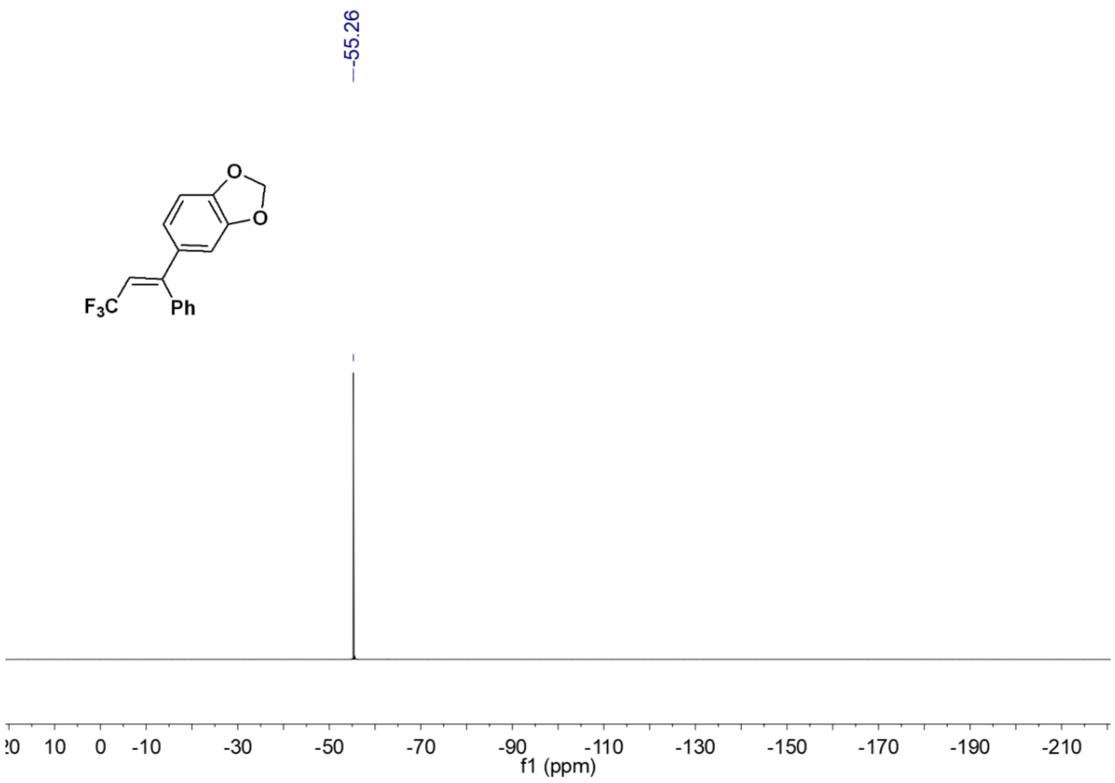

Supplementary Figure 232. ^{19}F NMR Spectra of product **15**

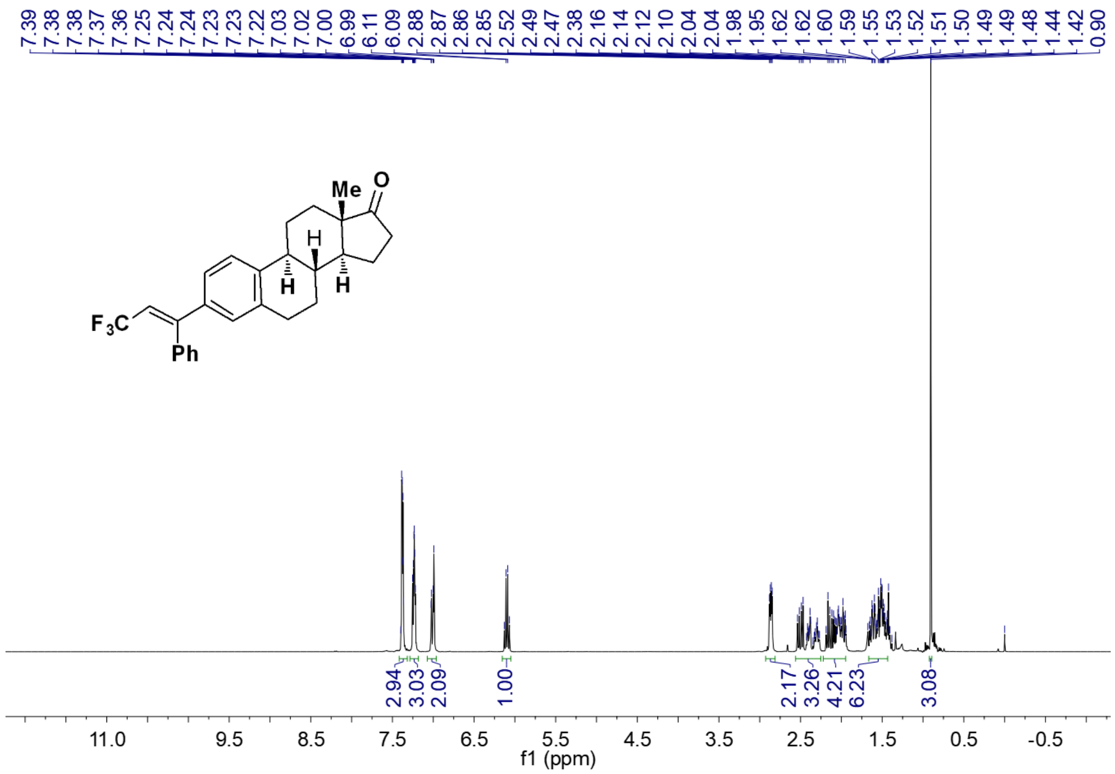

Supplementary Figure 233. ^{1}H NMR Spectra of product **16**

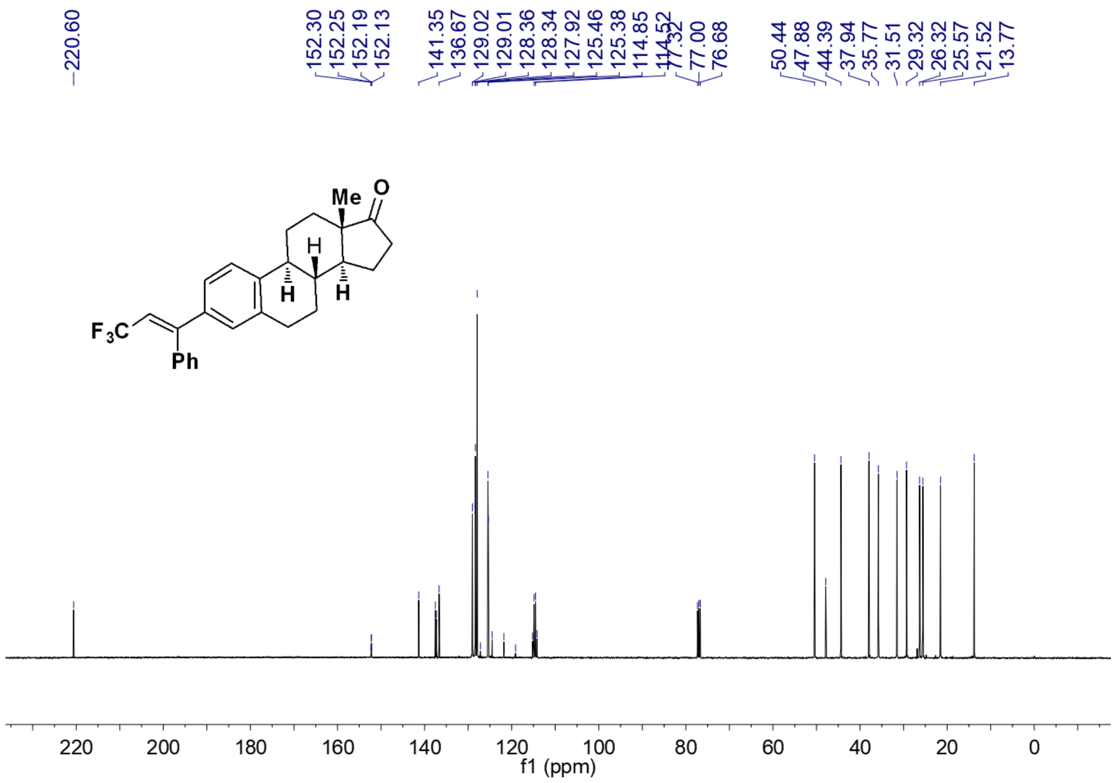

Supplementary Figure 234. ^{13}C NMR Spectra of product **16**

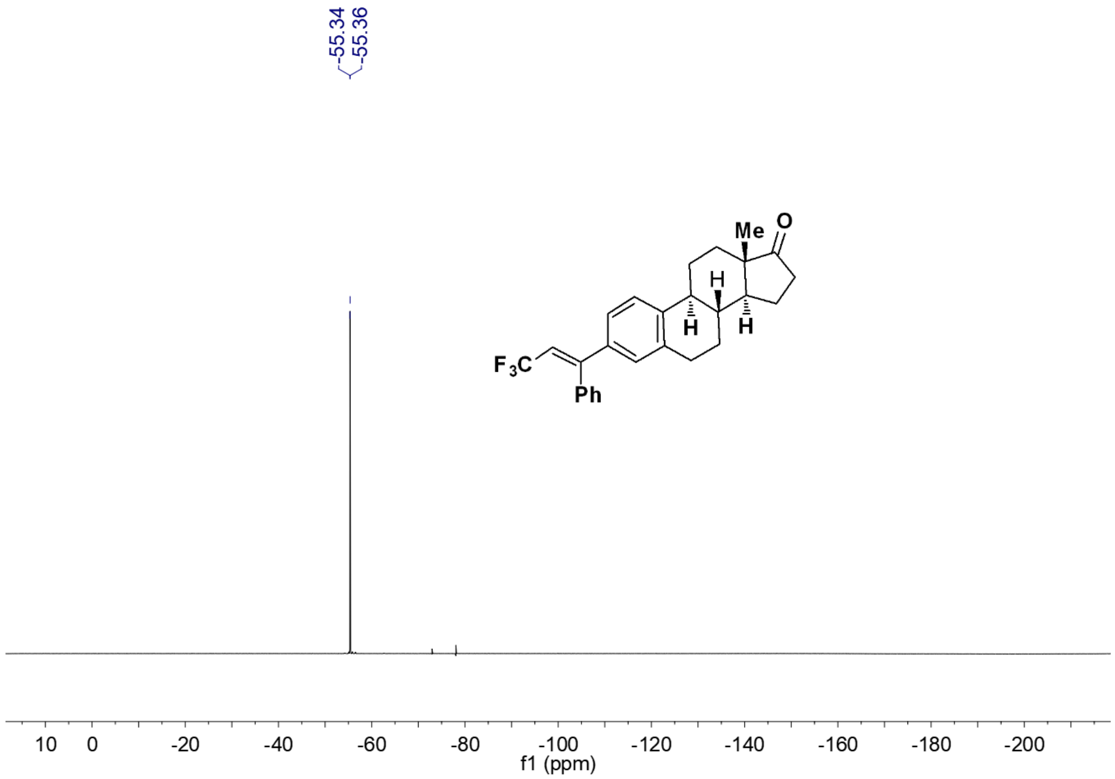

Supplementary Figure 235. ^{19}F NMR Spectra of product **16**

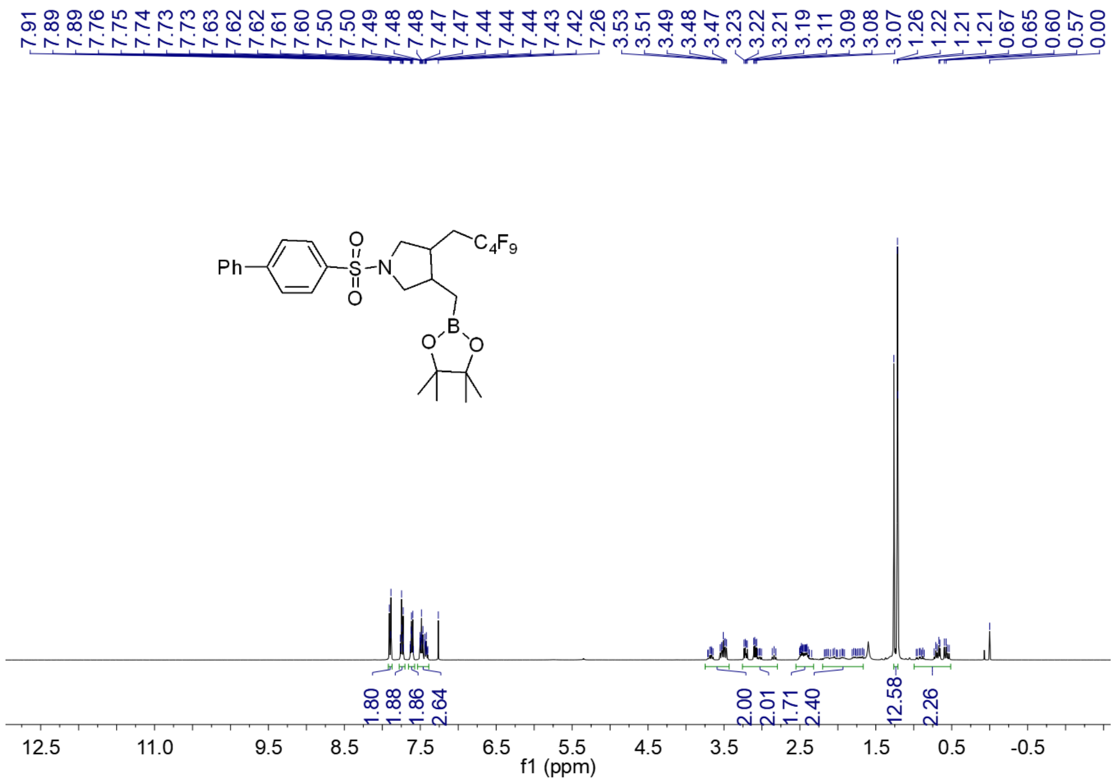

Supplementary Figure 236. ^{1}H NMR Spectra of product **18**

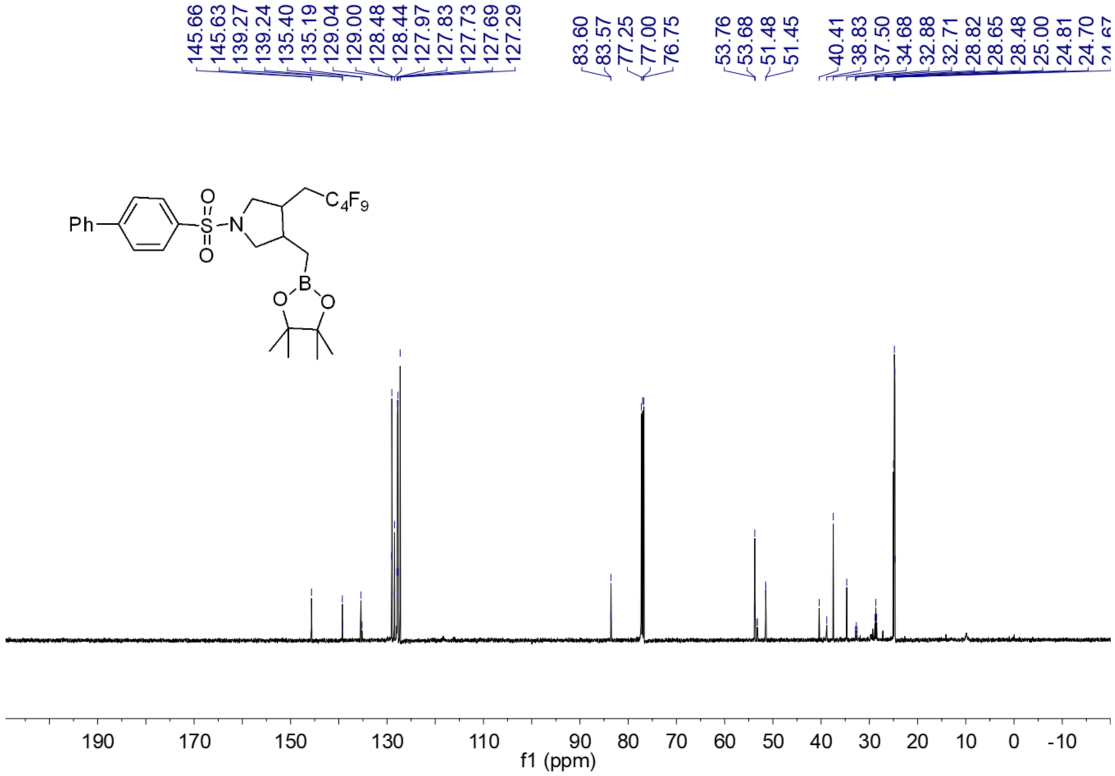

Supplementary Figure 237. ^{13}C NMR Spectra of product **18**

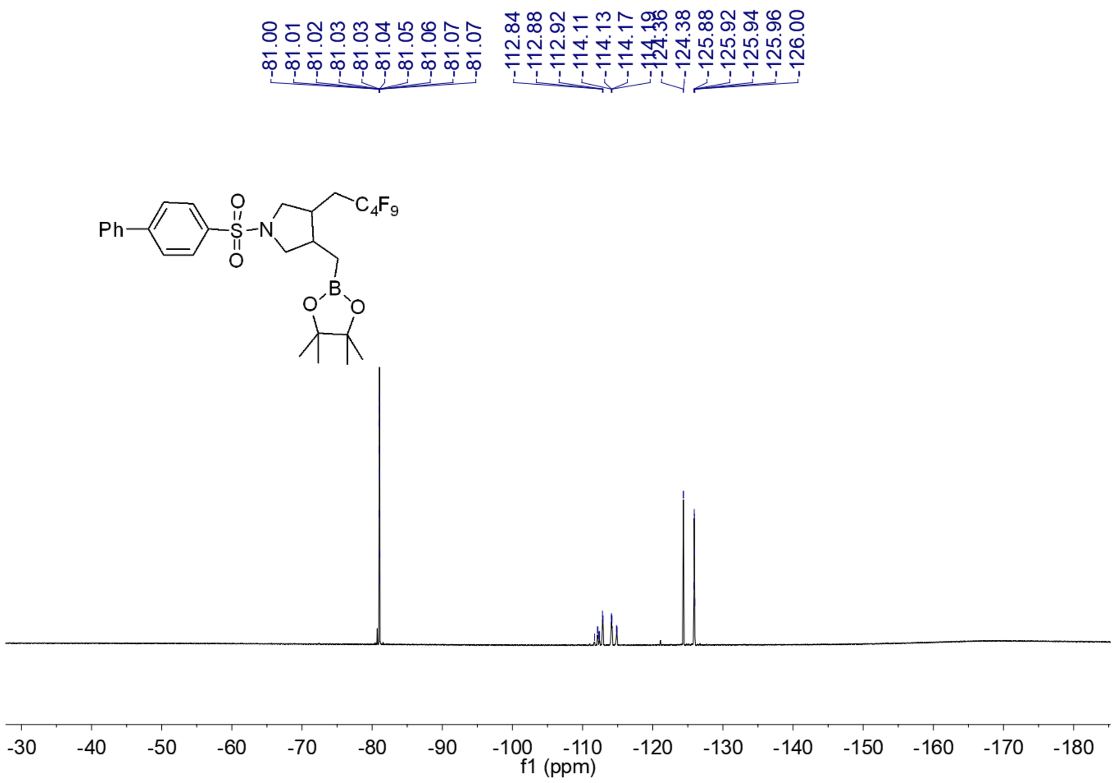

Supplementary Figure 238. ^{19}F NMR Spectra of product **18**

Supplementary References

1. Fukushima, M.; Takushima, D.; Satomura, H.; Onodera, G. and Kimura, M. Stereodefined Construction of Trisubstituted Alkenes by Direct Coupling Reaction of Allylating Agents, Alkynes, and Organoboranes. *Chem. Eur. J.* **18**, 8019-8023 (2012).
2. Lipshutz, B. H.; Ghorai, S.; Leong, W. W. Y. Deprotection of Homoallyl (^{h}Allyl) Derivatives of Phenols, Alcohols, Acids, and Amines. *J. Org. Chem.,* **74**, 2854-2857 (2009).
3. Mizuta, S.; Verhoog, S.; Engle, K. M.; Khotavivattana, T.; O'Duill, M.; Wheelhouse, K.; Rassias, G.; Médebielle, M.; Gouverneur, V. Catalytic Hydrotrifluoromethylation of Unactivated Alkenes. *J. Am. Chem. Soc,* **135**, 2505-2508 (2013).
4. Wu, X.; Chu, L.; Qing, F.-L. Silver- Catalyzed Hydrotrifluoromethylation of Unactivated Alkenes with CF_3SiMe_3. *Angew. Chem. Int. Ed.* **52**, 2198-2202 (2013).
5. Xu, J.; Fu, Y.; Luo, D.-F.; Jiang, Y.-Y.; Xiao, B.; Liu, Z.-J.; Gong, T.-J.; Liu, L. Copper-Catalyzed Trifluoromethylation of Terminal Alkenes through Allylic C–H Bond Activation. *J. Am. Chem. Soc.* **133**, 15300-15303 (2011).
6. Wu, Z.; Wang, D.; Liu, Y.; Huan, L.; Zhu, C. Chemo- and Regioselective Distal Heteroaryl *ipso*-Migration: A General Protocol for Heteroarylation of Unactivated Alkenes. *J. Am. Chem. Soc.* **139**, 1388-1391(2017).
7. Li, Y. W., Lu, Y., Qiu, G. and Ding, Q. P. Copper-Catalyzed Direct Trifluoromethylation of Propiolates: Construction of Trifluoromethylated Coumarins. *Org. Lett.* **16**, 4240−4243 (2014).
8. Wang, H.; Xu, Q. and Yu, S. Y. Visible Light-induced Aryltrifluoromethylation of Hydroxy Alkenes via Radical Trifluoromethylation-triggered Aryl and Heteroaryl Migration. *Org. Chem. Front.* **5**, 2224–2228 (2018).
9. Kischkewitz, M., Okamoto, K., Mück-Lichtenfeld, C., Studer, A. Radical-polar Crossover Reactions of Vinylboron ate Complexes. *Science*, **355**, 936-938 (2017).
10. Wu, N.-Y., Xu, X.-H. and Qing, F.-L. Copper-Catalyzed Regioselective Borylfluoromethylation of Alkenes. *ACS Catal.* **9**, 5726−5731 (2019).
11. Cheng, Y., Mück-Lichtenfeld, C. and Studer, A. Transition Metal-Free 1,2-Carboboration of Unactivated Alkenes. *J. Am. Chem. Soc.* **140**, 6221−6225 (2018).
12. Su, W., Gong, T.-J., Zhang, Q., Zhang, Q. Xiao, B. and Fu, Y. Ligand-Controlled Regiodivergent Copper-Catalyzed Alkylboration of Unactivated Terminal Alkynes. *ACS Catal.* **6**, 6417−6421 (2016).
13. Guo, W.-H., Zhao, H.-Y., Luo, Z.-J. Zhang, S., and Zhang, X.G. Fluoroalkylation–Borylation of Alkynes: An Efficient Method To Obtain (Z)-Tri- and Tetrasubstituted Fluoroalkylated Alkenylboronates. *ACS Catal.* **9**, 38−43 (2019).
14. Frisch, M. J.; Trucks, G. W.; Schlegel, H. B.; Scuseria, G. E.; Robb, M. A.; Cheeseman, J. R.; Scalmani, G.; Barone, V.; Mennucci, B.; Petersson, G. A.; Nakatsuji, H.; Caricato, M.; Li, X.; Hratchian, H. P.; Izmaylov, A. F.; Bloino, J.; Zheng, G.; Sonnenberg, J. L.; Hada, M.; Ehara,

M.; Toyota, K.; Fukuda, R.; Hasegawa, J.; Ishida, M.; Nakajima, T.; Honda, Y.; Kitao, O.; Nakai, H.; Vreven, T.; Montgomery, J. A., Jr.; Peralta, J. E.; Ogliaro, F.; Bearpark, M.; Heyd, J. J.; Brothers, E.; Kudin, K. N.; Staroverov, V. N.; Keith, T.; Kobayashi, R.; Normand, J.; Raghavachari, K.; Rendell, A.; Burant, J. C.; Iyengar, S. S.; Tomasi, J.; Cossi, M.; Rega, N.; Millam, J. M.; Klene, M.; Knox, J. E.; Cross, J. B.; Bakken, V.; Adamo, C.; Jaramillo, J.; Gomperts, R.; Stratmann, R. E.; Yazyev, O.; Austin, A. J.; Cammi, R.; Pomelli, C.; Ochterski, J. W.; Martin, R. L.; Morokuma, K.; Zakrzewski, V. G.; Voth, G. A.; Salvador, P.; Dannenberg, J. J.; Dapprich, S.; Daniels, A. D.; Farkas, O.; Foresman, J. B.; Ortiz, J. V.; Cioslowski, J.; Fox, D. J. Gaussian 09, revision D.01; Gaussian Inc., Wallingford, CT, 2013.

15. Becke, A. D. Density- functional Thermochemistry. III. The Role of Exact Exchange. *J. Chem. Phys.* **98**, 5648 (1993).
16. Lee, C.; Yang, W.; Parr, R. G. Development of the Colle-Salvetti Correlation-energy Formula into a Functional of the Electron Density. *Phys. Rev. B.* **37**, 785 (1998).
17. Grimme, S.; Antony, J.; Ehrlich, S.; Krieg, H. A Consistent and Accurate *ab initio* Parametrization of Density Functional Dispersion Correction (DFT-D) for the 94 Elements H-Pu. *J. Chem. Phys.* **132**, 154104-154123 (2010).
18. Barone, V.; Cossi, M. Quantum Calculation of Molecular Energies and Energy Gradients in Solution by a Conductor Solvent Model. *J. Phys. Chem. A*. **102**, 1995-2001 (1998).
19. Cossi, M.; Rega, N.; Scalmani, G.; Barone, V. Energies, Structures, and Electronic Properties of Molecules in Solution with the C- PCM Solvation Model. *J. Comput. Chem.* **24**, 669-681 (2003).
20. Takano, Y.; Houk, K. N. Benchmarking the Conductor-like Polarizable Continuum Model (CPCM) for Aqueous Solvation Free Energies of Neutral and Ionic Organic Molecules. *J. Chem. Theory Comput.* **1**, 70-77 (2005).
