# Supplementary material for: Integrated redox-active reagents for photoinduced regio- and stereoselective fluorocarboborylation
Source: Nat Commun. 2020 May 22;11:2572. doi: 10.1038/s41467-020-16477-1 (PMC7244735; doi:10.1038/s41467-020-16477-1)
Supplement: Supplementary file 3 — Description of Additional Supplementary Files [file 41467_2020_16477_MOESM3_ESM.pdf]

Description of Additional Supplementary Files

File Name: Supplementary Data 1
Description: Coordinates and energies of DFT-computed stationary points
