# Supplementary material for: Integrated redox-active reagents for photoinduced regio- and stereoselective fluorocarboborylation
Source: Nat Commun. 2020 May 22;11:2572. doi: 10.1038/s41467-020-16477-1 (PMC7244735; doi:10.1038/s41467-020-16477-1)
Supplement: Supplementary file 4 — Supplementary Data 1 [file 41467_2020_16477_MOESM4_ESM.pdf]

17

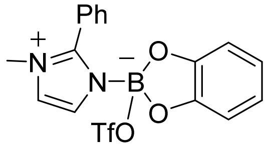

G(Ethyl acetate)=-1864.894789Hartree

C	2.859455	0.889238	-0.256301
C	2.367397	1.713183	0.766155
C	3.212068	2.494415	1.533926
C	4.588669	2.426607	1.249903
C	5.077310	1.604178	0.234574
C	4.209699	0.814677	-0.542869
H	2.822821	3.129454	2.322235
H	5.279050	3.025323	1.835424
H	6.143973	1.568863	0.037614
H	4.577590	0.171004	-1.334404
O	1.824923	0.236783	-0.874516
O	1.004089	1.609559	0.842753
B	0.634664	0.586931	-0.120661
C	-1.436884	1.582172	-3.033655
C	-0.243049	1.311910	-2.440044
C	-1.808221	1.145381	-0.881536
N	-2.404366	1.478021	-2.048852
H	-1.687442	1.841399	-4.048730
H	0.754008	1.266306	-2.841716
C	-3.829342	1.730846	-2.260172

H	-4.296659	1.963182	-1.305448
H	-3.936308	2.579923	-2.934932
H	-4.307442	0.852513	-2.697631
N	-0.484126	1.044548	-1.105417
C	-2.497210	0.914781	0.392312
C	-3.549675	-0.010592	0.458478
C	-2.085203	1.592105	1.548989
C	-4.187096	-0.250804	1.673243
H	-3.838686	-0.563749	-0.428047
C	-2.728819	1.345992	2.758720
H	-1.256692	2.288106	1.496734
C	-3.779513	0.428321	2.822763
H	-4.992823	-0.975381	1.723213
H	-2.408325	1.870180	3.652864
H	-4.275489	0.237360	3.768886
S	-0.385839	-1.970704	0.133511
O	0.105820	-0.593114	0.690478
O	-1.024534	-2.693138	1.228308
O	-1.060285	-1.850402	-1.159403
C	1.232104	-2.858709	-0.200205
F	1.008079	-4.172518	-0.110279
F	1.672868	-2.573482	-1.422288
F	2.144799	-2.502397	0.703044

18

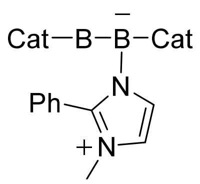

G(Ethyl acetate)=-1309.668546Hartree

C	1.773758	-2.573308	0.274102
C	1.939241	-1.755744	-0.858810
C	3.008702	-1.928707	-1.721513
C	3.926104	-2.956158	-1.428251
C	3.760566	-3.768488	-0.307394
C	2.671686	-3.585772	0.567205
H	3.127848	-1.289618	-2.590353
H	4.774842	-3.113136	-2.086918
H	4.480885	-4.553877	-0.099838
H	2.535377	-4.212513	1.442519
O	0.646851	-2.221098	0.958289
O	0.926679	-0.849204	-0.949274
B	0.198901	-0.947225	0.337505
B	-1.500303	-0.784657	0.214996
O	-2.352113	-1.707622	-0.404189
O	-2.256941	0.299188	0.692745
C	-3.624896	-1.187682	-0.311756
C	-3.568218	0.036512	0.355178
C	-4.818584	-1.715515	-0.773278
C	-4.699408	0.797141	0.594098

C	-5.973863	-0.955061	-0.537332
H	-4.853261	-2.667622	-1.290325
C	-5.915956	0.273480	0.130811
H	-4.643381	1.747515	1.112695
H	-6.932096	-1.329767	-0.881828
H	-6.829812	0.834818	0.295201
C	1.592458	1.177879	3.153434
C	1.039705	0.035401	2.654716
C	1.206582	1.456850	0.979337
N	1.691747	2.061531	2.094800
H	1.920816	1.442607	4.145034
H	0.811134	-0.903041	3.130259
C	2.220876	3.420276	2.187063
H	1.842058	4.007878	1.352910
H	1.888185	3.864150	3.125416
H	3.312337	3.407310	2.156033
N	0.807331	0.220333	1.307646
C	1.144820	2.083074	-0.350782
C	2.324722	2.494252	-0.985283
C	-0.090916	2.270456	-0.984530
C	2.268434	3.080115	-2.248636
H	3.281790	2.335265	-0.499252
C	-0.139812	2.855727	-2.247574
H	-1.000453	1.959382	-0.484308
C	1.037218	3.259570	-2.881617
H	3.185229	3.389438	-2.739724

H	-1.098202	2.998368	-2.736190
H	0.995040	3.713312	-3.866639

1e

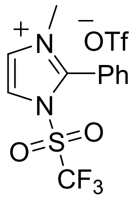

G(Ethyl acetate)=-2344.729386Hartree

C	0.785685	-0.034614	2.462225
C	0.462118	-1.121851	1.724070
C	-0.670883	0.657373	0.917730
N	0.068312	1.043242	1.960425
H	1.475027	0.077651	3.281530
H	0.797524	-2.142874	1.751617
C	0.275947	2.433919	2.367956
H	-0.587554	3.024439	2.070711
H	0.396047	2.468641	3.449630
H	1.172936	2.804148	1.869084
C	-1.602035	1.496570	0.149037
C	-1.245965	1.980370	-1.116616
C	-2.846439	1.812035	0.714847
C	-2.150973	2.774000	-1.817282
H	-0.269456	1.737943	-1.515932
C	-3.740827	2.607936	0.003364

H	-3.110857	1.426037	1.693541
C	-3.393779	3.085599	-1.261865
H	-1.883071	3.151607	-2.798225
H	-4.706008	2.850283	0.434684
H	-4.093297	3.703692	-1.815289
N	-0.453613	-0.679307	0.773418
S	-1.104403	-1.721498	-0.472221
O	-1.060037	-1.033609	-1.745162
O	-0.530043	-3.025624	-0.203423
C	-2.922200	-1.784132	0.083769
F	-3.618486	-0.903054	-0.617008
F	-3.001841	-1.505841	1.381953
F	-3.355166	-3.013091	-0.151297
S	2.509664	0.072851	-1.028050
O	1.912644	-1.273494	-0.850749
O	3.072237	0.345675	-2.365482
O	1.710376	1.186037	-0.447281
C	3.998493	0.024807	0.081589
F	3.640632	-0.212753	1.358279
F	4.844973	-0.944564	-0.298185
F	4.654929	1.195450	0.047361

1-phenylpropyne

G(Ethyl acetate)=-347.7508653Hartree

C	-2.144448	1.208227	0.000033

C	-0.751996	1.212658	0.000040
C	-0.036211	0.000005	-0.000006
C	-0.751978	-1.212655	0.000042
C	-2.144431	-1.208246	0.000031
C	-2.845477	-0.000015	0.000015
H	-2.684102	2.150164	0.000067
H	-0.205205	2.149615	0.000108
H	-0.205169	-2.149599	0.000108
H	-2.684071	-2.150191	0.000061
H	-3.930869	-0.000023	0.000036
C	1.392895	0.000019	-0.000121
C	2.605747	0.000059	-0.000447
C	4.064287	-0.000000	0.000022
H	4.460100	-0.047566	1.020610
H	4.459533	-0.860421	-0.550388
H	4.459450	0.907709	-0.468248

3a

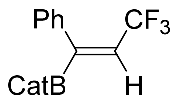

G(Ethyl acetate)=-1052.756314Hartree

C	1.877277	3.145876	-0.869259
C	1.140971	1.963108	-0.880231
C	1.466666	0.909353	-0.011266
C	2.526762	1.083604	0.892567

C	3.252537	2.272505	0.912223
C	2.934436	3.305428	0.028515
H	1.621037	3.945187	-1.557501
H	0.313511	1.849565	-1.572387
H	2.775293	0.287120	1.583951
H	4.065836	2.392846	1.621019
H	3.501780	4.230682	0.044105
C	1.158899	-1.590284	-0.029575
H	0.484459	-2.439489	0.011402
C	2.598811	-1.999345	-0.092531
F	3.365177	-1.184439	-0.845869
F	3.171891	-2.054865	1.140737
F	2.702089	-3.241437	-0.619187
C	0.660737	-0.337814	-0.027499
B	-0.890100	-0.224207	-0.006216
O	-1.599242	0.964979	0.094319
O	-1.748195	-1.317534	-0.070532
C	-2.932486	0.600242	0.094315
C	-3.022614	-0.788984	-0.004044
C	-4.053173	1.407009	0.175315
C	-4.239279	-1.447038	-0.026636
C	-5.293651	0.752656	0.153643
H	-3.971245	2.484905	0.251510
C	-5.384302	-0.640919	0.055206
H	-4.298844	-2.526366	-0.103242
H	-6.202000	1.342422	0.215008

H	-6.361794	-1.111092	0.041351

----

bis(catecholato)diboron

G(Ethyl acetate)=-813.0317044Hartree

C	0.697504	2.937011	0.000294
C	-0.697504	2.937011	-0.000294
C	-1.433590	4.109418	-0.000550
C	-0.700400	5.304255	-0.000248
C	0.700400	5.304255	0.000248
C	1.433590	4.109418	0.000550
H	-2.517268	4.098400	-0.000961
H	-1.232433	6.249660	-0.000437
H	1.232433	6.249660	0.000437
H	2.517268	4.098400	0.000961
O	1.145402	1.630678	0.000490
O	-1.145402	1.630678	-0.000490
B	-0.000000	0.840641	-0.000000
B	-0.000000	-0.840641	-0.000000
O	-1.145402	-1.630678	0.000490
O	1.145402	-1.630678	-0.000490
C	-0.697504	-2.937011	0.000294
C	0.697504	-2.937011	-0.000294
C	-1.433590	-4.109418	0.000550
C	1.433590	-4.109418	-0.000550
C	-0.700400	-5.304255	0.000248

H	-2.517268	-4.098400	0.000961
C	0.700400	-5.304255	-0.000248
H	2.517268	-4.098400	-0.000961
H	-1.232433	-6.249660	0.000437
H	1.232433	-6.249660	-0.000437

***fac*-Ir(ppy)$_3$$^+$**

G(Ethyl acetate)=-1540.715427Hartree

Ir	-0.000421	0.000174	0.063327
N	1.092589	-1.491280	-1.072715
C	0.952551	-2.756005	-0.584553
C	1.638781	-3.813368	-1.196392
C	2.599055	-2.255691	-2.760088
C	1.896482	-1.246364	-2.120610
C	-0.533786	-1.666733	1.042558
C	0.065057	-2.873240	0.574735
C	-0.224760	-4.097883	1.192240
C	-1.103656	-4.145713	2.269429
C	-1.711004	-2.970013	2.728430
C	-1.438912	-1.750290	2.114279
H	1.527107	-4.821295	-0.818299
H	3.235679	-2.021463	-3.604384
H	1.961955	-0.213242	-2.439403
H	0.228173	-5.017429	0.837711
H	-1.320460	-5.094820	2.748004

H	-2.396021	-3.010095	3.569961
H	-1.910563	-0.846954	2.484153
N	0.746629	1.691096	-1.072707
N	-1.837970	-0.199320	-1.073509
C	1.911996	2.201680	-0.584126
C	-2.863186	0.554396	-0.585452
C	2.486450	3.322955	-1.197382
C	-4.121493	0.490183	-1.198486
C	1.859760	3.910057	-2.287825
C	0.658912	3.375212	-2.763010
C	-3.252078	-1.118962	-2.763267
C	0.134240	2.263768	-2.122199
C	-2.027193	-1.016641	-2.122541
C	1.708795	0.371696	1.044602
C	-1.177159	1.294629	1.043307
C	2.455368	1.492802	0.576675
C	-2.521338	1.380263	0.574884
C	3.660073	1.854398	1.195554
C	-3.436959	2.243214	1.192821
C	4.139128	1.118093	2.274161
C	-3.039181	3.026855	2.271164
C	3.423619	0.004913	2.733199
C	-1.717569	2.964016	2.730857
C	2.231954	-0.369495	2.117776
C	-0.797279	2.118971	2.116246
H	3.415318	3.729716	-0.819160

H	-4.938564	1.090845	-0.820444
H	2.300234	4.779266	-2.763911
H	0.139045	3.808552	-3.608552
H	-3.367142	-1.786336	-3.608475
H	-0.793452	1.804368	-2.441058
H	-1.165223	-1.590036	-2.441184
H	4.230782	2.705931	0.841155
H	-4.459555	2.311615	0.837839
H	5.068858	1.405106	2.753817
H	-3.752745	3.688895	2.750094
H	3.799553	-0.567610	3.575766
H	-1.410136	3.576248	3.573276
H	1.684728	-1.229162	2.487653
H	0.220640	2.074921	2.486570
C	2.462432	-3.563234	-2.285220
H	2.996911	-4.378649	-2.760209
C	-4.316146	-0.346847	-2.288459
H	-5.289142	-0.400930	-2.764420

***fac*-Ir(ppy)$_3$_singlet**

G(Ethylacetate)=-1540.909844Hartree

Ir	0.000012	-0.000226	0.033648
N	1.833923	0.218794	-1.087467
C	2.876258	-0.512754	-0.601549
C	4.123504	-0.449456	-1.240356

C	3.210705	1.101199	-2.835901
C	1.999990	1.002210	-2.165414
C	1.225931	-1.217214	1.074534
C	2.561002	-1.314589	0.584445
C	3.514670	-2.128854	1.218662
C	3.171899	-2.862451	2.348297
C	1.865430	-2.782326	2.845652
C	0.916399	-1.976459	2.217869
H	4.954811	-1.028625	-0.858359
H	3.300579	1.744581	-3.703044
H	1.124611	1.556541	-2.483342
H	4.528786	-2.192232	0.835415
H	3.910744	-3.490138	2.836615
H	1.589933	-3.352655	3.729373
H	-0.088813	-1.926270	2.624018
N	-1.109283	1.478525	-1.085184
N	-0.727500	-1.700474	-1.083765
C	-0.994885	2.747240	-0.600302
C	-1.882954	-2.235521	-0.597489
C	-1.675137	3.795635	-1.237379
C	-2.451678	-3.348874	-1.233680
C	-2.460900	3.538679	-2.352861
C	-2.567703	2.229272	-2.828968
C	-0.651244	-3.338624	-2.827891
C	-1.874699	1.230361	-2.160330
C	-0.131556	-2.239184	-2.159841

C	0.444284	1.670415	1.072434
C	-1.667271	-0.450976	1.075203
C	-0.139064	2.875468	0.582966
C	-2.420208	-1.558361	0.586446
C	0.092396	4.108775	1.215582
C	-3.603577	-1.974134	1.220148
C	0.902267	4.178725	2.342960
C	-4.067512	-1.307981	2.348291
C	1.486137	3.006973	2.839725
C	-3.343397	-0.217245	2.844828
C	1.259893	1.781941	2.213405
C	-2.169982	0.199111	2.217214
H	-1.587689	4.805289	-0.856125
H	-3.369207	-3.778019	-0.851102
H	-2.987480	4.349157	-2.845904
H	-3.173046	1.985184	-3.693798
H	-0.138493	-3.740669	-3.693567
H	-1.918976	0.194956	-2.477568
H	0.786696	-1.759688	-2.478459
H	-0.359777	5.018957	0.832915
H	-4.166697	-2.820289	0.837734
H	1.078818	5.132606	2.830070
H	-4.981548	-1.631685	2.836232
H	2.120204	3.053551	3.721727
H	-3.699465	0.308364	3.727486
H	1.719121	0.886106	2.618991

H	-1.623112	1.044487	2.622444
C	4.292186	0.355991	-2.358653
H	5.256753	0.405504	-2.853106
C	-1.837914	-3.900888	-2.350067
H	-2.277240	-4.762161	-2.842522

***fac*-Ir(ppy)$_3$_triplet**

G(Ethyl acetate)=-1540.821866Hartree

Ir	-0.011272	0.021320	0.060872
N	1.193075	1.424177	-1.075608
C	2.440827	1.619251	-0.564543
C	3.316707	2.502555	-1.212900
C	1.618386	2.955609	-2.859019
C	0.793034	2.070412	-2.181970
C	1.706749	0.005006	1.139774
C	2.742057	0.849647	0.648380
C	3.984831	0.928707	1.299426
C	4.226524	0.175358	2.442396
C	3.222571	-0.665536	2.936206
C	1.987945	-0.746713	2.291727
H	4.311963	2.663181	-0.818414
H	1.261527	3.461151	-3.748247
H	-0.217263	1.854239	-2.509968
H	4.767589	1.578717	0.920490
H	5.186778	0.240127	2.944268

H	3.406174	-1.256900	3.829604
H	1.219960	-1.400214	2.693931
N	-1.743156	0.275298	-1.156635
N	0.729265	-1.721146	-1.032795
C	-2.606175	1.282796	-0.666296
C	0.237098	-2.904196	-0.574438
C	-3.813996	1.553998	-1.380766
C	0.639523	-4.102940	-1.181103
C	-4.146195	0.833724	-2.497500
C	-3.269980	-0.203946	-2.944813
C	2.040253	-2.850859	-2.683188
C	-2.098799	-0.429797	-2.236694
C	1.607226	-1.696349	-2.047469
C	-0.862864	1.492672	1.065794
C	-0.947797	-1.472804	1.035678
C	-2.168647	1.921461	0.510351
C	-0.690663	-2.787585	0.554956
C	-2.889876	2.958003	1.181951
C	-1.302464	-3.905386	1.146255
C	-2.363579	3.553883	2.305148
C	-2.172583	-3.746357	2.218220
C	-1.099281	3.165983	2.834980
C	-2.437457	-2.460202	2.702246
C	-0.379524	2.152950	2.207284
C	-1.835927	-1.346649	2.117943
H	-4.466910	2.344158	-1.026918

H	0.249357	-5.047212	-0.823183
H	-5.063347	1.045130	-3.037114
H	-3.501042	-0.805756	-3.815265
H	2.747268	-2.787645	-3.501625
H	-1.401260	-1.203838	-2.541859
H	1.958169	-0.715895	-2.346811
H	-3.856424	3.280201	0.808988
H	-1.101982	-4.904623	0.772594
H	-2.926260	4.340724	2.800943
H	-2.641044	-4.613403	2.672864
H	-0.713872	3.654328	3.723994
H	-3.116273	-2.328028	3.540646
H	0.576701	1.843350	2.616105
H	-2.045636	-0.357766	2.512067
C	2.906126	3.170132	-2.358974
H	3.583249	3.854002	-2.860071
C	1.539936	-4.076966	-2.237470
H	1.852318	-5.003175	-2.708287

IMD

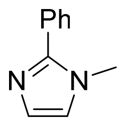

G(Ethyl acetate)=-496.6328677Hartree

C	3.038633	0.053096	-0.011753
C	2.807190	-1.268616	0.279574

C	0.862104	-0.349148	0.096646
N	1.795103	0.638698	-0.130618
H	3.944018	0.622797	-0.152267
H	3.535732	-2.049394	0.448247
C	1.576315	2.023701	-0.529766
H	0.664708	2.096630	-1.122534
H	1.491610	2.681272	0.339796
H	2.420317	2.350189	-1.138352
N	1.457476	-1.514121	0.341959
C	-0.594774	-0.169442	0.057953
C	-1.387563	-1.238128	-0.395901
C	-1.231224	1.004344	0.493653
C	-2.774302	-1.128494	-0.429220
H	-0.895527	-2.148265	-0.719761
C	-2.621186	1.113056	0.451766
H	-0.648100	1.824362	0.895967
C	-3.397574	0.050944	-0.012173
H	-3.370847	-1.962061	-0.786899
H	-3.096620	2.026610	0.794980
H	-4.479058	0.137950	-0.043234

Int1

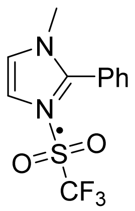

G(Ethyl acetate)=-1383.036263Hartree

C	-1.028626	3.204779	-0.301214
C	0.140979	2.669369	-0.775740
C	-1.186627	0.991812	-0.367162
N	-1.867930	2.136718	-0.045103
H	-1.345228	4.221774	-0.131811
H	1.045461	3.174837	-1.079161
C	-3.250610	2.262789	0.404382
H	-3.834842	1.425518	0.024486
H	-3.308931	2.278009	1.495611
H	-3.663213	3.192347	0.011506
C	-1.688058	-0.380855	-0.245861
C	-1.320509	-1.320450	-1.223360
C	-2.467866	-0.799261	0.843703
C	-1.729024	-2.646631	-1.115067
H	-0.719058	-0.997366	-2.065210
C	-2.883055	-2.126194	0.942087
H	-2.722718	-0.099855	1.631646
C	-2.516135	-3.052829	-0.035043
H	-1.437100	-3.362139	-1.876923
H	-3.481896	-2.438335	1.791662
H	-2.837326	-4.086231	0.046838
N	0.026504	1.302581	-0.821562
S	2.097999	-0.381807	-0.801740
O	2.188208	-1.864953	-0.938106

O	3.235631	0.476263	-1.227736
C	2.009517	-0.132202	1.084516
F	0.869236	-0.649042	1.539368
F	2.064957	1.164899	1.369471
F	3.038720	-0.758876	1.658397

Int2

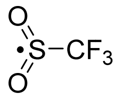

G(Ethyl acetate)=-886.4054789Hartree

S	-1.018967	-0.000041	0.340309
O	-1.493531	-1.296812	-0.204206
O	-1.493477	1.296820	-0.204260
C	0.882289	-0.000025	-0.007510
F	1.085670	-0.000017	-1.317747
F	1.396483	-1.090107	0.540414
F	1.396271	1.090206	0.540427

Int3

• CF_3

G(Ethyl acetate)=-337.6948773Hartree

C	0.000000	0.000000	0.329939
F	-0.000000	1.264495	-0.073320
F	-1.095085	-0.632248	-0.073320

F	1.095085	-0.632248	-0.073320

Int4

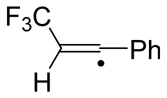

G(Ethyl acetate)=-646.1762248Hartree

C	-3.194623	1.213284	-0.149678
C	-1.850524	1.229916	0.177930
C	-1.136194	0.000650	0.352027
C	-1.849369	-1.229343	0.178439
C	-3.193497	-1.214094	-0.149181
C	-3.878267	-0.000767	-0.315930
H	-3.723101	2.152583	-0.279173
H	-1.320513	2.167053	0.306081
H	-1.318495	-2.165942	0.306962
H	-3.721075	-2.153953	-0.278292
H	-4.932165	-0.001306	-0.572652
C	0.194728	0.001220	0.679714
C	1.464628	0.000406	0.964928
H	1.846667	0.000347	1.987743
C	2.540824	-0.000091	-0.085410
F	2.046199	-0.000813	-1.337730
F	3.342524	1.085425	0.035582
F	3.342659	-1.085264	0.036848

Int4-a

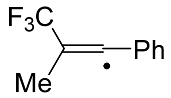

G(Ethyl acetate)=-685.4856299Hartree

C	-1.373277	0.726827	0.003359
C	-2.264333	-0.494485	-0.002323
C	3.341331	-0.130120	-1.214046
C	1.986188	0.149752	-1.227775
C	1.264632	0.290661	0.001377
C	1.986083	0.137771	1.229162
C	3.341203	-0.141993	1.212798
C	4.031375	-0.278128	-0.001293
H	3.873416	-0.236653	-2.154323
H	1.451538	0.263489	-2.164320
H	1.451352	0.242337	2.166725
H	3.873231	-0.257736	2.152020
H	5.093833	-0.497066	-0.002285
C	-0.076685	0.580339	0.002679
F	-1.576384	-1.651065	-0.007092
F	-3.077872	-0.512134	1.083475
F	-3.077131	-0.502688	-1.088719
C	-2.111459	2.049946	0.009458
H	-2.752389	2.131225	-0.874172
H	-2.752687	2.122956	0.893575
H	-1.406157	2.881008	0.013434

Int4-b

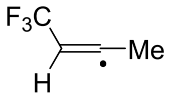

G(Ethyl acetate)=-454.4048413Hartree

C	0.500872	-0.592167	-0.000152
C	-0.876037	-0.012796	-0.000022
C	1.590324	0.142898	0.000065
H	0.530953	-1.685313	-0.000495
F	-0.878918	1.335811	0.001224
F	-1.584179	-0.415806	-1.085255
F	-1.584726	-0.417871	1.084175
C	3.048354	0.004989	-0.000017
H	3.486739	0.481483	0.883623
H	3.344936	-1.054991	-0.000317
H	3.486696	0.482055	-0.883355

Int5

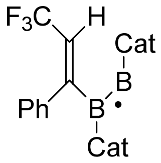

G(Ethyl acetate)=-1459.212988Hartree

B	0.784032	0.614191	-0.100597
C	0.817026	-1.008234	-0.090818

C	-2.869362	1.271704	-1.217824
C	-2.933252	1.720621	0.101428
C	-4.112776	2.169704	0.668490
C	-5.248998	2.150538	-0.154427
C	-5.184788	1.700560	-1.478269
C	-3.981516	1.248531	-2.040725
H	-4.151826	2.515411	1.695032
H	-6.197331	2.492014	0.247019
H	-6.084221	1.699246	-2.084983
H	-3.921353	0.897091	-3.064293
O	-1.572559	0.886331	-1.498109
O	-1.676973	1.626499	0.668919
B	-0.851364	1.100692	-0.323552
O	1.700640	1.196698	-1.146602
O	1.353800	1.179994	1.189685
C	2.596981	1.946370	-0.537507
C	2.385290	1.935766	0.879829
C	3.638172	2.686130	-1.113142
C	3.213613	2.663880	1.745568
C	4.449975	3.397880	-0.245011
H	3.789662	2.689276	-2.185622
C	4.241022	3.386680	1.162745
H	3.045804	2.649659	2.815528
H	5.271362	3.983224	-0.643458
H	4.909887	3.963807	1.791706
C	-2.105441	-3.102131	1.111510

C	-1.127586	-2.550039	0.286418
C	-0.167343	-1.666468	0.806852
C	-0.226598	-1.333769	2.169874
C	-1.196704	-1.898830	2.995804
C	-2.141221	-2.782353	2.469891
H	-2.841597	-3.780725	0.691650
H	-1.102797	-2.798027	-0.768419
H	0.492396	-0.632752	2.578154
H	-1.220486	-1.641118	4.050190
H	-2.903518	-3.212350	3.112054
C	1.631599	-1.654012	-0.939033
H	2.263740	-1.097675	-1.622579
C	1.780791	-3.134626	-1.084381
F	3.012334	-3.436126	-1.565733
F	1.631771	-3.821254	0.069584
F	0.888604	-3.665406	-1.968963

Int6

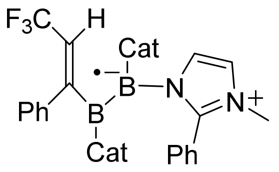

G(Ethyl acetate)=-1955.872552Hartree

B	-0.063745	-1.272839	0.475646
C	-1.582423	-0.972402	0.732635

C	-1.603329	0.173635	-2.497047
C	-1.458691	1.350087	-1.747292
C	-2.428662	2.335159	-1.738672
C	-3.567868	2.112057	-2.530615
C	-3.709443	0.944756	-3.284351
C	-2.721741	-0.053999	-3.279113
H	-2.311616	3.231547	-1.140226
H	-4.353452	2.860397	-2.549514
H	-4.604000	0.798553	-3.880978
H	-2.833311	-0.972173	-3.844130
O	-0.522773	-0.644998	-2.296489
O	-0.272186	1.320956	-1.057835
B	0.244887	-0.000162	-1.265481
O	0.339873	-2.567274	-0.048641
O	0.951143	-0.868452	1.429964
C	1.588954	-2.805229	0.442328
C	1.969465	-1.769659	1.310562
C	2.443629	-3.856935	0.153730
C	3.228852	-1.724623	1.884927
C	3.719456	-3.825473	0.739723
H	2.142501	-4.653419	-0.517609
C	4.108102	-2.778388	1.580751
H	3.517825	-0.908107	2.537452
H	4.417917	-4.628931	0.528942
H	5.102430	-2.780408	2.015678
C	3.670346	-1.143105	-1.893136

C	2.324781	-1.226733	-2.058829
C	2.736240	0.614144	-0.882105
N	3.912937	0.002937	-1.159393
H	4.467265	-1.797478	-2.203378
H	1.714933	-1.966014	-2.547358
C	5.228674	0.386712	-0.652687
H	5.135971	1.312064	-0.089504
H	5.596799	-0.405577	0.001809
H	5.916533	0.532893	-1.486402
N	1.748580	-0.135601	-1.422611
C	2.616855	1.918919	-0.208134
C	3.158984	3.050027	-0.838559
C	1.994604	2.047790	1.040204
C	3.068417	4.299417	-0.228392
H	3.635462	2.948914	-1.808387
C	1.904416	3.301717	1.641014
H	1.582644	1.171983	1.523338
C	2.438054	4.427292	1.010873
H	3.483486	5.170992	-0.723649
H	1.416522	3.395292	2.605827
H	2.364308	5.401463	1.483617
C	-1.717003	2.141488	2.912929
C	-1.390714	1.275163	1.870913
C	-1.956494	-0.008207	1.801765
C	-2.840933	-0.402636	2.819878
C	-3.158495	0.462209	3.866121

C	-2.599146	1.740077	3.917884
H	-1.278843	3.135286	2.937165
H	-0.716205	1.600207	1.091169
H	-3.278081	-1.393832	2.786671
H	-3.842797	0.134724	4.643209
H	-2.847003	2.413892	4.732365
C	-2.486512	-1.630963	-0.026290
H	-2.150225	-2.371183	-0.743639
C	-3.959650	-1.413779	-0.061454
F	-4.635716	-2.133665	0.886066
F	-4.330701	-0.124849	0.108917
F	-4.466493	-1.815975	-1.255275

Int7

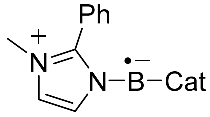

G(Ethyl acetate)=-903.121823Hartree

C	-3.027053	-0.673294	0.131294
C	-2.517103	0.565841	-0.264835
C	-3.339033	1.637016	-0.560830
C	-4.721557	1.417619	-0.445095
C	-5.232261	0.177912	-0.049427
C	-4.385461	-0.902081	0.249761
H	-2.931446	2.593806	-0.865965
H	-5.404826	2.230135	-0.668453

H	-6.305850	0.043399	0.029972
H	-4.771498	-1.866835	0.557955
O	-1.980649	-1.544102	0.362871
O	-1.136813	0.505326	-0.296737
B	-0.827372	-0.795899	0.093464
C	1.910060	-3.070793	-0.051394
C	0.627266	-2.785946	0.232028
C	1.738031	-0.807926	-0.066689
N	2.608808	-1.887002	-0.271696
H	2.404419	-4.026723	-0.121924
H	-0.215754	-3.432765	0.404144
C	4.058357	-1.809594	-0.175510
H	4.350655	-1.123484	0.625055
H	4.442976	-2.803912	0.052384
H	4.510653	-1.469093	-1.110350
N	0.466813	-1.376122	0.188958
C	2.090038	0.565450	0.039877
C	3.174275	1.127128	-0.699957
C	1.371344	1.449980	0.899047
C	3.512330	2.466113	-0.573502
H	3.719383	0.510501	-1.404335
C	1.719620	2.786202	1.007094
H	0.560248	1.062177	1.503049
C	2.793541	3.317307	0.277091
H	4.338075	2.858638	-1.160849
H	1.156940	3.424564	1.683046

H	3.058870	4.365562	0.365328

Int8

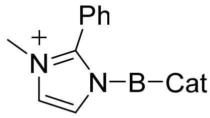

G(Ethyl acetate)=-903.0075196Hartree

C	-2.964131	-0.760017	0.038844
C	-2.458330	0.524494	-0.154860
C	-3.277038	1.625782	-0.312623
C	-4.656958	1.377731	-0.267971
C	-5.165227	0.087901	-0.074010
C	-4.320101	-1.020299	0.085419
H	-2.872067	2.619319	-0.462285
H	-5.344343	2.207916	-0.387226
H	-6.238862	-0.061885	-0.045837
H	-4.703165	-2.022331	0.236073
O	-1.899655	-1.649917	0.165995
O	-1.065242	0.469015	-0.155835
B	-0.785357	-0.857215	0.040562
C	2.098501	-3.032426	-0.014297
C	0.762602	-2.829256	0.067048
C	1.763565	-0.824257	0.028842
N	2.703047	-1.781517	-0.032953
H	2.676675	-3.940460	-0.052973
H	-0.061101	-3.521974	0.101023

C	4.154536	-1.574141	-0.064236
H	4.378333	-0.573639	0.298744
H	4.621251	-2.315204	0.583297
H	4.521610	-1.689647	-1.084907
N	0.553177	-1.448924	0.088559
C	2.003636	0.619833	0.061037
C	2.745415	1.231226	-0.961454
C	1.499995	1.386618	1.122959
C	2.976332	2.603809	-0.919562
H	3.117407	0.640888	-1.791682
C	1.739226	2.756589	1.157074
H	0.938311	0.910203	1.918501
C	2.475067	3.365343	0.137657
H	3.542488	3.077261	-1.714098
H	1.355162	3.348105	1.980878
H	2.658154	4.434254	0.167960

Int9

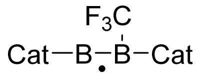

G(Ethyl acetate)=-1150.746293Hartree

B	0.731024	0.650991	-0.079192
C	-3.081155	0.108366	0.407798
C	-2.764420	-1.004447	-0.370990
C	-3.716685	-1.937549	-0.741972

C	-5.024954	-1.706829	-0.291725
C	-5.342892	-0.589654	0.489893
C	-4.367458	0.348653	0.858020
H	-3.460265	-2.798825	-1.347949
H	-5.806257	-2.411365	-0.556695
H	-6.366145	-0.443976	0.819649
H	-4.603189	1.216987	1.462263
O	-1.935096	0.852770	0.613037
O	-1.414486	-0.975065	-0.666198
B	-0.918017	0.173128	-0.052664
O	1.450988	0.205362	1.175371
O	1.495274	-0.047393	-1.176283
C	2.421588	-0.606230	0.813004
C	2.448639	-0.758904	-0.615010
C	3.340278	-1.272849	1.635161
C	3.396020	-1.579607	-1.242227
C	4.271017	-2.076683	1.000185
H	3.311503	-1.152430	2.710988
C	4.298430	-2.227644	-0.416924
H	3.408719	-1.689180	-2.319516
H	5.005351	-2.611390	1.592381
H	5.052676	-2.872144	-0.854639
C	0.930854	2.254922	-0.244883
F	2.242758	2.624248	-0.301931
F	0.350001	2.722048	-1.384399
F	0.380969	2.947822	0.786708

----

phenylacetylene

G(Ethyl acetate)=-308.4342362Hartree

C	-1.511612	-1.209009	-0.000007
C	-0.119326	-1.214021	0.000005
C	0.592556	-0.000023	0.000018
C	-0.119302	1.214009	0.000007
C	-1.511574	1.209034	-0.000009
C	-2.211210	0.000015	-0.000013
H	-2.051886	-2.150339	-0.000012
H	0.428218	-2.150331	0.000011
H	0.428289	2.150291	0.000013
H	-2.051843	2.150367	-0.000014
H	-3.296555	0.000040	-0.000024
C	2.021870	-0.000018	0.000042
C	3.232711	0.000001	0.000006
H	4.299098	0.000048	-0.000266

----

propyne

G(Ethyl acetate)=-116.6725889Hartree

C	-0.217755	0.000213	0.000008
C	-1.426383	-0.000090	-0.000001
H	-2.492562	-0.000068	-0.000015
C	1.241835	-0.000057	-0.000003

H	1.635553	0.511935	-0.884160
H	1.635559	0.509599	0.885500
H	1.635267	-1.021858	-0.001353

2-butyne

G(Ethyl acetate) = -155.9849717 Hartree

C	0.000000	-0.000000	0.605142
C	0.000000	-0.000000	-0.605142
C	-0.000000	0.000000	-2.066602
H	0.509527	0.884565	-2.464536
H	0.511292	-0.883546	-2.464536
H	-1.020819	-0.001019	-2.464536
C	-0.000000	0.000000	2.066602
H	-0.509527	0.884565	2.464536
H	-0.511292	-0.883546	2.464536
H	1.020819	-0.001019	2.464536

Int4-c

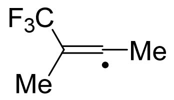

G(Ethyl acetate) = -493.7129238 Hartree

C	1.469060	-0.532441	-0.000019

C	0.458015	0.313080	0.000001
C	0.573983	1.825026	0.000026
H	0.083752	2.244855	0.884171
H	1.619090	2.136852	0.000041
H	0.083769	2.244884	-0.884116
C	2.934711	-0.549322	-0.000034
H	3.322176	-1.068588	-0.883630
H	3.349529	0.470411	0.000014
H	3.322194	-1.068677	0.883502
C	-0.948621	-0.213461	0.000004
F	-1.641230	0.223106	-1.085143
F	-1.017945	-1.559154	-0.000032
F	-1.641204	0.223046	1.085192

TS3-c

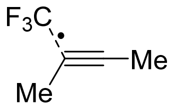

G(Ethyl acetate) = -493.6648981 Hartree

C	-0.981051	-0.376919	0.000080
C	0.899471	1.026407	-0.000153
C	1.813770	0.211083	-0.000106
C	0.155950	2.294389	-0.000243
H	-0.486377	2.370683	0.882409

H	0.845804	3.143215	-0.000404
H	-0.486526	2.370470	-0.882806
C	2.704465	-0.941583	-0.000038
H	2.118324	-1.868388	0.000110
H	3.348465	-0.949705	-0.885687
H	3.348595	-0.949503	0.885518
F	-1.720102	-0.138970	-1.092759
F	-0.586959	-1.655427	0.000281
F	-1.720041	-0.138607	1.092880

----

sulfurdioxide

G(Ethyl acetate)=-548.7181574Hartree

O	1.256051	-0.377862	-0.000000
S	0.000000	0.377489	0.000000
O	-1.256051	-0.377116	-0.000000

----

triflate anion

G(Ethyl acetate)=-961.8655504Hartree

S	0.908614	0.000018	0.000049
O	1.241765	-0.966887	1.072519
O	1.242556	1.412170	0.300857
O	1.242593	-0.445714	-1.373259
C	-0.947568	0.000085	-0.000116

F	-1.432185	0.843019	-0.930689
F	-1.432312	-1.227307	-0.264759
F	-1.431917	0.384583	1.195333

TS1

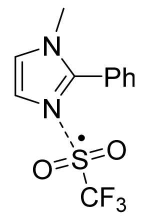

G(Ethyl acetate)=-1383.031609Hartree

S	-1.227453	-0.654775	0.887810
N	2.570369	-0.254418	1.418898
C	3.795553	-1.312330	-0.143956
C	3.568065	-1.162564	1.206463
C	2.173275	0.150613	0.210320
N	2.907141	-0.474739	-0.776361
H	4.496696	-1.913232	-0.701783
H	4.070494	-1.661074	2.023288
C	2.869538	-0.270752	-2.220697
H	2.697079	0.782073	-2.444128
H	3.833574	-0.563669	-2.636171
H	2.086589	-0.875472	-2.685620
C	1.097817	1.118331	0.016640
C	0.295185	1.177911	-1.143565
C	0.798207	1.992596	1.083201

C	-0.745628	2.102035	-1.239186
H	0.451550	0.480905	-1.955184
C	-0.246550	2.902217	0.983051
H	1.398727	1.932176	1.982575
C	-1.019310	2.967994	-0.181809
H	-1.355779	2.124227	-2.135696
H	-0.461405	3.564817	1.814968
H	-1.834547	3.679790	-0.256449
O	-0.461488	-1.930968	0.922763
O	-2.094883	-0.262460	2.033425
C	-2.472782	-0.932575	-0.541922
F	-1.788834	-1.169275	-1.660423
F	-3.213548	0.162437	-0.687270
F	-3.254902	-1.973549	-0.266671

TS2

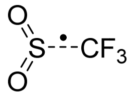

G(Ethyl acetate)=-886.401709Hartree

S	-1.790692	0.179350	-0.210150
C	1.635861	0.120994	0.154839
O	-1.291857	-1.186998	-0.028405
O	-2.952665	0.594234	0.580200
F	1.315451	0.388216	-1.108314
F	1.926500	-1.160476	0.315870

F	2.623836	0.899655	0.572334

TS3

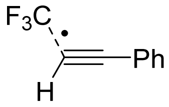

G(Ethyl acetate)=-646.1181639Hartree

C	1.162500	-0.008216	1.818390
C	2.629493	0.000960	-0.269120
C	-3.044975	1.211723	-0.360561
C	-1.823913	1.214703	0.306481
C	-1.199349	-0.002800	0.642649
C	-1.825387	-1.217196	0.298098
C	-3.046444	-1.208144	-0.368898
C	-3.659326	0.003316	-0.699694
H	-3.518908	2.153669	-0.617280
H	-1.341552	2.149263	0.571305
H	-1.344161	-2.154144	0.556478
H	-3.521517	-2.147723	-0.632101
H	-4.611704	0.005689	-1.220036
C	0.050157	-0.005906	1.322204
H	2.021030	-0.011008	2.451946
F	1.756412	0.006681	-1.273919
F	3.392499	1.094467	-0.315603
F	3.391120	-1.092969	-0.326879

TS3'

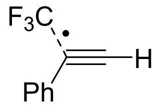

G(Ethyl acetate)=-646.1071174Hartree

C	-0.475400	1.249103	-0.464299
C	-1.974366	-0.372976	0.202332
C	2.241198	-1.348501	-0.524538
C	1.004577	-0.738479	-0.720059
C	0.783020	0.568876	-0.257561
C	1.819545	1.249480	0.406084
C	3.051727	0.630905	0.599991
C	3.266645	-0.668700	0.135604
H	2.403323	-2.357946	-0.888538
H	0.209192	-1.269454	-1.228530
H	1.645947	2.258673	0.763071
H	3.844582	1.164273	1.114600
H	4.227769	-1.149053	0.288359
C	-1.213382	2.198839	-0.723449
H	-2.036379	2.859135	-0.885193
F	-3.119873	0.248061	0.476904
F	-2.173074	-1.264438	-0.778088
F	-1.519921	-0.996613	1.291361

TS3-a

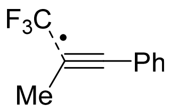

G(Ethyl acetate)=-685.4315974Hartree

C	1.184953	1.460646	-0.000305
C	2.230308	-0.770444	0.000233
C	-3.244818	-0.239302	-1.209534
C	-1.961327	0.297660	-1.215270
C	-1.300818	0.572894	-0.000158
C	-1.961608	0.298733	1.215044
C	-3.245098	-0.238232	1.209485
C	-3.890931	-0.508961	0.000020
H	-3.743248	-0.449139	-2.150638
H	-1.455310	0.507299	-2.151486
H	-1.455809	0.509201	2.151191
H	-3.743747	-0.447236	2.150659
H	-4.892123	-0.927865	0.000089
C	0.011363	1.117738	-0.000249
F	1.271267	-1.694935	0.000209
F	2.990409	-0.899828	-1.093278
F	2.990022	-0.899482	1.094052
C	2.453554	2.192660	-0.000459
H	3.048415	1.941785	0.883016
H	3.048468	1.941323	-0.883767
H	2.274598	3.272478	-0.000746

TS3-a'

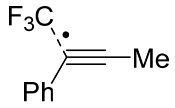

G(Ethyl acetate)=-685.4246914Hartree

C	-0.459006	0.943951	-0.223473
C	-1.608492	-1.031836	0.211796
C	2.706295	-1.035063	-0.674232
C	1.364414	-0.669296	-0.751336
C	0.918342	0.517565	-0.146268
C	1.845255	1.325987	0.537512
C	3.184584	0.952942	0.611189
C	3.620249	-0.228388	0.006270
H	3.038261	-1.953524	-1.147934
H	0.658136	-1.298807	-1.278655
H	1.501683	2.242017	1.005837
H	3.888870	1.585146	1.142803
H	4.664500	-0.518158	0.065761
C	-1.390200	1.743842	-0.314559
F	-2.850708	-0.684764	0.566716
F	-1.668486	-1.822283	-0.871756
F	-1.026758	-1.697880	1.214476
C	-2.655468	2.456753	-0.384932
H	-2.698276	3.261010	0.356931
H	-2.809912	2.899848	-1.374506
H	-3.485537	1.768064	-0.186967

TS3-b

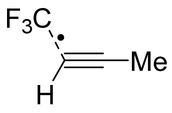

G(Ethyl acetate)=-454.3531146Hartree

C	-0.930489	1.367465	0.000017
C	1.027551	-0.024606	0.000003
C	-1.857623	0.574042	0.000009
H	-0.361345	2.271068	0.000026
F	0.574682	-1.281457	-0.000003
F	1.766776	0.188061	-1.092887
F	1.766816	0.188035	1.092871
C	-2.810991	-0.525654	-0.000002
H	-3.452840	-0.493371	0.886164
H	-3.452838	-0.493355	-0.886169
H	-2.278128	-1.483581	-0.000010

TS4

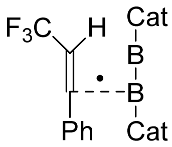

G(Ethylacetate)=-1459.196673Hartree

B	-0.120001	-1.341919	-0.296219
C	-0.264455	0.942163	-0.052634

C	3.704050	-1.022957	-0.539436
C	3.549698	-1.298621	0.819202
C	4.629365	-1.361197	1.682990
C	5.892730	-1.129868	1.119893
C	6.046765	-0.852530	-0.243959
C	4.944146	-0.793629	-1.108607
H	4.499527	-1.576226	2.737253
H	6.769349	-1.166786	1.757901
H	7.040269	-0.678190	-0.643187
H	5.051781	-0.579461	-2.165554
O	2.459189	-1.025191	-1.141389
O	2.208253	-1.478021	1.092439
B	1.547681	-1.303283	-0.121799
O	-0.828908	-1.365096	-1.522925
O	-0.961765	-1.807017	0.737701
C	-2.116748	-1.692103	-1.200047
C	-2.199401	-1.952448	0.175406
C	-3.229000	-1.752904	-2.027144
C	-3.399018	-2.274654	0.788442
C	-4.444798	-2.075364	-1.414696
H	-3.154182	-1.546037	-3.088324
C	-4.528262	-2.327564	-0.036512
H	-3.455134	-2.456218	1.854985
H	-5.344072	-2.127628	-2.019235
H	-5.491422	-2.568005	0.401026
C	-3.028920	1.049361	2.447741

C	-1.750648	0.965374	1.917496
C	-1.550392	1.104864	0.522892
C	-2.675155	1.294875	-0.317292
C	-3.949983	1.371577	0.227168
C	-4.132598	1.247370	1.607318
H	-3.174363	0.952571	3.518718
H	-0.893793	0.789212	2.557876
H	-2.522375	1.380547	-1.386750
H	-4.804464	1.519083	-0.424597
H	-5.131006	1.298896	2.029097
C	0.719752	1.653010	-0.563212
H	1.589812	1.206099	-1.034767
C	0.750151	3.154363	-0.545953
F	0.835774	3.648419	-1.805319
F	-0.333888	3.707867	0.029965
F	1.838542	3.608830	0.121431

TS4'

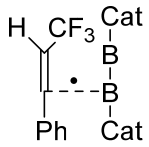

G(Ethyl acetate)=-1459.188577Hartree

B	-0.669749	0.295851	-0.943998
C	-0.321421	-0.266975	1.207332
C	3.050053	1.297882	-0.887085

C	3.131800	-0.064862	-1.179109
C	4.345004	-0.715832	-1.320121
C	5.496191	0.068494	-1.156928
C	5.414504	1.436327	-0.867851
C	4.178225	2.083408	-0.726292
H	4.395894	-1.775263	-1.542906
H	6.470896	-0.397330	-1.256555
H	6.327122	2.010599	-0.748317
H	4.104384	3.140772	-0.500074
O	1.721929	1.659017	-0.794387
O	1.856296	-0.577571	-1.279675
B	0.995983	0.492243	-1.024706
O	-1.623108	1.331244	-0.846274
O	-1.314881	-0.883383	-1.390048
C	-2.836455	0.759372	-1.107322
C	-2.648857	-0.587395	-1.443989
C	-4.095665	1.336047	-1.058450
C	-3.712891	-1.421249	-1.752056
C	-5.176598	0.502550	-1.371850
H	-4.227541	2.376014	-0.784285
C	-4.989384	-0.845616	-1.711585
H	-3.557842	-2.462800	-2.008477
H	-6.182070	0.909096	-1.347331
H	-5.853072	-1.459105	-1.945201
C	1.764766	-3.349227	1.527970
C	1.394326	-2.010527	1.486610

C	0.048021	-1.652566	1.256330
C	-0.908040	-2.668782	1.042701
C	-0.527834	-4.003899	1.091392
C	0.807270	-4.349020	1.330002
H	2.800060	-3.615604	1.714318
H	2.132239	-1.229748	1.634012
H	-1.938914	-2.395296	0.850016
H	-1.270785	-4.780471	0.941203
H	1.099863	-5.393632	1.359439
C	-0.619933	0.609901	2.149733
H	-0.688868	0.267856	3.183520
C	-0.919072	2.072874	2.004344
F	-0.198723	2.684264	1.047493
F	-2.228270	2.307476	1.745266
F	-0.640616	2.702098	3.173305

TS4-a

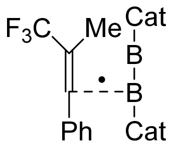

G(Ethyl acetate)=-1498.501734Hartree

B	-0.440540	-1.377995	-0.240327
C	-0.291215	0.804167	-0.277188
C	3.408681	-1.414859	-0.352410
C	3.183486	-1.185448	1.005300

C	4.221426	-1.001675	1.901873
C	5.519468	-1.056455	1.373206
C	5.745336	-1.286715	0.010714
C	4.683679	-1.473097	-0.886844
H	4.035586	-0.823916	2.954769
H	6.365937	-0.916075	2.037033
H	6.763558	-1.321366	-0.361978
H	4.847298	-1.651605	-1.943254
O	2.193223	-1.556721	-0.991830
O	1.823366	-1.179266	1.239886
B	1.223129	-1.396254	-0.003833
O	-1.111272	-1.575657	-1.478071
O	-1.344069	-1.654452	0.815888
C	-2.423079	-1.802029	-1.162679
C	-2.562601	-1.846370	0.231905
C	-3.505304	-1.960693	-2.014504
C	-3.793254	-2.050739	0.837209
C	-4.751394	-2.170534	-1.410541
H	-3.386098	-1.920917	-3.090994
C	-4.892012	-2.213370	-0.015448
H	-3.891910	-2.074102	1.916144
H	-5.627551	-2.298975	-2.037186
H	-5.875369	-2.372364	0.414305
C	-1.069749	1.506321	3.315244
C	-0.354130	1.164268	2.176316
C	-0.980400	1.201398	0.910686

C	-2.348494	1.549350	0.828308
C	-3.055189	1.884591	1.976861
C	-2.420340	1.865502	3.222401
H	-0.579663	1.489537	4.283464
H	0.683465	0.859510	2.242545
H	-2.832203	1.555612	-0.142302
H	-4.102352	2.159803	1.903566
H	-2.975171	2.124592	4.118340
C	0.262966	1.390499	-1.328034
C	0.407032	2.899947	-1.354171
F	-0.337931	3.436705	-2.354101
F	0.043813	3.517729	-0.217024
F	1.694229	3.247543	-1.603540
C	0.805397	0.733899	-2.572665
H	0.441285	1.271493	-3.453229
H	1.898265	0.775726	-2.586348
H	0.489144	-0.302502	-2.642309

TS4-a'

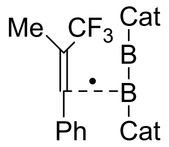

G(Ethyl acetate)=-1498.496558Hartree

B	0.261434	-0.358775	-0.976403
C	0.556213	0.196317	1.167130

C	-3.582380	-0.267891	-1.264491
C	-3.338812	0.967868	-0.664790
C	-4.359615	1.845429	-0.345100
C	-5.661940	1.427492	-0.656434
C	-5.907058	0.187926	-1.259210
C	-4.861817	-0.691413	-1.578684
H	-4.158217	2.802889	0.121340
H	-6.495942	2.081171	-0.423818
H	-6.927844	-0.101876	-1.485074
H	-5.041282	-1.653174	-2.045034
O	-2.379949	-0.916906	-1.461152
O	-1.979107	1.111839	-0.475243
B	-1.400085	-0.062738	-0.960227
O	0.878800	-1.634189	-0.875327
O	1.145420	0.521237	-1.650195
C	2.158531	-1.468967	-1.325321
C	2.319935	-0.158616	-1.796637
C	3.199718	-2.384959	-1.333624
C	3.529565	0.294330	-2.301169
C	4.423821	-1.936384	-1.844621
H	3.065408	-3.393208	-0.959483
C	4.585008	-0.625201	-2.317608
H	3.646260	1.313018	-2.651768
H	5.267218	-2.618182	-1.871439
H	5.551277	-0.314134	-2.700440
C	1.490409	3.791498	0.715793

C	0.715517	2.646642	0.843539
C	1.333126	1.396572	1.067564
C	2.743366	1.323736	1.123215
C	3.508594	2.477303	0.990750
C	2.886949	3.712249	0.786897
H	1.010297	4.751396	0.554401
H	-0.364413	2.690173	0.761806
H	3.215957	0.359319	1.273325
H	4.590711	2.413992	1.042758
H	3.486781	4.610247	0.679322
C	0.210815	-0.578240	2.184226
C	-0.518145	-1.887246	2.031393
F	-1.366062	-2.090049	3.069610
F	-1.260105	-1.987161	0.908120
F	0.345252	-2.931798	2.031010
C	0.576439	-0.249266	3.619771
H	1.194805	0.646687	3.659232
H	-0.322823	-0.090531	4.221761
H	1.132559	-1.080068	4.067407

TS5

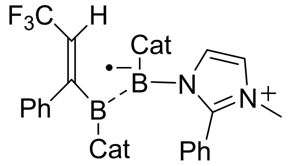

G(Ethyl acetate)=-1955.87201Hartree

B	-0.075575	-1.394094	0.568353
B	0.142350	0.304042	-1.333384
C	-1.584722	-1.116864	0.658817
C	-1.818912	0.681336	-2.266993
C	-1.524559	1.742925	-1.404109
C	-2.421758	2.767733	-1.170118
C	-3.645081	2.704208	-1.857677
C	-3.934160	1.651112	-2.729445
C	-3.017854	0.611328	-2.952025
H	-2.187787	3.574058	-0.484687
H	-4.379558	3.487540	-1.702247
H	-4.890561	1.628957	-3.241440
H	-3.244549	-0.223447	-3.603714
O	-0.763868	-0.201944	-2.293210
O	-0.264784	1.571638	-0.867612
O	0.470603	-2.542032	-0.091954
O	0.887619	-0.914579	1.510865
C	1.741874	-2.688079	0.394706
C	2.000506	-1.695380	1.348809
C	2.703195	-3.619556	0.041976
C	3.231272	-1.579004	1.971827
C	3.955432	-3.516145	0.671884
H	2.498427	-4.382152	-0.700822
C	4.215829	-2.515802	1.612879
H	3.420445	-0.797292	2.698667

H	4.734151	-4.228288	0.418821
H	5.193467	-2.461225	2.080578
C	3.302435	-1.235514	-2.228012
C	1.953266	-1.124797	-2.302615
C	2.694293	0.517234	-0.978644
N	3.752011	-0.223505	-1.402114
H	3.977434	-1.955290	-2.659059
H	1.218972	-1.722327	-2.812470
C	5.138588	-0.090203	-0.959402
H	5.178585	0.602517	-0.122368
H	5.491310	-1.068722	-0.632087
H	5.761909	0.282354	-1.774365
N	1.570571	-0.041429	-1.510457
C	2.807864	1.750517	-0.193643
C	3.651952	2.773396	-0.661569
C	2.112559	1.927368	1.012302
C	3.790551	3.954461	0.063114
H	4.179867	2.648110	-1.601260
C	2.252337	3.113917	1.726979
H	1.484072	1.132708	1.389152
C	3.088567	4.129061	1.257539
H	4.439018	4.740237	-0.310210
H	1.709858	3.240587	2.658437
H	3.194420	5.051271	1.819795
C	-1.912209	1.841761	3.031196
C	-1.473413	1.031731	1.985365

C	-2.079100	-0.210112	1.728463
C	-3.125157	-0.619738	2.575688
C	-3.558762	0.187758	3.624814
C	-2.956321	1.425682	3.858793
H	-1.434096	2.803008	3.197823
H	-0.676705	1.377435	1.342543
H	-3.596033	-1.581298	2.408875
H	-4.367231	-0.154256	4.264398
H	-3.294620	2.054962	4.676352
C	-2.404472	-1.802815	-0.194322
H	-1.975988	-2.524971	-0.879954
C	-3.867215	-1.646435	-0.369351
F	-4.609101	-2.477041	0.436779
F	-4.332508	-0.397701	-0.137055
F	-4.232197	-1.973129	-1.640355

TS6

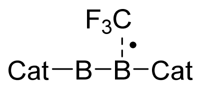

G(Ethyl acetate)=-1150.714461Hartree

B	-0.710245	-0.642614	-0.101288
C	-0.462543	1.940083	0.281360
C	3.059433	-0.379190	-0.759516
C	3.061492	-0.703189	0.597560
C	4.233549	-0.820072	1.324565

C	5.425891	-0.593097	0.623100
C	5.423823	-0.267678	-0.739407
C	4.229306	-0.153963	-1.464248
H	4.224049	-1.072406	2.378452
H	6.370946	-0.671661	1.149897
H	6.367310	-0.099141	-1.247581
H	4.216572	0.096980	-2.518433
O	1.753969	-0.335727	-1.207941
O	1.757330	-0.867594	1.021252
B	0.968102	-0.636083	-0.100057
O	-1.509105	-0.484168	-1.235857
O	-1.500445	-0.868249	1.027202
C	-2.810068	-0.580227	-0.791149
C	-2.804606	-0.812031	0.586087
C	-3.985958	-0.474734	-1.514856
C	-3.974951	-0.951529	1.313008
C	-5.177122	-0.613266	-0.790099
H	-3.978805	-0.293614	-2.583265
C	-5.171709	-0.846125	0.591922
H	-3.959571	-1.130772	2.381646
H	-6.124668	-0.537343	-1.312734
H	-6.115183	-0.947083	1.117684
F	-0.837734	2.511608	-0.851583
F	-1.327102	2.180720	1.255535
F	0.766710	2.310397	0.624253

TS7

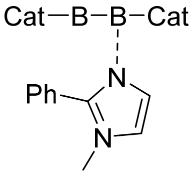

G(Ethyl acetate)=-1309.660413Hartree

B	-0.227466	-1.379602	-0.478591
N	0.047794	0.105984	1.143565
C	1.648201	-2.492451	-0.097692
C	1.895054	-1.529088	-1.082689
C	3.164622	-1.299661	-1.581834
C	4.201777	-2.091364	-1.061972
C	3.955416	-3.062289	-0.086359
C	2.662304	-3.280692	0.418141
H	3.343431	-0.531148	-2.325190
H	5.214153	-1.941020	-1.422984
H	4.778882	-3.656703	0.296263
H	2.464132	-4.024942	1.181476
O	0.311451	-2.485386	0.224925
O	0.719192	-0.902536	-1.411468
B	-1.849553	-0.964127	-0.518738
O	-2.823033	-1.453644	0.357665
O	-2.391142	0.036699	-1.332061
C	-3.967457	-0.728678	0.094347
C	-3.704597	0.177801	-0.933458

C	-5.211026	-0.817053	0.694973
C	-4.671551	1.043200	-1.414203
C	-6.200416	0.056188	0.218262
H	-5.404992	-1.525113	1.492476
C	-5.936701	0.965460	-0.812561
H	-4.456945	1.743605	-2.213187
H	-7.190721	0.023817	0.660305
H	-6.726079	1.625912	-1.155843
C	0.378836	0.528377	3.317216
C	-0.316426	-0.240742	2.422767
C	0.961687	1.072162	1.243581
N	1.187040	1.360426	2.564335
H	0.372870	0.573923	4.394650
H	-1.042319	-1.018038	2.607046
C	2.055358	2.393439	3.118742
H	2.169812	3.198499	2.394026
H	1.595670	2.790449	4.024343
H	3.040160	1.988090	3.364548
C	1.608261	1.734757	0.105024
C	2.986901	1.994351	0.090400
C	0.834896	2.069113	-1.017523
C	3.578538	2.590484	-1.022132
H	3.603159	1.699216	0.932393
C	1.430382	2.660421	-2.127480
H	-0.225888	1.845922	-1.014325
C	2.802399	2.925989	-2.132422

H	4.647146	2.779959	-1.025317
H	0.824087	2.913872	-2.991313
H	3.264210	3.388062	-2.999297

TS8

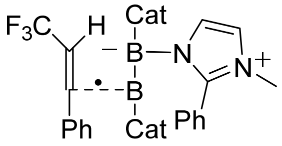

G(Ethyl acetate)=-1955.833037Hartree

B	-0.571964	-0.747404	-1.071764
C	-1.871564	0.755860	0.120370
C	1.168573	2.515412	-1.357870
C	1.385476	2.247066	0.004691
C	1.813319	3.235248	0.874890
C	2.025988	4.523225	0.347044
C	1.810567	4.790005	-1.004270
C	1.373651	3.779751	-1.882925
H	1.976274	3.015941	1.925100
H	2.361959	5.317707	1.006276
H	1.979050	5.790882	-1.389597
H	1.198473	3.978944	-2.935109
O	0.722497	1.399132	-2.002348
O	1.096264	0.949744	0.292744
B	0.795875	0.300834	-1.003919
O	-1.464113	-0.820509	-2.173585

O	-0.715952	-1.933211	-0.305028
C	-2.218710	-1.949253	-1.986010
C	-1.765895	-2.624365	-0.847323
C	-3.283392	-2.419604	-2.737625
C	-2.356324	-3.796759	-0.406190
C	-3.887433	-3.608420	-2.301873
H	-3.630825	-1.887943	-3.616232
C	-3.434496	-4.281822	-1.160846
H	-2.002232	-4.304729	0.483679
H	-4.726199	-4.010700	-2.860461
H	-3.928910	-5.196349	-0.850037
C	3.622060	-1.514841	-2.741292
C	2.481157	-0.769343	-2.726844
C	3.013153	-1.177739	-0.624570
N	3.945188	-1.766227	-1.420977
H	4.225694	-1.885872	-3.553121
H	1.905964	-0.346445	-3.532952
C	5.089731	-2.561900	-0.982036
H	4.888177	-2.961802	0.009924
H	5.233119	-3.383953	-1.683196
H	5.992682	-1.948773	-0.949764
N	2.115551	-0.564932	-1.411877
C	3.028476	-1.228954	0.845849
C	4.145997	-0.752461	1.546161
C	1.936723	-1.754636	1.550052
C	4.168878	-0.797678	2.939016

H	4.983383	-0.327453	1.002348
C	1.968640	-1.799560	2.941455
H	1.067375	-2.111451	1.011629
C	3.080592	-1.322003	3.638485
H	5.032609	-0.418627	3.475360
H	1.119089	-2.205053	3.480246
H	3.098366	-1.356435	4.723199
C	-1.497886	-0.681405	3.534219
C	-1.203074	0.012971	2.369588
C	-2.169376	0.101689	1.337427
C	-3.410524	-0.563211	1.494506
C	-3.684982	-1.260744	2.663637
C	-2.734538	-1.321130	3.688835
H	-0.759597	-0.731773	4.328322
H	-0.236915	0.477368	2.215190
H	-4.138769	-0.515566	0.692382
H	-4.641647	-1.760215	2.779219
H	-2.953467	-1.867572	4.600583
C	-2.054478	1.853637	-0.577102
H	-1.667497	1.965895	-1.585269
C	-2.732485	3.071409	-0.028977
F	-3.771487	3.449712	-0.816446
F	-3.219399	2.895772	1.216380
F	-1.884381	4.127964	0.017130
