# Supplementary material for: Integrated redox-active reagents for photoinduced regio- and stereoselective fluorocarboborylation
Source: Nat Commun. 2020 May 22;11:2572. doi: 10.1038/s41467-020-16477-1 (PMC7244735; doi:10.1038/s41467-020-16477-1)
Supplement: Supplementary file 2 — Peer Review File [file 41467_2020_16477_MOESM2_ESM.pdf]

Reviewers' comments:

Reviewer #1 (Remarks to the Author):

The difunctionalization of unsaturated carbon-carbon bond represents one of the fundamental transformations in the organic synthesis. Considerable efforts have been made in this area and impressive achievements have been achieved. In this manuscript, Wang and coworkers also describes a method for the difunctionalization of unsaturated carbon-carbon bond, namely, trifluoromethylation-borylation of alkynes/alkenes, which is a good extension to the previous works. For example, the concept of radical trifluoromethylation-borylation of alkenes is based on the previous work reported by Studer (ref 20), and the preparation of trifluoromethylating reagent IMDN-SO2CF3 derived from Tf2O as well as the generation of trifluoromethyl radical by photo-catalysis are based on the previous work reported by Qing (ref 46). Therefore, the originality of this paper is not strong. The advantage of this method is the trifluoromethylating reagent IMDN-SO2CF3 developed in the present process is bench stable and easy-to-handle. Generally, moderate to good yields trifluoromethyl-borylated products were obtained, and high stereoselectivity was observed when alkynes were used as a coupling partner. The resulting trifluoromethyl-borylated alkenes or alkanes can serve as useful building blocks for diversified transformations, which is noteworthy. Given the importance of trifluoromethyl group in the pharmaceuticals and agrochemicals and lack of efficient methods to access trifluoromethyl-borylated compounds, the paper can be published in the Nat. Commun., but the author should address the following issues.
1) In the introduction section, the authors emphasized that "the development of regio- and stereoselective installation of fluoroalkyl and boronated functionalities to unactivated hydrocarbons in sequence represents unmet challenge and urgent demand". However, the regio- and stereoselectivity of radical fluoroalkylation of aromatic alkynes is not a challenge topic. Usually, a trans-configuration was obtained when aromatic substrates were used. The challenging substrates are the internal aliphatic alkynes, but no data was shown with this type of substrate. The authors should revise the introduction and examine the internal aliphatic alkynes and internal aliphatic alkenes.
2) To demonstrate the scalability of this method, 10-g scale reaction should be performed, which will make the method more useful.

Reviewer #2 (Remarks to the Author):

The manuscript by Zhang and coworkers introduces a novel redox-active imidazolium sulfonate derived fluoroalkylation reagent for fluorocarboborylations of alkynes and alkenes. The reaction proceeds with high regio- and stereoselectivity and uses readily available starting materials. A similar strategy for 1,2-carboboration of unactivated alkenes was developed by Studer and coworkers. In this case, alkyl radicals add to the double bond and their adduct radicals engage in a formal homolytic substitution at boron of bis-boron catechol in the presence of a Lewis-basic solvent like DMF. However, these conditions were not applicable for trifluorocarboborylation of less reactive alkynes. Authors have designed a dual functional redox-active reagent, which undergoes SET to generate a perfluoroalkyl radical along with a Lewis-basic imidazole successively activating the diboron reagent for the targeted homolytic substitution step. The proposed mechanism is reasonable and in accordance to the recent literature. The substrate scope is well documented, although limitations became apparent considering aliphatic alkynes as substrates. Overall, I recommend this work for publication in Nature Communications after addressing the following minor issues:
- In the introduction the authors claim that direct the 1,2-trifluoromethylboration of alkynes has remained uncharted territory. I think this statement is unclear, especially since Pd-catalyzed fluorocarboborations of alkynes using perfluoroalkyl iodides and diboron reagents [21, 22] might also proceed via a direct radical pathway, in which the transient radical adduct undergoes radical-radical coupling with a persistent Pd(I)X species.

- Along these lines, an earlier report on Pd-catalyzed anti-selective fluoroalkylboration of internal and terminal alkynes by G. Zhu considers such direct recombination pathway and should be added to the references anyway (Org. Lett. 2018, 20, 5631).

Reviewer #3 (Remarks to the Author):

The manuscript describes fluorocarboborylation of alkynes and alkenes utilizing a new integrated redox-active reagent as a source of fluoroalkyl radical. It provides an interesting alternative to the method reported by Studer (ref. 20) utilizing gaseous CF3I to achieve borotrifluoromethylation of alkenes. Unprecedented borotrifluoromatylation of alkynes is also reported, providing a method complementary to Pd-catalyzed fluorocarboborylation enabling installation of higher homologues of fluoroalkanes (eg. C4F9). Moreover a family of new, readily available and bench stable reagents being a precursors of fluoroalkyl radical were developed. All these aspects stands for significant level of novelty of the report and makes a manuscript potentially suitable for publication in Nature Communications, however several issues have to be addressed:

- The introduction is in some points unclear, not precise or over-exaggerated should be revised and rewritten. For instance:
'Thus, the development of regio- and stereoselective installation of fluoroalkyl and boronated functionalities to unactivated hydrocarbons in sequence represents unmet challenge and urgent demand.' (lines 38-40) This challenge were addressed in few recent papers, e.g. ref. 20 (for alkenes, including installation of CF3), refs 21-22 and Org. Lett. 2018, 20, 5631−5635. (for borofluoroalkylation of alkynes). The latter paper should be cited.
'Traditional transition metal-catalyzed difunctionalization has suffered from inefficient reductive elimination of M-X intermediate and competing β-hydride elimination.' These aspects could potentially be challenging, however they were overcame and many effective methods for metal-catalyzed difunctionalization were reported. The sentences suggest that no, or only few effective methods based on metal catalysis are known, which is not true.
- Control experiments with excess of added base (e.g. imidazole, bezimidazole and/or pyridine) to show if high concentration of base have in fact detrimental effect on the reaction as authors postulate based on calculations (ref. 39).
- Several IMDN-SO2RF reagents were prepared and used for introduction of CF3 and other floroalkyl groups. However, IMDN-SO2CF2H was not shown. Extension of the scope on borodifluoromethylation would significantly strengthen the method due to high importance of difluoromethyl group.
- Are internal alkenes (eg. cyclohexene, 3-octene) compatible with the reaction conditions?
- The manuscript should be proofread. In Fig 1d, should be aerobic not areobic. Line 46 and 71 should be homolytic not hemolytic.
Issues to be addressed concerning Supporting information:
- Authors claim that chemical shifts in 1H and 13C NMR spectra were calibrated on TMS or solvents signals. Scans of MNR spectra shows that in fact they were not. For instance in 1H spectra of 1a, 1b and 1c solvents peaks appear at: 1.97, 2.05, 1.97, respectively, while in 13C at 0.90, 0.90, 1.32, respectively. (should be set at 1.94 for 1H, and 1.32 for 13C as stated in "general methods"). Reported values of chemical shifts corresponds to those taken directly from uncalibrated spectra (passed in chapter XII of SI).
- Authors claim that chemical shifts in 1H and 13C NMR spectra were calibrated on TMS or solvents signals. What about shifts in 19F NMR?
- HR MS data of compound 3n is wrong ('HRMS (ESI): caled for C15H16DBF4O2Na+ [M + Na]+ 340.1213; found 424.1209.') analysis should be repeated. By the way, there is a typo all reported mass data: is 'called', should 'calc'd' or 'calcd'
- There is a mistake in reported 1H NMR of compound 1a - 3.93 (s, 5H). Should be 3H.
- The yields were reported as both weights (to 0.1 mg) and percentages. The amount of the

limiting reagent used (alkyne or alkene) should also be reported (to 0.1 mg).
- Procedure for …. Bubbled
- Procedures VI-3 to VI-8 are specific procedures for certain compounds not General procedures.
- Most compounds seem reasonably pure, but purity of some compounds is insufficient, as shown by NMR spectra, eg. compounds: 15, 13, 9, 8, 5s, 3r and 3d. Their synthesis should be repeated, spectra of pure compounds should be reported and yields revised.

Point-by-point response to reviewers' comments

Manuscript ID: NCOMMS-20-04077

Title: Integrated redox-active reagents for photoinduced regio- and stereoselective fluorocarboborylation

Referee: 1

a) In the introduction section, the authors emphasized that "the development of regio- and stereoselective installation of fluoroalkyl and boronated functionalities to unactivated hydrocarbons in sequence represents unmet challenge and urgent demand". However, the regio- and stereoselectivity of radical fluoroalkylation of aromatic alkynes is not a challenge topic. Usually, a trans-configuration was obtained when aromatic substrates were used. The challenging substrates are the internal aliphatic alkynes, but no data was shown with this type of substrate. The authors should revise the introduction and examine the internal aliphatic alkynes and internal aliphatic alkenes.

Response: Thank you for your comments. Indeed, the issue for regio- and stereoselectivity of radical fluoroalkylation of aromatic alkynes has been well addressed. We have examined internal aliphatic alkynes and no reaction was observed. In addition, we also conducted DFT calculations using 2-butyne as a model of internal aliphatic alkynes (Scheme S3). It was found that the barrier for reaction of 2-butyne with the CF_3 radical is about 2 $kcal\cdot mol^{-1}$ higher than that for the reaction of B_2Cat_2 with the CF_3 radical. Therefore, no desired product is obtained using internal aliphatic alkynes as substrate. The internal aliphatic alkenes were tolerated. Two examples 5q and 5y were shown in Table 3 with moderate yields.

b) To demonstrate the scalability of this method, 10-g scale reaction should be performed, which will make the method more useful.

Response: A large scale reaction has been included in Table 2, using 1.02 g of phenylacetylene and 12.2 g of IMDN-SO_2CF_3 reagent 1a. The product 3a was obtained in 61% yield and >20:1 Z/E ratio.

Referee: 2

Comments: The manuscript by Zhang and coworkers introduces a novel redox-active imidazolium sulfonate derived fluoroalkylation reagent for fluorocarboborylations of alkynes and alkenes. The reaction proceeds with high regio- and stereoselectivity and uses readily available starting materials. A similar strategy for 1,2-carboboration of unactivated alkenes was developed by Studer and coworkers. In this case, alkyl radicals add to the double bond and their adduct radicals engage in a formal homolytic substitution at boron of bis-boron catechol in the presence of a Lewis-basic solvent like DMF. However, these conditions were not applicable for trifluorocarboborylation of less reactive alkynes. Authors have designed a dual functional

redox-active reagent, which undergoes SET to generate a perfluoroalkyl radical along with a Lewis-basic imidazole successively activating the diboron reagent for the targeted homolytic substitution step. The proposed mechanism is reasonable and in accordance to the recent literature. The substrate scope is well documented, although limitations became apparent considering aliphatic alkynes as substrates. Overall, I recommend this work for publication in Nature Communications after addressing the following minor issues:

a) In the introduction the authors claim that direct the 1,2-trifluoromethylboration of alkynes has remained uncharted territory. I think this statement is unclear, especially since Pd-catalyzed fluorocarboborations of alkynes using perfluoroalkyl iodides and diboron reagents [21, 22] might also proceed via a direct radical pathway, in which the transient radical adduct undergoes radical-radical coupling with a persistent Pd(I)X species.

Response: The claim in the introduction has been revised to be more appropriate.

b) Along these lines, an earlier report on Pd-catalyzed anti-selective fluoroalkylboration of internal and terminal alkynes by G. Zhu considers such direct recombination pathway and should be added to the references anyway (Org. Lett. 2018, 20, 5631).

Response: Thank you for your comments. The reference has been included in the revised manuscript.

Referee: 3

Comments: The manuscript describes fluorocarboborylation of alkynes and alkenes utilizing a new integrated redox-active reagent as a source of fluoroalkyl radical. It provides an interesting alternative to the method reported by Studer (ref. 20) utilizing gaseous CF_3I to achieve borotrifluoromethylation of alkenes. Unprecedented borotrifluoromatylation of alkynes is also reported, providing a method complementary to Pd-catalyzed fluorocarboborylation enabling installation of higher homologues of fluoroalkanes (eg. C_4F_9). Moreover a family of new, readily available and bench stable reagents being a precursors of fluoroalkyl radical were developed. All these aspects stands for significant level of novelty of the report and makes a manuscript potentially suitable for publication in Nature Communications, however several issues have to be addressed:

a) The introduction is in some points unclear, not precise or over-exaggerated should be revised and rewritten. For instance:
'Thus, the development of regio- and stereoselective installation of fluoroalkyl and boronated functionalities to unactivated hydrocarbons in sequence represents unmet challenge and urgent demand.' (lines 38-40) This challenge were addressed in few recent papers, e.g. ref. 20 (for alkenes, including installation of CF_3), refs 21-22 and Org. Lett. 2018, 20, 5631−5635. (for borofluoroalkylation of alkynes). The latter paper should be cited.

Response: Thank you for your comments. The introduction has been rephrased to be more accurate. Also, the recent papers have been included in the references.

b)'Traditional transition metal-catalyzed difunctionalization has suffered from inefficient reductive elimination of M-X intermediate and competing β-hydride elimination.' These aspects could potentially be challenging, however they were overcame and many effective methods for metal-catalyzed difunctionalization were reported. The sentences suggest that no, or only few effective methods based on metal catalysis are known, which is not true.

Response: Thank you for your comments. The introduction has been rephrased.

c) Control experiments with excess of added base (e.g. imidazole, bezimidazole and/or pyridine) to show if high concentration of base have in fact detrimental effect on the reaction as authors postulate based on calculations (ref. 39).

Response: The control experiments have been performed and addition of excess bases such as imidazole and pyridine resulted in much lower yields (See Table 1 entries 11-12).

d) Several IMDN-SO_2R_F reagents were prepared and used for introduction of CF_3 and other floroalkyl groups. However, IMDN-SO_2CF_2H was not shown. Extension of the scope on borodifluoromethylation would significantly strengthen the method due to high importance of difluoromethyl group.

Response: Thank you for your suggestion. We have attempted the synthesis of CF_2H analogue but no desired IMDN-SO_2CF_2H product was obtained with the standard procedure.

e) Are internal alkenes (eg. cyclohexene, 3-octene) compatible with the reaction conditions?

Response: Yes, the internal aliphatic alkenes were tolerated. Two examples 5q and 5y were shown in Table 3 with moderate yields.

f) The manuscript should be proofread. In Fig 1d, should be aerobic not areobic. Line 46 and 71 should be homolytic not hemolytic.

Response: We apologize for our mistakes. The typos and errors in the manuscript have been corrected.

Issues to be addressed concerning Supporting information:
g) Authors claim that chemical shifts in ^{1}H and ^{13}C NMR spectra were calibrated on TMS or solvents signals. Scans of MNR spectra shows that in fact they were not. For instance in ^{1}H spectra of 1a, 1b and 1c solvents peaks appear at: 1.97, 2.05, 1.97, respectively, while in ^{13}C at 0.90, 0.90, 1.32, respectively. (should be set at 1.94 for 1H, and 1.32 for ^{13}C as stated in "general methods"). Reported values of chemical shifts corresponds to those taken directly from uncalibrated spectra (passed in chapter XII of SI).

Response: We apologize for our mistakes. All the NMR spectra have been recalibrated and peaks corrected.

h) Authors claim that chemical shifts in ^{1}H and ^{13}C NMR spectra were calibrated on TMS or solvents signals. What about shifts in ^{19}F NMR?

Response: ^{19}F NMR is measured with 5mm BBFO SMART probe and auto-calibrated by TopSpin 4.0.6. The crude yields are determined by ^{19}F NMR using benzotrifluoride (-62.7 ppm) as an internal standard.

i) HR MS data of compound 3n is wrong ('HRMS (ESI): caled for $C_{15}H_{16}DBF_4O_2Na^+$ $[M + Na]^+$ 340.1213; found 424.1209.') analysis should be repeated. By the way, there is a typo all reported mass data: is 'called', should 'calc'd' or 'calcd'

Response: The typos have been corrected.

j) There is a mistake in reported ^{1}H NMR of compound 1a - 3.93 (s, 5H). Should be 3H.

Response: The assignment has been corrected.

k) The yields were reported as both weights (to 0.1 mg) and percentages. The amount of the limiting reagent used (alkyne or alkene) should also be reported (to 0.1 mg).

Response: The amount of the alkyne or alkene used for each reaction has been reported to 0.1 mg.

l) Procedures VI-3 to VI-8 are specific procedures for certain compounds not General procedures.

Response: The procedures have been corrected.

m) Most compounds seem reasonably pure, but purity of some compounds is insufficient, as shown by NMR spectra, eg. compounds: 15, 13, 9, 8, 5s, 3r and 3d. Their synthesis should be repeated, spectra of pure compounds should be reported and yields revised.

Response: The syntheses of the unclean compounds 15 and 3r have been repeated and characterized. 9 and 3d contain trace amount of moisture. 8 and 13 are volatile compounds and small amount of solvents in the samples cannot be completed removed. The NMR spectra of 5s contain two distereomers with 6.9:1 ratio.

REVIEWERS' COMMENTS:

Reviewer #3 (Remarks to the Author):

Authors provided satisfactory response to all comments raised by the reviewers. Therefore, the manuscript is suitable for publication.
